# Scarabaeinae dung beetles from Ecuador: a catalog, nomenclatural acts, and distribution records

**DOI:** 10.3897/zookeys.826.26488

**Published:** 2019-02-28

**Authors:** William Chamorro, Diego Marin-Armijos, Angélico senjo, Fernando Z. Vaz-De-Mello

**Affiliations:** 1 Universidad Técnica Particular de Loja, Museo de Zoología MUTPL, Departamento de Ciencias Biológicas, Universidad Técnica Particular de Loja, San Cayetano Alto s/n C.P. 11 01 608, Loja, Ecuador Universidad Técnica Particular de Loja Loja Ecuador; 2 Universidade Federal de Mato Grosso, Instituto de Biociências, Departamento de Biologia e Zoologia. Av. Fernando Correa da Costa, 2367 Boa Esperança, Cuiabá Mato Grosso, 78060-900, Brazil Universidade Federal de Mato Grosso Cuiabá Mato Grosso Brazil

**Keywords:** biomas, dung beetles, Neotropical region, taxonomic historic, type specimens

## Abstract

The Ecuadorian territory is divided into four natural regions: the coastal lowlands, the Andean highlands, the Amazon basin, and the Galapagos Islands. Each of these regions has its own ecosystems and specific vegetation. The purpose of this work is to compile an updated catalog of the Scarabaeinae (Coleoptera: Scarabaeidae) of Ecuador that includes distributional data and several nomenclatural acts. We compiled data from 25 scientific collections, with the examination of 13,550 scarabaeines; additionally, 390 bibliographic references were reviewed for their taxonomic histories. The Scarabaeinae fauna from Ecuador is represented by 33 genera and 223 species, with 45 range-restricted species; 27 species were erroneously recorded from the country. The following nomenclatural acts are made: (A) two new status as valid species: Canthon (Glaphyrocanthon) ohausi Balthasar, 1939, **stat. n.**, and Deltochilum (Calhyboma) arrowi Paulian, 1939 **stat. n.**; (B) one new synonym: *Uroxysmagnus* Balthasar, 1940 = *Uroxyslatesulcatus* Bates, 1891, **syn. n.**; (C) two revalidated names: Canthidium (Canthidium) orbiculatum (Lucas, 1857) and Dichotomius (Luederwaldtinia) fortepunctatus (Luederwaldt, 1923); and (D) ten lectotypes designated for: *Choeridiumorbiculatum* Lucas, 1857; *Choeridiumcupreum* Blanchard, 1846; *Canthidiumcoerulescens* Balthasar, 1939; *Canthonangustatus* Harold, 1867; *Canthonangustatusohausi* Balthasar, 1939; *Deltochilumtessellatum* Bates, 1870; *Pinotusfortepunctatus* Luederwaldt, 1923; *Pinotusglobulus* Felsche, 1901; *Uroxyslatesulcatus* Bates, 1891; and *Uroxysmagnus* Balthasar, 1940. The type specimens related to the new nomenclatural acts are illustrated and the maps of the geographical distribution of all species are provided.

## Introduction

Ecuador is a country located in northern South America. It has an area of 283,561 km^2^ and geographically is divided into four natural regions: the coastal lowlands, the Andean highlands, the Amazon basin, and the Galapagos Islands. The Ecuadorean territory is politically divided into 24 provinces, and it has 72 types of natural ecosystems (Sierra 1999).

Scarab beetles from the subfamily Scarabaeinae (Coleoptera: Scarabaeidae), commonly called dung beetles (in Spanish, “estercoleros”, “mierderos”, “peloteros”, or “ruedacacas”) can be found worldwide. Current estimates indicate there are more than 6500 species and 279 genera belonging to this group (Schoolmeesters et al. 2018). However, despite Ecuador’s impressive biodiversity, taxonomic research of scarab beetles in this region is scarce. Specific knowledge of Scarabaeinae is based entirely on a short list compiled by Campos (1921) in his work entitled “Studies about the entomological fauna of Ecuador” and 265 Scarabaeinae species cited by Carvajal et al. (2011) in their book “Escarabajos del Ecuador principales géneros” (Scarabs of Ecuador main genera). However, the number of species cited in the latter work is erroneous, as the authors considered numerous synonyms as valid names. Recently, Chamorro et al. (2018) provided an updated checklist for Ecuador with 33 genera, 28 subgenera, and 220 species including 19 new species records. However, no new nomenclatural acts were made.

There are several historical catalogs and checklists for Scarabaeinae that mention Ecuadorian taxa. Gemminger and Harold (1869) were the first to publish a catalog of worldwide species distribution patterns including eleven records from Ecuador. Later, Gillet (1911a) published another catalog of species distributions where the number of records for Ecuador increased to 30 dung beetle species. This was followed by Blackwelder’s (1944) checklist that compiled information about Neotropical beetles, including the West Indies, and reported 82 dung beetle species from Ecuador. Finally, Krajcik’s (2012) checklist of dung beetles reported 94 records for the country.

According to Fávila and Halffter (1997), the easy and very cheap methods to collect dung beetles, along with the advanced state of biology, taxonomy and natural history, make these insects excellent bioindicators. In Ecuador, environmental legislation associated with the ministry agreement of 2006 categorizes Scarabaeinae as an indicator group of insects used in biodiversity and environmental impact studies (TULAS 2007).

Here we present an updated catalog of the Scarabaeinae (Coleoptera: Scarabaeidae) species of Ecuador based on an extensive literature search and the examination of more than 13,500 specimens belonging to 26 collections from 13 different countries. This work aims to compile an updated catalog of the Scarabaeinae (Coleoptera: Scarabaeidae) of Ecuador, including distributional data and several nomenclatural changes.

## Materials and methods

**Plate 1.**
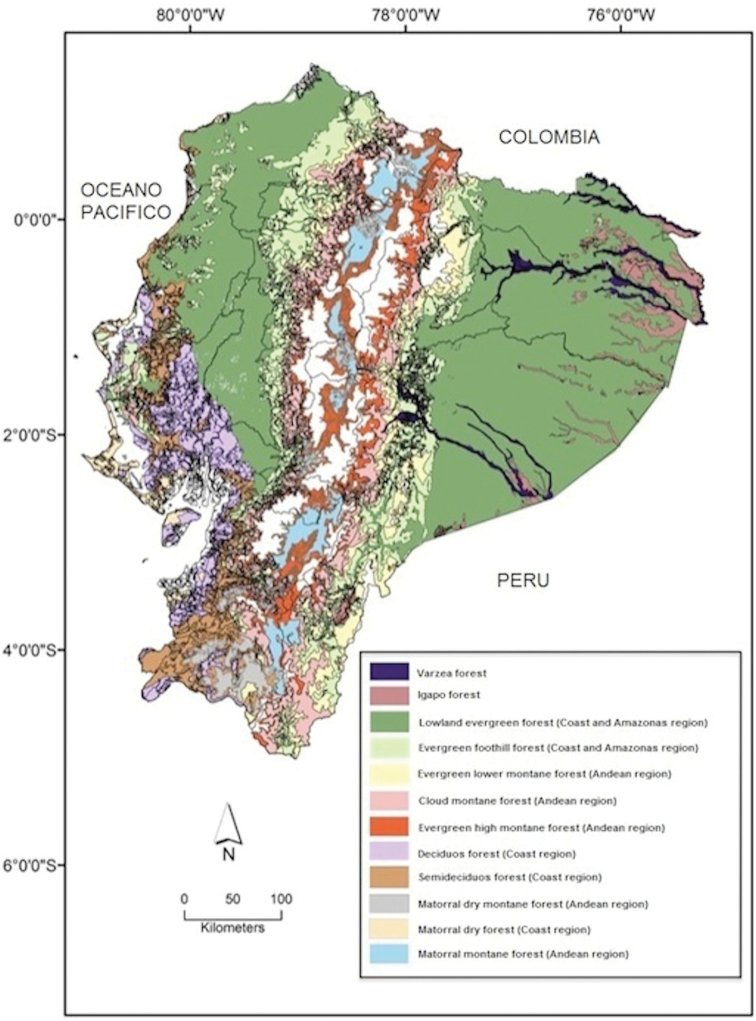
Natural ecosystems in Ecuador (Modified from Sierra 1999).

This catalog reports two sets of data for each species. The first dataset includes species’ taxonomic history including bibliographical references (e.g., original description, type species for each genus, type locality for each species, nomenclatural changes, comments, etc.). Original description and type locality data, as well as original symbols and language are maintained as they were originally published. The second dataset consist of information about the type specimens and their original labels, species occurrence outside of Ecuador, collecting localities in Ecuador and phenological data, including habitat preferences and/or biomes. For the latter, we follow Sierra’s (1999) plant formations (Plate 1). The collection method for each species is given. For some species, comments regarding nomenclatural and taxonomic changes are included following the ICZN (1999) guidelines. The specific usage of parenthesis, brackets, and punctuation are described below, together with the notes related to the spelling of the generic and species names.

**Parenthesis ()** Comments on the type of bibliographic reference.

**Brackets** [] Used to emphasize orthographic errors written in past publications (for example, the synonym of *Anomiopus* [cited as *Onomiopus*]) or to add important comments.

**Brackets and equal sign** [=] Indicates a change made for a locality, year or author (for example Ega [= Tefé]).

**Colon** : (specifically for author, year) Separates the author and specifies the page where the bibliographic reference is written.

**Semicolon** ; Separates historical bibliographic references of genera and species, but also provinces and localities in the distribution section of each species.

**Quotation marks** “ ” Emphasize an old author’s name which is not available (for example, “Müller, 1764”). Also, it indicates label data (see Type specimens).

**Comma** , Separates information among labels used in the description of type specimens.

**Slash** / Separates lines used in the description of the type specimens.

**p** Represents data printed and used in the description of the type specimens.

**hw** Represents data handwritten and used in the description of the type specimens.

The material was kindly provided by several institutions and private collections as listed below (curators name and/or type records cited in bibliographical references are shown in parenthesis):

**AMIC** Colección Antonio Martínez, Rosario de Lerma, Argentina (see Martínez 1991). Name-bearing types now in MACN.

**CECC** Colección de Escarabajos Coprófagos de Colombia, Bogotá, Colombia (see González et al. 2009).

**CEMT** Setor de Entomologia da Coleção Zoológica da Universidade Federal de Mato Grosso, Cuiabá, Brazil (Fernando Vaz-de-Mello).

**CFPL** Colección Familia Pardo-Locarno, Palmira, Colombia (Luis Carlos Pardo Locarno).

**CMNC** Canadian Museum of Nature, Ottawa, Canada (François Génier), (see Génier and Kohlmann 2003, Génier 2009, Cupello and Vaz-de-Mello 2018).

**CNCI** Canadian National Insect Collection, Agriculture et Agroalimentaire, Ottawa, Canada, (see Howden 1966).

**CPFA** Patrick Arnaud personal collection, Saintry-sur-Seine, France (see Arnaud 2000, 2002b).

**FGIC** F Génier Collection, Aylmer, Quebec, Canada (see Cook 1998).

**HAHC** A and H Howden collection Ottawa, Canada (see Arnaud 1997). Name-bearing types now in CMNC.

**IAvH** Instituto Alexander von Humboldt, Villa de Leiva, Colombia (see González et al. 2009).

**IRSN** Institut Royal des Sciences Naturelles de Belgique, Brussels, Belgium (Alain Drumont), (see Génier 1996).

**MACN** Museo Argentino de Ciencias Naturales, Buenos Aires, Argentina, formally know as Bernardino Rivadavia (Arturo Roig Alsina), (see Génier 2009, Martínez 1988b).

**MECN** Museo Ecuatoriano de Ciencias Naturales, Quito, Ecuador (Santiago Villamarín).

**MEPN** Colección Entomológica, Museo de la Escuela Politécnica Nacional, Quito, Ecuador (Vladimir Carvajal).

**MGO-UCE** Museo Laboratorio Gustavo Orces, Universidad Central, Quito, Ecuador (Fabiola Montenegro).

**MLUH** Wissenschaftsbereich Zoologie Martin-Luther-Universität, Halle, Germany (Karla Schneider).

**MNHN** Muséum National d’Histoire Naturelle, Paris, France (Olivier Montreuil and Antoine Mantilleri), (see Arnaud 1982a, Edmonds 1994, Génier 2009, Vaz-de-Mello 2008, Génier and Arnaud 2016).

**MQCAZ** Museo de Zoología de la Pontificia Universidad Católica, Quito, Ecuador (Álvaro Barragán, Carlos Carpio and Fernanda Salazar), (see Donoso et al. 2009, Génier and Kohlmann 2003).

**MSMF** Forschungsinstitut und Naturmuseum Senckenberg, Frankfurt, Germany (Damir Kovac and Andrea Hastenpflug-Vesmanis), (see Génier 2009).

**MXAL** Coleción privada Miguel Angel Morón, Xalapa, México, (see Morón 2006).

**MUTPL** Museo de Zoología de la Universidad Técnica Particular de Loja, Loja, Ecuador (Diego Marín).

**MZSP** Museu de Zoologia da Universidade de São Paulo, São Paulo, Brazil (Sonia Casari), (see Génier 1996, González et al. 2009).

**NHML** The Natural History Museum, London, United Kingdom, formally known as the British Museum (Natural History) (Maxwell VL Barclay), (see: Arnaud 2002a, Génier 1996, 2009, Kohlmann and Solís 2001, Zunino and Halffter 1988).

**NHMW** Naturhistorisches Museum Wien, Wien, Austria (see Vaz-de-Mello and Cupello 2018a).

**NHRS** Naturhistoriska Riksmuseet. Sweden, Stockholm (Johannes Bergsten and Mattias Forshage, (see Génier 2009).

**MZc** Mario Zunino private collection, Asti, Italy, (see Zunino and Halffter 1997).

**NMPC** Narodní Muzeum, Praha, Czech Republic (Jirí Hajek), (see Bezdek and Hajek 2011, 2012, 2013, Génier and Kohlmann 2003).

**OUMNH** Oxford University Museum of Natural History, Oxford, United Kingdom (Darren J. Mann).

**SMTD** Staatliches Museum für Tierkunde, Dresden, Germany (Klaus-Dieter Klass and Olaf Jaeger), (see Edmonds 1994, Edmonds and Zídek 2010, Génier 2009).

**TAMU** Texas A&M University, College Station, Texas, USA (Edward G. Riley). (see Cupello and Vaz-de-Mello 2018).

**USNM** National Museum of Natural History, Washington DC, USA (David Furth and Floyd Shockley), (see Génier 1996, Howden and Young 1981).

**ZMHB** Naturshistorisches Museum Humboldt Universität, Berlin, Germany (Johannes Frisch and Joachim Willers, (see Edmonds 1994, Edmonds and Zídek 2010, Génier 1996, 2009, Solís and Kohlmann 2002).

**ZMUC** University of Copenhagen, Zoological Museum, København, Denmark (Alexey Solodovnikov).

**ZSM** Zoologische Staatssammlung, München, Germany (Michael Balke), (see Edmonds 1994).

New lectotypes have been designated (Plates 55D, 56A–D, 57B–D, 58A–B) to fix species names over single name-bearing type specimens (see ICZN 1999, Article 46). Additionally, labels of some syntypes with records for Ecuador have been examined.

Maps of the geographical distributions are provided for each species (Plates 3–55) in a geographical-regional scale (Ecuador). Maps were generated for 825 localities using the GIS software ARCGIS, 10.1 SP1, 2012 (Plate 2). Coordinates were standardized in degrees-minutes-seconds.

## Results

A total of 13,550 scarabaeine specimens were analyzed from 26 scientific collections. We recorded a total of 223 species classified among 33 genera (Table 1) from the mainland Ecuador; no records of dung beetles from the Galapagos Islands were available. The taxonomic findings are reported as follows:

**Table 1.** Genera and species of dung beetles registered for Ecuador.

Genera	Number of species in Ecuador (this study)	Number of species worldwide (Schoolmeesters et al. 2018)
*Anomiopus* Westwood, 1842	3	60
*Ateuchus* Weber, 1801	5	98
*Bdelyrus* Harold, 1869	9	27
*Bradypodidium* Vaz-de-Mello, 2008	1	3
*Canthidium* Erichson, 1847	18	173
*Canthon* Hoffmannsegg, 1817	20	160
*Canthonella* Chapin, 1930	3 (registered species, undescribed)	17
*Copris* Geoffroy, 1762	2	251
*Coprophanaeus* d’Olsoufieff, 1924	8	50
*Cryptocanthon* Balthasar, 1942	6	38
*Deltochilum* Eschscholtz, 1822	20	106
*Dendropaemon* Perty, 1830	2	41
*Dichotomius* Hope, 1838	20	171
*Eurysternus* Dalman, 1824	15	53
*Eutrichillum* Martínez, 1969	1 (registered species, undescribed)	3
*Gromphas* Brullé, 1837	1	6
*Homocopris* Burmeister, 1846	2	3
*Malagoniella* Martínez, 1961	2	10
*Megatharsis* Waterhouse, 1891	1	1
*Onoreidium* Vaz-de-Mello, 2008	3	4
*Ontherus* Erichson, 1847	16	60
*Onthophagus* Latreille, 1802	24	2157
*Oruscatus* Bates, 1870	1	2
*Oxysternon* Castelnau, 1840	3	11
*Phanaeus* Macleay, 1819	9	61
*Scatimus* Erichson, 1847	7	12
*Scybalocanthon* Martínez, 1948	4	21
*Sinapisoma* Boucomont, 1928	1 (registered species, undescribed)	1
*Streblopus* Lansberge, 1874	1	2
*Sulcophanaeus* d’Olsoufieff, 1924	3	15
*Sylvicanthon* Halffter & Martínez, 1977	4	16
*Trichillidium* Vaz-de-Mello, 2008	1	4
*Uroxys* Westwood, 1842	12	58

(A). Two new species status: Canthon (Glaphyrocanthon) ohausi Balthasar, 1939 stat. n., and Deltochilum (Calhyboma) arrowi Paulian, 1939, stat. n.

(B). One new synonym: *Uroxysmagnus* Balthasar, 1940 = *Uroxyslatesulcatus* Bates, 1891.

(C). Two revalidated names: Canthidium (Canthidium) orbiculatum (Lucas, 1857) and Dichotomius (Luederwaldtinia) fortepunctatus (Luederwaldt, 1923).

(D). Ten new lectotypes designated: *Choeridiumorbiculatum* Lucas, 1857; *Choeridiumcupreum* Blanchard, 1846; *Canthidiumcoerulescens* Balthasar, 1939; *Canthonangustatus* Harold, 1867; *Canthonangustatusohausi* Balthasar, 1939; *Deltochilumtessellatum* Bates, 1870; *Pinotusfortepunctatus* Luederwaldt, 1923; *Pinotusglobulus* Felsche, 1901; *Uroxyslatesulcatus* Bates, 1891 and *Uroxysmagnus* Balthasar, 1940.

The following 45 species are currently known only from Ecuador:

*Ateuchusecuadorensis* (Boucomont, 1928);

*Ateuchusparvus* (Balthasar, 1939);

*Bdelyrusecuadorae* Cook, 2000;

*Bdelyrusgenieri* Cook, 1998;

*Bdelyrusparvoculus* Cook, 1998;

*Bdelyrustriangulus* Cook, 1998;

Canthidium (Canthidium) flavum Balthasar, 1939;

Canthidium (Canthidium) opacum Balthasar, 1939;

Canthidium (Canthidium) pseudaurifex Balthasar, 1939;

Canthidium (Neocanthidium) inoptatum Balthasar, 1939;

Canthidium (Neocanthidium) luteum Balthasar, 1939;

Canthon (Canthon) delicatulus Balthasar, 1939;

Canthon (Glaphyrocanthon) subhyalinoides Balthasar, 1939;

Coprophanaeus (Coprophanaeus) conocephalus (d’Olsoufieff, 1924);

*Cryptocanthoncurticrinis* Cook, 2002;

*Cryptocanthongenieri* Cook, 2002;

*Cryptocanthonnapoensis* Cook, 2002;

*Cryptocanthonotonga* Cook, 2002;

*Cryptocanthonurguensis* Cook, 2002;

Deltochilum (Aganhyboma) arturoi Silva, Louzada & Vaz-de-Mello, 2015;

Deltochilum (Deltohyboma) batesi Paulian, 1938;

Deltochilum (Deltochilum) rosamariae Martínez, 1991;

Deltochilum (Deltohyboma) speciosissimum Balthasar, 1939;

Dichotomius (Dichotomius) provisorius (Luederwaldt, 1925);

Dichotomius (Luederwaldtinia) hempeli (Pereira, 1942);

Dichotomius (Selenocopris) fonsecae (Luederwaldt, 1926);

*Onoreidiumhowdeni* (Ferreira & Galileo, 1993);

*Onoreidiumohausi* (Arrow, 1931);

Ontherus (Caelontherus) hadros Génier, 1996;

Ontherus (Caelontherus) magnus Génier, 1996;

Onthophagus (Onthophagus) dicranoides Balthasar, 1939;

Onthophagus (Onthophagus) insularis Boheman, 1858;

Onthophagus (Onthophagus) lojanus Balthasar, 1939;

*Scatimuscribrosus* Génier & Kohlmann, 2003;

*Scatimusfurcatus* Balthasar, 1939;

*Scatimusonorei* Génier & Kohlmann, 2003;

*Scybalocanthonkaestneri* (Balthasar, 1939);

*Scybalocanthonmaculatus* (Schmidt, 1920);

*Uroxysfrankenbergeri* Balthasar, 1940;

*Uroxyslatesulcatus* Bates, 1891;

*Uroxyslojanus* Arrow, 1933;

*Uroxysmonstruosus* Balthasar, 1940;

*Uroxysohausi* (Balthasar, 1938);

*Uroxysspaethi* Balthasar, 1940; and

*Uroxyssulai* Balthasar, 1940.

Finally, 27 species were erroneously recorded from Ecuador:

Canthon (Canthon) cyanellussallei Harold, 1863;

Canthon (Canthon) lituratus (Germar, 1813);

Canthon (Canthon) morsei Howden, 1966;

Canthon (Canthon) mutabilis Lucas, 1857;

Canthon (Glaphyrocanthon) rubrescens Blanchard, 1843;

Canthon (Goniocanthon) smaragdulussmaragdulus Fabricius, 1781;

Copris (Copris) incertus Say, 1835;

Copris (Copris) lugubris Boheman, 1858;

Deltochilum (Deltochilum) tumidum Howden, 1966;

Deltochilum (Deltohyboma) femorale Bates, 1870;

Deltochilum (Deltohyboma) parile Bates, 1887;

Deltochilum (Deltohyboma) spinipes Paulian, 1938;

Dichotomius (Dichotomius) alyattes Harold, 1880;

Dichotomius (Dichotomius) horridus Felsche, 1911;

Dichotomius (Dichotomius) longiceps (Taschenberg, 1870);

Dichotomius (Luederwaltinia) carbonarius Mannerheim, 1829;

*Eucraniumcyclosoma* Burmeister, 1861;

Ontherus (Ontherus) appendiculatus (Mannerheim, 1829);

Ontherus (Caelontherus) obliquus Génier, 1996;

Ontherus (Ontherus) sulcator (Fabricius, 1775);

Onthophagus (Onthophagus) clypeatus Blanchard, 1843;

Onthophagus (Onthophagus) incensus Say, 1835;

Onthophagus (Onthophagus) ophion Erichson, 1847;

*Sulcophanaeusactaeon* (Erichson, 1847);

*Sulcophanaeusnoctis* (Bates, 1887);

*Sylvicanthoncandezei* Harold, 1869; and

*Sylvicanthonaequinoctialis* (Harold, 1868).

### Genera and species records of Ecuador

#### Genus *Anomiopus* Westwood, 1842

*Anomiopus* Westwood, 1842: 59 (original description. Type species: *Anomipusvirescens* Westwood, 1842 present designation).

*Anomiopus*: Westwood 1843: 62 (redescription); Agassiz 1846: 69 (catalog, unjustifiably cited as *Anomoeopus*); Westwood 1847: 231 (redescription); Erichson 1843: 189 (list of species); Lacordaire 1856: 94 (synonym of *Onthocharis*, see footnote 2); Gemminger and Harold 1869: 1002 (catalog, synonym of *Onthocharis*); Gillet 1911a: 50 (catalog, synonym of *Onthocharis*); Lucas 1920: 100 (catalog, synonym of *Onthocharis*); Blackwelder 1944: 204 (cited as synonym of *Onthocharis* Westwood, 1847); Medina and Lopera 2000: 306 (characters in key); Vaz-de-Mello 2000: 190 (list of species of Brazil); Medina et al. 2001: 137 (list of species of Colombia); Canhedo 2006: 354 (revision); Hamel-Leigue et al. 2006: 12 (list of species of Bolívia); Vaz-de-Mello et al. 2011: 25 (characters in key); Carvajal et al. 2011: 124 (diagnosis), 318 (list of species of Ecuador); Krajcik 2012: 28 (complete list of species); Solís and Kohlmann 2012: 2 (list of species of Costa Rica); Boilly and Vaz-de-Mello 2013: 107 (characters in key); Chamorro et al. 2018: 75 (characters in key), 91 (list of species of Ecuador).

*Onthocharis* Dejean, 1833: 144 (nom. nud.); 1837: 160 (nom. nud.); Erichson 1843: 189 (nom. nud.); Agassiz 1846: 749 (catalog); Westwood 1847: 230 (cited as valid); Lacordaire 1856: 94 (description. Type species: *Onthocharissmaragdinus* (Westwood, 1842), subsequent designation); Harold 1867a: 9 (characters in key); Gemminger and Harold 1869: 1002 (complete list of species); Gillet 1911a: 50 (complete list of species); Lucas 1920: 459 (catalog, distribution); Luederwaldt 1931a: 366 (characters in key); Paulian 1938: 234 (characters in key); Pessôa and Lane 1941: 436 (characters in key); Blackwelder 1944: 204 (list of species of Latin America); Martínez 1959: 67 (catalog of species of Argentina); Pereira 1954a: 56 (characters in key); Halffter and Matthews 1966: 256 (catalog, distribution); Vulcano and Pereira 1967: 576 (characters in key); Howden and Young 1981: 13 (characters in key), 47 (redescription); Halffter and Edmonds 1982: 137 (synonym of *Anomiopus* [= cited as *Onomiopus*]).

*Hypocanthidium* Balthasar, 1938: 214 (original description. Type species: *Hypocanthidiumglobulum* Balthasar, 1938); Pereira 1954a: 56 (characters in key); Halffter and Matthews 1966: 256 (catalog, distribution); Vaz-de-Mello et al. 2011: 3 (junior synonym of *Anomiopus* Westwood, 1842); Solís and Kohlmann 2012: 2 (synonym of *Anomiopus* Westwood, 1842).

##### Anomiopus brevipes

Taxon classificationAnimaliaColeopteraScarabaeidae

(Waterhouse, 1891)

Plate 3A

Onthocharis brevipes Waterhouse, 1891a: 350 (original description. Type locality: Brazil, Amazonas, Ega [= Tefé]).
Onthocharis brevipes : Gillet 1911a: 51 (list, distribution); Blackwelder 1944: 204 (list of species of Latin America); Vulcano and Pereira 1967: 581 (list of species).
Anomiopus brevipes : Vaz-de-Mello 2000: 190 (new combination, cited for Brazil); Canhedo 2006: 362 (characters in key), 444 (redescription); Carvajal et al. 2011: 318–319 (cited for Ecuador); Krajcik 2012: 28 (complete list of species); Ratcliffe et al. 2015: 196 (cited for Peru); Chamorro et al. 2018: 84 (figures 7E–F), 91 (cited for Ecuador).

###### Type specimens.

*Onthocharisbrevipes* Waterhouse, 1891. The holotype (♂) is deposited at the NHML. Locality: Ega, examined.

**Holotype** (♂): “Type [p, red margin]”, “[one face] Ega [opposite face] 56 / . / 84 [hw, light blue label]”, “Onthocharis / brevipes, / (Type) Waterh. [hw]”, “♂ / V.L.C.1999 [hw]”, “Anomiopus / brevipes ♂ / (Waterhouse, 1891) / V.L. Canhedo det. 1999 [p, black margin]”.

###### Distribution.

Brazil, Colombia, Ecuador, and Peru.

###### Literature records.

ORELLANA: Sacha Río Coca (Canhedo 2006: 447).

###### Temporal data.

Collected in June.

###### Remarks.

Inhabits the lowland evergreen forests of the Amazon region. The collection method is unknown.

##### Anomiopus intermedius

Taxon classificationAnimaliaColeopteraScarabaeidae

(Waterhouse, 1891)

Plate 3B

Onthocharis intermedia Waterhouse, 1891a: 354 (original description. Type locality: Brazil, Amazons, Tapajos [= Rio Tapajós, Pará]).
Onthocharis intermedia : Gillet 1911a: 51 (list, distribution); Blackwelder 1944: 204 (list of species of Latin America); Vulcano and Pereira 1967: 581 (list).
Anomiopus intermedius : Vaz-de-Mello 2000: 190 (cited for Brazil); Canhedo 2006: 362 (characters in key), 452 (redescription); Carvajal et al. 2011: 318–319 (cited for Ecuador); Krajcik 2012: 28 (complete list of species, cited as Anomiopusintermedia); Ratcliffe et al. 2015: 196 (cited for Peru); Chamorro et al. 2018: 91 (cited for Ecuador).

###### Type specimens.

*Onthocharisintermedia* Waterhouse, 1891. The holotype (♀) is deposited at the NHML. Locality: Bras. Tapajos, examined.

**Holotype** (♀): “Onthocharis / intermedia / (Type) Waterh. [hw]”, “Bras / Tapajos [hw, light blue label]”, “Type [p, red margin]”, “♀ / V.L.C.1999 [hw]”, “Anomiopus / intermedius ♀ / (Waterhouse, 1891) / V.L. Canhedo 1999 [p, black margin]”.

###### Distribution.

Brazil, Ecuador, and Peru.

###### Literature records.

ORELLANA: Coca, 250 m (Canhedo 2006: 454). PASTAZA: Mera, 1100 m (Canhedo 2006: 454). SUCUMBÍOS: 2 km N of Limoncocha, 250 m (Canhedo 2006: 454); Lago Agrio, 30 km E road to Tarapoa (Canhedo 2006: 454).

###### Temporal data.

Collected in May, June, and October.

###### Remarks.

Inhabits the lowland evergreen forests and the foothill evergreen forests of the Amazon region from 250–1100 m a.s.l. The collection method is unknown.

##### Anomiopus pictus

Taxon classificationAnimaliaColeopteraScarabaeidae

(Harold, 1862)

Plate 3C

Onthocharis picta Harold, 1862: 398 (original description. Type locality: Brazil, Amazonas, Ega [= Tefé]).
Onthocharis picta : Gemminger and Harold 1869: 1002 (catalog); Gillet 1911a: 51 (catalog); Blackwelder 1944: 204 (list of species for Latin America); Vulcano and Pereira 1967: 581 (list).
Anomiopus pictus : Vaz-de-Mello 2000: 190 (cited for Brazil); Canhedo 2006: 362 (characters in key), 449 (redescription); Krajcik 2012: 28 (complete list of species, cited as Anomiopuspicta Harold, 1862); Ratcliffe et al. 2015: 196 (cited for Peru); Chamorro et al. 2018: 91 (cited for Ecuador).

###### Type specimens.

*Onthocharispicta* Harold, 1862. The holotype is deposited at the MNHN (ex coll. E Harold, ex coll. R Oberthür). Locality: Ega [= Tefé], examined.

**Holotype** (unsexed specimen): “Type [hw]”, “Ega [hw]”, “picta / Harold [hw]”, “HOLOTYPE [p, red label, black margin]”, “Ex. Museo / E. Harold [p, black margin]”, “Museum Paris / ex. Coll. / R. Oberthur [p, black margin]”.

###### Distribution.

Brazil, Ecuador, and Peru.

###### Records examined.

ORELLANA: Parque Nacional Yasuni, Río Rumiyacu Pozo Apaika, 215 m (1 specimen CEMT).

###### Temporal data.

Collected in October.

###### Remarks.

Inhabits the lowland evergreen forests of the Amazon region at 215 m a.s.l. Collected with canopy fogging methods.

#### Genus *Ateuchus* Weber, 1801

*Ateuchus* Weber, 1801: 10 (original description. Type species: *Ateuchushisteroires* Weber, 1801, by primary monotypy).

*Ateuchus*: Latreille 1829: 532 (redescription); Castelnau 1840: 63 (redescription); Reiché 1841: 212 (characters in key); Agassiz 1846: 112 (catalog); Lacordaire 1856: 66 (redescription); Lucas 1920: 125 (catalog, distribution); Pereira 1954a: 56 (characters in key); Roze 1955: 43 (list of species for Venezuela); Martínez 1959: 76 (catalog of species for Argentina); Halffter and Matthews 1966: 256 (catalog, distribution); Vulcano and Pereira 1967: 577 (characters in key, cited as *Atheuchus* Weber, 1801); Howden and Young 1981: 13 (characters in key), 68 (redescription); Halffter and Edmonds 1982: 137 (catalog, distribution); Kohlmann 1984: 25 (redescription); Kohlmann 1997: 178 (redescription); Medina and Lopera 2000: 306 (characters in key); Vaz-de-Mello 2000: 190 (list of species for Brazil); Medina et al. 2001: 137 (list of species for Colombia); Arnett et al. 2002: 49 (characters in key); Ratcliffe 2002: 13 (list of species for Panama); Morón 2003: 52 (list of species for Mexico); Hamel-Leigue et al. 2006: 12 (list of species for Bolivia); Vaz-de-Mello et al. 2011: 28 (characters in key); Carvajal et al. 2011: 126 (diagnosis), 318 (list of species for Ecuador); Krajcik 2012: 49 (complete list of species); Solís and Kohlmann 2012: 4 (list of species for Costa Rica); Boilly and Vaz-de-Mello 2013: 108 (characters in key); Chamorro et al. 2018: 77 (characters in key), 91 (list of species of Ecuador).

*Ateuchus* Fabricius, 1801: 54 (description. Type species: unnamed).

*Choeridium* Audinet-Serville, 1825: 356 (original description. Type species: *Choeridiumsimplex* Serville, 1825); Castelnau 1840: 83 (redescription); Lacordaire 1856: 93 (redescription, synonym of *Ateuchus* Fabricius); Harold 1867a: 9 (characters in key); Harold 1868a: 32 (redescription); Harold 1868b: 55 (characters in key); Gemminger and Harold 1869: 1006 (catalog); Gillet 1911a: 52 (catalog); Lucas 1920: 182 (catalog, distribution); Dawson 1922: 61 (characters in key); Luederwaldt 1929: 11 (characters in key); Luederwaldt 1931a: 369 (characters in key); Paulian 1938: 234 (characters in key); Balthasar 1939a: 44 (comment); Pessôa and Lane 1941: 437 (characters in key); Blackwelder 1944: 204 (list of species de Latin America); Chapin 1946: 79 (synonym of *Ateuchus* Weber).

##### Ateuchus aeneomicans

Taxon classificationAnimaliaColeopteraScarabaeidae

(Harold, 1868)

Plate 3D

Choeridium aeneomicans Harold, 1868c: 82 (original description. Type locality: Brazil, S Paulo [= São Paulo de Olivença], Amazon).
Choeridium aeneomicans : Harold 1868a: 37 (characters in key), 66 (redescription); Gemminger and Harold 1869: 1006 (list, distribution); Gillet 1911a: 52 (list, distribution); Balthasar 1939a: 63 (characters in key); Blackwelder 1944: 204 (list of species de Latin America).
Ateuchus aenomicans : Vulcano and Pereira 1967: 589 (characters in key); Howden and Young 1981: 68 (characters in key), 69 (redescription); Kohlmann 1997: 178 (characters in key), 179 (redescription); Vaz-de-Mello 2000: 190 (cited for Brazil); Medina et al. 2001: 137 (cited for Colombia); Ratcliffe 2002: 13 (cited for Panama); Solís and Kohlmann 2012: 4 (cited for Costa Rica); Krajcik 2012: 49 (complete list of species); Ratcliffe et al. 2015: 196 (cited for Peru); Chamorro et al. 2018: 91 (cited for Ecuador).

###### Type specimens.

*Choeridiumaeneomicans* Harold, 1868. Three syntypes examined deposited at the MNHN (ex coll. E Harold, ex coll. R Oberthür and ex coll. HW Bates). Lectotype to be designated in a future work on this species group.

###### Distribution.

Brazil, Colombia, Costa Rica, Ecuador, and Panama.

###### Records examined.

ORELLANA: Bloque 31 Timara, Parque Nacional Yasuní (1 specimen CEMT). PASTAZA: Villano (1 specimen CEMT).

###### Temporal data.

Collected in July and December.

###### Remarks.

Inhabits lowland evergreen forests of the Amazon region. Collected with pitfall traps baited with human feces.

##### Ateuchus connexus

Taxon classificationAnimaliaColeopteraScarabaeidae

(Harold, 1868)

Plate 4A

Choeridium connexum Harold, 1868a: 36 (characters in key), 55 (original description. Type locality: Ega [= Tefé]).
Choeridium connexum : Gemminger and Harold 1869: 1007 (list, distribution); Gillet 1911a: 52 (list, distribution); Balthasar 1939a: 60 (characters in key); Blackwelder 1944: 204 (list of species for Latin America); Roze 1955: 43 (list of species for Venezuela).
Ateuchus connexus : Vulcano and Pereira 1967: 589 (new combination, characters in key); Vaz-de-Mello 2000: 190 (cited for Brazil); Hamel-Leigue et al. 2006: 12 (cited for Bolivia); Krajcik 2012: 49 (complete list of species); Ratcliffe et al. 2015: 196 (cited for Peru); Chamorro et al. 2018: 91 (cited for Ecuador).

###### Type specimens.

*Choeridiumconnexum* Harold, 1868. Two syntypes examined deposited at the MNHN (ex coll. R Oberthür and ex coll. HW Bates). Lectotype to be designated in a future work on this species group.

###### Distribution.

Brazil, Bolivia, Ecuador, Peru, and Venezuela.

###### Records examined.

ORELLANA: Parque Nacional Yasuní, Estación de Biodiversidad Tiputini, 220 m USFQ (3 specimens CEMT).

###### Temporal data.

Collected in June and July.

###### Remarks.

Inhabits the lowland evergreen forests of the Amazon region at 220 m a.s.l. Collected with flight interception traps and pitfall traps baited with human feces.

##### Ateuchus ecuadorensis

Taxon classificationAnimaliaColeopteraScarabaeidae

(Boucomont, 1928)

Plate 4B

Choeridium ecuadorense Boucomont, 1928a: 191 (original description. Type locality: Ecuador, Balzapamba, Chimbo).
Choeridium ecuadorense : Balthasar 1939a: 62 (characters in key); Blackwelder 1944: 204 (list of species of Latin America).
Ateuchus ecuadorensis : Vulcano and Pereira 1967: 590 (characters in key); Krajcik 2012: 49 (complete list of species, cited as Ateuchusecuadorense); Carvajal et al. 2011: 318–319 (cited for Ecuador); Chamorro et al. 2018: 91 (cited for Ecuador).

###### Type specimens.

*Choeridiumecuadorense* Boucomont, 1928. Four syntypes examined deposited at the MNHN (ex coll. A. Boucomont). Lectotype to be designated in a future work on this species group.

**Syntype** (♂): “Equateur [hw]”, “Ch. ecuadorense. / Bouc. [hw]”, “♂ [hw, discontinuos black margin]”, “MUSEUM PARIS / 1936 / COLL. A. BOUCOMONT [p]”, “Boucomont det. 1927 / choeridium / ecuadorense n. sp [hw and p]”, “Typus [p, pink label, black double margin]”.

**Syntype** (♂): “Type [p]”, “Rosembery [hw]”, “♂ [hw, discontinuos black margin]” , “Fry Coll. / 1905.100. [p]”, [Equador / Chimbo [hw], “Boucomont det. 1927 / choeridium / ecuadorense n. sp [hw and p]”.

**Syntype** (♀): “Equateur [hw]”, “Typus [p, pink label, black double margin]”, “♀ [hw, discontinuos black margin]”, “MUSÉUM PARIS / 1936 / COLL. A. BOUCOMONT.”.

**Syntype** (♀): “Equateur [hw]”, “Typus [p, pink label, black double margin]”, “♀ [hw, discontinuos black margin]”, “MUSÉUM PARIS / 1936 / COLL. A. BOUCOMONT.”.

###### Distribution.

Only known from Ecuador.

###### Records examined.

BOLIVAR: Chimbo [= San José de Chimbo] (1 specimen MNHN). EL ORO: 3 km E de Abañin, 800 m (2 specimens CEMT); Uzhcurrumi 500 m (2 specimens CEMT). LOS RÍOS: Estación Biologica Río Palenque, 220 m (28 specimens CEMT). MANABÍ: Cabo Pasado, 0 m (9 specimens CEMT); El Carmen (2 specimens CEMT). SANTA ELENA: Olón, 10 m (82 specimens CEMT). SANTO DOMINGO DE LOS TSÁCHILAS: 7 km road to Quevedo, 550 m (1 specimen CEMT); Puerto Limón, 400 m (3 specimens CEMT). UNDETERMINED PROVINCE: without specific locality (3 specimens MNHN).

###### Literature records.

BOLIVAR: Chimbo (Boucomont 1928a: 191); Balzapamba (Boucomont 1928a: 191).

###### Temporal data.

Collected in March, May, June, August, and December.

###### Remarks.

Inhabits coastal lowland evergreen forests, coastal lowland semi-deciduous forests and coastal evergreen foothill forests from 0–800 m a.s.l. Species was collected with pitfall traps baited with human and pig feces.

##### Ateuchus parvus

Taxon classificationAnimaliaColeopteraScarabaeidae

(Balthasar, 1939)

Plate 4C

Choeridium parvum Balthasar, 1939a: 49 (original description. Type locality: Ecuador).
Choeridium parvum : Blackwelder 1944: 204 (list, distribution).
Ateuchus parvus : Vulcano and Pereira 1967: 589 (characters in key); Bezdek and Hajek 2011: 352 (catalog of types NMPC); Krajcik 2012: 50 (complete list of species); Carvajal et al. 2011: 318–319 (cited for Ecuador, misspelled name Ateuchusparvum Balthasar, 1939); Chamorro et al. 2018: 91 (cited for Ecuador).

###### Type specimens.

*Choeridiumparvum* Balthasar, 1939. The holotype (♂) is deposited at the NMPC (ex coll. V Balthasar). Locality: Quevedo, examined.

**Holotype** (♂): “W. ECUADOR / Quevedo A.M. / Jan. 08 F.v. B [p]”, “Typus [p, red label, black margin]”, “Choeridium / parvum / n. sp / Dr. V. Balthasar det. [hw and p]”, “parvum m. [hw, green label, black margin]”, “HOLOTYPE [hw, red label]”.

###### Distribution.

Only known from Ecuador.

###### Records examined.

LOS RÍOS: CCRP [= Estación Biológica Río Palenque] (18 specimens MQCAZ; 5 specimens CEMT); Quevedo (1 specimen NMPC).

###### Temporal data.

Collected in January, February, March, June, July, August, November, and December.

###### Remarks.

Inhabits coastal lowland evergreen forests at 45 m a.s.l. Species was collected with pitfall traps baited with human feces.

##### Ateuchus scatimoides

Taxon classificationAnimaliaColeopteraScarabaeidae

(Balthasar, 1939)

Plate 4D

Choeridium scatimoides Balthasar, 1939a: 47 (original description. Type locality: Ecuador, Loja, Ost-Cordill., Sabavilla [= Sabanilla, currently El Tambo]).
Choeridium scatimoides : Blackwelder 1944: 204 (list, distribution).
Ateuchus scatimoides : Medina et al. 2001: 137 (cited for Colombia); Krajcik 2012: 50 (complete list of species); Carvajal et al. 2011: 318–319 (cited for Ecuador); Ratcliffe et al. 2015: 196 (cited for Peru); Chamorro et al. 2018: 88 (figure 11F), 91 (cited for Ecuador).

###### Type specimens.

*Choeridiumscatimoides* Balthasar, 1939. Type material not examined.

###### Distribution.

Colombia and Ecuador.

###### Records examined.

ORELLANA: El Dorado plataforma Guarango, 300 m (1 specimen MUTPL); Parque Nacional Yasuní, Estación de Biodiversidad Tiputini, 220 m USFQ (1 specimen CEMT); Río Tiputini Yasuni Res. (1 specimen CEMT); Yampuna (1 specimen MQCAZ); Yuturi (1 specimen MQCAZ). PASTAZA: Bosque Protector Oglán Alto, 810 m (1 specimen CEMT; 4 specimens MUTPL; 1 specimen MGO-UC); Pandanuque, 420 m (1 specimen MUTPL). SUCUMBÍOS: La Selva Bio Station, 175 km ESE del Coca (1 specimen MQCAZ). ZAMORA CHINCHIPE: RVS El Zarza campamento las Peñas, Cordillera del Cóndor, conseción El Colibri, 1530 m (2 specimens MEPN); PN Podocarpus Bombuscaru, 1150 m (1 specimen MECN); Tundayme campamento Mirador, La Mina 1320 m (1 specimen MUTPL); Tundayme campamento Mirador, San Marcos, 900 m (1 specimen MUTPL).

###### Literature records.

LOJA: Ost-Cordill., Sabavilla [= Sabanilla, El Tambo, ZAMORA CHINCHIPE] (Balthasar 1939a: 47).

###### Temporal data.

Collected in January, February, March, May, July, September, November, and December.

###### Remarks.

Inhabits the lowland evergreen forests and the foothill evergreen forests of the Amazon region from 420–1300 m a.s.l. Species was collected with pitfall traps baited with carrion and human feces.

#### Genus *Bdelyrus* Harold, 1869

*Bdelyrus* Harold, 1869a: 97 (original description. Type species: *Bdelyruslagopus* Harold, 1869 by monotypy).

*Bdelyrus*: Gemminger and Harold 1869: 1001 (catalog); Gillet 1911a: 48 (catalog); Lucas 1920: 136 (catalog, distribution); Luederwaldt 1931a: 367 (characters in key); Paulian 1936a: 207 (characters in key); Paulian 1938: 233 (characters in key); Pessôa and Lane 1941: 436 (characters in key); Blackwelder 1944: 203 (list of species for Latin America); Pereira 1954a: 55 (characters in key); Pereira et al. 1960: 156 (biology); Halffter and Matthews 1966: 256 (catalog, distribution); Vulcano and Pereira 1967: 576 (characters in key); Halffter and Edmonds 1982: 137 (catalog, distribution); Howden and Young 1981: 12 (characters in key), 46 (redescription); Cook 1998: 632 (revision); Medina and Lopera 2000: 306 (characters in key); Vaz-de-Mello 2000: 190 (list of species for Brazil); Medina et al. 2001: 137 (list of species for Colombia); Ratcliffe 2002: 14 (list of species for Panama); Hamel-Leigue et al. 2006: 12 (list of species for Bolivia); Vaz-de-Mello et al. 2011: 21 (characters in key); Carvajal et al. 2011: 127 (diagnosis), 316 (list of species for Ecuador); Krajcik 2012: 52 (complete list of species); Solís and Kohlmann 2012: 4 (list of species for Costa Rica); Boilly and Vaz-de-Mello 2013: 106 (characters in key); 74 (characters in key), 91 (list of species of Ecuador); Chamorro et al. 2018: 74 (characters in key), 91 (list of species of Ecuador).

##### Bdelyrus ecuadorae

Taxon classificationAnimaliaColeopteraScarabaeidae

Cook, 2000

Bdelyrus ecuadorae Cook, 2000: 560 (original description. Type locality: Ecuador, Santa Jnéz [= Santa Inés]).
Bdelyrus ecuadorae : Carvajal et al. 2011: 316–317 (cited for Ecuador); Krajcik 2012: 52 (complete list of species); Chamorro et al. 2018: 91 (cited for Ecuador).

###### Type specimens.

*Bdelyrusecuadorae* Cook, 2000. The holotype (♂) is deposited at the MNHN (ex coll. R Oberthür and ex coll. A Boucomont). Locality: Santa Jnéz [= Santa Inés], examined.

**Holotype** (♂): “Bdelyrus n.sp. / det. J.Huijbregts 1984 [hw and p]”, “Museum Paris / ex Coll. / R. Oberthur [p, green label, black margin]”, “MUSEUM PARIS / Boucomont [hw and p, black margin]”, “Santa Jnéz / (Ecuad.) / R. Haerish S. [p, blak margin]”, “HOLOTYPE / Bdelyrusecuadorae / Cook [p, red label]”.

###### Distribution.

Only known from Ecuador.

###### Records examined.

UNDETERMINED PROVINCE: Santa Jnéz [= Santa Inés] (1 specimen MNHN).

###### Temporal data.

It is not known when this species was collected.

###### Remarks.

The habitat requirements and collection methods are unknown.

##### Bdelyrus genieri

Taxon classificationAnimaliaColeopteraScarabaeidae

Cook, 1998

Plate 5A

Bdelyrus genieri Cook, 1998: 646 (original description. Type locality: Ecuador, Napo Jatun Sacha, Biological Station).
Bdelyrus genieri : Cook 2000: 553–554 (characters in key); Carvajal et al. 2011: 318–319 (cited for Ecuador); Krajcik 2012: 52 (complete list of species); Chamorro et al. 2018: 91 (cited for Ecuador).

###### Type specimens.

*Bdelyrusgenieri* Cook, 1998. The holotype (♂) is deposited at the FGIC (Cook 1998: 647, [= currently deposited at the CMNC]). Locality: Napo Jatun Sacha, Biological Station, not examined.

###### Distribution.

Only known from Ecuador.

###### Records examined.

NAPO: Cotundo, 1100 m (1 specimen MUTPL). ORELLANA: Dayuma Río Rumiyacu, 290 m (1 specimen MUTPL); Onkone Gare Bloque 16 km 38.5, Parque Nacional Yasuni (1 specimen MUTPL); Pozo Záparo, 10 km NE road to Maxus Parque Nacional Yasuní (1 specimen MUTPL); road to Maxus km 80 Reserva Étnica Huaorani, 250 m, Parque Nacional Yasuní (1 specimen MUTPL); road to Maxus km 117 Iro, 250 m, Parque Nacional Yasuní (1 specimen MUTPL); Tiputini Biodiversity Station, Parque Nacional Yasuni, 220 m (1 specimen CEMT). PASTAZA: Bosque Protector Oglán Alto, 570 m (1 specimen MUTPL); Cuenca Villano, Río Villano, cabeceras, 800 m, Oleoducto km 25 (1 specimen MUTPL); Chuyayacu, río Acaro Oñampare, 515 m (1 specimen MUTPL); La Independencia (1 specimen MUTPL). SUCUMBÍOS: Cascales Rio Bermejo comuna Etza, 350 m (1 specimen MUTPL); Cuyabeno Tarapoa, Pueblo Aguas Negras 240 m (1 specimen MUTPL); Pacayacu Campo Libertador, Tapi 265 m (1 specimen MUTPL).

###### Literature records.

NAPO: Jatun Sacha, Biological Station, 21 km east of Puerto Napo (Cook 1998: 646).

###### Temporal data.

Collected in January, April, May, June, July, August, September, October, and December.

###### Remarks.

Inhabits the lowland evergreen forests and the foothill evergreen forests of the Amazon region from 240–1100 m a.s.l. Collected using both beat-sheet and canopy fogging methods.

##### Bdelyrus grandis

Taxon classificationAnimaliaColeopteraScarabaeidae

Cook, 1998

Plate 5B

Bdelyrus grandis Cook, 1998: 649 (original description. Type locality: Colombia, Amazonas, Leticia).
Bdelyrus grandis : Cook 2000: 553–554 (characters in key); Medina et al. 2001: 137 (cited for Colombia); Donoso et al. 2009: Appendix II. 16 (catalog of types MQCAZ); Carvajal et al. 2011: 318–319 (cited for Ecuador); Krajcik 2012: 52 (complete list of species); Chamorro et al. 2018: 91 (cited for Ecuador).

###### Type specimens.

*Bdelyrusgrandis* Cook, 1998. The holotype (♂) is deposited at the CNCI (Cook 1998: 650). Locality: Colombia, Amazonas, Leticia, not examined. One paratype is deposited in MQCAZ. Locality: Napo [= Sucumbíos], Cuyabeno, examined.

**Paratype** (sex unknown): “ECUADOR / NAPO Cuyabeno / IV – 1986 / legit G. Onore [hw and p, blak margin]”, “PARATYPE / Bdelyrus / grandis Cook [p, yellow label]”.

###### Distribution.

Colombia and Ecuador.

###### Records examined.

SUCUMBÍOS: Cascales Río Bermejo Comunidad ETZA, 350 m (1 specimen CEMT); Cuyabeno (1 specimen MQCAZ).

###### Temporal data.

Collected in April and August.

###### Remarks.

Inhabits the lowland evergreen forests of the Amazon region at 350 m a.s.l. Collected with canopy fogging methods.

##### Bdelyrus howdeni

Taxon classificationAnimaliaColeopteraScarabaeidae

Cook, 1998

Plate 5C

Bdelyrus howdeni Cook, 1998: 651 (original description. Type locality: Colombia, Amazonas, Leticia).
Bdelyrus howdeni : Cook 2000: 553–554 (characters in key); Medina et al. 2001: 137 (cited for Colombia); Carvajal et al. 2011: 318–319 (cited for Ecuador); Krajcik 2012: 52 (complete list of species); Ratcliffe et al. 2015: 196 (cited for Peru); Ratcliffe et al. 2015: 196 (cited for Peru); Chamorro et al. 2018: 91 (cited for Ecuador).

###### Type specimens.

*Bdelyrushowdeni* Cook, 1998. The holotype (♂) is deposited at the CMNC (ex coll. H. Howden), (see Cook 1998: 652). Locality: Amazonas, Leticia, 700 ft, not examined.

###### Distribution.

Colombia, Ecuador, and Peru.

###### Records examined.

ORELLANA: El Coca (2 specimens CEMT); Pozo Apaica, 220 m (1 specimen MUTPL); Río Capirón-Río Piraña road to Maxus km 38, 250 m, Parque Nacional Yasuní (1 specimen MUTPL); Río Rumiyacu, Parque Nacional Yasuní (1 specimen MUTPL); Río Tiputini Yasuní Res. (1 specimen CEMT); road Auca-Dayuma Río Tiputini, 350 m (1 specimen CEMT); road to Maxus km 80, Parque Nacional Yasuní, 250 m (1 specimen CEMT); road to Maxus km 117 Iro, 250 m, Parque Nacional Yasuní (1 specimen MUTPL); Záparo road to Maxus km 90, 245 m, Parque Nacional Yasuní (1 specimen CEMT). PASTAZA: B. P. Oglán, 550–655 m (2 specimens CEMT); Chuyayacu (2 specimen MUTPL); Río LLiquino Comunidad Villano, 420 m (3 specimens MUTPL); Río Villano cabeceras, Oleoducto km 25, 800 m (1 specimen CEMT). SUCUMBÍOS: Río Coca-Río Supayacu, 380 m, Parque Nacional Sumaco (1 specimen MUTPL).

###### Literature records.

NAPO: 20 km south of Tena, 600 m (Cook 1998: 652). NAPO [= ORELLANA]: Onkone Gare Camp, 220 m (Cook 1998: 652).

###### Temporal data.

Collected in January, June, July, August, September, and October.

###### Remarks.

Inhabits the lowland evergreen forests and the foothill evergreen forests of the Amazon region from 220–800 m a.s.l. Collected with canopy fogging methods.

##### Bdelyrus lobatus

Taxon classificationAnimaliaColeopteraScarabaeidae

Cook, 1998

Plate 5D

Bdelyrus lobatus Cook, 1998: 645 (original description. Type locality: Peru, Huánuco).
Bdelyrus lobatus : Cook 2000: 553 (characters in key); Krajcik 2012: 52 (complete list of species); Ratcliffe et al. 2015: 196 (cited for Peru); Chamorro et al. 2018: 91 (cited for Ecuador).

###### Type specimens.

*Bdelyruslobatus* Cook, 1998. The holotype (♂) is deposited at the CMNC (ex coll. H. Howden), (see Cook 1998: 645). Locality: Huánuco, 14 km east of Tingo Maria, not examined.

###### Distribution.

Ecuador and Peru.

###### Records examined.

PASTAZA: Bosque Protector Oglán Alto, 575 m (1 specimen CEMT); La Independencia, 1090 m (2 specimens CEMT).

###### Temporal data.

Collected in July and November.

###### Remarks.

Inhabits the foothill evergreen forests of the Amazon region from 575–1090 m a.s.l. Collected with canopy fogging methods.

##### Bdelyrus parvoculus

Taxon classificationAnimaliaColeopteraScarabaeidae

Cook, 1998

Plate 6A

Bdelyrus parvoculus Cook, 1998: 640 (original description, Type locality: Ecuador, Napo, El Reventador).
Bdelyrus parvoculus : Cook 2000: 552 (characters in key); Donoso et al. 2009: Appendix II. 16 (catalog of types QCAZ); Carvajal et al. 2011: 318–319 (cited for Ecuador); Krajcik 2012: 52 (complete list of species); Chamorro et al. 2018: 91 (cited for Ecuador).

###### Type specimens.

*Bdelyrusparvoculus* Cook, 1998. The holotype (♂) is deposited at the MQCAZ. Locality: El Reventador, examined.

**Holotype** (♂): “ECUADOR II. 88 / NAPO / El REVENTADOR / Legit: G. ONORE [hw and p, black margin]”, “HOLOTYPE / Bdelyrus / parvoculus Cook [p, red label]”.

###### Distribution.

Only known from Ecuador.

###### Record examined.

NAPO [= SUCUMBÍOS]: El Reventador (1 specimen MQCAZ).

###### Temporal data.

Collected in February.

###### Remarks.

It is possible that this species may occur in the Andean evergreen high montane forests. The collection method is unknown.

##### Bdelyrus pecki

Taxon classificationAnimaliaColeopteraScarabaeidae

Cook, 1998

Plate 6B

Bdelyrus pecki Cook, 1998: 652 (original description. Type locality: Ecuador, Pastaza, 25 km N of Puyo).
Bdelyrus pecki : Cook 2000: 553–554 (characters in key); Donoso et al. 2009: Appendix II. 16 (catalog of types MQCAZ); Carvajal et al. 2011: 318–319 (cited for Ecuador); Krajcik 2012: 52 (complete list of species); Ratcliffe et al. 2015: 196 (cited for Peru); Chamorro et al. 2018: 78 (figure 1H), 79 (figure 2A), 91 (cited for Ecuador).

###### Type specimens.

*Bdelyruspecki* Cook, 1998. The holotype (♂) is deposited at the HAHC (ex. coll. H. Howden) (see Cook 1998: 653) [= name-bearing types now in CMCN]. Locality: Ecuador, Pastaza, 25 km N of Puyo, 1000 m, not examined. One paratype is deposited in the MQCAZ. Locality: Napo, Hollín 1100 m, examined.

**Paratype** (sex unknown): “ECUADOR / NAPO / HOLLIN 1100m / 7–12–91 / F.CACERES [p]”, “PARATYPE Bdelyrus / pecki Cook [p, yellow label]”.

###### Distribution.

Brazil, Ecuador, and Peru.

###### Records examined.

NAPO: Pacto Sumaco, 1620 m (2 specimens MUTPL). Reventador (2 specimens CEMT); Sc Reventador, 1100 m (1 specimen CEMT). SUCUMBÍOS: Reventador (1 specimen CEMT). ZAMORA CHINCHIPE: RVS El Zarza campamento las Peñas conseción El Zarza, Cordillera del Cóndor, 1530 m (1 specimen MUTPL).

###### Literature records.

NAPO: km 7.3 road Sarayacu-Loreto, 1200 m (Cook 1998: 553); 12 km southwest of Tena, 500 m (Cook 1998: 553); Hollín, 1100 m (Cook 1998: 553; Donoso et al. 2009: Appendix II. 16); Río Hollín, 1100 m (Cook 1998: 553). PASTAZA: 25 km N of Puyo, 1000 m (Cook 1998: 553); 22 km southeast of Puyo, 900 m (Cook 1998: 553); Llandia 17 km N of Puyo, 1000 m (Cook 1998: 553); Puyo (Cook 1998: 553).

###### Temporal data.

Collected in January, May, June, July, September, November, and December.

###### Remarks.

Inhabits the evergreen foothill forests and evergreen lower montane forests of the Amazon region from 1000–1620 m a.s.l. Collected with flight interception traps and canopy fogging methods.

##### Bdelyrus seminudus

Taxon classificationAnimaliaColeopteraScarabaeidae

Bates, 1887

Plate 6C

Aphengium semi-nudum Bates, 1887: 42 (original description. Type locality: Nicaragua, Chontales; Panama, Volcán de Chiriquí).
Aphengium seminudum : Waterhouse 1890a: 379 (comment).
Bdelyrus seminudum : Gillet 1911a: 48 (list, distribution).
Bdelyrus seminudus : Blackwelder 1944: 203 (list of species for Latin America); Pereira et al. 1960: 156 (characters in key); Howden and Young 1981: 46 (redescription); Huijbregts 1984: 64 (distribution), 66 (characters in key); Cook 1998: 634–635 (characters in key), 656 (redescription); Cook 2000: 552–553 (characters in key); Medina et al. 2001: 137 (cited for Colombia); Ratcliffe 2002: 14 (cited for Panama); Solís and Kohlmann 2012: 4 (cited for Costa Rica); Carvajal et al. 2011: 318–319 (cited for Ecuador); Krajcik 2012: 52 (complete list of species); Chamorro et al. 2018: 91 (cited for Ecuador).

###### Type specimens.

*Aphengiumseminudum* Bates, 1887. The lectotype (♂) is deposited at the MNHN (ex coll. R Oberthür and ex coll. HW Bates). Locality: V de Chiriqui, examined.

**Lectotype** (♂): “Aphengium / seminudum Bates / lectotype dets.J.Huijbregts 1984 [hw and p]”, “♂ [p]”, “Museum Paris / ex Coll / R. Oberthur [p, green label]”, “V. de Chiriqui, / 25–4000 ft. / Champion. [p, pink label]”, “MUSEUM PARIS / COLL. H. W. BATES / 1952 [p, green label]”, “LECTOTYPE [p, red label]”.

###### Distribution.

Colombia, Costa Rica, Ecuador, and Panama.

###### Records examined.

PICHINCHA: Bosque Protector Milpe-Río Pachijal, 1200 m (1 specimen CEMT; 3 specimens MUTPL); Puerto Quito, 750 m (1 specimen CEMT); Río Guayllabamba Guayabilla-Manduriacus, 520 m (1 specimen MUTPL).

###### Literature records.

PICHINCHA: Pto. Quito (Cook 1998: 657). PICHINCHA [= SANTO DOMINGO DE LOS TSÁCHILAS]: 16 km southeast of Sto. Domingo, 500 m (Cook 1998: 657); Hba. Pupusa (Cook 1998: 657).

###### Temporal data.

Collected in April, June, October, and December.

###### Remarks.

Inhabits coastal evergreen foothill forests from 500–1200 m a.s.l. Species was collected with beat-sheet collecting and canopy fogging methods.

##### Bdelyrus triangulus

Taxon classificationAnimaliaColeopteraScarabaeidae

Cook, 1998

Plate 6D

Bdelyrus triangulus Cook, 1998: 660 (original description. Type locality: Ecuador, Napo, Sunka).
Bdelyrus triangulus : Cook 2000: 553 (characters in key); Donoso et al. 2009: Appendix II. 16 (catalog of types MQCAZ); Carvajal et al. 2011: 318–319 (cited for Ecuador); Krajcik 2012: 52 (complete list of species); Chamorro et al. 2018: 91 (cited for Ecuador).

###### Type specimens.

*Bdelyrustriangulus* Cook, 1998. The holotype (♀) is deposited at the MQCAZ. Locality: Napo [= Orellana] Sunka, examined.

**Holotype** (♀): “Ex: Hojarasca / Bosque Alto [hw]”, “ECUADOR / NAPO: SUNKA / 29-I-89 / Legit SANDOVAL”, “HOLOTYPE / Bdelyrus / triangulus Cook [p, red label]”.

###### Distribution.

Only known from Ecuador.

###### Records examined.

NAPO [= ORELLANA]: Sunka (1 specimen MQCAZ).

###### Temporal data.

Collected in January.

###### Remarks.

Inhabits the lowland evergreen forests of the Amazon region at 300 m a.s.l. According to Donoso et al. (2009) this species was collected in leaf-litter.

#### Genus *Bradypodidium* Vaz-de-Mello, 2008

*Bradypodidium* Vaz-de-Mello, 2008: 18 (original description. Type species: *Trichillumbradyporum* Boucomont, 1928 by original designation).

*Bradypodidium*: Vaz-de-Mello et al. 2011: 22 (characters in key); Carvajal et al. 2011: 316 (list of species for Ecuador); Krajcik 2012: 57 (complete list of species); Solís and Kohlmann 2012: 5 (list of species for Costa Rica); Boilly and Vaz-de-Mello 2013: 106 (characters in key); Chamorro et al. 2018: 74 (characters in key), 92 (list of species of Ecuador).

##### Bradypodidium bradyporum

Taxon classificationAnimaliaColeopteraScarabaeidae

(Boucomont, 1928)

Plate 7A

Trichillum bradyporum Boucomont, 1928a: 188 (original description. Type locality: Costa Rica, Hamburg Farm Reventezon Riv., Prov. Santa Clara).
Trichillum bradyporum : Balthasar 1939b: 17 (characters in key), 26 (redescription); Blackwelder 1944: 204 (list of species for Latin America).
Pedaridium bradyporum : Martínez 1969: 119 (comment); Ferreira and Galileo 1993: 8 (characters in key), 36 (redescription); Vaz-de-Mello et al. 2002: 676 (figure of genitalia); Solís and Kohlmann 2003: 10 (distribution), 11 (figure of dorsal habitus).
Bradypodidium bradyporum : Vaz-de-Mello 2008: 19 (distribution), 60 (figures of head and parameres); Carvajal et al. 2011: 316–317 (cited for Ecuador); Solís and Kohlmann 2012: 5 (cited for Costa Rica); Krajcik 2012: 57 (complete list of species); Boilly and Vaz-de-Mello 2013: 104 (figure 7); Chamorro et al. 2018: 80 (figure 3E), 92 (cited for Ecuador).

###### Type specimens.

*Trichillumbradyporum* Boucomont, 1928. The holotype (sex unknown) is deposited at the MNHN (see Vaz-de-Mello 2008: 19). Type locality: Hamburgfarm Reventazón, Ebene Limón, not examined.

###### Distribution.

Costa Rica and Ecuador.

###### Records examined.

ESMERALDAS: Tangareal, 125 m (1 specimen CEMT).

###### Literature records.

ESMERALDAS: 11 km SE San Lorenzo La Chiquita, 5 m (Vaz-de-Mello 2008: 19).

###### Temporal data.

Collected in March and April.

###### Remarks.

Inhabits coastal lowland evergreen forests from 5–125 m a.s.l. Collected with canopy fogging methods.

#### Genus *Canthidium* Erichson, 1847

*Canthidium* Erichson, 1847: 109 (original description. Type species: *Canthidiumthalassinum* Erichson, 1847 by subsequent designation of Martínez et al. 1964: 161).

*Canthidium*: Lacordaire 1856: 96 (redescription); Harold 1867a: 10 (characters in key, redescription); Harold 1867b: 61 (list of species, distribution); Gemminger and Harold 1869: 1004 (complete list of species); Bruch 1911: 186 (list of species for Argentina); Gillet 1911a: 54 (complete list of species); Lucas 1920: 164 (catalog, distribution); Luederwaldt 1929: 11 (characters in key); Luederwaldt 1931a: 369 (characters in key); Paulian 1938: 234 (characters in key); Pessôa and Lane 1941: 437 (characters in key); Blackwelder 1944: 205 (list of species for Latin America); Pereira 1954a: 56 (characters in key); Roze 1955: 44 (list of species for Venezuela); Martínez 1959: 72 (list of species for Argentina); Halffter and Matthews 1966: 257 (catalog, distribution); Vulcano and Pereira 1967: 577 (characters in key); Howden and Young 1981: 13 (characters in key), 71 (redescription); Halffter and Edmonds 1982: 137 (catalog, distribution); Martínez and Halffter 1986: 23 (redescription); Medina and Lopera 2000: 306 (characters in key); Vaz-de-Mello 2000: 190 (list of species for Brazil); Medina et al. 2001: 137 (list of species for Colombia); Arnett et al. 2002: 49 (characters in key); Ratcliffe 2002: 14 (list of species for Panama); Morón 2003: 54 (diagnosis); Solís and Kohlmann 2004: 5 (redescription); Hamel-Leigue et al. 2006: 12 (list of species for Bolivia); Vaz-de-Mello et al. 2011: 27 (characters in key); Carvajal et al. 2011: 128 (diagnosis), 318 (list of species for Ecuador); Krajcik 2012: 62 (complete list of species); Solís and Kohlmann 2012: 5 (list of species for Costa Rica); Boilly and Vaz-de-Mello 2013: 108 (characters in key); Cupello, 2018: 455 (list of Neotropical species); Chamorro et al. 2018: 77 (characters in key), 92 (list of species for Ecuador).

*Pleronyx* Lansberge, 1874a: 12 (original description. Type species *Pleronyxdimidiatus* Lansberge, 1874); Lucas 1920: 556 (catalog, distribution, cited as *Pteronyx*); Blackwelder 1944: 208 (list of species for Latin America, cited as *Pteronyx*); Halffter and Edmonds 1982: 137 (catalog, distribution, cited as *Pteronyx*); Vaz-de-Mello 2000: 194 (list of species for Brazil); Vaz-de-Mello et al. 2011: 3 (junior synonym of *Canthidium* Erichson, 1847); Solís and Kohlmann 2012: 2 (synonym of *Canthidium* Erichson, 1847); Cupello, 2018: 455 (synonym of *Canthidium* Erichson, 1847).

*Eucanthidium* Martínez & Halffter, 1986: 30 (original description. Type species: *Choeridiumcupreum* Blanchard, 1846), 31 (list of species of the Neotropical region); Solís and Kohlmann 2004: 7 (diagnosis), 8 (characters in key); Vaz-de-Mello et al. 2011: 27 (characters in key); Boilly and Vaz-de-Mello 2013: 109 (characters in key); Cupello, 2018: 455 (synonym of *Canthidium* Erichson, 1847); Chamorro et al. 2018: 77 (characters in key), 92 (list of species of Ecuador).

##### Subgenus Canthidium (Canthidium) Erichson, 1847

Canthidium (Canthidium) s. str. Erichson, 1847: 109 (original description. Type species: *Canthidiumthalassinum* Erichson, 1847); Martínez and Halffter 1986: 25 (redescription), 26 (list of species of the Neotropical region); Solís and Kohlmann 2004: 7 (diagnosis), 8 (characters in key); Vaz-de-Mello et al. 2011: 27 (characters in key); Boilly and Vaz-de-Mello 2013: 108 (characters in key); Cupello, 2018: 455 (list of Neotropical species); Chamorro et al. 2018: 77 (characters in key), 92 (list of species of Ecuador).

###### Canthidium (Canthidium) aurifex

Taxon classificationAnimaliaColeopteraScarabaeidae

Bates, 1887

Plate 7B

Canthidium aurifex Bates, 1887: 48 (original description. Type locality: Panama, Bugaba [= Bugabá]).
Canthidium aurifex : Gillet 1911a: 54 (list, distribution); Blackwelder 1944: 205 (list, distribution); Howden and Young 1981: 72 (characters in key), 91 (redescription); Medina et al. 2001: 137 (cited for Colombia); Ratcliffe 2002: 14 (cited for Panama); Solís and Kohlmann 2004: 11 (characters in key), 30 (redescription); Solís and Kohlmann 2012: 5 (cited for Costa Rica); Krajcik 2012: 62 (complete list of species).
Canthidium (Eucanthidium) aurifex : Martínez and Halffter 1986: 31 (cited for Ecuador, Panama, Costa Rica); Morón 2003: 55 (cited for Mexico); Carvajal et al. 2011: 318–319 (cited for Ecuador).
Canthidium (Canthidium) aurifex : Cupello 2018: 456 (transferred to the subgenus Canthidium (Canthidium) Erichson, 1847. Cited for Ecuador); Chamorro et al. 2018: 92 (cited for Ecuador).

####### Type specimens.

*Canthidiumaurifex* Bates, 1887. One syntype examined deposited at the NHML. Lectotype to be designated in a future work on this species group.

####### Distribution.

Colombia, Costa Rica, Ecuador, Mexico, and Panama.

####### Literature records.

LOS RÍOS: Río Palenque, 200 m (Howden and Young 1981: 92).

####### Temporal data.

It is not known when this species was collected.

####### Remarks.

Inhabits coastal lowland evergreen forests at 200 m a.s.l. Collection method is unknown.

###### Canthidium (Canthidium) flavum

Taxon classificationAnimaliaColeopteraScarabaeidae

Balthasar, 1939

Plate 7C

Canthidium flavum Balthasar, 1939c: 125 (original description. Type locality: Ecuador, Kordillieren).
Canthidium flavum : Vulcano and Pereira 1967: 595 (characters in key); Carvajal et al. 2011: 318–319 (cited for Ecuador). Krajcik 2012: 62 (complete list of species); Bezdek and Hajek 2012: 301 (catalog of types NMPC); Cupello, 2018: 475 (transferred to Canthidium incertae sedis, cited from Ecuador); Chamorro et al. 2018: 92 (cited for Ecuador).

####### Type specimens.

*Canthidiumflavum* Balthasar, 1939. The holotype (♀) is deposited at the NMPC (ex coll. V Balthasar). Locality: Loja Ostcordill, examined.

**Holotype** (♀): “Loja Ostcordill. / Sabanilla / F. Ohs. 2. 10. 05”, “Canthidium / flavum n. sp. / Dr. V. Balthasar det [p and hw]”, “SEM [p]”, “Typus [p, red label, black margin]”, “Mus. Nat. Pragae / 26 236 / Inv. [p and hw, orange label]”, “flavum / m. [hw, green label, black margin]’, “HOLOTYPE [hw, red label]”.

####### Distribution.

Only known from Ecuador.

####### Records examined.

LOJA: Sabanilla [= Sabanilla, El Tambo, ZAMORA CHINCHIPE] (1 specimen NMPC).

####### Temporal data.

Collected in October.

####### Remarks.

Inhabits the evergreen foothill forests of the Amazon region. Collection method is unknown.

###### Canthidium (Canthidium) funebre

Taxon classificationAnimaliaColeopteraScarabaeidae

Balthasar, 1939

Plate 7D

Canthidium funebre Balthasar, 1939c: 125 (original description. Type locality: Holländisch Guyane, Gebiet des Lucia-Flusses).
Canthidium funebre : Vulcano and Pereira 1967: 594 (characters in key); Medina et al. 2001: 137 (cited for Colombia); Krajcik 2012: 62 (complete list of species).
Canthidium (Eucanthidium) funebre : Martínez and Halffter 1986: 32 (cited for Surinam and Venezuela); Bezdek and Hajek 2012: 302 (catalog of types NMPC).
Canthidium (Canthidium) funebre : Cupello 2018: 459 (transferred to the subgenus Canthidium (Canthidium) Erichson, 1847. Cited for Ecuador); Chamorro et al. 2018: 92 (cited for Ecuador).

####### Type specimens.

*Canthidiumfunebre* Balthasar, 1939. The holotype is deposited at the NMPC (ex coll. V Balthasar). Locality: Surinam (Holländisch Guyane, Gebiet des Lucia-Flusses), examined.

**Holotype** (sex unknown): “Suriname-Exped. / Lucie-riv.-Gebied / VII-VIII. 1926 [p]”, “TYPUS [p, red label, black margin]”, “C. funebre / n. sp. m / Dr. V. Balthasar det. [p and hw]”, “funebre / m [hw, green label, blak margin]”, “HOLOTYPE [hw, red label]”.

####### Distribution.

Colombia, Ecuador, Surinam, and Venezuela.

####### Records examined.

SUCUMBÍOS: Tarapoa Campo Marian, Plataforma Fanny 18B60, 245 m (1 specimen MUTPL).

####### Temporal data.

Collected in June.

####### Remarks.

Inhabits the lowland evergreen forests of the Amazon region at 245 m a.s.l. Collected with pitfall traps baited with human feces.

###### Canthidium (Canthidium) hespenheidei

Taxon classificationAnimaliaColeopteraScarabaeidae

Howden & Young, 1981

Plate 8A

Canthidium hespenheidei Howden & Young, 1981: 92 (original description. Type locality: Panama, Panama Prov., Cerro Campana 850 m).
Canthidium hespenheidei : Ratcliffe 2002: 14 (cited for Panama); Solís and Kohlmann 2004: 16 (characters in key), 46 (redescription); Solís and Kohlmann 2012: 5 (cited for Costa Rica); Krajcik 2012: 62 (complete list of species).
Canthidium (Eucanthidium) hespenheidei : Martínez and Halffter 1986: 32 (cited for Surinam and Venezuela); Carvajal et al. 2011: 318 (cited for Ecuador).
Canthidium (Canthidium) hespenheidei : Cupello 2018: 459 (transferred to the subgenus Canthidium (Canthidium) Erichson, 1847. Cited for Ecuador); Chamorro et al. 2018: 92 (cited for Ecuador).

####### Type specimens.

*Canthidiumhespenheidei* Howden & Young, 1981. The holotype is deposited at the CMNC (ex coll. H. Howden) (see Solís and Kohlmann 2004: 46). Locality: Cerro Campaná, 850 m, Panamá, not examined.

####### Distribution.

Costa Rica, Ecuador, and Panama.

####### Literature records.

PICHINCHA [= LOS RÍOS]: 47 km S de Santo Domingo Río Palenque, 215 m (Howden and Young 1981: 93). PICHINCHA: 3 km E de Tandapi, 1310 m (Howden and Young 1981: 93). PICHINCHA [= SANTO DOMINGO DE LOS TSÁCHILAS]: Tinalandia, 650 m (Howden and Young 1981: 93).

####### Temporal data.

Collected in February and June.

####### Remarks.

Inhabits coastal lowland evergreen forests and coastal evergreen foothill forests from 215–1310 m a.s.l. According to Howden and Young (1981), this species was collected while perching on leaves at about 10–15 cm above the ground and with pitfall traps baited with human feces.

###### Canthidium (Canthidium) macroculare

Taxon classificationAnimaliaColeopteraScarabaeidae

Howden & Gill, 1987

Plate 8B

Canthidium macroculare Howden & Gill, 1987: 215 (original description. Type locality: Panama, Chiriqui, La Fortuna Dam).
Canthidium macroculare : Medina et al. 2001: 137 (cited for Colombia); Ratcliffe 2002: 14 (cited for Panama); Solís and Kohlmann 2004: 13 (characters in key), 52 (redescription); Carvajal et al. 2011: 318 (cited for Ecuador); Solís and Kohlmann 2012: 6 (cited for Costa Rica); Krajcik 2012: 62 (complete list of species).
Canthidium (Eucanthidium) macroculare : Carvajal et al. 2011: 318–319 (cited for Ecuador).
Canthidium (Canthidium) macroculare : Cupello 2018: 461 (transferred to the subgenus Canthidium (Canthidium) Erichson, 1847); Chamorro et al. 2018: 92 (cited for Ecuador).

####### Type specimens.

*Canthidiummacroculare* Howden & Gill, 1987. The holotype is deposited at the CMNC (ex coll. H Howden) (see Howden and Gill 1987: 215). Locality: Panama, Chiriqui, La Fortuna Dam, not examined.

####### Distribution.

Costa Rica, Ecuador, Mexico, and Panama.

####### Records examined.

LOS RÍOS: Estación Biológica Río Palenque, 220 m (18 specimens MQCAZ).

####### Literature records.

PICHINCHA [= LOS RÍOS]: 47 km S of Sto Domingo [= Santo Domingo de los Tsáchilas], Río Palenque, 700 m (Howden and Gill 1987: 215). PICHINCHA [= SANTO DOMINGO DE LOS TSÁCHILAS]: 16 km E Sto Domingo Tinalandia, 680 m (Howden and Gill 1987: 215).

####### Temporal data.

Collected in February, May, and July.

####### Remarks.

Inhabits coastal lowland evergreen forests and coastal evergreen foothill forests from 220–700 m a.s.l. Collected with pitfall traps baited with human feces and flight interception traps.

###### Canthidium (Canthidium) muticum

Taxon classificationAnimaliaColeopteraScarabaeidae

(Boheman, 1858)

Plate 8C

Onthophagus muticus Boheman, 1858: 48 (original description. Type locality: Insula Ohau, Honolulu).
Onthophagus muticus : Macleay 1864: 124 (redescription), Harold 1867c: 48 (comment, proposed as Canthidium Erichson, 1847).
Canthidium muticum : Harold 1867b: 88 (new combination); Gemminger and Harold 1869: 1005 (complete list of species); Gillet 1911a: 56 (complete list of species); Vulcano and Pereira 1967: 593 (characters in key); Krajcik 2012: 62 (complete list of species); Cupello, 2018: 477 (transferred to Canthidium incertae sedis); Chamorro et al. 2018: 92 (cited for Ecuador).

####### Type specimens.

*Onthophagusmuticus* Boheman, 1858. The holotype (♀) is deposited at the NRMS. Locality: Honolulu, examined.

**Holotype** (♀): “Hono- / lulu. [p]”, “Kimb [p]”, “Type [p]”, “muticum . Bhm [p]”, “Typus [p, red label, black margin]”, “129 / 62 [p and hw, red label]”, “Canthidium / muticum / Boh. [hw]”, “ 3980 / E92 + [p, blue label]”, “muticum Boh. [hw]”, “HOLOTYPE ♀ [hw, red label]”.

####### Distribution.

Colombia and Ecuador.

####### Records examined.

EL ORO: Arenillas, 15 m (126 specimens CEMT); Reserva Biológica Arenillas, 325 m (1 specimen CEMT).

####### Temporal data.

Collected in April and June.

####### Remarks.

Inhabits coastal lowland semi-deciduous forests from 15–325 m a.s.l. Collected with pitfall traps baited with human feces and light traps. The distribution of this species is limited to the lowland semi-deciduous forests of the Pacific coast of Ecuador and Colombia.

According to Bousquet (2016: 84) and subsequently Cupello (2018: 477) the reports by Boheman (1858) are possibly incorrect with regard to their type localities. Specifically, it is likely that some specimens collected in the Neotropics were mingled with others caught in the Hawaiian archipelago.

###### Canthidium (Canthidium) onitoides

Taxon classificationAnimaliaColeopteraScarabaeidae

(Perty, 1830)

Plate 8D

Onthophagus onitoides Perty, 1830: 41 (original description. Type locality: Brazilia australi, Prov. S Pauli.).
Canthidium onitoides : Harold 1867a: 31 (redescription under the new combination as C.onitoides); Harold 1867b: 79 (redescription); Gemminger and Harold 1869: 1006 (complete list of species); Gillet 1911a: 56 (complete list of species); Blackwelder 1944: 205 (list of species of Latin America); Vulcano and Pereira 1967: 594 (characters in key); Vaz-de-Mello 2000: 191 (cited for Brazil); Medina et al. 2001: 138 (cited for Colombia); Krajcik 2012: 62 (complete list of species).
Canthidium (Eucanthidium) onitoides : Martínez and Halffter 1986: 33 (cited for Brazil: Amazonas).
Canthidium (Canthidium) onitoides : Cupello 2018: 462 (transferred to the subgenus Canthidium (Canthidium) Erichson, 1847); Chamorro et al. 2018: 88 (figure 11E), 89 (figure 12 D), 92 (cited for Ecuador).
Choeridium trituberculatum Lucas, 1857: 102 (original description); Harold 1867b: 79 (cited as synonym of Canthidiumonitoides Perty, 1830); Gemminger and Harold 1869: 1006 (cited as trituberculatum Luc); Gillet 1911a: 56 (cited as trituberculatum Luc); Blackwelder 1944: 205 (cited as trituberculatum Luc); Cupello, 2018: 462 (cited for Peru).

####### Type specimens.

*Onthophagusonitoides* Perty, 1830. One syntype examined deposited at the ZSM. Lectotype to be designated in a future work on this species group.

*Choeridiumtrituberculatum* Lucas, 1857. The holotype is deposited at the MNHN. Locality: Perou Rio Ucayali, examined.

**Holotype** (sex unknown): “10 / 47 [hw]”, “Perou / Rio. / Ucayali / de Castelnau / 10-1847 [hw]”, “*Choeridium / trituberculatum* / Luc. [hw]”, “HOLOTYPE [p, red label, black margin]”.

####### Distribution.

Brazil, Colombia, and Ecuador.

####### Records examined.

ORELLANA: Ines Arango road Tiwino-río Shiripuno, 250 m (1 specimen MUTPL); Tiputini Biodiversity Station, Parque Nacional Yasuní, 215 m (1 specimen MUTPL); road to Maxus km 90 Zaparo, Parque Nacional Yasuní, 245 m (1 specimen MUTPL). PASTAZA: Bosque Protector Oglán Alto, 950 m (3 specimens MUTPL). SUCUMBÍOS: Pacayacu Campo Libertador Tapi, 265 m (1 specimen MUTPL).

####### Literature records.

PASTAZA: Sarayacu (Gemminger and Harold 1869:1006).

####### Temporal data.

Collected in January, April, July, October, and December.

####### Remarks.

Inhabits the lowland evergreen forests and the foothill evergreen forests of the Amazon region from 215–950 m a.s.l. Collected with pitfall traps baited with human feces and canopy fogging methods.

###### Canthidium (Canthidium) opacum

Taxon classificationAnimaliaColeopteraScarabaeidae

Balthasar, 1939

Plate 9A

Canthidium opacum Balthasar, 1939c: 133 (original description. Type locality: Süd-Ecuador).
Canthidium opacum : Vulcano and Pereira 1967: 595 (characters in key).
Canthidium (Eucanthidium) opacum : Martínez and Halffter 1986: 33 (cited for Ecuador); Carvajal et al. 2011: 318–319 (cited for Ecuador); Krajcik 2012: 62 (complete list of species); Bezdek and Hajek 2012: 303 (catalog of type NMPC).
Canthidium (Canthidium) opacum : Cupello 2018: 462 (transferred to the subgenus Canthidium (Canthidium) Erichson, 1847. Cited for Ecuador); Chamorro et al. 2018: 92 (cited for Ecuador).

####### Type specimens.

*Canthidiumopacum* Balthasar, 1939. The holotype is deposited at the NMPC (ex coll. V Balthasar). Locality: Landangui, examined.

**Holotype** (sex unknown): “S. ECUADOR / Landangui EW [p]”, “Typus [p, red label, black margin]”, “Canthidium / opacum / n. sp. / Dr. V. Balthasar det. [p and hw]”, “opacum / m. [hw, green label, black margin]”, “HOLOTYPE [hw, red label]”.

####### Distribution.

Only known from Ecuador.

####### Records examined.

Loja: Landangui (1 specimen NMPC).

####### Temporal data.

It is not known when this species was collected.

####### Remarks.

Inhabits the foothill evergreen forests of the Amazon region. Collection method unknown.

###### Canthidium (Canthidium) orbiculatum

Taxon classificationAnimaliaColeopteraScarabaeidae

(Lucas, 1857), revalidated name

Plates 9B , 55D , 56A

Choeridium orbiculatum Lucas, 1857: 103 (original description. Type locality: Sarayacu).
Choeridium orbiculatum : Gemminger and Harold 1869: 1006 (complete list of species); Gillet 1911a: 55 (complete list of species, synonym of Canthidiumcupreum Blanch.); Blackwelder 1944: 205 (list of species of Latin America, cited as synonym of Canthidiumcupreum Blanchard, 1843); Cupello 2018: 457 (cited as synonym of Canthidiumcupreum Blanchard, 1846).
Canthidium (Canthidium) orbiculatum : Chamorro et al. 2018: 92 (cited for Ecuador).
Chæridium cupreum Blanchard, 1846: 169 (original description. Type locality: province de Valle Grande [= Bolivia]).
Canthidium cupreum : Gemminger and Harold 1869: 1004 (transferred to the genus Canthidium Erichson, 1847. Complete list of species); Harold 1869d: 57 (cited for Bolivien [= Bolivia], comment); Gillet 1911a: 55 (complete list of species); Blackwelder 1944: 205 (list of species of Latin America); Vulcano and Pereira 1967: 595 (characters in key); Vaz-de-Mello 2000: 191 (cited as Canthidiumcupreum (Blanchard, 1843), cited for Brazil); Medina et al. 2001: 137 (cited as Canthidiumcupreum (Blanchard, 1843), cited for Colombia); Krajcik 2012: 62 (complete list of species); Ratcliffe et al. 2015: 196 (cited for Peru).
Canthidium (Eucanthidium) cupreum : Martínez and Halffter 1986: 31 (cited as Canthidium (Eucanthidium) cupreum (Blanchard), 1843. Cited for Argentina, Bolivia, and Brazil).
Canthidium (Canthidium) cupreum : Cupello 2018: 457 (transferred to the subgenus Canthidium (Canthidium) Erichson, 1847).
Canthidium aureolum Harold, 1867b: 83 (original description); Gemminger and Harold 1869: 1004 (complete list of species); Harold 1869d: 57 (synonym of Canthidiumorbiculatum Lucas, 1857, comment); Gillet 1911a: 55 (complete list of species, cited as synonym of Canthidiumcupreum Blanch.); Blackwelder 1944: 205 (list of species of Latin America, cited as synonym of Canthidiumcupreum Blanch.); Cupello 2018: 457 (cited as synonym of Canthidiumcupreum (Blanchard, 1846)).
Canthidium nitidum “Harold, 1867”a: 35 (original description); 1867b: 83 (synonym of Canthidiumaureolum Harold, 1867b); Gemminger and Harold 1869: 1004 (complete list of species, cited as synonym of Canthidiumaureolum Harold); Gillet 1911a: 55 (complete list of species, cited as synonym of Canthidiumcupreum Blanch.); Blackwelder 1944: 205 (list of species of Latin America, cited as synonym of Canthidiumcupreum Blanch.); Cupello 2018: 479 (cited as “nitidum Harold, 1867”, name not available).

####### Type specimens.

*Choeridiumorbiculatum* Lucas, 1857. The lectotype (♂) (here designated) and four paralectotypes are deposited at the MNHN. Locality: Pérou Rio. Ucayali, examined.

**Lectotype** (**here designated**) (♂): “10 / 47 [hw]”, “Choeridium / orbiculatum, / Luc. [hw]”, “Canthidium / Det. J. Huijbregts 198 [p]”, “Pérou / Rio Ucayali / de Castelnau / 10 – 47 [hw]”, “Choeridium / orbiculatum Lucas / syntype HI 1983 [hw, red label]”, “C. orbiculatum / Lucas [hw, green label]”, “LECTOTYPE ♂ / Choeridium / orbiculatum / Lucas / des. F.Z. Vaz-de-Mello, 2014 [hw and p, red label, black margin]”.

**Paralectotype** (♂): “Choeridium / orbiculatum, / Luc. [hw]”, “10 / 47 [hw]”, “Pérou / Rio Ucayali / de Castelnau / 10 – 47 [hw]”, “SYNTYPE [p, red label]”, “PARALECTOTYPE / Choeridium ♂ / orbiculatum / Lucas / des. F.Z. Vaz-de-Mello, 2014 [hw and p, yellow label, black margin]”.

**Paralectotype** (♀): “Pérou / Rio Ucayali / de Castelnau / 10 – 47 [hw]”, “10 / 47 [hw]”, “SYNTYPE [p, red label]”, “PARALECTOTYPE / Choeridium ♀ / orbiculatum / Lucas / des. F.Z. Vaz-de-Mello, 2014 [hw and p, yellow label, black margin]”.

**Paralectotype** (♂): “Pérou / Rio Ucayali / de Castelnau / 10 – 47 [hw]”, “10 / 47 [hw]”, “SYNTYPE [p, red label]”, “PARALECTOTYPE / Choeridium ♂ / orbiculatum / Lucas / des. F.Z. Vaz-de-Mello, 2014 [hw and p, yellow label, black margin]”.

**Paralectotype** (♀): “10 / 47 [hw]”, “Pérou / Rio Ucayali / de Castelnau / 10 – 47 [hw]”, “SYNTYPE [p, red label]”, “PARALECTOTYPE / Choeridium ♀ / orbiculatum / Lucas / des. F.Z. Vaz-de-Mello, 2014 [hw and p, yellow label, black margin]”.

*Choeridiumcupreum* Blanchard, 1846. The lectotype (♂) (here designated) is deposited at the MNHN. Locality: Valle Grande, Santa Cruz. Examined.

**Lectotype** (**here designated**) (♂): “G3 14 / 84 [hw]”, “MUSEUM PARIS / SANTA-CRUZ / (VALLE GRANDE) / D’Orbigny 1834 [p]”, “C. Cupreum / Blanch. / Valle-Grande / M. D’Orbigny. [hw, green label]”, “Choeridium / cupreum Blanchard / .J. Huijbregts 1983 det [hw and p]”, “LECTOTYPE [p]”, “LECTOTYPE ♂ / Choeridium / cupreum / Blanch. / des. F.Z. Vaz-de-Mello, 2014 [hw and p, red label, black margin]”.

*Canthidiumaureolum* Harold, 1867. Location of syntypes unknown, possibly deposited at the MNHN (ex coll. E Harold), (see Cupello 2018, 457). Not examined.

*Canthidiumnitidum* Harold, 1867. Type material not examined.

####### Distribution.

Colombia, Ecuador, and Peru.

####### Records examined.

ORELLANA: Estación Científica Yasuní PUCE, Parque Nacional Yasuní, 220 m (5 specimens CEMT); Rodrigo Borja IAMOE (9 specimens CEMT). SUCUMBÍOS: Comunidad Cofanes Río Zábalo, 280 m (1 specimen CEMT); Limoncocha (1 specimen CEMT).

####### Temporal data.

Collected in February, May, June, July, August, September, October, and November.

####### Remarks.

Inhabits the lowland evergreen forests of the Amazon region from 220–390 m a.s.l. Collected with pitfall traps baited with human feces.

Lucas (1857) described the species *Choeridiumorbiculatum* (type locality: de la mission de Sarayacu, Peru). Subsequently, Gillet (1911a) synonymized it, without any explanation, with *Canthidiumcupreum* (Blanchard, 1846) (a species described from Bolivia, Valle Grande). However, upon examining the external morphology (specifically, punctation on pronotal disc, elytral microsculpture and dorsal coloration) of the type specimens of *C.orbiculatum* (lectotype ♂ here designated, deposited at the MNHN, Plate 55D) and *C.cupreum* (lectotype ♂ here designated, deposited at the MNHN, Plate 56A), we could confirm they belong to the two distinct species. Therefore, maintaining the specific name originally proposed by Lucas, we revalidate it as Canthidium (Canthidium) orbiculatum (Lucas, 1857). Two lectotypes (♂, with localities: Pérou [= Peru] Rio Ucayali and Valle Grande) are designated and illustrated here (Plate 55D and Plate 56A) in order to fix the name over a single name-bearing type.

###### Canthidium (Canthidium) pseudaurifex

Taxon classificationAnimaliaColeopteraScarabaeidae

Balthasar, 1939

Plate 9D

Canthidium pseudaurifex Balthasar, 1939c: 136 (original description. Type locality: Ecuador, Prov. Los Rios).
Canthidium pseudaurifex : Vulcano and Pereira 1967: 596 (characters in key); Carvajal et al. 2011: 318–319 (cited for Ecuador); Krajcik 2012: 62 (complete list of species); Bezdek and Hajek 2012: 304 (catalog of type NMPC); Cupello, 2018: 477 (transferred to Canthidium incerta sedis, cited from Ecuador); Chamorro et al. 2018: 92 (cited for Ecuador).

####### Types specimens.

*Canthidiumpseudaurifex* Balthasar, 1939. The holotype (♂) is deposited at the NMPC. Locality: Babahoyo. Examined.

**Holotype** (♂): “Babahoyo / 7. 05 O. v. B. [p]”, “Typus [p, red label, black margin]”, “C. pseudauri- / fex n. sp. / Dr. V. Balthasar det. [hw and p]”, “pseudaurifex / m. [hw, green label, black margin]”, “HOLOTYPE [hw, red label]”.

####### Distribution.

Only known from Ecuador.

####### Records examined.

ESMERALDAS: Calle Mansa (2 specimens CEMT); Chispero (2 specimens CEMT); Colón del Ónzole (6 specimens CEMT); Gualpi (1 specimen CEMT); Padre Santo, Playa de Oro (6 specimens CEMT); Palma Real (2 specimens CEMT). IMBABURA: Río Guayllabamba alrededores de Pacto, 700–1150 m (2 specimens CEMT). LOS RÍOS: Babahoyo (1 specimen NMPC); Río Palenque Station (15 specimens CEMT). SANTA ELENA: Olón, 50 m (70 specimens CEMT).

####### Temporal data.

Collected in January, February, March, April, May, June, July, August, and December.

####### Remarks.

Inhabits coastal lowland evergreen forests and coastal evergreen foothill forests from 50–1150 m a.s.l. Collected with pitfall traps baited with human and pig feces.

###### Canthidium (Canthidium) rufinum

Taxon classificationAnimaliaColeopteraScarabaeidae

Harold, 1867

Plate 9C

Canthidium rufinum Harold, 1867b: 79 (original description. Type locality: Columbien [= Colombia], Costa Rica, und Bogotá, auch vom oberen Amazonenstrom).
Canthidium rufinum : Gemminger and Harold 1869: 1006 (complete list of species); Gillet 1911a: 56 (complete list of species); Blackwelder 1944: 205 (list of species de Latin America); Contreras 1951: 221 (cited for Colombia); Vulcano and Pereira 1967: 594 (characters in key); Vaz-de-Mello 2000: 191 (cited for Brazil); Medina et al. 2001: 138 (cited for Colombia); Hamel-Leigue et al. 2006: 12 (cited for Bolivia); Krajcik 2012: 63 (complete list of species).
Canthidium (Eucanthidium) rufinum : Martínez and Halffter 1986: 33 (cited for Bolivia and Colombia).
Canthidium (Canthidium) rufinum : Cupello 2018: 464 (transferred to the subgenus Canthidium (Canthidium) Erichson, 1847); Chamorro et al. 2018: 92 (cited for Ecuador).

####### Type specimens.

*Canthidiumrufinum* Harold, 1867. Six syntypes examined deposited at the MNHN (ex coll. E Harold, ex coll. HW Bates and ex coll. R Oberthur). Lectotype to be designated in a future work on this species group.

####### Distribution.

Bolivia, Brazil, Colombia, and Ecuador.

####### Records examined.

ORELLANA: Río Tiputini, Parque Nacional Yasuní 250 (1 specimen CEMT); Yasuní (1 specimen CEMT). SUCUMBÍOS: 6 km de Dureno, Precooperativa Los Vergeles, 300 m (1 specimen CEMT).

####### Temporal data.

Collected in August and November.

####### Remarks.

Inhabits the lowland evergreen forests of the Amazon region from 250–300 m a.s.l. Collected with pitfall traps baited with carrion.

##### Subgenus Canthidium (Neocanthidium) Martínez, Halffter & Pereira, 1964

Canthidium (Neocanthidium) Martínez, Halffter & Pereira 1964: 166 (original description. Type species: *Neocanthidiumbokermanni* Martínez, Halffter & Pereira, 1964 by original designation).

Canthidium (Neocanthidium): Halffter and Matthews 1966: 257 (catalog, distribution); Howden and Young 1981: 71 (synonym of *Canthidium* Erichson, 1847); Halffter and Edmonds 1982: 137 (catalog, distribution); Martínez and Halffter 1986: 22 (invalid name for *Neocanthidium* Martínez, Halffter & Pereira, 1964); Ratcliffe 2002: 14 (synonym of *Canthidium* Erichson, 1847); Solís and Kohlmann 2012: 5 (synonym of *Canthidium* Erichson, 1847); Cupello, 2018: 455 (revalidated as subgenus of *Canthidium* Erichson, 1847), 465 (list of species of the Neotropical region); Chamorro et al. 2018: 77 (characters in key), 92 (list of species of Ecuador, cited as Canthidium (Neocanthidium) Martínez, Halffter & Pereira, 1986).

###### Canthidium (Neocanthidium) centrale

Taxon classificationAnimaliaColeopteraScarabaeidae

Boucomont, 1928

Plate 10A

Canthidium centrale Boucomont, 1928b: 203 (original description. Type locality: Panama, Chiriqui, Guyane française: St-Jean du Maroni, St-Laurent du Maroni, Guyane hollandaise, Surinam).
Canthidium centrale : Blackwelder 1944: 205 (list, distribution); Vulcano and Pereira 1967: 593 (characters in key); Howden and Young 1981: 74 (characters in key), 75 (redescription); Medina et al. 2001: 137 (cited for Colombia); Ratcliffe 2002: 14 (cited for Panama); Solís and Kohlmann 2004: 9 (characters in key), 33 (redescription); Solís and Kohlmann 2012: 5 (cited for Costa Rica); Krajcik, 2012: 62 (complete list of species).
Canthidium (Canthidium) centrale : Martínez and Halffter 1986: 26 (cited for Ecuador, Panama, Guatemala, Mexico); Morón 2003: 55 (redescription, cited for Mexico); Carvajal et al. 2011: 318–319 (cited for Ecuador); Chamorro et al. 2018: 92 (cited for Ecuador).
Canthidium (Neocanthidium) centrale : Cupello 2018: 467 (transferred to the subgenus Canthidium (Neocanthidium) Martínez, Halffter & Pereira, 1964. Distribution, cited for Ecuador); Chamorro et al. 2018: 92 (cited for Ecuador).
Neocanthidium martinezi Edmonds & Halffter, 1978: 319 (original description); Solís and Kohlmann 2004: 33 (synonym of Canthidiumcentrale Boucomont, 1928); Solís and Kohlmann 2012: 5 (cited as synonym of Canthidiumcentrale Boucomont, 1928); Cupello, 2018: 467 (comment).

####### Type specimens.

*Canthidiumcentrale* Boucomont, 1928. The holotype (sex unknown) is deposited at the MNHN (see Solís and Kohlmann 2004: 33). Locality: Panama, Chiriqui. Not examined.

*Neocanthidiummartinezi* Edmonds & Halffter, 1978. Type material not examined.

####### Distribution.

Colombia, Ecuador, Guatemala, Panama, and Mexico.

####### Records examined.

ESMERALDAS: Majua (1 specimen CEMT); Playa de Oro (1 specimen CEMT); Pote (1 specimen CEMT). LOS RÍOS: Río Palenque Station (1 specimen CEMT).

####### Temporal data.

Collected in February and March.

####### Remarks.

Inhabits coastal lowland evergreen forests. Collected with pitfall traps baited with human feces.

###### Canthidium (Neocanthidium) coerulescens

Taxon classificationAnimaliaColeopteraScarabaeidae

Balthasar, 1939

Plates 10B , 55D

Canthidium coerulescens Balthasar, 1939c: 117 (original description. Type locality: Ecuador, Ostcordillieren, Teremotillo, Jivaria, Santa Inéz [= Santa Inés]).
Canthidium coerulescens : Krajcik 2012: 62 (complete list of species); Ratcliffe et al. 2015: 196 (cited for Peru).
Canthidium (Canthidium) coerulescens : Martínez and Halffter 1986: 26 (transferred to the subgenus Canthidium (Canthidium) s. str., cited for Ecuador); Carvajal et al. 2011: 318–319 (cited for Ecuador); Bezdek and Hajek 2012: 300 (catalog of types NMPC).
Canthidium (Neocanthidium) coerulescens : Vulcano and Pereira 1967: 592 (characters in key. Transferred to the genus Neocanthidium Martínez, Halffter & Pereira, 1964); Cupello, 2018: 468 (distribution, cited for Ecuador); Chamorro et al. 2018: 89 (figure 12C), 92 (cited for Ecuador).

####### Type specimens.

*Canthidiumcoerulescens* Balthasar, 1939. The lectotype (♂) (here designated) and one paralectotype are deposited at the NMPC (ex coll. V Balthasar). Locality: Jivaria, examined.

**Lectotype** (**here designated**) (♂): “O. Ecuador / Jivaria / 17 12. 05 F. Ohs.S. [p]”, “Typus [p, red label, black margin]”, “LECTOTYPE ♂ / Canthidium / coerulescens / Balth. / des. F.Z. Vaz-de-Mello, 2014 [hw and p, red label, black margin]”.

**Paralectotype** (♂): “O. Ecuador / Jivaria / 17 12. 05 F. Ohs.S. [p]”, “Typus [p, red label, black margin]”, “PARALECTOTYPE / Canthidium ♂ / coerulescens Balth / des. F.Z. Vaz-de-Mello, 2013 [hw and p, yellow label, black margin]”.

####### Distribution.

Ecuador, Peru, and Venezuela.

####### Records examined.

SUCUMBÍOS: Río Azuela Reventador, 1720 m (1 specimen CEMT); La Sofía, 1800 m (2 specimens MUTPL). TUNGURAHUA: Baños El Topo, 1590 m (6 specimens CEMT; 8 specimens MUTPL). ZAMORA CHINCHIPE: Chito Río San Francisco, 1800 m (2 specimens MUTPL); RVS El Zarza conseción El Zarza, campamento las Peñas, Cordillera del Cóndor, 1710 m (2 specimens MUTPL); Tundayme campamento Mirador Jardín Botánico, 925 m (1 specimen CEMT); Tundayme campamento Mirador Escombrera Norte, 1225 m (2 specimens).

####### Literature records.

UNDETERMINED PROVINCE: Teremotillo (Balthasar 1939c: 118); Jivaria (Balthasar 1939c: 118; Bezdek and Hajek 2012: 300); Santa Jnéz [= Santa Inés] (Balthasar 1939c: 118; Bezdek and Hajek 2012: 300).

####### Temporal data.

Collected in January, February, May, August, September, and December.

####### Remarks.

Inhabits lower montane forests and in the montane cloud forests of the Andean regions from 1710–1800 m a.s.l. It was also registered in the evergreen foothill forests and evergreen lower montane forests in the Amazon region from 925–1700 m a.s.l. Collected with pitfall traps baited with human feces.

The lectotype is here designated and illustrated (♂ deposited at the NMPC, Plate 56B) and belongs to the locality Ecuador, Jivaria [= eastern slopes of the Andes mountains].

###### Canthidium (Neocanthidium) escalerai

Taxon classificationAnimaliaColeopteraScarabaeidae

Balthasar, 1939

Plate 10C

Canthidium escalerai Balthasar, 1939c: 121 (original description. Type locality: Ecuador).
Canthidium escalerai : Vulcano and Pereira 1967: 592 (characters in key); Medina et al. 2001: 137 (cited for Colombia); Krajcik 2012: 62 (complete list of species); Ratcliffe et al. 2015: 196 (cited for Peru).
Canthidium (Canthidium) escalerai : Martínez and Halffter 1986: 27 (cited for Ecuador); Carvajal et al. 2011: 318–319 (cited for Ecuador); Bezdek and Hajek 2012: 301 (catalog of types NMPC).
Canthidium (Neocanthidium) escalerai : Cupello 2018: 469 (transferred to the subgenus Canthidium (Neocanthidium) Martínez, Halffter & Pereira, 1964. Distribution, cited for Ecuador); Chamorro et al. 2018: 92 (cited for Ecuador).

####### Type specimens.

*Canthidiumescalerai* Balthasar, 1939. The holotype (♀) is deposited at the NMPC (ex coll. V Balthasar). Locality: Ecuador, Bucay, examined.

**Holotype** (♀): “Typus [p, red label, black margin]”, “C. escalerai / n. sp. m. / Dr. V. Balthasar det [p and hw]”, “escalerai / m. [hw, green label, black margin]”, “Bucay 300 m / F. Ohs. 23. 6. 05 [p]”, “W. Ecuador / Pucay / F. Ohaus S. [p]”, “HOLOTYPE [hw, red label]”.

####### Distribution.

Ecuador and Peru.

####### Records examined.

GUAYAS: Pucay [= Bucay] (1 specimen NMPC).

####### Temporal data.

Collected in June.

####### Remarks.

Inhabits coastal lowland evergreen forests at 300 m a.s.l. Collection method unknown.

###### Canthidium (Neocanthidium) haroldi

Taxon classificationAnimaliaColeopteraScarabaeidae

Preudhomme de Borre, 1886

Canthidium haroldi Preudhomme de Borre, 1886: 111 (original description. Type locality: Nicaragua).
Canthidium haroldi : Gillet 1911a: 55 (list, distribution); Blackwelder 1944: 205 (list, distribution); Howden and Young 1981: 73 (characters in key), 77 (redescription); Medina et al. 2001: 137 (cited for Colombia); Ratcliffe 2002: 14 (cited for Panama); Solís and Kohlmann 2004: 9 (characters in key), 41 (redescription); Solís and Kohlmann 2012: 5 (cited for Costa Rica); Krajcik 2012: 62 (complete list of species).
Canthidium (Canthidium) haroldi : Martínez and Halffter 1986: 28 (transferred to the subgenus Canthidium (Canthidium) s. str., cited for Ecuador, Panama, Costa Rica, Nicaragua and Guatemala); Carvajal et al. 2011: 318–319 (cited for Ecuador).
Canthidium (Neocanthidium) haroldi : Vulcano and Pereira 1967: 590 (characters in key. Transferred to the genus Neocanthidium Martínez, Halffter & Pereira, 1964); Cupello, 2018: 470 (distribution, cited for Ecuador).

####### Type specimens.

*Canthidiumharoldi* Preudhomme de Borre, 1886. The holotype (♂) is deposited at the IRSN (ex coll. E. Candeze). Locality: Nicaragua, examined.

**Holotype** (♂): “Canth. Haroldi / De Borre Type [hw]”, “Haroldi / P. [illegible] Borre / Type / Nicarag. J [hw, green margin]”, “Collection / E. CANDEZE [p, black margin]”, “TYPE [p, pink label, black margin]”, “Type [p, black margin]”, “HOLOTYPE ♂ [p and hw, red label, black margin]”, “Canthidium / haroldi / rev. Preudh [p and hw]”.

####### Distribution.

Colombia, Costa Rica, Ecuador, Guatemala, Nicaragua, and Panama.

####### Literature records.

UNDETERMINED PROVINCE: without specific locality (Martínez and Halffter 1986: 28).

####### Temporal data.

It is not known when this species was collected.

####### Remarks.

Habitat and collection methods are unknown.

###### Canthidium (Neocanthidium) inoptatum

Taxon classificationAnimaliaColeopteraScarabaeidae

Balthasar, 1939

Plate 10D

Canthidium inoptatum Balthasar, 1939c: 130 (original description. Type locality: Ecuador).
Canthidium inoptatum : Vulcano and Pereira 1967: 592 (characters in key).
Canthidium (Canthidium) inoptatum : Martínez and Halffter 1986: 28 (cited for Ecuador); Carvajal et al. 2011: 318–319 (cited for Ecuador); Krajcik 2012: 62 (complete list of species); Bezdek and Hajek 2012: 303 (catalog of types NMPC).
Canthidium (Neocanthidium) inoptatum : Cupello 2018: 470 (transferred to the subgenus Canthidium (Neocanthidium) Martínez, Halffter & Pereira, 1964. Distribution, cited for Ecuador); Chamorro et al. 2018: 92 (cited for Ecuador).

####### Type specimens.

*Canthidiuminoptatum* Balthasar, 1939. Two syntypes examined deposited at the MSMF and NMPC. Lectotype to be designated in a future work on this species group.

**Syntype** (♀): “Ecuador / Catamayo / Ohaus S. [p]”, “Senckember / Museum [p]”, “Typus [p, red label, black margin]”, “Canthidium / inoptatum / n. sp. m / Dr. V. Balthasar det. [p and hw]”, “[one face, p] Typus [oposite face, hw] SMF C / 16818”.

**Syntype** (♀): “W.ECUADOR / Huigra / Dr. Davis [p]”, “Moser determ. [p] / Canthidium sp. [hw]”, “Senckenberg / Museum [p]”, “Typus [red label, black margin]”, “inoptatum [hw, green label, black margin]”.

####### Distribution.

Only known from Ecuador.

####### Records examined.

CHIMBORAZO: Huigra (1 specimen MSMF). LOJA: Catamayo (1 specimen MSMF). UNDETERMINED PROVINCE: Jivaria (1 specimen NMPC).

####### Temporal data.

It is not known when this species was collected.

####### Remarks.

Habitat and collection methods are unknown.

###### Canthidium (Neocanthidium) lentum

Taxon classificationAnimaliaColeopteraScarabaeidae

Erichson, 1847

Canthidium lentum Erichson, 1847: 109 (original description. Type locality: Peru).
Canthidium lentum : Harold 1867b: 62 (redescription); Gemminger and Harold 1869: 1005 (complete list of species); Gillet 1911a: 55 (complete list of species); Blackwelder 1944: 205 (list of species of Latin America); Vaz-de-Mello 2000: 191 (cited for Brazil); Krajcik 2012: 62 (complete list of species); Ratcliffe et al. 2015: 196 (cited for Peru).
Canthidium (Canthidium) lentum : Martínez and Halffter 1986: 28 (transferred to the subgenus Canthidium (Canthidium) s. str., cited for French Guiana, Peru and Ecuador); Carvajal et al. 2011: 318–319 (cited for Ecuador).
Canthidium (Neocanthidium) lentum : Vulcano and Pereira 1967: 591 (characters in key. Transferred to the genus Neocanthidium Martínez, Halffter & Pereira, 1964); Cupello, 2018: 471 (distribution, cited for Ecuador). Chamorro et al. 2018: 92 (cited for Ecuador).

####### Type specimens.

*Canthidiumlentum* Erichson, 1847. One syntype examined deposited at the NMHU. Lectotype to be designated in a future work on this species group.

####### Distribution.

French Guiana, Peru, and Ecuador.

####### Literature records.

UNDETERMINED PROVINCE: without specific locality (Martínez and Halffter 1986: 28).

####### Temporal data.

It is not known when this species was collected.

####### Remarks.

Habitat and collection methods unknown.

###### Canthidium (Neocanthidium) luteum

Taxon classificationAnimaliaColeopteraScarabaeidae

Balthasar, 1939

Plate 11A

Canthidium luteum Balthasar, 1939c: 132 (original description. Type locality: Ecuador).
Canthidium luteum : Vulcano and Pereira 1967: 592 (characters in key); Krajcik 2012: 62 (complete list of species).
Canthidium (Canthidium) luteum : Martínez and Halffter 1986: 28 (cited for Ecuador); Carvajal et al. 2011: 318–319 (cited for Ecuador); Bezdek and Hajek 2012: 303 (catalog of types NMPC).
Canthidium (Neocanthidium) luteum : Cupello 2018: 471 (transferred to the subgenus Canthidium (Neocanthidium) Martínez, Halffter & Pereira, 1964. Distribution, cited for Ecuador); Chamorro et al. 2018: 92 (cited for Ecuador).

####### Type specimens.

*Canthidiumluteum* Balthasar, 1939. The holotype (♀) is deposited at the NMPC. Locality: Loja Ostcordill, examined.

**Holotype** (♀): “Loja Ostcordill. / Sabanilla / F. Ohs. 2. 10. 05 [p]”, “Typus [p, red label, black margin]”, “/ Canthidium / luteum n. sp. / Dr. V. Balthasar det. [p and hw]”, “luteum m. [hw, green label, black margin]”.

####### Distribution.

Only known from Ecuador.

####### Records examined.

LOJA: Ostcordill. Sabanilla [= Sabanilla El Tambo ZAMORA CHINCHIPE] (1 specimen NMPC).

####### Temporal data.

Collected in October.

####### Remarks.

Inhabits the evergreen foothill forests of the Amazon region. The collection method is unknown.

#### Genus *Canthon* Hoffmannsegg, 1817

*Canthon* Hoffmannsegg, 1817: 38 (original description. Type species: *Scarabaeuspilularius*, Linnaeus, 1758 subsequent designation by Paulian, 1939).

*Canthon*: Agassiz 1846: 184 (catalog); Lacordaire 1856: 77 (redescription); Leconte 1861: 125 (characters in key); Harold 1868d: 1 (redescription); Gemminger and Harold 1869: 989 (list, distribution); Blanchard 1885: 163 (redescription); Blatchey 1910: 912 (characters in key); Gillet 1911a: 27 (complete list of species); Lucas 1920: 164 (catalog, distribution); Dawson 1922: 61 (characters in key); Paulian 1938: 235 (characters in key); Paulian 1939: 22 (redescription, type species designation); Pessôa and Lane 1941: 414 (diagnosis); Islas 1942: 303 (redescription); Blackwelder 1944: 198 (list of species of Latin America); Lane 1947: 110 (comment); Roze 1955: 41 (list of species for Venezuela); Pereira and Martínez 1956a: 96 (characters in key); Martínez 1959: 27 (list of species for Argentina); Halffter 1961: 231 (characters in key), 258 (redescription); Vulcano and Pereira 1964: 602 (catalog of species); Halffter and Matthews 1966: 261 (catalog, distribution); Vulcano and Pereira 1967: 549 (characters in key); Halffter and Martínez 1968: 265 (diagnosis); Halffter and Martínez 1977: 38 (characters in key), 69 (redescription); Howden and Young 1981: 14 (characters in key), 19 (redescription); Halffter and Edmonds 1982: 139 (catalog, distribution); Medina and Lopera 2000: 311 (characters in key); Vaz-de-Mello 2000: 191 (list of species for Brazil); Medina et al. 2001: 135 (list of species for Colombia); Solís and Kohlmann 2002: 2 (redescription); Arnett et al. 2002: 49 (characters in key); Ratcliffe 2002: 13 (list of species for Panama); Morón 2003: 30 (redescription); Medina et al. 2003: 64 (distribution); Hamel-Leigue et al. 2006: 13 (list of species for Bolivia); Vaz-de-Mello et al. 2011: 26 (characters in key); Carvajal et al. 2011: 113 (diagnosis), 314 (list of species for Ecuador); Krajcik 2012: 63 (complete list of species); Solís and Kohlmann 2012: 2 (list of species for Costa Rica); Boilly and Vaz-de-Mello 2013: 108 (characters in key); Chamorro et al. 2018: 76 (characters in key), 92 (list of species of Ecuador).

*Coprobius* Latreille, 1829: 535 (original description. Type species: *Scarabaeusvolvens* Fabricius, 1792 subsequent designation by Reiche, 1841); Brullé 1837: 294 (diagnosis); Castelnau 1840: 68 (synonym of *Canthon* Hffsg.); Reiché 1841: 213 (characters in key, type species designation); Agassiz 1846: 282 (catalog); Gemminger and Harold 1869: 989 (synonym of *Canthon* Hoffmannsegg); Burmeister 1873a [1874]: 410 (synonym of *Canthon* Hoffsg); Gillet 1911a: 27 (synonym of *Canthon* Hffsg.); Lucas 1920: 201 (synonym of *Canthon* Hffsg); Blackwelder 1944: 198 (synonym of *Canthon* Hffsg); Pereira and Martínez 1956a: 112 (synonym of *Canthon* Hffsg); Martínez 1959: 27 (synonym of *Canthon* Hffsg); Vulcano and Pereira 1964: 602 (synonym of *Canthon* Hffsg); Solís and Kohlmann 2002: 2 (synonym of *Canthon* Hffsg); Ratcliffe 2002: 12 (synonym of *Canthon* Hffsg); Solís and Kohlmann 2012: 2 (synonym of *Canthon* Hffsg).

*Coeloscelis* Reiche, 1841: 213 (original description. Type species: *Coelosceliscoriaceus* Reiche, 1841 nomen dubium, by original designation. See Cupello and Vaz-de-Mello 2018: 17); Agassiz 1846: 268 (catalog); Lacordaire 1856: 76 (redescription); Gemminger and Harold 1869: 989 (cited as synonym of *Canthon* Hffsg); Gillet 1911a: 27 (synonym of *Canthon* Hffsg.); Blackwelder 1944: 198 (synonym of *Canthon* Hffsg.); Pereira and Martínez 1956a: 112 (synonym of *Canthon* Hffsg.); Martínez 1959: 27 (synonym of *Canthon* Hoffmannsegg); Vulcano and Pereira 1964: 602 (synonym of *Canthon* Hoffmannsegg); Solís and Kohlmann 2002: 2 (synonym of *Canthon* Hffsg); Ratcliffe 2002: 12 (synonym of *Canthon* Hffsg, cited as *Coeloschelis*); Solís and Kohlmann 2012: 2 (synonym of *Canthon* Hffsg).

*Paedhyboma* Kolbe, 1893: 191 (original description. Type species: *Canthonaberrans* Harold, 1868 by primary monotypy); Paulian 1938: 235 (characters in key); Paulian 1939: 21 (redescription); Vulcano and Pereira 1964: 636 (catalog of species); Vulcano and Pereira 1967: 549 (characters in key); Halffter and Martínez 1977: 38 (synonym of *Canthon* Hoffmannsegg); Halffter and Edmonds 1982: 139 (cited as synonym of *Canthon* Hffsg.); Solís and Kohlmann 2002: 2 (cited as synonym of *Canthon* Hffsg); Ratcliffe 2002: 12 (synonym of *Canthon* Hffsg., cited as *Paedohyboma*); Solís and Kohlmann 2012: 3 (synonym of *Canthon* Hffsg., cited as *Paedohyboma*).

*Canthomoechus* Pereira & Martínez, 1959: 165 (original description. Type species: *Canthonquadratus* Blanchard, 1846 by original designation); Halffter 1961: 231 (characters in key); Vulcano and Pereira 1964: 590 (catalog of species); Halffter and Matthews 1966: 261 (catalog, distribution); Halffter and Martínez 1977: 38 (synonym of *Canthon* Hoffmannsegg, 1817); Halffter and Edmonds 1982: 139 (cited as synonym of *Canthon* Hoffmannsegg, 1817); Solís and Kohlmann 2002: 2 (cited as synonym of *Canthon* Hoffmannsegg, 1817); Ratcliffe 2002: 13 (synonym of *Canthon* Hoffmannsegg, 1817); Solís and Kohlmann 2012: 3 (cited as synonym of *Canthon* Hoffmannsegg, 1817).

##### Subgenus Canthon (Canthon) Hoffmannsegg, 1817

Canthon (Canthon) s. str. Hoffmannsegg, 1817: 38 (original description. Type species: *Scarabaeuspilularius*, Linnaeus, 1758); *Canthon*Halffter and Martínez 1968: 270 (characters in key); Halffter and Martínez 1977: 42 (characters in key), 86 (diagnosis); Halffter and Edmonds 1982: 139 (cited as subgenus of *Canthon* Hoffmannsegg, 1817); Vaz-de-Mello 2000: 191 (cited as subgenus of *Canthon* Hoffmannsegg, 1817); Morón 2003: 39 (redescription); Medina et al. 2003: 64 (cited as subgenus of *Canthon* Hoffmannsegg, 1817); Vaz-de-Mello et al. 2011: 27 (characters in key); Boilly and Vaz-de-Mello 2013: 108 (characters in key); Chamorro et al. 2018: 77 (characters in key), 92 (list of species of Ecuador).

###### Canthon (Canthon) aberrans

Taxon classificationAnimaliaColeopteraScarabaeidae

(Harold, 1868)

Plate 11B

Deltochilum aberrans Harold, 1868d: 8 (original description. Type locality: Columbia [= Colombia]).
Deltochilum aberrans : Gemminger and Harold 1869: 995 (list, distribution); Harold 1880a: 18 (distribution); Kolbe 1893: 191 (diagnosis); Gillet 1911a: 35 (complete list of species); Campos 1921: 55 (cited for Ecuador); Blackwelder 1944: 202 (list of species of Latin America); Solís and Kohlmann 2002: 5 (cited as Canthonaberrans (Harold, 1868), synonym).
Paedhyboma aberrans : Shipp 1897: 195 (transferred to the genus Paedhyboma Kolbe, 1893); Paulian 1939: 21 (redescription, distribution); Balthasar 1941: 345 (cited for Peru); Martínez 1947: 113 (cited as synonym of Deltochilumaberrans Harold, 1868); Contreras 1951: 221 (cited for Colombia); Balthasar 1951: 330 (cited for Peru); Vulcano and Pereira 1964: 636 (catalog of species); Vulcano and Pereira 1967: 551 (characters in key); Solís and Kohlmann 2002: 5 (synonym of Canthonaberrans Harold).
Canthon aberrans : Pereira and D’Andretta 1955a: 48 (transferred to the genus Canthon Hoffmannsegg, 1817); Ratcliffe et al. 2015: 195 (cited for Peru).
Canthon (Canthon) aberrans : Halffter and Martínez 1977: 87 (transferred to the subgenus Canthon s. str.); Vaz-de-Mello 2000: 191 (cited for Brazil); Medina et al. 2001: 135 (cited for Colombia); Solís and Kohlmann 2002: 4 (characters in key), 5 (redescription); Ratcliffe 2002: 12 (cited for Panama); Medina et al. 2003: 64 (distribution); Hamel-Leigue et al. 2006: 13 (cited for Bolivia); Carvajal et al. 2011: 314–315 (cited for Ecuador); Krajcik 2012: 63 (complete list of species); Solís and Kohlmann 2012: 3 (cited for Costa Rica); Chamorro et al. 2018: 85 (figure 8A), 87 (figure 10H), 92 (cited for Ecuador).
Canthon bifurcatus Robinson, 1948a: 37 (original description); Martínez 1951a: 23 (distribution, synonym of Canthonjuanae Martínez, 1949); Pereira 1953: 394 (synonym of Paedhybomaaberrans (Harold, 1968)); Pereira and D’Andretta 1955a: 48 (cited as Canthonaberrans (Harold, 1968), synonym); Solís and Kohlmann 2002: 5 (cited as synonym of Canthonaberrans Harold); Solís and Kohlmann 2012: 3 (cited as synonym of Canthonaberrans Harold).
Canthon juanae Martínez, 1949b: 176 (original description); Martínez 1951a: 23 (synonym of Canthonbifurcatus Martínez, 1951); Pereira 1953: 394 (cited as synonym of Canthonplicatipennis Blanchard, 1843); Pereira and D’Andretta 1955a: 48 (cited as Canthonaberrans (Harold, 1968), synonym); Solís and Kohlmann 2002: 5 (cited as Canthon (Canthon) aberrans (Harold), synonym); Solís and Kohlmann 2012: 3 (cited as Canthonaberrans (Harold, 1868), synonym).

####### Type specimens.

*Deltochilumaberrans* Harold, 1868. One syntype examined deposited at the MNHN (ex coll. E Harold and R Oberthur). Lectotype to be designated in a future work on this species group.

*Canthonbifurcatus* Robinson, 1948. The holotype (♀) is deposited at the USNM. Locality: Merida Venezuela. Examined.

**Holotype** (♀): “Merida / Venezuela [p]”, “♀ [p]”, “TypeNo / 65620 / U S N M [p and hw, red label]”, “M.Robinson / Collection / 1959 [p]”, “HOLOTYPE / Canthon / bifurcatus / Mark Robinson [p]”.

*Canthonjuanae* Martínez, 1949. The holotype (♂) is deposited in the AMIC (see Martínez 1949b: 179) [= name-bearing types now in the MACN]. Locality: Bolivia. Dep. La Paz. Nor Yungas Rios Carioco, Choro, Dalem. 700 m. Not examined.

Two paratypes are deposited in CEMT. Examined.

**Paratype** (♀): “BOLIVIA / Dep. La Paz / Pcia. Nor Yungas / Ríos Carioco, Choro / Dalen 700 mts. / Coll. Martínez / Ene-949 [hw]”, “PARATIPO ♀ [hw, green label, black margin]”, “Canthon / juanae ♀ / sp.n. / A. MARTÍNEZ-DET.1949 [p and hw, green label, black margin]”.

**Paratype** (♂): “BOLIVIA / Dep. La Paz / Pcia. Nor Yungas / Ríos Carioco, Choro / Dalen 700 mts. / Coll. Martínez / Ene-949 [hw]”, “PARATIPO ♂ [hw, green label, black margin]”, “Canthon / juanae ♂ / sp.n. / A. MARTÍNEZ-DET.1949 [p and hw, green label, black margin]”.

####### Distribution.

Bolivia, Brazil, Colombia, Costa Rica, Ecuador, Panama, Peru, and Venezuela.

####### Records examined.

COTOPAXI: Bosque Integral Otonga, 1300 m (3 specimens CEMT; 152 specimens MQCAZ); La Mana (5 specimens MQCAZ). IMBABURA: Junín La Mina (7 specimens MQCAZ). EL ORO: Piñas, 1200 m (18 specimens MQCAZ). ESMERALDAS: Los Ajos (8 specimens MQCAZ; 3 specimens MECN); Palma Real (19 specimens MQCAZ; 10 specimens MECN); San Francisco, 20 m (4 specimens MQCAZ); Ricuaute (7 specimens MQCAZ; 1 specimen MECN); Santa Rita (3 specimens MQCAZ). MANABÍ: El Carmen, 600 m (2 specimens MQCAZ). MORONA SANTIAGO: Alshi 9 de Octubre, Rio Upano, 1500 m (2 specimens MUTPL); Gualaquiza (3 specimens MQCAZ); Limón Indanza (2 specimens MQCAZ); Macas, 1000 m (2 specimens MQCAZ). NAPO: Archidona (4 specimens MQCAZ); Cosanga (7 specimens MQCAZ); El Reventador (1 specimen MUTPL); El Reventador, Cascada de San Rafael (8 specimens MQCAZ); Los Guacamayos Piviyacu, 1800 m (4 specimens CEMT); Misahualli, 500 m (3 specimens MQCAZ); Parahuacu (2 specimens MECN); Puente Río Azuela, road Baeza-Lago Agrio (7 specimens MQCAZ); Río Hollín, 1100 m (4 specimens CEMT); San Rafael (6 specimens MQCAZ); Tena Talag, 750 m (2 specimens MQCAZ). ORELLANA: El Coca (1 specimen MQCAZ); Loreto (5 specimens MQCAZ). PICHINCHA: Chiriboga (8 specimens MQCAZ); Chiriboga km 59 (3 specimens MQCAZ); Estación Biológica Maquipucuna, 1250 m (3 specimens MUTPL); Hda Las Palmeras km 57 (4 specimens MQCAZ); Jerusalen (1 specimen MQCAZ); Mindo, 1400–1650 m (4 specimens CEMT); Nanegalito, 1800 m (19 specimens MQCAZ); Pampas Argentinas, 1300 m (8 specimens MQCAZ); Puerto Quito (3 specimens MQCAZ); Puerto Quito km 113 (1 specimen MQCAZ); San Vicente km 4, La Armenia, 1800 m (3 specimens MQCAZ); road Calacalí

Nanegalito, 2000 m (5 specimens MQCAZ); road Chiriboga-Santo Domingo (1 specimen MQCAZ); Tandayapa (3 specimens MQCAZ); Yaruquí, 2700 m (2 specimens MQCAZ); road to Nanegalito km 37 El Vergel, 1600 m (1 specimen CEMT). SANTO DOMINGO DE LOS TSÁCHILAS: ECR Guajalito (25 specimens MQCAZ); Río Toachi (3 specimens MQCAZ); Santo Domingo (7 specimens MQCAZ). SUCUMBÍOS: La Bonita, 1800 m (3 specimens MQCAZ); Limoncocha (1 specimen MQCAZ); El Reventador (2 specimens MQCAZ); road La Alegría-La Bonita km 32 (2 specimens MECN). TUNGURAHUA: Baños (1 specimen MQCAZ); Baños El Topo, 1530 m (3 specimens MUTPL); San Francisco (5 specimens MQCAZ); ZAMORA CHINCHIPE: El Pangui (4 specimens MUTPL); RVS El Zarza campamento las Peñas, Cordillera del Cóndor, 1710 m (4 specimens MUTPL); Guaguaymi, 2000 m (1 specimen CEMT); San Andres, 1850 m (3 specimens MQCAZ); Tundayme campamento Mirador La Mina, 1320 m (2 specimens MUTPL).

####### Temporal data.

Collected every month of the year.

####### Remarks.

Inhabits coastal lowland evergreen forests and coastal evergreen foothill forests from 20–1250 m a.s.l. In the Andean region, it was registered in the lower montane forests and the montane cloud forests from 1300–2300 m a.s.l. In the Amazon, it was registered on the foothill evergreen forests from 500–1100 m a.s.l. Collected with pitfall traps baited with human feces and occassionally in mouse carrion.

###### Canthon (Canthon) delicatulus

Taxon classificationAnimaliaColeopteraScarabaeidae

Balthasar, 1939

Plate 11C

Canthon delicatulus Balthasar, 1939d: 234 (original description. Type locality: West-Ecuador, Guayaquil und Pucay [= Bucay]).
Canthon delicatulum : Blackwelder 1944: 199 (list of species of Latin America).
Canthon delicatulus : Vulcano and Pereira 1964: 610 (catalog of species); Carvajal et al. 2011: 314–315 (cited for Ecuador).
Canthon (Canthon) delicatulus : Halffter and Martínez 1977: 89 (transferred to the subgenus Canthon s. str.); Bezdek and Hajek 2011: 362 (catalog of types NMPC); Krajcik 2012: 63 (complete list of species); Chamorro et al. 2018: 92 (cited for Ecuador).

####### Type specimens.

*Canthondelicatulus* Balthasar, 1939. Two syntypes examined deposited at the MSMF and NMPC. Lectotype to be designated in a future work on this species group.

**Syntype** (♂): “W Ecuador / Guayaquil / F. Ohaus S. [p]”, “Guayaquil / F. Ohs. 28. 5. 05 [p]”, “Canthon / delicatulus / n. sp. m. / Dr. V. Balthasar det. [p and hw]”, “[one face] Typus [p, red label, black margin], [opposite face] 6. 423 [hw]”, “[one face] Senckemberg- / Museum / Frankfurt/Main [p], [opposite face] Canthon / delicatulus B. [hw]”.

**Syntype** (♀): “W Ecuador / Pucay / F. Ohaus S. [p]”, “ Bucay 300 m. / F. Ohs. 23. 6. 05 [p]”, “Typus [p, red label, black margin]”, “C. delicatulus / n. sp. m. / Dr. V. Balthasar det. [p and hw]”, “delicatulus m. [hw, green label, black margin]”.

####### Distribution.

Only known from Ecuador.

####### Records examined.

AZUAY: Ponce Enriquez Mina Sorresdor, 40 m (5 specimens MUTPL); Ponce Enriquez Río Tenguel, 195 m (8 specimens MUTPL). EL ORO: Buenaventura Bajo, 500 m (4 specimens MQCAZ); El Pache 60 m, Río El Pache (5 specimens MQCAZ); 3 km E de Abañin, 800 m (1 specimen CEMT; 7 specimens MQCAZ); Piñas, 1200 m (1 specimen CEMT; 6 specimens MQCAZ); Uzhcurrumi, 500 m (1 specimen CEMT; 3 specimens MQCAZ). ESMERALDAS: Playa de Oro, 200 m (7 specimens MQCAZ); Puerto Balao, 200 m (2 specimens MUTPL); San Mateo (1 specimen CEMT; 2 specimens MQCAZ); Vainilla (3 specimens MQCAZ). GUAYAS: Guayaquil (1 specimen MSMF; 4 specimens CEMT; 5 specimens MQCAZ); Bucay, 300 m (1 specimen NMPC; 6 specimens MQCAZ). LOJA: Catamayo Alamala, 1380 m (1 specimen CEMT); Zapotillo Chaquiro, 340 m (1 specimen CEMT). LOS RÍOS: Estación Río Palenque (6 specimens MQCAZ). MANABÍ: Ayampe, 35 m (1 specimen MUTPL); El Aromo, 370 m (5 specimens MUTPL); San Juan de Manta, 20 m (8 specimens MUTPL); Puerto Rico, 25 m (1 specimen MQCAZ). PICHINCHA: Puerto Quito (5 specimens MQCAZ). SANTO DOMINGO DE LOS TSÁCHILAS: Alluriquín, 1200 m (7 specimens MQCAZ); Santo Domingo, Pupusa (3 specimens MQCAZ); Santo Domingo, Puerto Limón (3 specimens MUTPL).

####### Temporal data.

Collected all months of the year except August.

####### Remarks.

Inhabits coastal lowland evergreen forests, lowland semi-deciduous forests, and evergreen foothill forests from 40–1200 m a.s.l. Collected with pitfall traps baited with carrion and human feces.

###### Canthon (Canthon) gemellatus

Taxon classificationAnimaliaColeopteraScarabaeidae

Erichson, 1847

Canthon gemellatus Erichson, 1847: 105 (original description. Type locality: Peru).
Canthon gemellatum : Harold 1868d: 16 (characters in key); 118 (redescription); Gemminger and Harold 1869: 991 (list, distribution); Kirsch 1873: 340 (cited for Peru); Gillet 1911a: 29 (complete list of species); Schmidt 1922: 75 (list, distribution); Balthasar 1939d: 197 (characters in key, cited for Ecuador); Balthasar 1941: 342 (cited for Peru); 1951: 327 (cited for Peru); Vulcano and Pereira 1964: 613 (catalog of species); Halffter and Martínez 1977: 70 (list of species); Carvajal et al. 2011: 314–315 (cited for Ecuador); Krajcik 2012: 63 (complete list of species); Ratcliffe et al. 2015: 195 (cited for Peru).
Canthon (Canthon) gemellatus : Chamorro et al. 2018: 92 (cited for Ecuador).
Canthon gemellatum : Blackwelder 1944: 199 (list of species for Latin America).

####### Type specimens.

*Canthongemellatus* Erichson, 1847. Four syntypes examined deposited at the NMHU. Lectotype to be designated in a future work on this species group.

####### Distribution.

Ecuador and Peru.

####### Literature records.

UNDETERMINED PROVINCE: without specific locality (Balthasar 1939d: 197).

####### Temporal data.

It is not known when this species was collected.

####### Remarks.

Habitat and collection methods unknown.

###### Canthon (Canthon) obscuriellus

Taxon classificationAnimaliaColeopteraScarabaeidae

Schmidt, 1922

Plate 11D

Canthon obscuriellus Schmidt, 1922: 89 (original description. Type locality: Columbiem [= Colombia], Paramba).
Canthon obscuriellus : Balthasar 1939d: 219 (characters in key); Vulcano and Pereira 1964: 622 (catalog of species); Vulcano and Pereira 1967: 553 (characters in key); Medina et al. 2001: 136 (cited for Colombia); Krajcik 2012: 64 (complete list of species).
Canthon obscuriellum : Blackwelder 1944: 200 (list of species for Latin America); Contreras 1951: 221 (cited for Colombia).
Canthon (Canthon) obscuriellus : Halffter and Martínez 1977: 89 (transferred to the subgenus Canthon s. str.); Chamorro et al. 2018: 92 (cited for Ecuador); Vaz-de-Mello and Cupello 2018b: 62 (figures 80–82), 63 (lectotype designated).

####### Type specimens.

*Canthonobscuriellus* Schmidt, 1922. The lectotype (♂) and one paralectotype are deposited at the SMTD (see Vaz-de-Mello and Cupello 2018b: 63, figure 82). Locality: Paramba, examined.

**Lectotype** (♂): “Paramba / 3500’. IV. 97. [p]”, “dry season. / (Rosenberg). [p]”, “Canthon / obscuriell / n. sp. a. Schmidt. [hw]”, “Coll. C Felche / Kauf 20, 1918 [p, green label, black margin]”, “LECTOTYPE ♂ / Canthon / obscuriellus / Smimidt / des. F.Z. Vaz-de-Mello, 2014 [hw and p, red label, black margin]”.

**Paralectotype** (♀): “Paramba / 3500’. III. 97. [p]”, “dry season. / (Rosenberg). [p]”, “Coll. C Felche / Kauf 20, 1918 [p, green label, black margin]”, “PARALECTOTYPE / Canthon ♀ / obscuriellus / Smimidt / des. F.Z.Vaz-de-Mello, 2014 [hw and p, yellow label, black margin]”.

####### Distribution.

Colombia and Ecuador.

####### Records examined.

IMBABURA: La Carolina, 1000 m (2 specimens MQCAZ; 4 specimens CEMT); Paramba, 3500 feet [= Parambas, 1065 m] (2 specimens SMTD).

####### Temporal data.

Collected in November.

####### Remarks.

Inhabits coastal foothill forests at 1000 m a.s.l. Collection method unknown.

##### Subgenus Canthon (Glaphyrocanthon) Martínez, 1948

Canthon (Glaphyrocanthon) Martínez, 1948a: 41 (original description. Type species: *Glaphyrocanthonvariabilis* Martínez, 1948); Martínez 1949a: 160 (characters in key); Roze 1955: 43 (list of species for Venezuela); Pereira and Martínez 1956a: 96 (characters in key), 125 (list of species); Martínez 1959: 59 (list of species of Argentina); Halffter 1961: 231 (characters in key); Vulcano and Pereira 1964: 660 (catalog of species); Halffter and Matthews 1966: 261 (catalog, distribution); Vulcano and Pereira 1967: 560 (characters in key); Halffter and Martínez 1977: 40 (cited as new status, subgenus of *Canthon* Hoffmannsegg, 1817); Halffter and Edmonds 1982: 139 (cited as subgenus of *Canthon* Hoffmannsegg, 1817); Rivera-Cervantes and Halffter 1999: 32 (redescription); Vaz-de-Mello 2000: 191 (cited as subgénus of *Canthon* Hoffmannsegg, 1817); Ratcliffe 2002: 12 (synonym of *Canthon* Hoffmannsegg, 1817); Medina et al. 2003: 64 (cited as subgenus of *Canthon* Hoffmannsegg, 1817); Vaz-de-Mello et al. 2011: 27 (characters in key); Solís and Kohlmann 2012: 3 (cited as subgenus of *Canthon* Hoffmannsegg, 1817); Boilly and Vaz-de-Mello 2013: 108 (characters in key); Chamorro et al. 2018: 77 (characters in key), 92–93 (list of species of Ecuador).

###### Canthon (Glaphyrocanthon) angustatus

Taxon classificationAnimaliaColeopteraScarabaeidae

Harold, 1867

Plates 12A and 56C

Canthon angustatus Harold, 1867d: 79 (original description, Type locality: Costa Rica).
Canthon angustatus : Harold 1868d: 63 (redescription); Gemminger and Harold 1869: 989 (list, distribution); Bates 1887: 28 (distribution); Kolbe 1905: 579 (list, distribution); Gillet 1911a: 28 (complete list of species); Schmidt 1922: 72 (distribution); Balthasar 1939d: 216 (characters in key); Ratcliffe et al. 2015: 195 (cited for Peru).
Canthon angustatum : Blackwelder 1944: 199 (list of species for Latin America); Contreras 1951: 221 (cited for Colombia); Ratcliffe et al. 2015: 195 (cited for Peru).
Geocanthon angustatus : Pereira and Martínez 1956a: 155 (cited as new combination, diagnosis); Vulcano and Pereira 1964: 669 (catalog of species); Vulcano and Pereira 1967: 550 (characters in key).
Canthon (Glaphyrocanthon) angustatus : Halffter and Martínez 1977: 79 (transferred to the subgenus Glaphyrocanthon Martínez, 1948); Howden and Young 1981: 21 (characters in key), 30 (redescription); Medina et al. 2001: 135 (cited for Colombia); Solís and Kohlmann 2002: 3 (characters in key), 8 (redescription); Ratcliffe 2002: 12 (cited for Panama); Medina et al. 2003: 64 (distribution); Carvajal et al. 2011: 314–315 (cited for Ecuador); Krajcik 2012: 63 (complete list of species); Solís and Kohlmann 2012: 3 (cited for Costa Rica); Chamorro et al. 2018: 92 (cited for Ecuador).

####### Type specimens.

*Canthonangustatus* Harold, 1867. The lectotype (♂) (here designated) is deposited at the MNHN. Locality: Costa Rica. One paralectotype is deposited at the NHRS (ex coll. E Harold and ex coll. R Oberthur), examined.

**Lectotype** (**here designated**) (♂): “Costa Rica [hw]”, “Museúm Paris / ex coll. / R. Oberthür / 1952 [p, green label, black margin]”, “angustatus / +. +. Har. [hw]”, “Ex-Musæo / E. Harold [p, black margin]”, “LECTOTYPE ♂ / Canthon / angustatus / Harold / des. F.Z. Vaz-de-Mello, 2014 [hw and p, red label, black margin]”.

**Paralectotype** (♀): “♀ / Type [p and hw, black margin]”, “Zoolog. / Staatssg [p, black margin]”, “TIPO [p, pink label, black margin]”, “Costarica. / C. / angustatus. / Hrld. [hw, green margin]”, “PARALECTOTYPE / Canthon ♀ / angustatus / Harold / des. F.Z. Vaz-de-Mello, 2014 [hw and p, yellow label, black margin]”.

####### Distribution.

Belize, Colombia, Costa Rica, Ecuador, Guatemala, Nicaragua, and Panama.

####### Records examined.

ESMERALDAS: Colón del Ónzole (2 specimens CEMT; 19 specimens MQCAZ); El Progreso (9 specimens MQCAZ); Gualpí (7 specimens CEMT; 15 specimens MQCAZ; 8 specimens MECN); Mayronga (7 specimens MQCAZ); Palma Real (2 specimens CEMT, 15 specimesn MQCAZ); Playa de Oro, Playa Rica (18 specimens MQCAZ; 15 specimens MECN). LOS RÍOS: Estación Biológica Río Palenque, 250 m (32 specimens MQCAZ; 9 specimens MECN). MANABÍ: El Carmen (3 specimens MQCAZ). SANTO DOMINGO DE LOS TSÁCHILAS: 47 km S de Santo Domingo (1 specimen CEMT; 6 specimens MQCAZ).

####### Temporal data.

Collected in January, February, March, April, May, September, October, and November.

####### Remarks.

Inhabits coastal lowland evergreen forests at 250 m a.s.l. Collected with pitfall traps baited with human feces.

A lectotype is here designated and illustrated (♂, deposited at the MNHN, Plate 56C), recorded in Costa Rica (without specific locality).

###### Canthon (Glaphyrocanthon) bimaculatus

Taxon classificationAnimaliaColeopteraScarabaeidae

Schmidt, 1922

Plate 12B

Canthon bimaculatus Schmidt, 1922: 83 (original description, Type locality: Amazonas, Columbien [= Colombia]).
Canthon bimaculatus : Balthasar 1939d: 216 (characters in key); Carvajal et al. 2011: 314–315 (cited for Ecuador); Ratcliffe et al. 2015: 195 (cited for Peru).
Canthon bimaculatum : Blackwelder 1944: 198 (list of species for Latin America); Contreras 1951: 221 (cited for Colombia); Ratcliffe et al. 2015: 195 (cited for Peru).
Geocanthon bimaculatus : Pereira and Martínez 1956a: 144 (cited as new combination, diagnosis); Vulcano and Pereira 1964: 669 (catalog of species); Vulcano and Pereira 1967: 550 (characters in key).
Geocanthon femoralis bimaculatus : Martínez and Halffter 1972: 58 (cited as new status, redescription).
Canthon (Glaphyrocanthon) femoralisbimaculatus : Halffter and Martínez 1977: 80 (transferred to the subgenus Glaphyrocanthon Martínez, 1948); Rivera-Cervantes and Halffter 1999: 46 (comment); Vaz-de-Mello 2000: 191 (list of species for Brazil); Medina et al. 2001: 136 (cited for Colombia); Medina et al. 2003: 64 (distribution); Morón 2003: 32 (comment).
Canthon (Glaphyrocanthon) bimaculatus : Solís and Kohlmann 2002: 52 (comment); Hamel-Leigue et al. 2006: 13 (cited for Bolivia); Krajcik 2012: 63 (complete list of species); Chamorro et al. 2018: 92 (cited for Ecuador); Vaz-de-Mello and Cupello 2018b: 55 (figures 60 and 61), 56 (lectotype designated).

####### Type specimens.

*Canthonbimaculatus* Schmidt, 1922. The lectotype (♂) is deposited at the NHRS (see Vaz-de-Mello and Cupello 2018b: 56, figure 61). Locality: Colombia, Amazonas, examined.

**Lectotype** (♂): “Amazonas [p, green label]”, “Typ. [p]”, “bimaculatus / type m. [hw]”, “Coll. C. Felsche / Kauf 20, 1918 [p, green label]”, “9285 / E92 + [p, blue label]”, “Typus [p, red label, black margin]”, “LECTOTYPE ♂ / Canthon / bimaculatus Sch. / des. F.Z. Vaz-de-Mello, 2013 [hw and p, red label, black margin]”.

####### Distribution.

Bolivia, Brazil, Colombia, Ecuador, and Peru.

####### Records examined.

ORELLANA: Río Yasuní Garzacocha Ishpingo, 200 m (1 specimen MUTPL); Estación Científica Yasuní PUCE, 250 m (77 specimens MQCAZ). SUCUMBÍOS: Trocha Zábalo-Güepí km 10, Reserva de Producción Faunística Cuyabeno (1 specimen MUTPL).

####### Temporal data.

Collected in February, May, July, and December.

####### Remarks.

Inhabits the lowland evergreen forests of the Amazon region from 200–220 m a.s.l. Collected with canopy fogging methods.

###### Canthon (Glaphyrocanthon) brunnipennis

Taxon classificationAnimaliaColeopteraScarabaeidae

Schmidt, 1922

Plate 12C

Canthon brunnipennis Schmidt, 1922: 84 (original description, type locality: Amazonas).
Canthon brunnipennis : Balthasar 1939d: 204 (characters in key).
Canthon brunnipenne : Blackwelder 1944: 198 (list of species of Latin America).
Glaphyrocanthon brunnipennis : Vulcano and Pereira 1964: 661 (catalog of species).
Glaphyrocanthon (Coprocanthon) brunnipennis : Vulcano and Pereira 1967: 560 (characters in key).
Canthon (Glaphyrocanthon) brunnipennis : Halffter and Martínez 1977: 79 (transferred to the subgenus Glaphyrocanthon Martínez, 1948); Vaz-de-Mello 2000: 191 (cited for Brazil, cited as Canthon (Glaphyrocanthon) brunneipenne); Krajcik 2012: 63 (complete list of species); Chamorro et al. 2018: 92 (cited for Ecuador); Vaz-de-Mello and Cupello 2018b: 56 (lectotype designated), 57, figs 64, 65.

####### Type specimens.

*Canthonbrunnipennis* Schmidt, 1922. The lectotype (♂) is deposited at the NHRS (see Vaz-de-Mello and Cupello 2018b: 56, figure 65). Locality: Amazonas, examined.

**Lectotype** (♂): “Amazonas [p, green label]”, “Coll. C. Felsche / Kauf 20, 1918 [p, green label]”, “brunnipennis / Type m. [hw]”, “14 / 56 [p and hw, pink label]”, “Typus [p, red label, black margin]”, “Glaphyrocanthon / brunnipennis / (Schm) / P. Pereira det.60 [p and hw]”, “9294 / E92 + [p, blue label]”, “Typ. [p]”, “brunnipennis a. schms [hw]”, “NHRS-JLKB / 000021104 [p]”, “LECTOTYPE ♂ / Canthon / brunnipennis Schmidt. / des. F.Z. Vaz-de-Mello, 2013 [hw and p, red label, black margin]”.

####### Distribution.

Brazil and Ecuador.

####### Records examined.

SUCUMBÍOS: Sacha Lodge, 250 m (2 specimens CEMT).

####### Temporal data.

Collected in October.

####### Remarks.

Inhabits lowland evergreen forests of the Amazon region at 250 m a.s.l. Collection method unknown.

###### Canthon (Glaphyrocanthon) luteicollis

Taxon classificationAnimaliaColeopteraScarabaeidae

Erichson, 1847

Plate 12D

Canthon luteicollis Erichson, 1847: 105 (original description. Type locality: Peru).
Canthon luteicollis : Harold 1868d: 13 (characters in key), 59 (redescription); Gemminger and Harold 1869: 992 (list, distribution); Gillet 1911a: 31 (complete list of species); Schmidt 1922: 77 (distribution); Boucomont 1928c: 2 (cited for Peru, Guyana and Ecuador); Balthasar 1939d: 217 (characters in key); Balthasar 1941: 343 (cited for Peru); 1951: 328 (cited for Peru); Medina et al. 2001: 136 (cited for Colombia); Hamel-Leigue et al. 2006: 13 (cited for Bolivia); Carvajal et al. 2011: 314–315 (cited for Ecuador); Ratcliffe et al. 2015: 195 (cited for Peru).
Canthon luteicollis var. nitidicollis : Schmidt 1922: 77 (list, distribution); Balthasar 1939d: 217 (characters in key); Balthasar 1941: 343 (cited for Peru); 1951: 328 (cited for Peru).
Canthon luteicolle var. nitidicolle : Blackwelder 1944: 200 (list of species for Latin America); Carvajal et al. 2011: 314–315 (cited for Ecuador).
Geocanthon luteicollis : Pereira and Martínez 1956a: 177 (cited as new combination); Vulcano and Pereira 1964: 671 (catalog of species); Vulcano and Pereira 1967: 551 (characters in key).
Canthon (Glaphyrocanthon) luteicollis : Halffter and Martínez 1977: 80 (transferred to the subgenus Glaphyrocanthon Martínez, 1948); Vaz-de-Mello 2000: 191 (cited for Brazil, written as *C. Luteicolle*); Medina et al. 2003: 64 (distribution); Krajcik 2012: 64 (complete list of species); Chamorro et al. 2018: 92 (cited for Ecuador).
Canthon nitidicolle Lucas, 1857: 98 (original description); Harold 1868d: 13 (characters in key); 58 (redescription); Gemminger and Harold 1869: 992 (list, distribution); Kirsch 1873: 340 (cited for Peru); Gillet 1911a: 31 (complete list of species); Schmidt 1920: 124 (cited).

####### Type specimens.

*Canthonluteicollis* Erichson, 1847. Two syntypes examined deposited at the NMHU and MNHN. Lectotype to be designated in a future work on this species group.

*Canthonnitidicolle* Lucas, 1857. Six syntypes examined deposited at the MNHN. Lectotype to be designated in a future work on this species group.

####### Distribution.

Bolivia, Brazil, Ecuador, Guyana, and Peru.

####### Records examined.

MORONA SANTIAGO: Comunidad Untsuants Cordillera del Kutukú, 900–1100 m (19 specimens MECN; 24 specimens MQCAZ); Cumpi Cordillera del Kutukú, (1 specimen MUTPL). NAPO: Archidona (10 specimens MQCAZ); Archidona-Jumandi (4 specimens MQCAZ); Baeza (1 specimen MQCAZ); Baeza Oritoyacu (1 specimen MQCAZ); Bloque 20 Pungarayacu, cerca al Tena, 505 m (1 specimen MUTPL); Campanacocha, 220 m (10 specimens MQCAZ); Cosanga, 1900 m (1 specimen MQCAZ); Isla de los Monos (3 specimens MQCAZ); Jarawa (2 specimens MQCAZ); Jatun Sacha Estación Científica, 450 m (19 specimens MQCAZ); Misahualli (1 specimen CEMT); Misahualli Jungle Lodge unión río Napo y río Misahualli, 1600–1900 m (7 specimens MQCAZ); Río Hollín, 1200 m (1 specimen MQCAZ); Pichira (3 specimens MQCAZ); San Luis del Río Hollín, 550 m (3 specimens MQCAZ); Talag Marungachi, 750 m (7 specimens MQCAZ). ORELLANA: Bloque 31, Parque Nacional Yasuní, Perez Companc línea 9, 200 m (10 specimens MECN); Dayuma Campo Hormiguero, plataforma Hormiguero, 320 m (2 specimens MUTPL); Dayuma Campo Palanda, LLumpac, 295 m (2 specimens MUTPL); Dayuma Campo Palanda, Yuca 13, 255 m (2 specimens MUTPL); Dayuma plataforma Ungurahua, 300 m (2 specimens MUTPL); El Coca Primavera (2 specimens MQCAZ); El Coca, Palmoriente (4 specimens MQCAZ); El Dorado plataforma Guarango, 300 m (3 specimens MUTPL); Ines Arango Pre-Cooperativa Andina, Campo Cononaco, 300 m (2 specimens MUTPL); Estación Científica Yasuní PUCE, 250 m (12 specimens CEMT; 85 specimens MQCAZ); Estación de Biodiversidad Tiputini, 215 m, Parque Nacional Yasuní (3 specimens MUTPL); Pozo Daimi (1 specimen CEMT); Río Huataracu, 500 m (2 specimens MQCAZ); Río Rumiyacu-Pozo Apaika (3 specimens MQCAZ); Río Tiputini Yasuní Res. (2 specimens CEMT; 9 specimens MQCAZ); San Sebastian de Coca Comuna Guataraco Campo Pata, 345 m (3 specimens MUTPL); Lago San Pedro, plataforma Copal, 310 m (2 specimens MUTPL); Taracoa, 250 m (4 specimens MQCAZ); Yampuna (3 specimens MQCAZ), Yasuní, 250 m (4 specimens CEMT). PASTAZA: Balsaura (3 specimens MQCAZ); Bosque Protector Oglán Alto, 545–810 m (5 specimens MUTPL); Chuyayacu, 810 m (2 specimens MUTPL); Kapawi, 350 m (3 specimens MQCAZ); Loracachi, 220 m (5 specimens CEMT; 9 specimens MQCAZ); Moretecocha (2 specimens CEMT; 8 specimens MQCAZ); plataforma Villano (7 specimens CEMT; 15 specimens MQCAZ); Tipirishca km 51 road Puyo-Macas, 1050 m (5 specimes MECN). SUCUMBÍOS: Aucayacu Río El Eno, 16 km de Lago Agrio, 275 m (75 specimens MGO-UC); Bermejo plataforma ER-A road to Lumbaqui (1 specimen MUTPL); Campo Drago Shushufindi, 295 m (2 specimens MUTPL); Campo Hormiga, 225 m (1 specimen MUTPL); Cuyabeno (8 specimens MQCAZ); Cuyabeno Cabañas la Hormiga, 240 m (5 specimens MQCAZ); Cuyabeno Laguna Imuya, 220 m (2 specimens MQCAZ); Cuyabeno Laguna Grande, 220 m (6 specimens CEMT); Laguna de Cuyabeno (4 specimens MQCAZ); Limoncocha (4 specimens MQCAZ); Nueva Loja plataforma Iguana, 310 m (2 specimens MUTPL); Pacayacu Campo Libertador, 260 m (1 specimen MUTPL); Río Cuyabeno (3 specimens MQCAZ); 6 km de Dureno, Precooperativa Los Vergeles, 310 m (3 specimens MUTPL); Lagartococha (3 specimens MQCAZ); Sacha Lodge, 270 m (3 specimens MQCAZ); Sta Cecilia, 150 m (1 specimen MQCAZ); Tarapoa Campo Marian, 260 m, plataforma Fanny 5 (1 specimen MUTPL); Tarapoa Nuevo Manabí, 270 m (1 specimen MUTPL). TUNGURAHUA: Machay (1 specimen CEMT; 7 specimens MQCAZ). ZAMORA CHINCHIPE: Tundayme, 800 m (3 specimens MUTPL); Tundayme campamento Mirador, San Marcos, 900 m (1 specimen MUTPL); Tundayme campamento Mirador, Enerentsa, 1030 m (2 specimens MUTPL).

####### Temporal data.

Collected every month of the year.

####### Remarks.

Inhabits the lowland evergreen forests and foothill evergreen forests of the Amazon region from 215–1030 m a.s.l. Collected with pitfall traps baited with carrion, human feces, and dead chilopods.

###### Canthon (Glaphyrocanthon) ohausi

Taxon classificationAnimaliaColeopteraScarabaeidae

Balthasar, 1939 stat. n.

Plates 13A , 56D

Canthon angustatus ohausi Balthasar, 1939d: 216 (original description. Type locality: Ecuador).
Canthon angustatus ohausi : Bezdek and Hajek 2011: 361 (catalog of types NMPC); Chamorro et al. 2018: 92 (cited for Ecuador).
Canthon ohausi : Blackwelder 1944: 200 (list of species for Latin America); Carvajal et al. 2011: 314–315 (cited for Ecuador).

####### Type specimens.

*Canthonangustatusohausi* Balthasar, 1939. The lectotype (♂) (here designated) and one paralectotype are deposited at the NMPC. Locality: Mera, examined.

**Lectotype** (**here designated**) (♂): “Mera / Ecuador [p]”, “Typus [p, red label, black margin]”, “C. angustatus / ssp. m. / Typus ! [p and hw] / Dr. V. Balthasar det. [p]”, “LECTOTYPE ♂ / Canthon angus- / tatus ohausi / Balth. / des. F.Z.Vaz-de-Mello, 2013 [hw and p, red label, black margin]”.

**Paralectotype** (♀): “ECUADOR / Sabanilla / F. OhausS. [p]”, “Senckenberg / Museum [p]”, “Typus [p, red label, black margin]”, “C. angustatus / ssp. Ohausi m. / Typus ! [hw] / Dr. V. Balthasar det. [p]”, “ssp. Ohausi / m. [hw, green label, black margin]”, “PARALECTOTYPE / Canthon angusta- / tus ohausi ♀ / Balth. / des. F.Z.Vaz-de-Mello, 2013 [hw and p, yellow label, black margin]”.

####### Distribution.

Brazil, Colombia, Ecuador, Peru, and French Guiana.

####### Records examined.

NAPO: Río Nushiño Gareno-Waponi, 370 m (1 specimen MUTPL). ORELLANA: Campo Palanda-Yuca Sur, Estación Palanda 5, 320 m (1 specimen MUTPL); Estación de Biodiversidad Tiputini, 215 m, Parque Nacional Yasuní (3 specimens MUTPL); Ines Arango road Tiwino-río Shiripuno, 250 m (1 specimen MUTPL); road to Maxus km 45 Río Capirón, 220 m, Parque Nacional Yasuní Tiputini (1 specimen MUTPL); Lago San Pedro, plataforma Copal, 310 m (1 specimen MUTPL). PASTAZA: Bosque Protector Oglán Alto, 945 m (2 specimens MUTPL; 1 specimen MGO-UC); Chuyayacu, Oleoducto km 25, 200 m (1 specimen MGO-UC); Mera (2 specimens NMPC); Pandanuque, 420 m (1 specimen MUTPL). SUCUMBÍOS: Aucayacu Río El Eno, 16 km de Lago Agrio, 275 m (3 specimens CEMT; 36 specimens MGO-UC); Cascales Pozo Aguas Blancas, 385 m (2 specimens MUTPL); Cascales road to Lumbaqui Pozo Mascarey, 395 m (1 specimen MUTPL); Limoncocha, Reserva Biológica (1 specimen MUTPL); Pacayacu Campo Libertador, Tapi, 265 m (1 specimen MUTPL). ZAMORA CHINCHIPE: Sabanilla [= El Tambo, ZAMORA CHINCHIPE] (1 specimen NMPC); Tundayme campamento Mirador, Enerentsa, 1030 m (1 specimen MUTPL).

####### Temporal data.

Collected in January, February, March, May, June, July, August, September, November, and December.

####### Remarks.

Inhabits the lowland evergreen forests and the foothill evergreen forests of the Amazon region from 215–1300 m a.s.l. Collected with pitfall traps baited with carrion and human feces.

Balthasar (1939) described *Canthonangustatusohausi* (type locality: Ecuador) as a variety of *Canthonangustatus* Harold, 1867 (type locality: Costa Rica), which made the former name available as a subspecific category (see ICZN 1999, Articles 45.6.4 and 46.6.4.1). However, upon examining the external and genital morphology of the type specimens of *C.angustatusohausi* (lectotype ♂ here designated, deposited at the NMPC, Plate 56D) and *C.angustatus* (lectotype ♂ here designated, deposited at the MNHN, Plate 56C), specifically, the differences in the aedeagus and the shapes of the pronotal spots, we think that they belong to two distinct species. Therefore, maintaining the subspecific name originally proposed by Balthasar, we elevate it to species level under the following new status: Canthon (Glaphyrocanthon) ohausi Balthasar, 1939 stat. n. The lectotype is here designated and illustrated (♂ Plate 56D) and originates from Mera, Ecuador (associated with Amazon forests).

###### Canthon (Glaphyrocanthon) pallidus

Taxon classificationAnimaliaColeopteraScarabaeidae

Schmidt, 1922

Plate 13B

Canthon pallidus Schmidt, 1922: 89 (original description. Type locality: Columbien [= Colombia]; Yungas de la Paz, Bolivia; Chanchamayo, Peru; Santa Jnéz [= Santa Inés], Ecuador; Chaco, Bolivia).
Canthon pallidus : Balthasar 1939d: 204 (characters in key); Balthasar 1941: 342 (cited for Peru); 1951: 327 (cited for Peru); Medina et al. 2001: 136 (cited for Colombia); Hamel-Leigue et al. 2006: 13 (cited for Bolivia); Krajcik 2012: 64 (complete list of species); Ratcliffe et al. 2015: 195 (cited for Peru).
Canthon pallidum : Blackwelder 1944: 200 (list of species for Latin America); Contreras 1951: 221 (cited for Colombia).
Geocanthon pallidus : Pereira and Martínez 1960: 47 (cited as new combination); Vulcano and Pereira 1964: 671 (catalog of species).
Canthon (Glaphyrocanthon) pallidus : Halffter and Martínez 1977: 80 (transferred to the subgenus Glaphyrocanthon Martínez, 1948); Medina et al. 2003: 64 (distribution); Carvajal et al. 2011: 314–315 (cited for Ecuador); Chamorro et al. 2018: 87 (figure 10G), 92 (cited for Ecuador); Vaz-de-Mello and Cupello 2018b: 62 (figures 83 and 84), 63 (lectotype designated).
Glaphyrocanthon (Coprocanthon) gutierrezi Martínez, 1949a: 161 (original description).
Geocanthon gutierrezi : Pereira and Martínez 1956a: 151 (cited as new combination, redescription); Pereira and Martínez 1960: 47 (cited as Geocanthonpallidus (Schmidt, 1922) synonym); Halffter and Martínez 1977: 80 (synonym of Canthonpallidus Schmidt).

####### Type specimens.

*Canthonpallidus* Schmidt, 1922. The lectotype (♂) is deposited in the NHRS (see Vaz-de-Mello and Cupello 2018b: 63, figure 84). Locality: Columbia [= Colombia], examined.

**Lectotype** (♂): “Columbia [p]”, “pallidus / a.Schmidth [hw]”, “9971 / E92 + [p, blue label]”, “PARALECTOTYPE / Canthon ♂ / pallidus / A. Schmidt / des. F.Z.Vaz-de-Mello, 2014 [hw and p, yellow label, black margin]”, “LECTOTYPE ♂ / Canthon pallidus / A. Schmidt / des. F.Z.Vaz-de- Mello, 2014 [hw and p, red label, black margin]”.

*Geocanthongutierrezi* Martínez, 1949. The holotype is deposited at the MACN. Locality: Bolivia Dep. La Paz Pcia. Nor Yungas, Sacramento 2500 m. Examined.

**Holotype** (♂): “MACN-En / 1099 [p, black margin]”, “geocanthon / pallidus (Schm.) / A. MARTÍNEZ-DET 1957 [p and hw, black margin]”, “Ene-949 / BOLIVIA / Dep. La Paz / Pcia. Nor Yungas / Sacramento 2500 mts / Coll. Martínez [hw]”, “HOLOTIPO ♂ [hw, red label]”, “glaphyrocanhton / (coprocanthon) ♂ / gutierrezi sp. n / A. MARTÍNEZ-DET 1950 [p and hw, red label, black margin]”.

####### Distribution.

Bolivia, Colombia, Ecuador, and Peru.

####### Records examined.

NAPO: Cosanga Estación Científica Yanayacu, 2130 m (1 specimen MQCAZ); Cuyuja, 2200 m (1 specimen MQCAZ); Hacienda San Isidro, Quijos Valley, 2000 m (1 specimen MQCAZ); Las Palmas, 2050 m (1 specimen MQCAZ). SUCUMBÍOS: Río Azuela, El Reventador, 1720 m (1 specimen CEMT; 2 specimens MUTPL). TUNGURAHUA: Baños, El Topo, 1590 m (1 specimen CEMT). ZAMORA CHINCHIPE: Tundayme campamento Mirador, La Escombrera, 1225 m (1 specimen MUTPL).

####### Literature records.

UNDETERMINED PROVINCE: Santa Jnéz [= Santa Inés] (Schmidt 1922: 90).

####### Temporal data.

Collected in January, August, October, November, and December.

####### Remarks.

Inhabits the evergreen foothill forests and lower montane evergreen forests of the Amazon region from 1225–1720 m a.s.l. In the Andean region, it was registered in the montane cloud forests from 2000–2200 m a.s.l. Collected manually in horse feces and with canopy fogging methods.

###### Canthon (Glaphyrocanthon) plagiatus

Taxon classificationAnimaliaColeopteraScarabaeidae

Harold, 1880

Canthon plagiatus Harold, 1880a: 15 (original description. Type locality: La Meza).
Canthon plagiatus : Gillet 1911a: 32 (complete list of species); Schmidt 1922: 78 (distribution); Balthasar 1939d: 201 (characters in key); Vulcano and Pereira 1964: 625 (catalog of species); Vulcano and Pereira 1967: 552 (characters in key); Carvajal et al. 2011: 314–315 (cited for Ecuador).
Canthon plagiatum : Blackwelder 1944: 200 (list of species of Latin America); Contreras 1951: 221 (cited for Colombia).
Canthon (Glaphyrocanthon) plagiatus : Halffter and Martínez 1977: 80 (transferred to the subgenus Glaphyrocanthon Martínez, 1948); Medina et al. 2001: 136 (cited for Colombia); Hamel-Leigue et al. 2006: 13 (cited for Bolivia); Krajcik 2012: 64 (complete list of species); Chamorro et al. 2018: 93 (cited for Ecuador).

####### Type specimens.

*Canthonplagiatus* Harold, 1880. One syntype examined deposited at the MNHN (ex coll. E. Steinheil). Lectotype to be designated in a future work on this species group.

####### Distribution.

Bolivia, Colombia, and Ecuador.

####### Records examined.

UNDETERMINED PROVINCE: Santa Inés (3 specimens CEMT).

####### Temporal data.

It is not known when this species was collected.

####### Remarks.

Habitat and collection methods unknown.

###### Canthon (Glaphyrocanthon) politus

Taxon classificationAnimaliaColeopteraScarabaeidae

Harold, 1868

Plate 13C

Canthon politus Harold, 1868d: 60 (original description. Type locality: Columbien [= Colombia], Bogotá und das südliche Mexiko [= Mexico]).
Canthon politus : Gemminger and Harold 1869: 992 (list, distribution); Harold 1880a: 16 (cited for Nueva Granada [= Colombia]); Bates 1887: 31 (distribution); Gillet 1911a: 32 (complete list of species); Schmidt 1922: 79 (list, distribution); Balthasar 1939d: 205 (characters in key); Islas 1942: 304 (characters in key), 307 (description).
Canthon politum : Blackwelder 1944: 201 (list of species for Latin America); Roze 1955: 42 (cited for Venezuela).
Canthon politum var. granadense : Blackwelder 1944: 201 (cited as new combination, list of species for Latin America); Contreras 1951: 221 (cited for Colombia).
Geocanthon politus : Pereira and Martínez 1956a: 137 (cited as new combination, redescription); Vulcano and Pereira 1964: 672 (catalog of species); Vulcano and Pereira 1967: 550 (characters in key).
Canthon (Glaphyrocanthon) politus : Halffter and Martínez 1977: 80 (transferred to the subgenus Glaphyrocanthon Martínez, 1948); Medina et al. 2001: 136 (cited for Colombia); Medina et al. 2003: 64 (distribution); Carvajal et al. 2011: 314–315 (cited for Ecuador); Krajcik 2012: 64 (complete list of species); Chamorro et al. 2018: 93 (cited for Ecuador).
Canthon granadensis Lansberge, 1874a: 5 (original description); Gillet 1911a: 29 (complete list of species); Schmidt 1922: 79 (cited); Balthasar 1939d: 223 (characters in key).
Canthon granadense : Blackwelder 1944: 199 (list of species for Latin America).

####### Type specimens.

*Canthonpolitus* Harold, 1868. One syntype examined deposited at the MNHN (ex coll. E Harold and ex coll. R Oberthur). Lectotype to be designated in a future work on this species group.

*Canthongranadensis* Lansberge, 1874. The holotype (♂) is deposited at the MNHN (ex coll. V Lansberge and ex coll. R Oberthur). Locality: without specific locality. Examined.

**Holotype** (♂): “granadensis / Lansb type [hw]”, “Muséum Paris / ex Coll. / R. Oberthür / 1952 [p]”, “Ex-Musæo / VAN LANSBERGE [p, black margin]”, “Canthon (Glaphy- / rocanthon) / politus Harold. G.H. y A.M. det. 76 [hw, black margin]”, “♂ [hw] HOLOTYPE [p, red label, black margin]”.

####### Distribution.

Colombia, Ecuador, and Venezuela.

####### Records examined.

NAPO: B. P. La Cascada, Parque Nacional Sumaco, 1300 m (1 specimen CEMT). ZAMORA CHINCHIPE: Chito Río San Francisco, 1800 m (1 specimen CEMT); El Tambo Reserva El Colibri 2080 m (17 specimens CEMT).

####### Temporal data.

Collected in March and May.

####### Remarks.

Inhabits the evergreen foothill forests and lower evergreen montane forests throughout the Amazonian range from 1300–1800 m a.s.l. In the Andean region, it was recorded in the montane cloud forests from 1800–2080 m a.s.l. This species was collected with pitfall traps baited with human feces. According to Rivera-Cervantes and Halffter (1999), the previous records from Mexico are erroneous.

###### Canthon (Glaphyrocanthon) quadriguttatus

Taxon classificationAnimaliaColeopteraScarabaeidae

(Olivier, 1789)

Plate 13D

Scarabeus quadriguttatus Olivier, 1789: 173 (original description. Type locality: Cayenne, Surinam).
Copris quadriguttatus : Olivier 1790: 178 (new combination, redescription).
Ateuchus bidens Fabricius, 1801: 62 (transferred to the genus Ateuchus Fabricius, 1801); Harold 1868d: 123 (synonym of C.quadriguttatus Oliv.).
Choeridium elegans Castelnau, 1840: 83 (transferred to the genus Choeridium Serville, 1825; redescription); Harold 1868d: 123 (synonym of C.quadriguttatus Oliv.).
Canthon quadriguttatus : Harold 1868d: 123 (transferred to the genus CanthonHoffmannsegg 1817, redescription); Gemminger and Harold 1869: 993 (list, distribution); Gillet 1911a: 32 (complete list of species); Schmidt 1920: 125 (comment, list of species); Medina et al. 2001: 136 (cited for Colombia); Ratcliffe et al. 2015: 195 (cited for Peru).
Glaphyrocanthon quadriguttatus : Pereira and Martínez 1956a: 132 (new combination, catalog of species); Martínez et al. 1964: 12 (characters in key); Vulcano and Pereira 1964: 664 (catalog of species); Vulcano and Pereira 1967: 560 (characters in key).
Canthon (Glaphyrocanthon) quadriguttatus : Halffter and Martínez 1977: 80 (list of species); Rivera-Cervantes and Halffter 1999: 44 (characters in key); Vaz-de-Mello 2000: 191 (cited for Brazil, cited as C.quadriguttatum); Medina et al. 2003: 64 (distribution); Krajcik 2012: 64 (complete list of species); Boilly and Vaz-de-Mello 2013: 112 (figure 36); Chamorro et al. 2018: 93 (cited for Ecuador).
Copris obliquatus Voet, 1806: 47 (original description).
Canthon obliquatus : Schmidt 1920: 125 (comment, list of species); Schmidt 1922: 78 (distribution); Boucomont 1928c: 2 (distribution [quadriguttatus Olivier], cited for Guyana); Balthasar 1939d: 205 (characters in key); Balthasar 1941: 342 (cited for Peru); 1951: 327 (cited for Peru); Halffter and Martínez 1977: 80 (synonym of Canthonquadriguttatus, (Olivier, 1789)); Vaz-de-Mello and Cupello 2018b: 49 (cited as junior objective synonym of Canthon (Glaphyrocanthon) quadriguttatus (Olivier, 1789), comment).

####### Type specimens.

*Scarabeusquadriguttatus* Olivier, 1789. Not found.

*Ateuchusbidens* Fabricius, 1801. One syntype examined deposited at the ZMUC (ex coll. E Harold and R Oberthur). Lectotype to be designated in a future work on this species group.

*Choeridiumelegans* Castelnau, 1840. Type material not examined.

*Canthonobliquatus* Schmidt, 1920. Two syntypes examined deposited at the NHRS. Lectotype to be designated in a future work on this species group.

####### Distribution.

Brazil, Colombia, Ecuador, Peru, Guyana, and Surinam.

####### Records examined.

ORELLANA: El Dorado plataforma Guarango, 300 m (1 specimen MUTPL); El Dorado, plataforma Pitalala 1, 325 m (1 specimen MUTPL); road Auca Tiguino-Ñemenguno, Parque Nacional Yasuní, 350 m (1 specimen MUTPL). PASTAZA: Bosque Protector Oglán Alto, 545 m (2 specimens MUTPL); Pandanuque, 420 m (1 specimen CEMT). SUCUMBÍOS: Aucayacu Río El Eno, 16 km de Lago Agrio, 295 m (1 specimen MUTPL); El Dorado de Cascales, Pozo Cristal 1, 425 m (1 specimen MUTPL); Tarapoa Campo Marian, plataforma Fanny 5 (1 specimen MUTPL); Tarapoa Campo Marian, plataforma Fanny 18B60, 245 m (1 specimen MUTPL). TUNGURAHUA: Baños (1 specimen MUTPL). ZAMORA CHINCHIPE: Tundayme campamento Mirador, Las Maravillas, 1060 m (3 specimens MUTPL); Tundayme campamento Mirador, San Marcos, 900 m (1 specimen MUTPL).

####### Temporal data.

Collected in March, May, July, June, August, October, November, and December.

####### Remarks.

Inhabits the lowland evergreen forests and the foothill evergreen forests of the Amazon region from 245–1060 m a.s.l. Collected in aerial fruit traps, with canopy fogging methods and pitfall traps baited with carrion and human feces.

###### Canthon (Glaphyrocanthon) semiopacus

Taxon classificationAnimaliaColeopteraScarabaeidae

Harold, 1868

Plate 14A

Canthon semiopacus Harold, 1868d: 57 (original description, Type locality: Brazilien [= Brazil], Cayenne).
Canthon semiopacus : Gemminger and Harold 1869: 993 (list, distribution); Harold 1875a: 59 (comment); Gillet 1911a: 33 (complete list of species); Schmidt 1922: 80 (list, distribution); Balthasar 1939d: 208 (characters in key); Medina et al. 2001: 136 (cited for Colombia); Hamel-Leigue et al. 2006: 13 (cited for Bolivia); Krajcik 2012: 64 (complete list of species); Ratcliffe et al. 2015: 195 (cited for Peru).
Canthon semiopacum : Blackwelder 1944: 201 (erroneously cited, list of species from Latin America).
Geocanthon semiopacus : Pereira and Martínez 1956a: 170 (new combination, redescription); Vulcano and Pereira 1964: 673 (catalog of species); Vulcano and Pereira 1967: 550 (characters in key).
Canthon (Glaphyrocanthon) semiopacus : Halffter and Martínez 1977: 80 (list of species); Vaz-de-Mello 2000: 191 (cited for Brazil); Medina et al. 2003: 64 (distribution); Chamorro et al. 2018: 86 (figure 9E), 93 (cited for Ecuador).

####### Type specimens.

*Canthonsemiopacus* Harold, 1868. Two syntypes examined deposited at the MNHN and ZSM (ex coll. E Harold and ex coll. R Oberthür). Lectotype to be designated in a future work on this species group.

####### Distribution.

Bolivia, Brazil, Colombia, Ecuador, and Guyana.

####### Records examined.

NAPO: Talag Marungachi, 750 m (3 specimens MQCAZ). SUCUMBÍOS: Cuyabeno, Jungla lodge, 230 m (18 specimens MQCAZ); Cuyabeno Río Cuyabeno, Campo Hormiga, 225 m (5 specimens MUTPL); Pacayacu Campo Libertador Tapi, 260 m (1 specimen MUTPL); Cuyabeno Laguna Grande, 270 m (20 specimens MQCAZ); Tarapoa Campo Marian, plataform Fanny 5, 260 m (2 specimens MUTPL); Tarapoa, Nuevo Manabí, 270 m (1 specimen MUTPL).

####### Temporal data.

Collected in January, February, April, June, July, August, September, November, and December.

####### Remarks.

Inhabits the lowland evergreen forests and the foothill evergreen forests of the Amazon region from 225–750 m a.s.l. Collected with pitfall traps baited with human feces.

###### Canthon (Glaphyrocanthon) subhyalinus

Taxon classificationAnimaliaColeopteraScarabaeidae

Harold, 1867

Canthon subhyalinus Harold, 1867d: 79 (original description. Type locality: Nova Granada).
Canthon subhyalinus : Harold 1868d: 16 (characters in key), 124 (redescription); Gemminger and Harold 1869: 994 (list, distribution); Harold 1880a: 17 (list of species); Gillet 1911a: 34 (complete list of species); Schmidt 1922: 81 (cited for Ecuador, list of species); Balthasar 1939d: 205 (characters in key); Howden and Young 1981: 22 (characters in key), 31 (redescription).
Canthon subhyalinum : Blackwelder 1944: 202 (list of species for Latin America); Contreras 1951: 221 (cited for Colombia); Roze 1955: 43 (cited for Venezuela); Vulcano and Pereira 1964: 665 (catalog of species); Vaz-de-Mello 2000: 191 (cited for Brazil); Medina et al. 2001: 136 (cited for Colombia); Ratcliffe 2002: 13 (cited for Panama); Hamel-Leigue et al. 2006: 13 (cited for Bolivia); Krajcik 2012: 63 (complete list of species); Ratcliffe et al. 2015: 195 (cited for Peru).
Glaphyrocanthon (Glaphyrocanthon) subhyalinus : Pereira and Martínez 1956a: 132 (new combination, distribution).
Glaphyrocanthon (Coprocanthon) subhyalinus : Martínez et al. 1964: 12 (characters in key); Vulcano and Pereira 1967: 560 (characters in key).
Canthon (Glaphyrocanthon) subhyalinus : Halffter and Martínez 1977: 80 (list of species); Medina et al. 2003: 64 (distribution); Chamorro et al. 2018: 93 (cited for Ecuador).
Canthon (Glaphyrocanthon) subhyalinussubhyalinus : Rivera-Cervantes and Halffter 1999: 63 (diagnosis); Solís and Kohlmann 2002: 5 (characters in key), 47 (redescription); Morón 2003: 33 (cited for Mexico); Carvajal et al. 2011: 314–315 (cited for Ecuador); Solís and Kohlmann 2012: 3 (cited for Costa Rica).

####### Types specimens.

*Canthonsubhyalinus* Harold, 1867. One syntype examined deposited at the MNHN Lectotype to be designated in a future work on this species group.

####### Distribution.

Brazil, Bolivia, Colombia, Costa Rica, Guyana, Ecuador, Mexico, Panama, Peru, and Venezuela.

####### Literature records.

Without specific locality (Schmidt 1922: 81).

####### Temporal data.

It is not known when this species was collected.

####### Remarks.

Habitat and collection methods unknown.

###### Canthon (Glaphyrocanthon) subhyalinoides

Taxon classificationAnimaliaColeopteraScarabaeidae

Balthasar, 1939

Plate 14B

Canthon subhyalinoides Balthasar, 1939d: 231 (original description, Type locality: Ecuador, Quevedo).
Canthon subhyalinoides : Blackwelder 1944: 202 (list of species from Latin America).
Glaphyrocanthon subhyalinoides : Vulcano and Pereira 1964: 665 (new combination, distribution).
Canthon (Glaphyrocanthon) subhyalinoides : Halffter and Martínez 1977: 80 (list of species); Chamorro et al. 2018: 93 (cited for Ecuador).
Canthon (Glaphyrocanthon) subhyalinussubhyalinoides : Rivera-Cervantes and Halffter 1999: 67 (cited as new status, diagnosis); Carvajal et al. 2011: 314–315 (cited for Ecuador); Bezdek and Hajek 2011: 365 (catalog of the types of the NMPC).

####### Type specimens.

*Canthonsubhyalinoides* Balthasar, 1939. One syntype examined deposited at the NMPC. Lectotype to be designated in a future work on this species group.

####### Distribution.

Only known from Ecuador.

####### Records examined.

EL ORO: Salvias Río San José, 1200 m (2 specimens MUTPL). ESMERALDAS: Puerto Balao, 200 m (1 specimen MUTPL). GUAYAS: Río Congo, 35 m (4 specimens MUTPL). LOS RÍOS: Quevedo (1 specimen NMPC). MANABÍ: Ayampe, 35 m (1 specimen MUTPL); El Aromo, Río de los Napos, 280 m (2 specimens MGO-UC; 1 specimen CEMT); Embalse Daule Peripa, Bosque Protector Carrizal-Chone, 110 m (2 specimens MUTPL); RVS Pacoche, 340 m (7 specimens MGO-UC; 1 specimen CEMT); Puerto López Cerro La Gotera, Parque Nacional Machalilla, 350 m (1 specimen MUTPL); Puerto López Guale, 310 m (1 specimen MUTPL); Puerto López Las Tunas, 200 m (1 specimen MUTPL). SANTA ELENA: Olón, 50 m (2 specimens CEMT).

####### Literature records.

LOS RÍOS: Quevedo, Pichilingue 450 m (Rivera-Cervantes and Halffter 1999: 67).

####### Temporal data.

Collected in March, April, July, August, September, October, November, and December.

####### Remarks.

Inhabits coastal lowland evergreen forests, coastal lowland semi-deciduous forests, and coastal evergreen foothill forests from 35–1200 m a.s.l. Collected with pitfall traps baited with human feces.

##### Subgenus Canthon (Goniocanthon) Pereira & Martínez, 1956

Canthon (Goniocanthon) Pereira & Martínez, 1956a: 109 (original description. Type species *Scarabaeussmaragdulus* Fabricius, 1781); Martínez 1959: 49 (list of species for Argentina); Halffter 1961: 231 (characters in key); Vulcano and Pereira 1964: 592 (catalog of species); Halffter and Matthews 1966: 261 (catalog, distribution); Vulcano and Pereira 1967: 551 (characters in key); Halffter and Martínez 1977: 39 (cited new status, subgenus of *Canthon* Hoffmannsegg, 1817); Halffter and Edmonds 1982: 139 (cited as subgenus of *Canthon* Hoffmannsegg, 1817); Vaz-de-Mello 2000: 191 (cited as subgenus of *Canthon* Hoffmannsegg, 1817); Ratcliffe 2002: 12 (synonym of *Canthon* Hoffmannsegg, 1817); Medina et al. 2003: 64 (cited as subgenus of *Canthon* Hoffmannsegg, 1817); Vaz-de-Mello et al. 2011: 26 (characters in key); Solís and Kohlmann 2012: 3 (cited as synonym of *Canthon* Hoffmannsegg, 1817); Boilly and Vaz-de-Mello 2013: 108 (characters in key); Chamorro et al. 2018: 76 (characters in key), 93 (list of species of Ecuador).

###### Canthon (Goniocanthon) fulgidusmartinezi

Taxon classificationAnimaliaColeopteraScarabaeidae

Nunes, Nunes & Vaz-de-Mello, 2018

Plate 14C

Canthon (Goniocanthon) fulgidusmartinezi Nunes, Nunes & Vaz-de-Mello, 2018: 14 (original description. Type locality: Francisco de Orellana, Rodrigo Borja, IAMOE).
Canthon fulgidus Redtenbacher, 1868: 51 (original description, Type locality: Brazilien [= Brazil]); Gemminger and Harold 1869: 991 (list, distribution); Gillet 1911a: 29 (complete list of species); Schmidt 1922: 75 (distribution); Balthasar 1939d: 203 (characters in key); Balthasar 1941: 342 (cited for Peru); Guêrin 1953: 257 (diagnosis); Carvajal et al. 2011: 314–315 (cited for Ecuador); Ratcliffe et al. 2015: 195 (cited for Peru).
Canthon fulgidum : Blackwelder 1944: 199 (misspelled name, list of species of Latin America).
Goniocanthon fulgidus : Pereira and Martínez 1956a: 111 (new combination); Vulcano and Pereira 1964: 593 (catalog of species); Vulcano and Pereira 1967: 551 (characters in key).
Canthon (Goniocanthon) fulgidus : Halffter and Martínez 1977: 75 (list of species); Vaz-de-Mello 2000: 191 (cited for Brazil); Medina et al. 2001: 136 (cited for Colombia); Medina et al. 2003: 64 (distribution); Krajcik 2012: 63 (complete list of species); Vaz-de-Mello and Cupello 2018: 47–48 (cited for Brazil); Chamorro et al. 2018: 87 (figure 10E), 93 (cited for Ecuador).

####### Type specimens.

Canthon (Goniocanthon) fulgidusmartinezi Nunes, Nunes & Vaz-de-Mello, 2018. The holotype is deposited at the CEMT (see Nunes et al. 2018: 15). Locality: Francisco de Orellana, Rodrigo Borja, IAMOE, Ecuador, not examined.

*Canthonfulgidus* Redtenbacher, 1867. The lectotype (sex unknown) is deposited at the MHMW (see Vaz-de-Mello and Cupello 2018a: 47–48). Locality: Brazil. Examined.

**Lectotype**, (sex unknown): “Nott. [hw]”, “fulgidus / det. [illegible]. 93 [p, black margin]”, “TYPUS [p, red label]”, “fulgidus / Redtb [hw]”, “Fulgidus / Brasil Redt. [hw, black margin]”, “LECTOTYPE / Canthon / fulgidus / Redtenb. / des. F.Z. Vaz-de-Mello, 2013 [hw and p, red label, black margin]”.

####### Distribution.

Bolivia, Brazil, Colombia, Ecuador, and Peru.

####### Records examined.

NAPO: Archidona, 500 m (4 specimens MQCAZ); Sunka (7 specimens MQCAZ). ORELLANA: Apaika Sur (8 specimens MQCAZ); Bloque 31 Pozo Petrolero PSCA 2, Parque Nacional Yasuní (6 specimens MECN); Bloque 31 Perez Companc línea 9, Parque Nacional Yasuní, 200 m (3 specimens MECN); Daimi 1 (28 specimens MQCAZ); Dayuma plataforma Ungurahua, 300 m (1 specimen MUTPL); El Dorado, 350 m, plataforma Pitala 1 (1 specimen MUTPL); Estación Río Huiririma (5 specimens MQCAZ); Loreto (2 specimens MQCAZ); Nashiño, 255 m (7 specimens MQCAZ); Río Rumiyacu Pozo Apaika I, Parque Nacional Yasuní (1 specimen MUTPL); Estación Científica Yasuní PUCE, 250 m (3 specimens CEMT; 168 specimens MQCAZ); Estación de Biodiversidad Tiputini, 215–285 m, Parque Nacional Yasuní (2 specimens MGO-UC; 21 specimens MQCAZ); Pozo Ginta 1, Parque Nacional Yasuní, 230 m (18 specimens MQCAZ); Parque Nacional Yasuní (1 specimen CEMT; 22 specimens MQCAZ); Río Tiputini, Yasuní Res. (1 specimen CEMT; 8 specimens MQCAZ); Rodrigo Borja IAMOE (7 specimens CEMT; 25 specimens MQCAZ); San Sebastian del Coca Comuna Shamanal, Campo Palo Azul, 345 m (1 specimen MUTPL); Zamona-Yuturi (10 specimens MQCAZ). PASTAZA: Campo Tiguino cerca al estero Ñemenguno, 300 m (1 specimen MUTPL); Kurintza, 300 m (3 specimens MECN). SUCUMBÍOS: Aucayacu Río El Eno, 16 km de Lago Agrio 290 m (3 specimens MGO-UC); Cuyabeno, 250–400 m (19 specimens MQCAZ); Cuyabeno Jungla Lodge, 230 m (12 specimens MQCAZ); Río Aguarico, 300 m (5 specimens MQCAZ); Tarapoa Campo Marian, plataforma Fanny 5, 255 m (1 specimen MUTPL); Tarapoa, Nuevo Manabí, 270 m (1 specimen MUTPL).

####### Literature records.

ORELLANA: Francisco de Orellana (Nunes et al. 2018: 15); PUCE Yasuni, 250 m (Nunes et al. 2018: 15); Parque Nacional Yasuni (Nunes et al. 2018: 15); Mid. Rio Tiputini, Yasuni Res. Stn. (Nunes et al. 2018: 15); Yasuni National Park, 215 m (Nunes et al. 2018: 15).

####### Temporal data.

Collected every month of the year.

####### Remarks.

Inhabits the lowland evergreen forests of the Amazon region from 215–345 m a.s.l. Collected with canopy fogging methods and pitfall traps baited with carrion and human feces.

##### *Canthon* incertae sedis (sensu Halffter and Martínez 1977)

*Canthon* incertae sedis Halffter & Martínez, 1977: 70 (comment, list of species); Halffter and Edmonds 1982: 139 (cited as *Canthon* not assigned to subgenera according to Halffter and Martínez 1977); Vaz-de-Mello 2000: 191 (cited as incertae sedis); Medina et al. 2003: 64 (cited as incertae sedis); Vaz-de-Mello et al. 2011: 4 (cited as incertae sedis sensu Halffter & Martínez, 1977); Chamorro et al. 2018: 76–77 (characters in key), 93 (list of species of Ecuador).

###### Canthon balteatus

Taxon classificationAnimaliaColeopteraScarabaeidae

Boheman, 1858

Plate 14D

Canthon balteatus Boheman, 1858: 41 (original description, type locality: Insula Ohau, Honolulu).
Canthon balteatus : Harold 1868d: 16 (characters in key); 125 (redescription); Gemminger and Harold 1869: 990 (species list, distribution); Gillet 1911a: 28 (complete list of species); Schmidt 1922: 73 (list, distribution); Balthasar 1939d: 209 (characters in key); Vulcano and Pereira 1964: 604 (catalog of species, erroneously cited as Canthonbaltheatus); Halffter and Martínez 1977: 70 (list of species); Medina et al. 2003: 65 (distribution); Carvajal et al. 2011: 314–315 (cited for Ecuador); Krajcik 2012: 63 (complete list of species); Ratcliffe et al. 2015: 195 (cited for Peru); Chamorro et al. 2018: 93 (cited for Ecuador).
Canthon balteatum : Blackwelder 1944: 198 (misspelled name, list of species of Latin America).
Canthon balteatus var. lojanus Balthasar, 1939d: 210 (original description); Bezdek and Hajek 2011: 362 (catalog of the types of the NMPC); Chamorro et al. 2018: 93 (cited for Ecuador).
Canthon lojanum : Blackwelder 1944: 200 (list of species from Latin America).

####### Type specimens.

*Canthonbalteatus* Boheman, 1858. Five syntypes examined deposited at the NHRS and IRSN. Lectotype to be designated in a future work on this species group.

Canthonbalteatusvar.lojanus Balthasar, 1939. One syntype (♀) examined is deposited at the NMPC. Locality: Loja. Examined.

**Syntype** (♀): “ECUADOR / Loja / Ohaus S. [p]”, “Typus [p, red label, black margin] “, “var. lojanus m. [hw, green label, black margin]”.

####### Distribution.

Ecuador and Peru.

####### Records examined.

AZUAY: 5 km W and 3 km S de Sta Isabel, 1450 m (7 specimens CEMT). EL ORO: Arenillas, 15 m (100 specimens CEMT; 165 specimens MUTPL); GUAYAS: Guayaquil (5 specimens MQCAZ). MANABÍ: Aguas Blancas, 50 m (3 specimens MUTPL); Crucita (3 specimes MQCAZ); El Aromo, La Fabril, 290 m (2 specimens MUTPL); El Aromo, 370 m (2 specimens MQCAZ); Manta, 10 m (6 specimens MUTPL; 7 specimens MQCAZ); Montecristi, Pichihuama, 190 m (10 specimens MQCAZ); Puerto López (2 specimens MUTPL; 3 specimens MQCAZ); San Clemente (5 specimens MQCAZ). LOJA: Catamayo (3 specimens MQCAZ; 8 specimens MUTPL); Catamayo, Alamala, 1400 m (10 specimens CEMT; 18 specimens MUTPL); Celica (29 specimens MUTPL); Río Catamayo, 1500 m (1 specimen CEMT; 5 specimens MUTPL); Saraguro, Manú, 1300 m (16 specimens MUTPL); Zapotillo, Chaquiro, 310 m (15 specimens MUTPL). LOS RÍOS: CRP [= Centro Río Palenque] (1 specimen CEMT; 4 specimens MQCAZ). SANTA ELENA: Olón, 10 m (4 specimens CEMT; 12 specimens MUTPL).

####### Temporal data.

Collected every month of the year.

####### Remarks.

Inhabits coastal lowland semi-deciduous forests and coastal lowland dry scrub from 10–1400 m a.s.l. Collected with pitfall traps baited with carrion and human feces.

According to Bousquet (2016: 84) and subsequently Cupello (2018: 477) the reports by Boheman (1858) are possibly incorrect with regard to their type localities. Specifically, it is likely that some specimens collected in the Neotropics were mingled with others caught in the Hawaiian archipelago.

###### Canthon fuscipes

Taxon classificationAnimaliaColeopteraScarabaeidae

Erichson, 1847

Canthon fuscipes Erichson, 1847: 105 (original description. Type locality: Peru).
Canthon fuscipes Harold, 1868d: 14 (characters in key); 75 (redescription); Gemminger and Harold 1869: 991 (list, distribution); Gillet 1911a: 29 (complete list of species); Schmidt 1922: 75 (list, distribution, written as cited as Canthonfuscips); Balthasar 1939d: 198 (characters in key); Balthasar 1941: 342 (cited for Peru); Blackwelder 1944: 199 (list of species for Latin America); Balthasar 1951: 327 (cited for Peru); Vulcano and Pereira 1964: 613 (catalog of species); Halffter and Martínez 1977: 70 (list of species); Carvajal et al. 2011: 314–315 (cited for Ecuador); Krajcik 2012: 63 (complete list of species); Ratcliffe et al. 2015: 195 (cited for Peru); Chamorro et al. 2018: 93 (cited for Ecuador).

####### Type specimens.

*Canthonfuscipes* Erichson, 1847. Two syntypes examined deposited at the NMHU. Lectotype to be designated in a future work on this species group.

####### Distribution.

Ecuador and Peru.

####### Literature records.

UNDETERMINED PROVINCE: without specific locality (Schmidt 1922: 75).

####### Temporal data.

It is not known when this species was collected.

####### Remarks.

Habitat and collection methods are unknown.

###### Canthon sericatus

Taxon classificationAnimaliaColeopteraScarabaeidae

Schmidt, 1922

Plate 15A

Canthon sericatus Schmidt, 1922: 92 (original description, type locality: Argentinien [= Argentina], Salinas).
Canthon sericatus : Balthasar 1939d: 186 (characters in key); Martínez 1959: 42 (cited for Argentína); Vulcano and Pereira 1964: 630 (catalog of species); Halffter and Martínez 1977: 71 (list of species); Krajcik 2012: 64 (complete list of species); Ratcliffe et al. 2015: 195 (cited for Peru); Chamorro et al. 2018: 93 (cited for Ecuador); Vaz-de-Mello and Cupello 2018b: 67 (lectotype designated), figs 97 and 98.
Canthon sericatum : Blackwelder 1944: 201 (misspelled name, list of species for Latin America).

####### Type specimens.

*Canthonsericatus* Schmidt, 1922. The lectotype (♂) is deposited at the SMTD (see Vaz-de-Mello and Cupello 2018b: 67, figure 98). Locality: Salinas, Beni, examined.

**Lectotype** (♂): “Salinas / Beni B vii. 95 / M. Stuart [p]”, “Coll. C. Felsche / Kauf 20, 1918 [p, green label, black margin]”, “Typus [red label]”, “canthon / sericatus / n. sp. a. Schmidth [hw]”, “LECTOTYPE ♂ / Canthon / sericatus / Schmidt / des. F.Z.Vaz-de-Mello, 2014 [hw and p, red label, black margin]”.

####### Distribution.

Argentina, Ecuador, and Peru.

####### Records examined.

PASTAZA: Amazanga Norte del Puyo, 1000 m (1 specimen CEMT).

####### Temporal data.

Collected in November.

####### Remarks.

Inhabits the foothill evergreen forests in the Amazon region at 1000 m a.s.l. Collected with light trap.

#### Genus *Canthonella* Chapin, 1930

*Canthonella* Chapin, 1930: 1 (original description. Type species: *Canthonellaparva* Chapin, 1930 by original designation).

*Canthonella*: Blackwelder 1944: 198 (list of species of Latin America); Martínez 1954b: 64 (comment); Pereira and Martínez 1956a: 94 (characters in key), 99 (list of species, distribution); Halffter 1961: 230 (characters in key); Vulcano and Pereira 1964: 581 (catalog of species); Matthews 1965: 433 (characters in key), 447 (redescription); Zayas and Matthews 1966: 3 (characters in key), 16 (redescription); Matthews 1966: 7 (characters in key), 75 (redescription); Halffter and Matthews 1966: 260 (catalog, distribution); Halffter and Martínez 1967: 90 (redescription); Halffter and Martínez 1977: 34 (characters in key), 58 (comment); Halffter and Edmonds 1982: 139 (catalog, distribution); Ratcliffe and Smith 1999; 2 (comment, list of species); Medina and Lopera 2000: 311 (characters in key); Vaz-de-Mello 2000: 192 (list of species for Brazil); Medina et al. 2001: 136 (list of species for Colombia); Medina et al. 2003: 65 (distribution); Vaz-de-Mello et al. 2011: 25 (characters in key); Carvajal et al. 2011: 115 (diagnosis), 314–315 (List of species for Ecuador); Krajcik 2012: 64 (complete list of species); Boilly and Vaz-de-Mello 2013: 107 (characters in key); Chamorro et al. 2018: 75 (characters in key), 84 (figure 7H), 93 (cited for Ecuador).

*Ipselissus* d’Olsouffief, 1935: 35 (nom. prov., comment); Martínez 1954b: 59 (redescription); Pereira and Martínez 1956a: 94 (characters in key), 99 (distribution); Halffter 1961: 230 (characters in key); Vulcano and Pereira 1964: 581 (catalog of species); Halffter and Matthews 1966: 260 (catalog, distribution); Halffter and Martínez 1967: 103 (redescription); Halffter and Martínez 1968: 211 (comment); Halffter and Martínez 1977: 34 (characters in key), 51 (synonym of *Canthonella* Chapin).

*Ipsepilissus* Paulian, 1938: 235 (nom. nud., characters in key); Paulian 1939: 29 (nom. nud., description); Martínez 1947: 113 (nom. nud., cited).

**Remarks.** There are possibly three new Ecuadorian species from Orellana, Pichincha, and Sucumbíos provinces. Their description will be included in a future work on this genus.

#### Genus *Copris* Geoffroy, 1762

*Copris* Geoffroy, 1762: 87 (original description. Type species: *Scarabaeuslunaris* Linnaeus, 1758. Type subsequent designated by Latreille, 1810).

*Copris*: Fourcroy 1785: 13 (redescription, list of species); Olivier 1790: 144 (list of species); Geoffroy 1799: 87 (redescription); Fabricius 1801: 30 (redescription); Agassiz 1846: 282 (catalog); Lacordaire 1856: 96 (redescription); Leconte 1861: 126 (characters in key); Gemminger and Harold 1869: 1013 (list, distribution); Reitter 1893: 160 (characters in key); Peringuey 1900: 110 (characters in key), 342 (redescription); Blatchey 1910: 915 (redescription); Gillet 1911a: 71 (complete list of species); Lucas 1920: 201 (catalog, distribution); Dawson 1922: 61 (characters in key); Paulian 1938: 232 (characters in key); Blackwelder 1944: 208 (list of species of Latin America); Roze 1955: 45 (list of species for Venezuela); Howden and Young 1981: 12 (characters in key); ICZN 1994: 61 (decision on the availability of the name *Copris* under the authorship of Geoffroy, 1762); Arnett et al. 2002: 49 (characters in key); Ratcliffe 2002: 15 (list of species for Panama); Vaz-de-Mello et al. 2011: 28 (characters in key); Krajcik 2012: 78 (complete list of species); Solís and Kohlmann 2012: 6 (list of species for Costa Rica); Chamorro et al. 2018: 77 (characters in key), 90 (figure 13F), 93 (cited for Ecuador).

*Copris* “Müller, 1764”: XI (original description. Type species: *Scarabaeuslunaris* Linnaeus, 1758); Matthews 1961: 35 (redescription); Halffter and Matthews 1966: 258 (catalog, distribution); Howden and Young 1981: 130 (redescription); Halffter and Edmonds 1982: 136 (catalog, distribution); Medina and Lopera 2000: 300 (characters in key); Medina et al. 2001: 133 (list of species for Colombia); Morón 2003: 45 (redescription); Carvajal et al. 2011: 136 (diagnosis, cited as *Copris* “Müller, 1764”), 320 (list of species for Ecuador); Marchisio and Zunino 2012: 23 (comment), 28 (redescription).

*Litocopris* Waterhouse, 1891b: 53 (original description. Type species: *Litocoprispunctiventris* Waterhouse 1891); Peringuey 1900: 342 (cited as synonym); Blackwelder 1944: 208 (synonym of *Copris* Goeffr); Balthasar 1958: 474 (redescription); Halffter and Matthews 1966: 258 (cited as subgenus of *Copris* “Müller 1764”); Halffter and Edmonds 1982: 136 (cited as subgenus of *Copris* “Müller 1764”); Ratcliffe 2002: 15 (cited as synonym of *Copris* Goeffroy, 1762); Solís and Kohlmann 2012: 6 (cited as synonym of *Copris* Goeffroy, 1762).

##### Subgenus Copris (Copris) Geoffroy, 1762

Copris (Copris) s. str. Geoffroy, 1762: 87 (original description. Type species: *Scarabaeuslunaris* Linnaeus, 1758); Balthasar 1958: 473 (redescription); Marchisio and Zunino 2012: 28 (redescription); Chamorro et al. 2018: 77 (characters in key), 93 (list of species of Ecuador).

###### Copris (Copris) davidi

Taxon classificationAnimaliaColeopteraScarabaeidae

Darling & Génier, 2018

Plate 15B

Copris davidi Darling & Génier, 2018: 31 (original description. Type locality: Pajonal, Esmeraldas, Ecuador).

####### Type specimens.

*Coprisdavidi* Darling & Génier, 2018. The holotype (♂) is deposited at the CMNC (see Darling and Génier 2018: 32). Locality: Ecuador, Esmeraldas, Pajonal, not examined.

####### Distribution.

Colombia and Ecuador.

####### Records examined.

IMBABURA: Carolina, 1000 m (1 specimen CEMT); Lita, 500 m (2 specimens CEMT). ESMERALDAS: Colón del Ónzole (2 specimens CEMT); Gualpi (1 specimen CEMT); Gualpi el Pajonal (5 specimens CEMT); Jeyambi PMFC (1 specimen CEMT).

####### Literature records.

IMBABURA: Carolina (Darling and Génier 2018: 32); Lita (Darling and Génier 2018: 32). ESMERALDAS: Chispero (Darling and Génier 2018: 32); Colón del Ónzole (Darling and Génier 2018: 32); Gualpí del Ónzole (Darling and Génier 2018: 32); Esmeraldas (Darling and Génier 2018: 32); Estación Forestal La Chiquita, 11km SE San Lorenzo, 5m (Darling and Génier 2018: 32); La Concordia (Darling and Génier 2018: 32); Majua (Darling and Génier 2018: 32), Pajonal (Darling and Génier 2018: 32); Jeyambi PMFC (Darling and Génier 2018: 32); Punta Venado (Darling and Génier 2018: 32). MANABÍ: 78 km NE Chone, 85 km WSW Santo Domingo, 450 m (Darling and Génier 2018: 32); 90 km WSW Santo Domingo, 73 km NE Chone, 300 m (Darling and Génier 2018: 32).

####### Temporal data.

Collected in April, May, June, July, August, September, October, and November.

####### Remarks.

Inhabits coastal lowland evergreen forests and coastal evergreen foothill forests from 5–1000 m a.s.l. Collected with pitfall traps baited with human feces.

###### Copris (Copris) susanae

Taxon classificationAnimaliaColeopteraScarabaeidae

Darling & Génier, 2018

Plate 15C

Copris susanae Darling & Génier, 2018: 28 (original description. Type locality: 20 km N Chone, 300 m, Manabí, Ecuador).

####### Type specimens.

*Coprissusanae* Darling & Génier, 2018. The holotype (♂) is deposited at the CMNC (see Darling and Génier 2018: 29). Locality: Ecuador, Manabí, 20 km N Chone, 300 m, not examined.

####### Distribution.

Colombia and Ecuador.

####### Records examined.

BOLIVAR: B. P. Filo Palanga (1 specimen CEMT). COTOPAXI: Guasaganda, 500 m (1 specimen CEMT). EL ORO: Piñas, 1200 m (5 specimens CEMT); Uzhcurrumi, 500 m (1 specimen CEMT). LOJA: 5 km N de Zambi, 1300 m (1 specimen CEMT). LOS RIOS: Río Palenque Station (6 specimens CEMT). SANTA ELENA: Olón, 10 m (76 specimens CEMT).

####### Literature records.

BOLIVAR: Balzapamba (Darling and Génier 2018: 30); CANAR [= CAÑAR]: Route La Troncal-Canar [= Cañar], 300 m (Darling and Génier 2018: 30). ESMERALDAS: Esmeraldas (Darling and Génier 2018: 30); San Mateo (Darling and Génier 2018: 30). GUAYAS [= SANTA ELENA]: 27 km S Puerto Lopez, 76 km N Santa Elena, 150 m (Darling and Génier 2018: 30); GUAYAS: Bucay (Darling and Génier 2018: 30). LOS RÍOS: Estación Científica Río Palenque, 47 km S Santo Domingo, 250 m (Darling and Génier 2018: 30); Estación Experimental Tropical Pichilingue, Quevedo (Darling and Génier 2018: 30); Hacienda Ana María, Quevedo (Darling and Génier 2018: 30); MANABÍ: 20 km N Chone, 300 m (Darling and Génier 2018: 30); 78 km NE Chone, 85 km WSW Santo Domingo, 450 m (Darling and Génier 2018: 30); 90 km WSW Santo Domingo, 73 km NE Chone, 300 m (Darling and Génier 2018: 30); MORONA SANTIAGO: Macas (Darling and Génier 2018: 30). SANTA ELENA: Olón, 10 m (Darling and Génier 2018: 30). UNDETERMINED PROVINCE: San Rafael (Darling and Génier 2018: 30); without specific locality (Darling and Génier 2018: 30).

####### Temporal data.

Collected in February, March, May, June, July, August, September, and December.

####### Remarks.

Inhabits coastal lowland evergreen forests and coastal evergreen foothill forests from 10–1200 m a.s.l. In the Amazon region, this species has been collected in the foothill evergreen forests. Collected with pitfall traps baited with human feces. According to Darling and Génier (2018) the Macas record (Morona Santiago province) might be erroneous.

#### Genus *Coprophanaeus* d’Olsoufieff, 1924

*Coprophanaeus* d’Olsoufieff, 1924: 22 (original description. Type species: *Scarabeusjasius* Olivier, 1789, by original designation).

*Coprophanaeus*: Pessôa 1934: 295 (redescription); Pessôa and Lane 1941: 476 (characters in key); Blackwelder 1944: 209 (list of species from Latin America); Martínez 1959: 100 (cited as subgenus Coprophanaeus Olsuefieff); Halffter and Matthews 1966: 258 (cited as subgenus Coprophanaeus Olsuefieff); Vulcano and Pereira 1967: 570 (characters in key, cited as subgenus Coprophanaeus Olsuefieff); Edmonds 1972: 820 (characters in key); 839 (redescription); Howden and Young 1981: 12 (characters in key); Halffter and Edmonds 1982: 136 (catalog, distribution); Medina and Lopera 2000: 303 (characters in key); Vitolo 2000: 593 (characters in key); Vaz-de-Mello 2000: 192 (list of species for Brazil); Medina et al. 2001: 140 (list of species for Colombia); Arnaud 2002a: 13 (characters in key); Arnett et al. 2002: 49 (characters in key); Ratcliffe 2002: 16 (list of species for Panama); Morón 2003: 59 (redescription); Edmonds 1994: 17 (characters in key); Vitolo 2004: 289 (redescription); Hamel-Leigue et al. 2006: 17 (list of species for Bolivia); Hamel-Leigue et al. 2009: 56 (distribution of records for Bolivia); Edmonds and Zídek 2010: 8 (revision); Vaz-de-Mello et al. 2011: 24 (characters in key); Carvajal et al. 2011: 138 (diagnosis), 320 (list of species for Ecuador); Solís and Kohlmann 2012: 7 (list of species for Costa Rica); Krajcik 2012: 204 (complete list of species, cited as subgenus of *Phanaeus* Macleay, 1819); Boilly and Vaz-de-Mello 2013: 107 (characters in key); Chamorro et al. 2018: 75 (characters in key), 93 (list of species of Ecuador).

##### Subgenus Coprophanaeus (Coprophanaeus) d’Olsoufieff, 1924

Coprophanaeus (Coprophanaeus) s. str. d’Olsoufieff, 1924: 22 (original description. Type species: *Scarabeusjasius* Olivier, 1789); Pessôa and Lane 1941: 476 (characters in key); Blackwelder 1944: 209 (list of species from Latin America); Martínez 1959: 100 (list of species from Argentina, cited as subgenus of *Phanaeus* Macleay, 1819); Halffter and Matthews 1966: 258 (catalog, distribution, cited as subgenus of *Phanaeus* Macleay, 1819); Vulcano and Pereira 1967: 570 (characters in key, cited as subgenus of *Phanaeus* Macleay, 1819); Edmonds 1972: 840 (characters in key), 843 (redescription); Halffter and Edmonds 1982: 136 (catalog, distribution); Vaz-de-Mello 2000: 192 (list of species from Brazil); Arnaud 2002a: 24 (catalog of species); Vitolo 2004: 290 (diagnosis); Hamel-Leigue et al. 2009: 56 (distribution of records for Bolivia); Edmonds and Zídek 2010: 9 (characters in key), 38 (diagnosis); Vaz-de-Mello et al. 2011: 24 (characters in key); Krajcik 2012: 204 (complete list of species, cited as subgenus of *Phanaeus* Macleay, 1819); Carvajal et al. 2011: 318 (list of species from Ecuador); Chamorro et al. 2018: 75 (characters in key), 93 (list of species of Ecuador).

###### Coprophanaeus (Coprophanaeus) callegarii

Taxon classificationAnimaliaColeopteraScarabaeidae

Arnaud, 2002

Plate 15D

Coprophanaeus (Coprophanaeus) callegarii Arnaud, 2002b: 4 (original description. Type locality: PERU, Iquitos).
Coprophanaeus (Coprophanaeus) callegarii : Arnaud 2002a: 53 (diagnosis, cited for Ecuador); Edmonds and Zídek 2010: 97 (characters in key, redescription); Carvajal et al. 2011: 320–321 (cited for Ecuador); Krajcik 2012: 204 (complete list of species, cited as subgenus of Phanaeus Macleay, 1819); Figueroa et al. 2014: 128 (distribution of records for Peru); Ratcliffe et al. 2015: 197 (cited for Peru); Chamorro et al. 2018: 93 (cited for Ecuador).

####### Type specimens.

Coprophanaeus (Coprophanaeus) callegarii Arnaud, 2002. The holotype (♂) is deposited at the CPFA (see Arnaud 2002b: 4). Locality: Iquitos, not examined.

####### Distribution.

Brazil, Ecuador, and Peru.

####### Records examined.

ORELLANA: Bloque 31, Parque Nacional Yasuni, 200 m (1 specimen MUTPL); Estación de Biodiversidad Tiputini, 220 m, Parque Nacional Yasuni (2 specimens MUTPL); Ginta Pompeya Sur-Iro, 250 m (1 specimen MUTPL); Cononaco, Bloque 16 YPF, Parque Nacional Yasuní, 250 m (1 specimen MUTPL).

####### Literature records.

SUCUMBÍOS: without specific locality (Arnaud 2002a: 53).

####### Temporal data.

Collected in May, June, September, and November.

####### Remarks.

Inhabits the lowland evergreen forests and the foothill evergreen forests of the Amazon region from 220–250 m a.s.l. Collected with pitfall traps baited with carrion and human feces. Arnaud (2002a) cites Succumbios [= Sucumbíos] province but no specific locality is provided.

###### Coprophanaeus (Coprophanaeus) conocephalus

Taxon classificationAnimaliaColeopteraScarabaeidae

(d’Olsoufieff, 1924)

Plate 16A

Phanaeus (C.) conocephalus d’Olsoufieff, 1924: 72 (original description. Type locality: Equateur [= Ecuador], Loja).
Coprophanaeus conocephalus : Blackwelder 1944: 209 (list of species from Latin America); Arnaud 1982a: 116 (list of types at the MNHN).
Coprophanaeus (Coprophanaeus) conocephalus : Arnaud 2002a: 48 (diagnosis); Edmonds and Zídek 2010: 59 (characters in key), 69 (redescription); Carvajal et al. 2011: 320–321 (cited for Ecuador); Krajcik 2012: 204 (complete list of species, cited as subgenus of Phanaeus Macleay, 1819); Arnaud, 2018: 6 (comment), 7 (figure 2a); Chamorro et al. 2018: 93 (cited for Ecuador).
Phanaeus roubali Balthasar, 1939e: 241 (original description); Blackwelder 1944: 210 (list of species of Latin America); Arnaud 1997: 7 (synonym of Coprophanaeusconocephalus Ols); Arnaud 2002a: 48 (cited as synonym); Edmonds and Zídek 2010: 69 (cited as synonym); Bezdek and Hajek 2013: 433 (catalog of types NMPC).

####### Type specimens.

Phanaeus (Coprophanaeus) conocephalus d’Olsoufieff, 1924. The holotype (♀) is deposited at the MNHN. Locality: Equateur Loja, examined.

**Holotype** (♀): “Equateur / Loja / Abbé Gaujon [p, black margin]”, “Ph. Conocephalus n. sp ♀ / det. G. Olsouffieff [hw and p]”, “HOLOTYPE [p, red label]”, “Coprophanaeus / conocephalus / Ols. / Holotype ♀ / P. Arnaud DET 1981 [p and hw]”.

*Phanaeusroubali* Balthasar, 1939. The holotype (♂) is deposited at the NMPC (see Bezdek and Hajek 2013: 433) Locality: Ecuador, Lola [= Loja], not examined.

####### Distribution.

Only known from Ecuador.

####### Records examined.

BOLIVAR: Bosque Protector Filo Palanga, 970 m (1 specimen MUTPL). CAÑAR: Javin. La Trancel [= La Troncal], 850–1300 m (3 specimens CEMT; 1 specimen MQCAZ). GUAYAS: Cerecita Pta Chapella (3 specimens CEMT; 1 specimen MQCAZ). LOJA: without specific locality (1 specimen MNHN). MANABÍ: El Aromo, Río de los Napos, 280 m (1 specimen CEMT); El Aromo, 370 m (1 specimen MUTPL); Refugio de Vida Silvestre Pacoche, 340 m (3 specimens CEMT); Puerto López, Río Blanco, 272 m (1 specimen MUTPL).

####### Literature records.

GUAYAS: Balzar (Edmonds and Zídek 2010: 71)

####### Temporal data.

Collected in January, February, April, August, and September.

####### Remarks.

Inhabits the coastal montane cloud forests and coastal evergreen foothill forests from 280–1300 m a.s.l. Collected with pitfall traps baited with carrion and human feces.

###### Coprophanaeus (Coprophanaeus) edmondsi

Taxon classificationAnimaliaColeopteraScarabaeidae

Arnaud, 1997

Plate 16B

Coprophanaeus edmondsi Arnaud, 1997: 5 (original description. Type locality: Pich. 4600 feet [= 1400 m], 23 km E of Alluriquín, Chiriboga Road).
Coprophanaeus edmondsi : Carvajal et al. 2011: 320–321 (cited for Ecuador).
Coprophanaeus (Coprophanaeus) edmondsi : Medina et al. 2001: 140 (cited for Colombia); Arnaud 2002a: 47 (diagnosis); Edmonds and Zídek 2010: 69 (cited as synonym of Coprophanaeusconocephalus d’Olsoufieff, 1924); Arnaud, 2018: 6 (revalidated name), 7 (figure 2b).

####### Type specimens.

*Coprophanaeusedmondsi* Arnaud, 1997. The holotype (♂) is deposited at the CMNC (formerly in A and H Howden collection, see Arnaud 1997: 6). Locality: Pich. 4600 feet [= 1400 m], 23 km E of Alluriquín, Chiriboga Road, not examined. One paratype is deposited in the MQCAZ. Javin, examined.

**Paratype** (♂): “ECUADOR (CAN) / Javin II. 92 / 850 – 1400m / P. Arnaud leg [p]”, “*Coprophanaeus / edmondsi* / 94 / P.ARNAUD DET /PARATYPE ♂ [p, pink margin]”.

####### Distribution.

Colombia and Ecuador.

####### Records examined.

CAÑAR: Javin, 850–1400 m (3 specimens CEMT; 1 specimen MQCAZ). CARCHI: Goaltal, Hacienda San Francisco, 1200 m (5 specimens MECN); Maldonado, 1830 m (2 specimens CEMT). PICHINCHA: Curipoglio, Cerro San Cristobal (1 specimen MUTPL); Mindo, 1500 m (1 specimen MUTPL); Estación Biológica la Hesperia (1 specimen MUTPL).

####### Literature records.

PICHINCHA: 4600 feet [1400 m], 23 km E de Alluriquin, Chiriboga Road (Arnaud, 1997: 6); Chiriboga Road 1200–1830, km 12–20 m (Arnaud, 1997: 6).

####### Temporal data.

Collected in February, March, June, September, October, and December.

####### Remarks.

Inhabits coastal foothill evergreen forests from 850–1200 m a.s.l. In the Andean region it has been registered in montane evergreen forests from 1400–1800 m a.s.l. Collected with pitfall traps baited with carrion and human feces.

###### Coprophanaeus (Coprophanaeus) jasius

Taxon classificationAnimaliaColeopteraScarabaeidae

(Olivier, 1789)

Scarabeus jasius Olivier, 1789: 109 (original description. Type locality: Cayenne, Curaçao).
Copris jasius : Olivier 1790: 156 (transferred to the genus Copris “Müller, 1764”, redescription); Sturm 1802: 66 (redescription).
Phanaeus jasius : Macleay 1819: 126 (transferred to the PhanaeusMacleay 1819, redescription); Gemminger and Harold 1869: 1018 (list, distribution, cited as C. Jasius Oliv); Nevinson 1892: 4 (list of species for the genus Phanaeus Macleay, 1819, written as PhanaeusJasius Olivier); Gillet 1911a: 83 (complete list of species, cited as PhanaeusJasius Ol).
Phanaeus (Coprophanaeus) jasius : d’Olsoufieff 1924: 24 (characters in key), 64 (distribution); Pessôa 1934: 296 (characters in key, redescription); Vulcano and Pereira 1967: 571 (characters in key).
Coprophanaeus jasius : Vitolo 2000: 599 (characters in key); Medina et al. 2001: 140 (cited for Colombia); Vitolo 2004: 291 (diagnosis); Hamel-Leigue et al. 2006: 17 (cited for Bolivia); Hamel-Leigue et al. 2009: 47 (comment); Boilly et al. 2016: 88 (figures 3a, 3b and 3c); 89 (characters in key); 90 (cited for Guyana).
Coprophanaeus (Coprophanaeus) jasius : Vaz-de-Mello 2000: 192 (cited for Brazil); Arnaud 2002a: 26 (diagnosis); Edmonds and Zídek 2010: 43 (characters in key); 48 (diagnosis); Carvajal et al. 2011: 320–321 (cited for Ecuador); Krajcik 2012: 204 (complete list of species, cited as species of the genus Phanaeus Macleay, 1819); Chamorro et al. 2018: 93 (cited for Ecuador).
Phanaeus satyrus Castelnau, 1840: 80 (original description); Gemminger and Harold 1869: 1018 (cited as Coprophanaeus Satyrus Casteln, synonym of Coprophanaeusjasius Olivier, 1790); Nevinson 1892: 1 (cited and proposed as synonym of Phanaeus Acrisinus Macleay); Gillet 1911a: 84 (cited as synonym of Coprophanaeusjasius Olivier); d’Olsoufieff 1924: 141 (cited as synonym of Coprophanaeusjasius Olivier); Pessôa 1934: 296 (cited as synonym of Coprophanaeusjasius Olivier); Arnaud 2002a: 26 (cited as Phanaeussatyrus Laporte, 1840 synonym of Coprophanaeusjasius Olivier); Edmonds and Zídek 2010: 48 (synonym of Coprophanaeus (Coprophanaeus) jasius (Olivier, 1789)).

####### Type specimens.

*Scarabeusjasius* Olivier, 1789. The neotype is deposited at the MNHN. Guyane, Cayenne, examined.

**Neotype** (♂): “VII. 78 [hw]”, “GUYANE F se / CAYENNE / La Chaumière / Leg: P. ARNAUD [p, black margin]”, “Scarabeus / jasius Ol. / P. ARNAUD DET 2001 / NEOTYPE ♂”.

*Phanaeussatyrus* Castelnau, 1840. Type material not examined.

####### Distribution.

Brazil, Colombia, Guyana, and Ecuador.

####### Literature records.

NAPO: without specific locality (Arnaud, 2002a: 26).

####### Temporal data.

It is not known when this species was collected.

####### Remarks.

Arnaud (2002a) cited Coprophanaeus (Coprophanaeus) jasius (Olivier, 1789) as a species distributed in the Napo province with no specific locality. Although Edmonds and Zídek (2010) did not report this species from Ecuador, it could occur in the Amazon lowlands.

###### Coprophanaeus (Coprophanaeus) morenoi

Taxon classificationAnimaliaColeopteraScarabaeidae

Arnaud, 1982

Plate 16C

Coprophanaeus morenoi Arnaud, 1982b: 121 (original description. Type locality: Equateur [= Ecuador], km 36 route Sto Domingo-Quevedo).
Coprophanaeus morenoi : Vitolo 2000: 599 (characters in key); Medina et al. 2001: 140 (cited for Colombia); Arnaud 2002a: 42 (diagnosis); Vitolo 2004: 290 (redescription); Donoso et al. 2009: Appendix II. 16 (catalog of type MQCAZ); Edmonds and Zídek 2010: 57 (characters in key), 60 (redescription); Kohlmann and Solís 2012: 46 (cited for Ecuador); Krajcik 2012: 204 (complete list of species, cited as subgenus of Phanaeus Macleay, 1819);
Coprophanaeus (Coprophanaeus) morenoi : Carvajal et al. 2011: 320–321 (cited for Ecuador); Chamorro et al. 2018: 83 (figure 6C), 93 (cited for Ecuador).

####### Type specimens.

*Coprophanaeusmorenoi* Arnaud, 1982. The holotype (♂) is deposited at the MNHN. Locality: Equateur [= Ecuador], Sto. Domingo km 36, examined. One Paratype is deposited in the MQCAZ, examined.

**Holotype** (♂): “EQUATEUR / STO DOMINGO K. 36 / JANV. 1982 / P&L ARNAUD coll [p and hw, black margin]”, “Coprophanaeus / morenoi Mihi / HOLOTYPE ♂ / P. ARNAUD DET 1982 [p and hw, pink margin]”, “HOLOTYPE [p, red label]”.

**Paratype** (♀): “ECUADOR (PICH) / TINA LANDIA / I. 82 650 m / P & L ARNAUD leg [p]”, “Coprophanaeus / morenoi Mihi / P. ARNAUD DET / PARATYPE ♀ [p and hw, pink margin]”.

####### Distribution.

Colombia, Costa Rica, Ecuador, Nicaragua, and Panama.

####### Records examined.

CARCHI: Tobar Donoso, 300 m (8 specimens MECN). COTOPAXI: Guasaganda La Mana, 500 m (1 specimen CEMT; 3 specimens MQCAZ). ESMERALDAS: Colón del Onzole (17 specimens MECN); Charco Vicente (13 specimens MGO-UC; 28 specimens MECN); Gualpi (15 specimens MECN); Gualpi Pajonal (17 specimens MECN); Palma Real (1 specimen MGO-UC; 15 specimens MECN); Playa de Oro (1 specimen MUTPL; 10 speciemens MECN); Playa de Oro, Padre Santo (19 specimens MGO-UC; 31 specimens MECN); Playa de Oro, Estero Pote, 200 m (3 specimen CEMT; 16 specimens MECN); road Ibarra-San Lorenzo, El Placer, 670 m (1 specimen CEMT). PICHINCHA: Guayabilla Río Guayllabamba, Manduriacus, 520 m (2 specimens MUTPL); Llurimaguas Río Guayllabamba, Pedro Vicente Maldonado, 290 m (1 specimen MUTPL); Mangaloma, San Miguel de los Bancos, 820 m (1 specimen MUTPL); Tortugo Río Guayllabamba, Pedro Vicente Maldonado, 450 m (1 specimen MUTPL). SANTO DOMINGO DE LOS TSÁCHILAS: Tinalandia, 650 m (1 specimen MQCAZ).

####### Literature records.

ESMERALDAS: 11 km SE San Lorenzo, La Chiquita Sta, 5 m (Edmonds and Zídek 2010: 62; Kohlmann and Solís 2012: 46); Yalare (Edmonds and Zídek 2010: 62; Kohlmann and Solís 2012: 46); Punta Venado (Edmonds and Zídek 2010: 62; Kohlmann and Solís 2012: 46); Playa de Oro (Edmonds and Zídek 2010: 62; Kohlmann and Solís 2012: 46); La Concordia (Edmonds and Zídek 2010: 62); Palma Real (Edmonds and Zídek 2010: 62). GUAYAS: Los Ceibos (Kohlmann and Solís 2012: 46); Borbón, 25 m (Edmonds and Zídek 2010: 62); San Miguel (Edmonds and Zídek 2010: 62). PICHINCHA: [= LOS RÍOS]: Station Río Palenque (Arnaud 1982b: 122); 47 km S Sto. Domingo, Río Palenque (Kohlmann and Solís 2012: 46); 113 km NW Quito on Puerto Quito road, 800 m (Edmonds and Zídek 2010: 62). LOS RÍOS: Quevedo (Edmonds and Zídek 2010: 62); Quevedo Pichilinge (Kohlmann and Solís 2012: 46); Río Palenque Research Station, 200 m (Edmonds and Zídek 2010: 62). SANTO DOMINGO DE LOS TSÁCHILAS: km 36 route Sto. Domingo-Quevedo (Arnaud 1982b: 122); Tina Landia [= Tinalandia] (Env. Sto. Domingo) (Arnaud 1982b: 122); i1 km E Tinalandia, 600 m (Edmonds and Zídek 2010: 62) 16 km E Sto. Domingo, Tinalandia (Kohlmann and Solís 2012: 46).

####### Temporal data.

Collected every month of the year.

####### Remarks.

Inhabits coastal lowland evergreen forests and coastal evergreen foothill forests from 5–850 m a.s.l. Species was collected with flight interception traps and pitfall traps baited with carrion and human feces.

###### Coprophanaeus (Coprophanaeus) ohausi

Taxon classificationAnimaliaColeopteraScarabaeidae

(Felsche, 1911)

Plate 16D

Phanaeus ohausi Felsche, 1911: 138 (original description. Type locality: Oscordillere Teremotillo, zwichen Baños und Canelos [= between Baños and Canelos]).
Phanaeus ohausi : Gillet 1911a: 85 (complete list of species, cited as PhanaeusOhausi Felsche); d’Olsoufieff 1924: 74 (distribution).
Coprophanaeus ohausi : Blackwelder 1944: 209 (list of species of Latin America); Vitolo 2000: 599 (characters in key); Medina et al. 2001: 140 (cited for Colombia); Ratcliffe et al. 2015: 197 (cited for Peru).
Coprophanaeus (Coprophanaeus) ohausi : Arnaud 2002a: 44 (diagnosis); Vitolo 2004: 291 (redescription); Edmonds and Zídek 2010: 97 (characters in key); 104 (redescription); Carvajal et al. 2011: 320 (cited for Ecuador); Krajcik 2012: 204 (complete list of species, cited as subgenus of Phanaeus Macleay, 1819); Figueroa et al. 2014: 128 (distribution of records for Peru); Chamorro et al. 2018: 93 (cited for Ecuador).
Coprophanaeus (Coprophanaeus) florenti Arnaud, 2002b: 5 (original description); Edmonds and Zídek 2010: 104 (synonym of Coprophanaeusohausi Felsche, 1911); Carvajal et al. 2011: 320–321 (cited for Ecuador).

####### Type specimens.

*Phanaeusohausi* Felsche, 1911. The lectotype (♂) is deposited at the SMTD (see Edmonds and Zídek 2010: 104). Locality: without specific locality, not examined.

Coprophanaeus (Coprophanaeus) florenti Arnaud, 2002b. The holotype (♂) is deposited at the CPFA (see Arnaud 2002b: 4). Locality: Napo, Rte de Loretko Pk 21, 1200 m, not examined.

####### Distribution.

Colombia, Ecuador, and Peru.

####### Records examined.

LOJA: Loja-Zamora, 1400 m (4 specimens CEMT). MORONA SANTIAGO: Comunidad Ángel Rouby, 1300–1700 m, Cordillera del Kutukú (1 specimen MUTPL, 18 specimens MECN); Comunidad Untsuants, 1100 m, Cordillera del Kutukú (14 specimens MECN); Cumanda (1 specimen CEMT); Chiguinda Río Blanco, 1750 m (1 specimen MUTPL); Nuevo Israel, Cordillera del Kutukú, 1290 m (1 specimen MUTPL). NAPO: Quebrada Granadillas Bosque Protector la Cascada, 1300 m, Parque Nacional Sumaco (1 specimen MUTPL); Pacto Sumaco, 1620 m (3 specimens MUTPL). NAPO: Cotundo, 1070 m Río Osayacu, sector Shamato (2 specimens MUTPL); ORELLANA: Ines Arango road Tiwino-río Shiripuno, 250 m (3 specimens MECN). PASTAZA: Chuyayacu Oleoducto km 25, 200 m (1 specimen MUTPL); Ñemenguno, 280 m (2 specimens CEMT). PASTAZA: Mera, Estación Biológica de la UTE Pindo Mirador, 1000 m (1 specimen MUTPL). SUCUMBÍOS: Gonzalo Pizarro, Simon Bolivar, 1200 m (4 specimens MECN). TUNGURAHUA: Baños El Topo, 1590 m (33 specimens CEMT). ZAMORA CHINCHIPE: Cordillera la Curintza, Parque Nacional Podocarpus, 1790 m (9 specimens MECN); RVS El Zarza campamento las Peñas conseción El Colibri 1530 m, Cordillera del Cóndor (1 specimen MUTPL); Romerillos Sendero Nangaritza, 2200 m, Parque Nacional Podocarpus (12 specimens MECN); Tundayme campamento Mirador La Mina, 1320 (1 specimen MUTPL); Tundayme campamento Mirador, Tambo 3, 1055 m (2 specimens MUTPL); Tundayme campamento Mirador, Condor Mirador, 1420 m (1 specimen MUTPL); Zamora km 12–18, 1500 m (5 specimens MECN); Zurmi Comunidad Miazi, 1380 m (1 specimen MEPN); Zurmi, Pachikuntza, 1685 m (1 specimen MEPN); Zurmi, Comunidad La Wants, 1010 m (1 specimen MEPN).

####### Literature records.

MORONA SANTIAGO: Angel Rouby, Cordillera Cutucú [= Kutukú] (Edmonds and Zídek 2010: 104); Cordillera Cutucú [= Kutukú] (Edmonds and Zídek 2010: 104). NAPO: Río Hollin, 1068 m (Edmonds and Zídek 2010: 104); Aliñahui (Edmonds and Zídek 2010: 104), Puerto Napo (Edmonds and Zídek 2010: 104). NAPO: Rte de Loreto Pk 21, 1200 m (Arnaud, 2002b: 5, cited as Coprophanaeus (Coprophanaeus) florenti Arnaud, 2002); Rte de Loreto Pk 11, 1380 m (Arnaud, 2002b: 5, cited as Coprophanaeus (Coprophanaeus) florenti Arnaud, 2002). NAPO [= ORELLANA]: Loreto road, 7.9 km E Narupa junction, 1380 m. (Edmonds and Zídek 2010: 104); Yasuní National Park, Yasuní Research Station, 215 m (Edmonds and Zídek 2010: 104); Limoncocha, 250m (Edmonds and Zídek 2010: 104); Puerto Francisco de Orellana [= El Coca] (Edmonds and Zídek 2010: 104); km 11.1 road Sarayacu-Loreto, 1200 m (Edmonds and Zídek 2010: 104). PASTAZA: 22 km SE Puyo, 900 m (Edmonds and Zídek 2010: 104); 17 km N Puyo, Llandia 1000 m (Edmonds and Zídek 2010: 104). TUNGURAHUA: 6 km and 8 km E Río Negro, 1400 m (Edmonds and Zídek 2010: 104). ZAMORA CHINCHIPE: Bombuscaro, Parque Nacional Podocarpus 1146 m (Edmonds and Zídek 2010: 104). UNDETERMINED PROVINCE: Oscordillere Teremotillo, between Baños and Canelos (Felsche 1911: 138)

####### Temporal data.

Collected in January, February, March, April, May, July, September, November, and December.

####### Remarks.

Inhabits the lowland evergreen forests, evergreen foothill forests, and lower evergreen montane forests throughout the Amazonian range from 225–1700 m a.s.l. In the Andean region, it was registered in the montane cloud forests from 1800–2200 m a.s.l. Species was collected with flight interception traps and pitfall traps baited with carrion and human feces.

###### Coprophanaeus (Coprophanaeus) suredai

Taxon classificationAnimaliaColeopteraScarabaeidae

Arnaud, 1996

Plate 17A

Coprophanaeus suredai Arnaud, 1996: 6 (original description. Type locality: BRAZIL, Amazonas Río Javari).
Coprophanaeus suredai : Medina et al. 2001: 140 (cited for Colombia); Arnaud 2002a: 51 (diagnosis); Hamel-Leigue et al. 2009: 58 (cited for Bolivia); Krajcik 2012: 204 (complete list of species, cited as subgenus of Phanaeus Macleay, 1819); Ratcliffe et al. 2015: 197 (cited for Peru).
Coprophanaeus (Coprophanaeus) suredai : Vaz-de-Mello 2000: 192 (cited for Brazil); Edmonds and Zídek 2010: 97 (characters in key); 102 (diagnosis); Carvajal et al. 2011: 320–321 (cited for Ecuador); Figueroa et al. 2014: 128 (distribution of records for Peru); Chamorro et al. 2018: 93 (cited for Ecuador).

####### Type specimens.

*Coprophanaeussuredai* Arnaud, 1996. The holotype (♂) is deposited at the CPFA (see Arnaud 1996: 7). Locality: Amazonas Río Javari, not examined.

####### Distribution.

Brazil, Colombia, Ecuador, and Peru.

####### Literature records.

SUCUMBÍOS: Lago Agrio (Arnaud 1996: 7).

####### Temporal data.

Collected in August.

####### Remarks.

There are no specimens of this species in entomological collections in Ecuador. However, Arnaud (1996) cited Lago Agrio as its distribution and it is possible that it may inhabit other lowland evergreen forests. Edmonds and Zídek (2010) also cited several records from Amazon localities in Colombia and Peru. The collection method is unknown.

###### Coprophanaeus (Coprophanaeus) telamon

Taxon classificationAnimaliaColeopteraScarabaeidae

(Erichson, 1847)

Plate 17B

Phanaeus telamon Erichson, 1847: 106 (original description, without type locality).
Phanaeus telamon : Gemminger and Harold 1869: 1019 (list, distribution, written as *C. Telamon* Erichs); Kirsch 1873: 341 (cited for Peru, written as *C. Telamon* Erichs); Bates 1887: 56 (diagnosis); Nevinson 1892: 7 (list of species for the genus Phanaeus Macleay, 1819, written as *C. Telamon* Erichson); Gillet 1911a: 86 (complete list of species, written as *C. Telamon* Er); d’Olsoufieff 1924: 26 (characters in key), 68 (distribution); Balthasar 1941: 350 (cited for Peru); Blackwelder 1944: 210 (list of species for Latin America); Balthasar 1951: 336 (cited for Peru); Pereira 1953: 391 (catalog of species).
Phanaeus (Coprophanaeus) telamon : Pessôa 1934: 296 (characters in key); 299 (redescription); Martínez 1947: 114 (cited); Vulcano and Pereira 1967: 572 (characters in key).
Coprophanaeus (Coprophanaeus) telamonvar.telamon : Pereira and Martínez 1956b: 233 (cited as new combination); Arnaud 2002a: 35 (redescription); Hamel-Leigue et al. 2006: 17 (cited for Bolivia); Carvajal et al. 2011: 320–321 (cited for Ecuador); Ratcliffe et al. 2015: 197 (cited for Peru).
Coprophanaeus telamon : Vitolo 2000: 599 (characters in key); Medina et al. 2001: 140 (cited for Colombia); Vitolo 2004: 290 (redescription); Krajcik 2012: 204 (complete list of species, cited as species of the genus Phanaeus Macleay, 1819).
Coprophanaeus (Coprophanaeus) telamon : Vaz-de-Mello 2000: 192 (cited for Brazil); Edmonds and Zídek 2010: 77 (characters in key); 91 (cited as recombination, redescription); Cupello and Vaz-de-Mello 2013a: 367 (distribution); Figueroa et al. 2014: 127 (distribution of records for Peru); Chamorro et al. 2018: 93 (cited for Ecuador).

####### Type specimens.

*Phanaeustelamon* Erichson, 1847. The holotype (♂) is deposited in the SMTD (see Edmonds and Zídek 2010: 91). Locality: without specific locality, not examined.

####### Distribution.

Bolivia, Brazil, Colombia, Ecuador, Peru, and Venezuela.

####### Records examined.

MORONA SANTIAGO: Comunidad Untsuants, 700 m, Cordillera del Kutukú (21 specimens MECN); Nuevo Israel, Cordillera del Kutukú (1 specimen MUTPL); km 8 road Mendez-Paute, 1250 m (1 specimen CEMT). NAPO: La Merced de Jondachi Río Jondachi, 1100 m (1 specimen MUTPL); Pacto Sumaco, Cotundo, 1500 m (1 specimen MUTPL); Shiqui cerca al Tena, 485 m, Pungarayacu (1 specimen MQCAZ); Tena, 400 m (15 specimens CEMT). ORELLANA: Bloque 16, Parque Nacional Yasuní (1 specimen MUTPL); Bloque 31, Parque Nacional Yasuní, 210 m (3 specimens MECN); Dayuma Campo Hormiguero, plataforma Hormiguero, 320 m (2 specimens MUTPL); Dayuma Campo Palanda, LLumpac, 295 m (1 specimen MGO-UC); Dayuma, Campo Palanda Yuca 13, 255 m (1 specimen MGO-UC); Dayuma plataforma Ungurahua, 300 m (1 specimens MUTPL); Eden Yuturí Bloque 15, 225 m (1 specimen MUTPL); Estación Biológica Yasuní, 215 m (3 specimens MQCAZ); Estación de Biodiversidad Tiputini Torre, 220 m, Parque Nacional Yasuni (3 specicmen MUTPL); Rodrigo Borja IAMOE (7 specimens CEMT; 19 specimens MECN); San Sebastian del Coca, 345 m, Comuna Guataraco Campo Pata (2 specimens MUTPL). PASTAZA: Bosque Protector Oglán Alto, 660–810 m (2 specimens MUTPL); Nuevo San Jose del Curaray, 245 m (1 specimen MUTPL); Mera Estación Pindo Mirador UTE, 1000 m (4 specimens CEMT). SUCUMBÍOS: Aucayacu Río El Eno, 16 km de Lago Agrio, 290 m (1 specimen MUTPL); Bermejo plataforma ER-A road to Lumbaqui (1 specimen MUTPL); Laguna Grande de Cuyabeno, 250 m, Reserva de Producción Faunística Cuyabeno (10 specimens MECN); Pacayacu Campo Libertador, 260 m (2 specimens MUTPL); Sacha, 270 m (1 specimen MUTPL); Sansahuari, 255–290 m, Pozo Singüe (1 specimen MUTPL); Tarapoa plataforma Fanny 18B60, 245 m (1 specimen MUTPL); Tarapoa, Nuevo Manabí, 270 m (1 specimen MUTPL). ZAMORA CHINCHIPE: Tundayme, 800 m (2 specimens MUTPL); Tundayme, campamento Mirador, Tambo 3, 1055 m (1 specimen MUTPL); Zurmi, Comunidad La Wants, 1010 m (2 specimens MGO-UC; 1 specimen MEPN).

####### Literature records.

MORONA SANTIAGO: Untsuants, Cordillera de Cutucú [= Kutukú], 600 m (Edmonds and Zídek 2010: 92). NAPO: 0.6 km E Río Arajuno, 380 m (Edmonds and Zídek 2010: 93); 3.3 km E Puerto Napo (Edmonds and Zídek 2010: 92); 12 km WSW Tena, 600 m (Edmonds and Zídek 2010: 92); 20 km S Tena (Edmonds and Zídek 2010: 92); 21 km E Puerto Napo, Jatun Sacha Biological Station (Edmonds and Zídek 2010: 92); 24.5 km E Ahuano (Edmonds and Zídek 2010: 93); 29 km E, 1.5 km N San Pedro de Arajuno, 360 m (Edmonds and Zídek 2010: 93); Aliñahui, 24 km E Atahualpa (Edmonds and Zídek 2010: 93); Archidona (Edmonds and Zídek 2010: 93); Ávila (Pereira and Martínez 1956b: 234); Talag Pimpilata, 750 m (Edmonds and Zídek 2010: 92); Tena, 400 m (Edmonds and Zídek 2010: 92). ORELLANA: Daimi (Edmonds and Zídek 2010: 93); Payamino Research Station, 400 m (Edmonds and Zídek 2010: 93); Puerto Franciso de Orellana [= El Coca] (Edmonds and Zídek 2010: 92); Tiputini Biological Station, 220 m (Edmonds and Zídek 2010: 93); Yampuna (Edmonds and Zídek 2010: 93); Yasuní Biological Station, 215 m (Edmonds and Zídek 2010: 92). PASTAZA: 22 km SE Puyo (Edmonds and Zídek 2010: 93); Llandia, 17 km N Puyo, 1000 m (Edmonds and Zídek 2010: 93); Puyo, 940 m (Edmonds and Zídek 2010: 93). SUCUMBÍOS: 2 km N Limoncocha, 250 m (Edmonds and Zídek 2010: 93); Dureno, Río Aguarico (Edmonds and Zídek 2010: 92); Lago Agrio, 200 m (Edmonds and Zídek 2010: 93); Limoncocha, 250 m (Edmonds and Zídek 2010: 93). TUNGURAHUA: Baños (Edmonds and Zídek 2010: 93); Río Negro (Edmonds and Zídek 2010: 93).

####### Temporal data.

Collected in collected every month of the year.

####### Remarks.

Inhabits the lowland evergreen forests, varzea forests, the foothill evergreen forests and lower evergreen montane forests in the Amazonian range from 200–1500 m a.s.l. Species was collected with flight interception traps and pitfall traps baited with carrion and human feces.

#### Genus *Cryptocanthon* Balthasar, 1942

*Cryptocanthon* Balthasar, 1942: 36 (original description. Type species: *Cryptocanthonparadoxus* Balthasar, 1942 by primary monotypy).

*Cryptocanthon*: Pereira and Martínez 1956a: 96 (characters in key), 181 (distribution); Halffter 1961: 231 (characters in key); Vulcano and Pereira 1964: 673 (catalog of species); Halffter and Matthews 1966: 261 (catalog, distribution); Vulcano and Pereira 1967: 548 (characters in key); Howden 1973: 39 (redescription); Halffter and Martínez 1977: 35 (characters in key), 60 (list of species); Howden and Young 1981: 13 (characters in key), 39 (redescription); Halffter and Edmonds 1982: 139 (catalog, distribution); Medina and Lopera 2000: 306 (characters in key); Vaz-de-Mello 2000: 192 (list of species for Brazil); Medina et al. 2001: 136 (list of species for Colombia); Cook 2002: 4 (revision); Ratcliffe 2002: 13 (list of species for Panama); Morón 2003: 24 (redescription); Medina et al. 2003: 65 (distribution); Hamel-Leigue et al. 2006: 14 (list of species for Bolivia); Vaz-de-Mello et al. 2011: 23 (characters in key); Carvajal et al. 2011: 118 (diagnosis), 316 (list of species from Ecuador); Krajcik 2012: 82 (complete list of species); Solís and Kohlmann 2012: 3 (list of species from Costa Rica); Boilly and Vaz-de-Mello 2013: 106 (characters in key); Chamorro et al. 2018: 74 (characters in key), 93 (list of species of Ecuador).

##### Cryptocanthon curticrinis

Taxon classificationAnimaliaColeopteraScarabaeidae

Cook, 2002

Plate 17C

Cryptocanthon curticrinis Cook, 2002: 59 (original description. Type locality: Ecuador, Napo Prov. Limoncocha, 250 m [= currently Sucumbíos Prov]).
Cryptocanthon curticrinis : Carvajal et al. 2011: 316–317 (cited for Ecuador); Krajcik 2012: 82 (complete list of species); Chamorro et al. 2018: 93 (cited for Ecuador).

###### Type specimens.

*Cryptocanthoncurticrinis* Cook, 2002. The holotype (♂) is deposited at the CMNC (see Cook 2002: 59). Locality: Napo Prov. Limoncocha, not examined.

###### Distribution.

Only known from Ecuador.

###### Literature records.

NAPO [= ORELLANA]: Río Tiputini, Parque Nacional Yasuní (Cook 2002: 59). NAPO [= SUCUMBÍOS]: Limoncocha, 250 m (Cook 2002: 59).

###### Temporal data.

Collected in June.

###### Remarks.

Inhabits the lowland evergreen forests of the Amazon region at 250 m a.s.l. According to Cook (2002), this species was collected by sifting leaf litter and processing samples with Berlese method.

##### Cryptocanthon genieri

Taxon classificationAnimaliaColeopteraScarabaeidae

Cook, 2002

Plate 17D

Cryptocanthon genieri Cook, 2002: 66 (original description. Type locality: Ecuador, Napo, 1200 m, km 7.3 Sarayacu-Loreto Rd).
Cryptocanthon genieri : Carvajal et al. 2011: 316–317 (cited for Ecuador); Krajcik 2012: 82 (complete list of species); Chamorro et al. 2018: 93 (cited for Ecuador).

###### Type specimens.

*Cryptocanthongenieri* Cook, 2002. The holotype (♂) is deposited at the CMNC (see Cook 2002: 66). Locality: Napo, 1200 m, km 7.3 Sarayacu-Loreto Rd, not examined.

###### Distribution.

Only known from Ecuador.

###### Literature records.

NAPO: km 7.3 Sarayacu-Loreto, 1200 m (Cook 2002: 66); Jatun Sacha Estación Biológica, 21 km E of Puerto Napo, 400 m (Cook 2002: 66). PASTAZA: 17 km N del Puyo, Llandia, 1000 m (Cook 2002: 66); 25 km N del Puyo, 1000 m (Cook 2002: 66).

###### Temporal data.

Collected in July.

###### Remarks.

Inhabits the foothill evergreen forests of the Amazon region from 1000–1200 m a.s.l. According to Cook (2002), this species was collected with pitfall traps baited with human feces.

##### Cryptocanthon napoensis

Taxon classificationAnimaliaColeopteraScarabaeidae

Cook, 2002

Plate 18A

Cryptocanthon napoensis Cook, 2002: 76 (original description. Type locality: Ecuador, Napo, 4200’/ 17 km NE Baeza; 4 km SW Chaco).
Cryptocanthon napoensis : Carvajal et al. 2011: 316–317 (cited for Ecuador); Krajcik 2012: 82 (complete list of species); Chamorro et al. 2018: 81 (figures 4A and 4E), 93 (cited for Ecuador).

###### Type specimens.

*Cryptocanthonnapoensis* Cook, 2002. The holotype (♂) is deposited at the CMNC (see Cook 2002: 76). Locality: Ecuador, Napo, 4200’/ 17 km NE Baeza; 4 km SW Chaco, not examined.

###### Distribution.

Only known from Ecuador.

###### Records examined.

NAPO: Las Palmas, Cuchilla San Pedro, 2000 m (2 specimens CEMT; 12 specimens MUTPL).

###### Literature records.

NAPO: 7 km S Baeza 2000 m (Cook 2002: 76); 17 km NE Baeza; 4 km SW Chaco, 4200’ (Cook 2002: 76).

###### Temporal data.

Collected in February, March, and April.

###### Remarks.

Inhabits the montane cloud forests of the Andean region at 1300 m a.s.l. Collected with pitfall traps baited with human feces.

##### Cryptocanthon otonga

Taxon classificationAnimaliaColeopteraScarabaeidae

Cook, 2002

Plate 18B

Cryptocanthon otonga Cook, 2002: 77 (original description. Type locality: Cotopaxi, Otonga, 2000 m).
Cryptocanthon otonga : Donoso et al. 2009: Appendix II. 16 (catalog of types of the MQCAZ); Carvajal et al. 2011: 316–317 (cited for Ecuador); Krajcik 2012: 82 (complete list of species); Chamorro et al. 2018: 93 (cited for Ecuador).

###### Type specimens.

*Cryptocanthonotonga* Cook, 2002. The holotype (♂) is deposited at the MQCAZ. Locality: Otonga, 2000 m, examined.

**Holotype** (♂): “COTOPAXIECUADOR / OTONGA 2000m / 0°25'S, 79°0'W / 24MAR 1999 TEnríquez [p]”, “Ex: Primary forest / Pitfall Trap / Human dung [p]”, “HOLOTYPE / Cryptocanthon / otonga / Cook [p, red label]”.

###### Distribution.

Only known from Ecuador.

###### Records examined.

COTOPAXI: Bosque Integral Otonga, 2000 m (9 specimens CEMT); Bosque Integral Otonga 2000 m (2 specimens MQCAZ).

###### Literature records.

COTOPAXI: Bosque Integral Otonga (Cook 2002: 77; Donoso et al. 2009: Appendix II. 16).

###### Temporal data.

Collected in February, March, April, and August.

###### Remarks.

Inhabits the montane cloud forests of the Andean region at 2000 m a.s.l. According to Cook (2002), this species was collected with pitfall traps baited with human excrements and NTP80 traps (necrotrap).

##### Cryptocanthon paradoxus

Taxon classificationAnimaliaColeopteraScarabaeidae

Balthasar, 1942

Plate 18C

Cryptocanthon paradoxus Balthasar, 1942: 37 (original description. Type locality: Loja, Villonaco).
Cryptocanthon paradoxus : Vulcano and Pereira 1967: 551 (distribution); Howden 1973: 40 (characters in key, redescription); Cook 2002: 10 (characters in key), 78 (revision); Carvajal et al. 2011: 316–317 (cited for Ecuador); Bezdek and Hajek 2011: 366 (catalog of types NMPC); Krajcik 2012: 82 (complete list of species); Albuquerque et al. 2017 (cited for Peru); Chamorro et al. 2018: 93 (cited for Ecuador).

###### Type specimens.

*Cryptocanthonparadoxus* Balthasar, 1942. The holotype (♂) is deposited at the NMPC. Locality: Loja Villonaco, examined.

**Holotype** (♂): “(Ecuad.) [p]”, “Loja Villonaco / F. Ohs. 23.8.05 [p]”, “TYPUS [p, red label, black margin]”, “Cryptocanthon / paradoxus / n. gen. n. sp. / mihi / Dr. V. Balthasar det. [p and hw]”, “Cryptocanthon / paradoxus Balth. [hw, green label, black margin]”.

###### Distribution.

Ecuador and Peru.

###### Records examined.

LOJA: Villonaco (1 specimen NMPC).

###### Temporal data.

Collected in August.

###### Remarks.

Inhabits the montane cloud forests of the Andean region at 2000 m a.s.l. The collection method is unknown.

##### Cryptocanthon urguensis

Taxon classificationAnimaliaColeopteraScarabaeidae

Cook, 2002

Plate 18D

Cryptocanthon urguensis Cook, 2002: 86 (original description. Type locality: Ecuador, Napo, Misahualli Rumi Urgu Mt).
Cryptocanthon urguensis : Carvajal et al. 2011: 316–317 (cited for Ecuador); Krajcik 2012: 82 (complete list of species); Chamorro et al. 2018: 93 (cited for Ecuador).

###### Type specimens.

*Cryptocanthonurguensis* Cook, 2002. The holotype (♂) is deposited at the CMNC (see Cook 2002: 86). Locality: Ecuador, Napo, Misahualli Rumi Urgu Mt, not examined.

###### Distribution.

Only known from Ecuador

###### Literature records.

NAPO: Misahualli Rumi Urgu Mt (Cook 2002: 86).

###### Temporal data.

Collected in February.

###### Remarks.

Inhabits the foothill evergreen forests of the Amazon region. The collection method is unknown.

#### Genus *Deltochilum* Eschscholtz, 1822

*Deltochilum* Eschscholtz, 1822: 37 (original description. Type species: *Deltochilumdentipes* Eschscholtz, 1822 by monotypy).

*Deltochilum*: Agassiz 1846: 341 (catalog); Burmeister 1848: 134 (redescription); Lacordaire 1856: 79 (redescription); LeConte 1861: 125 (characters in key); Gemminger and Harold 1869: 995 (list of species, distribution); Burmeister 1873a [= 1874]: 408 (redescription); Lansberge 1874b: 188 (characters in key); Kolbe 1893: 191 (redescription); Shipp 1897: 194 (comment); Gillet 1911a: 35 (complete list of species); Lucas 1920: 228 (catalog, distribution); Paulian 1938: 235 (characters in key), 237 (redescription); Pessôa and Lane 1941: 411 (characters in key), 426 (redescription); Blackwelder 1944: 202 (list of species of Latin America); Lane 1946: 171 (comment); Roze 1955: 43 (list of species from Venezuela); Pereira and Martínez 1956a: 96 (characters in key); Martínez 1959: 50 (list of species for Argentina); Halffter 1961: 231 (characters in key); Vulcano and Pereira 1964: 639 (catalog of species); Halffter and Matthews 1966: 261 (catalog, distribution); Vulcano and Pereira 1967: 549 (characters in key); Halffter and Martínez 1977: 36 (characters in key); Howden and Young 1981: 14 (characters in key); 36 (redescription); Halffter and Edmonds 1982: 139 (catalog, distribution); Medina and Lopera 2000: 311 (characters in key); Vaz-de-Mello 2000: 192 (list of species from Brazil); Medina et al. 2001: 136 (list of species from Colombia); Arnett et al. 2002: 49 (characters in key); Ratcliffe 2002: 13 (list of species from Panama); Morón 2003: 26 (redescription); Medina et al. 2003: 64 (distribution); Hamel-Leigue et al. 2006: 14 (list of species from Bolivia); Vaz-de-Mello et al. 2011: 25 (characters in key); Carvajal et al. 2011: 123 (diagnosis), 316 (list of species from Ecuador); Krajcik 2012: 88 (complete list of species); Solís and Kohlmann 2012: 4 (list of species from Costa Rica); Boilly and Vaz-de-Mello 2013: 107 (characters in key); Ratcliffe et al. 2015: 195 (list of species from Peru); Chamorro et al. 2018: 76 (characters in key), 93–94 (list of species of Ecuador).

*Anamnesis* Vigors, 1826: 510 (original description. Type species: *Anamnesis Macleayii* Vigors, 1826); Agassiz 1846: 58 (catalog); Gemminger and Harold 1869: 995 (cited as synonym, cited as *Annamesis* Vigors); Shipp 1897: 194 (comment); Gillet 1911a: 35 (cited as synonym, cited as *Annamesis* Vigors); Paulian 1938: 237 (cited as synonym, cited as *Annamesis* Vigors); Blackwelder 1944: 202 (cited as synonym, cited as *Annamesis* Vigors); Lane 1946: 171 (comment); Pereira and Martínez 1956a: 120 (cited as synonym); Martínez 1959: 50 (cited as synonym); Vulcano and Pereira 1964: 639 (cited as synonym); Ratcliffe 2002: 13 (cited as synonym); Solís and Kohlmann 2012: 4 (cited as synonym, cited as *Annamesis* Harold, 1869).

*Meghyboma* Kolbe, 1893: 192 (original description. Type species: *Deltochilumdentipes* Eschscholtz, 1822); Shipp 1897: 194 (comment); Paulian 1938: 243 (characters in key); 246 (redescription); Blackwelder 1944: 202 (cited as subgenus of *Deltochilum* Eschz.); Lane 1946: 174 (synonym of *Deltochilum* Eschz.); Halffter and Edmonds 1982: 139 (cited as subgenus of *Deltochilum* Eschscholtz, 1822); Solís and Kohlmann 2012: 4 (cited as synonym).

*Eudactylides* Paulian, 1939: 8 (original description. Type species: *Deltochilumcarinatum* Westwood, 1837); Lane 1946: 173 (synonym of *Calhyboma*Kolbe 1893); Pereira and D’Andretta 1955a: 7 (synonym of *Calhyboma* Kolbe, 1893); Pereira and Martínez 1956a: 121 (cited as synonym of *Calhyboma* Kolbe, 1893., cited as nom. nud.); Martínez 1959: 52 (cited as synonym of *Calhyboma* Kolbe, 1893., cited as nom. nud.); Vulcano and Pereira 1964: 642 (cited as synonym of *Calhyboma* Kolbe, 1893., cited as nom. nud.); Halffter and Edmonds 1982: 139 (cited as subgenus of *Deltochilum* Eschz.); Solís and Kohlmann 2012: 4 (cited as subgenus of *Deltochilum* Eschz).

##### Subgenus Deltochilum (Aganhyboma) Kolbe, 1893

Deltochilum (Aganhyboma) Kolbe, 1893: 192 (original description. Type species by later designation: *Deltochilumtrisignatum* Harold 1881); Shipp 1897: 194 (comment); Paulian 1938: 243 (characters in key), 252 (redescription); Blackwelder 1944: 202 (cited as subgenus of *Deltochilum* Eschz.); Lane 1946: 172 (comment); Pereira and Martínez 1956a: 120 (characters in key); Martínez 1959: 51 (list of species from Argentina); Vulcano and Pereira 1964: 641 (catalog of species); Halffter and Matthews 1966: 261 (distribution, cited as subgenus of *Deltochilum* Eschscholtz, 1822); Vulcano and Pereira 1967: 555 (characters in key); Halffter and Edmonds 1982: 139 (cited as subgenus of *Deltochilum* Eschscholtz, 1822); Vaz-de-Mello et al. 2011: 26 (characters in key); Krajcik 2012: 88 (cited as synonym of *Deltochilum* Eschscholtz, 1822); Silva et al. 2015: 459 (revision); Chamorro et al. 2018: 76 (characters in key), 93 (list of species of Ecuador).

###### Deltochilum (Aganhyboma) arturoi

Taxon classificationAnimaliaColeopteraScarabaeidae

Silva, Louzada & Vaz-de-Mello, 2015

Plate 19A

Deltochilum (Aganhyboma) arturoi Silva, Louzada & Vaz-de-Mello, 2015: 477 (original description. Type locality: Ecuador, Pichincha, BP Milpe, 1200 m).
Deltochilum (Aganhyboma) arturoi : Silva et al. 2018: 5 (figure 5), 8 (characters in key, cited for Ecuador); Chamorro et al. 2018: 93 (cited for Ecuador).

####### Type specimens.

Deltochilum (Aganhyboma) arturoi Silva, Louzada & Vaz-de-Mello, 2015. The holotype (♀) is deposited at the CEMT. Locality: Pichincha: BP Milpe, 1200 m, examined.

**Holotype** (♀): “ECUADOR: PICHINCHA: B.P. / Milpe, 1200m, IV.2003 / Fumigación dosel. PAraujo y / William Chamorro [p, black margin]”.

####### Distribution.

Only known from Ecuador.

####### Records examined.

PICHINCHA: Bosque Potector Milpe-Río Pachijal, 1200 m (1 specimen CEMT). SANTO DOMINGO DE LOS TSÁCHILAS: Puerto Limón, 395 m (1 specimen CEMT).

####### Temporal data.

Collected in April, May, and October.

####### Remarks.

Inhabits coastal lowland evergreen forests and coastal evergreen foothill forests from 395–1200 m a.s.l. Collected with canopy fogging methods and malayse traps baited with pig feces.

###### Deltochilum (Aganhyboma) larseni

Taxon classificationAnimaliaColeopteraScarabaeidae

Silva, Louzada & Vaz-de-Mello, 2015

Plate 19B

Deltochilum (Aganhyboma) larseni Silva, Louzada & Vaz-de-Mello, 2015: 478 (original description. Type locality: Ecuador, SUCUMBIOS [= SUCUMBÍOS], RPF Cuyabeno, Trocha Zábalo-Güepí, km 10).
Deltochilum (Aganhyboma) larseni : Chamorro et al. 2018: 85 (figure 8E), 93 (cited for Ecuador).

####### Type specimens.

Deltochilum (Aganhyboma) larseni Silva, Louzada & Vaz-de-Mello, 2015. The holotype (♂) is deposited at the CEMT. Locality: Ecuador, Sucumbíos: RPF Cuyabeno, Trocha Zábalo-Güepí, km 10, examined.

**Holotype** (♂): “ECUADOR, Sucumbios, R.P.F. / Cuyabeno, Trocha Zábalo-Güepí, / km 10. 9-Agosto-2000 / Colección Manual nocturna / Bosque de tierra firme colinado / Pablo Araujo / LOTE 557 [p,]”.

####### Distribution.

Ecuador and Peru.

####### Records examined.

PASTAZA: Bosque Protector Oglán Alto, 555–605 m (2 specimens CEMT). SUCUMBÍOS: Trocha Zábalo-Guepi km 10, 220 m, Reserva de Producción Faunística Cuyabeno (1 specimen CEMT).

####### Temporal data.

Collected in January and August.

####### Remarks.

Inhabits the lowland evergreen forests and the foothill evergreen forests of the Amazon region from 220–605 m a.s.l. Collected using dead chilopods as bait.

##### Subgenus Deltochilum (Calhyboma) Kolbe, 1893

Deltochilum (Calhyboma) Kolbe, 1893: 191 (original description. Type species: *Deltochilumburmeisteri* Harold, 1867 = *Deltochilummexicanum* Burmeister, 1848, subsequent designation by Paulian, 1939: 18); Shipp 1897: 194 (comment); Paulian 1938: 239 (diagnosis); Blackwelder 1944: 202 (cited as subgenus of *Deltochilum* Eschz.); Lane 1946: 172 (comment); Pereira and D’Andretta 1955a: 8 (redescription); Pereira and Martínez 1956a: 121 (characters in key); Martínez 1959: 52 (list of species from Argentina); Vulcano and Pereira 1964: 642 (catalog of species); Halffter and Matthews 1966: 261 (catalog, distribution, cited as subgenus of *Deltochilum* Eschscholtz, 1822); Vulcano and Pereira 1967: 555 (characters in key); Halffter and Edmonds 1982: 139 (cited as subgenus of *Deltochilum* Eschscholtz, 1822); González et al. 2009: 254 (characters in key, redescription); Vaz-de-Mello et al. 2011: 26 (characters in key); Krajcik 2012: 88 (cited as subgenus of *Deltochilum* Eschscholtz, 1822); Chamorro et al. 2018: 76 (characters in key), 93–94 (list of species of Ecuador).

###### Deltochilum (Calhyboma) arrowi

Taxon classificationAnimaliaColeopteraScarabaeidae

Paulian, 1939 stat. n.

Plates 19C , 57A

Deltochilum (E.) tessellatum var. *Arrowi* Paulian, 1939: 18 (original description. Type locality: Équateur [= Ecuador], Río Pescado).
Deltochilum tessellatum var. arrowi : Blackwelder 1944: 203 (list of species from Latin America); Pereira and D’Andretta 1955a: 43 (redescription); Chamorro et al. 2018: 94 (cited for Ecuador).
Deltochilum var. arrowi : Contreras 1951: 222 (cited for Colombia).

####### Types specimens.

Deltochilum (E.) tessellatum var. *Arrowi* Paulian, 1939. The holotype (♂) and one paratype are deposited at the NHML (see Paulian 1939: 18). Locality: Ecuador Río Pescado. Examined.

**Holotype** (♂): “Ecuador / Río- / Pescado [p]”, “v-17-1922 / 15000 ft / GHTate [p]”, “Frank R. Manson / Collection [p]”, “Ex coll. / F.R.Manson. / Brit. Mus. / 1923-141 [p]”, “Deltochilum / tessellatum / var. arrowi Paulian / Type [hw]”, “Holo- / type [p, red margin]”.

**Paratype** (♀): “Ecuador / Río- / Pescado [p]”, “v-17-1922 / 15000 ft / GHTate [p]”, “Ex coll. / F.R.Manson. / Brit. Mus. / 1923-141 [p]”, “Para- Type [p, yellow margin]”.

####### Distribution.

Colombia and Ecuador.

####### Records examined.

CAÑAR: Javin, 1400 m (4 specimens CEMT). COTOPAXI: San Francisco de las Pampas (1 specimen CEMT). MANABÍ: Río Pescado (2 specimens NHML). PICHINCHA: E B La Hesperia, 1200 m (1 specimen CEMT); Pacto, 1000 m (1 specimen CEMT).

####### Temporal data.

Collected in January, February, March, and October.

####### Remarks.

Inhabits coastal evergreen foothill forests from 850–1200 m a.s.l. Collected using pitfall traps baited with pig feces.

Paulian in 1939 described Deltochilum (E.) tessellatumvar.arrowi (written *Arrowi*, type locality Río Pescado, Ecuador) as a variety of *Deltochilumtessellatum* Bates, 1870 (type locality Gualaquiza, Ecuador, Amazonian region), which made the former name available as a subspecific category (see: ICZN 1999, Article 45.6.4). However, upon examining the external and genital morphology of the type specimens of *D.tessellatumarrowi* (holotype ♂, deposited at the NHML, Plate 57A) and *D.tessellatum* (lectotype ♂ here designated, deposited at the MNHN, Plate 57B) (specifically, the differences observed in the elytral striae and shape of the aedeagus) we determined them to be two distinct species. Therefore, to maintain the subspecific name originally proposed by Paulian (1939), we elevate the variation *arrowi* to species level as follows: Deltochilum (Calhyboma) arrowi Paulian, 1939 stat. n. The holotype is here illustrated (♂ Plate 57A) and originates from the locality Río Pescado, Ecuador (Manabí province, in the coastal region).

###### Deltochilum (Calhyboma) carinatum

Taxon classificationAnimaliaColeopteraScarabaeidae

(Westwood, 1837)

Plate 19D

Hyboma carinata Westwood, 1837: 256 (original description. Type locality: America Meridionali [= South America]).
Hyboma carinata : Burmeister 1848: 135 (redescription).
Hyboma carinatum : Gemminger and Harold 1869: 995 (list of species, distribution); Gillet 1911a: 35 (complete list of species).
Deltochilum (Eudactyles) carinatum : Paulian 1939: 11 (redescription).
Deltochilum carinatum : Pessôa and Lane 1941: 428 (characters in key), 435 (redescription); Medina et al. 2001: 136 (cited for Colombia); Ratcliffe et al. 2015: 195 (cited for Peru); Boilly 2015a: 85 (characters in key); 87 (figure 2), 88 (cited for Guyana).
Deltochilum (Calhyboma) carinatum : Pereira and D’Andretta 1955a: 8 (characters in key), 22 (redescription); Vulcano and Pereira 1964: 643 (catalog of species, distribution); Vulcano and Pereira 1967: 556 (characters in key); Vaz-de-Mello 2000: 192 (cited for Brazil); González et al. 2009: 263 (redescription); 264 (characters in key); Carvajal et al. 2011: 316–317 (cited for Ecuador); Krajcik 2012: 88 (complete list of species); Chamorro et al. 2018: 85 (figure 8D), 93 (cited for Ecuador).
Hyboma monstrosa Dejean, 1837: 151 (nom. nud.); Gemminger and Harold 1869: 995 (cited as synonym of Deltochilumcarinatum Westwood, 1837); González et al. 2009: 263 (cited as synonym of Deltochilum (Calhyboma) carinatum (Westwood, 1837)).
Hyboma dromedarium Castelnau, 1840: 74 (original description); Gemminger and Harold 1869: 995 (cited as synonym of Deltochilumcarinatum Westwood, 1837).

####### Type specimens.

*Hybomacarinata* Westwood, 1837. The holotype is deposited at the OUMNH. Locality: America Meridionali [= South America]), examined.

**Holotype** (sex unknown): “Hyboma / carinata / Westw : / Am: Merid : / Mag: / Zool & Bot /30/ : / Vol / page 256 [hw, red label]”, “Deltochilum / Carinatum West. / J.J.E Gillet. / t.1910-1912. / (type) / [p and hw]”, “TYPE COL : 454 / Hyboma / carinatum / Westw. / HOPE DEPT. OXFORFD [p, black margin]”.

*Hybomamonstrosa* Dejean, 1837. Type material not examined.

*Hybomadromedarium* Castelnau, 1840. One syntype examined deposited at the IRSN. Lectotype to be designated in a future work on this species group.

####### Distribution.

Bolivia, Brazil, Colombia, Ecuador, French Guiana, Guyana, Peru, Surinam, and Venezuela.

####### Records examined.

MORONA SANTIAGO: Nuevo Israel, Cordillera del Kutukú, 1290 m (1 specimen MUTPL); Untsuants sitio 1, Cordillera del Kutukú 700 m (7 specimens MECN; 4 specimens MQCAZ). NAPO: Chalcanapuni (1 specimen CEMT); sector Talac, 730 m, Pungarayacu (1 specimen MQCAZ). ORELLANA: Bosque Daimi Sendero 3 (4 specimens MQCAZ); Bloque 31, Parque Nacional Yasuní, 200 m (5 specimens MECN); Cononaco, Bloque 16 YPF, Parque Nacional Yasuní, 250 m (2 specimens MUTPL); Daimi (6 specimens MQCAZ); Dayuma Campo Hormiguero, plataforma Hormiguero, 320 m (1 specimen MUTPL); Dayuma Campo Palanda, LLumpac, 295 m (1 specimen MGO-UC); Dayuma Campo Palanda, Yuca 13, 255 m (1 specimen MGO-UC); Estación Científica Yasuní, 250 m (37 speciemens MQCAZ); San Sebastián del Coca, 345 m, Comuna Guataraco, Campo Pata (1 specimen MGO-UC); San Sebastian del Coca, 345 m, Comuna Shamanal, Campo Palo Azul (1 specimen MGO-UC). PASTAZA: Bosque Protector Oglán Alto, 590 m (1 specimen MUTPL); Ñemenguno, 280 m (1 specimen MUTPL); Nuevo San José del Curaray, cercanias Río Villano, 245 m (1 specimen MUTPL); Villano, Tarangaro, 330 m (3 specimens MQCAZ). SUCUMBÍOS: Aucayacu Río El Eno 16 km de Lago Agrio, 290 m (2 specimens MGO-UC); Campo Drago Shushufindi (1 specimen MGO-UC); Pacayacu Campo Libertador, 290 m, Tetete (1 specimen MGO-UC); Tarapoa Campo Marian plataforma Fanny 5, 260 m (1 specimen MUTPL); Tarapoa, Nuevo Manabí, 270 m (1 specimen MUTPL). ZAMORA CHINCHIPE: Tundayme campamento Mirador, Las Maravillas, 1060 m (1 specimen MUTPL); Zurmi, Comunidad La Wants, 1010 m (1 specimen MEPN).

####### Temporal data.

Collected every month of the year.

####### Remarks.

Inhabits the lowland evergreen forests, evergreen foothill forests, and lower evergreen montane forests of the Amazon region from 245–1290 m a.s.l. Collected with pitfall traps baited with carrion and human feces.

###### Deltochilum (Calhyboma) hypponum

Taxon classificationAnimaliaColeopteraScarabaeidae

(Buquet, 1844)

Plate 20A

Hyboma hyppona Buquet, 1844: 19 (original description. Type locality: Colombie [= Colombia]).
Deltochilum hypponum : Gemminger and Harold 1869: 996 (list, distribution); Harold 1880a: 17 (cited for Colombia); Heyne and Taschenberg 1907 [= 1908]: 61 (redescription); Gillet 1911a: 36 (complete list of species); Blackwelder 1944: 203 (list of species from Latin America); Contreras 1951: 222 (cited for Colombia); Medina et al. 2001: 136 (cited for Colombia).
Deltochilum hypponum var. arrogans : Gemminger and Harold 1869: 996 (list, distribution); Heyne and Taschenberg 1907 [= 1908]: 61 (cited); Gillet 1911a: 36 (complete list of species); Blackwelder 1944: 203 (list of species of Latin America); Contreras 1951: 222 (cited for Colombia).
Deltochilum (Eudactyles) hypponum : Paulian 1939: 9 (characters in key), 16 (redescription).
Deltochilum (Calhyboma) hypponum : Pereira and D’Andretta 1955a: 8 (characters in key), 26 (redescription); Vulcano and Pereira 1964: 643 (catalog of species, distribution); Vulcano and Pereira 1967: 556 (characters in key); Medina et al. 2003: 65 (distribution); González et al. 2009: 255 (redescription); 264 (characters in key); Carvajal et al. 2011: 316–317 (cited for Ecuador); Krajcik 2012: 88 (complete list of species); Chamorro et al. 2018: 93 (cited for Ecuador).
Hyboma arrogans Buquet, 1844: 20 (original description); Gemminger and Harold 1869: 996 (cited as Deltochilumvar.arrogans Buquet).
Hyboma speciosum Burmeister, 1848: 135 (original description); Gemminger and Harold 1869: 996 (cited as synonym of D.hypponum).

####### Type specimens.

*Hybomahyppona* Buquet, 1844. One syntype examined deposited at the MNHN (ex coll. Guér-Ménev). Lectotype to be designated in a future work on this species group.

*Hybomaarrogans* Buquet, 1844. One syntype examined deposited at the MNHN (ex coll. D. Sharp). Lectotype to be designated in a future work on this species group.

*Hybomaspeciosum* Burmeister, 1848. Type material not examined.

####### Distribution.

Colombia and Ecuador.

####### Records examined.

SUCUMBÍOS: road La Alegría-La Bonita km 32 (1 specimen MECN); La Sofía, 1800 m (1 specimen MUTPL).

####### Temporal data.

Collected in April and May.

####### Remarks.

Inhabits the montane cloud forests of the Andean region at 1800 m a.s.l. Collected manually.

###### Deltochilum (Calhyboma) luederwaldti

Taxon classificationAnimaliaColeopteraScarabaeidae

Pereira & D’Andretta, 1955

Plate 20B

Deltochilum (Calhyboma) luederwaldti Pereira & D’Andretta, 1955a: 29 (original description. Type locality Equador [= Ecuador]).
Deltochilum (Calhyboma) luederwaldti : Vulcano and Pereira 1964: 644 (catalog of species); González et al. 2009: 257 (redescription); 264 (characters in key); Carvajal et al. 2011: 316–317 (cited for Ecuador); Chamorro et al. 2018: 93 (cited for Ecuador).
Deltochilum luederwaldti : Krajcik 2012: 88 (complete list of species).

####### Type specimens.

Deltochilum (Calhyboma) luederwaldti Pereira & D’Andretta, 1955. The holotype (♂) is deposited at the MZUSP (see González and Molano 2009: 257). Locality: Equador, Quito, not examined.

####### Distribution.

Colombia and Ecuador.

####### Literature records.

PICHINCHA: Quito (González et al. 2009: 257).

####### Temporal data.

It is not known when this species was collected.

####### Remarks.

González et al. (2009) cited the distribution of this species as Quito. However, among the scientific collections we examined, we did not find any specimen collected in this locality. The collection method is unknown.

###### Deltochilum (Calhyboma) mexicanum

Taxon classificationAnimaliaColeopteraScarabaeidae

Burmeister, 1848

Plate 20C

Deltochilum mexicanum Burmeister, 1848: 135 (original description. Type locality: Mexico).
Deltochilum mexicanum : Gemminger and Harold 1869: 996 (list of species, distribution); Bates 1887: 37 (cited for Mexico, Costa Rica, Panama); Gillet 1911a: 36 (complete list of species); Blackwelder 1944: 203 (list of species from Latin America); Medina et al. 2001: 136 (cited for Colombia); Ratcliffe 2002: 13 (list of species from Panama); Morón 2003: 30 (redescription); Hamel-Leigue et al. 2006: 14 (list of species from Bolivia); Ratcliffe et al. 2015: 195 (cited for Peru).
Deltochilum (Eudactyles) mexicanum : Paulian 1939: 9 (characters in key), 18 (redescription); Balthasar 1941: 345 (cited for Peru); Islas 1942: 334 (cited for Mexico); Balthasar 1951: 330 (cited for Peru).
Deltochilum (Calhyboma) mexicanum : Pereira and D’Andretta 1955a: 9 (characters in key), 44 (redescription); Vulcano and Pereira 1964: 642 (catalog of species, distribution); Vulcano and Pereira 1967: 556 (characters in key); Howden and Young 1981: 36 (characters in key), 37 (redescription); Medina et al. 2003: 65 (distribution); González et al. 2009: 258 (characters in key), 264 (redescription); Carvajal et al. 2011: 316–317 (cited for Ecuador); Krajcik 2012: 88 (complete list of species); Solís and Kohlmann 2012: 4 (list of species from Costa Rica); Chamorro et al. 2018: 94 (cited for Ecuador).
Deltochilum burmeisteri Harold, 1867d: 76 (original description); Gemminger and Harold 1869: 995 (list, distribution, cited as DeltochilumBurmeisteri); Harold 1880a: 17 (cited for Colombia, cited as D.Burmeisteri); Shipp 1897: 195 (cited as type of Calhyboma Kolbe); Gillet 1911a: 35 (complete list of species, cited as D.Burmeisteri); Paulian 1939: 19 (synonym of Deltochilummexicanum Burmeister); Blackwelder 1944: 202 (list of species for Latin America); Lane 1946: 173 (cited as synonym of D.mexicanum Burmeister); Pereira and D’Andretta 1955a: 44 (cited as synonym of D.mexicanum Burmeister); Pereira and Martínez 1956a: 122 (cited as synonym of D.mexicanum Burmeister); Carvajal et al. 2011: 316–317 (cited for Ecuador); Ratcliffe et al. 2015: 195 (cited for Peru).

####### Type specimens.

*Deltochilummexicanum* Burmeister, 1848. One syntype examined deposited in the MLUH. Lectotype to be designated in a future work on this species group.

*Deltochilumburmeisteri* Harold, 1867. Two syntypes examined deposited at the MNHN. Lectotype to be designated in a future work on this species group.

####### Distribution.

Belize, Bolivia, Colombia, Costa Rica, Ecuador, El Salvador, Guatemala, Honduras, Mexico, Nicaragua, Panama, and Peru.

####### Literature records.

GUAYAS: Bucay (Pereira and D’Andretta 1955a: 44). PICHINCHA: Quito (Paulian 1939: 19).

####### Temporal data.

It is not known when this species was collected.

####### Remarks.

Inhabits coastal evergreen foothill forests. Paulian (1939) cited this species from Quito; however, among the scientific collections we visited, we did not find any specimen collected in this locality. The collection method is unknown.

###### Deltochilum (Calhyboma) robustus

Taxon classificationAnimaliaColeopteraScarabaeidae

Molano & González, 2009

Plate 20D

Deltochilum (Calhyboma) robustus Gonzáles et al. 2009: 259 (original description. Type locality: Colombia, Caquetá. San José del Fragua. Vda. La Esmeralda Alto del Río Yurayaco 1500 m).
Deltochilum robustus : Krajcik 2012: 88 (complete list of species); Ratcliffe et al. 2015: 196 (cited for Peru).
Deltochilum (Calhyboma) robustus : Chamorro et al. 2018: 94 (cited for Ecuador).

####### Type specimens.

Deltochilum (Calhyboma) robustus González & Molano, 2009. The holotype (♂) is deposited at the IAvH (see González et al. 2009: 259). Locality: Colombia, Caquetá. San José del Fragua. Vda. La Esmeralda Alto del Río Yurayaco, 1500 m, not examined.

####### Distribution.

Colombia, Bolivia, Ecuador, and Peru.

####### Records examined.

LOJA: Parque Nacional Podocarpus, 2800 m (3 specimens MQCAZ). NAPO: Quebrada Granadillas Bosque Protector La Cascada, 1300 m, Parque Nacional Sumaco (1 specimen MUTPL); Río Quijos, 1400 m, Parque Nacional Sumaco (1 specimen CEMT). MORONA SANTIAGO: Angel Rouby sitio 8, 1300 m (4 specimens MQCAZ); Nuevo Israel, Cordillera del Kutukú (2 specimens MUTPL); Untsuants sitio 6, 1100 m (1 specimen MUTPL); Unsuants sitio 7, 900 m (3 specimen MQCAZ); Unsuants sitio 4, 1100 m (1 specimen CEMT). PASTAZA: Merá, Estación Biológica Pindo Mirador, 1100 m (1 specimen MUTPL). ZAMORA CHINCHIPE: Tundayme campamento Mirador, La Mina, 1320 m (5 specimens MUTPL); Tundayme campamento Mirador, Condor Mirador, 1460 m (1 specimen MUTPL); RVS El Zarza campamento las Peñas, Cordillera del Cóndor, conseción El Colibri, 1530 m (1 specimen MUTPL); Zurmi Comunidad Miazi, 1380 m (1 specimen MEPN; 1 specimen MUTPL); Zurmi, Pachikuntza, 1685 m (1 specimen MEPN; 2 specimens MUTPL).

####### Temporal data.

Collected in January, March, April, May, September, November, and December.

####### Remarks.

Inhabits the foothill evergreen forests and lower evergreen montane forests in the Amazonian range from 1100–1685 m a.s.l. In the Andean region, it was collected in the montane cloud forests from 1800–2800 m a.s.l. Collected with pitfall traps baited with carrion and human feces and flight interception traps.

###### Deltochilum (Calhyboma) tessellatum

Taxon classificationAnimaliaColeopteraScarabaeidae

Bates, 1870

Plates 21A , 57B

Deltochilum tessellatum Bates, 1870: 175 (original description. Type locality: Gualaquiza, Equador [= Ecuador]).
Deltochilum tessellatum : Gillet 1911a: 36 (complete list of species); Campos 1921: 55 (cited for Ecuador); Blackwelder 1944: 203 (list of species from Latin America); Medina et al. 2001: 136 (cited for Colombia); Krajcik 2012: 88 (complete list of species); Ratcliffe et al. 2015: 196 (cited for Peru).
Deltochilum (Eudactyles) tessellatum : Paulian 1939: 9 (characters in key), 17 (redescription); Balthasar 1941: 345 (cited for Peru); Balthasar 1951: 330 (cited for Peru).
Deltochilum (Calhyboma) tessellatum : Pereira and D’Andretta 1955a: 9 (characters in key), 41 (redescription); Vulcano and Pereira 1964: 645 (catalog of species, distribution); Vulcano and Pereira 1967: 556 (characters in key); González et al. 2009: 260 (redescription); 264 (characters in key); Carvajal et al. 2011: 316–317 (cited for Ecuador); Chamorro et al. 2018: 85 (figure 8F), 94 (cited for Ecuador).

####### Types specimens.

*Deltochilumtessellatum* Bates, 1870. The lectotype (♂) (here designated) and one paralectotype are deposited at the MNHN. Locality: Ecuador. Examined.

**Lectotype** (**here designated**) (♂): “Ecuador / Buckley [hw]”, “Ex Musæo / H.W. BATES / 1892 [p, black margin]”, “Muséum Paris / ex Coll. / R. Oberthür [p, green label, black margin]”, “Deltochilum / tessellatum / Bates – type [hw]”, “LECTOTYPE ♂ / Deltochilum / tesselatum / Bates / des. F.Z. Vaz-de-Mello. 2014 [p and hw, red label, black margin]”.

**Paralectotype** (♀): “near / Cuenca [hw]”, “Ex Musæo / H.W. BATES / 1892 [p, black margin]”, “R. PAULIAN / Vidit [p, black margin]”, “Museum Paris / ex Coll. / R. Oberthur [p, green label, black margin]”, “tessellatum / Bates T. E. S 1870 [hw, black margin]”, “tesellatum Bates / Paulian vd. [hw]”, “PARALECTOTYPE / Deltochilum ♀ / teselatum / Bates / des. F.Z. Vaz-de-Mello, 2014 [hw and p, yellow label, black margin]”.

####### Distribution.

Colombia, Ecuador, and Peru.

####### Records examined.

CARCHI: El Corazón 2100 m (1 specimen MQCAZ). MORONA SANTIAGO: Angel Rouby sitio 9, 2000 m, cordillera del Kutukú (7 specimens MECN); Angel Rouby sitio 10, 1700 m, cordillera del Kutukú (2 specimens MQCAZ); San Antonio, Limón Indaza, Centro Shuar Wuarints (2 specimens MECN). NAPO: Archidona (1 specimen MQCAZ); Pacto Sumaco, Cotundo, 1500 m (1 specimen MUTPL); Quebrada Granadillas, 1300 m, Bosque Protector la Cascada, Parque Nacional Sumaco (1 specimen MUTPL); Río Quijos, 1400 m, Parque Nacional Sumaco (1 specimen MUTPL); Río Hollín (2 specimens MQCAZ). TUNGURAHUA: Baños El Topo, 1590 m (29 specimens CEMT). ZAMORA CHINCHIPE: Chamusquin, 2080 m (1 specimen CEMT; 3 specimens MQCAZ); Cordillera de Curintza, 1790 m, Parque Nacional Poducarpus (12 specimens MECN); La Pituca Cuenca del Río Curitza, 1830 m (1 specimen MUTPL); RVS El Zarza campamento las Peñas, Cordillera del Cóndor, conseción El Zarza, Parcela 5, 1525 m (3 specimens MUTPL); Romerillos senderos Nangaritza, 2200 m (9 specimens MECN); Tundayme campamento Mirador, Condor Mirador, 1460 m (1 specimen MUTPL); Tundayme campamento Mirador, Cara de Indio, 1670 m (1 specimen MUTPL); Zurmi, Pachikuntza, 1685 m (1 specimen MEPN); Zamora km 12–18 (3 specimens MQCAZ).

####### Literature records.

AZUAY: Cuenca (Paulian 1939: 18). CHIMBORAZO: Riobamba (Pereira and Andretta 1955a: 41). MORONA SANTIAGO: Gualaquiza (Paulian 1939: 18); Macas (Paulian 1939: 18). NAPO: Archidona (Pereira and Andretta 1955a: 41); between Archidona and Napo (Paulian 1939: 18). PASTAZA: Sarayacu (Paulian 1939: 18). UNDETERMINED PROVINCE: Santa Inez (Pereira and Andretta 1955a: 41).

####### Temporal data.

Collected in January, February, March, April, May, August, September, October, and November.

####### Remarks.

Inhabits the evergreen montane forests in the Amazonian range from 1300–1700 m a.s.l. In the Andean region, it was registered in the montane cloud forests from 1830–2200 m a.s.l. Collected with flight interception traps and pitfall traps baited with carrion and human feces.

The lectotype (without specific locality for Ecuador) is here designated and illustrated (♂, deposited at the MNHN, Plate 57B).

##### Subgenus Deltochilum (Deltochilum) Eschscholtz, 1822

*Deltochilum* s. str. Eschscholtz, 1822: 37 (original description. Type species: *Deltochilumdentipes* Eschscholtz, 1822); Kolbe 1893: 191 (redescription); Shipp 1897: 194 (comment); Paulian 1938: 243 (characters in key), 268 (redescription); Lane 1946: 172 (comment); Pereira and Martínez 1956a: 120 (characters in key); Halffter and Matthews 1966: 261 (cited as subgenus of *Deltochilum* Eschz.); Vulcano and Pereira 1967: 555 (characters in key); Halffter and Edmonds 1982: 139 (cited as subgenus of *Deltochilum* Eschz.); Vaz-de-Mello 2000: 192 (cited as subgenus of *Deltochilum* Eschz.); González et al. 2009: 254 (characters in key), Vaz-de-Mello et al. 2011: 25 (characters in key); Génier 2012: 26 (redescription, catalog); Boilly and Vaz-de-Mello 2013: 107 (characters in key); Chamorro et al. 2018: 76 (characters in key), 94 (list of species of Ecuador).

*Telhyboma* Kolbe, 1893: 192 (original description. Type species: *Deltochilumorbiculare* Lansberge, 1874); Shipp 1897: 194 (comment); Paulian 1938: 243 (characters in key), 244 (redescription); Blackwelder 1944: 202 (cited as subgenus of *Deltochilum* Eschz.); Lane 1946: 172 (comment); Pereira and Martínez 1956a: 120 (characters in key); Vulcano and Pereira 1964: 647 (catalog of species); Halffter and Matthews 1966: 261 (catalog, distribution, cited as subgenus of *Deltochilum* Eschscholtz, 1822); Halffter and Edmonds 1982: 139 (cited as subgenus of *Deltochilum* Eschscholtz, 1822); González et al. 2009: 254 (characters in key), 271 (redescription); Vaz-de-Mello et al. 2011: 25 (characters in key); Krajcik 2012: 88 (cited as subgenus of *Deltochilum* Eschscholtz, 1822); Solís and Kohlmann 2012: 4 (cited as synonym); Génier 2012: 26 (synonym of *Deltochilum* s. str.).

###### Deltochilum (Deltochilum) orbiculare

Taxon classificationAnimaliaColeopteraScarabaeidae

Lansberge, 1874

Plate 21B

Deltochilum orbiculare Lansberge, 1874a: 6 (original description. Type locality: Bahia).
Deltochilum orbiculare : Kolbe 1893: 194 (distribution); Gillet 1911a: 36 (complete list of species); Pessôa and Lane 1941: 428 (characters in key), 429 (redescription); Blackwelder 1944: 203 (list of species from Latin America); Medina et al. 2001: 136 (cited for Colombia); Hamel-Leigue et al. 2006: 14 (cited for Bolivia); Ratcliffe et al. 2015: 196 (cited for Peru); Boilly 2015a: 85 (characters in key); 87 (figure 7), 88 (cited for Guyana).
Deltochilum (Telhyboma) orbiculare : Paulian 1938: 244 (redescription); Balthasar 1941: 344 (cited for Peru); Pereira and Martínez 1956a: 121 (cited); Vulcano and Pereira 1964: 647 (catalog of species); Vulcano and Pereira 1967: 555 (characters in key); Vaz-de-Mello 2000: 192 (cited for Brazil); Medina et al. 2003: 65 (distribution); González et al. 2009: 271 (redescription); Carvajal et al. 2011: 316–317 (cited for Ecuador).
Deltochilum (Deltochilum) orbiculare : Génier 2012: 31 (distribution); Chamorro et al. 2018: 84 (figure 7G), 94 (cited for Ecuador).

####### Type specimens.

*Deltochilumorbiculare* Lansberge, 1874. One syntype examined deposited at the MNHN (ex coll. V Lansberge). Lectotype to be designated in a future work on this species group.

####### Distribution.

Bolivia, Brazil, Colombia, Ecuador, and Peru.

####### Records examined.

MORONA SANTIAGO: Comunidad Unsuants, 1100 m, Cordillera del Kutukú (5 specimens MQCAZ). NAPO: cerca al Tena, 505 m, Pungarayacu (1 specimen MQCAZ); Estación Científica Jatun Sacha, 450 m (11 specimens MQCAZ). ORELLANA: Dayuma Campo Hormiguero, plataforma Hormiguero, 320 m (1 specimen MUTPL); Dayuma Campo Palanda-Yuca Sur, Estación Palanda 5, 320 m (1 specimen MUTPL); Dayuma Campo Pindo Suyana, 270 m (1 specimen MUTPL); El Dorado plataforma Guarango, 300 m (1 specimen MUTPL); Estación Científica Yasuní PUCE, 250 m (36 specimens MQCAZ); Ines Arango road Tiwino-río Shiripuno, 250 m (1 specimen MUTPL); road to Maxus km 117 Iro, Parque Nacional Yasuní (1 specimen MUTPL); Estación de Biodiversidad Tiputini, 215 m, Parque Nacional Yasuní (4 specimens MUTPL); San Sebastian de Coca, 345 m, Comuna Guataraco, Campo Pata (1 specimen MUTPL); Yampuna (4 specimens MQCAZ). PASTAZA: Bosque Protector Oglán Alto, 660 m (1 specimen MUTPL). SUCUMBÍOS: Aucayacu Río El Eno, 16 km de Lago Agrio, 290 m (1 specimen MUTPL); Quebrada Mansoya, 200 m, Río Putumayo Cuyabeno (1 specimen MUTPL).

####### Literature records.

MORONA SANTIAGO: Untsuants sitio 7, 900 m (Génier 2012: 31).

####### Temporal data.

Collected in January, February, March, April, May, June, July, August, September, November, and December.

####### Remarks.

Inhabits the lowland evergreen forests and evergreen foothill forests of the Amazon region from 200–1100 m a.s.l. Collected with canopy fogging methods and pitfall traps baited with human feces.

###### Deltochilum (Deltochilum) rosamariae

Taxon classificationAnimaliaColeopteraScarabaeidae

Martínez, 1991

Plate 21C

Deltochilum rosamariae Martínez, 1991: 390 (original description. Type locality: Ecuador, provincia Los Ríos, Quevedo, Pichilingue).
Deltochilum rosamariae : Carvajal et al. 2011: 316–317 (cited for Ecuador); Krajcik 2012: 88 (complete list of species).
Deltochilum (Deltochilum) rosamariae : Génier 2012: 32 (comment); Chamorro et al. 2018: 85 (figure 8G), 94 (cited for Ecuador).

####### Type specimens.

*Deltochilumrosamariae* Martínez, 1991. The holotype (♂) is deposited at the AMIC (see Martínez 1991: 392, figure 3) [= name-bearing types now in the MACN]. Locality: Ecuador, provincia Los Ríos, Quevedo, Pichilingue, not examined.

####### Distribution.

Only known from Ecuador.

####### Records examined.

ESMERALDAS: Puerto Balao, 200 m (3 specimens MUTPL). GUAYAS: Cerecita Pta Chapella (3 specimens CEMT); Guayaquil (1 specimen MQCAZ); Guayaquil Los Ceibos (1 specimen CEMT). LOS RÍOS: Quevedo, 45 m, Estación Experimental Pichilingue (1 specimen CEMT); Estación Científica rio Palenque, 200 m (2 specimens MQCAZ). MANABÍ: Ayampe, 35 m (1 specimen MUTPL); Montecristi, Pichihuama, 120 m (2 specimens MUTPL); Puerto López, Guale, 110 m (1 specimen MUTPL); Puerto López, Las Tunas, 100 m (1 specimen MUTPL); Puerto López, Puerto Rico, 115 m (1 specimen MUTPL); Puerto López, Río Blanco, 270 m (1 specimen MUTPL); Reserva Jama Coaque, 15 m (3 specimens MQCAZ). SANTA ELENA: Olón, 50 m (2 specimens CEMT). SANTO DOMINGO DE LOS TSÁCHILAS: Santo Domingo (1 specimen MQCAZ).

####### Temporal data.

Collected in all months except October.

####### Remarks.

Inhabits coastal lowland evergreen forests and coastal lowland semi-deciduous forests from 45–200 m a.s.l. Collected with pitfall traps baited with carrion and human feces.

##### Subgenus Deltochilum (Deltohyboma) Lane, 1946

Deltochilum (Deltohyboma) Lane, 1946: 175 (cited as subgen. n. Type species: *Deltochilumsubmetallicum* (Castelnau, 1840), cited as ortotipo); Pereira and Martínez 1956a: 121 (characters in key); Martínez 1959: 53 (cited as subgenus of *Deltochilum* Eschz.); Vulcano and Pereira 1964: 652 (cited as subgenus of *Deltochilum* Eschz.); Halffter and Matthews 1966: 261 (cited as subgenus of *Deltochilum* Eschz.); Vulcano and Pereira 1967: 555 (characters in key); Vaz-de-Mello 2000: 192 (cited as subgenus of *Deltochilum* Eschz); Vaz-de-Mello et al. 2011: 26 (characters in key); Krajcik 2012: 88 (complete list of species, cited as subgenus of *Deltochilum* Eschz); Boilly and Vaz-de-Mello 2013: 107 (characters in key); Chamorro et al. 2018: 76 (characters in key), 94 (list of species of Ecuador).

###### Deltochilum (Deltohyboma) aequinoctiale

Taxon classificationAnimaliaColeopteraScarabaeidae

(Buquet, 1844)

Plate 21D

Hyboma aequinoctialis Buquet, 1844: 21 (original description. Type locality: Colombie [= Colombia]).
Deltochilum aequinoctiale : Burmeister 1848: 135 (redescription); Gemminger and Harold 1869: 995 (list, distribution); Gillet 1911a: 35 (complete list of species); Balthasar 1941: 344 (cited for Peru); Blackwelder 1944: 202 (list of species from Latin America); Balthasar 1951: 329 (cited for Peru); Contreras 1951: 221 (cited for Colombia, cited as D.aequinoctiale); Medina et al. 2001: 136 (cited for Colombia); Medina et al. 2003: 65 (distribution); Krajcik 2012: 88 (complete list of species); Ratcliffe et al. 2015: 195 (cited for Peru).
Deltochilum (Deltohyboma) aequinoctiale : Paulian 1938: 270 (characters in key), 278 (redescription); Vulcano and Pereira 1964: 652 (catalog of species, distribution); Vulcano and Pereira 1967: 557 (characters in key); Carvajal et al. 2011: 316–317 (cited for Ecuador); Chamorro et al. 2018: 85 (figure 8H), 86 (figure 9A), 94 (cited for Ecuador).
Deltochilum erodioides Harold, 1867d: 77 (original description); Gemminger and Harold 1869: 995 (list, distribution); Harold 1880a: 18 (cited for Colombia); Kirsch 1885: 212 (cited for Ecuador); Gillet 1911a: 36 (complete list of species); Paulian 1938: 278 (synonym of D. (D.) aequinoctiale Buq.); Blackwelder 1944: 202 (list of species of Latin America); Contreras 1951: 222 (cited for Colombia); Vulcano and Pereira 1964: 652 (cited as synonym of Deltochilumaequinotiale Buquet); Carvajal et al. 2011: 316–317 (cited for Ecuador); Ratcliffe et al. 2015: 195 (cited for Peru).

####### Type specimens.

*Hybomaaequinotialis* Buquet, 1844. One syntype examined deposited at the MNHN (ex coll. D Sharp). Lectotype to be designated in a future work on this species group.

*Deltochilumerodioides* Harold, 1867. Two syntypes examined deposited at the MNHN (ex coll. E Steinheil, ex coll. E Harold, ex coll. R Oberthur). Lectotype to be designated in a future work on this species group.

####### Distribution.

Colombia, Ecuador, and Peru.

####### Records examined.

COTOPAXI: Bosque Integral Otonga, 2080 m (78 specimens CEMT: 7 specimens MQCAZ; 4 specimens MUTPL). PICHINCHA: Chespi, Bellavista, 1380 m (1 specimen MUTPL); Yunguilla, Loma La Liberia, 2400 m (2 specimens MUTPL); Reserva Orquideológica El Pahuma, 1975 m (1 specimen MUTPL).

####### Literature records.

BOLIVAR: au dessus de Chimbo, 1900 m (Paulian 1938: 280). MORONA SANTIAGO: Macas (Paulian 1938: 280).

####### Temporal data.

Collected in January, February, March, April, June, July, September, and December.

####### Remarks.

Inhabits the evergreen lower montane forests and montane cloud forests of the Andean region from 1300–2400 m a.s.l. Collected with pitfall traps baited with carrion and human feces. It is possible that the records cited by Paulian (1938) from Macas, Morona Santiago province, in the Ecuadorian Amazon, is a different species belonging to the same subgenus. A review of these species is needed.

###### Deltochilum (Deltohyboma) barbipes

Taxon classificationAnimaliaColeopteraScarabaeidae

Bates, 1870

Plate 22A

Deltochilum barbipes Bates, 1870: 177 (original description. Type locality: Upper Amazons).
Deltochilum barbipes : Gillet 1911a: 35 (complete list of species); Paulian 1938: 271 (characters in key); Paulian 1939: 4 (redescription); Blackwelder 1944: 202 (list of species from Latin America).
Deltochilum (Deltohyboma) barbipes : Vulcano and Pereira 1964: 653 (catalog of species, distribution); Vulcano and Pereira 1967: 560 (characters in key); Vaz-de-Mello, 2000: 192 (cited for Brazil); Carvajal et al. 2011: 316–317 (cited for Ecuador); Krajcik 2012: 88 (complete list of species); Chamorro et al. 2018: 94 (cited for Ecuador).

####### Type specimens.

*Deltochilumbarbipes* Bates, 1870. Two syntypes examined deposited at the MNHN (ex coll. HW Bates and ex coll. R Oberhür). Lectotype to be designated in a future work on this species group.

####### Distribution.

Colombia, Bolivia, Brazil, Ecuador, and Venezuela.

####### Records examined.

MORONA SANTIAGO: Nuevo Israel, Cordillera del Kutukú, 1290 m (1 specimen MUTPL). PASTAZA: Bosque Protector Oglán, 600 m (1 specimen CEMT; 2 specimens MUTPL).

####### Temporal data.

Collected in January, June, and December.

####### Remarks.

Inhabits the foothill evergreen forests of the Amazon region from 600–1290 m a.s.l. Collected with pitfall traps baited with carrion and/or dead chilopods.

###### Deltochilum (Deltohyboma) batesi

Taxon classificationAnimaliaColeopteraScarabaeidae

Paulian, 1938

Plate 22B

Deltochilum (D.) batesi Paulian, 1938: 286 (original description. Type locality: Équateur [= Ecuador], Sarayacu, Macas, Loja).
Deltochilum (Deltohyboma) batesi : Vulcano and Pereira 1964: 653 (catalog of species, distribution); Vulcano and Pereira 1967: 557 (characters in key); Carvajal et al. 2011: 316–317 (cited for Ecuador); Chamorro et al. 2018: 94 (cited for Ecuador).
Deltochilum batesi : Blackwelder 1944: 202 (list of species from Latin America); Krajcik 2012: 88 (complete list of species).

####### Type specimens.

Deltochilum (Deltochilum) batesi Paulian, 1938. Three syntypes are deposited at the MNHM (ex coll. D. Sharp). Lectotype to be designated in a future work on this species group.

**Syntype** (♂): “Sarayacu Ecuador / Bucley 1879 [hw]”, “Ex-Musæo / D. Sharp 1890 [p, black margin]”, “R. Paulian / Vidit [p, black margin]”, “MUSÉUM PARIS / 1952 / coll. R. OBERTHÜR [p]”, “-TYPE- / DELTOCHILUM / BATESI / PAULIAN / Dét. F. Génier, 1998 [p and hw, black margin]”, “SYNTYPE [p, red label]”, “MNHN / EC2493 [p, black margin]”.

**Syntype** (♀): “Equateur / Loja / Abbe Gaujon [p, black margin]”, “D. Batesi Paul. / R. Paulian det. [p and hw]”, “MUSÉUM PARIS / 1952 / coll. R. OBERTHÜR [p]”, “SYNTYPE [p, red label]”, “MNHN / EC2497 [p, black margin]”.

**Syntype** (♂): “Sarayacu Ecuador / Bucley 1879 [hw]”, “Ex-Musæo / D. Sharp 1890 [p, black margin]”, “Museun Paris / ex Coll. / R. Oberthur [p, green label]”.

####### Distribution.

Only known from Ecuador.

####### Records examined.

ORELLANA: Estación Científica Yasuní, 250 m (1 specimen CEMT); Río Tiputini Yasuní Res. (1 specimen CEMT); Rodrigo Borja IAMOE (5 specimens CEMT); Yampuna (1 specimen CEMT). PASTAZA: Sarayacu (2 specimens MNHN). LOJA: without specific locality (1 specimen MNHN).

####### Literature records.

MORONA SANTIAGO: Macas (Paulian 1938: 286).

####### Temporal data.

Collected in January and September.

####### Remarks.

Inhabits the lowland evergreen forests of the Amazon region at 250 m a.s.l. Collected with flight interception traps and pitfall traps baited with carrion.

###### Deltochilum (Deltohyboma) crenulipes

Taxon classificationAnimaliaColeopteraScarabaeidae

Paulian, 1938

Plate 22C

Deltochilum (D.) crenulipes Paulian, 1938: 286 (original description. Type locality: Pérou [= Peru], Poazu-Pozuzo, Chanchamayo, Amazones Yurimaguas).
Deltochilum crenulipes : Balthasar 1941: 345 (cited for Peru); Blackwelder 1944: 202 (list of species from Latin America); Balthasar 1951: 330 (cited for Peru); Krajcik 2012: 88 (complete list of species); Ratcliffe et al. 2015: 195 (cited for Peru).
Deltochilum (Deltohyboma) crenulipes : Vulcano and Pereira 1964: 653 (catalog of species, distribution); Vulcano and Pereira 1967: 558 (characters in key); Vaz-de-Mello 2000: 192 (cited for Brazil); Génier 2001: 5 (comment); Carvajal et al. 2011: 316–317 (cited for Ecuador); Bezdek and Hajek 2011: 368 (catalog of the types of the NMPC); Chamorro et al. 2018: 94 (cited for Ecuador).
Deltochilum (s. str.) obenbergeri Balthasar, 1939f: 13 (original description); Blackwelder 1944: 203 (list of species for Latin America); Vulcano and Pereira 1964: 657 (catalog of species, distribution); Vulcano and Pereira 1967: 558 (characters in key); Medina et al. 2001: 136 (cited for Colombia); Génier 2001: 5 (synonym of Deltochilumcrenulipes Paulian, 1938); Carvajal et al. 2011: 316–317 (cited for Ecuador); Bezdek and Hajek 2011: 367 (catalog of the types of the NMPC).

####### Type specimens.

*Deltochilumcrenulipes* Paulian, 1938. The lectotype is deposited at the MNHN (ex coll. R Oberthür). Locality: Amazones Yurimaguas, examined.

**Lectotype** (sex unknown): “Amazones / Yurimaguas [p, black margin]”, “R. PAULIAN / Vidit [p, black margin]”, “D. crenulipes n. sp / Type [hw]”, “MUSÉUM PARIS / 1952 / coll. R. OBERTHÜR [p, black margin]”, “LECTOTYPE [p, red label]”, “MNHN / EC2496 [p, black margin]”, “DELTOCHILUM / CRENULIPES / PAULIAN / LECTOTYPE / Dés. F. Génier, 2000 [p and hw, black margin]”.

Deltochilum(s. str.)obenbergeri Balthasar, 1939. The holotype is deposited at the NMPC. Locality: Mera Ecuador, examined.

**Holotype** (sex unknown): “Mera / Ecuador [p]”, “TYPUS [p, red label]”, “HOLOTYPE [p, red label]”, “1324 / Dok. L. Mencl, 2011 [p, green label]”, “Obenbergeri / m. [hw, green label, black margin]”, “DELTOCHILUM / OBENBERGERI / BALTHASAR / HOLOTYPE [hw] / Det. F. Génier [p] 2000 [hw]”.

####### Distribution.

Brazil, Colombia, Ecuador, and Peru.

####### Records examined.

MORONA SANTIAGO: Angel Rouby, Codillera del Kutuku, 1300 m (12 specimens MECN); Untsuants sitio 3, Cordillera del Kutuku (7 specimens MQCAZ). NAPO: Archidona (11 specimens MQCAZ); Río Hollín, 1100 m (1 specimen CEMT); Bloque 20, Pungarayacu, 610 m (1 specimen MQCAZ); Santo Domingo de Hollín, Río Hollin, 635 m (3 specimens MQCAZ); Cotundo, La Merced de Jondachi Río Jondachi, 1100 m (1 specimen MGO-UC); Misahualli Jungle Lodge unión río Napo y río Misahualli, 1900 m (2 specimens MQCAZ); Río Osayacu, 1070 m (1 specimen MUTPL); Sunka, 300 m (5 specimens MQCAZ). ORELLANA: Bloque 31, Parque Nacional Yasuní, 200 m (9 specimens MECN); Daimi (1 specimen CEMT); Dayuma Campo Palanda, Llumpac, 295 m (3 specimens MGO-UC); Dayuma plataforma Ungurahua, 300 m (1 specimen MUTPL); El Dorado plataforma Guarango, 300 m (1 specimen MUTPL); Rodrigo Borja IAMOE (33 specimens CEMT; 11 specimens MQCAZ); Estación Científica Yasuní PUCE 215 m (2 specimen CEMT; 84 specimens MQCAZ); Estación de Biodiversidad Tiputini USFQ (4 specimens MGO-UC; 1 specimen MUTPL); Lago San Pedro, plataforma Copal, 310 m (1 specimen MUTPL); Río Tiputini Yasuní Res. Stn. (2 specimens CEMT); San Sebastián del Coca, Comuna Guataraco, Campo Pata (1 specimen MGO-UC); Yuturi Lodge Río Napo, 270 m (3 specimens MQCAZ). PASTAZA: Bosque Protector Oglan Alto, 540 m (1 specimen MGO-UC); Campo Tiguino, cerca al estero Ñemenguno, 300 m (1 specimen MUTPL); Chuyayacu Oleoducto km 25, 200 m (1 specimen MGO-UC); E. B. Pindo Mirador UTE, 1000 m (1 specimen MUTPL); Mera (1 specimen NMPC); road El Triunfo-Arajuno (1 specimen CEMT); Villano Pandanuque, 420 m (1 specimen MUTPL); SUCUMBÍOS: 6 km de Dureno, Precooperativa Los Vergeles, 290 m (7 specimens MGO-UC); Aucayacu Río El Eno, 16 km de Lago Agrio, 275 m (2 specimens MGO-UC); Bermejo plataforma ER-A road to a Lumbaqui (1 specimen MUTPL); La Selva Bio. Station 175 km ESE del Coca (4 specimens MQCAZ); Nueva Loja, plataforma Iguana, 310 m (1 specimen MQCAZ); Pichira, Limoncocha (1 specimen MQCAZ); Sacha Lodge, 270 m (4 specimens MQCAZ); Tarapoa, Nuevo Manabí, 270 m (1 specimen MUTPL). ZAMORA CHINCHIPE: Tundayme, Cara de Indio, 1670 m (1 specimen MUTPL); Tundayme Ecsa, road to Polvorín, 1300 m (1 specimen MUTPL); Tundayme Ecsa vivero, 820 m (1 specimen MUTPL); Zurmi Las Orquideas Río Nangaritza, 870 m (1 specimen MUTPL).

####### Temporal data.

Collected every month of the year.

####### Remarks.

Inhabits the lowland evergreen forests and evergreen foothill forests of the Amazon region from 200–1680 m a.s.l. Collected with flight interception traps and pitfall traps baited with carrion and human feces.

###### Deltochilum (Deltohyboma) peruanum

Taxon classificationAnimaliaColeopteraScarabaeidae

Paulian, 1938

Plate 22D

Deltochilum (Deltochilum) peruanum Paulian, 1939: 2 (original description. Type locality: Pérou [= Peru], Pozuzo-Chanchamayo, Amazones).
Deltochilum (s. str.) peruanum : Paulian 1938: 271 (characters in the key); Balthasar 1941: 345 (cited for Peru); Balthasar 1951: 330 (cited for Peru).
Deltochilum peruanum : Blackwelder 1944: 203 (list of species from Latin America); Krajcik 2012: 88 (complete list of species); Ratcliffe et al. 2015: 196 (cited for Peru).
Deltochilum (Deltohyboma) peruanum : Vulcano and Pereira 1964: 657 (catalog of species, distribution); Vulcano and Pereira 1967: 559 (characters in key); Génier 2001: 2 (comment); Carvajal et al. 2011: 316 (cited for Ecuador); Bezdek and Hajek 2011: 367 (catalog of type NMPC); Chamorro et al. 2018: 94 (cited for Ecuador).
Deltochilum (s. str.) laevigatum Balthasar, 1939f: 7 (original description); Balthasar 1941: 345 (cited for Peru); Balthasar 1951: 330 (cited for Peru); Bezdek and Hajek 2011: 367 (catalog of the types of the NMPC).
Deltochilum (Deltohyboma) laevigatum : Vulcano and Pereira 1964: 656 (catalog of species, distribution); Vulcano and Pereira 1967: 558 (characters in key).
Deltochilum laevigatum : Blackwelder 1944: 203 (list of species from Latin America); Génier 2001: 2 (synonym of Deltochilumperuanum Paulian, 1939).

####### Type specimens.

Deltochilum (Deltochilum) peruanum Paulian, 1938. The lectotype (sex unknown) is deposited at the MNHN. Locality: Chanchamayo Peru, examined.

**Lectotype** (sex unknown): “Chanchamayo [hw]”, “Ex-Musæo / D. Sharp 1890 [p, black margin]”, “R. Paulian / Vidit [p, black margin]”, “D. peruanum n. sp / Type [hw]”, “MUSÉUM PARIS / 1952 / coll. R. Oberthur [p]”, “MNHN / EC2492 [p, black margin]”, “LECTOTYPE [p, red label]”, “DELTOCHILUM / PERUANUM / PAULIAN / LECTOTYPE / Dés. F. Génier, 2000 [p and hw, black margin]”.

Deltochilum(s. str.)laevigatum Balthasar, 1939. The lectotype (♂) and one paralectotype are deposited at the NMPC. Locality: Peru Chanchamayo, examined.

**Lectotype** (♂): “O. PERU / Chanchamayo / 1000 m [p]”, “Kolbe determ. / Deltochilum / laevigatum / cotype Kolbe [p and hw]”, “Typus [p, red label, black margin]”, “laevigatum / m. [hw, green label, black margin]”, “1321 / Dok. L Menci, 2001 [p, green label]”, “Senckenberg / Museum [p]”, “LECTOTYPE [p, red label black margin]”, “DELTOCHILUM ♂ / LAEVIGATUM / BALTHASAR / LECTOTYPE [hw] / Des. F. Génier 2000 [p and hw]”.

**Paralectotype** (♀): “Mera / Ecuador [p]”, “Typus [p, red label, black margin]”, “PARALECTOTYPE [p, yellow label]”, “DELTOCHILUM ♀ / LAEVIGATUM / BALTHASAR / Det. F. Génier, 2000 [p and hw]’.

####### Distribution.

Ecuador and Peru.

####### Records examined.

PASTAZA: Mera (1 specimen NMPC).

####### Literature records.

MORONA SANTIAGO: Macas (Balthasar 1939f: 8; Paulian 1939: 3).

####### Temporal data.

It is not known when this species was collected.

####### Remarks.

Inhabits the foothill evergreen forests in the Amazon. The collection method is unknown.

###### Deltochilum (Deltohyboma) speciosissimum

Taxon classificationAnimaliaColeopteraScarabaeidae

Balthasar, 1939

Plate 23A

Deltochilum (s. str.) speciosissimum Balthasar, 1939f: 16 (original description. Type locality: Ecuador).
Deltochilum speciosissimum : Blackwelder 1944: 203 (list of species from Latin America); Krajcik, 2012: 88 (complete list of species).
Deltochilum (Deltohyboma) speciosissimum : Lane 1847: 110 (synonym of Deltochilum(s. str.)speciosissimum Balthasar, 1939); Vulcano and Pereira 1964: 659 (catalog of species, distribution); Vulcano and Pereira 1967: 557 (characters in key); Génier 2001: 7 (comment); Carvajal et al. 2011: 316–317 (cited for Ecuador); Bezdek and Hajek 2011: 368 (catalog of the types of the NMPC); Chamorro et al. 2018: 94 (cited for Ecuador).

####### Type specimens.

Deltochilum(s. str.)speciosissimum Balthasar, 1939. The lectotype (♂) and one paralectotype are deposited at the NMPC. Locality: Ecuador Canelos, examined.

**Lectotype** (♂): “ECUADOR / Canelos / F. Ohaus S. [p]”, “Kolbe determ. / Deltochilum / pretiosum / ? ♂ Har. [p and hw]”, “LECTOTYPE [p, red label]”, “Speciosissimun / m [hw, green label, black margin]”, “1323 / Dok L. Mencl, 2001 [p, green label]”, “DELTOCHILUM / SPECIOSISSIMUM / BALTHASAR / LECTOTYPE / Dés F. Génier 2000 [p and hw]”.

**Paralectotype** (♂): “ECUADOR / Sabanilla / F. Ohaus S. / 20. 9. 05 [p and hw]”, “Typus [p, red label]”, “PARALECTOTYPE [p, yellow label]”, “DELTOCHILUM / SPECIOSISSIMUM / BALTHASAR / PARALECTOTYPE / Dés. F. Génier 2000 [p and hw]’.

####### Distribution.

Only known from Ecuador.

####### Records examined.

PASTAZA: Canelos (1 specimen NMPC). ZAMORA CHINCHIPE: Sabanilla [= El Tambo] (1 specimen NMPC).

####### Temporal data.

Collected in September and December.

####### Remarks.

This species may be distributed in the foothill evergreen forests as well as on the low-montane evergreen forests of the Amazon region. The collection method is unknown.

##### Subgenus Deltochilum (Hybomidium) Shipp, 1897

Deltochilum (Hybomidium) Shipp, 1897: 195 (cited as nom. n. Type species: *Deltochilumgibbosum* (Fabricius 1775) according to Lane, 1846); Gillet 1911a: 35 (cited as synonym of *Deltochilum* Eschscholtz, 1822); Lucas 1920: 339 (synonym of *Deltochilum* Eschz.); Paulian 1938: 237 (cited as synonym of *Deltochilum* Eschscholtz, 1822); Pessôa and Lane 1941: 426 (cited as synonym of *Deltochilum* Eschscholtz, 1822); Blackwelder 1944: 202 (cited as synonym of *Deltochilum* Esch); Lane 1946: 172 (comment); Pereira and Martínez 1956a: 121 (characters in key); Martínez 1959: 55 (list of species from Argentina); Vulcano and Pereira 1964: 647 (catalog of species); Halffter and Matthews 1966: 261 (catalog, distribution, cited as subgenus of *Deltochilum* Eschscholtz, 1822); Vulcano and Pereira 1967: 555 (characters in key); Vaz-de-Mello 2000: 192 (list of species from Brazil, cited as subgenus of *Deltochilum* Eschscholtz, 1822); Ratcliffe 2002: 13 (cited as synonym of *Deltochilum* Eschscholtz, 1822); González et al. 2009: 254 (characters in key), 265 (redescription); Vaz-de-Mello et al. 2011: 26 (characters in key); Krajcik 2012: 88 (cited as subgenus of *Deltochilum* Eschscholtz, 1822); Boilly and Vaz-de-Mello 2013: 107 (characters in key); González-Alvarado and Vaz-de-Mello 2014: 432: (revision); Chamorro et al. 2018: 76 (characters in key), 94 (list of species of Ecuador).

*Hyboma* Audinet-Serville, 1825: 352 (original description. Type species: *Ateuchusgibbosus* Fabricius, 1801); Dejean 1837: 151 (catalog, distribution); Castelnau 1840: 73 (redescription); Reiche 1841: 212 (characters in key); Sturm 1843: 103 (catalog, distribution); Gemminger and Harold 1869: 995 (cited as synonym); Shipp 1897: 195 (synonym of *Hybomidium* Shipp); Gillet 1911a: 35 (cited as synonym); Lucas 1920: 339 (catalog, distribution); Paulian 1938: 237 (cited as synonym); Pessôa and Lane 1941: 426 (cited as synonym); Blackwelder 1944: 202 (cited as synonym); Lane 1946: 172 (comment); Pereira and Martínez 1956a: 120 (cited as synonym); Martínez 1959: 50 (cited as synonym); Vulcano and Pereira 1964: 639 (cited as synonym); Ratcliffe 2002: 13 (cited as synonym); Solís and Kohlmann 2012: 4 (cited as synonym); González et al. 2009: 432 (cited as synonym of Deltochilum (Hybomidium) Shipp, 1897).

*Tetradontides* Paulian, 1938: 259 (original description. Type species: *Deltochilumgibbosum*Fabricius 1775, cited as *Deltochilumgibbosum* (F.), 1775); Lane, 1946: 173 (comment), 175 (synonym of *Hybomidium* Shipp, 1897); Pereira and Martínez 1956a: 125 (cited as synonym of *Hybomidium* Shipp, 1897); Martínez, 1959: 55 (cited as synonym of *Hybomidium* Shipp, 1897); Vulcano and Pereira 1964: 647 (cited as synonym of *Hybomidium* Shipp, 1897); Halffter and Edmonds 1982: 139 (cited as subgenus of *Deltochilum* Eschscholtz, 1822).

###### Deltochilum (Hybomidium) loperae

Taxon classificationAnimaliaColeopteraScarabaeidae

González & Molano, 2009

Plate 23B

Deltochilum (Hybomidium) loperae González et al. 2009: 268 (original description. Type locality: Colombia. Valle del Cauca. Estación forestal Bajo Calima. 50 m.).
Deltochilum loperae : Krajcik 2012: 88 (complete list of species).
Deltochilum (Hybomidium) loperae : González-Alvarado and Vaz-de-Mello 2014: 454 (redescription), 472 (characters in key); Chamorro et al. 2018: 94 (cited for Ecuador).

####### Type specimens.

Deltochilum (Hybomidium) loperae González & Molano, 2009. The holotype (♂) is deposited at the CECC (see González et al. 2009: 268). Locality: Colombia. Valle del Cauca. Estación forestal Bajo Calima. 50 m, not examined.

####### Distribution.

Colombia and Ecuador.

####### Records examined.

CARCHI: Tobar Donoso, 300 m (2 specimens MECN). COTOPAXI: km 4 Guasaganda, 500 m (1 specimen CEMT). ESMERALDAS: Playa de Oro, Estero Pote, 200 m (4 specimens CEMT); Playa de Oro, La Tabla (1 specimen MECN); Tsejpi (1 specimen MECN); Tsejpi, Rio Zapallo (1 specimen MECN).

####### Temporal data.

Collected in March, April, February, October, November, and December.

####### Remarks.

Inhabits coastal lowland evergreen forests from 200–500 m a.s.l. Collected with pitfall traps baited with carrion and human feces.

###### Deltochilum (Hybomidium) orbignyiamazonicum

Taxon classificationAnimaliaColeopteraScarabaeidae

Bates, 1887

Plate 23C

Deltochilum amazonicum Bates, 1887: 37 (original description. Type locality: Amazons, Ega [= Tefé], Pebas).
Deltochilum amazonicum : Gillet 1911a: 35 (complete list of species); Boucomont 1928c: 3 (distribution); Blackwelder 1944: 202 (list of species from Latin America); Medina et al. 2001: 136 (cited for Colombia); Hamel-Leigue et al. 2006: 14 (list of species from Bolivia); Krajcik 2012: 88 (complete list of species); Ratcliffe et al. 2015: 195 (cited for Peru).
Deltochilum gibbosum Subsp. amazonicum : Kolbe 1905: 534 (distribution).
Deltochilum (Tetraodontides) amazonicum : Paulian 1938: 262 (redescription).
Deltochilum (Hybomidium) amazonicum : Vulcano and Pereira 1964: 647 (catalog of species); Vulcano and Pereira 1967: 560 (characters in key); Vaz-de-Mello 2000: 192 (cited for Brazil); González et al. 2009: 265 (redescription), 270 (characters in key).
Deltochilum (Hybomidium) orbignyiamazonicum : González-Alvarado and Vaz-de-Mello 2014: 450 (cited as new status, distribution), 472 (characters in key); Chamorro et al. 2018: 86 (figure 9B, C), 94 (cited for Ecuador).

####### Type specimens.

*Deltochilumamazonicum* Bates, 1887. The lectotype and four paralectotypes are deposited at the MNHN (ex coll. HW Bates, ex coll. R Oberthur) (see González-Alvarado and Vaz-de-Mello 2014: 450). Locality: Pebas Amazonas, examined.

LECTOTYPE, (♂): “Pebas / Amaz. [hw]”, “Deltochilum / amazonicum / Bates ♂ major [hw]”, “Ex Musæo / H.W. BATES / 1892 [p, black margin]”, “R. PAULIAN / Vidit [p, black margin]”, “LECTOTYPE [p, red label]”, “Museum Paris / ex Coll. / R. Oberthur [p, green label, black margin]”, “LECTOTYPE ♂ / Deltochilum / amazonicum / Bates / des. F.Z. Vaz-de-Mello. 2014 [p and hw, red label, black margin]”.

####### Distribution.

Brazil, Colombia, Ecuador, and Peru.

####### Records examined.

MORONA SANTIAGO: Bosque Domoso, 1650 (1 specimen CEMT; 6 specimens MQCAZ); Nuevo Israel, Cordillera del Kutukú, 1290 m (1 specimen MGO-UC); Untsuants, sitio 7, 900 m, Cordillera del Kutukú (8 specimens MQCAZ); road Mendez-Paute km 8 (1 specimen CEMT; 7 specimens MQCAZ). NAPO: cerca al Tena, 505 m, Pungarayacu (1 specimen MQCAZ); Estación Jatun Sacha, 450 m (19 specimens MQCAZ); Puerto Misahualli Jungle (6 specimens MQCAZ); Tena (4 specimens CEMT; 7 specimens MQCAZ). ORELLANA: Bloque 31, Parque Nacional Yasuní, 200 m (3 specimens MQCAZ); Cononaco, Bloque 16 YPF Parque Nacional Yasuní, 250 m (1 specimen MUTPL); Daimi, Pozo Daimi (5 specimens MQCAZ); Dayuma Campo Hormiguero, plataforma Hormiguero, 320 m (1 specimen MUTPL); Dayuma Campo Palanda-Yuca Sur, plataforma Yuca 13, 255 m (1 specimen MUTPL); Dayuma plataforma Ungurahua, 300 m (1 specimen MUTPL); El Dorado plataforma Guarango, 300 m (1 specimen MUTPL); Estación de Biodiversidad Tiputini USFQ, Río Tiputini, 270 m (1 specimen MGO-UC); Estación Científica Yasuní PUCE, 250 m (58 specimens MQCAZ); Lago San Pedro, plataforma Copal, 310 m (1 specimen MUTPL); Rodrigo Borja IAMOE (1 specimen CEMT; 9 specimens MQCAZ); San Sebastian del Coca, Comuna Guataraco, 345 m, Campo Pata (2 specimen MGO-UC); San Sebastian del Coca, Comuna Shamanal, 345 m, Campo Palo Azul (1 specimen MUTPL); Yampuna (2 specimens MQCAZ). PASTAZA: Bosque Protector Oglán Alto, 510 m (1 specimen MUTPL); Nuevo San José del Curaray, cercanias Río Villano, 245 m (1 specimen MGO-UC); San Virgilio (2 specimen MGO-UC). SUCUMBÍOS: 6 km de Dureno, 290 m, Precooperativa Los Vergeles (1 specimen MGO-UC); Nueva Loja plataforma Iguana, 310 m (1 specimen MUTPL); Pacayacu Campo Libertador, 260 m (1 specimen MUTPL); Shushufindi Campo Drago, 295 m (1 specimen MGO-UC); Tarapoa Campo Marian, plataforma Fanny 5, 260 m (1 specimen MUTPL); Tarapoa, Nuevo Manabí, 270 m (1 specimen MUTPL). TUNGURAHUA: Baños, El Topo, 1590 m (1 specimen MUTPL). ZAMORA CHINCHIPE: Tundayme, campamento Mirador, Valle del Quimi, 1000 m (1 specimen MUTPL); road Mendes-Paute km 8, 1250 m (4 specimens MQCAZ); road Zumbi-Yantzaza km 4, 900 m (1 specimen CEMT; 6 specimens MQCAZ); road Cumbaritza-Gualaquiza km 1, 1100 m (4 specimens MQCAZ).

####### Literature records.

FCO. DE ORELLANA [= ORELLANA]: Rodrigo Borja, IAMOE (González and Vaz-de-Mello 2014: 451). MORONA SANTIAGO: Bosque Domono [= Bosque Domoso], 1650 m (González and Vaz-de-Mello 2014: 451); Via Mendez-Paute km 8, 1250 m (González and Vaz-de-Mello 2014: 451). NAPO: Tena (González and Vaz-de-Mello 2014: 451); Jatun, Sacha Biol. Station, 21 km E Puerto Napo, 400 m (González and Vaz-de-Mello 2014: 451); 3.3 km E Puerto Napo, 400 m (González and Vaz-de-Mello 2014: 451). PASTAZA: 9 km ESE Veracruz, 900 m (González and Vaz-de-Mello 2014: 451). ZAMORA CHINCHIPE: Via Zumbi-Yantzaza km 4, 900 m (González and Vaz-de-Mello 2014: 451). UNDETERMINED PROVINCE: Dureno, 150 m (González and Vaz-de-Mello 2014: 451).

####### Temporal data.

Collected every month of the year.

####### Remarks.

Inhabits the lowland evergreen forests, varzea forests, foothill evergreen forests, and lower evergreen montane forests of the Amazon region from 250–1590 m a.s.l. Collected with flight interception traps and pitfall traps baited with carrion and human feces.

###### Deltochilum (Hybomidium) panamensis

Taxon classificationAnimaliaColeopteraScarabaeidae

Howden, 1966

Plate 23D

Deltochilum (Hybomidium) gibbosumpanamensis Howden, 1966: 736 (original description. Type locality: Río Changuena [= Río Changuinola], 2400 feet [= 730 m], Bocas del Toro, Panama).
Deltochilum gibbosum panamensis : Howden and Young 1981: 36 (characters in key), 37 (redescription); Medina et al. 2001: 136 (cited for Colombia); Ratcliffe 2002: 13 (cited for Panama); Krajcik 2012: 88 (complete list of species); Solís and Kohlmann 2012: 4 (cited for Costa Rica).
Deltochilum (Hybomidium) gibbosumpanamensis : González et al. 2009: 270 (redescription, characters in key).
Deltochilum (Hybomidium) panamensis : González-Alvarado and Vaz-de-Mello 2014: 440 (cited as new status, redescription), 472 (characters in key); Chamorro et al. 2018: 94 (cited for Ecuador).

####### Type specimens.

Deltochilum (Hybomidium) gibbosumpanamensis Howden, 1966. The holotype (♂) is deposited at the CMNC (see Howden 1966: 736, fig 20). Locality: Río Changuena [= Río Changuinola], 2400 feet [= 730 m], Bocas del Toro, Panama, not examined.

####### Distribution.

Colombia, Ecuador, Costa Rica, and Panama.

####### Records examined.

BOLIVAR: Bosque Protector Filo Palanga, 970 m (1 specimen MUTPL). EL ORO: San Roque, 930 m (3 specimens MQCAZ). ESMERALDAS: Alto Tambo, 850 m (4 specimens MQCAZ); Carondelet (5 specimens MECN); Colón del Ónzole (25 specimens MQCAZ, 7 specimens MECN); Charco Vicente (15 specimens MGO-UC; 11 specimens MECN; 17 specimens MQCAZ); Chispero (17 specimnes MQCAZ; 13 specimens MECN); El Progreso (7 specimens MQCAZ; 3 specimens MECN); Guadal (5 specimens MQCAZ); Gualpi (12 specimens MQCAZ; 18 specimens MECN); Majua (19 specimens MQCAZ, 7 specimens MECN); Palma Real (7 specimens MGO-UC; 15 specimens MECN; 11 specimens MQCAZ); Playa de Oro (1 specimen CEMT; 8 specimens MQCAZ); Playa de Oro, Estero Pote, 200 m (14 specimens CEMT; 21 specimens MQCAZ); Playa de Oro, Padre Santo (58 specimens MGO-UC; 15 specimens MECN; 33 specimens MQCAZ); Playa de Oro, Playa Rica (30 specimens MGO-UC; 17 specimens MQCAZ; 4 specimens MECN); Playa de Oro Pistolas (2 specimens MQCAZ); Playa de Oro, Río Santiago, 200 m (4 specimens MQCAZ); Ricauter (8 specimens MQCAZ; 2 specimens MECN); Tsejpi (17 specimnes MQCAZ; 7 specimens MECN); Tsejpi rio Zapallo (5 specimens MQCAZ); Vainilla (5 specimens MQCAZ). IMBABURA: El Chontal, El Cauchero, 900 m (1 specimen MUTPL); Lita, 680 m (3 specimens MQCAZ). MANABÍ: Ayampe, 35 m (1 specimen MUTPL); Puerto López Comunidad Agua Blanca, 245 m (1 specimen MUTPL); Puerto López, Guale, 200 m (1 specimen MUTPL); Puerto López Río Chico (1 specimen MUTPL). PICHINCHA: Llurimaguas, Río Guayllabamba, 290 m, Pedro Vicente Maldonado (1 specimen MGO-UC); El Tigre Río Guayllabamba, Pedro Vicente Maldonado (1 specimen MUTPL); Guayabilla, 520 m, Río Guayllabamba Manduriacus (1 specimen MGO-UC). SANTA ELENA: Olón, 50 m (1 specimen MUTPL). SANTO DOMINGO DE LOS TSÁCHILAS: Valle Hermoso km 24 road to Santo Domingo (1 specimen MGO-UC).

####### Literature records.

ESMERALDAS: Pajonal (González and Vaz-de-Mello 2014: 439) Playa de Oro, Estero Pote (González and Vaz-de-Mello 2014: 439); Playa de Oro (González and Vaz-de-Mello 2014: 440); 5 m, 11 km SE S. Lorenzo [= San Lorenzo] (González and Vaz-de-Mello 2014: 440); La Chiquita, For. Stat (González and Vaz-de-Mello 2014: 440). Pichincha: 113 km N Puerto Quito, 2420–2680 m (González and Vaz-de-Mello 2014: 440).

####### Temporal data.

Collected every month of the year.

####### Remarks.

Inhabits coastal lowland evergreen forests, coastal lowland semi-deciduous forests, and coastal evergreen foothill forests from 200–930 m a.s.l. Collected with pitfall traps baited with carrion and human feces.

#### Genus *Dendropaemon* Perty, 1830

*Dendropaemon* Perty, 1830: 38 (original description. Type species: *Eurysternuspiceus* Perty, 1830 by subsequent designation by Blut, 1939: 267).

*Dendropaemon*: Agassiz 1846: 343 (catalog, unjustifiably cited as *Dendropemon*); Lacordaire 1856: 102 (redescription); Gemminger and Harold 1869: 1020 (list, distribution); Gillet 1911a: 88 (complete list of species); Lucas 1920: 230 (catalog, distribution, cited as *Dendropemon* O Perty); d’Olsoufieff 1924: 19 (characters in key), 121 (redescription); Blut 1939: 267 (redescription); Pessôa and Lane 1941: 470 (characters in key); Blackwelder 1944: 210 (list of species of Latin America); Martínez 1959: 106 (list of species from Argentina); Halffter and Matthews 1966: 258 (catalog, distribution); Vulcano and Pereira 1967: 566 (characters in key); Edmonds 1972: 843 (description); Halffter and Edmonds 1982: 136 (catalog, distribution); Edmonds 1994: 17 (characters in key); Medina and Lopera 2000: 301 (characters in key); Vitolo 2000: 593 (characters in key); Vaz-de-Mello 2000: 192 (list of species from Brazil); Medina et al. 2001: 140 (list of species from Colombia); Arnaud 2002a: 14 (characters in key); Vitolo 2004: 292 (redescription); Hamel-Leigue et al. 2006: 17 (list of species from Bolivia); Hamel-Leigue et al. 2009: 59 (distribution of records from Bolivia); Vaz-de-Mello et al. 2011: 24 (characters in key); Carvajal et al. 2011: 142 (diagnosis); Krajcik 2012: 89 (complete list of species); Boilly and Vaz-de-Mello 2013: 107 (characters in key); Figueroa et al. 2014: 136 (distributional records from Peru); Génier and Arnaud 2016: 6 (revision); Chamorro et al. 2018: 75 (characters in key), 94 (list of species of Ecuador).

*Tetramereia* Klages, 1907: 141 (original description. Type species: *Tetramereiafredereckii*Klages 1907 = *Dendropaemonconvexum* Harold, 1869); Gillet 1911a: 88 (cited as synonym of genus *Dendropaemon* Perty, 1830); Lucas 1920: 634 (catalog, cited as synonym of genus *Dendropaemon* Perty, 1830); d’Olsoufieff 1924: 159 (cited as synonym of genus *Dendropaemon* Perty, 1830); Pessôa and Lane 1941: 490 (cited as synonym of genus *Dendropaemon* Perty, 1830); Blackwelder 1944: 210 (catalog, cited as synonym of genus *Dendropaemon* Perty, 1830); Halffter and Matthews 1966: 258 (catalog, cited as genus *Tetramereia* Klages); Vulcano and Pereira 1967: 566 (characters in key; cited as genus *Tetramereia* Klages); Edmonds 1972: 819 (characters in key; cited as genus *Tetramereia* Klages), 851 (description); Halffter and Edmonds 1982: 137 (catalog, distribution, cited as genus *Tetramereia* Klages); Edmonds 1994: 17 (characters in key, cited as genus *Tetramereia* Klages); Vitolo 2000: 593 (characters in key, cited as genus *Tetramereia* Klages); Vaz-de-Mello 2000: 195 (list of species from Brazil, cited as genus *Tetramereia* Klages); Arnaud 2002a: 16 (characters in key, cited as genus *Tetramereia* Klages); Vaz-de-Mello et al. 2011: 24 (characters in key, cited as genus *Tetramereia* Klages); Krajcik 2012: 253 (complete list of species, cited as genus *Tetramereia* Klages); Boilly and Vaz-de-Mello 2013: 106 (characters in key, cited as genus *Tetramereia* Klages); Figueroa et al. 2014: 137 (distributional records from Peru, cited as genus *Tetramereia* Klages); Génier and Arnaud 2016: 88 (cited as synonym of the genus *Dendropaemon* Perty, 1830).

*Boucomontius* d’Olsoufieff, 1924: 120 (original description. Type species: *Dendropaemonconvexum* Harold, 1869); Blut 1939: 296 (redescription, cited as genus *Boucomontius* d’Olsoufieff, 1924); Pessôa and Lane 1941: 488 (redescription, cited as genus *Boucomontius* d’Olsoufieff); Blackwelder 1944: 21 (catalog, cited as synonym of genus *Eurypodea* Klages, 1906); Halffter and Matthews 1966: 258 (catalog, cited as synonym of genus *Tetramereia* Klages, 1907); Edmonds 1972: 851 (cited as synonym of genus *Tetramereia* Klages, 1907); Génier and Arnaud 2016: 41 (cited as synonym of genus *Dendropaemon* Perty, 1830).

##### Subgenus Dendropaemon (Crassipaemon) Cupello & Génier, 2017

Dendropaemon (Crassipaemon) Cupello & Génier, 2017: 823 (redescription, distribution, cited as new subgenus of *Dendropaemon* Perty, 1830. Type species: *Dendropaemonamyntas* Lacordaire, 1856); Chamorro et al. 2018: 75 (characters in key), 94 (list of species of Ecuador).

Dendropaemon (Onthoecus) Dejean, 1833: 140 (nom. nud.); Agassiz 1846: 749 (catalog); Lacordaire 1856: 103 (original description. Type species: *Dendropaemonamyntas* Harold, 1868 = Dendropaemon (Onthoecus) attalus Génier & Arnaud, 2016); Burmeister 1861: 56 (cited as synonym of genus *Enicotarus* Lap.); Gemminger and Harold 1869: 1020 (list, cited as synonym of genus *Dendropaemon* Perty, 1830); Blut 1939: 267 (cited as synonym of genus *Dendropaemon* Perty); Edmonds 1972: 850 (cited as synonym of subgenus Dendropaemon Perty, s. str.); Génier and Arnaud 2016: 55 (cited as subgenus of *Dendropaemon* Perty, 1830), 86 (characters in key).

###### Dendropaemon (Crassipaemon) morettoi

Taxon classificationAnimaliaColeopteraScarabaeidae

Génier & Arnaud, 2016

Plate 24A

Dendropaemon (Onthoecus) morettoi Génier & Arnaud, 2016: 61 (original description. Type locality: Santé Fé/ de Bogota).
Dendropaemon (Crassipaemon) morettoi : Cupello and Génier 2017: 823 (comment, distribution); Chamorro et al. 2018: 82 (figure 5C), 94 (cited for Ecuador).

####### Type specimens.

Dendropaemon (Onthoecus) morettoi Génier & Arnaud, 2016. The lectotype (♂) is deposited at the MNHN (see: Génier and Arnaud 2016: 62). Locality: Santé Fé/ de Bogota [= Santa Fé de Bogotá], not examined.

####### Distribution.

Colombia and Ecuador.

####### Literature records.

MORONA SANTIAGO: Macas (Génier and Arnaud 2016: 62).

####### Temporal data.

It is not known when this species was collected.

####### Remarks.

It is possible that this species occurs in the evergreen foothill forests of the Amazon region. The collection method is unknown.

##### Subgenus Dendropaemon (Glaphyropaemon) Génier & Arnaud, 2016

Dendropaemon (Glaphyropaemon) Génier & Arnaud, 2016: 46 (original description. Type species: *Dendropaemonangustipennis* Harold, 1869), 85 (characters in key); Chamorro et al. 2018: 75 (characters in key), 94 (list of species of Ecuador).

###### Dendropaemon (Glaphyropaemon) angustipennis

Taxon classificationAnimaliaColeopteraScarabaeidae

Harold, 1869

Plate 24B

Dendropaemon angustipennis Harold, 1869a: 99 (original description. Type locality: Ega [= Tefé]).
Dendropaemon angustipennis : Gemminger and Harold 1869: 1020 (list, distribution); Waterhouse 1891b: 57 (redescription); Gillet 1911a: 88 (list, distribution); d’Olsoufieff 1924: 161 (cited as synonym of Dendropaemonbahianus Har.); Blut 1939: 277 (synonym of Dendropaemonsilvanus Blut, 1939); Vulcano and Pereira 1967: 567 (characters in key); Edmonds 1972: 851 (comment); Arnaud 1982a: 115 (catalog of the types of the MNHN); Krajcik 2012: 89 (complete list of species); Figueroa et al. 2014: 136 (cited for Peru); Ratcliffe et al. 2015: 197 (cited for Peru).
Dendropaemon angustipenne : Blackwelder 1944: 210 (misspelled name, list, distribution).
Dendropaemon (Dendropaemon) angustipenne : Vaz-de-Mello 2000: 192 (misspelled name, cited for Brazil).
Dendropaemon (Glaphyropaemon) angustipennis : Génier and Arnaud 2016: 47 (redescription, transferred to the subgenus Glaphyropaemon Génier & Arnaud, 2016), 48 (distribution), 85 (characters in key); Chamorro et al. 2018: 82 (figures 5A and 5B), 94 (cited for Ecuador).
Dendropaemon silvanus Blut, 1939: 277 (cited as nom. nov., original description); Edmonds 1972: 851 (comment); Génier and Arnaud 2016: 47 (synonym of Dendropaemonangustipennis Harold, 1869).
Dendropaemon silvanum : Blackwelder 1944: 210 (list, distribution).
Dendropaemon (Dendropaemon) silvanum : Vaz-de-Mello 2000: 192 (cited for Brazil).

####### Type specimens.

Dendropaemon (Glaphyropaemon) angustipennis Harold, 1869. The lectotype (♀) is deposited at the MNHN (see: Génier and Arnaud 2016: 48). Locality: Ega [= Tefé], not examined.

*Dendropaemonsilvanus* Blut, 1939. Type material not examined.

####### Distribution.

Bolivia, Brazil, Colombia, Ecuador, and Peru.

####### Records examined.

ORELLANA: Estación Científica Yasuní, 250 m (1 specimen MQCAZ).

####### Literature records.

ORELLANA: Estación Científica Yasuní PUCE, 250 m (Génier and Arnaud 2016: 48); Estación de Biodiversidad Tiputini USFQ, Parque Nacional Yasuní (Génier and Arnaud 2016: 48). SUCUMBÍOS: Cuyabeno (Génier and Arnaud 2016: 48).

####### Temporal data.

Collected in February, April, July, and September.

####### Remarks.

Inhabits the lowland evergreen forests of the Amazon at 250 m a.s.l. Collected manually.

#### Genus *Dichotomius* Hope, 1838

*Dichotomius* Hope, 1838: 321 (original description. Type species: *Dichotomiusboreus* (Olivier, 1789) by original designation, see Martínez 1951b: 140).

*Dichotomius*: Agassiz 1846: 353 (catalog); Lucas 1920: 237 (cited as synonym of *Pinotus* Erichson, 1847); Blackwelder 1944: 206 (cited as synonym of *Pinotus* Erichson, 1847); Martínez 1951b: 139 (restored genus, comment); Pereira 1954a: 57 (characters in key); Roze 1955: 44 (list of species from Venezuela); Martínez 1959: 80 (list of species from Argentina); Halffter and Matthews 1966: 257 (catalog, distribution); Vulcano and Pereira 1967: 577 (characters in key); Howden and Young 1981: 13 (characters in key), 123 (redescription); Halffter and Edmonds 1982: 137 (catalog, distribution); Kohlmann and Solís 1997: 344 (redescription); Medina and Lopera 2000: 306 (characters in key); Vaz-de-Mello 2000: 193 (list of species from Brazil); Medina et al. 2001: 138 (list of species from Colombia); Arnett et al. 2002: 49 (characters in key); Ratcliffe 2002: 15 (list of species for Panama); Morón 2003: 49 (list of species from Mexico); Hamel-Leigue et al. 2006: 15 (list of species from Bolivia); Sarmiento-Garcés and Amat-García 2009: 286 (description); Vaz-de-Mello et al. 2011: 20 (characters in key); Carvajal et al. 2011: 129 (diagnosis), 320 (list of species from Ecuador); Krajcik 2012: 91 (complete list of species); Solís and Kohlmann 2012: 6 (list of species from Costa Rica); Boilly and Vaz-de-Mello 2013: 109 (characters in key); Sarmiento-Garcés and Amat-García 2014: 23 (redescription); Chamorro et al. 2018: 77 (characters in key), 94–95 (list of species of Ecuador).

*Pinotus* Erichson, 1847: 108 (original description); Lacordaire 1856: 98 (redescription, designation type species: *Pinotuscarolinus* Linnaeus, 1767); Harold 1869c: 124 (redescription); Gemminger and Harold 1869: 1009 (complete list of species); Bruch 1911: 187 (list of species from Argentina); Gillet 1911a: 59 (complete list of species); Lucas 1920: 514 (catalog, distribution); Luederwaldt 1929: 8 (redescription), 10 (characters in key); Luederwaldt 1931a: 369 (characters in key); Paulian 1938: 234 (characters in key); Pessôa and Lane 1941: 437 (characters in key); Blackwelder 1944: 206 (list of species from Latin America); Martínez 1951b: 139 (synonym of *Dichotomius* Hope, 1838); Martínez 1959: 80 (cited as synonym of *Dichotomius* Hope, 1838); Halffter and Matthews 1966: 257 (cited as synonym of *Dichotomius* Hope, 1838); Howden and Young 1981: 123 (cited as synonym of *Dichotomius* Hope, 1838); Halffter and Edmonds 1982: 137 (cited as synonym of *Dichotomius* Hope, 1838); Kohlmann and Solís 1997: 344 (cited as synonym of *Dichotomius* Hope, 1838); Ratcliffe 2002: 15 (cited as synonym of *Dichotomius* Hope, 1838); Solís and Kohlmann 2012: 6 (cited as synonym of *Dichotomius* Hope, 1838); Sarmiento-Garcés and Amat-García 2014: 23 (cited as synonym of *Dichotomius* Hope, 1838).

*Brachycopris* Haldeman, 1848: 125 (original description. Type species: *Copriscarolina* Linnaeus, 1767); Gemminger and Harold 1869: 1009 (cited as synonym *Pinotus* Erichson, 1847); Gillet 1911a: 59 (cited as synonym *Pinotus* Erichson, 1847); Lucas 1920: 146 (synonym *Pinotus* Erichson, 1847); Blackwelder 1944: 206 (cited as synonym *Pinotus* Erichson, 1847); Martínez 1951b: 139 (comment, synonym of *Pinotus* Erichson, 1847); Martínez 1959: 80 (cited as synonym of *Dichotomius* Hope, 1838); Kohlmann and Solís 1997: 344 (cited as synonym of *Dichotomius* Hope, 1838); Ratcliffe 2002: 15 (cited as synonym of *Dichotomius* Hope, 1838); Solís and Kohlmann 2012: 6 (cited as synonym of *Dichotomius* Hope, 1838); Sarmiento-Garcés and Amat-García 2014: 23 (cited as synonym of *Dichotomius* Hope, 1838).

##### Subgenus Dichotomius (Dichotomius) Hope, 1838

Dichotomius (Dichotomius) s. str. Hope, 1838: 321 (original description. Type species: *Dichotomiusboreus* (Olivier, 1789), original combination); Martínez 1951b: 139 (comment); Martínez 1959b: 81 (list of species for Argentina); Halffter and Edmonds 1982: 137 (cited as subgenus of *Dichotomius* Hope, 1838); Vaz-de-Mello 2000: 193 (list of species from Brazil); Sarmiento-Garcés and Amat-García 2009: 287 (redescription); Vaz-de-Mello et al. 2011: 20 (characters in key); Boilly and Vaz-de-Mello 2013: 109 (characters in key); Sarmiento-Garcés and Amat-García, 2014: 24 (characters in key); Chamorro et al. 2018: 77 (characters in key), 94–95 (list of species of Ecuador).

###### Dichotomius (Dichotomius) compressicollis

Taxon classificationAnimaliaColeopteraScarabaeidae

(Luederwaldt, 1929)

Plate 24C

Pinotus compressicollis Luederwaldt, 1929: 125 (original description. Type locality: Columbia).
Pinotus compressicollis : Blackwelder 1944: 207 (list of species of Latin America); Contreras 1951: 222 (cited for Colombia).
Dichotomius compressicollis : Medina et al. 2001: 138 (cited for Colombia); Krajcik 2012: 91 (complete list of species); Sarmiento-Garcés and Amat-García 2014: 104 (diagnosis, cited as incerta sedis).
Dichotomius (Dichotomius) compressicollis : Chamorro et al. 2018: 104 (cited for Ecuador).

####### Type specimens.

*Pinotuscompressicollis* Luederwaldt, 1929. Two syntypes examined deposited at the MZUSP. Lectotype to be designated in a future work on this species group.

####### Distribution.

Colombia and Ecuador

####### Records examined.

NAPO: Puerto Napo, 480 m (1 specimen MUTPL). ORELLANA: Dayuma, 310 m (1 specimen MUTPL); Dayuma, plataforma Primavera, 300 m (1 specimen CEMT); Rodrigo Borja IAMOE (1 specimen CEMT). PASTAZA: Bosque Protector Oglán Alto, 555–610 m (2 specimens MUTPL); road Triunfo-Arajuno (1 specimen CEMT). SUCUMBÍOS: Pacayacu Campo Libertador (1 specimen CEMT).

####### Temporal data.

Collected in June, August, November, and December.

####### Remarks.

Inhabits the lowland evergreen forests and evergreen foothill forests of the Amazon region from 230–610 m a.s.l. Collected with pitfall traps baited with human feces.

###### Dichotomius (Dichotomius) cotopaxi

Taxon classificationAnimaliaColeopteraScarabaeidae

(Guérin-Méneville, 1855)

Plate 24D

Copris cotopaxi Guérin-Méneville, 1855: 588 (original description. Type locality: Ecuador).
Pinotus cotopaxi : Harold 1869c: 132 (redescription); Gemminger and Harold 1869: 1009 (complete list of species); Harold 1875b: 104 (comment, cited for Ecuador); Bates 1891: 26 (cited for Ecuador); Gillet 1911a: 60 (complete list of species); Campos 1921: 56 (cited for Ecuador); Luederwaldt 1929: 45 (characters in key); Balthasar 1941: 349 (cited for Peru); Blackwelder 1944: 207 (list of species of Latin America); Balthasar 1951: 334 (cited for Peru).
Dichotomius cotopaxi : Pereira 1953: 389 (cited as new combination, comment); Carvajal et al. 2011: 320–321 (cited for Ecuador,); Krajcik 2012: 91 (complete list of species); Arias-Buriticá and Vaz-de-Mello, 2013: 216 (morphology, figure 3); Ratcliffe et al. 2015: 196 (cited for Peru).
Dichotomius (Dichotomius) cotopaxi : Chamorro et al. 2018: 89 (figure 12E), 94 (cited for Ecuador).
Copris scalpellum Taschenberg, 1870: 181 (original description); Harold 1875b: 104 (synonym of Pinotuscotopaxi Guérin, 1855); Luederwaldt 1929: 45 (cited as synonym of Pinotuscotopaxi Guérin, 1855); Pereira 1953: 389 (synonym of Dichotomiuscotopaxi Guérin, 1855).
Pinotus abnormis Luederwaldt, 1923: 3 (original description); Luederwaldt 1929: 46 (characters in key); Blackwelder 1944: 206 (list of species of Latin America); Pereira 1953: 389 (synonym of Dichotomiuscotopaxi Guérin, 1855).

####### Type specimens.

*Copriscotopaxi* Guérin-Méneville, 1855. Type material not examined.

*Coprisscalpellum* Taschenberg, 1870. One syntype examined deposited at the MLHU. Lectotype to be designated in a future work on this species group.

**Syntype** (sex unknown): “Scalpellum / Tascher. 1870 / Loja (Ecuad) Wallis [hw, black margin]”, “MLU.- Halle / WB Zoologie / S.- Nr. 81415 [p and hw]”.

*Pinotusabnormis* Luederwaldt, 1923. Six syntypes examined deposited at the MZUSP. Lectotype to be designated in a future work on this species group.

**Syntype** (♂): “ECUADOR / Bannos / II. 12. 05 F. Ohs. [p]”, “Pinotus ♂ / abnormis Lüd. / Lüd. det. 22 [hw]”, “ forma a [hw]”, “17256 [p]”, “COTIPO [p, pink label]”.

**Syntype** (♀): “Colta 3400 m. / F. Ohs. 9. 7. 05 [p]”, “Pinotus ♀ / abnornis Lüd. / Lüd. det. 22 [hw]”, “17255 [p]”, “COTIPO [p, pink label]”.

**Syntype** (♂): “ S. Ecuador / Loja / F. Ohs. 6. 10. 05 [p]”, “Pinotus ♂ / abnornis Lüd. / Lüd. det. 22. / forma b. [hw]”, “17257 [p]”, “COTIPO [p, pink label]”.

**Syntype** (♀): “Loja Calvario / F. Ohs. 5. 8. 05 [p]”, “Pinotus ♀ / abnornis Lüd. / Lüd. det. 22. / forma b. [hw]”, “17258 [p]”, “COTIPO [p, pink label]”.

**Syntype** (♂): “Riobamba / Cubillin 3500 m / F. Ohs. 5. 7. 05 [p]”, “Pinotus ♂ / abnornis Lüd. / Lüd. det. 22 [hw]”, “17259 [p]”, “COTIPO [p, pink label]”.

**Syntype** (♂): “Riobamba / Cubillin 3500 m / F. Ohs. 5. 7. 05 [p]”, “COTIPO [p, pink label]”, “17258 [p]”, “Pinotus ♂ / abnornis Lüd. / Lüd. det. 22 [hw]”.

####### Distribution.

Ecuador and Peru.

####### Records examined.

AZUAY: SigSig (3 specimens MQCAZ). BOLIVAR: Cashca Totoras (3 specimens MQCAZ); Santiago (2 specimens MQCAZ). CAÑAR: El Tambo (2 specimens MQCAZ); road to Taday, 3370 m (1 specimen MUTPL). COTOPAXI: Callo Caspi, Parque Nacional Cotopaxi (5 specimens MQCAZ); Cusubamba (2 specimens MQCAZ); Latacunga (4 specimens MQCAZ); Laso, 3400 m (3 specimens MQCAZ); Salcedo (4 specimens MQCAZ). CHIMBORAZO: Candelaria, 3050 (2 specimen CEMT); Colta, 3400 m (1 specimen MZUSP); Penipe (1 specimen MQCAZ); Riobamba-Cubillin, 3500 m (1 specimen MZUSP). LOJA: Angashcola, 2740 m (5 specimen MUTPL); Argelia (3 specimen MQCAZ); Loja, Amauta (1 specimen MUTPL); Loja, Cerro El Villonaco, 2740 m (4 specimen MUTPL); Rocafuerte, 2900 m (1 specimen CEMT); San Lucas, 2475 m (2 specimens MQCAZ); without specific locality (1 specimen MLHU, 1 specimen MZUSP). TUNGURAGUA: Bannos [= Baños] (1 specimen MZUSP); Baños (3 specimens CEMT); Baños, El Pelotero (1 specimen MQCAZ); Pillaro, 2850 m (2 specimens CEMT).

####### Literature records.

BOLIVAR: Telimbela (Campos 1921: 56). CHIMBORAZO: Chunchi (Campos 1921: 56); Riobamba-Cubillin, 3500 m (Luederwaldt 1923: 3). LOJA: Colta, 3400 m (Luederwaldt 1923: 3); Loja, El Calvario (Luederwaldt 1923: 3); without specific locality (Harold 1875b: 104; Luederwaldt 1923: 3; Taschenberg 1870: 181). Pichincha: Nanegal (Campos 1921: 56); Mindo (Campos 1921: 56). TUNGURAGUA: Bannos [= Bannos]. UNDETERMINED PROVINCE: La Asuncion (Campos 1921: 56); Pacific slopes, below 1400 feet (Bates 1891: 26).

####### Temporal data.

Collected in January, February, March, April, May, June, October, November, and December.

####### Remarks.

Inhabits the montane cloud forests and the evergreen high montane forest from of the Andean region from 2270–3500 m a.s.l. Collected manually and with pitfall traps baited with human feces. According to our data, *D.cotopaxi* is distributed across the eastern side of the Andes. However, Campos (1921) cited the following four localities for this species: Telimbela, La Asunción, Mindo, and Nanegal, all located in the western side of the Andes, below 2000 m a.s.l. It is possible that these specimens were misidentified.

###### Dichotomius (Dichotomius) divergens

Taxon classificationAnimaliaColeopteraScarabaeidae

(Luederwaldt, 1923)

Plate 25A

Pinotus divergens Luederwaldt, 1923: 3 (original description. Type locality: Bucay, west Ecuador, 300 m).
Pinotus divergens : Luederwaldt 1929: 36 (characters in key); Blackwelder 1944: 206 (list of species from Latin America).
Dichotomius divergens : Medina et al. 2001: 138 (cited for Colombia); Carvajal et al. 2011: 320–321 (cited for Ecuador); Krajcik 2012: 91 (complete list of species); Sarmiento-Garcés and Amat-García 2014: 51 (characters in key), 71 (diagnosis).
Dichotomius (Dichotomius) divergens : Chamorro et al. 2018: 94 (cited for Ecuador).

####### Type specimens.

*Pinotusdivergens* Luederwaldt, 1923. Two syntypes examined deposited at the MZUSP. Lectotype to be designated in a future work on this species group.

**Syntype** (♂): “W. ECUADOR / Pucay / 1–5.XII.05. F. Ohs. 5. 7. 05 [p]”, “COTIPO [p, pink label]”, “Pinotus ♂ / divergens L. / Lüd. det. 22 [hw]”.

**Syntype** (♂): “W. ECUADOR / Pucay / 1–5.XII.05. F. Ohs. 5. 7. 05 [p]”, “COTIPO [p, pink label]”, “Pinotus ♂ / divergens L. / Lüd. det. 22 [hw]”.

####### Distribution.

Ecuador and Colombia.

####### Records examined.

BOLIVAR: Bosque Protector Filo Palanga, 970 m (1 specimen MUTPL). CARCHI: Tobar Donoso, 300 m (6 specimens MECN). COTOPAXI: Guasaganda km 4, 500 m (7 specimens MQCAZ). EL ORO: Bella María Los Ingleses, 440 m (2 specimens MUTPL); Buenaventura Bajo, 500 m (1 specimen MUTPL); Reserva Jocotoco, 1250 m (1 specimen MQCAZ). ESMERALDAS: Carondelet (8 specimens MQCAZ); Colon del Ónzole (21 specimens MQCAZ; 17 specimens MECN); Chispero (11 specimens MQCAZ; 15 specimens MECN); El Progreso (3 specimens MQCAZ; 8 specimens MECN); Gallinazo (4 specimens MQCAZ); Jeyambi (7 specimens MQCAZ; 11 specimens MECN); Majua (17 specimens MQCAZ; 20 specimens MECN); Los Ajos (2 specimens MQCAZ); Pajonal (9 specimens MQCAZ; 16 specimens MECN); Palma Real (1 specimen CEMT; 25 specimens MQCAZ; 17 specimens MECN); Playa de Oro, La Tabla (26 specimens MQCAZ; 11 specimens MECN); Playa de Oro, Padre Santo (33 specimens MQCAZ; 20 specimens MECN); Playa de Oro, Pote (3 specimens CEMT; 16 specimens MQCAZ; 8 specimens MECN); Playa de Oro, Playa Rica (7 specimens MQCAZ; 3 specimens MECN); Ricauter (1 specimen MQCAZ); Salto del Bravo (9 specimens MQCAZ; 5 specimens MECN); Tjespi (19 specimens MQCAZ; 5 specimens MECN); Zabalito (4 specimens MQCAZ). GUAYAS: Pucay [= Bucay] (1 specimen MZUSP). IMBABURA: Lita, 685 m (2 specimens CEMT; 2 specimens MECN). LOS RÍOS: Río Palenque Science Ctr, 150–220 m (7 specimens MQCAZ). PICHINCHA: Chespi, 1300 m, Bellavista (2 specimens MUTPL); El Encuentro, 620 m, San Miguel de los Bancos (1 specimen MUTPL); El Tigre Río Guayllabamba, Pedro Vicente Maldonado (1 specimen MUTPL); Estación Biológica la Hesperia, 1200 m (4 specimens MUTPL); Río Guayllabamba Guayabilla, 520 m, Manduriacus (3 specimens MUTPL); Río Guayllabamba LLurimaguas, 290 m, Pedro Vicente Maldonado (2 specimens MUTPL); Mangaloma, 720 m, San Miguel de los Bancos (1 specimen MUTPL); Nanegalito, 1000 m (5 specimens MQCAZ); Río Guayllabamba Tortugo, 450 m, Pedro Vicente Maldonado (2 specimens MUTPL); Río Guayllabamba San Roque, 580 m, Pedro Vicente Maldonado (1 specimen MUTPL). SANTO DOMINGO DE LOS TSÁCHILAS: 2 km N de Alluriquin, 1070 m (1 specimen MUTPL); 47 km S de Santo Domingo (3 specimens CEMT); Río Toachi, 900 m (5 specimens MQCAZ); Tinalandia, 650 m (1 specimen CEMT).

####### Literature records.

GUAYAS: Bucay, 300 m (Luederwaldt 1923: 4).

####### Temporal data.

Collected every month of the year.

####### Remarks.

Inhabits coastal lowland evergreen forests and coastal evergreen foothill forests from 150–1300 m a.s.l. Collected with pitfall traps baited with carrion, cow dung, and human feces.

###### Dichotomius (Dichotomius) mamillatus

Taxon classificationAnimaliaColeopteraScarabaeidae

(Felsche, 1901)

Plate 25B

Pinotus mamillatus Felsche, 1901: 143 (original description. Type locality: Ecuador).
Pinotus mamillatus : Gillet 1911a: 61 (complete list of species); Luederwaldt 1929: 53 (characters in key); Balthasar 1941: 349 (cited for Peru); Blackwelder 1944: 207 (list of species from Latin America); Balthasar 1951: 334 (cited for Peru).
Dichotomius mamillatus : Vulcano and Pereira 1967: 585 (characters in key); Vaz-de-Mello 2000: 193 (cited for Brazil); Medina et al. 2001: 138 (cited for Colombia); Sarmiento-Garcés and Amat-García 2009: 291 (diagnosis), 295 (characters in key); Carvajal et al. 2011: 320–321 (cited for Ecuador); Krajcik 2012: 91 (complete list of species); Sarmiento-Garcés and Amat-García 2014: 53 (characters in key), 73 (diagnosis); Ratcliffe et al. 2015: 196 (cited for Peru).
Dichotomius (Dichotomius) mamillatus : Chamorro et al. 2018: 94 (cited for Ecuador).

####### Types specimens.

*Pinotusmamillatus* Felsche, 1901. One syntype (♂) examined deposited at the SMTD. Lectotype to be designated in a future work on this species group.

**Syntype** (♂): “Ecuador / Baron [p]”, “Typus [p, red label, black margin]”, “Coll. C. Felsche / Kauf 20, 1918 [p, green label, black margin]”, “mamillatus / Felsche / Ecuador [hw, purple label]”, “Staatl. Museum für / Tierkunde D esden [p]”.

####### Distribution.

Brazil, Colombia, Ecuador, and Peru.

####### Records examined.

MORONA SANTIAGO: Cumpi, Cordillera del Kutukú (1 specimen MUTPL); road Mendez-Paute, 1250 m (2 specimens CEMT; 5 specimens MQCAZ); NAPO: Nor Oeste de Puerto Napo, 470 m, Pungarayacu (1 specimen MQCAZ); Tena (3 specimens CEMT). ORELLANA: Bloque 31 Parque Nacional Yasuní (5 specimens MECN); Dayuma Campo Hormiguero, plataforma Hormiguero, 320 m (1 specimen MUTPL); Dayuma Campo Palanda-Yuca Sur plataforma Yuca 13, 255 m (1 specimen MGO-UC); Dayuma plataforma Ungurahua, 300 m (1 specimen MUTPL); El Dorado plataforma Guarango, 300 m (1 specimen MUTPL); Estación Científica Yasuní Puce, 250 m (19 specimens MQCAZ); Estación de Biodiversidad Tiputini, 285 m, Parque Nacional Yasuni (7 specimes MUTPL); Rodrigo Borja IAMOE (1 specimen CEMT; 9 specimens MQCAZ); San Sebastian del Coca, 345 m, Comuna Guataraco, Campo Pata (1 specimen MUTPL); San Sebastián del Coca, 345 m, Comuna Shamanal, Campo Palo Azul (1 specimen MUTPL). PASTAZA: Bosque Protector Oglán Alto, 660 m (1 specimen MUTPL); Chuyayacu, 200 m, Oleoducto km 25 (1 specimen MUTPL); Nuevo San Jose del Curaray, 245 m (2 specimens MUTPL); Pandanuque, 420 m (1 specimen MUTPL); San Virgilio (1 specimen MUTPL); Villano (3 specimens MECN). SUCUMBÍOS: Limoncocha, 250 m (2 speciemens CEMT); 6 km de Dureno, Precooperativa Los Vergeles, 290 m (4 specimens MGO-UC); Aucayacu Río El Eno, 275 m, 16 km de Lago Agrio (2 specimens MGO-UC); Nueva Loja plataforma Iguana, 310 m (1 specimen MUTPL); Pacayacu, 265 m, Campo Libertador, Tapi (1 specimen MUTPL); Tarapoa Campo Marian, plataforma Fanny 5, 260 m (1 specimen MUTPL); Tarapoa, Nuevo Manabí, 270 m (1 specimen MUTPL); Zabalo, 520 m (1 specimen CEMT). ZAMORA CHINCHIPE: Tundayme campamento, 800 m (2 specimens MUTPL); Tundayme campamento Mirador, San Marcos, 900 m (1 specimen MUTPL); Tundayme campamento Mirador, Las Maravillas, 1060 m (1 specimen COSEC); Zurmi, Comunidad La Wants, 1010 m (2 specimens MEPN; 1 specimen MUTPL); Zurmi Las Orquideas Río Nangaritza, 870 m (1 specimen MUTPL). UNDETERMINED PROVINCE: without specific locality (1 specimen SMTD).

####### Temporal data.

Collected every month of the year.

####### Remarks.

Inhabits the lowland evergreen forests, varzea forests and the foothill evergreen forests of the Amazon region from 200–1060 m a.s.l. Collected with flight interception traps and pitfall traps baited with carrion and human feces.

###### Dichotomius (Dichotomius) monstrosus

Taxon classificationAnimaliaColeopteraScarabaeidae

(Harold, 1875)

Plate 25C

Pinotus monstrosus Harold, 1875c: 210 (original description. Type locality: Medellin und La Mesa Nue Granda [= Nueva Granada, Colombia]).
Pinotus monstrosus : Gillet 1911a: 61 (complete list of species); Campos 1921: 56 (cited for Ecuador); Luederwaldt 1929: 45 (characters in key); Blackwelder 1944: 207 (list of species from Latin America); Contreras 1951: 222 (cited for Colombia).
Dichotomius monstrosus : Medina et al. 2001: 138 (cited for Colombia); Carvajal et al. 2011: 320–321 (cited for Ecuador); Krajcik 2012: 91 (complete list of species); Arias-Buriticá and Vaz-de-Mello 2013: 216 (distribution); Sarmiento-Garcés and Amat-García 2014: 34 (characters in key), 35 (diagnosis).
Dichotomius (Dichotomius) monstrosus : Chamorro et al. 2018: 94 (cited for Ecuador).

####### Type specimens.

*Pinotusmonstrosus* Harold, 1875. Two syntypes examined deposited at the MNHN (ex coll. E Steinheil). Lectotype to be designated in a future work on this species group.

####### Distribution.

Colombia and Ecuador.

####### Records examined.

CARCHI: El Carmelo, 2800 m (2 specimens MUTPL). NAPO: Cosanga, 2150 m (1 specimen MEPN); Cordillera de los Guacamayos (1 specimen CEMT); Oyacachi road to Chaco, 2600 m (1 specimen CEMT); Oyacachi, 2350 (1 specimen CEMT); Sumaco, 3100 m (2 specimen MQCAZ). SUCUMBÍOS: La Bonita (1 specimen CEMT).

####### Literature records.

MORONA SANTIAGO: Macas (Campos 1921: 56). PASTAZA: Canelos (Campos 1921: 56); Sarayacu (Campos 1921: 56).

####### Temporal data.

Collected in January, February, March, July, October, and November.

####### Remarks.

Inhabits the montane cloud forests and the evergreen high montane forests of the Andean region from 2150–3100 m a.s.l. Collected manually.

###### Dichotomius (Dichotomius) ohausi

Taxon classificationAnimaliaColeopteraScarabaeidae

(Luederwaldt, 1923)

Plate 25D

Pinotus ohausi Luederwaldt, 1923: 6 (original description. Type locality: Napo, Ecuador).
Pinotus ohausi : Luederwaldt 1929: 33 (characters in key); Blackwelder 1944: 207 (list of species of Latin America).
Dichotomius ohausi : Vaz-de-Mello 2000: 193 (cited for Brazil); Medina et al. 2001: 138 (cited for Colombia); Sarmiento-Garcés and Amat-García 2009: 288 (redescription), 295 (characters in key); Carvajal et al. 2011: 320–321 (cited for Ecuador); Krajcik 2012: 91 (complete list of species); Sarmiento-Garcés and Amat-García 2014: 50 (characters in key), 56 (diagnosis), 57 (cited for Ecuador); Ratcliffe et al. 2015: 196 (cited for Peru).
Dichotomius (Dichotomius) ohausi : Chamorro et al. 2018: 94 (cited for Ecuador).

####### Type specimens.

*Pinotusohausi* Luederwaldt, 1923. One syntype examined deposited at the MZUSP. Lectotype to be designated in a future work on this species group.

**Syntype** (♂): “-Napo- / (Ecuad.) / R. Haensch S [p]”, “Pinotus ♂ / divergens L. / Lüd. det. 22 [hw]”, “17130 [p]”, “F. Pereira 1941 [hw]”, “COTIPO [p, red label, black margin]”.

####### Distribution.

Brazil, Colombia, Ecuador, and Peru.

####### Records examined.

MORONA SANTIAGO: Koan, Cordillera del Kutukú (2 specimens MUTPL); NAPO: Bloque 20, Pungarayacu, 610 m (1 specimen MQCAZ); Estación Biológica Jatun Sacha, 400 m, 21 km E de Puerto Napo (8 specimens MECN); without specific locality (1 specimen MZUSP). ORELLANA: Cononaco, Bloque 16 YPF, Parque Nacional Yasuní, 250 m (2 specimens MUTPL); Dayuma Campo Hormiguero, plataforma Hormiguero, 320 m (1 specimen MUTPL); Dayuma Campo Palanda, LLumpac, 295 m (1 specimen MGO-UC; 1 specimen MUTPL); El Dorado plataforma Guarango, 300 m (1 specimen MUTPL); Estación de Biodiversidad Tiputini USFQ, 220 m, Parque Nacional Yasuní (1 specimen MUTPL); Estación Yasuní PUCE, 300 m, Parque Nacional Yasuní (19 specimens MQCAZ); Pozo Daimi 1 (1 specimen CEMT); Rodrigo Borja IAMOE (2 specimens CEMT; 9 specimens MECN); San Sebastian de Coca, 345 m, Comuna Guataraco Campo Pata (1 specimen MUTPL); San Sebastián del Coca, 345 m, Comuna Shamanal Campo Palo Azul (1 specimen MUTPL); Lago San Pedro, plataforma Copal 310 m (1 specimen MUTPL). PASTAZA: Bosque Protector Oglán Alto, 660–810 m (1 specimen MUTPL); road Triunfo-Arajuno (1 specimen CEMT). SUCUMBÍOS: Limoncocha, 250 m (1 specimen MUTPL); 6 km de Dureno, Precooperativa Los Vergeles, 290 m (2 specimens MGO-UC); Bermejo plataforma ER-A road to Lumbaqui (1 specimen MUTPL); Gonzalo Pizarro, Simón Bolivar, 1200 m (5 specimens MECN); Nueva Loja, plataforma Iguana, 310 m (1 specimen MUTPL); Pacayacu Campo Libertador, Tapi, 260 m (1 specimen MUTPL); Pacayacu Campo Libertador, Tetete, 290 m (2 specimens MUTPL); Shushufindi (5 specimens MECN); Tarapoa Campo Marian, plataforma Fanny 5, 260 m (1 specimen MUTPL); Tarapoa, Nuevo Manabí, 270 m (1 specimen MUTPL).

####### Temporal data.

Collected every month of the year.

####### Remarks.

Inhabits the lowland evergreen forests, varzea forests and the foothill evergreen forests of the Amazon region from 200–1060 m a.s.l. Collected with pitfall traps baited with carrion and human feces.

###### Dichotomius (Dichotomius) podalirius

Taxon classificationAnimaliaColeopteraScarabaeidae

(Felsche, 1901)

Plate 26A

Pinotus podalirius Felsche, 1901: 137 (original description. Type locality: Ecuador).
Pinotus podalirius : Gillet 1911a: 61 (complete list of species, cited as DichotomiusPodalirius Felche); Luederwaldt 1929: 27 (characters in key), 29 (redescription); Blackwelder 1944: 207 (list of species from Latin America).
Dichotomius podalirius : Vulcano and Pereira 1967: 585 (characters in key); Martínez and Martínez 1982: 3 (comment); Medina et al. 2001: 138 (cited for Colombia); Sarmiento-Garcés and Amat-García 2009: 288 (diagnosis), 294 (characters in key); Carvajal et al. 2011: 320–321 (cited for Ecuador); Krajcik 2012: 91 (complete list of species); Sarmiento-Garcés and Amat-García 2014: 41 (characters in key), 46 (diagnosis).
Dichotomius (Dichotomius) podalirius : Chamorro et al. 2018: 89 (figure 12G), 94 (cited for Ecuador).

####### Type specimens.

*Pinotuspodalirius* Felsche, 1901. One syntypes examined deposited at the SMTD. Lectotype to be designated in a future work on this species group.

**Syntype** (♂): “Ecuador / Baron [p]”, “Coll. C. Felsche / Kauf 20, 1918 [p, green label, black margin]”, “Staatl. Museum für / Tierkunde D esden [p]”.

####### Distribution.

Brazil, Colombia, and Ecuador.

####### Records examined.

NAPO: Bloque 20, Pungarayacu, 610 m (1 specimen MUTPL); Estación Biológica Jatun Sacha, 400 m, 21 km E de Puerto Napo (4 specimens MECN); Tena (1 specimen CEMT). ORELLANA: Cononaco, Bloque 16 YPF, Parque Nacional Yasuní, 250 m (2 specimens MUTPL); Dayuma, Campo Palanda plataforma Pindo 14, 255 m (1 specimen MUTPL); Dayuma plataforma Ungurahua, 300 m (1 specimen MUTPL); El Dorado plataforma Guarango, 300 m (1 specimen MUTPL); Estación Científica Yasuní PUCE, 200 m (6 specimens CEMT; 18 specimens MQCAZ); Estación de Biodiversidad Tiputini, 285 m, Parque Nacional Yasuni (2 specimens MUTPL); Rodrigo Borja IAMOE (1 specimen CEMT; 5 specimens MQCAZ); San Sebastian del Coca, 345 m, Comuna Guataraco Campo Pata (1 specimen MUTPL). PASTAZA: Bosque Protector Oglán Alto, 555–950 m (1 specimen MUTPL); Nuevo San Jose del Curaray, 245 m (1 specimen MUTPL); Ñemenguno, 280 m (1 specimen MUTPL); San Virgilio (1 specimen MUTPL); Villano (1 specimen CEMT; 3 specimens MQCAZ). SUCUMBÍOS: 6 km de Dureno, Precooperativa Los Vergeles, 290 m (1 specimen MGO-UC); km 10 Trocha Zabalo-Guepi, 220 m, Reserva de Producción Faunística Cuyabeno (1 specimen MUTPL); Bermejo plataforma ER-A road to Lumbaqui (1 specimen MUTPL); Lago Agrio (3 specimens MECN); Nueva Loja, plataforma Iguana, 310 m (1 specimen MUTPL); Pacayacu Campo Libertador, 260 m (3 specimens MUTPL); Sansahuari, 290 m, Pozo Singüe (1 specimen MUTPL); Tarapoa Campo Marian, plataforma Fanny 5, 260 m (1 specimen MUTPL); Tarapoa, Nuevo Manabí, 270 m (1 specimen MUTPL); Tipirishca (2 specimen MECN). ZAMORA CHINCHIPE: Tundayme campamento, 800 m (1 specimen MUTPL); Tundayme, campamento Mirador, San Marcos, 900 m (1 specimen MUTPL); Zurmi, Comunidad La Wants, 1010 m (3 specimens MUTPL); Zurmi Las Orquideas Río Nangaritza, 870 m (1 specimen MUTPL). UNDETERMINED PROVINCE: without specific locality (1 specimen SMTD).

####### Temporal data.

Collected every month of the year.

####### Remarks.

Inhabits the lowland evergreen forests and the foothill evergreen forests of the Amazon region from 220–1010 m a.s.l. Collected with pitfall traps baited with carrion and human feces.

###### Dichotomius (Dichotomius) prietoi

Taxon classificationAnimaliaColeopteraScarabaeidae

Martínez & Martínez, 1982

Plate 26B

Dichotomius (*D*) prietoi Martínez & Martínez, 1982: 3 (original description. Type locality: Departamento de Cochabamba, provincia de Chaparé, Chimoré, 250–450 m).
Dichotomius prietoi : Vaz-de-Mello 2000: 193 (cited for Brazil); Medina et al. 2001: 138 (cited for Colombia); Krajcik 2012: 92 (complete list of species); Sarmiento-Garcés and Amat-García 2014: 108 (comment table 2); Ratcliffe et al. 2015: 196 (cited for Peru).
Dichotomius (Dichotomius) prietoi : Chamorro et al. 2018: 94 (cited for Ecuador).

####### Types specimens.

*Dichotomius* (*D*) *prietoi* Martínez & Martínez, 1982. The holotype (♂) is deposited at the MACN. Locality: Boliva, Dpto. Cochabamba, Chimore, 250 m. Examined.

**Holotype**: “Ene: 972 / BOLIVIA / D° Cachabamba / Pcia Chapare / Chimore, 250 m / Coll. Martínez [hw]”, “Dichotomius (D.) prietoi / sp. nov. / ♂ A. Martínez y / A. MARTÍNEZ DET. 1981 [p and hw, pink label, black margin]”, “MACN-En / 1574 [p]”, “HOLOTYPUS [p, orange label]”.

####### Distribution.

Bolivia, Ecuador, and Peru.

####### Records examined.

MORONA SANTIAGO: Comunidad Angel Rouby Sitio 8, Cordillera del Kutukú, 1300 m (9 specimens MQCAZ); Comunidad Unsuants sitio 3, Cordillera del Kutukú, 700 m (1 specimen MUTPL; 11 specimens MQCAZ). ZAMORA CHINCHIPE: Tundayme campamento Mirador, San Marcos, 900 m (3 specimens MUTPL); Tundayme campamento Mirador, Escombrera Norte, 1245 m (2 specimens MUTPL); road Namirez-Zamora km 1, 1000 m (4 specimens MQCAZ); Río Nangaritza, 1000 m (3 specimens MQCAZ); Zurmi, Comunidad La Wants, 1010 m (2 specimens MEPN; 2 specimens MUTPL); Zurmi, Reserva Maycu, 875 m (1 specimen MUTPL); Comunidad La Wants, 1010 m (2 specimens MUTPL; 2 specimens MEPN); Zurmi Las Orquideas Río Nangaritza, 870 m (1 specimen MUTPL).

####### Temporal data.

Collected in January, February, April, May, July, August, September, November, and December.

####### Remarks.

Inhabits the foothill evergreen forests across the Amazonian range from 700–1300 m a.s.l. Collected manually and with pitfall traps baited with carrion and human feces.

###### Dichotomius (Dichotomius) protectus

Taxon classificationAnimaliaColeopteraScarabaeidae

(Harold, 1867)

Plate 26C

Pinotus protectus Harold, 1867e: 98 (original description. Type locality: Columbia [= Colombia]).
Pinotus protectus : Harold 1869c: 130 (redescription); Gemminger and Harold 1869: 1010 (complete list of species); Gillet 1911a: 61 (complete list of species); Campos 1921: 56 (cited for Ecuador); Luederwaldt 1929: 34 (characters in key); Blackwelder 1944: 207 (list of species of Latin America); Contreras 1951: 222 (cited of Colombia).
Dichotomius protectus : Roze 1955: 44 (cited for Venezuela); Medina et al. 2001: 138 (cited for Colombia); Hamel-Leigue et al. 2006: 15 (cited for Bolivia); Sarmiento-Garcés and Amat-García 2009: 290 (diagnosis), 295 (characters in key); Carvajal et al. 2011: 320–321 (cited for Ecuador); Krajcik 2012: 92 (complete list of species); Sarmiento-Garcés and Amat-García 2014: 51 (characters in key), 64 (diagnosis); Ratcliffe et al. 2015: 197 (cited for Peru).
Dichotomius (Dichotomius) protectus : Chamorro et al. 2018: 94 (cited for Ecuador).

####### Type specimens.

*Pinotusprotectus* Harold, 1867. Four syntypes examined deposited at the MZUSP. Lectotype to be designated in a future work on this species group.

**Syntype** (♂): “Loja / Oscordill. / Sabanilla / F. Ohs. 30.9.05 [p]”, “forma a. [hw]”, “Pinotus ♂ / protectus Har. / Lüd. det. 22 [hw]”, “17200 [p]”, “COTIPO [p, pink label, black margin]”.

**Syntype** (♂): “Loja / Oscordill. / Sabanilla / F. Ohs. 30.9.05 [p]”, “forma a. [hw]”, “Pinotus ♂ / protectus Har. / Lüd. det. 25 [hw]”, “17201 [p]”, “COTIPO [p, pink label, black margin]”.

**Syntype** (♂): “O. ECUADOR / Canelos / F. Ohs. 23.12.05 [p]”, “Pinotus ♂ / protectus Har. / Lüd. det. 25 [hw]”, “Forma a”, “17202 [p]”, “COTIPO [p, pink label, black margin]”.

**Syntype** (♀): “Loja / Oscordill. / Sabanilla / F. Ohs. 30.9.05 [p]”, “forma b. [hw]”, “Pinotus ♀ / protectus H. / Lüd. det. 22 [hw]”, “17203 [p]”, “COTIPO [p, pink label, black margin]”.

####### Distribution.

Bolivia, Colombia, Ecuador, Peru, and Venezuela.

####### Records examined.

LOJA: Sabanilla [= El Tambo, ZAMORA CHINCHIPE] (3 specimens MZUSP). MORONA SANTIAGO: Chiguinda Río Blanco, 1730 m (2 specimens MUTPL); Macas, 1000 m (1 specimen CEMT). NAPO: Quebrada Granadillas Bosque Protector La Cascada, 1300 m, Parque Nacional Sumaco (1 specimen MUTPL); Pacto Sumaco, 1620 m (1 specimen MUTPL); Las Palmas (1 specimen MUTPL); Punte Río Quijos, 1400 m, Parque Nacional Sumaco (3 specimens MUTPL); Puente Río El Salado-Río Quijos, 1280 m, Parque Nacional Sumaco (1 specimen MUTPL); Santa Rosa (1 specimen MUTPL). PASTAZA: Canelos (1 specimen MZUSP), Puyo (1 specimen CEMT). SUCUMBÍOS: La Sofía, 1800 m (1 specimen MUTPL). TUNGURAHUA: 2 km N de Baños, 1800 m (1 specimen CEMT); 4.3 km de Río Negro, 1200 m (2 specimens MQCAZ); Baños, 2200 m (1 specimen CEMT); Baños, El Topo, 1590 m (44 specimens CEMT). ZAMORA CHINCHIPE: Cordillera la Curintza, 1790 m (12 specimens MECN); Chito Río Sangolas, 1540 m (2 specimens MUTPL); Chito Río San Francisco, 1800 m (1 specimen MUTPL); RVS El Zarza campamento las Peñas, Cordillera del Cóndor, conseción El Zarza, 1510 m (1 specimen MUTPL); RVS El Zarza campamento las Peñas, Cordillera del Cóndor conseción El Colibri, 1535 m (1 specimen MUTPL); Tundayme, campamento Mirador road to La Cara del Indio, 1670 m (1 specimen MUTPL); Tundayme campamento Mirador, La Mina, 1320 m (1 specimen MUTPL); Zurmi, Comunidad Miazi, 1380 m (1 specimen MUTPL; 1 specimen MEPN); Zurmi, Pachikuntza, 1685 m (1 specimen MUTPL; 1 specimen MEPN); road to Condor km 38, 1800 m (1 specimen CEMT).

####### Literature records.

LOJA: Sabanilla [= El Tambo, ZAMORA CHINCHIPE], 1900 m (Luederwaldt 1923: 10). PASTAZA: Canolas [= Canelos] (Luederwaldt 1923: 10).

####### Temporal data.

Collected in January, February, March, April, May, July, September, October, and November.

####### Remarks.

Inhabits the lower evergreen montane forests in the Amazonian range from 1300–1730 m a.s.l. In the Andean region, it was registered in the montane cloud forests from 1800–2200 m a.s.l. Collected manually and with pitfall traps baited with carrion and human feces.

###### Dichotomius (Dichotomius) provisorius

Taxon classificationAnimaliaColeopteraScarabaeidae

(Luederwaldt, 1925)

Plate 26D

Pinotus provisorius Luederwaldt, 1925: 67 (original description. Type locality: Ecuador).
Pinotus provisorius : Luederwaldt 1929: 37 (characters in key); Blackwelder 1944: 207 (list of species from Latin America).
Dichotomius provisorius : Carvajal et al. 2011: 320–321 (cited for Ecuador); Krajcik 2012: 92 (complete list of species).
Dichotomius (Dichotomius) provisorius : Chamorro et al. 2018: 94 (cited for Ecuador).

####### Type specimens.

*Pinotusprovisorius* Luederwaldt, 1925. Two syntypes examined deposited at the MZUSP. Lectotype to be designated in a future work on this species group.

**Syntype** (♀): “Loja [hw]”, “bengder. / F. Ohaus lg. [hw]”, “Pinotus ♀ / provisorius Lüd. / Lüeder. det. 25. [hw]”, “17246 [p]”, “♀ [hw]”, “COTIPO [p, black margin, purple label]”.

**Syntype** (♀): “O.ECUADOR / Macas Feyer [p]”, “Pinotus ♀ / provisorius Lüd. / Lüeder. det. 24. [hw]”, “17247 [p]”, “♀ [hw]”, “COTIPO [p, black margin, purple label]”.

####### Distribution.

Only known from Ecuador.

####### Records examined.

LOJA: without specific locality (1 specimen MZUSP). MORONA SANTIAGO: Macas: (1 specimen MZUSP).

####### Temporal data.

It is not known when this species was collected.

####### Remarks.

Inhabits the evergreen foothill forests of the Amazon region. The collection method is unknown.

###### Dichotomius (Dichotomius) quinquedens

Taxon classificationAnimaliaColeopteraScarabaeidae

(Felsche, 1910)

Plate 27A

Pinotus quinquedens Felsche, 1910: 343 (original description. Type locality: Ecuador, Los Llanos).
Pinotus quinquedens : Gillet 1911a: 62 (complete list of species); Luederwaldt 1929: 35 (characters in key); Blackwelder 1944: 208 (list of species from Latin America).
Dichotomius quinquedens : Medina et al. 2001: 138 (cited for Colombia); Carvajal et al. 2011: 320–321 (cited for Ecuador); Krajcik 2012: 92 (complete list of species); Sarmiento-Garcés and Amat-García 2014: 53 (characters in key), 64 (diagnosis).
Dichotomius (Dichotomius) quinquedens : Chamorro et al. 2018: 95 (cited for Ecuador).

####### Types specimens.

*Pinotusquinquedens* Felsche, 1910. Seven syntypes examined deposited at the SMTD. Lectotype to be designated in a future work on this species group.

**Syntype** (♂): “Los Lanos / Ecuador [p, black margin]”, “quinquendens- / tatus Felsche / Ecuador [hw, purpure margin]”, “Typus. [p, red label]”, “Coll. C. Felsche / Kauf 20, 1918 [p, green label, black margin]”.

**Syntype** (♂): “Los Lanos / Ecuador [p, black margin]”, “Coll. C. Felsche / Kauf 20, 1918 [p, green label, black margin]”.

**Syntype** (♀): “Los Lanos / Ecuador [p, red label, black margin]”, “Coll. C. Felsche / Kauf 20, 1918 [p, green label, black margin]”.

**Syntype** (♂): “Los Lanos / Ecuador [p, black margin]”, “Coll. C. Felsche / Kauf 20, 1918 [p, green label, black margin]”.

**Syntype** (♂): “Los Lanos / Ecuador [p, black margin]”, “Coll. C. Felsche / Kauf 20, 1918 [p, green label, black margin]”.

**Syntype** (♂): “Los Lanos / Ecuador [p, black margin]”, “Coll. C. Felsche / Kauf 20, 1918 [p, green label, black margin]”.

**Syntype** (♂): “Los Lanos / Ecuador [p, black margin]”, “Coll. C. Felsche / Kauf 20, 1918 [p, green label, black margin]”.

####### Distribution.

Colombia and Ecuador.

####### Records examined.

BOLIVAR: Bosque Protector Filo Palanga, 970 m (1 specimen MUTPL). CAÑAR: Cochancay, 1000 m (4 specimens CEMT); Javín, 850–1400 m (4 specimens CEMT); Joyapal (1 specimen MQCAZ). COTOPAXI: 4 km de Guasaganda, 500 m (2 specimens MQCAZ); Bosque Integral Otonga, 1800 m (2 specimens CEMT; 7 specimens MQCAZ); Los Libres, 2015 m (1 specimen MQCAZ); San Francisco de Las Pampa, 1500 m (7 specimens CEMT). EL ORO: Salvias Río Elvira, 1180 m (2 specimens MUTPL); Reserva Jocotoco, 1250 m (1 specimen MQCAZ). ESMERALDAS: El Placer, road Ibarra-San Lorenzo, 670 m (2 specimens MQCAZ); Estación Biológica Bilsa, 500 m (1 specimen MEPN); Playa de Oro, Estero Pote, 200 m (1 specimen CEMT). IMBABURA: Lita (2 specimens CEMT); Santa Cecilia (1 specimen QCAZ). MANABÍ: Puerto López, Guale, 200 m (1 specimen MUTPL); Puerto López, San Sebastian, 480 m (1 specimen MUTPL). PICHINCHA: Choconde San Miguel de los Bancos, 1200 m (4 specimens MUTPL); Chespi, Bellavista, 1380 (4 specimens MUTPL); Chiriboga, 1500 m (1 specimen MQCAZ); Estación Biológica Maquipucuna, 1650 (2 specimens MUTPL); Estación Biológica la Hesperia, 1200 m (2 specimens CEMT); Mindo, 1500 m (1 specimen MQCAZ); Palmeras (1 specimen MQCAZ); Pampas Argentinas (1 specimen MQCAZ). SANTO DOMINGO DE LOS TSÁCHILAS: Río Toachi (1 specimen MQCAZ); Santo Domingo (2 specimens CEMT). UNDETERMINED PROVINCE: Los Lanos [= Los Llanos] (7 specimens SMTD).

####### Temporal data.

Collected in all months except September.

####### Remarks.

Inhabits coastal lowland evergreen forests and coastal evergreen foothill forests from 200–1300 m a.s.l. In the Andean region, it was registered for the evergreen lower montane forests from 1500–1800 m a.s.l. Collected manually and with pitfall traps baited with human feces.

###### Dichotomius (Dichotomius) quinquelobatus

Taxon classificationAnimaliaColeopteraScarabaeidae

(Felsche, 1901)

Plate 27B

Pinotus quinquelobatus Felsche, 1901: 138 (original description. Type locality: Ecuador).
Pinotus quinquelobatus : Gillet 1911a: 62 (complete list of species); Luederwaldt 1929: 35 (characters in key), 40 (redescription); Blackwelder 1944: 208 (list of species from Latin America); Contreras 1951: 222 (cited for Colombia).
Dichotomius quinquelobatus : Medina et al. 2001: 138 (cited for Colombia); Sarmiento-Garcés and Amat-García 2009: 289 (redescription), 295 (characters in key): Carvajal et al. 2011: 320–321 (cited for Ecuador); Krajcik 2012: 92 (complete list of species); Sarmiento-Garcés and Amat-García 2014: 52 (characters in key), 59 (diagnosis); Ratcliffe et al. 2015: 197 (cited for Peru).
Dichotomius (Dichotomius) quinquelobatus : Chamorro et al. 2018: 79 (figure 2D), 95 (cited for Ecuador).

####### Types specimens.

*Pinotusquinquelobatus* Felsche, 1901. One syntype examined deposited at the SMTD. Lectotype to be designated in a future work on this species group.

**Syntype** (♂): “Ecuador / Baron [p]”, “quinquelobatus / Felsche / Ecuador. [hw, purple label]”, “Coll. C. Felsche / Kauf 20, 1918 [p, green label, black margin]”, “Staatl. Museum für / Tierkunde D esden [p]”, “TYPUS [p, red label, black margin]”.

####### Distribution.

Colombia and Ecuador.

####### Records examined.

MORONA SANTIAGO: Bosque Domoso, 1650 m (1 specimen CEMT; 7 specimens MQCAZ); Comunidad Angel Rouby sitio 8, Cordillera del Kutukú, 1300 m (5 specimens MQCAZ; 4 specimens MECN); Comunidad Untsuants sitio 6, Cordillera del Kutukú, 1100 m (3 specimens MQCAZ; 2 specimens MECN); Macas (2 specimens MQCAZ); Yapitia, 1075 m (1 specimen MQCAZ). NAPO: Cotundo Río Osayacu sector Shamato, 1070 m (2 specimens MUTPL); Río Hollín, 1100 m (1 specimen CEMT; 4 specimens MQCAZ). PASTAZA: Bosque Protector Oglán Alto, 810–950 m (2 specimens MUTPL); LLandia 17 km N del Puyo, 1000 m (5 specimens MQCAZ); Mera Estación Biológica de la UTE Pindo Mirador, 1000 m (1 specimen MUTPL). SUCUMBÍOS: Bosque Protector la Cascada Río Coca, 640 m (1 specimen MUTPL); La Bonita, 1800 m (1 specimen CEMT); Lumbaqui, 860 m (2 specimens MQCAZ); road La Alegría-La Bonita km 32 (3 specimens MECN). ZAMORA CHINCHIPE: Bombuscaru, Parque Nacional Podocarpus (4 specimens MECN); Tundayme campamento Mirador, La Mina, 1320 m (1 specimen MUTPL); road Namirez-Zamora km 1, 1000 m (3 specimens CEMT; 4 specimens MQCAZ); Zurmi, Comunidad La Wants, 1010 m (1 specimen MEPN); Zurmi, Cumunidad Miazi, 1380 m (1 specimen MEPN; 1 specimen MUTPL). UNDETERMINED PROVINCE: without specific locality (1 specimen SMTD).

####### Temporal data.

Collected every month of the year.

####### Remarks.

Inhabits the foothill evergreen forests and lower evergreen montane forests in the Amazonian range from 640–1800 m a.s.l. Collected with pitfall traps baited with carrion, human feces, and pig feces.

###### Dichotomius (Dichotomius) reclinatus

Taxon classificationAnimaliaColeopteraScarabaeidae

(Felsche, 1901)

Plate 27C

Pinotus reclinatus Felsche, 1901: 135 (original description. Type locality: Columbia, Cachabé).
Pinotus reclinatus : Gillet 1911a: 62 (complete list of species); Luederwaldt 1929: 22 (characters in key); Blackwelder 1944: 208 (list of species from Latin America); Pereira 1954b: 464 (characters in key).
Dichotomius reclinatus : Vulcano and Pereira 1967: 584 (characters in key); Medina et al. 2001: 138 (cited for Colombia); Krajcik 2012: 92 (complete list of species); Sarmiento-Garcés and Amat-García 2014: 33 (diagnosis).
Dichotomius (Dichotomius) reclinatus : Chamorro et al. 2018: 95 (cited for Ecuador).

####### Types specimens.

*Pinotusreclinatus* Felsche, 1901. The holotype is deposited at the SMTD. Locality: Cachabé. Examined.

**Holotype** (♀): “Cachabé / low c. XII. 96. / (Rosenberg). [p, black margin]” , “Typus. [p, red label]”, “Coll. C. Felsche / Kauf 20, 1918 [p, green label, black margin]”, “reclinatus / Felsche / Colombia [hw, purpure margin]”, “HOLOTYPE ♀ [p, black margin]”.

####### Distribution.

Colombia and Ecuador.

####### Records examined.

ESMERALDAS: Playa de Oro, Pote (1 specimen MQCAZ). COTOPAXI: 4 km de Guasaganda, 300 m (1 specimen MQCAZ). GUAYAS: Bucay, 300 m (1 specimen MZSP). IMBABURA: Lita, 680 m (2 specimens CEMT); Junin (1 specimen MUTPL). LOS RIOS: 47 km S de Santo Domingo, 250 m, Río Palenque Station (1 specimen CMNC). PICHINCHA: Chiriboga, 1800 m (2 specimens CEMT); Choconde, 1200 m, San Miguel de los Bancos (1 specimen MUTPL); Estr. Chiriboga, 1300 m (1 specimen CEMT). SANTO DOMINGO DE LOS TSÁCHILAS: 16 K m SE Santo Domingo, 680 m, Tinalandia (1 specimen CMNC).

####### Temporal data.

Collected in February, March, June, July, October, November, and December.

####### Remarks.

Inhabits coastal lowland evergreen forests and coastal evergreen foothill forests from 250–1300 m a.s.l. Collected with pitfall traps baited with human feces.

###### Dichotomius (Dichotomius) robustus

Taxon classificationAnimaliaColeopteraScarabaeidae

(Luederwaldt, 1935)

Plate 27D

Pinotus (s. str.) robustus Luederwaldt, 1935: 337 (original description. Type locality: British Guiana, Essequibo R., Moraballi Creek).
Pinotus robustus : Blackwelder 1944: 208 (list of species from Latin America).
Dichotomius robustus : Vulcano and Pereira 1967: 584 (characters in key); Medina et al. 2001: 138 (cited for Colombia); Sarmiento-Garcés and Amat-García 2009: 292 (diagnosis), 295 (characters in key); Krajcik 2012: 92 (complete list of species); Sarmiento-Garcés and Amat-García 2014: 75 (characters in key), 78 (diagnosis); Ratcliffe et al. 2015: 197 (cited for Peru).
Dichotomius (Dichotomius) robustus : Chamorro et al. 2018: 95 (cited for Ecuador).

####### Types specimens.

Pinotus(s. str.)robustus Luederwaldt, 1935. Five syntypes examined, deposited at the NHML and MNHN. Lectotype to be designated in a future work on this species group.

####### Distribution.

Colombia, Guyana, Ecuador, and Peru.

####### Records examined.

ORELLANA: Estación de Biodiversidad Tiputini campamento, 220 m, Parque Nacional Yasuní (1 specimen MUTPL); Ines Arango road Tiwino-río Shiripuno, 250 m (1 specimen MUTPL). SUCUMBÍOS: Parahuaco, 290 m (1 specimen CEMT); Sansahuari, Pozo Singue, 285 m (1 specimen CEMT).

####### Temporal data.

Collected in January, April, and July.

####### Remarks.

Inhabits the lowland evergreen forests of the Amazon region from 220–290 m a.s.l. Collected manually and with pitfall traps baited with human feces.

###### Dichotomius (Dichotomius) satanasangustus

Taxon classificationAnimaliaColeopteraScarabaeidae

(Luederwaldt, 1923)

Plate 28A

Pinotus satanas var. angustus Luederwaldt, 1923: 10 (original description. Type locality: Ecuador Sarayacu; Ost-Kordillere, Sued-Ecuador [= eastern Cordillera, southern Ecuador], 300 m; Canolos [= Canelos] Ost-Ecuador [= eastern Cordillera, Ecuador], 700 m; Macas Ost-Ecuador [= eastern Cordillera, Ecuador]; Loja-Sabanilla Ost-Ecuador [= eastern Cordillera, Ecuador]; Bannos-Mirador [= Baños-Mirador] Ost-Ecuador [= eastern Cordillera, Ecuador], 14–1600 m).
Pinotus satanas var. angustus : Luederwaldt 1929: 36 (characters in key).
Pinotus angustus : Blackwelder 1944: 206 (list of species from Latin America); Carvajal et al. 2011: 320–321 (cited for Ecuador).
Dichotomius (Dichotomius) satanasangustus : Chamorro et al. 2018: 95 (cited for Ecuador).

####### Type specimens.

Pinotussatanasvar.angustus Luederwaldt, 1923. Two syntypes examined deposited at the MZUSP. Lectotype to be designated in a future work on this species group.

**Syntype** (♂): “O.ECUADOR / Canelos / F. Ohs. 23. 12. 05 [p]”, “COTIPO [p, black margin, red label]”, “Pinotus ♂ / angustus Lüd. / Lüd. det. 22. [hw]”, “17228 [p]”.

**Syntype** (♂): “Sud-Ecuador, / Ostcordill 3000 m. / F. Ohaus 19-x- [hw]”, “17226 [p]”, “COTIPO [p, black margin, red label]”.

####### Distribution.

Colombia and Ecuador.

####### Records examined.

LOJA: without specific locality (1 specimen MZUSP). NAPO: Archidona (1 specimen CEMT); Cabañas San Isidro, 2 km NW de Cosanga, 2150 m (2 specimens CEMT); Cosanga Yanayacu Biost, 2150 m (3 specimens MECN); Río Hollín, 1100 m (1 specimen CEMT). PASTAZA: Canelos (1 specimen MZUSP). TUNGURAHUA: Baños El Topo, 1590 m (24 specimens CEMT). SUCUMBÍOS: La Sofía, 1800 m (2 specimens MUTPL); Sebundoy, 2200 m (2 specimens MECN). ZAMORA CHINCHIPE: Bombuscaru, Parque Nacional Podocarpus, 1150 m (2 specimens MECN); Chito Río Sangolas, 1800 m (4 specimens MUTPL); San Andres, 1850 m (4 specimens CEMT). UNDETERMINED PROVINCE: Ostcordill [= without specific locality], 3000 m (1 specimen MZUSP).

####### Literature records.

LOJA: Sabanilla [= El Tambo, ZAMORA CHINCHIPE] (Luederwaldt 1923: 11). MORONA SANTIAGO: Macas: (Luederwaldt 1923: 11). PASTAZA: Canolos [= Canelos], 700 m (Luederwaldt 1923: 11); Sarayacu (Luederwaldt 1923: 11). TUNGURAHUA: Bannos-Mirador [= Baños Mirador], 1400–1600 m. (Luederwaldt 1923: 11). UNDETERMINED PROVINCE: Ost-Kordillere, Sued-Ecuador, 3000 m (Luederwaldt 1923: 11).

####### Temporal data.

Collected in January, February, May, July, October, September, November, and December.

####### Remarks.

Inhabits the evergreen foothill forests of the Amazon region from 700–1150 m a.s.l. In the Andean region, it was recorded in the evergreen montane forests and montane cloud forests from 1600–2200 m a.s.l. Collected with pitfall traps baited with human feces.

##### Subgenus Dichotomius (Luederwaldtinia) Martínez, 1951

Dichotomius (Luederwaldtinia) Martínez, 1951b: 140 (cited as new name subgeneric. Designation type species: *Coprisnisus* Olivier, 1789); Martínez 1959: 88 (list of species from Argentina); Halffter and Edmonds 1982: 137 (cited as subgenus of *Dichotomius* Hope, 1838); Vaz-de-Mello 2000: 193 (list of species from Brazil); Sarmiento-Garcés and Amat-García 2009: 292 (redescription); Vaz-de-Mello et al. 2011: 20 (characters in key); Krajcik 2012: 91 (cited as synonym of *Dichotomius* Hope, 1838); Boilly and Vaz-de-Mello 2013: 109 (characters in key); Nunes and Vaz-de-Mello, 2013: 418 (characters in key for group of species); Sarmiento-Garcés and Amat-García 2014: 24 (characters in key), 102 (redescription); Chamorro et al. 2018: 77 (characters in key), 95 (list of species of Ecuador).

###### Dichotomius (Luederwaldtinia) fortepunctatus

Taxon classificationAnimaliaColeopteraScarabaeidae

(Luederwaldt, 1923), revalidated name

Plates 28B , 57C–D

Pinotus fortepunctatus Luederwaldt, 1923: 4 (original description. Type locality: Bucay, west- Ecuador 300 m).
Pinotus fortepunctatus : Luederwaldt 1929: 76 (characters in key); Blackwelder 1944: 207 (list of species of Latin America); Pereira 1947: 318 (characters in key), 320 (redescription); Pereira 1953: 387 (synonym of Dichotomiusglobulus (Felsche, 1901), comment).
Dichotomius fortepunctatus : Carvajal et al. 2011: 320 (cited for Ecuador).
Dichotomius (Dichotomius) fortepunctatus : Chamorro et al. 2018: 90 (figure 13A), 95 (cited for Ecuador).
Pinotus fortepunctatus var. catenatus Luederwaldt, 1931b: 300 (original description); Blackwelder 1944: 207 (list of species of Latin America); Pereira 1947: 318 (characters in key), 322 (redescription).
Pinotus globulus Felsche, 1901: 141 (original description. Type locality: Amazonas).
Pinotus globulus : Blackwelder 1944: 208 (list of species from Latin America); Pereira 1947: 316 (characters in key), 319 (redescription, comment); Pereira 1953: 387 (comment).
Dichotomius globulus : Vulcano and Pereira 1967: 586 (characters in key, cited for Ecuador); Medina et al. 2001: 138 (cited for Colombia); Carvajal et al. 2011: 320–321 (cited for Ecuador); Krajcik 2012: 92 (complete list of species); Sarmiento-Garcés and Amat-García 2014: 97 (characters in key), 98 (diagnosis).

####### Type specimens.

*Pinotusfortepunctatus* Luederwaldt, 1923. The lectotype (♂) (here designated) and one paralectotype are deposited at the MZUSP. Locality: Bucay, Equador, examined.

**Lectotype (here designated)** (♂): “Bucay 200 m / F. Ohs. II. 05 [p]”, “Equador / Bucay / II. 90 5 [hw]”, “Pinotus ♀ / fortepuntatus Lüd. i. Lit. [hw]”, “♀ [p]”, “COTIPO [p, pink label, black margin]”, “17741 [p]”, “LECTOTYPE ♂ / Pinotus / fortepunctatus / Luederwaldt, 1923 / des. F.Z. Vaz-de-Mello. 2018 [p and hw, red label, black margin]”.

**Paralectotype** (♂): “Equador / Bucay / 12. VI. 905 [hw]”, “COTIPO [p, pink label, black margin]”, “Pinotus ♂ / fortepunctatus / Lüd. i. Lit. [hw]”, “17741 [p]”, “PARALECTOTYPE ♂ / Pinotus / fortepunctatus / Luederwaldt, 1923 / des. F.Z. Vaz-de-Mello. 2018 [p and hw, yellow label, black margin]”.

*Pinotusglobulus* Felsche, 1901. The lectotype (♂) (here designated) and six paralectotypes are deposited at the SMTD. Locality: Iquitus. Examined.

**Lectotype (here designated)** (♂): “Iquitos [p, green label]”, “Coll. C. Felsche / Kauf 21 1918 [p, green lable, black margin]”, “Staatl. Museum für / Tierkunde Dresden [p]”, “Typus [p, red label]”, “globulus / Felsche [illegible] [hw, purple margin]”, “LECTOTYPE ♂ / Pinotus / globulus /Felsche / des. F.Z.Vaz-de-Mello, 2014 [p and hw, red label, black margin]”.

**Paralectotype** (♀): “Iquitos [p, green label]”, “Coll. C. Felsche / Kauf 20 1918 [p, green lable, black margin]”, “Staatl. Museum für / Tierkunde Dresden [p]”, “PARALECTOTYPE / Pinotus ♀ / globulus / Felche / des. F.Z. Vaz-de-Mello. 2014 [p and hw, yellow label, black margin]”.

**Paralectotype** (♀): “Manaos [p, green label]”, “Coll. C. Felsche / Kauf 20 1918 [p, green lable, black margin]”, “Staatl. Museum für / Tierkunde Dresden [p]”, “PARALECTOTYPE / Pinotus ♀ / globulus / Felche / des. F.Z. Vaz-de-Mello. 2014 [p and hw, yellow label, black margin]”.

**Paralectotype** (♀): “Rio Cachiyacu / Iquitos / Stuart. 93 [p,]”, “Coll. C. Felsche / Kauf 20 1918 [p, green lable, black margin]”, “Staatl. Museum für / Tierkunde Dresden [p]”, “PARALECTOTYPE / Pinotus ♀ / globulus / Felche / des. F.Z. Vaz-de-Mello. 2014 [p and hw, yellow label, black margin]”.

**Paralectotype** (♀): “Iquitos [p, green label]”, “Coll. C. Felsche / Kauf 20 1918 [p, green lable, black margin]”, “Staatl. Museum für / Tierkunde Dresden [p]”, “PARALECTOTYPE / Pinotus ♀ / globulus / Felche / des. F.Z. Vaz-de-Mello. 2014 [p and hw, yellow label, black margin]”.

####### Distribution.

Colombia, Ecuador, and Peru.

####### Records examined.

CARCHI: Tobar Donoso, 300 m (7 specimens MECN). COTOPAXI: Guasaganda km 4, 500 m (3 specimens CEMT; 4 specimens MQCAZ). ESMERALDAS: Colón del Onzole (4 specimens MQCAZ; 6 specimens MECN); Charco Vicente (6 specimens MQCAZ; 5 specimens MECN); Gualpi (2 specimens MECN); Gualpi, El Pajonal (11 specimens MECN); El Progreso (2 specimens MECN); Salto del Bravo (7 specimens MQCAZ; 12 specimens MECN). GUAYAS: Bucay (2 specimens MZUSP). IMBABURA: El Chontal, El Cauchero, 900 m (1 specimen MUTPL). LOS RÍOS: 47 km S. Sto Domingo, Río Palenque Station, 250 m (4 specimens CEMT; 3 specimens MQCAZ); Río Palenque Station (34 specimens CEMT). PICHINCHA: Bosque Protector Milpe-Río Pachijal, 1200 m (1 specimen MUTPL); El Encuentro, 620 m, San Miguel de los Bancos (1 specimen MUTPL); La Florida (1 specimen MQCAZ); Guayabilla, 520 m, Río Guayllabamba Manduriacus; Mindo; Pedro Vicente Maldonado, 600 m (1 specimen MUTPL); Llurimaguas, 290 m, Río Guayllabamba Pedro Vicente Maldonado (1 specimen MUTPL); Tortugo, 450 m, Río Guayllabamba Pedro Vicente Maldonado (1 specimen MUTPL); San Roque, 580 m, Río Guayllabamba Pedro Vicente Maldonado (1 specimen MUTPL). SANTO DOMINGO DE LOS TSÁCHILAS: Hda Pupusa (1 specimen MQCAZ); Río Silanche (1 specimen MQCAZ); Santo Domingo, Puerto Limón, 395 m (2 specimens MUTPL).

####### Literature records.

GUAYAS: Bucay (Luederwaldt 1923: 4).

####### Temporal data.

Collected in all months except January.

####### Remarks.

Inhabits coastal lowland evergreen forests and coastal evergreen foothill forests from 290–1200 m a.s.l. Collected using canopy fogging methods and pitfall traps baited with human feces.

Luederwaldt (1923) described *Pinotusfortepuctatus* (original designation) for Equador [= Ecuador, Bucay west]. Subsequently, Pereira (1953) considered this species as a synonym of *Dichotomiusglobulus* [= *Pinotusglobulus* original designation, described by Felsche in 1901, with type locality Amazonas]; according to Pereira, the two species have the same morphological characteristics. However, upon examining the external morphology (specifically, the depressions of the pronotal disc and elytral microsculpture) of the type specimens, *P.fortepuctatus* (lectotype ♂ here designated, deposited at the MZUSP, Plate 57C) and *P.globulus* (lectotype ♂ here designated, deposited at the SMTD, Plate 57D), we could confirm they belong to distinct species. Therefore, maintaining the specific name originally proposed by Luederwaldt, we elevate it to species level under the following status: Dichotomius (Luederwaltinia) fortepunctatus (Luederwaldt, 1923) revalidated name. Two lectotypes (♂, with localities Bucay and Iquitos) are here designated and illustrated (Plate 57C, D).

###### Dichotomius (Luederwaldtinia) hempeli

Taxon classificationAnimaliaColeopteraScarabaeidae

(Pereira, 1942)

Plate 28C

Dichotomius hempeli Pereira, 1942: 38 (original description. Type locality: Equador, Loja EW, Piscobamba).
Dichotomius hempeli : Martínez 1947: 112 (cited for Ecuador); Carvajal et al. 2011: 320–321 (cited for Ecuador); Krajcik 2012: 92 (complete list of species).
Dichotomius (Luederwaldtinia) hempeli : Chamorro et al. 2018: 95 (cited for Ecuador).

####### Type specimens.

*Dichotomiushempeli* Pereira, 1942. The holotype (♀) is deposited at the MZUSP (see Pereira 1942: 38). Locality: Loja, Piscobamba, examined.

**Holotype** (♀): “S. ECUADOR / Piscobamba / M. Watt [p]”, “TIPO [p, red label, black margin]”, “Pinotus / Hempeli / Lüd. / Lüd. det. 23 [hw]”, “♀ [hw]”, “Pinotus ♀ / hempeli / sp. n. / P. Pereira det. 942 [p and hw, black margin]”, “17464 [p]”.

####### Distribution.

Only known from Ecuador.

####### Records examined.

LOJA: Piscobamba (1 specimen MZUSP).

####### Temporal data.

It is not known when this species was collected.

####### Remarks.

Inhabits the montane cloud forests in the Andean region. The collection method is unknown.

###### Dichotomius (Luederwaldtinia) problematicus

Taxon classificationAnimaliaColeopteraScarabaeidae

(Luederwaldt, 1923)

Plate 28D

Pinotus problematicus Luederwaldt, 1923: 7 (original description. Type locality: Franz Guiana [= French Guiana], Peru, Loja Sued-Ecuador, Piscobamba Sued-Ecuador, Guayaquil West-Ecuador).
Pinotus problematicus : Luederwaldt 1929: 66 (characters in key); Balthasar 1941: 349 (cited for Peru); Balthasar 1951: 335 (cited for Peru).
Dichotomius problematicus : Medina et al. 2001: 138 (cited for Colombia); Krajcik 2012: 92 (complete list of species); Sarmiento-Garcés and Amat-García 2014: 111 (comment Table 2); Ratcliffe et al. 2015: 197 (cited for Peru).
Dichotomius (Luederwaldtinia) problematicus : Chamorro et al. 2018: 95 (cited for Ecuador).
Dichotomius (Luederwaldtinia) problematicusvar.problematicus : Carvajal et al. 2011: 320–321 (cited for Ecuador).
Dichotomius problematicus var. planus Luederwaldt, 1923: 9 (original description); Luederwaldt 1929: 66 (characters in key); Carvajal et al. 2011: 320–321 (cited for Ecuador).

####### Type specimens.

*Pinotusproblematicus* Luederwaldt, 1923. Twelve syntypes examined deposited at the MZUSP. Lectotype to be designated in a future work on this species group.

**Syntype** (♂): “Loja / Oscordill. / Sabanilla / F. Ohs. 29. 9. 05 [p]”, “Pinotus / problemat. Lüd. / forma b. / Lüd. det. 23 [hw]”, “Pinotus ♂ / inachus Er. / var. problemat. Lueder. / Lueder. det. 28. [hw]”, “17543 [p]”, “COTIPO [p, pink label, black margin]”.

**Syntype** (♂): “ECUADOR / Sigiro E. W. [p]”, “Pinotus ♂ / problemat. Lüd. / forma a. / Lüd. det. 23. [hw]”, “Pinotus ♂ / inachus Er. / forma a. / Lueder. det. 28. [hw]”, “17532 [p]”, “COTIPO [p, pink label, black margin]”.

**Syntype** (♂): “ECUADOR / Arenal E. W. [p]”, “Pinotus ♂ / problemat. Lüd. / forma a. / Lüd. det. 23. [hw]”, “Pinotus ♂ / inachus Er. / forma a. / Lueder. det. 28. [hw]”, “17533 [p]”, “COTIPO [p, pink label, black margin]”.

**Syntype** (♀): “ECUADOR / Arenal E. W. [p]”, “Pinotus ♀ / problemat. Lüd. ? / forma a. / Lüd. det. 23. [hw]”, “Pinotus ♀ / inachus Er. / Lueder. det. 28. [hw]”, “17534 [p]”, “COTIPO [p, pink label, black margin]”.

**Syntype** (♀): “S. Ecuador / Piscobamba / M. Witt [p]”, “Pinotus ♀ / inachus Er. / Lueder. det. 28. [hw]”, “Pinotus ♀ / problemat. Lüd. / forma c. / Lüd. det. 23. [hw]”, “17535 [p]”, “COTIPO [p, pink label, black margin]”.

**Syntype** (♀): “S. Ecuador / Piscobamba / M. Witt [p]”, “Pinotus ♀ / problemat Lüd / forma c / Lüd. det. 23. [hw]”, “Pinotus ♀ / inachus Er. / Lueder. det. 28. [hw]”, “17536 [p]”, “COTIPO [p, pink label, black margin]”.

**Syntype** (♀): “ECUADOR / Sigiro E. W. [p]”, “Pinotus ♀ / problemat. Lüd. / forma a. / Lüd. det. 23. [hw]”, “Pinotus ♀ / inachus Er. / forma a. / Lueder. det. 28. [hw]”, “17537 [p]”, “COTIPO [p, pink label, black margin]”.

**Syntype** (♀): “Ecuador / Loja E. W [p]”, “Kein Penis. [hw]”, “Pinotus ♀ / problemat. Lüd. / forma a. / Lüd. det. 23. [hw]”, “Pinotus ♀ / inachus Er. / forma a. / Lueder. det. 28. [hw]”, “17538 [p]”, “COTIPO [p, pink label, black margin]”.

**Syntype** (♀): “Ecuador / Loja E. W. [p]”, “Pinotus ♀ / problemat. Lüd. / forma c. / Lüd. det. 23. [hw]”, “Pinotus ♀ / inachus Er. / Lueder. det. 28. [hw]”, “17539 [p]”, “COTIPO [p, pink label, black margin]”.

**Syntype** (♀): “S. Ecuador / Loja / F. Ohs. 8. 10. 05 [p]”, “Pinotus ♀ / problemat. Lüd. / forma c. / Lüd. det. 23. [hw]”, “Pinotus ♀ / inachus Er. / Lueder. det. 28. [hw]”, “17540 [p]”, “COTIPO [p, pink label, black margin]”.

**Syntype** (♂): “S. Ecuador / Piscobamba / M. Witt [p]”, “Pinotus sp / nr inachus Har [hw]” , “Pinotus ♂ / problemat. Lüd. / forma c. / Lüd. det. 23. [hw]”, “Pinotus ♂ / inachus Er. / Lueder. det. 28. [hw]”, “17541 [p]”, “COTIPO [p, pink label, black margin]”.

**Syntype** (♀): “Loja [illegible, p]”, “Kein Penis ! [hw]”, “Pinotus ♀ / problemat. Lüd. / forma b. / Lüd. det. 23. [hw]”, “Pinotus ♀ / inachus var. pro- / blemat Lueder. / Lueder. det. 28. [hw]”, “♀ [hw]”, “17544 [p]”, “COTIPO [p, pink label, black margin]”.

####### Distribution.

Ecuador and Peru.

####### Records examined.

LOJA: Comunidades Río Yangana, 1500 m (2 specimens MQCAZ); El Arenal (2 specimens MZUSP); Los Malacatos (1 specimen MQCAZ); Piscobamba (3 specimens MZUSP); San Pedro de Vilcabamba (1 specimen MQCAZ); Sabanilla [= El Tambo ZAMORA CHINCHIPE] (1 specimen MZUSP); Sigiro (2 specimens MZUSP); without specific locality (4 specimens MZUSP). MORONA SANTIAGO: Bosque Domoso (5 specimens CEMT; 2 specimens MQCAZ); road Mendez-Paute km 8 (1 specimen CEMT). NAPO: Rio Hollín, 1500 m (2 specimens MQCAZ). PASTAZA: road Triunfo-Arajuno (3 specimens CEMT; 4 specimens MQCAZ). SUCUMBÍOS: Gonzalo Pizarro, Simón Bolivar, 1200 m (3 specimens MECN). TUNGURAHUA: Baños, El Topo, 1590 m (56 specimens CEMT). ZAMORA CHINCHIPE: km 1 road Cumbaritza-Gualaquiza, 1100 m (12 specimens CEMT; 13 specimens MQCAZ); km 1 road Namirez-Zamora, 1000 m (8 specimens CEMT; 13 specimens MQCAZ); km 4 road Zumbi-Yantzaza (5 specimens CEMT; 7 specimens MQCAZ).

####### Literature records.

GUAYAS: Guayaquil, west Ecuador (Luederwaldt 1923: 7). LOJA: without specific locality, Sued-Ecuador (Luederwaldt 1923: 7); Piscobamba, 2200 m (Luederwaldt 1923: 7).

####### Temporal data.

Collected in January, February, March, June, July, August, October, November, and December.

####### Remarks.

Inhabits the foothill evergreen forests and lower evergreen montane forests in the Amazonian range from 1200–1590 a.s.l. In the Andean region, it was registered for the montane cloud forests from 1800–2200 m a.s.l. Collected with pitfall traps baited with human feces.

Luederwaldt (1923) cited the Franz Guyana [= French Guiana] and Guayaquil, west Ecuador (towards the Pacific coast) as the type localities for this species. It is possible that this species is also found in the Amazon foothill forests in southern Ecuador and northern Peru.

###### Dichotomius (Luederwaldtinia) simplicicornis

Taxon classificationAnimaliaColeopteraScarabaeidae

(Luederwaldt, 1935)

Plate 29A

Pinotus (Selenocopris) simplicicornis Luederwaldt, 1935: 340 (original description. Type locality: Peru).
Pinotus simplicicornis : Blackwelder 1944: 208 (list of species from Latin America); Krajcik 2012: 92 (complete list of species).
Dichotomius simplicicornis : Ratcliffe et al. 2015: 197 (cited for Peru).
Dichotomius (Luederwaldtinia) simplicicornis : Chamorro et al. 2018: 95 (cited for Ecuador).

####### Types specimens.

Pinotus (Selenocopris) simplicicornis Luederwaldt, 1935. The holotype (♂) is deposited at the NHML. Locality: Peru. Examined.

**Holotype** (♂): “Peru [hw]”, “34600 [hw]”, “Pinotus ♂ / simplicicornis n. sp / Lüeder. det. 34 [hw, black margin]”, “Fry Coll. / 1905-100. [p]”, “HOLOTYPE [p, red label, black margin]”.

####### Distribution.

Ecuador and Peru.

####### Records examined.

LOJA: Catamayo, Alamala, 1100 m (2 specimens CEMT). MORONA SANTIAGO: Nuevo Israel, Cordillera del Kutukú (1 specimen CEMT). NAPO: Pte. Río Salado-Río Quijos, Parque Nacional Sumaco (1 specimen CEMT); Quebrada Granadillas, Parque Nacional Sumaco, 1300 m (1 specimen CEMT). ZAMORA CHINCHIPE: Bombuscaro, Parque Nacional Podocarpus, 970 m (4 specimens MECN); Río Bombuscaro, Parque Nacional Podocarpus, 1145 m (7 specimens MEPN); Tundayme, Escombrera, 1225 m (1 specimen MUTPL); Zurmi Comunidad La Wants, 1000 m (2 specimens CEMT).

####### Temporal data.

Collected in January, March, May, August, September, and December.

####### Remarks.

Inhabits the foothill evergreen forests in the Amazonian range from 970–1225 m a.s.l. Collected manually and with pitfall traps baited with carrion and human feces. According to Kohlmann and Solís (1997) and Solís and Kohlmann (2012), the previous records from Costa Rica are erroneous.

##### Subgenus Dichotomius (Selenocopris) Burmeister, 1846

Dichotomius (Selenocopris) Burmeister, 1846: 87 (original description. Type species: *Coprisbicuspis* Germar, 1824, subsequently designation by Martínez 1951b: 141); Burmeister 1873b [= 1874]: 127 (cited as genus *Selenocopris* Burmeister, 1846 redescription); Gemminger and Harold 1869: 1009 (cited as synonym of *Pinotus* Erichson, 1847); Bruch 1911: 188 (list of species for Argentina, cited as genus *Selenocopris*, Burmeister, 1846); Gillet 1911a: 59 (cited as synonym of *Pinotus* Erichson, 1847); Lucas 1920: 589 (cited as synonym of *Pinotus* Erichson, 1847); Luederwaldt 1929: 12 (characters in key), 61 (redescription); Pessôa and Lane 1941: 461 (characters in key); Blackwelder 1944: 206 (cited as synonym *Pinotus* Erichson, 1847); Martínez 1951b: 139 (comment, restored as subgenus Selenocopris Burmeister, 1846) 141 (designation type species); Martínez 1959: 91 (cited as subgenus of *Dichotomius* Hope, 1838); Halffter and Edmonds 1982: 137 (cited as subgenus of *Dichotomius* Hope, 1838); Kohlmann and Solís 1997: 344 (cited as synonym of *Dichotomius* Hope, 1838); Vaz-de-Mello 2000: 193 (list of species from Brazil); Ratcliffe 2002: 15 (cited as synonym of *Dichotomius* Hope, 1838); Krajcik 2012: 91 (cited as synonym of *Dichotomius*Hope 1838); Boilly and Vaz-de-Mello 2013: 109 (characters in key); Sarmiento-Garcés and Amat-García 2014: 24 (characters in key), 102 (redescription); Chamorro et al. 2018: 77 (characters in key), 89 (figure 12H), 95 (list of species of Ecuador).

*Cephagonus* Luederwaldt, 1929: 12 (original description. Without type species); Pessôa and Lane 1941: 461 (characters in key); Blackwelder 1944: 206 (cited as synonym *Pinotus* Erichson, 1847); Martínez 1951b: 139 (synonym of *Selenocopris* Burmeister, 1846).

###### Dichotomius (Selenocopris) fonsecae

Taxon classificationAnimaliaColeopteraScarabaeidae

(Luederwaldt, 1926)

Plate 29B

Pinotus fonsecae Luederwaldt, 1926: 135 (original description. Type locality: Macas).
Pinotus fonsecae : Luederwaldt 1929: 110 (characters in key); Blackwelder 1944: 207 (list of species of Latin America).
Dichotomius fonsecae : Carvajal et al. 2011: 320–321 (cited for Ecuador); Krajcik 2012: 91 (complete list of species); Carvajal 2012: 197 (redescription).
Dichotomius (Selenocopris) fonsecae : Chamorro et al. 2018: 95 (cited for Ecuador).

####### Type specimens.

*Pinotusfonsecae* Luederwaldt, 1926. The holotype (♂) is deposited at the MZUSP. Locality: Macas, Ecuador, examined.

**Holotype** (♂): “O. ECUADOR / Macas Feyer [p]”, “TIPO [p, red label, black margin]”, “Pinotus ♂ / fonsecae Lüd / Lüd. det. 23 [hw]”, “25904 [p]”.

####### Distribution.

Only known from Ecuador.

####### Records examined.

MORONA SANTIAGO: Macas (1 specimen MZUSP); San Isidro, Cordillera de Domoso Alto, 1680 m (1 specimen MEPN). ZAMORA CHINCHIPE: Cordillera del Cóndor, Colibrí, 1445 m (1 specimen MEPN).

####### Literature records.

MORONA SANTIAGO: Macas (Luederwaldt 1926: 135).

####### Temporal data.

Collected in November and December.

####### Remarks.

Inhabits the lower evergreen montane forests in the Amazonian range from 1445–1680 m a.s.l. Collected manually and with pitfall traps baited with human feces.

#### Genus *Eurysternus* Dalman, 1824

*Eurysternus* Dalman, 1824: 8 (original description. Type species: *Eurysternusplanus* Dalman, 1854 subsequently designated by Jessop 1985: 1089).

*Eurysternus*: Latreille 1829: 535 (redescription); Castelnau 1840: 92 (redescription); Agassiz 1846: 436 (catalog); Lacordaire 1856: 106 (redescription); Gemminger and Harold 1869: 1023 (complete list of species); Gillet 1911a: 25 (catalog); Bruch 1911: 182 (list of species from Argentina); Lucas 1920: 289 (catalog, distribution); Paulian 1938: 232 (characters in key); Pessôa and Lane 1941: 406 (redescription); Blackwelder 1944: 197 (list of species from Latin America); Martínez 1959: 19 (list of species from Argentina); Halffter and Matthews 1966: 259 (catalog, distribution); Halffter and Halffter 1976: 47 (redescription); Howden and Young 1981: 11 (characters in key), 14 (redescription); Halffter and Edmonds 1982: 137 (catalog, distribution); Jessop 1985: 1089 (redescription); Martínez 1988a: 281 (characters in key, cited as subgenus Eurysternus); López-Guerrero and Halffter 2000: 244 (morphology); Medina and Lopera 2000: 301 (characters in key); Vaz-de-Mello 2000: 193 (list of species from Brazil); Medina et al. 2001: 135 (list of species from Colombia); Ratcliffe 2002: 11 (list of species from Panama); Morón 2003: 44 (list of species from Mexico); Huerta et al. 2003: 6 (biology); Hamel-Leigue et al. 2006: 16 (list of species from Bolivia); Génier 2009: 22 (revision); Camero 2010: 149 (distribution of records from Colombia); Vaz-de-Mello et al. 2011: 21 (characters in key); Carvajal et al. 2011: 111 (diagnosis), 314 (list of species from Ecuador); Krajcik 2012: 107 (complete list of species); Solís and Kohlmann 2012: 7 (list of species from Costa Rica); Boilly and Vaz-de-Mello 2013: 106 (characters in key); Chamorro et al. 2018: 73 (characters in key), 95 (list of species of Ecuador).

*Aeschrotes* Le Peletier de Saint-Fargeu & Audinet-Serville, 1828: 357 (original description. Type species: *Aeschrotesplanus* Dalman, 1824 = *Scarabaeuscaribaeus* Herbst, 1789 subsequently designated by Jessop 1985); Castelnau 1840: 92 (cited as synonym); Agassiz 1846: 26 (catalog); Lacordaire 1856: 106 (synonym of *Eurysternus* Dalman, 1824 see footnote 1); Gemminger and Harold 1869: 1023 (synonym of *Eurysternus* Dalman, 1824); Gillet 1911a: 25 (synonym of *Eurysternus* Dalman, 1824); Pessôa and Lane 1941: 406 (cited as synonym of *Eurysternus* Dalman, 1824); Blackwelder 1944: 197 (cited as synonym of *Eurysternus* Dalman, 1824); Martínez 1959: 19 (synonym of *Eurysternus* Dalman, 1824); Howden and Young 1981: 14 (synonym of *Eurysternus* Dalman, 1824); Jessop 1985: 1089 (synonym of *Eurysternus* Dalman, 1824); Ratcliffe 2002: 11 (synonym of *Eurysternus* Dalman, 1824); Génier 2009: 23 (synonym of *Eurysternus* Dalman, 1824); Solís and Kohlmann 2012: 7 (synonym of *Eurysternus* Dalman, 1824).

*Eurysternodes* Martínez, 1988a: 281 (original description. Type species: *Eurysternusvelutinus* Bates, 1887); Vaz-de-Mello 2000: 193 (list of species from Brazil, cited as subgenus); Génier 2009: 23 (synonym of *Eurysternus* Dalman, 1824); Solís and Kohlmann 2012: 7 (cited as synonym of *Eurysternus* Dalman).

*Pareurysternus* Martínez, 1988a: 282 (original description. Type species: Eurysternus (Pareurysternus) navajasi Martínez, 1988); Vaz-de-Mello 2000: 193 (list of species from Brazil); Génier 2009: 23 (synonym of *Eurysternus* Dalman, 1824); Solís and Kohlmann 2012: 7 (cited as synonym of *Eurysternus* Dalman, 1824); Krajcik 2012: 107 (cited as synonym of *Eurysternus* Dalman, 1824).

*Amartinezus* Ozdikmen, 2009: 143 (original description. Type species: *Eurysternusvelutinus* Bates, 1887); Génier 2009: 23 (synonym of *Eurysternus* Dalman); Carvajal et al. 2011: 110 (description, cited as *Amartinezus* nom. Nov); Solís and Kohlmann 2012: 7 (cited as synonym of *Eurysternus* Dalman).

##### Eurysternus atrosericus

Taxon classificationAnimaliaColeopteraScarabaeidae

Génier, 2009

Eurysternus atrosericus Génier, 2009: 86 (original description. Type locality: Brazil, Obidos = PA[RÁ]).
Eurysternus atrosericus : Camero 2010: 149 (characters in key), 153 (diagnosis); Krajcik 2012: 107 (complete list of species); Chamorro et al. 2018: 95 (cited for Ecuador).

###### Type specimens.

*Eurysternusatrosericus* Génier, 2009. The holotype (♂) is deposited at the MZUSP (see Génier 2009). Locality: Brazil, Obidos = PA[RÁ], not examined.

###### Distribution.

Brazil, Ecuador, Guyana, and Venezuela.

###### Literature records.

CHIMBORAZO: without specific locality (Génier 2009: 90). PICHINCHA: without specific locality (Génier 2009: 90).

###### Temporal data.

It is not known when this species was collected.

###### Remarks.

The natural history is unknown. According to Génier (2009), this species has been collected using pitfall traps baited with carrion and human feces.

##### Eurysternus caribaeus

Taxon classificationAnimaliaColeopteraScarabaeidae

(Herbst, 1789)

Plate 29C

Scarabaeus caribaeus Herbst, 1789: 300 (original description. Without type locality).
Eurysternus caribaeus : Gemminger and Harold 1869: 1023 (list, distribution); Harold 1880a: 13 (distribution); Gillet 1911a: 25 (catalog); Bruch 1911: 182 (cited for Argentina); Blackwelder 1944: 197 (list of species from Latin America); Guêrin 1953: 256 (diagnosis); Roze 1955: 41 (cited for Venezuela); Martínez 1959: 20 (cited for Argentina); Vulcano and Pereira 1967: 547 (characters in key); Halffter and Halffter 1976: 57 (redescription); Halffter et al. 1980: 600 (biology); Howden and Young 1981: 14 (characters in key), 17 (redescription); Jessop 1985: 1093 (characters in key), 1102 (distribution); Medina et al. 2001: 135 (cited for Colombia); Ratcliffe 2002: 11 (cited for Panama); Morón 2003: 45 (cited for Mexico); Huerta et al. 2003: 16 (biology); Hamel-Leigue et al. 2006: 16 (cited for Bolivia); Génier 2009: 215 (redescription), 291 (characters in key); Camero 2010: 150 (characters in key), 164 (diagnosis); Carvajal et al. 2011: 314–315 (cited for Ecuador); Krajcik 2012: 107 (complete list of species); Solís and Kohlmann 2012: 7 (cited for Costa Rica); Ratcliffe et al. 2015: 195 (cited for Peru); Chamorro et al. 2018: 95 (cited for Ecuador).
Eurysternus (Eurysternus) s. str.caribaeus : Vaz-de-Mello 2000: 193 (cited for Brazil).
Eurysternus planus Dalman, 1824: 10 (original description); Dejean 1837: 160 (catalog, cited as EurysternusPlanus Dej); Castelnau 1840: 92 (redescription); Gemminger and Harold 1869: 1023 (synonym of Eurysternuscaribaeus (Herbst, 1789), catalog); Gillet 1911a: 25 (cited as synonym of E.caribaeus Herbst); Bruch 1911: 182 (cited as synonym of E.caribaeus Herbst); Blackwelder 1944: 197 (cited as synonym of E.caribaeus Herbst); Martínez 1959: 20 (cited as synonym of E.caribeus Herbst); Jessop 1985: 1102 (cited as synonym of E.caribeus Herbst); Génier 2009: 214 (cited as synonym of E.caribeus Herbst); Camero 2010: 164 (cited as synonym of E.caribeus Herbst); Solís and Kohlmann 2012: 7 (cited as synonym of E.caribeus Herbst).
Eurysternus nebulosus Kirsch, 1871: 361 (original description); Harold, 1880a: 13 (synonym of Eurysternuscaribeus Herbst); Bates, 1887: 40 (comment, distribution); Gillet, 1911a: 25 (cited as synonym of Eurysternuscaribaeus Herbst); Blackwelder, 1944: 197 (cited as synonym of Eurysternuscaribaeus Herbst); Martínez, 1959: 20 (cited as synonym of Eurysternuscaribeus Herbst); Jessop, 1985: 1102 (cited as synonym of Eurysternuscaribeus Herbst); Génier, 2009: 214 (cited as synonym of Eurysternuscaribeus Herbst), Camero, 2010: 164 (cited as synonym of Eurysternuscaribeus Herbst); Solís and Kohlmann 2012: 7 (cited as synonym of Eurysternuscaribeus Herbst).
Eurysternus peruanus Harold, 1875d: 137 (original description); Balthasar 1941: 340 (cited for Peru); Blackwelder 1944: 197 (list of species from Latin America); Balthasar 1951: 325 (cited for Peru); Jessop 1985: 1102 (synonym of Eurysternuscaribeus Herbst); Génier 2009: 214 (cited as synonym of Eurysternuscaribeus Herbst); Camero 2010: 164 (cited as synonym of Eurysternuscaribeus Herbst); Solís and Kohlmann 2012: 7 (cited as synonym of E.caribeus Herbst).

###### Type specimens.

*Scarabaeuscaribaeus* Herbst, 1789. The neotype (♂) is deposited at the NMHU (see Génier 2009: 215). Locality: Cayene Ban[on], not examined.

*Eurysternusplanus* Dalman, 1824. The holotype (♂) is deposited at the NHRS (see Génier 2009: 215). Locality: Cayene, not examined.

*Eurysternusnebulosus* Kirsch, 1871. The lectotype (♂) is deposited at the SMTD (see Génier 2009: 216). Locality: Bogota, not examined.

*Eurysternusperuanus* Harold, 1875. The lectotype (♀) is deposited at the MNHN (see Génier 2009: 216). Locality: Peru, not examined.

###### Distribution.

Argentina, Belize, Bolivia, Brazil, Colombia, Costa Rica, Ecuador, Guatemala, French Guiana, Guyana, Honduras, Mexico, Nicaragua, Panama, Paraguay, Peru, Surinam, Trinidad, and Venezuela.

###### Records examined.

BOLIVAR: Bosque Protector Filo Palanga, 970 m (1 specimen MUTPL). CARCHI: El Goaltal Hacienda San Francisco, 1200 m (16 specimens MECN). ESMERALDAS: Palma Real (11 specimens MECN); Pote, Playa de Oro (9 specimens MECN); Playa de Oro (17 specimens MECN). IMBABURA: Lita, 680 m (3 specimens CEMT; 4 specimens MQCAZ); Río Getsemani, 600 m (3 specimens CEMT; 5 specimens MQCAZ). MANABÍ: Ayampe, 35 m (1 specimen MUTPL); Puerto López Comunidad Agua Blanca, 245 m (1 specimen MUTPL); Puerto López Cerro La Gotera, Parque Nacional Machalilla, 350 m (1 specimen MUTPL); Puerto López, Guale, 310 m (1 specimen MUTPL); Puerto López, Las Tunas, 200 m (1 specimen MUTPL). MORONA SANTIAGO: Comunidad Untsuants, 700 m, Cordillera del Kutukú (8 specimens MECN); Nuevo Israel, 1290 m, Cordillera Del Kutukú (2 specimens MUTPL). NAPO: Shiqui cerca al Tena, 480 m, Pungarayacu (1 specimen MQCAZ); Tena, 400–500 m (1 specimen CEMT; 3 specimens MQCAZ). ORELLANA: Bloque 31, Parque Nacional Yasuní, 200 m (5 specimens MECN); Cononaco, Bloque 16 YPF, 250 m (1 specimen MUTPL); Dayuma Campo Hormiguero, plataforma Hormiguero, 320 m (5 specimens MUTPL); Dayuma Campo Palanda, 255 m, Yuca 13 (1 specimen MGO-UC); Dayuma plataforma Ungurahua, 300 m (1 specimen MUTPL); El Dorado plataforma Guarango, 300 m (1 specimen MUTPL); Estación Biológica y Centro de Capacitación IAMOE, Rodrigo Borja (8 specimens CEMT; 6 specimens MECN; 11 specimens MQCAZ); Estación Científica Yasuní, PUCE, 250 m (48 specimens MQCAZ); Estación de Biodiversidad Tiputini, Parque Nacional Yasuní (2 specimens MUTPL); San Pedro del Lago, plataforma Copal, 310 m (1 specimen MUTPL); San Sebastián del Coca, 345 m, Comuna Guataraco Campo Pata (1 specimen MUTPL); San Sebastián del Coca, 345 m, Comuna Shamanal Campo Palo Azul (1 specimen MUTPL). PASTAZA: Bosque Protector Oglán Alto, 950 m (2 specimens MUTPL); Chuyayacu Oleoducto km 25, 200 m (1 specimen MUTPL); Estación Biológica Pindo Mirador UTE, 1000 m (3 specimens CEMT); Nuevo Israel, Cordillera del Kutukú (2 specimens MUTPL); Nuevo San José del Curaray, 245 m (1 specimen MUTPL); road Triunfo-Arajuno (2 specimens CEMT; 8 specimens MQCAZ). PICHINCHA: Curipoglio, 1820 m, Cerro San Cristobal (1 specimen MUTPL); Estación Biológica La Hesperia (5 specimens MUTPL); El Tigre Río Guayllabamba, Pedro Vicente Maldonado (1 specimen MUTPL); Llurimaguas, 290 m, Río Guayllabamba Pedro Vicente Maldonado (1 specimen MUTPL); San Roque, 580 m, Río Guayllabamba Pedro Vicente Maldonado (1 specimen MUTPL); Tortugo, 450 m, Río Guayllabamba Pedro Vicente Maldonado (1 specimen MUTPL); Guayabilla, 520 m, Río Guayllabamba Manduriacus (1 specimen MUTPL). SANTA ELENA: La Rinconada, 10 m (1 specimen MQCAZ); Olón, 50 m (9 specimens CEMT). SUCUMBÍOS: 6 km de Dureno, Precooperativa Los Vergeles, 290 m (12 specimens MGO-UC); Aucayacu Río El Eno, 275 m, 16 km de Lago Agrio (11 specimens MGO-UC); Bermejo plataforma ER-A road to Lumbaqui (1 specimen MUTPL); Gonzalo Pizarro Simón Bolivar 1200 m (3 specimens MECN); Shusufindi (2 specimens MECN); Tarapoa Campo Marian, plataforma Fanny 5, 260 m (1 specimen MUTPL); Tarapoa, Nuevo Manabí, 270 m (1 specimen MUTPL). TUNGURAHUA: Baños (3 specimens MQCAZ). ZAMORA CHINCHIPE: RVS El Zarza campamento las Peñas, Cordillera del Cóndor, conseción El Colibri, 1535 m (1 specimen MUTPL); Tundayme campamento Mirador, San Marcos, 900 m (2 specimens MUTPL); Tundayme campamento Mirador, Condor Mirador, 1465 m (1 specimen MUTPL); Tundayme, 800 m (1 specimen MUTPL); Zurmi Comunidad Miazi, 1380 m (1 specimen MEPN); Zurmi, Pachikuntza, 1685 m (1 specimen MEPN); Zurmi, Comunidad La Wants, 1010 m (1 specimen MUTPL, 1 specimen MEPN); Zurmi Las Orquideas Río Nangaritza, 870 m (1 specimen MEPN).

###### Literature records.

ESMERALDAS: Bilsa (Génier 2009: 225); Estación Forestal La Chiquita, 11 km SE de San Lorenzo, 5 m (Génier 2009: 225); Pote, Playa de Oro (Génier 2009: 225). GUAYAS [= SANTA ELENA]: 27 km S Puerto López, 76 km N de Santa Elena, 152 m (Génier 2009: 225). LOJA: Loja/Zamora, 1400 m (Génier 2009: 225). LOS RÍOS: 47 km S de Santo Domingo, 213 m (Génier 2009: 225); Estación Científica Río Palenque, 150–220 m (Génier 2009: 225); Estación Científica Río Palenque, 47 km S de Santo Domingo, 250 m (Génier 2009: 225); Estación Experimental Tropical Pichilingue Quevedo, 45 m (Génier 2009: 225). MANABÍ: 78 km NE Chone, 85 km WSW de Santo Domingo, 450 m (Génier 2009: 225); 90 km WSW de Santo Domingo, 73 km NE de Chone, 300 m (Génier 2009: 225). MORONA SANTIAGO: Bosque Domono, 1650 m (Génier 2009: 225); Untsuants sitio 1, 700 m (Génier 2009: 225); road Mendez-Paute km 8, 1250 m (Génier 2009: 225). NAPO: 12 km WSW Tena, 600 m (Génier 2009: 225); 5 km O Tena, 500 m (Génier 2009: 225); Estación Biológica Jatun Sacha, 450 m (Génier 2009: 225); Estación Biológica Jatun Sacha, 21 km E Puerto Napo, 400 m (Génier 2009: 225); Hacienda Aragón, Sierra Azul, 2300 m (Génier 2009: 226); km 11.1 road Sarayacu-Loreto, 1200 m (Génier 2009: 226); km 7.3 road Sarayacu-Loreto, 1200 m (Génier 2009: 226); Río Jatun Yacu, río Napo (Génier 2009: 226); Tena, 400–500 m (Génier 2009: 226). NAPO [= ORELLANA]: Laguna Taracoa, 244 m (Génier 2009: 226). Scyasuni, 250 m (Génier 2009: 226). NAPO [= SUCUMBÍOS]: Río Aguarico, 150 m (Génier 2009: 226); Río Napo-río Aguarico (Génier 2009: 226). ORELLANA: Estación Biológica y Centro de Capacitación IAMOE, Rodrigo Borja (Génier 2009: 226); Estación Científica Yasuní PUCE, 250 m (Génier 2009: 226); Estación de Biodiversidad Tiputini USFQ, Parque Nacional Yasuní, 220 m (Génier 2009: 226); Sendero Chorongo, Estación de Biodiversidad Tiputini USFQ, Parque Nacional Yasuní (Génier 2009: 226); Yuturi Lodge (Génier 2009: 226). PASTAZA: 1 km E de Mera, 1100 m (Génier 2009: 226); 8 km Río Negro, 10 km O Pastaza, Shell, 1400 m (Génier 2009: 227); Llandia, 17 km N del Puyo, 1000 m (Génier 2009: 226); Pandanuque Villano, 420 m (Génier 2009: 226); plataforma Villano (Génier 2009: 226); Villano (Génier 2009: 226). PICHINCHA: 113 km NW Quito on Puerto Quito road, 740–820 m (Génier 2009: 226); Estación Biológica Maquipucuna, 1600–1650 m (Génier 2009: 226). PICHINCHA [= SANTO DOMINGO DE LOS TSÁCHILAS]: Tinalandia 16 km SE Santo Domingo, 680 m (Génier 2009: 226); Tinalandia, Santo Domingo (Génier 2009: 226). SUCUMBÍOS: 2 km N de Limoncocha, 250 m (Génier 2009: 227); Dureno Río Aguarico, 150 m (Génier 2009: 227); El Reventador (Génier 2009: 227); Selva Lodge 150 km down Río Napo from Coca (Génier 2009: 227); Limoncocha, 250 m (Génier 2009: 227); Zabalo 520 m (Génier 2009: 227). TUNGURAHUA: 3 km O Río Negro, 1200 m (Génier 2009: 227). ZAMORA CHINCHIPE: road Cumbaritza-Gualaquiza km 1, 1100 m (Génier 2009: 227); road Namirez-Zamora km 1, 1000 m (Génier 2009: 227); road Zumbi-Yantzaza, km 4, 900 m (Génier 2009: 227).

###### Temporal data.

Collected every month of the year.

###### Remarks.

Inhabits coastal lowland evergreen forests, coastal lowland semi-deciduous forests, and coastal evergreen foothill forests from 200–1200 m a.s.l. In the Andean region, it was recorded in the evergreen lower montane forests and montane cloud forests from 1400–2300 m a.s.l. In the Amazon, it was recorded in the lowland evergreen forests, varzea forests, and the foothill evergreen forests from 150–1300 m a.s.l. Collected with flight interception traps and pitfall traps baited with carrion and different vertebrate feces.

##### Eurysternus cayennensis

Taxon classificationAnimaliaColeopteraScarabaeidae

Castelnau, 1840

Plate 29D

Eurysternus cayennensis Castelnau, 1840: 93 (original description. Type locality: Cayenne).
Eurysternus cayennensis : Gemminger and Harold 1869: 1023 (catalog, distribution); Gillet 1911a: 25 (catalog); Blackwelder 1944: 197 (list of species from Latin America); Vulcano and Pereira 1967: 547 (characters in key); Halffter and Halffter 1976: 73 (redescription); Jessop 1985: 1095 (characters in key), 1105 (redescription); Martínez 1988a: 283 (comment); Medina et al. 2001: 135 (list of species from Colombia); Génier 2009: 25 (diagnosis), 279 (characters in key); Camero 2010: 150 (characters in key) 161 (diagnosis); Carvajal et al. 2011: 314–315 (cited for Ecuador); Krajcik 2012: 107 (complete list of species); Boilly and Vaz-de-Mello 2013: 104 (figure 1); Ratcliffe et al. 2015: 195 (cited for Peru); Chamorro et al. 2018: 95 (cited for Ecuador).
Eurysternus (Eurysternus) cayennensis : Vaz-de-Mello 2000: 193 (cited for Brazil).
Eurysternus confusus Jessop, 1985: 1106 (original description); Medina et al. 2001: 135 (cited for Colombia); Celi et al. 2004 (cited for Ecuador); Génier 2009: 25 (synonym of Eurysternuscayennensis Castelnau, 1840); Camero 2010: 162 (cited as synonym of Eurysternuscayennensis Castelnau, 1840); Carvajal et al. 2011: 314–315 (cited for Ecuador).
Eurysternus (Eurysternus) confusus : Martínez 1988a: 283 (comment); Vaz-de-Mello 2000: 193 (cited for Brazil).

###### Type specimens.

*Eurysternuscayennensis* Castelnau, 1840. The neotype (♂) is deposited at the MNHN (see Génier 2009: 26). Locality: Cayene, Paramana, not examined.

*Eurysternusconfusus* Jessop, 1985. The holotype (♂) is deposited at the CMNC (see Génier 2009: 26). Locality: Ecuador, Dureno, not examined.

###### Distribution.

Bolivia, Brazil, Colombia, Ecuador, French Guiana, Peru, and Surinam.

###### Records examined.

MORONA SANTIAGO: Cumpi, Cordillera del Kutukú (1 specimen CEMT; 1 specimen MUTPL); Macas, 1000 m (1 specimen CEMT); Comunidad Unsuants sitio 3, 700 m, Cordillera del Kutukú (1 specimen CEMT; 12 specimens MECN). NAPO: Chaco Parroquia Gonzalo Diaz de Pineda (1 specimen CEMT); Santo Domingo de Hollin, 635 m, Rio Hollin (1 specimen MUTPL). ORELLANA: Bloque 31, Parque Nacional Yasuní, 200 m (7 specimens MECN); Bloque 31 Punto III, Parque Nacional Yasuní, 200 m (4 specimens MECN); Dayuma Campo Hormiguero, plataforma Hormiguero, 320 m (12 specimens MUTPL); Dayuma Campo Palanda, plataforma Yuca 13, 255 m (2 specimens MUTPL); Cononaco, Bloque 16 YPF, 250 m (1 specimen MUTPL); Dayuma plataforma Ungurahua, 300 m (1 specimen MUTPL); El Dorado plataforma Guarango, 300 m (1 specimen MUTPL); Estación Biológica y Centro de Capacitación IAMOE, Rodrigo Borja (8 specimens CEMT; 7 specimens MECN); Estación Científica Yasuní, 250 m, Parque Nacional Yasuní (1 specimen CEMT); Estación de Biodiversidad Tiputini, 220 m, Parque Nacional Yasuní (3 specimens MUTPL); Ines Arango road Tiwino-río Shiripuno, 250 m (1 specimen MUTPL); Parque Nacional Yasuní, Bloque 31 Punto IV, 200 m (1 specimen CEMT); Parque Nacional Yasuní, Bloque 31 Punto III, 200 m (2 specimens CEMT); Parque Nacional Yasuní, Bloque 31 Punto I OBE, 200 m (1 specimen CEMT); Pozo Daimi 1 (5 specimens CEMT); Río Tiputini Yasuní Res. Stn. (10 specimens CEMT); Rumiyacu (2 specimens MUTPL); San Sebastian del Coca Comuna Guataraco, 345 m, Campo Pata (1 specimen MUTPL); Yasuní (1 specimen CEMT). PASTAZA: Bosque Protector Oglán Alto, 660–705 m (1 specimen MUTPL); San Virgilio (2 specimens MUTPL). SUCUMBÍOS: Bermejo plataforma ER-A road to Lumbaqui (1 specimen MUTPL); Nueva Loja plataforma Iguana, 310 m (1 specimen MUTPL); Tarapoa Campo Marian, plataforma Fanny 5, 260 m (4 specimens MUTPL); Tarapoa, Nuevo Manabí, 270 m (1 specimen MUTPL).

###### Literature records.

MORONA SANTIAGO: Comunidad Unsuants sitio 2, 500 m (Génier, 2009: 27); Comunidad Unsuants sitio 4, 1100 m (Génier, 2009: 27). NAPO: 12 km WSW Tena, 600 m (Génier, 2009: 27); 20 km S del Tena, 600 m (Génier, 2009: 27); Estación Biológica Jatun Sacha, 21 km E Puerto Napo, 400 m (Génier, 2009: 27). NAPO [= ORELLANA]: Scyasuní, 250 m (Génier 2009: 27). NAPO [= SUCUMBÍOS]: Río Aguarico, 150 m (Génier 2009: 27); Río Napo-Río Aguarico (Génier 2009: 27); Zancudo Cocha (Génier 2009: 28); Limoncocha (Jessop 1985: 1106). ORELLANA: Coca [= Puerto Francisco de Orellana], 250 m (Génier 2009: 27); Estación Biológica y Centro de Capacitación IAMOE, Rodrigo Borja (Génier 2009: 27); Estación Científica Yasuní, 250 m, Parque Nacional Yasuní (Génier 2009: 27); Estación de Biodiversidad Tiputini, 220 m, Parque Nacional Yasuní (Génier 2009: 28); Onkone Gare Camp, 220 m (Génier 2009: 28); Río Yasuní, Site No. 2 (Génier 2009: 28). PASTAZA: Limoncocha, 250 m (Génier 2009: 28). PICHINCHA: Nono (Génier 2009: 28). SUCUMBÍOS: Dureno, Río Aguarico, 150 m (Génier 2009: 28; Jessop 1985: 1106); Limoncocha, 250 m (Jessop 1985: 1106; Génier 2009: 28); Shushufindi, 215 m (Génier 2009: 28).

###### Temporal data.

Collected in every month of the year.

###### Remarks.

Species recorded in the cloud forests of the western slopes of the Andes; however, there is only a single record from this locality cited by Génier (2009) and may be erroneous. In the Amazon it was registered in the lowland evergreen and evergreen foothill forests from 150–1100 m a.s.l. Collected with flight interception traps and pitfall traps, baited with carrion and human feces.

##### Eurysternus contractus

Taxon classificationAnimaliaColeopteraScarabaeidae

Génier, 2009

Plate 30A

Eurysternus contractus Génier, 2009: 116 (original description. Type locality: Ecuador, Pastaza, 1000 m, Llandia, 17 km N de Puyo).
Eurysternus contractus : Camero 2010: 149 (characters in key), 157 (diagnosis); Krajcik 2012: 107 (complete list of species); Ratcliffe et al. 2015: 195 (cited for Peru); Chamorro et al. 2018: 95 (cited for Ecuador).

###### Type specimens.

*Eurysternuscontractus* Génier, 2009. The holotype (♂) is deposited at the CMNC (see Génier 2009: 118). Locality: ECUADOR, PAST. 1000 m, Llandia (17 km N Puyo), not examined.

###### Distribution.

Colombia, Ecuador, and Peru.

###### Records examined.

LOJA: Loja/Zamora, 1400 m (1 specimen CEMT). MORONA SANTIAGO: Camino Río Chiviaza (1 specimen MECN); Chiguinda Río Blanco, 1730 m (4 specimens MUTPL); San Antonio, Limon Indazo Centro Shuar Wuarints (1 specimen MECN). NAPO: Pacto Sumaco, 1620 m (2 specimens MUTPL). TUNGURAHUA: Baños El Topo, 1590 m (7 specimens CEMT). ZAMORA CHINCHIPE: Chito Río Sangolas, 1800 m (2 specimens MUTPL); Chito Río San Francisco, 1505 m (1 specimen MUTPL); Bombuscaro, Parque Nacional Podocarpus (1 specimen MUTPL); RVS El Zarza campamento las Peñas, Cordillera del Cóndor, conseción El Colibri, 1535 m (1 specimen MUTPL); RVS El Zarza, Cordillera del Cóndor, conseción El Zarza, 1710 m (1 specimen MUTPL); Tundayme campamento Mirador, La Escombrera Norte, 1245 m (1 specimen MUTPL); Zurmi Comunidad Miazi, 1380 m (1 specimen MEPN); Zurmi, Pachikuntza, 1685 m (1 specimen MUTPL); Zurmi, Comunidad La Wants, 1010 m (1 specimen MEPN).

###### Literature records.

LOJA: Loja (Génier, 2009: 118); Loja/Zamora, 1400 m (Génier 2009: 118). MORONA SANTIAGO: Ángel Rouby, sitio 8, 1300 m (Génier 2009: 118). NAPO: Chonta Yacu road Tena-Coca, 1100 m (Génier 2009: 118); km 11.1 Sarayacu-Loreto road, 1200 m (Génier 2009: 118); km 25.4 Sarayacu-Loreto road, 950 m (Génier 2009: 118); km 7.3 Sarayacu-Loreto road, 1200 m (Génier 2009: 118); Río Chonta Yacu road Tena-Coca, 1100 m (Génier 2009: 118). PASTAZA: 1 km E de Mera, 1100 m (Génier 2009: 118); 22 km SE del Puyo, 900 m (Génier 2009: 118); 25 km NNE Puyo, 1000 m (Génier 2009: 118); Arajuno, 400–500 m (Génier 2009: 118); Cerros de Abitagua, 1200 m (Génier 2009: 118); Llandia 17 km N del Puyo, 1000 m (Génier 2009: 118); Puyo environs (Génier 2009: 118). SUCUMBÍOS: Lombaqui [= Lumbaqui] 800 m (Génier 2009: 118). TUNGURAHUA: 8 km E Río Negro, 10 km O Pastaza, Shell 1400 m. ZAMORA CHINCHIPE: road Namirez-Zamora km 1, 1000 m (Génier 2009: 118).

###### Temporal data.

Collected in every month except March and April.

###### Remarks.

Inhabits the foothill forests of the Amazon region from 800–1300 m a.s.l. In the Andean region, it was recorded in the evergreen lower montane from 1400–1800 m a.s.l. Collected with pitfall traps baited with carrion and human feces.

##### Eurysternus foedus

Taxon classificationAnimaliaColeopteraScarabaeidae

Guérin-Méneville, 1830

Plate 30B

Eurysternus foedus Guérin-Méneville, 1830: 76. [pl. 21, figs 5, 5.a] (original description. Type locality: Brésil [= Brazil]).
Eurysternus foedus : Guérin-Méneville 1844: 76 (redescription); Castelnau 1840: 93 (comment); Gemminger and Harold 1869: 1024 (catalog, cited as Eurysternusfoetidus Guér); Ohaus 1909: 94 (cited for Ecuador); Gillet 1911a: 25 (catalog); Pessôa and Lane 1941: 409 (redescription); Blackwelder 1944: 197 (list of species of Latin America); Vulcano and Pereira 1967: 547 (characters in key); Halffter and Halffter 1976: 78 (redescription); Jessop 1985: 1093 (characters in key), 1102 (distribution); Medina et al. 2001: 135 (cited for Colombia); Ratcliffe 2002: 11 (cited for Panama); Morón 2003: 45 (cited for Mexico); Huerta et al. 2003: 24 (biology); Hamel-Leigue et al. 2006: 16 (cited for Bolivia); Génier 2009: 146 (diagnosis), 288 (characters in key); Camero 2010: 149 (characters in key) 153 (diagnosis); Carvajal et al. 2011: 314–315 (cited for Ecuador); Krajcik 2012: 107 (complete list of species); Solís and Kohlmann 2012: 7 (cited for Costa Rica); Ratcliffe et al. 2015: 195 (cited for Peru); Chamorro et al. 2018: 95 (cited for Ecuador).
Eurysternus (Eurysternus) foedus : Vaz-de-Mello 2000: 193 (cited for Brazil).
Eurysternus claudicans Kirsch, 1871: 344 (original description); Harold 1880a: 13 (distribution); Bates 1887: 39 (distribution); Gillet 1911a: 25 (catalog); Campos 1921: 57 (cited for Ecuador); Blackwelder 1944: 197 (list of species from Latin America); Howden and Young 1981: 14 (characters in key), 15 (redescription); Jessop 1985: 1102 (synonym of Eurysternusfoedus Guérin-Méneville, 1830); Ratcliffe 2002: 11 (cited as synonym of Eurysternusfoedus Guérin-Méneville, 1830); Génier 2009: 145 (cited as synonym of Eurysternusfoedus Guérin-Méneville, 1830); Camero 2010: 153 (cited as synonym of Eurysternusfoedus Guérin-Méneville, 1830); Carvajal et al. 2011: 314–315 (cited for Ecuador); Solís and Kohlmann 2012: 7 (cited as synonym of Eurysternusfoedus Guérin-Méneville, 1830).

###### Type specimens.

*Eurysternusfoedus* Guérin-Méneville, 1830. The neotype (♀) is deposited at the NHML (see Génier 2009: 146). Locality: Brazil, Mato Grosso, 264 km N of Xavantina, Serra do Roncador, not examined.

*Eurysternusclaudicans* Kirsch, 1871. The holotype (♀) is deposited at the SMTD (see Génier 2009: 146). Locality: Bogota (not examined).

###### Distribution.

Belize, Bolivia, Brazil, Colombia, Costa Rica, Ecuador, French Guiana, Guatemala, Mexico, Nicaragua, Panama, Peru, and Venezuela.

###### Records examined.

ESMERALDAS: Colón del Ónzole (1 specimen MGO-UC; 3 specimens MECN); Gualpí del Ónzole (1 specimen MGO-UC); Kumanii Lodge, 40 m (1 specimen MQCAZ); Majua (5 specimens MGO-UC; 4 specimens MECN); Palma Real (3 specimens MECN); Playa de Oro, Padre Santo (1 specimen MGO-UC; 1 specimen MECN); Playa de Oro, Playa Rica (2 specimens MGO-UC); Tsejpi (5 specimens MGO-UC; 4 specimens MECN). LOS RÍOS: Estación Científica río Palenque, 250 m (3 specimens MQCAZ). MANABÍ: Chone, Antenas de Radío El Día (1 specimen MQCAZ); Puerto López San Sebastian, 350 m (1 specimen MUTPL); Puerto López, Las Tunas, 200 m (1 specimen MUTPL); Puerto López 5 m (1 specimen MUTPL). MORONA SANTIAGO: Comunidad Unsuants, 500–1110 m, Cordillera del Kutukú (5 specimens MECN); Indanza (2 specimens MECN); Nuevo Israel, 1290 m (1 specimen MUTPL). NAPO: Cotundo, Comunidad Rumiñahui, Kuriurcu (1 specimen MUTPL); Archidona, Santo Domingo de Hollín, Río Hollin, 635 m (1 specimen MUTPL). ORELLANA: Dayuma Campo Palanda, plataforma Primavera 1, 235 m (1 specimen MUTPL); Dayuma plataforma Ungurahua, 300 m (1 specimen MUTPL); Estación Científica Yasuní Puce, 250 m, Parque Nacional Yasuní (25 specimens MQCAZ); Estación de Biodiversidad Tiputini USFQ, 220 m, Parque Nacional Yasuní (4 specimens MGO-UC); Ines Arango road Tiwino-río Shiripuno, 250 m (1 specimen MUTPL). PASTAZA: Bosque Protector Oglán Alto, 555–950 m (1 specimen MUTPL); Chuyayacu Oleoducto km 25, 200 m (1 specimen MUTPL); Centro Fátima km 9 road Puyo-Tena, 980 m (3 specimens MECN); Estación Biologica Pindo-Mirador UTE, 1000 m (1 specimen MUTPL). PICHINCHA: Llurimaguas Río Guayllabamba, 290 m, Pedro Vicente Maldonado (2 specimens MUTPL). SANTA ELENA: Olón, 50 m (73 specimens CEMT). SANTO DOMINGO DE LOS TSÁCHILAS: Santo Domingo, Tinalandia Resort, 760 m (1 specimen MQCAZ); Santo Domingo, Puerto Limón, 400 m (1 specimen MUTPL). SUCUMBÍOS: Tarapoa Campo Marian, plataforma Fanny 5, 260 m (1 specimen MUTPL).

###### Literature records.

BOLIVAR: Chimbo (Campos 1921: 57). COTOPAXI: Chugchilán, 2600 m (Génier 2009: 153). ESMERALDAS: Bilsa (Génier 2009: 153); Colón del Ónzole (Génier 2009: 153); Estación Forestal La Chiquita, 11 km SE San Lorenzo, 5 m (Génier 2009: 153); Gualpí del Ónzole (Génier 2009: 153); Majua (Génier 2009: 153); Pajonal (Génier 2009: 153); Palma Real (Génier 2009: 153); Prov. San Mateo (Génier 2009: 153); Punta Venado (Génier 2009: 153); Salto del Bravo (Génier 2009: 153); San Mateo (Génier 2009: 153). GUAYAS: Bucay (Campos 1921: 57; Génier 2009: 153); Duran (Campos 1921: 57); Naranjito (Campos 1921: 57). GUAYAS [= SANTA ELENA]: 27 km S Puerto López, 76 km N de Santa Elena, 152 m (Génier 2009: 153). LOS RÍOS: 47 km S Santo Domingo 213 m (Génier 2009: 153); Estación Científica río Palenque, 250 m, 47 km S Santo Domingo (Génier 2009: 153). Estación Experimental Tropical Pichilingue, Quevedo (Génier 2009: 154); Río Palenque (Génier 2009: 154). MANABÍ: 20 km N de Chone, 300 m (Génier 2009: 154); 78 km NE Chone, 85 km WSW Santo Domingo, 450 m (Génier 2009: 154); 90 km WSW de Santo Domingo, 73 km NE de Chone, 300 m (Génier 2009: 154). NAPO: 10 km O Puerto Misahualli (Génier 2009: 154); 12 km WSW Tena, 600 m (Génier 2009: 154); 13 km SW Tena (Génier 2009: 154); 20 km S Tena, 600 m (Génier 2009: 154); 5 km O Tena, 500 m (Génier 2009: 154); Capirona, Río Arajuno (Génier 2009: 154); Estación Biológica Jatun Sacha, 21 km E Puerto Napo, 400 m (Génier 2009: 154); Misahualli Jungle Lodge area, junction of Río Napo-Río Misahuallí, 579 m (Génier 2009: 154); Río Jatun Yacu-Río Napo watershed (Génier 2009: 154); Tena, 400–500 m (Génier 2009: 154). ORELLANA: Estación Científica Yasuní PUCE, 250 m (Génier 2009: 154). PASTAZA: 22 km SE Puyo, 900 m (Génier 2009: 155); Arajuno, 400–500 m (Génier 2009: 155); Llandia 17 km N Puyo, 1000 m (Génier 2009: 155). PICHINCHA: 113 km NW Quito on Puerto Quito road, 792 m; 5.3 km on road Pachijal, 2800–3000 m (Génier 2009: 155). PICHINCHA [= SANTO DOMINGO DE LOS TSÁCHILAS]: 4 km SE Santo Domingo, 500 m (Génier 2009: 155); E. Alluriquin, Tinalandia, 700 m (Génier 2009: 155); Río Silanche, 760 m (Génier 2009: 155); Río Toachi, Santo Domingo de los Colorados (Génier 2009: 155). Tinalandia, 16 km SE Santo Domingo, 680 m (Génier 2009: 155). SUCUMBÍOS: 2 km N de Limoncocha, 250 m (Génier 2009: 155); Dureno Río Aguarico, 150 m (Génier 2009: 155); Limoncocha, 250 m (Génier 2009: 155). TUNGURAHUA: Baños (Génier 2009: 155). UNDETERMINED PROVINCE: El Salado (Campos 1921: 57); San Rafael (Campos 1921: 57).

###### Temporal data.

Collected every month of the year.

###### Remarks.

Inhabits coastal lowland evergreen forests, coastal lowland semi-deciduous forests, and coastal evergreen foothill forests from 5–790 m a.s.l. In the Andean region, it was recorded in the evergreen high montane forests from 2800–3000 m a.s.l. In the Amazon, it was recorded in the lowland evergreen forests and the foothill evergreen forests from 150–1100 m a.s.l. Collected manually and with pitfall traps baited with carrion and human feces.

##### Eurysternus hamaticollis

Taxon classificationAnimaliaColeopteraScarabaeidae

Balthasar, 1939

Plate 30C

Eurysternus hamaticollis Balthasar, 1939g: 113 (original description. Type locality: Guiana (Godebert-Maroni), Bolivia (Buenavista bei), and Brazilien [= Brazil]).
Eurysternus hamaticollis : Blackwelder 1944: 197 (list of species from Latin America); Vulcano and Pereira 1967: 547 (characters in key); Jessop 1985: 1093 (characters in key), 1101 (distribution); Medina et al. 2001: 135 (cited for Colombia); Hamel-Leigue et al. 2006: 16 (cited for Bolivia); Génier 2009: 240 (redescription), 291 (characters in key); Camero 2010: 150 (characters in key), 166 (diagnosis); Carvajal et al. 2011: 314–315 (cited for Ecuador); Bezdek and Hajek 2012: 316 (catalog of type NMPC); Krajcik 2012: 107 (complete list of species); Solís and Kohlmann 2012: 7 (cited for Costa Rica); Ratcliffe et al 2015: 195 (cited for Peru); Chamorro et al. 2018: 95 (cited for Ecuador).
Eurysternus (Eurysternus) s. str.hamaticollis : Martínez 1988a: 283 (distribution); Vaz-de-Mello 2000: 193 (list of species for Brazil).

###### Type specimens.

*Eurysternushamaticollis* Balthasar, 1939. The lectotype (♂) is deposited at the MSMF (see Génier 2009: 241). Locality: French Guiana, Godebert-Maroni, not examined.

###### Distribution.

Bolivia, Brazil, Colombia, Costa Rica, Ecuador, French Guiana, Guyana, Peru, and Venezuela.

###### Records examined.

ORELLANA: Comunidad Kiwcha Chiruisla Station, 180–250 m (1 specimen MQCAZ); Dayuma Campo Hormiguero, plataforma Hormiguero, 320 m (1 specimen MUTPL); Dayuma Campo Palanda, 235 m, plataforma Primavera 1 (1 specimen MUTPL); Dayuma plataforma Ungurahua, 300 m (1 specimen MUTPL); El Dorado plataforma Guarango, 300 m (1 specimen MUTPL); Estación Científica Yasuní PUCE, 250 m, Parque Nacional Yasuní (8 specimens MQCAZ); Estación de Biodiversidad Tiputini USFQ, Parque Nacional Yasuní, 200 m (3 specimens MUTPL); Estación Río Huiririma, 220 m (1 specimen MQCAZ); Rodrigo Borja IAMOE (2 specimens CEMT); San Sebastian del Coca, Comuna Guataraco, 345 m, Campo Pata (1 specimen MUTPL); San Sebastian del Coca, 345 m, Comuna Shamanal Campo Palo Azul (1 specimen MUTPL); Lago San Pedro, plataforma Copal, 310 m (1 specimen MUTPL). PASTAZA: Bosque Protector Oglán Alto, 555–660 m (1 specimen MUTPL); Pandanuque, 420 m (2 specimens MUTPL). SUCUMBÍOS: Tarapoa, Nuevo Manabí, 270 m (1 specimen MUTPL).

###### Literature records.

NAPO [= ORELLANA]: Scyasuni, 200 m (Génier 2009: 242). ORELLANA: Estación Científica Yasuní PUCE, 250 m (Génier 2009: 242); Estación de Biodiversidad Tiputini USFQ, Parque Nacional Yasuní, 220 m (Génier 2009: 242); Río Yasuní Site No. 2 (Génier 2009: 242). PASTAZA: Alto río Bobonaza, Oriente (Génier 2009: 242). SUCUMBÍOS: 2 km N de Limoncocha, 250 m (Génier 2009: 242); Dureno río Aguarico, 150 m (Génier 2009: 242); La Selva Lodge, 150 km down Río Napo from Coca (Génier 2009: 242); Limoncocha, 250 m (Génier 2009: 242); Lombaqui [= Lumbaqui], 800 m (Génier 2009: 242).

###### Temporal data.

Collected in every month except January and July.

###### Remarks.

Inhabits the lowland evergreen forests and evergreen foothill forests of the Amazon region from 180–800 m a.s.l. Collected manually and with pitfall traps baited with carrion and human feces.

##### Eurysternus hypocrita

Taxon classificationAnimaliaColeopteraScarabaeidae

Balthasar, 1939

Plate 30D

Eurysternus hypocrita Balthasar, 1939g: 114 (original description. Type locality: Franz Guiana (Gourdenville, Cayenne), Surinam, Peru, Ecuador, Columbien [= Colombia], and Brazilien [= Brazil]).
Eurysternus hypocrita : Balthasar 1941: 340 (cited for Peru); Blackwelder 1944: 197 (list of species of Latin America); Balthasar 1951: 325 (cited for Peru); Halffter and Halffter 1976: 55 (comment); Jessop 1985: 1101 (cited as synonym of Eurysternusvelutinus Bates, 1887); Génier 2009: 134 (redescription), 287 (characters in key); Camero 2010: 149 (characters in key), 156 (diagnosis); Carvajal et al. 2011: 314–315 (cited for Ecuador); Krajcik 2012: 107 (complete list of species); Bezdek and Hajek 2012: 316 (catalog of type NMPC); 317 (comment); Ratcliffe et al. 2015: 195 (cited for Peru); Chamorro et al. 2018: 95 (cited for Ecuador).

###### Type specimens.

*Eurysternushypocrita* Balthasar, 1939. The lectotype (♂) is deposited at the MSMF (see Génier 2009: 134). Locality: Cayenne, not examined.

###### Distribution.

Bolivia, Brazil, Colombia, Ecuador, French Guiana, Guyana, Peru, Surinam, and Venezuela.

###### Records examined.

MORONA SANTIAGO: Comunidad Unsuants, 700 m, Cordillera del Kutukú (3 specimens MECN); Nuevo Israel, Cordillera del Kutukú (2 specimens MUTPL). NAPO: sector Talac, 730 m, Pungarayacu (1 specimen MQCAZ); Tena (6 specimens CEMT). ORELLANA: Bloque 31 Parque Nacional Yasuní, 200 m (2 specimens MECN); Comunidad Kiwcha Chiruisla Station, 180–250 m (4 specimens MQCAZ); Dayuma Campo Hormiguero, plataforma Hormiguero, 320 m (1 specimen MUTPL); Dayuma Campo Palanda, 235 m, plataforma Primavera 1 (1 specimen MUTPL); Dayuma Campo Pindo, 290 m, plataforma Pindo 9 (1 specimen MUTPL); Dayuma plataforma Ungurahua, 300 m (1 specimen MUTPL); El Dorado plataforma Guarango, 300 m (1 specimen MUTPL); Estación Biológica y Centro de Capacitación IAMOE, Rodrigo Borja (6 specimens CEMT); Estación Científica Yasuní PUCE, 250 m, Parque Nacional Yasuní (24 specimens MQCAZ); Estación de Biodiversidad Tiputini USFQ, 220 m, Parque Nacional Yasuní (1 specimen MUTPL); Pozo Nashiño Bloque 31, Parque Nacional Yasuní, 250 m (2 specimens MECN); San Sebastian del Coca, Comuna Guataraco Campo Pata, 345 m (2 specimens CEMT); San Sebastian del Coca, Comuna Shamanal Campo Palo Azul, 345 m (1 specimen MUTPL). PASTAZA: Bosque Protector Oglán Alto, 555 m (1 specimen CEMT; 2 specimens MUTPL); Nuevo San Jose del Curaray, 245 m (1 specimen MUTPL); Villano Pandanuque (1 specimen MUTPL). SUCUMBÍOS: 6 km de Dureno, 290 m, Precooperativa Los Vergeles (1 specimen MGO-UC); Bermejo plataforma ER-A road to Lumbaqui (1 specimen MUTPL); Nueva Loja plataforma Iguana, 310 m (1 specimen MUTPL); Pacayacu Campo Libertador, 260 m (1 specimen MUTPL); Reserva de Producción Faunística Cuyabeno trocha Zábalo-Guepi (1 specimen MUTPL); Tarapoa Campo Marian, 260 m, plataforma Fanny 5 (1 specimen MUTPL); Tarapoa, Nuevo Manabí, 270 m (1 specimen MUTPL). ZAMORA CHINCHIPE: Zurmi, Comunidad La Wants, 1010 m (1 specimen MEPN; 1 specimen MUTPL); Zurmi Las Orquideas Río Nangaritza, 870 m (1 specimen MUTPL).

###### Literature records.

GUAYAS [= SANTA ELENA]: 27 km S de Puerto López, 76 km N de Santa Elena, 152 m (Génier 2009: 137). MORONA SANTIAGO: Comunidad Unsuants sitio 1, 700 m (Génier 2009: 137). NAPO: 12 km WSW Tena, 600 m (Génier 2009: 137); 20 km S Tena, 600 m (Génier 2009: 137); 3.3 km E Puerto Napo, 400 m (Génier 2009: 137); Estación Biologica Jatun Sacha, 450 m (Génier 2009: 137); Estación Biologica Jatun Sacha, 21 km Puerto Napo, 400 m (Génier 2009: 137). NAPO [= ORELLANA]: Scyasuni (Génier 2009: 137). ORELLANA: Estación Biológica y Centro de Capacitación IAMOE, Rodrigo Borja (Génier 2009: 137); Estación Científica Yasuní PUCE, 250 m (Génier 2009: 138); Estación de Biodiversidad Tiputini USFQ, Parque Nacional Yasuní, 220 m (Génier 2009: 138). NAPO [= SUCUMBÍOS]: Río Aguarico, 150 m (Génier 2009: 137); Río Yasuní site No. 2 (Génier 2009: 138). PASTAZA: 22 km SE Puyo, 900 m (Génier 2009: 138); Arajuno (Génier 2009: 138); Chichirota (Génier 2009: 138). SUCUMBÍOS: Dureno Río Aguarico, 150 m (Génier 2009: 138); Tarapoa (Génier 2009: 138); Zábalo, 520 m (Génier 2009: 138).

###### Temporal data.

Collected every month of the year.

###### Remarks.

Inhabits the lowland evergreen forests at 152 m a.s.l. However, this record cited by Génier (2009) may be erroneus. In the Amazon it was recorded in the lowland evergreen forests and foothill evergreen forests from 150–1010 m a.s.l. Collected manually and with pitfall traps baited with carrion and human feces.

##### Eurysternus lanuginosus

Taxon classificationAnimaliaColeopteraScarabaeidae

Génier, 2009

Plate 31A

Eurysternus lanuginosus Génier, 2009: 70 (original description. Type locality: Ecuador, Pastaza, 1000 m, Llandia, 17 km N of Puyo).
Eurysternus lanuginosus : Camero 2010: 149 (characters in key), 152 (diagnosis); Krajcik 2012: 107 (complete list of species); Ratcliffe et al. 2015: 195 (cited for Peru); Chamorro et al. 2018: 95 (cited for Ecuador).

###### Type specimens.

*Eurysternuslanuginosus* Génier, 2009. The holotype (♂) is deposited at the CMNC (see Génier 2009: 71). Locality: Ecuador, Pastaza. 1000 m. Llandia, 17 km N de Puyo, not examined.

###### Distribution.

Bolivia, Brazil, Colombia, Ecuador, and Peru.

###### Records examined.

MORONA SANTIAGO: Nuevo Israel, Cordillera del Kutukú, 1290 m (1 specimen MUTPL); Untsuants, 700 m, Cordillera del Kutukú (2 specimens MECN). NAPO: Cotundo, 1070 m, Río Osayacu sector Shamato (1 specimen MUTPL); El Capricho, km 51 ruta Tena-Ambato (1 specimen CEMT); La Merced de Jondachi, 1100 m, Río Jondachi (1 specimen MUTPL). ORELLANA: Bloque 31 Parque Nacional Yasuní, 200 m (3 specimens MECN); Campo Palanda, LLumpac, 295 m (1 specimen CEMT); Cononaco, Bloque 16 YPF Parque Nacional Yasuní, 250 m (1 specimen MUTPL); El Dorado plataforma Guarango, 300 m (1 specimen MUTPL); Río Tiputini Yasuní Res. Stn. (1 specimen CEMT; 1 specimen MUTPL); San Sebastian del Coca, Comuna Guataraco Campo Pata, 345 m (1 specimen MUTPL); San Sebastian del Coca, Comuna Shamanal 345 m, Campo Palo Azul (1 specimen CEMT); Lago San Pedro, plataforma Copal, 310 m (1 specimen CEMT). PASTAZA: Bosque Protector Oglán Alto, 540–950 m (3 specimens CEMT; 1 specimen MUTPL); Chuyayacu km 25 Oleoducto, 200 m (1 specimen MUTPL); Villano Pandanuque, 420 m (1 specimen CEMT; 1 specimen MUTPL). SUCUMBÍOS: Aucayacu Río El Eno, 275 m, 16 km de Lago Agrio (1 specimen CEMT; 4 specimens MGO-UC); Bermejo plataforma ER-A road to Lumbaqui (1 specimen MUTPL); Gonzalo Pizarro Símon Bolivar, 1200 m (1 specimen MECN); Shushufindi Recinto La Pantera, 250 m (1 specimen CEMT); Tarapoa, Nuevo Manabí, 270 m (1 specimen MUTPL). ZAMORA CHINCHIPE: (Zurmi Comunidad Miazi, 1380 m (1 specimen MEPN; 1 specimen MUTPL).

###### Literature records.

MORONA SANTIAGO: Macas (Génier 2009: 71); Untsuants sitio 1, 700 m (Génier 2009: 71). NAPO: 12 km WSW Tena, 600 m (Génier 2009: 71); 20 km S de Tena, 600 m (Génier 2009: 71); 5 km O de Tena, 500 m (Génier 2009: 71); Estación Biológica Jatun Sacha, 21 km E de Puerto Napo, 400 m (Génier 2009: 71); 11.1 km Sarayacu-Loreto road, 1200 m (Génier 2009: 72); km 25.4 Sarayacu-Loreto road, 950 m (Génier 2009: 72); km 7.3 Sarayacu-Loreto road, 1200 m (Génier 2009: 72); Tena, 400–500 m (Génier 2009: 72); Yampona (Génier 2009: 72). ORELLANA: Daimi [= Pozo petrolero Daimi] (Génier 2009: 72); Estación Científica Yasuní PUCE, 215 m (Génier 2009: 72); Estación de Biodiversidad Tiputini USFQ, Parque Nacional Yasuní, 220 m (Génier 2009: 72); Zancudo Cocha (Génier 2009: 72). PASTAZA: 1 km E de Mera, 1100 m (Génier 2009: 72); 22 km SE Puyo, 900 m (Génier 2009: 72); 25 km NNE del Puyo, 1000 m (Génier 2009: 72); 9 km ESE de Veracruz, 900 m (Génier 2009: 72); Amazanga, near Puyo, 1000 m (Génier 2009: 72); Arajuno, environ, 750 m (Génier 2009: 72); Llandia, 1000 m, 17 km N de Puyo (Génier 2009: 72). SUCUMBÍOS: 2 km N de Limoncocha, 250 m (Génier 2009: 72); Dureno río Aguarico, 150 m (Génier 2009: 72); Limoncocha, 250 m (Génier 2009: 72); Reserva Biológica Limoncocha, 250 m (Génier 2009: 73); Sacha Lodge, 270 m (Génier 2009: 72). TUNGURAHUA: 8 km E of río Negro, 10 km O Pastaza [= Shell], 1400 m.

###### Temporal data.

Collected every month of the year.

###### Remarks.

Inhabits the lowland evergreen forests, evergreen foothill forests, and evergreen lower montane forests of the Amazon from 150–1500 m a.s.l. Collected with pitfall traps baited with carrion and human feces.

##### Eurysternus marmoreus

Taxon classificationAnimaliaColeopteraScarabaeidae

Castelnau, 1840

Plate 31B

Eurysternus marmoreus Castelnau, 1840: 93 (original description. Type locality: Mexique [= Mexico] and Colombie [= Colombia]).
Eurysternus marmoreus : Guérin-Méneville 1855: 590 (redescription); Gemminger and Harold 1869: 1024 (catalog); Harold 1880a: 13 (distribution); Gillet 1911a: 25 (catalog); Blackwelder 1944: 197 (list of species from Latin America); Vulcano and Pereira 1967: 547 (characters in key); Jessop 1985: 1091 (characters in key), 1100 (redescription); Medina et al. 2001: 135 (list of species from Colombia); Huerta et al. 2003: 17 (Biology); Génier 2009: 193 (diagnosis), 290 (characters in key); Camero 2010: 150 (characters in key), 171 (diagnosis); Carvajal et al. 2011: 314–315 (cited for Ecuador); Krajcik 2012: 107 (complete list of species); Ratcliffe et al. 2015: 195 (cited for Peru); Chamorro et al. 2018: 78 (figures 1A and 1E), 95 (cited for Ecuador).
Eurysternus pectoralis Guérin-Méneville, 1855: 590 (original description); Gemminger and Harold 1869: 1024 (catalog); Gillet 1911a: 26 (catalog); Blackwelder 1944: 197 (list of species from Latin America); Jessop 1985: 1106 (comment); Génier 2009: 194 (synonym of Eurysternusmarmoreus Castelnau, 1840); Camero 2010: 171 (cited as synonym of Eurysternusmarmoreus Castelnau, 1840).
Eurysternus (Eurysternus) pectoralis : Vaz-de-Mello 2000: 193 (cited for Brazil).

###### Type specimens.

*Eurysternusmarmoreus* Castelnau, 1840. The neotype (♀) is deposited at the MNHN (see Génier 2009: 195). Locality: Fusagas[ugá], not examined.

*Eurysternuspectoralis* Guérin-Méneville, 1855. The neotype (♂) is deposited in MNHN (see Génier 2009: 195). Locality: Ecuador 71, E deVille, not examined.

###### Distribution.

Bolivia, Colombia, Ecuador, Peru, and Venezuela.

###### Records examined.

COTOPAXI: Bosque Integral Otonga, 2200 m (1 specimen CEMT; 6 specimens MQCAZ). NAPO: San Rafael (2 specimens MECN). PICHINCHA: Chiriboga (2 specimens MECN). SUCUMBÍOS: Sebundoy, 2200 m (2 specimens MECN); TUNGURAHUA: Baños EL Topo, 1590 m (3 specimens CEMT). ZAMORA CHINCHIPE: Río San Francisco, 1470 m (1 specimen MUTPL); Reserva Biológica el Colibrí, 2200 m (14 specimens MUTPL); Romerillos sendero Nangaritza, 2200 m (9 specimens MECN).

###### Literature records.

NAPO: 17 km NE de Baeza, 4 km SW del Chaco, 1280 m (Génier 2009: 197); 7 km S de Baeza, 2000 m (Génier 2009: 197); Cabañas San Isidro, 2 km NW de Cosanga, 2150 m (Génier 2009: 197); Piviyacu Los Guacamayos, 1800 m (Génier 2009: 197). UNDETERMINED PROVINCE: Naranjal [= possibly GUAYAS] (Génier 2009: 197).

###### Temporal data.

Collected in January, February, March, May, July, August, October, November, and December.

###### Remarks.

Inhabits coastal lowland evergreen forests. In the Andean region, it was recorded in the evergreen lower montane forests and the montane cloud forests from 1300–2300 m a.s.l. In the Amazon, it was recorded in the foothill evergreen forests at 1280 m a.s.l. Collected with flight interception traps and pitfall traps baited with carrion and human feces.

##### Eurysternus plebejus

Taxon classificationAnimaliaColeopteraScarabaeidae

Harold, 1880

Plate 31C

Eurysternus plebejus Harold, 1880a: 14 (original description. Type locality: Muzo).
Eurysternus plebejus : Ohaus 1909: 94 (cited for Ecuador); Gillet 1911a: 26 (catalog); Blackwelder 1944: 197 (list of species from Latin America); Roze 1955: 41 (cited for Venezuela); Halffter and Matthews 1966: 146 (cited for Ecuador); Vulcano and Pereira 1967: 547 (characters in key); Howden and Young 1981: 14 (characters in key), 15 (redescription); Jessop 1985: 1093 (characters in key), 1100 (distribution); Medina et al. 2001: 135 (cited for Colombia); Ratcliffe 2002: 11 (cited for Panama); Morón 2003: 44 (cited for Mexico); Hamel-Leigue et al. 2006: 16 (cited for Bolivia); Génier 2009: 172 (diagnosis), 289 (characters in key); Camero 2010: 150 (characters in key), 168 (diagnosis); Carvajal et al. 2011: 314–315 (cited for Ecuador); Krajcik 2012: 107 (complete list of species); Solís and Kohlmann 2012: 7 (cited for Costa Rica); Ratcliffe et al. 2015: 195 (cited for Peru); Chamorro et al. 2018: 95 (cited for Ecuador).
Eurysternus (Eurysternus) s. str.plebejus : Vaz-de-Mello 2000: 193 (cited for Brazil).
Eurysternus (Eurysternus) joffrei Martínez, 1988a: 290 (original description); Génier 2009: 172 (synonym of Eurysternusplebejus Harold, 1880); Camero 2010: 168 (cited as synonym of Eurysternusplebejus Harold, 1880); Solís and Kohlmann 2012: 7 (cited as synonym of Eurysternusplebejus Harold, 1880).

###### Type specimens.

*Eurysternusplebejus* Harold, 1880. The lectotype (♂) is deposited at the MNHN (see Génier 2009: 173). Locality: Muzo, not examined.

Eurysternus (Eurysternus) joffrei Martínez, 1988. The holotype (♂) is deposited at the MACN (see Génier 2009: 173). Locality: Peru, D°Huanuco, Tingo Maria, not examined.

###### Distribution.

Brazil, Bolivia, Colombia, Costa Rica, Ecuador, French Guiana, Mexico, Nicaragua, Panama, Peru, Surinam, and Venezuela.

###### Records examined.

EL ORO: Buenaventura Bajo, 500 m (4 specimens MQCAZ); Uzhcurrumi, 500 m (1 specimen CEMT; 3 specimens MQCAZ). ESMERALDAS: Carondelet (2 specimens MQCAZ); Calle Mansa (1 specimen CEMT; 3 specimens MQCAZ; 4 specimens MECN); Colón del Ónzole (4 specimens CEMT; 7 specimens MQCAZ; 11 specimens MECN); Charco Vicente (11 specimens MQCAZ; 6 specimens MECN); Chispero (1 specimen CEMT; 6 specimens MQCAZ; 4 specimens MECN); El Progreso, Guandal (1 specimen MQCAZ); Gallinazo (2 specimens MQCAZ); Gualpi (6 specimens CEMT); Gualpí El Pajonal (1 specimen CEMT; 3 specimens MQCAZ; 2 specimens MECN); Gualpí del Ónzole (5 specimens MQCAZ; 9 specimens MECN); Jeyambi (4 specimens MQCAZ; 3 specimens MECN); Majua (6 specimens CEMT; 11 specimens MQCAZ; 2 specimens MGO-UC; 1 specimen MUTPL; 8 specimens MECN); Pajonal (8 specimens MQCAZ; 4 specimens MECN); Palma Real (8 specimens CEMT; 5 specimens MQCAZ; 1 specimen MGO-UC; 5 specimens MECN); Playa de Oro (21 specimens CEMT; 7 specimens MQCAZ; 6 specimens MECN); Playa de Oro, Padre Santo (2 specimens CEMT; 5 specimens MQCAZ; 5 specimens MECN); Playa de Oro, Río Santiago (1 specimen CEMT; 3 specimens MQCAZ); Playa Rica (3 specimens MQCAZ; 3 specimens MECN); Puerto Balao, 200 m (2 specimens MUTPL); Ricauter (3 specimens MQCAZ); Río Savalo (1 specimen MQCAZ); San Miguel (1 specimen MQCAZ); Salto del Bravo (7 specimens MQCAZ; 8 specimens MECN); Tsejpi (12 specimens MQCAZ; 7 specimens MECN); Zabalito (3 specimens MQCAZ). IMBABURA: Lita, 680 m (3 specimens MQCAZ). LOS RÍOS: Estación Científica Río Palenque, 150–200 m (10 specimens CEMT; 14 specimens MQCAZ). MANABÍ: Ayampe, 35 m (1 specimen MUTPL). MORONA SANTIAGO: Comunidad Ángel Rouby, 1300 m, Cordillera del Kutukú (2 specimens MQCAZ); Comunidad Untsuants sitio 3, Cordillera del Kutukú, 700 m (4 specimens MQCAZ; 6 specimens MECN); Cumpi Cordillera del Kutukú (1 specimen MUTPL); km 8 road to Mendez-Paute, 1250 m (2 specimens MQCAZ). ORELLANA: Daimi (1 specimen MQCAZ); Dayuma Campo Hormiguero, plataforma Hormiguero, 320 m (1 specimen MUTPL); Dayuma plataforma Ungurahua, 300 m (1 specimen MUTPL); El Dorado plataforma Guarango, 300 m (1 specimen MUTPL); Estación Científica Yasuní PUCE, 250 m, Parque Nacional Yasuní (17 specimens MQCAZ); Ines Arango road Tiwino-río Shiripuno, 250 m (1 specimen MUTPL); Rodrigo Borja IAMOE (2 specimens CEMT; 4 specimens MQCAZ); San Sebastian del Coca, 345 m, Comuna Guataraco, Campo Pata (1 specimen MUTPL); Lago San Pedro, plataforma Copal, 310 m (1 specimen MUTPL); Río Tiputini Yasuní Res. Stn. (1 specimen CEMT). PASTAZA: Bosque Protector Oglán Alto, 555–990 m (4 specimens MGO-UC); Chuyayacu Oleoducto km 25, 200 m (1 specimen CEMT; 1 specimen MGO-UC); Estación Biológica Pindo Mirador UTE, 1100 m (1 specimen MUTPL); plataforma Villano (3 specimens MQCAZ). PICHINCHA: Curipoglio, 1000 m, Río Guayllabamba (1 specimen MUTPL); Guayabilla Río Guayllabamba, 520 m, Manduriacus (2 specimens MGO-UC); Llurimaguas Río Guayllabamba, 290 m, Pedro Vicente Maldonado (1 specimen MUTPL); Tortugo Río Guayllabamba, 450 m, Pedro Vicente Maldonado (1 specimen MUTPL); San Roque Río Guayllabamba, 580 m, Pedro Vicente Maldonado (4 specimens MGO-UC; 1 specimen MUTPL). SANTA ELENA: Olón, 50 m (23 specimens CEMT). SANTO DOMINGO DE LOS TSÁCHILAS: Tinalandia-Santo Domingo (2 specimens MQCAZ); Santo Domingo, Puerto Limon, 400 m (1 specimen CEMT). SUCUMBÍOS: 6 km de Dureno, 305 m, Precooperativa Los Vergeles (2 specimen MGO-UC); Aucayacu Río El Eno, 16 km de Lago Agrio, 275 m (4 specimen MGO-UC); Bermejo plataforma ER-A road to Lumbaqui (1 specimen MUTPL); Pacayacu, 260 m, Campo Libertador Tapi (1 specimen MGO-UC); Shusufindi, Recinto la Pantera 250 m (1 specimen CEMT).

###### Literature records.

EL ORO: Piñas, 1200 m (Génier 2009: 177). ESMERALDAS: Colón del Ónzole (Génier 2009: 177); Estación Forestal la Chiquita, 5 m, 11 km SE San Lorenzo (Génier 2009: 177); Gualpí del Ónzole (Génier 2009: 177); Majua (Génier 2009: 177); Pajonal (Génier 2009: 177); Palma Real (Génier 2009: 177); Playa de Oro (Génier 2009: 177); Playa Rica (Génier 2009: 177); Prov. San Mateo (Génier 2009: 177); Salto del Bravo (Génier 2009: 177); Tsejpi (Génier 2009: 177). GUAYAS: Bucay (Génier 2009: 177). LOS RÍOS: 47 km S Santo Domingo, 213 m (Génier 2009: 178); 57 km N Quevedo (Génier 2009: 178); Estación Científica Río Palenque, 47 km S Santo Domingo, 250 m (Génier 2009: 178); Estación Experimental Tropical Pichilingue, Quevedo (Génier 2009: 178); Río Palenque (Génier 2009: 178). MANABÍ: 20 km N Chone, 300 m (Génier 2009: 178); 78 km NE Chone, 85 km WSW Santo Domingo, 450 m (Génier 2009: 178); 90 km WSW Santo Domingo, 73 km NE Chone, 300 m (Génier 2009: 178). MORONA SANTIAGO: Ángel Rouby, sitio 8, 1300 m (Génier 2009: 178); road Mendez-Paute km 8, 1250 m (Génier 2009: 178). NAPO: 11.5 km SW Tena (Génier 2009: 178); 13 km SW Tena (Génier 2009: 178); 17 km SW Tena (Génier 2009: 178); 5 km O del Tena, 500 m (Génier 2009: 178); Estación Biológica Jatun Sacha, 450 m (Génier 2009: 178); Estación Biológica Jatun Sacha, 21 km NE de Puerto Napo, 400 m (Génier 2009: 179); Misahualli Jungle Lodge, area junction of Río Napo-Río Misahualli (Génier 2009: 179); Tena, 400–500 m (Génier 2009: 179). NAPO [= ORELLANA]: Scyasuní, 200 m (Génier 2009: 179). NAPO [= SUCUMBÍOS]: Río Aguarico, 150 m (Génier 2009: 179); Río Napo-Río Aguarico (Génier 2009: 179). ORELLANA: Estación Científica Yasuní PUCE, 250 m (Génier 2009: 179); Estación de Biodiversidad Tiputini USFQ, Parque Nacional Yasuní, 220 m (Génier 2009: 179). PASTAZA: Llandia 17 km N Puyo, 1000 m (Génier 2009: 179); plataforma Villano (Génier 2009: 179). PICHINCHA: Pachijal, 600 m (Génier 2009: 179). PICHINCHA [= SANTO DOMINGO DE LOS TSÁCHILAS]: 4 km SE Santo Domingo, 500 m (Génier 2009: 179); Tinalandia, 16 km SE de Santo Domingo, 600 m (Génier 2009: 179); Tinalandia, Santo Domingo (Génier 2009: 179). SUCUMBÍOS: 2 km N Limoncocha, 250 m (Génier 2009: 179); 30 km E Lago Agrio, road to Tarapoa (Génier 2009: 179); Dureno, Río Aguarico, 150 m (Génier 2009: 179); La Selva Lodge, 150 km down Río Napo from Coca (Génier 2009: 179); Limoncocha, 250 m (Génier 2009: 179); Lombaqui [= Lumbaqui], 800 m (Génier 2009: 180); Sacha Lodge, 270 m (Génier 2009: 180); Santa Cecilia (Génier 2009: 180).

###### Temporal data.

Collected every month of the year.

###### Remarks.

Inhabits coastal lowland evergreen forests and coastal evergreen foothill forests from 5–1200 m a.s.l. In the Amazon, it was recorded in lowland evergreen forests and foothill evergreen forests from 150–1250 m a.s.l. Collected with flight interception traps and pitfall traps baited with carrion and human feces.

##### Eurysternus squamosus

Taxon classificationAnimaliaColeopteraScarabaeidae

Génier, 2009

Plate 31D

Eurysternus squamosus Génier, 2009: 67 (original description. Type locality: Peru, Loreto. Campamento San Jacinto 175–215 m).
Eurysternus squamosus : Camero 2010: 149 (characters in key), 150 (diagnosis); Krajcik 2012: 107 (complete list of species); Ratcliffe et al. 2015: 195 (cited for Peru); Chamorro et al. 2018: 95 (cited for Ecuador).

###### Type specimens.

*Eurysternussquamosus* Génier, 2009. The holotype (♂) is deposited at the CMNC (see Génier 2009: 68). Locality: Peru, Loreto. Campamento San Jacinto 175–215 m, not examined.

###### Distribution.

Colombia, Ecuador, and Peru.

###### Literature records.

SUCUMBÍOS: Río Napo-río Aguarico (Génier 2009: 69).

###### Temporal data.

Collected in September-October.

###### Remarks.

Inhabits the lowland evergreen forests of the Amazon region at 200 m a.s.l. The collection method for this species is unknown. However, Génier (2009) indicated that this species was collected with flight interception traps and pitfall traps baited with human feces.

##### Eurysternus streblus

Taxon classificationAnimaliaColeopteraScarabaeidae

Génier, 2009

Plate 32A

Eurysternus streblus Génier, 2009: 159 (original description. Type locality: Ecuador, Pichincha, 5.3 km on road Pachijal, 2800–3000 m).
Eurysternus streblus : Camero 2010: 149 (characters in key), 155 (diagnosis); Krajcik 2012: 107 (complete list of species); Ratcliffe et al. 2015: 195 (cited for Peru); Chamorro et al. 2018: 95 (cited for Ecuador).

###### Type specimens.

*Eurysternusstreblus* Génier, 2009. The holotype (♂) is deposited at the CMNC (see Génier 2009: 161). Locality: Ecuador, Pichincha, 5.3 km on road Pachijal, 2800–3000 m, not examined.

###### Distribution.

Colombia, Costa Rica, Ecuador, Panama, and Peru.

###### Records examined.

CARCHI: Tobar Donoso, 300 m (2 specimens MECN). ESMERALDAS: Palma Real (2 specimens MUTPL); Pote, Playa de Oro (1 specimen CEMT; 1 specimen MQCAZ).

###### Literature records.

ESMERALDAS: Cachabé (Génier 2009: 161); Charco Vicente (Génier 2009: 161); Padre Santo, Playa de Oro (Génier 2009: 161); Playa Rica (Génier 2009: 161); Pote, Playa de Oro, 200 m (Génier 2009: 161); Salidero, 107 m (Génier 2009: 162). PICHINCHA: 5.3 km on road Pachijal, 2800–3000 m (Génier 2009: 162); km 5 on road Pachijal, 109 km NW de Quito, 915 m (Génier 2009: 162).

###### Temporal data.

Collected in February, March, May, August, November, and December.

###### Remarks.

Inhabits coastal lowland evergreen forests and coastal evergreen foothill forests from 200–915 m a.s.l. In the Andean region, this species has been collected in the montane cloud forests from 2800–3000 m a.s.l. Collected with pitfall traps baited with carrion and human feces According to our data *E.streblus* is distributed in the coastal region. Therefore, Génier’s (2009) altitudinal records might be erroneous.

##### Eurysternus strigilatus

Taxon classificationAnimaliaColeopteraScarabaeidae

Génier, 2009

Plate 32B

Eurysternus strigilatus Génier, 2009: 74 (original description. Type locality: Peru, Madre de Dios, 15 km NE Puerto Maldonado, Reserva Cuzco Amazónica, 200 m).
Eurysternus strigilatus : Krajcik 2012: 107 (complete list of species); Ratcliffe et al. 2015: 195 (cited for Peru); Chamorro et al. 2018: 95 (cited for Ecuador).

###### Type specimens.

*Eurysternusstrigilatus* Génier, 2009. The holotype (♂) is deposited at the CMNC (see Génier 2009: 75). Locality: Peru, Madre de Dios, 15 km NE Puerto Maldonado, Reserva Cuzco Amazónica, 200 m, not examined.

###### Distribution.

Brazil, Ecuador, and Peru.

###### Records examined.

PASTAZA: Chuyayaco Oleoducto km 25, 200 m (2 specimens CEMT).

###### Temporal data.

Collected in May.

###### Remarks.

Inhabits the lowland evergreen forests of the Amazon region at 200 m a.s.l. Collected with pitfall traps baited with human feces.

##### Eurysternus vastiorum

Taxon classificationAnimaliaColeopteraScarabaeidae

Martínez, 1988

Plate 32C

Eurysternus (Eurysternus) vastiorum Martínez, 1988a: 287 (original description. Type locality: Peru, departamento de Huánuco, Tingo María).
Eurysternus (Eurysternus) s. str.vastiorum : Vaz-de-Mello 2000: 193 (cited for Brazil).
Eurysternus vastiorum : Celi et al. 2004: 45 (cited for Ecuador); Hamel-Leigue et al. 2006: 16 (cited for Bolivia); Génier 2009: 37 (diagnosis), 280 (characters in key); Camero 2010: 150 (characters in key), 160 (diagnosis, distribution for Colombia); Carvajal et al. 2011: 314–315 (cited for Ecuador); Krajcik 2012: 107 (complete list of species); Ratcliffe et al. 2015: 195 (cited for Peru); Chamorro et al. 2018: 95 (cited for Ecuador).

###### Type specimens.

Eurysternus (Eurysternus) vastiorum Martínez, 1988. The holotype (♂) is deposited at the MACN (see Génier 2009: 37). Locality: Peru, D° Huánuco, Tingo María, not examined.

###### Distribution.

Bolivia, Brazil, Colombia, Ecuador, French Guiana, Peru, and Suriname.

###### Records examined.

MORONA SANTIAGO: Comunidad Unsuants, 500–1100 m, Cordillera del Kutukú (3 specimens MQCAZ). NAPO: Puerto Misahuallí, 350 m (1 specimen MQCAZ). ORELLANA: Comunidad Kiwcha Chiruisla Station, 180–250 m (2 specimens MQCAZ); Dayuma, Campo Hormiguero, plataforma Hormiguero, 320 m (2 specimens MUTPL); Dayuma, plataforma Ungurahua, 300 m (1 specimen MUTPL); El Dorado plataforma Guarango, 300 m (1 specimen MUTPL); Lago San Pedro, plataforma Copal, 310 m (1 specimen MUTPL). PASTAZA: Bosque Protector Oglán Alto, 610–890 m (2 specimens MUTPL). SUCUMBÍOS: Limoncocha (1 specimen CEMT; 1 specimen MUTPL); Reserva de Producción Faunística Cuyabeno Brazo del río Guepi (1 specimen MUTPL).

###### Literature records.

MORONA SANTIAGO: Bosque Domono, 1650 m (Génier 2009: 39); road Mendez-Paute km 8, 1250 m (Génier 2009: 39). NAPO: 20 km S Tena, 600 m (Génier 2009: 39); 3.3 km E Puerto Napo, 400 m (Génier 2009: 39); 5 km O Tena, 500 m (Génier 2009: 39); Estación Científica Yasuní PUCE, 250 m; Parque Nacional Yasuní (5 specimens MQCAZ); Estación Biológica Jatun Sacha, 450 m (Génier 2009: 39); Estación Biológica Jatun Sacha, 21 km E de Puerto Napo, 400 m (Génier 2009: 39); Hostería Misahuallí, Jungle Lodge (Génier 2009: 39); Misahuallí, Jungle Lodge area, juntion of Río Napo-Río Misahuallí, 579 m (Génier 2009: 39); Tena, 400–500 m (Génier 2009: 39). NAPO [= SUCUMBÍOS]: Río Aguarico, 150 m (Génier 2009: 39). ORELLANA: Estación Científica Yasuní PUCE, 250 m (Génier 2009: 39); Estación de Biodiversidad Tiputini USFQ, Parque Nacional Yasuní, 220 m (Génier 2009: 39); Yuturi Lodge (Génier 2009: 39). PASTAZA: Villano (Génier 2009: 39). SUCUMBÍOS: 2 km N Limoncocha, 250 m (Génier 2009: 39); 30 km E Lago Agrio, road to Tarapoa (Génier 2009: 39); Limoncocha, 250 m (Génier 2009: 39); Sacha Logde, 270 m (Génier 2009: 40). ZAMORA CHINCHIPE: road Cumbaritza-Gualaquiza km 1, 1100 m (Génier 2009: 40); road Namirez-Zamora km 1, 1000 m (Génier 2009: 40); road Zumbi-Yantzaga km 4, 900 m (Génier 2009: 40).

###### Temporal data.

Collected in February, March, June, July, August, September, October, and November.

###### Remarks.

Inhabits the lowland evergreen forests and evergreen foothill forests of the Amazon region from 150–1250 m a.s.l. Collected with flight interception traps, beat-sheet collecting method, and pitfall traps baited with carrion and human feces.

##### Eurysternus wittmerorum

Taxon classificationAnimaliaColeopteraScarabaeidae

Martínez, 1988

Plate 32D

Eurysternus wittmerorum Martínez, 1988a: 284 (original description. Type locality: Ecuador, provincia de Napo, Lago Agrio [= currently provincia de Sucumbíos], 250 m).
Eurysternus (Eurysternus) s. str.wittmerorum : Vaz-de-Mello 2000: 193 (cited for Brazil).
Eurysternus wittmerorum : Génier 2009: 56 (diagnosis), 281 (characters in key); Camero 2010: 150 (characters in key), 159 (diagnosis); Carvajal et al. 2011: 314–315 (cited for Ecuador); Krajcik 2012: 107 (complete list of species); Ratcliffe et al. 2015: 195 (cited for Peru); Chamorro et al. 2018: 78 (figure 1B), 95 (cited for Ecuador).

###### Type specimens.

*Eurysternuswittmerorum* Martínez, 1988. The holotype (♂) is deposited at the MACN (see Génier 2009: 56). Locality: Ecuador, provincia de Napo, Lago Agrio 250 m, not examined.

###### Distribution.

Bolivia, Brazil, Colombia, Ecuador, French Guiana, Peru, and Surinam.

###### Records examined.

ORELLANA: Bloque 31 Parque Nacional Yasuní, 200 m (1 specimen MECN); Comunidad Kiwcha Chiruisla Station, 180–250 m (3 specimens MQCAZ); Daimi 1 (2 specimens CEMT; 3 specimens MQCAZ); Estación Cientifica Yasuní, 200 m, Parque Nacional Yasuní (28 specimens MQCAZ); El Dorado, plataforma Guarango, 300 m (1 specimen MUTPL); Estación de Biodiversidad Tiputini USFQ, 220 m (5 specimens MUTPL); Ines Arango road Tiwino-río Shiripuno, 250 m (1 specimen MUTPL); San Sebastian del Coca, Comuna Guataraco Campo Pata, 345 m (1 specimen MUTPL); San Sebastian del Coca, Comuna Shamanal Campo Palo Azul, 345 m (1 specimen MGO-UC); Lago San Pedro, plataforma Copal, 310 m (1 specimen MUTPL); SC Yasuní (1 specimen CEMT; 4 specimen MQCAZ); Yampuna (2 specimens MQCAZ); Yuturi (1 specimen MQCAZ). PASTAZA: Bosque Protector Oglán Alto, 660–810 m (1 specimen MUTPL). SUCUMBÍOS: Cascales, 400 m, Pozo Ruby 1 (1 specimen MUTPL); La Selva Bio Station 175 km E.S.E del Coca (2 specimens MQCAZ); Trocha Zábalo-Guepi km 10, Reserva de Producción Faunística Cuyabeno (1 specimen MUTPL); Tarapoa Campo Marian, 260 m, plataforma Fanny 5 (1 specimen MUTPL).

###### Literature records.

NAPO [= ORELLANA]: SCyasuni, 200 m (Génier 2009: 57). ORELLANA: Estación Cientifica Yasuní, PUCE, 250 m (Génier 2009: 57); Estación de Biodiversidad Tiputini USFQ, Parque Nacional Yasuní, 220 m (Génier 2009: 58). SUCUMBÍOS: 2 km N de Limoncocha, 250 m (Génier 2009: 58); Dureno Río Aguarico, 150 m (Génier 2009: 58); Limoncocha, 250 m (Génier 2009: 58); Lago Agrio, 250 m (Martínez 1988a: 286).

###### Temporal data.

Collected in all months except December.

###### Remarks.

Inhabits the lowland evergreen forests and evergreen foothill forests of the Amazon region from 150–810 m a.s.l. Collected with flight interception traps and pitfall traps baited with carrion and human feces.

#### Genus *Eutrichillum* Martínez, 1968

Trichillum (Eutrichillum) Martínez, 1968: 121 (original description. Type species: *Trichillumboucomonti* Saylor, 1935 = *Trichillumhirsutum* Boucomont, 1928 (original designation) = *Eutrichillumhirsutum* Boucomont, 1928), see Vaz-de-Mello 2008: 22).

Trichillum (Eutrichillum): Martínez 1967 [sic]: Ratcliffe 1980: 340 (characters in key); Halffter and Edmonds 1982: 137 (cited as subgenus Eutrichillum 1967); Vaz-de-Mello 2000: 193 (list of species from Brazil, cited as subgenus Eutrichillum 1967).

*Eutrichillum*: Vaz-de-Mello 2008: 22 (cited as new status, redescription, distribution); Vaz-de-Mello et al. 2011: 22 (characters in key); Carvajal et al. 2011: 316 (cited for Ecuador); Solís and Kohlmann 2012: 5 (list of species from Costa Rica); Boilly and Vaz-de-Mello 2013: 109 (characters in key); Chamorro et al. 2018: 74 (characters in key), 79 (figures 2F and 2H), 95 (cited for Ecuador).

**Remarks.** Throughout our survey we identified a new species from Orellana and Sucumbíos provinces. However, its description will be provided in a future work.

#### Genus *Gromphas* Brullé, 1837

Copris (Gromphas) Brullé, 1837: 283, 298 and 304 (original description. Type species: *Onitisaeruginosus* Perty, 1830. Secondary monotypy by Sturm [= 1843]. See Cupello and Vaz-de-Mello 2014: 399).

*Gromphas*: Agassiz 1846: 481 (catalog); Blanchard 1846: 181 (redescription); Lacordaire 1856: 100 (redescription); Gemminger and Harold 1869: 1016 (catalog); Lacordaire and Chapuis 1876: 276 (catalog); Gillet 1911a: 80 (catalog); Lucas 1920: 309 (catalog, distribution); d’Olsoufieff 1924: 17 (characters in key); Pessôa and Lane 1941: 470 (characters in key); Blackwelder 1944: 208 (list of species from Latin America); Roze 1955: 45 (list of species from Venezuela); Martínez 1959: 95 (list of species from Argentina); Barattini and Saenz 1961: 21 (comment); 1964: 173 (comment); Halffter and Matthews 1966: 257 (catalog, distribution); Vulcano and Pereira 1967: 565 (characters in key); Edmonds 1972: 816 (comment); Halffter and Edmonds 1982: 137 (catalog, distribution); Zunino 1985: 104 (comment); Medina and Lopera 2000: 305 (characters in key); Vaz-de-Mello 2000: 193 (list of species from Brazil); Medina et al. 2001: 138 (list of species from Colombia); Philips et al. 2004: 50 (comment); Hamel-Leigue et al. 2006: 17 (list of species from Bolivia); Scholtz et al. 2009: 246 (evolutionary history); Bouchard et al. 2011: 245 (genotype of Gromphina Zunino, 1985); Vaz-de-Mello et al. 2011: 24 (characters in key); Krajcik 2012: 117 (complete list of species); Figueroa et al. 2012: 2 (redescription); Cupello and Vaz-de-Mello 2013b: 443 (revision); Cupello and Vaz-de-Mello 2014: 399 (comment); Figueroa et al. 2014: 137 (distributional records from Peru); Cupello and Vaz-de-Mello 2015: 3 (characters in key), 11 (distribution, figure 5); Chamorro et al. 2018: 75 (characters in key), 95 (list of species from Ecuador).

##### Gromphas aeruginosa

Taxon classificationAnimaliaColeopteraScarabaeidae

(Perty, 1830)

Plate 33A

Onitis aeruginosus Perty, 1830: 39 (original description. Type locality: Habitat in mediterraneis Prov. S. Pauli et Minarum [= South America]).
Onitis aeruginosus : Scherer 1983: 298 (designation of lectotype); Cupello 2013: 15–17 (comments on the homonymy of Onitisaeruginosus Perty, 1830).
Gromphas (onitis) aeruginosa : Harold 1859: 199 (transferred to the genus Gromphas Brullé, 1837).
Gromphas aeruginosa : Gemminger and Harold 1869: 1016 (catalog, distribution); Harold 1869d: 62 (comment); Heyne and Taschenberg 1908: 64 (redescription, distribution); Gillet 1911a: 80 (catalog, distribution); d’Olsoufieff 1924: 20 (characters in key), 58 (redescription, distribution); Blackwelder 1944: 208 (list of species from Latin America); Barattini and Saenz 1961: 23 (redescription); 1964: 177 (redescription); Vulcano and Pereira 1967: 566 (characters in key); Vaz-de-Mello 2000: 193 (cited for Brazil); Medina et al. 2001: 138 (cited for Colombia); Hamel-Leigue et al. 2006: 17 (cited for Bolivia); Hamel-Leigue et al. 2009: 61 (distribution of records from Bolivia); Krajcik 2012: 117 (complete list of species); Figueroa et al. 2012: 3 (redescription); Cupello 2013: 15 (comment); Cupello and Vaz-de-Mello 2013b: 447 (characters in key); 448 (redescription); Figueroa et al. 2014: 137 (distribution of records from Peru); Ratcliffe et al. 2015: 197 (cited for Peru); Chamorro et al. 2018: 82 (figure 5F), 83 (figure 6A), 96 (cited for Ecuador).
Gromphas lacordairei Blanchard, 1846: 181 (original description); Harold 1859: 199 (synonym of Gromphasaeruginosa Perty, comment); Gemminger and Harold 1869: 1016 (cited as synonym of Gromphasaeruginosa Perty, cited as GromphasLacordairei Dej.); Harold 1869d: 62 (comment); Gillet 1911a: 80 (cited as synonym of Gromphasaeruginosa Perty, cited as GromphasLacordairei); Burmeister 1874b: 130 (redescription); d’Olsoufieff 1924: 138 (cited as synonym of Gromphasaeruginosa Perty, 1830); Blackwelder 1944: 208 (cited as synonym of Gromphasaeruginosa Perty); Barattini and Saenz 1961: 23 (cited as synonym of Gromphasaeruginosa Perty), 1964: 177 (cited as synonym of Gromphasaeruginosa Perty); Hamel-Leigue et al. 2009: 61 (cited as synonym of Gromphasaeruginosa Perty); Cupello and Vaz-de-Mello 2013b: 448 (cited as synonym of Gromphasaeruginosa Perty); Figueroa et al. 2014: 137 (cited as synonym of Gromphasaeruginosa Perty, 1830); Cupello and Vaz-de-Mello 2015: 11 (distribution, figure 5).

###### Type specimens.

*Onitisaeruginosus* Perty, 1830. The lectotype is deposited at the ZSM (see Scherer 1983: 298). Locality: Brasilien [= Brazil], examined.

**Lectotype** (sex unknown): “Brasilien [p]”, “3 – 8. / Typi. [p]”, “Type von / gromphas / aeruginosus / Perty. [p and hw, red label]”, “alte / sammlung [p]”, “HOLOTYPUS / Onitis Perty / aeruginosus / det. Dr. G. Scherer 1981 [p and hw, red margin]”, “Gromphas / aeruginosus / (Perty) / det. G. Scherer 1981 [p and hw]”.

*Gromphaslacordairei* Blanchard, 1846. Type material not examined. Syntypes possibly deposited at the MNHN (see Cupello and Vaz-de-Mello 2013b: 448) not examined.

###### Distribution.

Bolivia, Brazil, Colombia, Ecuador, and Peru.

###### Records examined.

ORELLANA: Estación Chiruisla, 215 m (1 specimen MQCAZ).

###### Literature records.

NAPO [= ORELLANA]: Río Coca (Cupello and Vaz-de-Mello 2013b: 452).

###### Temporal data.

Collected in October and September.

###### Remarks.

Inhabits the lowland evergreen forests of the Amazon region at 215 m a.s.l. Collected manually at night.

#### Genus *Homocopris* Burmeister, 1846

Copris (Homocopris) Burmeister, 1846: 77 (original description. Type species: *Copristorulosus* Eschscholtz, 1822 for monotypy. See Vaz-de-Mello et al. 2010: 192).

*Pinotus* Erichson, 1847 [= *Homocopris* Burmeister, 1846]: Gemminger and Harold 1869: 1009 (cited as synonym of *Pinotus* Hope, 1838); Gillet 1911a: 59 (cited as synonym of *Pinotus* Erichson, 1847); Lucas 1920: 333 (cited as synonym of *Pinotus* Erichson, 1847); Blackwelder 1944: 206 (cited as synonym of *Pinotus* Erichson, 1847).

*Dichotomius* Hope, 1838 [= *Homocopris* Burmeister, 1846]: Martínez 1951b: 140 (cited as synonym of *Dichotomius* Hope, 1838); Martínez 1959: 80 (cited as synonym of *Dichotomius* Hope, 1838); Krajcik 2012: 91 (cited as synonym of *Dichotomius* Hope, 1838); Sarmiento-Garcés and Amat-García 2014: 23 (cited as synonym of *Dichotomius* Hope, 1838).

*Homocopris*Burmeister 1846: Vaz-de-Mello et al. 2010: 192 (cited as new status, comment); Vaz-de-Mello et al. 2011: 28 (characters in key); Chamorro et al. 2018: 77 (characters in key), 96 (list of species from Ecuador).

##### Homocopris achamas

Taxon classificationAnimaliaColeopteraScarabaeidae

(Harold, 1867)

Plate 33B

Pinotus achamas Harold, 1867e: 99 (original description. Type locality: Columbia).
Pinotus achamas : Harold 1869c: 130 (redescription, written as PinotusAchamas); Gemminger and Harold 1869: 1009 (complete list of species, written as PinotusAchamas); Harold 1880a: 24 (distribution, written as PinotusAchamas); Gillet 1911a: 59 (complete list of species, written as PinotusAchamas); Luederwaldt 1929: 32 (characters in key, written as PinotusAchamas); Blackwelder 1944: 206 (list of species from Latin America); Contreras 1951: 222 (cited for Colombia).
Pinotus (Pinotus) achamas : Luederwaldt 1936: 207 (redescription).
Dichotomius achamas : Medina et al. 2001: 138 (cited for Colombia); Carvajal et al. 2011: 320–321 (cited for Ecuador); Krajcik 2012: 91 (complete list of species).
Homocopris achamas : Vaz-de-Mello et al. 2010: 192 (cited as new combination, comment); Chamorro et al. 2018: 90 (figure 13G), 96 (cited for Ecuador).

###### Type specimens.

*Pinotusachamas* Harold, 1867. One syntype examined deposited at the MNHN. Lectotype to be designated in a future work on this species group.

###### Distribution.

Colombia and Ecuador.

###### Records examined.

CARCHI: El Angel (1 specimen MQCAZ); Guanderas Estación Cientifica Jatun Sacha, 3280 m (1 specimen CEMT); Mariscal Sucre, La Bretaña (1 specimen MEPN). CHIMBORAZO: Riobamba, 2755 m (1 specimen MQCAZ).

###### Temporal data.

Collected in June and December.

###### Remarks.

Inhabits evergreen high montane forests of the Andean region from 2150–3100 m a.s.l. Collected at light and manually.

##### Homocopris buckleyi

Taxon classificationAnimaliaColeopteraScarabaeidae

(Waterhouse, 1891)

Plate 33C

Pinotus buckleyi Waterhouse, 1891a: 359 (original description. Type locality: Ecuador, Chiguinda).
Pinotus buckleyi : Gillet 1911a: 59 (complete list of species, written as PinotusBuckleyi); Luederwaldt 1929: 50 (characters in key, written as PinotusBuckleyi); Blackwelder 1944: 206 (list of species from Latin America).
Pinotus (Pinotus) buckleyi : Luederwaldt 1936: 208 (redescription).
Dichotomius buckleyi : Carvajal et al. 2011: 320–321 (cited for Ecuador); Krajcik 2012: 91 (complete list of species).
Homocopris buckleyi : Vaz-de-Mello et al. 2010: 192 (cited as new combination, comment); Chamorro et al. 2018: 96 (cited for Ecuador).

###### Type specimens.

*Pinotusbuckleyi* Waterhouse, 1891. The holotype (♂) is deposited at the NHML. Locality: Chiguinda, examined.

**Holotype** (♂): “Chiquin / -da / 80. 14 [hw]”, “Type [p, red margin]”, “Pinotus / Buckleyi, / (Type) Waterh. [hw]”.

###### Distribution.

Ecuador and Peru.

###### Records examined.

LOJA: Amaluza, Angashcola, 2740 m (37 specimens MUTPL); Saraguro, Huashapamba, 2920 m (5 specimens CEMT; 2 specimens MUTPL).

###### Literature records.

LOJA [= ZAMORA CHINCHIPE]: Andes, Sabonilla [= Sabanilla, El Tambo] (Luederwaldt, 1936: 209). MORONA SANTIAGO: Chiguinda [= Chigüinda] (Waterhouse, 1891a: 359).

###### Temporal data.

Collected in October and December.

###### Remarks.

Inhabits the montane cloud forests and the evergreen high montane forests of the Andean region from 2150–3100 m a.s.l. Collected with pitfall traps baited with human feces.

#### Genus *Malagoniella* Martínez, 1961

*Malagoniella* Martínez, 1961: 82 (original description. Type species: *Megatophaargentina* Gillet, 1911).

*Malagoniella*: Vulcano and Pereira 1964: 574 (catalog of species); Halffter and Matthews 1966: 260 (catalog, distribution); Halffter and Martínez 1966: 114 (diagnosis); Vulcano and Pereira 1967: 547 (characters in key); Halffter and Martínez 1977: 33 (characters in key); Halffter and Edmonds 1982: 139 (catalog, distribution); Medina and Lopera 2000: 301 (characters in key); Vaz-de-Mello 2000: 194 (list of species from Brazil); Medina et al. 2001: 137 (list of species from Colombia); Arnett et al. 2002: 49 (characters in key); Morón 2003: 23 (redescription); Hamel-Leigue et al. 2006: 14 (list of species from Bolivia); Vaz-de-Mello et al. 2011: 21 (characters in key); Carvajal et al. 2011: 120 (diagnosis); Krajcik 2012: 156 (complete list of species); Solís and Kohlmann 2012: 4 (list of species from Costa Rica); Chamorro et al. 2018: 73 (characters in key), 96 (list of species from Ecuador).

##### Subgenus Malagoniella (Malagoniella) Martínez, 1961

Malagoniella (Malagoniella) s. str. Martínez, 1961: 82 (original description. Type species: *Megatophaargentina* Gillet, 1911 original combination); Halffter and Matthews 1966: 260 (cited as subgenus of *Malagoniella* Martínez); Halffter and Martínez 1966: 116 (redescription, characters in key); Halffter and Martínez 1977: 33 (characters in key); Halffter and Edmonds 1982: 139 (catalog, distribution); Vaz-de-Mello 2000: 194 (list of species from Brazil); Vaz-de-Mello et al. 2011: 21 (characters in key); Boilly and Vaz-de-Mello 2013: 106 (characters in key); Chamorro et al. 2018: 73 (characters in key), 96 (list of species from Ecuador).

###### Malagoniella (Malagoniella) astyanaxpolita

Taxon classificationAnimaliaColeopteraScarabaeidae

Halffter, Pereira & Martínez, 1960

Plate 33D

Megatopha astyanax polita Halffter, Pereira & Martínez, 1960: 203 (original description. Type locality: Bolivia: Departamento de la Paz, Provincia de Sud Yungas, Chulumani, 1800–2000 m).
Malagoniella astyanax polita : Martínez 1961: 83 (cited as new combination); Vulcano and Pereira 1964: 575 (cited as subespecie); Halffter and Martínez 1966: 117 (characters in key), 124 (distribution); Hamel-Leigue et al. 2006: 14 (cited for Bolivia); Carvajal et al. 2011: 316–317 (cited for Ecuador); Krajcik, 2012: 156 (cited as subspecies); Chamorro et al. 2018: 78 (figures 1C and 1F), 96 (cited for Ecuador).

####### Type specimens.

*Megatophaastyanaxpolita* Halffter, Pereira & Martínez, 1960. The holotype (♀) is deposited at the AMIC (Halffter, Pereira and Martínez 1960: 203) [= name-bearing types now at the MACN]. Locality: Bolivia: Departamento de la Paz, Provincia de Sud Yungas, Chulumani, 1800–2000 m, not examined.

####### Distribution.

Brazil, Colombia, Ecuador, French Guiana, Surinam, and Venezuela.

####### Records examined.

ORELLANA: Eden, Campo Eden plataforma G, 220 m (1 specimen CEMT); San Sebastian del Coca, Comuna Guataraco Campo Pata, 345 m (2 specimens MUTPL); San Sebastian del Coca, Comuna Shamanal Campo Palo Azul, 345 m (1 specimen CEMT); Lago San Pedro, plataforma Copal, 310 m (1 specimen MUTPL). SUCUMBÍOS: La Selva Bio Station 175 km E.S.E del Coca (2 specimens MQCAZ).

####### Temporal data.

Collected in March, May, August, and November.

####### Remarks.

Inhabits the lowland evergreen forests of the Amazon region from 220–345 m a.s.l. Collected with pitfall traps baited with human feces.

##### Subgenus Malagoniella (Megathopomima) Martínez, 1961

Malagoniella (Megathopomima) Martínez, 1961: 84 (original description. Type species: *Coprobiusbicolor* Guérin, 1840 original combination); Vulcano and Pereira 1964: 578 (catalog of species); Halffter and Matthews 1966: 260 (cited as subgenus of *Malagoniella* Martínez); Halffter and Martínez 1966: 116 (characters in key); Halffter and Martínez 1977: 33 (characters in key); Halffter and Edmonds 1982: 139 (catalog, distribution); Vaz-de-Mello 2000: 194 (list of species for Brazil); Vaz-de-Mello et al. 2011: 21 (characters in key); Chamorro et al. 2018: 74 (characters in key), 96 (list of species from Ecuador).

###### Malagoniella (Megatophomima) cupreicollis

Taxon classificationAnimaliaColeopteraScarabaeidae

(Waterhouse, 1890)

Plate 34A

Megatopha cupreicollis Waterhouse, 1890b: 412 (original description. Type locality: Peru).
Megatopha cupreicollis : Gillet 1911a: 27 (complete list of species); Balthasar 1941: 341 (cited for Peru); Blackwelder 1944: 198 (list of species for Latin America); Martínez 1950: 266 (distribution); Balthasar 1951: 326 (cited for Peru).
Megatophomina cupreicollis : Martínez 1961: 85 (cited as new combination, distribution); Vulcano and Pereira 1964: 579 (catalog of species, distribution).
Malagoniella (Megatophomima) cupreicollis : Halffter and Martínez 1966: 137 (distribution); Krajcik 2012: 156 (complete list of species); Chamorro et al. 2014a: 740 (cited for Ecuador, figure 1); Chamorro et al. 2018: 78 (figures 1D and 1G), 96 (cited for Ecuador).
Malagoniella cupreicollis : Ratcliffe et al. 2015: 196 (cited for Peru).

####### Type specimens.

*Megatophacupreicollis* Waterhouse, 1890. The holotype is deposited at the NHML. Locality: Peru, examined.

**Holotype** (sex unknown): “Peruvia [hw]”, “877 [p]”, “67.45 [p]”, “Type [p, red margin]”, “Cupricollis / Reich / Peruvia. [hw]”, “Megatopha / cupreicollis, / (Type) Waterh. [hw]”.

####### Distribution.

Ecuador and Peru.

####### Records examined.

LOJA: Amaluza, Angascola, 2741 m (2 specimens CEMT; 8 specimens MUTPL); Catamayo, Alamala, 1380 (4 specimens CEMT; 7 specimens MUTPL); Catamayo, Trapichillo, 1424 m (11 specimens MUTPL).

####### Temporal data.

Collected in February and December.

####### Remarks.

Inhabits the matorral dry montane forests of the Andean region from 1200–1700 m a.s.l. Collected with pitfall traps baited with pig feces.

#### Genus *Megatharsis* Waterhouse, 1891

*Megatharsis* Waterhouse, 1891b: 59 (original description. Type species: *Megatharsisbuckleyi* Waterhouse, 1891 by monotypy).

*Megatharsis*: Gillet 1911a: 88 (complete list of species); Lucas 1920: 398 (catalog, distribution); d’Olsoufieff 1924: 162 (distribution); Blackwelder 1944: 210 (list of species from Latin America); Halffter and Matthews 1966: 258 (catalog, distribution); Vulcano and Pereira 1967: 566 (characters in key); Edmonds 1972: 820 (characters in key), 854 (redescription); Halffter and Edmonds 1982: 137 (catalog, distribution); Arnaud 2002a: 14 (characters in key), 17 (diagnosis); Philips et al. 2004: 46 (figure 4); Gillett et al. 2009: 2 (distribution), 3 (figures 1–9); Vaz-de-Mello et al. 2011: 24 (characters in key); Carvajal et al. 2011: 141 (diagnosis); 322 (list of species from Ecuador); Krajcik 2012: 158 (complete list of species); Figueroa et al. 2014: 137 (cited for Peru); Chamorro et al. 2018: 75 (characters in key), 96 (list of species from Ecuador).

##### Megatharsis buckleyi

Taxon classificationAnimaliaColeopteraScarabaeidae

Waterhouse, 1891

Plate 34B

Megatharsis buckleyi Waterhouse, 1891b: 60 (original description. Type locality: Chiquinda [= Chigüinda]).
Megatharsis buckleyi : Gillet 1911a: 88 (complete list of species); d’Olsoufieff 1924: 162 (distribution); Blackwelder 1944: 210 (list of species of Latin America); Vulcano and Pereira 1967: 575 (distribution); Edmonds 1972: 853 (figures 314–315); Arnaud 2002a: 17 (diagnosis); Philips et al. 2004: 46 (figure 4); Gillett et al. 2009: 2 (distribution), 3 (figures 1–9); Carvajal et al. 2011: 322–323 (cited for Ecuador); Krajcik 2012: 158 (complete list of species); Figueroa et al. 2014: 137 (cited for Peru); Ratcliffe et al. 2015: 197 (cited for Peru); Chamorro et al. 2018: 82 (figure 5D), 96 (cited for Ecuador).

###### Type specimens.

*Megatharsisbuckleyi* Waterhouse, 1891. The holotype (♀) is deposited at the NHML. Locality: Chiguinda, examined.

**Holotype** (♀): “Chiquin / -da / 80.14 [hw]”, “Type [p, red margin]”, “Megatharsis / Buckleyi, / (Type) Waterh. [hw]”, “Megatharsis ♀ / buckleyi wat. / Holotypus. / Canada balsam / M. Zunino ’83 [hw]”.

###### Distribution.

Brazil, Ecuador, and Peru.

###### Records examined.

MORONA SANTIAGO: Chiquinda [= Chigüinda] (1 specimen NHML); Macas (1 specimen CEMT). NAPO: Estación Jatun Sacha, 500 m (1 specimen MEPN). ORELLANA: La Joya de los Sachas, Unión Milagreña, 330 m (1 specimen MECN).

###### Literature records.

ZAMORA CHINCHIPE: Sabanilla, 1900 m (Gillett et al. 2009: 4). MORONA SANTIAGO: Chiquinda [= Chigüinda] (Waterhouse 1891b: 60; Gillett et al. 2009: 4); Macas (Gillett et al. 2009: 2); Env. Macas, Macas-Puyo Road, 15 km N, 1100 m (Gillett et al. 2009: 4). NAPO: environs of Archidona, eastern slopes of the Andes, 640 m (Gillett et al. 2009: 4). ORELLANA: Payamino Research Station, 400 m (Gillett et al. 2009: 2). UNDETERMINED PROVINCE: Oriente Tapizal (Gillett et al. 2009: 4).

###### Temporal data.

Collected in February, June, August, and September.

###### Remarks.

Inhabits the lowland evergreen forests and evergreen foothill forests of the Amazon region from 330–1100 m a.s.l. In the Andean region, this species was recorded in montane cloud forests between 1900–2300 m a.s.l. The majority of specimens were collected manually; however, Gillett et al. (2009) reported that some specimens were collected using flight interception traps.

#### Genus *Onoreidium* Vaz-de-Mello, 2008

*Onoreidium* Vaz-de-Mello, 2008: 37 (original description. Type species: *Trichillumcristatum* Arrow, 1931, by original designation).

*Onoreidium*: Vaz-de-Mello et al. 2011: 22 (characters in key); Carvajal et al. 2011: 133 (diagnosis), 316 (cited for Ecuador); Krajcik 2012: 174 (complete list of species); Solís and Kohlmann 2012: 5 (list of species from Costa Rica); Chamorro et al. 2018: 74 (characters in key), 96 (list of species from Ecuador).

##### Onoreidium cristatum

Taxon classificationAnimaliaColeopteraScarabaeidae

(Arrow, 1931)

Plate 34C

Trichillum cristatum Arrow, 1931: 610 (original description. Type locality: Loja 6600 feet [= 2010 m]; Piscobamba).
Trichillum cristatum : Paulian 1936a: 206 (characters in key); Balthasar 1939b: 18 (characters in key), 22 (distribution); Blackwelder 1944: 204 (list of species from Latin America); Vulcano and Pereira 1967: 578 (characters in key); Martínez 1969: 120 (comment); Bacchus 1978: 101 (catalogue of the types of the species described by Arrow).
Pedaridium cristatum : Génier and Vaz-de-Mello 2002: 191 (redescription).
Onoreidium cristatum : Vaz-de-Mello, 2008: 39 (cited as new combination, distribution), 58 (figure 27); Carvajal et al. 2011: 316–317 (cited for Ecuador); Krajcik 2012: 174 (complete list of species); Ratcliffe et al. 2015: 197 (cited for Peru); Chamorro et al. 2018: 96 (cited for Ecuador).
Pedaridium equatoriensis Ferreira & Galileo, 1993: 14 (original description); Génier and Vaz-de-Mello 2002: 191 (synonym of Pedaridiumcristatum (Arrow, 1931), comment).

###### Type specimens.

*Trichillumcristatum* Arrow, 1931. The lectotype (♂) is deposited at the NHML (see Génier and Vaz-de-Mello 2002: 191). Locality: Ecuador, Piscobamba, not examined.

*Pedaridiumequatoriensis* Ferreira & Galileo, 1993. The holotype (sex unknown) is deposited at the HAHC (ex. coll. H. Howden) (see Ferreira and Galileo 1993: 15) [= name-bearing types now in CMCN]. Locality: Loja: Macará Catacocha 1100 m, not examined.

###### Distribution.

Ecuador and Peru.

###### Records examined.

GUAYAS: Isla Puna (1 specimen CEMT). LOJA: without specific locality (1 specimen CEMT); Río Catamayo, 1500 m (2 specimens CEMT).

###### Literature records.

LOJA: Piscobamba (Vaz-de-Mello 2008: 39); Macará-Catacocha, 1100 m (Vaz-de-Mello 2008: 39); without specific locality, 2200 m (Vaz-de-Mello 2008: 39).

###### Temporal data.

Collected in August.

###### Remarks.

Inhabits lowland semi-deciduous forests and matorral dry montane forests of the Andean region from 1110–2200 m a.s.l. The collection method is unknown.

##### Onoreidium howdeni

Taxon classificationAnimaliaColeopteraScarabaeidae

(Ferreira & Galileo, 1993)

Plate 34D

Pedaridium howdeni Ferreira & Galileo, 1993: 26 (original description. Type locality: EQUADOR. Guayas: Guayaquil).
Onoreidium howdeni : Vaz-de-Mello 2008: 39 (new combination, distribution), 65 (figure 82); Carvajal et al. 2011: 316–317 (cited for Ecuador); Krajcik 2012: 174 (complete list of species); Chamorro et al. 2018: 79 (figure 2E), 80 (figure 3C), 96 (cited for Ecuador).

###### Type specimens.

*Pedaridiumhowdeni* Ferreira & Galileo, 1993. The holotype (♀) is deposited at the CMNC (see Vaz-de-Mello 2008: 39). Locality: Ecuador, Guayas: 40 km SW Guayaquil, 50 m, not examined.

###### Distribution.

Only known from Ecuador.

###### Records examined.

GUAYAS: Guayaquil (2 specimens CEMT). EL ORO: Arenillas, 15 m (10 specimens CEMT). SANTA ELENA: 45 km W Guayaquil (2 specimens CEMT).

###### Literature records.

GUAYAS: 40 km SW Guayaquil 50 m (Vaz-de-Mello 2008: 39).

###### Temporal data.

Collected in January, February, March, and June

###### Remarks.

Inhabits coastal lowland semi-deciduous forests and coastal lowland dry scrub from 15–50 m a.s.l. Collected with pitfall traps baited with carrion and human feces.

##### Onoreidium ohausi

Taxon classificationAnimaliaColeopteraScarabaeidae

(Arrow, 1931)

Plate 35A

Trichillum ohausi Arrow, 1931: 610 (original description. Type locality: Loja Punzara, Calvario 6600 feet [= 2010 m]; Piscobamba).
Trichillum ohausi : Paulian 1936a: 206 (characters in key); Balthasar 1939b: 18 (characters in key), 22 (distribution); Blackwelder 1944: 204 (list of species of Latin America); Vulcano and Pereira 1967: 578 (characters in key); Bachus 1978: 106 (catalog of types of species described by Arrow).
Pedaridium ohausi : Martínez 1969: 119 (transferred to the genus Pedaridium Harold, 1868); Ferreira and Galileo 1993: 7 (characters in key); 12 (redescription); Génier and Vaz-de-Mello 2002: 190 (diagnosis).
Onoreidium ohausi : Vaz-de-Mello 2008: 39 (new combination, distribution); Carvajal et al. 2011: 316–317 (cited for Ecuador); Krajcik 2012: 174 (complete list of species); Chamorro et al. 2018: 96 (cited for Ecuador).

###### Type specimens.

*Trichillumohausi* Arrow, 1931. The lectotype (♂) is deposited at the NHML (see Génier and Vaz-de-Mello 2002: 190). Locality: Loja, Punzara Ecuador, not examined.

###### Distribution.

Only known from Ecuador.

###### Records examined.

LOJA: without specific locality (3 specimens CEMT).

###### Literature records.

LOJA: Punzara (Bachus 1978: 106; Ferreira and Galileo 1993: 13; Vaz-de-Mello 2008: 39); El Calvario (Bachus 1978: 106: Ferreira and Galileo 1993: 13; Vaz-de-Mello 2008: 39); Piscobamba (Ferreira and Galileo 1993: 13).

###### Temporal data.

Collected in March, August, and December.

###### Remarks.

Inhabits matorral dry montane forests in the Andean region at 2010 m a.s.l. The collection method is unknown.

#### Genus *Ontherus* Erichson, 1847

*Ontherus* Erichson, 1847: 107 (original description. Type species: *Scarabaeussulcator* Fabricius, 1775. Subsequent designation by Luederwaldt 1931a: 364).

*Ontherus*: Lacordaire 1856: 98 (redescription); Gemminger and Harold 1869: 1008 (complete list of species); Burmeister 1873b [= 1874]: 126 (redescription); Bruch 1911: 186 (list of species from Argentina); Gillet 1911a: 57 (complete list of species); Lucas 1920: 459 (catalog, distribution); Luederwaldt 1929: 10 (characters in key); Luederwaldt 1931a: 364 (redescription), 368 (characters in key); Paulian 1938: 233 (characters in key); Pessôa and Lane 1941: 437 (characters in key), 454 (redescription); Blackwelder 1944: 206 (list of species from Latin America); Pereira 1954a: 57 (characters in key); Roze 1955: 44 (list of species from Venezuela); Martínez 1959: 69 (list of species for Argentina); Halffter and Matthews 1966: 257 (catalog, distribution); Vulcano and Pereira 1967: 577 (characters in key); Howden and Young 1981: 12 (characters in key), 121 (diagnosis); Halffter and Edmonds 1982: 137 (catalog, distribution); Génier 1996: 22 (revision); Medina and Lopera 2000: 306 (characters in key); Vaz-de-Mello 2000: 194 (list of species from Brazil); Medina et al. 2001: 138 (list of species from Colombia); Arnett et al. 2002: 49 (characters in key); Ratcliffe 2002: 16 (list of species from Panama); Morón 2003: 55 (list of species from Mexico); Hamel-Leigue et al. 2006: 16 (list of species from Bolivia); Vaz-de-Mello et al. 2011: 27 (characters in key); Carvajal et al. 2011: 130 (diagnosis), 318 (cited for Ecuador); Krajcik 2012: 174 (complete list of species); Solís and Kohlmann 2012: 6 (list of species from Costa Rica); Chamorro et al. 2018: 77 (characters in key), 96 (list of species from Ecuador).

##### Subgenus Ontherus (Caelontherus) Génier, 1996

Ontherus (Caelontherus) Génier, 1996: 23 (original description. Type species: *Ontherusalexis* (Blanchard, 1845)); Vaz-de-Mello 2000: 194 (list of species from Brazil); Vaz-de-Mello et al. 2011: 27 (characters in key); Krajcik 2012: 174 (cited as subgenus); Chamorro et al. 2018: 77 (characters in key), 96 (list of species from Ecuador).

###### Ontherus (Caelontherus) aequatorius

Taxon classificationAnimaliaColeopteraScarabaeidae

Bates, 1891

Plate 35B

Ontherus aequatorius Bates, 1891: 25 (original description. Type locality: Ecuador, probably Pacific slopes).
Ontherus aequatorius : Gillet 1911a: 57 (complete list of species); Campos 1921: 56 (cited for Ecuador); Luederwaldt 1931a: 400 (characters in key); Blackwelder 1944: 206 (list of species from Latin America); Krajcik 2012: 174 (complete list of species).
Ontherus (Caelontherus) aequatorius : Génier 1996: 10 (characters in key), 34 (redescription); Carvajal et al. 2011: 318–319 (cited for Ecuador); Chamorro et al. 2018: 96 (cited for Ecuador).

####### Type specimens.

*Ontherusaequatorius* Bates, 1891. The holotype (♂) is deposited at the MNHN (see Génier 1996: 35). Locality: Ecuador, not examined.

####### Distribution.

Ecuador and Peru.

####### Records examined.

UNDETERMINED PROVINCE: without specific locality (2 specimens CEMT).

####### Literature records.

AZUAY: 8 km NE Giron, 2600 m (Génier 1996: 35); Tarqui (Génier 1996: 35). CAÑAR: 5 km E Zhud, 3000 m (Génier 1996: 35). CHIMBORAZO: Riobamba (Génier 1996: 35). NAPO: 24 km NW de Baeza, 2400 m (Génier 1996: 35). PICHINCHA: Gualea (Génier 1996: 35). SUCUMBÍOS: Sebundoy (Génier 1996: 35). UNDETERMINED PROVINCE: Pucay [= Bucay, Guyas] (Génier 1996: 35); without specific locality, probably Pacific slopes (Bates 1891: 25).

####### Temporal data.

Collected in March, April, June, July, and November.

####### Remarks.

Inhabits the montane cloud forests and the evergreen high montane forests of the Andean region from 2400–3000 m a.s.l.

Génier (1996), in his revision of the genus *Ontherus*, refers to the following two localities as uncertain specific locations: Sebundoy, located near Santa Bárbara in the Sucumbíos province, above 2300 m (mistaken by Génier Sibundoy at the Putumayo department in Colombia) and Pucay [= Bucay: Guayas] above 300 m, located along the Pacific coast of Ecuador. However, the distributional data for Pucay may be erroneous as the current distribution of *O.aequatorius* is limited to the Andean region.

###### Ontherus (Caelontherus) brevicollis

Taxon classificationAnimaliaColeopteraScarabaeidae

Kirsch, 1871

Ontherus brevicollis Kirsch, 1871: 340 (original description. Type locality: Bogotà).
Ontherus brevicollis : Harold 1880a: 23 (cited for Nueva Granada [= Colombia]); Gillet 1911a: 57 (complete list of species); Luederwaldt 1931a: 393 (characters in key); Blackwelder 1944: 206 (list of species for Latin America); Contreras 1951: 222 (cited for Colombia); Vulcano and Pereira 1967: 583 (characters in key); Medina et al. 2001: 138 (cited for Colombia); Krajcik 2012: 174 (complete list of species).
Ontherus (Caelontherus) brevicollis : Génier 1996: 31 (cited as new combination, redescription); Carvajal et al. 2011: 318–319 (cited for Ecuador); Chamorro et al. 2018: 96 (cited for Ecuador).

####### Type specimens.

*Ontherusbrevicollis* Kirsch, 1871. The holotype (♀) is deposited at the SMTD (see Génier 1996: 32). Locality: Bogota, not examined.

####### Distribution.

Bolivia, Brazil, Colombia, Ecuador, and Venezuela.

####### Literature records.

UNDETERMINED PROVINCE: without specific locality (Génier 1996: 32).

####### Temporal data.

It is not known when this species was collected.

####### Remarks.

There are currently no records for this species in Ecuador. However, Génier (1996) cited this species from Pasto at the Nariño department in Colombia (located just across the border of Ecuador) so it is possible that part of its distribution includes northern Ecuador too.

###### Ontherus (Caelontherus) compressicornis

Taxon classificationAnimaliaColeopteraScarabaeidae

Luederwaldt, 1931

Plate 35C

Ontherus compressicornis Luederwaldt, 1931a: 401 (original description. Type locality: Ecuador).
Ontherus compressicornis : Blackwelder 1944: 206 (list of species of Latin America); Medina et al. 2001: 138 (cited for Colombia); Gillett and Preziosi 2010: 89 (distribution); Krajcik 2012: 174 (complete list of species).
Ontherus (Caelontherus) compressicornis : Génier 1996: 51 (redescription); Carvajal et al. 2011: 318–319 (cited for Ecuador); Chamorro et al. 2018: 88 (figure 11B), 90 (figures 13B and 13D), 96 (cited for Ecuador).

####### Type specimens.

*Ontheruscompressicornis* Luederwaldt, 1931. The holotype (♂) is deposited at the MZSP (see Génier 1996: 52). Locality: Ecuador, not examained.

####### Distribution.

Ecuador and Colombia.

####### Records examined.

CAÑAR: Javín, 900–1400 m (3 specimens CEMT). CARCHI: 5 km NW de Maldonado, 550 m (2 specimens MQCAZ). Bosque Integral Otonga, 1815 m (40 specimens CEMT; 9 specimens MUTPL; 27 specimens MQCAZ). IMBABURA: Sta Rosa Intag, Cotacahi, 2000 m (1 specimen CEMT). LOJA: Alamor, Guambona, 1140 m (2 specimens CEMT). PICHINCHA: Curipoglio Cerro San Cristobal, 1800 m (1 specimen MUTPL).

####### Literature records.

COTOPAXI: 112 km W de Latcunga, 14 km de Pilalo, 1550 m (Génier 1996: 52). PICHINCHA: Bellavista Cloudforest Reserve, 2300 m (Gillett and Preziosi 2010: 89); 3 km E de Tandapi, Cornejo Astorga (Génier 1996: 52); 11 km E de Tandapi, 1310 m (Génier 1996: 52). PICHINCHA [= SANTO DOMINGO DE LOS TSÁCHILAS]: 23 km E de Alluriquín, Chiriboga Road, 1400 m (Génier 1996: 52); 28 km E de Alluriquín, Chiriboga Road, 1580 m (Génier 1996: 52); 31 km NE de Alluriquín, Chiriboga Road, 1770 m (Génier 1996: 52).

####### Temporal data.

Collected in February, March, April, May, June, July, August, September, and December.

####### Remarks.

Inhabits coastal evergreen foothill forests from 550–1300 m a.s.l. In the Andean region, it was recorded in both evergreen lower montane forests and montane cloud forests from 1550–2300 m a.s.l. According to Génier (1996) and Gillett and Preziosi (2010), this species has been collected using pitfall traps baited with carrion.

###### Ontherus (Caelontherus) diabolicus

Taxon classificationAnimaliaColeopteraScarabaeidae

Génier, 1996

Plate 35D

Ontherus (Caelontherus) diabolicus Génier, 1996: 48 (original description. Type locality: ECUADOR Pastaza: Llandia 1000 m. [17 km N del Puyo]).
Ontherus diabolicus : Medina et al. 2001: 139 (cited for Colombia); Hamel-Leigue et al. 2006: 16 (cited for Bolivia); Donoso et al. 2009: Appendix II. 17 (catalog of the types of the MQCAZ); Krajcik 2012: 174 (complete list of species).
Ontherus (Caelontherus) diabolicus : Morón 2006: 120 (catalog of types MXAL); Carvajal et al. 2011: 318–319 (cited for Ecuador); Chamorro et al. 2018: 96 (cited for Ecuador).

####### Type specimens.

Ontherus (Caelontherus) diabolicus Génier, 1996. The holotype (♂) is deposited at the CMNC (see Génier 1996: 49). Locality: ECUADOR Pastaza: Llandia 1000 m. [17 km N del Puyo], not examained.

####### Distribution.

Colombia and Ecuador.

####### Records examined.

MORONA SANTIAGO: Bosque Domoso, 1650 m (6 specimens CEMT); Comunidad Ángel Rouby, 1300 m, Cordillera del Kutukú (4 specimens MECN); Comunidad Unsuants, 600–1100 m, Cordillera del Kutukú (3 specimens MECN). NAPO: Bosque Protector la Cascada Río Coca, 640 m (1 specimen MUTPL); Cotundo Río Osayacu, 1070 m, sector Shamato (1 specimen MUTPL); Quebrada Granadillas, 1300 m, Bosque Protector la Cascada, Parque Nacional Sumaco (1 specimen MUTPL). ORELLANA: Comunidad Kiwcha Chiruisla Station, 180–250 m (7 specimens MQCAZ); Dayuma Campo Palanda plataforma Primavera 1, 235 m (1 specimen MUTPL); Eden, Campo Eden plataforma G, 220 m (1 specimen MUTPL); Estación Científica Yasuní PUCE, 250 m (21 specimens MQCAZ); Estación de Diversidad Tiputini, 285 m, Parque Nacional Yasuní (3 specimens MUTPL); Ines Arango road Tiwino-río Shiripuno, 250 m (1 specimen MUTPL); SCYasuní (2 specimens CEMT; 4 specimens MQCAZ). PASTAZA: Bosque Protector Oglán Alto, 555 m (1 specimen MUTPL); Chuyayacu oleoducto km 25, 200 m (2 specimens MUTPL). SUCUMBÍOS: Aucayacu Río El Eno, 16 km de Lago Agrio, 290 m (1 specimen MGO-UC); Bermejo plataforma ER-A road to Lumbaqui (1 specimen MUTPL); Gonzalo Pizarro, Simon Bolivar, 1200 m (2 specimens MECN); Tarapoa, Nuevo Manabí, 270 m (1 specimen MUTPL). ZAMORA CHINCHIPE: Zurmi, Comunidad La Wants, 1010 m (3 specimens MUTPL; 1 specimen MEPN); Zurmi Las Orquideas Río Nangaritza, 870 m (1 specimen MUTPL).

####### Literature records.

LOJA: Loja (Génier 1996: 50). MORONA SANTIAGO: Macas (Génier 1996: 50). NAPO: Aguamo [= Ahuano] (Génier 1996: 50); without specific locality (Génier 1996: 50); Río Jatun Yacu-Río Napo Wathersed (Génier 1996: 50); km 7.3 Sarayacu-Loreto Rd, 1200 m (Génier 1996: 50); km 11.1 Sarayacu-Loreto Rd, 1200 m (Génier 1996: 50); km 25.4 Sarayacu-Loreto Rd, 950 m (Génier 1996: 50); 12 km WSW Tena, 600 m. NAPO [= ORELLANA]: Coca (Génier 1996: 50). NAPO [= SUCUMBÍOS]: Limoncocha (Génier 1996: 50); 2 km N Limoncocha (Génier 1996: 50); Santa Cecilia, 340 m (Génier 1996: 50). PASTAZA: Llandia 17 km N Puyo, 1000 m (Génier 1996: 50; Morón, 2006: 120; Donoso et al. 2009: Appendix II. 17); 1 km E Mera, 1100 m (Génier 1996: 50); 22 km SE Puyo, 900 m (Génier 1996: 50); 25 km NE Puyo, 1000 m (Génier 1996: 50); 8 km E Río Negro 10 km W Pastaza, Shell, 1400 m (Génier 1996: 50); 9 km SE Veracruz, 900 m (Génier 1996: 50). SUCUMBÍOS: Dureno, 150 m (Génier 1996: 50). TUNGURAHUA: Ambato (Génier 1996: 50). ZAMORA CHINCHIPE: Sabanilla (Génier 1996: 50); without specific locality, Loja Oscordill. (Génier 1996: 50). UNDETERMINED PROVINCE: Chaca (Génier 1996: 50); Jarugui (Génier 1996: 50).

####### Temporal data.

Collected every month of the year.

####### Remarks.

Inhabits the lowland evergreen forests and evergreen foothill forests of the Amazon region from 150–1300 m a.s.l. Collected with pitfall traps baited with carrion and human feces.

###### Ontherus (Caelontherus) hadros

Taxon classificationAnimaliaColeopteraScarabaeidae

Génier, 1996

Plate 36A

Ontherus (Caelontherus) hadros Génier, 1996: 24 (original description. Type locality: Ecuador, Macas).
Ontherus hadros : Krajcik 2012: 174 (complete list of species).
Ontherus (Caelontherus) hadros : Carvajal et al. 2011: 318–319 (cited for Ecuador); Chamorro et al. 2018: 96 (cited for Ecuador).

####### Type specimens.

Ontherus (Caelontherus) hadros Génier, 1996. The holotype (♂) is deposited at the MNHN (see Génier 1996: 25). Locality: Ecuador, Macas, not examained.

####### Distribution.

Only known from Ecuador.

####### Records examined.

NAPO: Cuyuja, 2835 m (1 specimen CEMT; 2 specimens MUTPL).

####### Literature records.

MORONA SANTIAGO: Macas (Génier 1996: 25). NAPO: 27 km NW de Baeza, 2700 m (Génier 1996: 25). PASTAZA: Zarayacu [= Sarayacu] (Génier 1996: 25).

####### Temporal data.

Collected in March and December.

####### Remarks.

Inhabits the montane cloud forests and the evergreen high montane forests of the Andean region from 2700–2835 m a.s.l. Collected in Andean tapir feces.

###### Ontherus (Caelontherus) howdeni

Taxon classificationAnimaliaColeopteraScarabaeidae

Génier, 1996

Plate 36B

Ontherus (Caelontherus) howdeni Génier, 1996: 47 (original description. Type locality: Chanchamayo, Peru).
Ontherus howdeni : Krajcik 2012: 174 (complete list of species); Ratcliffe et al. 2015: 197 (cited for Peru).
Ontherus (Caelontherus) howdeni : Carvajal et al. 2011: 318–319 (cited for Ecuador); Chamorro et al. 2018: 96 (cited for Ecuador).

####### Type specimens.

Ontherus (Caelontherus) howdeni Génier, 1996. The holotype (♂) is deposited at the CMNC (see Génier 1996: 48). Locality: Chanchamayo, Peru, not examained.

####### Distribution.

Ecuador and Peru.

####### Records examined.

SUCUMBÍOS: La Bonita, 1800 m (3 specimens CEMT)

####### Literature records.

Without specific locality (Génier, 1996: 48)

####### Temporal data.

Collected in May.

####### Remarks.

Inhabits the montane cloud forests of the Andean region at 1800 m a.s.l. The collection method is unknown.

###### Ontherus (Caelontherus) incisus

Taxon classificationAnimaliaColeopteraScarabaeidae

(Kirsch, 1871)

Plate 36C

Pinotus incisus Kirsch, 1871: 341 (original description. Type locality: Bogotà).
Ontherus incisus : Harold 1880a: 23 (new combination for Pinotusincisus Kirsch, 1971); Gillet 1911a: 58 (complete list of species); Luederwaldt 1931a: 398 (characters in key); Blackwelder 1944: 206 (list of species from Latin America); Vulcano and Pereira 1967: 583 (characters in key); Medina et al. 2001: 139 (cited for Colombia); Krajcik 2012: 174 (complete list of species).
Ontherus (Caelontherus) incisus : Génier 1996: 45 (redescription); Carvajal et al. 2011: 318–319 (cited for Ecuador); Chamorro et al. 2018: 96 (cited for Ecuador).
Ontherus thoracicus Waterhouse, 1891a: 356 (original description); Gillet 1911a: 58 (complete list of species); Blackwelder 1944: 206 (list of species from Latin America); Vulcano and Pereira 1967: 583 (characters in key); Génier 1996: 45 (synonym of Ontherus (Caelontherus) incisus Kirsch, 1871).

####### Type specimens.

*Pinotusincisus* Kirsch, 1871. The holotype (♂) is deposited at the MNHN (see Génier 1996: 46). Locality: Bogota, not examained.

*Ontherusthoracicus* Waterhouse, 1891. The holotype (♂) is deposited at the NHML (see Génier 1996: 46). Locality: Colombia, not examained.

####### Distribution.

Colombia, Ecuador, and Peru.

####### Records examined.

NAPO: Cabañas San Isidro, 2 km NW de Cosanga, 2150 m (6 specimens MQCAZ); Oyacachi, 2550 m (1 specimen MQCAZ); Sierra de Los Guacamayos, 1900 m (1 specimen CEMT). ZAMORA CHINCHIPE: Chito río Sangolas, 1540 m (2 specimens MUTPL); Chito río San Francisco, 1800 m (2 specimens MUTPL).

####### Literature records.

MORONA SANTIAGO: Macas (Génier 1996: 46). NAPO: 17 km NE Baeza, 1280 m (Génier 1996: 46); 15 km NW Baeza, 2010 m (Génier 1996: 46); 7 km S Baeza, 2000 m (Génier 1996: 46). PASTAZA: Canelos (Génier 1996: 46). TUNGURAHUA: Santa Inéz [= Santa Inés] (Génier 1996: 46). UNDETERMINED PROVINCE: Normandia (Génier 1996: 46).

####### Temporal data.

Collected in February, April, July, May, and December.

####### Remarks.

Inhabits the evergreen foothill forests in the Amazonian range at 1280 m a.s.l. In the Andean region, it was recorded in evergreen lower montane forests and in montane cloud forests from 1540–2550 m a.s.l. Collected with pitfall traps baited with human feces.

###### Ontherus (Caelontherus) laminifer

Taxon classificationAnimaliaColeopteraScarabaeidae

Balthasar, 1938

Plate 36D

Ontherus laminifer Balthasar, 1938: 221 (original description. Type locality: Brazil, Manaos).
Ontherus laminifer : Blackwelder 1944: 206 (list of species for Latin America); Vulcano and Pereira 1967: 583 (characters in key); Krajcik 2012: 174 (complete list of species); Ratcliffe et al. 2015: 197 (cited for Peru).
Ontherus (Caelontherus) laminifer : Génier 1996: 27 (cited as new combination, redescription); Vaz-de-Mello 2000: 194 (cited for Brazil); Chamorro et al. 2018: 96 (cited for Ecuador).

####### Type specimens.

*Ontheruslaminifer* Balthasar, 1938. The holotype (♂) is deposited at the NMPC (see Génier 1996: 28). Locality: Amazonas, Manaos, not examained.

####### Distribution.

Brazil, Ecuador, and Peru.

####### Records examined.

ORELLANA: Estación Río Huiririma, 220 m (1 specimen MQCAZ).

####### Temporal data.

Collected in September.

####### Remarks.

Inhabits the lowland evergreen forests and evergreen foothill forests of the Amazon at 220 m a.s.l. The collection method is unknown.

###### Ontherus (Caelontherus) magnus

Taxon classificationAnimaliaColeopteraScarabaeidae

Génier, 1996

Plate 37A

Ontherus (Caelontherus) magnus Génier, 1996: 25 (original description. Type locality: Ecuador).
Ontherus magnus : Krajcik 2012: 174 (complete list of species).
Ontherus (Caelontherus) magnus : Carvajal et al. 2011: 318–319 (cited for Ecuador); Chamorro et al. 2018: 96 (cited for Ecuador).

####### Type specimens.

Ontherus (Caelontherus) magnus Génier, 1996. The holotype (♂) is deposited at the NMHU (see Génier 1996: 25). Locality: Ecuador, not examained.

####### Distribution.

Only known from Ecuador.

####### Records examined.

Without specific locality (1 specimen CEMT)

####### Literature records.

PICHINCHA: 21 km E Tandapi, Cornejos Astorga, 2600 m (Génier 1996: 26)

####### Temporal data.

Collected in June.

####### Remarks.

Inhabits the montane cloud forests of the Andean region at 2600 m a.s.l. The collection method is unknown.

###### Ontherus (Caelontherus) pilatus

Taxon classificationAnimaliaColeopteraScarabaeidae

Génier, 1996

Plate 37B

Ontherus (Caelontherus) pilatus Génier, 1996: 52 (original description. Type locality: Ecuador, Pichincha, 23 km E de Alluriquín Chiriboga Rd. 4600 feet [1400 m]).
Ontherus pilatus : Medina et al. 2001: 139 (cited for Colombia); Krajcik 2012: 174 (complete list of species).
Ontherus (Caelontherus) pilatus : Carvajal et al. 2011: 318–319 (cited for Ecuador); Chamorro et al. 2018: 96 (cited for Ecuador).

####### Type specimens.

Ontherus (Caelontherus) pilatus Génier, 1996. The holotype (♂) is deposited at the CMNC (see Génier 1996: 53). Locality: Ecuador, Pichincha, 23 km E de Alluriquín Chiriboga Rd. 4600’ [1400 m], not examained.

####### Distribution.

Colombia and Ecuador.

####### Records examined.

EL ORO: Bella María, Los Ingleses, 420 m (10 specimens MUTPL).

####### Literature records.

PICHINCHA [= SANTO DOMINGO DE LOS TSÁCHILAS]: 14 km NE de Alluriquín road to Chiriboga (Génier 1996: 53); 23 km E de Alluriquín, 1400 m (Génier 1996: 534).

####### Temporal data.

Collected in June and September.

####### Remarks.

Inhabits coastal evergreen foothill forests from 420–1400 m a.s.l. Collected with pitfall traps baited with human feces.

###### Ontherus (Caelontherus) politus

Taxon classificationAnimaliaColeopteraScarabaeidae

Génier, 1996

Plate 37C

Ontherus (Caelontherus) politus Génier, 1996: 33 (original description. Type locality: Ecuador, Napo, 6600 feet [= 2010 m] 15 km NW de Baeza).
Ontherus politus : Donoso et al. 2009: Appendix II. 17 (catalog of types MQCAZ); Krajcik 2012: 174 (complete list of species); González and Medina 2015: 88 (distribution).
Ontherus (Caelontherus) politus : Morón 2006: 120 (catalog of types MXAL); Carvajal et al. 2011: 318–319 (cited for Ecuador); Chamorro et al. 2018: 96 (cited for Ecuador).

####### Type specimens.

Ontherus (Caelontherus) politus Génier, 1996. The holotype (♂) is deposited at the CMNC (see Génier 1996: 33). Locality: Ecuador, Napo, 6600’ [= 2010 m] 15 km NW de Baeza, not examained.

####### Distribution.

Colombia and Ecuador.

####### Records examined.

CARCHI: km 3 road to Tufiño-Maldonado, 3400 m (2 specimens MQCAZ). NAPO: Cosanga Yanayacu Biost. (2 specimens MECN). SUCUMBÍOS: Santa Barbara, 2500 m (1 specimen MECN). TUNGURAHUA: Machay (1 specimen CEMT). ZAMORA CHINCHIPE: El Tambo (33 specimens CEMT; 41 specimens MUTPL); Reserva El Colibri, 2080 m (55 specimens MUTPL); Estación Biológica San Francisco, 1900 m (27 specimens MUTPL); La Pituca, 1830 m Cuenca del río Curitza (1 specimen MUTPL); Romerillos sendero Nagaritza, 2200 m, Parque Nacional Podocarpus (4 specimens MECN). UNDETERMINED PROVINCE: without specific locality (1 specimen CEMT).

####### Literature records.

CARCHI: Env[iron]. de Tulcán [= Environs of Tulcán] (Génier 1996: 34). CARCHI [= SUCUMBÍOS]: Sebondoi [= Sebundoy], 2600 m (Génier 1996: 34). NAPO: 6600 feet [= 2100 m], 15 NW de Baeza, 2010 m (Génier 1996: 33; Donoso et al. 2009: Appendix II. 17); 7 km S de Baeza, 2000 m (Génier 1996: 34); 24 km NW de Baeza, 2400 m (Génier 1996: 34; Morón 2006: 120). UNDETERMINED PROVINCE: without specific locality (Génier 1996: 34).

####### Temporal data.

Collected in February, March, July, September, October, November, and December.

####### Remarks.

Inhabits the montane cloud forests and evergreen high montane forests of the Andean region from 2000–3400 m a.s.l. Collected with pitfall traps baited with pig feces. Génier (1996) reports Sebundoy, a locality in Sucumbíos, near Santa Bárbara, with an altitude above 2600 m a.s.l. This locality was probably confused by the author with Sibundoy, located in the Putumayo department in Colombia.

###### Ontherus (Caelontherus) tenustriatus

Taxon classificationAnimaliaColeopteraScarabaeidae

Génier, 1996

Plate 37D

Ontherus (Caelontherus) tenustriatus Génier, 1996: 41 (original description. Type locality: Peru).
Ontherus tenustriatus : Krajcik 2012: 174 (complete list of species); Ratcliffe et al. 2015: 197 (cited for Peru).
Ontherus (Caelontherus) tenustriatus : Chamorro et al. 2018: 96 (cited for Ecuador).

####### Type specimens.

Ontherus (Caelontherus) tenustriatus Génier, 1996. The holotype (♂) is deposited at the NHML (see Génier 1996: 43). Locality: Peru, not examined.

####### Distribution.

Ecuador and Peru.

####### Records examined.

ORELLANA: Daimi 1 (1 specimen CEMT).

####### Temporal data.

Collected in September.

####### Remarks.

Inhabits the lowland evergreen forests of the Amazon region. Collected with pitfall traps baited with human feces.

###### Ontherus (Caelontherus) trituberculatus

Taxon classificationAnimaliaColeopteraScarabaeidae

Balthasar, 1938

Plate 38A

Ontherus trituberculatus Balthasar, 1938: 220 (original description. Type locality: Amerika merid. [= South America.], Cachabé).
Ontherus trituberculatus : Blackwelder 1944: 206 (list of species from Latin America); Medina et al. 2001: 139 (cited for Colombia); Krajcik 2012: 174 (complete list of species).
Ontherus (Caelontherus) trituberculatus : Génier 1996: 53 (cited as new combination, redescription); Carvajal et al. 2011: 318–319 (cited for Ecuador); Chamorro et al. 2018: 96 (cited for Ecuador).

####### Type specimens.

*Ontherustrituberculatus* Balthasar, 1938 Génier, 1996. The holotype (♂) is deposited at the SMTD (see Génier 1996: 54). Locality: Cachabé, not examained.

####### Distribution.

Colombia and Ecuador.

####### Records examined.

CARCHI: Maldonado, 1830 m (10 specimens CEMT; 8 specimens MQCAZ); Quinjul, 1700 m (2 specimens CEMT; 4 specimens MQCAZ); Tobar Donoso, 300 m (16 specimens MECN). EL ORO: Reserva Jocotoco, 1250 m (3 specimens MQCAZ). ESMERALDAS: Charco Vicente (21 specimens MECN; 17 specimens MQCAZ); Palma Real (14 specimens MECN; 11 specimens MQCAZ); Playa de Oro, 200 m (1 specimen MUTPL; 18 specimens MECN; 26 specimens MQCAZ); Playa de Oro, La Tabla (37 specimens MECN; 21 specimens MQCAZ); Playa de Oro, Padre Santo (21 specimens MECN; 28 specimens MQCAZ); Playa de Oro, Playa Rica (13 specimens MECN; 9 specimens MQCAZ); Playa de Oro, Pote (8 specimens CEMT; 20 specimens MQCAZ); Salto del Bravo (17 specimens MECN; 10 specimens MQCAZ); Tsejpi (28 specimens MECN; 17 specimens MQCAZ). IMBABURA: Río Getsemani, 600 m (4 specimens MQCAZ); Lita, 680 m (7 specimens MECN; 5 specimens MQCAZ). PICHINCHA: Choconde, 1200 m, San Miguel de los Bancos (1 specimen MUTPL); El Encuentro, 620 m, San Miguel de los Bancos (1 specimen MUTPL); El Tigre Río Guayllabamba, Pedro Vicente Maldonado (2 specimens MUTPL); Guayabilla Río Guayllabamba, 520 m, Manduriacus (1 specimen MUTPL); Llurimaguas Río Guayllabamba, 290 m, Pedro Vicente Maldonado (2 specimens MUTPL); Mangaloma, 820 m, San Miguel de los Bancos (1 specimen MUTPL); Pedro Vicente Maldonado, 640 m (3 specimens CEMT; 5 specimens MCAZ); Tortugo Río Guayllabamba, 450 m, Pedro Vicente Maldonado (2 specimens MUTPL).

####### Literature records.

CARCHI: Chical, 1250 m (Génier 1996: 55); 18 km SE Maldonado, 2420 m (Génier 1996: 55). ESMERALDAS: Cachabé (Génier 1996: 55). PICHINCHA: 5.3 km road to Pachija [= Pachijal], 2800–3000m (Génier 1996: 55); 85 km NW de Quito, on Puerto Quito Rd, 1520 m (Génier 1996: 55); 113 km NW Quito, on Puerto Quito Rd, 790 m (Génier 1996: 55). PICHINCHA: [= SANTO DOMINGO DE LOS TSÁCHILAS]: 4 km SE Santo Domingo, 500 m (Génier 1996: 55); 16 km SE Santo Domingo, Tinalandia, 680 m (Génier 1996: 55).

####### Temporal data.

Collected in February, March, April, May, July, September, October, November, and December.

####### Remarks.

Inhabits the coastal lowland evergreen forests and coastal evergreen foothill forests from 200–1250 m a.s.l. Additionally, there is a single record for this species for a locality 5.3 km along the road to Pachija [= Pachijal]. However, because this is the only record from the Andean region cited by Génier (1996), it may be erroneous. Collected with pitfall traps baited with carrion and human feces.

##### Subgenus Ontherus (Ontherus) Erichson, 1847

Ontherus (Ontherus) s. str. Erichson, 1847: 107 (original description. Type species: *Scarabaeussulcator*Fabricius 1775 original combination); Génier 1996: 70 (redescription); Vaz-de-Mello 2000: 194 (list of species for Brazil); Vaz-de-Mello et al. 2011: 28 (characters in key); Boilly and Vaz-de-Mello 2013: 109 (characters in key); Chamorro et al. 2018: 77 (characters in key), 96 (list of species from Ecuador).

###### Ontherus (Ontherus) azteca

Taxon classificationAnimaliaColeopteraScarabaeidae

Harold, 1869

Plate 38B

Ontherus azteca Harold, 1869b: 503 (original description. Type locality: Cordova).
Ontherus azteca : Bates 1887: 50 (redescription, distribution); Gillet 1911a: 57 (complete list of species); Blackwelder 1944: 206 (list of species of Latin America); Medina et al. 2001: 138 (cited for Colombia); Ratcliffe 2002: 16 (cited for Panama); Hamel-Leigue et al. 2006: 16 (cited for Bolivia); Solís and Kohlmann 2012: 6 (cited for Costa Rica); Krajcik 2012: 174 (complete list of species); Ratcliffe et al. 2015: 197 (cited for Peru).
Ontherus (Ontherus) azteca : Génier 1996: 87 (cited as new combination, redescripcion); Vaz-de-Mello 2000: 194 (list of species for Brazil); Morón 2003: 56 (cited for Mexico); Carvajal et al. 2011: 318–319 (cited for Ecuador); Chamorro et al. 2018: 96 (cited for Ecuador).
Ontherus villosus Luederwaldt, 1930: 107 (original description); Luederwaldt 1931a: 372 (characters in key), 391 (redescription); Pessôa and Lane 1941: 457 (characters in key); Blackwelder 1944: 206 (list of species of Latin America); Génier 1996: 87 (synonym of Ontherusazteca Harold, 1869).
Ontherus strius Howden & Young, 1981: 122 (original description); Génier 1996: 87 (synonym of Ontherusazteca Harold, 1869).

####### Type specimens.

*Ontherusazteca* Harold, 1869. The holotype (♀) is deposited at the NHML (see Génier 1996: 88). Locality: Mexico, Oaxaca, not examined.

*Ontherusvillosus* Luederwaldt, 1930. The lectotype (♂) is deposited at the MZSP (see Génier 1996: 88). Locality: São Paulo, Ypiranga, not examined.

*Ontherusstrius* Howden & Young, 1981. The holotype (♂) is deposited at the USNM (see Génier 1996: 89). Locality: Panama, Canal Zone, not examined.

####### Distribution.

Bolivia, Brazil, Colombia, Costa Rica, Ecuador, Mexico, Panama, Paraguay, and Peru.

####### Records examined.

ORELLANA: San Sebastian de Coca Comuna Huataraco (1 specimen MUTPL).

####### Literature records.

NAPO: km 7.3 Sarayacu-Loreto Rd., 1200 m (Génier 1996: 89). NAPO [= SUCUMBÍOS]: 2 km de Limoncocha, 250 m (Génier 1996: 89); Limoncocha, 250 m (Génier 1996: 89). PASTAZA: 25 km NNE Puyo, 1000 m (Génier 1996: 89); 22 km SE del Puyo, 900 m (Génier, 1996: 89).

####### Temporal data.

Collected in March.

####### Remarks.

Inhabits the lowland evergreen forests and evergreen foothill forests of the Amazon region from 250–1200 m a.s.l. Although the collection method for specimens from Ecuador is unknown, Génier (1996) reported that this species was collected in other countries using pitfall traps baited with feces and carrion.

###### Ontherus (Ontherus) edentulus

Taxon classificationAnimaliaColeopteraScarabaeidae

Génier, 1996

Plate 38C

Ontherus (Ontherus) edentulus Génier, 1996: 102 (original description. Type locality: Pérou [= Peru], Chanchamayo).
Ontherus edentulus : Krajcik 2012: 174 (complete list of species); Ratcliffe et al. 2015: 197 (cited for Peru).
Ontherus (Ontherus) edentulus : Vaz-de-Mello 2000: 194 (cited for Brazil); Carvajal et al. 2011: 318–319 (cited for Ecuador); Chamorro et al. 2018: 96 (cited for Ecuador).

####### Type specimens.

Ontherus (Ontherus) edentulus Génier, 1996. The holotype (♂) is deposited at the MNHN (see Génier 1996: 104). Locality: Pérou Chanchamayo, not examined.

####### Distribution.

Ecuador and Peru.

####### Records examined.

NAPO: Talag, 650 m (1 specimen MQCAZ); Tena (1 specimen CEMT). ORELLANA: Estación de Biodiversidad Tiputini, 250 m (1 specimen CEMT).

####### Literature records.

LOJA: Loja (Génier 1996: 104). MORONA SANTIAGO: Macas (Génier 1996: 104). ZAMORA CHINCHIPE: Zamora (Génier 1996: 104).

####### Temporal data.

Collected in June and September.

####### Remarks.

Inhabits the lowland evergreen forests and evergreen foothill forests of the Amazon region from 250–1020 m a.s.l. Species was collected with pitfall traps baited with carrion.

###### Ontherus (Ontherus) pubens

Taxon classificationAnimaliaColeopteraScarabaeidae

Génier, 1996

Plate 38D

Ontherus (Ontherus) pubens Génier, 1996: 71 (original description. Type locality: Ecuador, Napo, 400 m, Jatun Sacha Biol. Station, 21 km E Puerto Napo).
Ontherus pubens : Hamel-Leigue et al. 2006: 16 (cited for Bolivia); Donoso et al. 2009: Appendix II. 17 (catalog of types MQCAZ); Krajcik 2012: 174 (complete list of species); Ratcliffe et al. 2015: 197 (cited for Peru).
Ontherus (Ontherus) pubens : Vaz-de-Mello 2000: 194 (cited for Brazil); Morón 2006: 120 (catalog of types MXAL); Carvajal et al. 2011: 318–319 (cited for Ecuador); Chamorro et al. 2018: 90 (figure 13E), 96 (cited for Ecuador).

####### Type specimens.

Ontherus (Ontherus) pubens Génier, 1996. The holotype (♂) is deposited at the CMNC (see Génier 1996: 72). Locality: Ecuador, Napo, 400 m, Jatun Sacha Biol. Station [21 km E Puerto Napo], not examined.

####### Distribution.

Argentina, Bolivia, Brazil, Colombia, Ecuador, Peru, and Venezuela.

####### Records examined.

MORONA SANTIAGO: km 8 Mendes Paute (3 specimens CEMT); Bosque Domoso, 1650 m (3 specimens CEMT); Comunidad Unsuants, 500–700 m, Cordillera del Kutukú (6 specimens MECN); Gualaquiza (4 specimens MECN); Río Abanico, L. Proaño y 9 de Octubre, 1640 m (3 specimens MECN). NAPO: 5 km NE CJ Arosemena, 800 m (1 specimen CEMT); Cotundo (3 specimens MECN); Puerto Misahuallí (3 specimens CEMT); Tena, Parque Amazónico, 520 m (1 specimen MUTPL); Santo Domingo de Hollin, Rio Hollin, 635 m (1 specimen MUTPL). ORELLANA: Dayuma Campo Palanda, 235 m, plataforma Primavera 1 (1 specimen MUTPL); Rodrigo Borja IAMOE (7 specimens CEMT); San Sebastian del Coca, Comuna Guataraco, 345 m, Campo Pata (1 specimen MUTPL); San Sebastian del Coca, Comuna Shamanal, 345 m, Campo Palo Azul (1 specimen MUTPL); Taracoa (3 specimens MECN); Lago San Pedro, plataforma Copal, 310 m (1 specimen MUTPL). PASTAZA: Bosque Protector Oglán Alto, 545 m (1 specimen MUTPL); Pandanuque, 420 m (1 specimen MUTPL). SUCUMBÍOS: 6 km de Dureno, Precooperativa Los Vergeles, 290 m (84 specimens MGO-UC); Aucayacu 275 m Río El Eno, 16 km de Lago Agrio (1 specimen MGO-UC); Nueva Loja plataforma Iguana, 310 m (1 specimen MUTPL); Pacayacu Campo Libertador, Tapi, 265 m (3 specimens MUTPL); Pacayacu Campo Libertador, Tetete, 290 m (1 specimen MUTPL); Río Coca-Río Supayacu, 380 m, Parque Nacional Sumaco (1 specimen MUTPL); Tarapoa, Nuevo Manabí, 270 m (1 specimen MUTPL); Shushufindi (4 specimens MECN). ZAMORA CHINCHIPE: km 1 road Cumbaritza-Gualaquiza, 1100 m (3 specimens CEMT); Zurmi Las Orquideas Río Nangaritza, 870 m (1 specimen MUTPL).

####### Literature records.

LOJA: Loja (Génier 1996: 73). MORONA SANTIAGO: Macas (Génier 1996: 73). NAPO: without specific locality (Génier 1996: 73); Archidona (Génier 1996: 73); Jatun Sacha Estación Biológica, 21 km E Puerto Napo (Génier 1996: 73; Morón 2006: 121); 10 km W Puerto Misahualli (Génier 1996: 74); Reventador (Génier 1996: 74); Río Napo, Pozzi (Génier 1996: 74); km 7.3 Sarayacu-Loreto Rd, 1200 m (Génier 1996: 74); Tena, 400 m (Génier 1996: 74; Donoso et al. 2009: Appendix II. 17); 5 km W Tena, 500 m (Génier 1996: 74); 12 km SW Tena 600 m (Génier 1996: 74). NAPO [= ORELLANA]: Coca (Génier 1996: 73). Napo R, 250 m (Génier 1996: 73). NAPO [= SUCUMBÍOS]: Dureno, on Río Aguarico, 150 m (Génier 1996: 73); Limoncocha, 700 feet [= 210 m] (Génier 1996: 73); 2 km Limoncocha (Génier 1996: 73). PASTAZA: Canelos (Génier 1996: 74); Curaray (Génier 1996: 74); Llandia 1000 m, 17 km N del Puyo (Génier 1996: 74). ZAMORA CHINCHIPE: Sabanilla (Génier 1996: 74); Zamora (Génier 1996: 74). UNDETERMINED PROVINCE: without specific locality (Génier 1996: 73).

####### Temporal data.

Collected in January, February, March, April, May, June, July, August, September, October, November, and December.

####### Remarks.

Inhabits the lowland evergreen forests and evergreen foothill forests of the Amazon region from 150–1200 m a.s.l. Species was collected manually and with pitfall traps baited with carrion and human feces.

#### Genus *Onthophagus* Latreille, 1802

*Onthophagus* Latreille, 1802: 141 (original description. Type species: *Scarabaeustaurus* Schreber, 1759 by primary monotypy).

*Onthophagus*: Audinet-Serville 1825: 353 (redescription); Agassiz 1846: 749 (catalog); Latreille 1829: 536 (redescription); Brullé 1837: 300 (redescription); Castelnau 1840: 83 (redescription); Lacordaire 1856: 107 (redescription); Gemminger and Harold 1869: 1024 (catalog); Gillet 1911a: 118 (catalog); Boucomont and Gillet 1927: 205 (catalog of species); Dawson 1922: 61 (characters in key); Paulian 1938: 232 (characters in key); Blackwelder 1944: 211 (list of species from Latin America); Roze 1955: 45 (list of species from Venezuela); Martínez 1959: 108 (list of species from Argentina); Howden and Cartwright 1963: 6 (redescription); Halffter and Matthews 1966: 254 (catalog, distribution); Vulcano and Pereira 1967: 562 (characters in key); Howden and Young 1981: 11 (characters in key), 93 (redescription); Halffter and Edmonds 1982: 135 (catalog, distribution); Medina and Lopera 2000: 301 (characters in key); Vaz-de-Mello 2000: 194 (list of species from Brazil); Medina et al. 2001: 139 (list of species from Colombia); Kohlmann and Solís 2001: 160 (redescription); Arnett et al. 2002: 49 (characters in key); Ratcliffe 2002: 17 (list of species from Panama); Hamel-Leigue et al. 2006: 16 (list of species from Bolivia); Pulido-Herrera and Zunino 2007: 94 (catalog of species); Vaz-de-Mello et al. 2011: 23 (characters in key); Carvajal et al. 2011: 145 (diagnosis), 322 (list of species from Ecuador); Krajcik 2012: 174 (complete list of species); Solís and Kohlmann 2012: 8 (list of species from Costa Rica); Chamorro et al. 2018: 74 (characters in key), 96–97 (list of species from Ecuador).

*Chalcoderus* Erichson, 1848: 763 (original description. Type species: unnamed); Lacordaire 1856: 109 (comment); Gemminger and Harold 1869: 1024 (cited as synonym of *Onthophagus* Latreille, 1802); Gillet 1911a: 118 (cited as synonym of *Onthophagus* Latreille, 1802); Blackwelder 1944: 211 (cited as synonym of *Onthophagus* Latreille, 1802); Solís and Kohlmann 2012: 8 (cited as synonym of *Onthophagus*Latreille 1802).

*Monapus* Erichson, 1848: 763 (original description. Type species: unnamed, probably *Onthophagusmniszechi* Harold, 1869); Lacordaire 1856: 109 (comment); Gemminger and Harold 1869: 1024 (cited as synonym of *Onthophagus* Latreille, 1802); Gillet 1911a: 118 (cited as synonym of *Onthophagus* Latreille, 1802); Blackwelder 1944: 211 (cited as synonym of *Onthophagus* Latreille, 1802); Solís and Kohlmann 2012: 8 (cited as synonym of *Onthophagus* Latreille, 1802).

*Psilax* Erichson, 1848: 764 (original description. Type species: *Onthophaguspronus* Erichson, 1842); Lacordaire 1856: 109 (comment); Gemminger and Harold 1869: 1024 (cited as synonym of *Onthophagus* Latreille, 1802); Gillet 1911a: 118 (cited as synonym of *Onthophagus* Latreille, 1802); Blackwelder 1944: 211 (cited as synonym of *Onthophagus*Latreille 1802); Solís and Kohlmann 2012: 8 (cited as synonym of *Onthophagus* Latreille, 1802).

*Proagoderus* Lansberge, 1883: 14 (original description. Type species: unnamed); Gillet 1911a: 118 (cited as subgenus of *Onthophagus* Latreille, 1802); Blackwelder 1944: 211 (cited as synonym of *Onthophagus* Latreille, 1802).

*Gonocyphus* Lansberge, 1885: 382 (original description. Type species: unnamed); Gillet 1911a: 118 (cited as synonym of *Onthophagus*Latreille 1802); Blackwelder 1944: 211 (cited as synonym of *Onthophagus* Latreille, 1802); Solís and Kohlmann 2012: 8 (cited as synonym of *Onthophagus* Latreille, 1802).

*Diastellopalpus* Lansberge, 1886: 91 (original description. Type species: unnamed); Gillet, 1911a: 118 (cited as synonym of *Onthophagus* Latreille, 1802); Blackwelder, 1944: 211 (cited as synonym of *Onthophagus* Latreille, 1802).

*Tauronthophagus* Shipp, 1895: 179 (original description. Type species: *Onthophagusrangifer* Klug, 1855); Gillet 1911a: 118 (cited as synonym of *Onthophagus* Latreille, 1802); Blackwelder 1944: 211 (cited as synonym of *Onthophagus*Latreille 1802, written as *Pauronthophagus* Shipp); Solís and Kohlmann 2012: 8 (cited as synonym of *Onthophagus* Latreille, 1802).

*Macropocopris* Arrow, 1920: 435 (original description. Type species: *Macropocoprisprehensilis* Arrow, 1920); Halffter and Matthews 1966: 254 (cited as genus); Halffter and Edmonds 1982: 135 (cited as synonym of *Onthophagus* Latreille, 1802); Solís and Kohlmann 2012: 8 (cited as synonym of *Onthophagus* Latreille, 1802).

##### Subgenus Onthophagus (Onthophagus) Latreille, 1802

Onthophagus (Onthophagus) s. str. Latreille, 1802: 141 (original description. Type species: *Scarabaeustaurus* Schreber, 1759 original combination); Halffter and Matthews 1966: 254 (cited as subgenus Onthophagus Latreille, 1802); Zunino 1979: 4 (redescription); Halffter and Edmonds 1982: 135 (cited as subgenus of *Onthophagus* Latreille, 1802); Vaz-de-Mello 2000: 194 (cited as subgenus Onthophagus Latreille, 1802); Morón 2003: 67 (redescription); Vaz-de-Mello et al. 2011: 23 (characters in key); Boilly and Vaz-de-Mello 2013: 106 (characters in key); Chamorro et al. 2018: 74 (characters in key), 96–97 (list of species from Ecuador).

###### Onthophagus (Onthophagus) acuminatus

Taxon classificationAnimaliaColeopteraScarabaeidae

Harold, 1880

Plate 39A

Onthophagus acuminatus Harold, 1880a: 30 (original description. Type locality: Fusagasugá, Ambalema und Muzo; auch von Colon).
Onthophagus acuminatus : Gillet 1911a: 204 (complete list of species); Boucomont and Gillet 1927: 204 (catalog of species); Boucomont 1932: 307 (characters in key), 320 (distribution); Paulian 1936b: 506 (redescription); Blackwelder 1944: 211 (list of species from Latin America); Contreras 1951: 223 (cited for Colombia); Vulcano and Pereira 1967: 564 (characters in key); Howden and Young 1981: 98 (characters in key), 104 (redescription); Zunino and Halffter 1997: 161 (list of species); Kohlmann and Solís 2001: 167 (characters in key, redescription); Medina et al. 2001: 139 (cited for Colombia); Ratcliffe 2002: 17 (cited for Panama); Morón 2003: 71 (cited for Mexico); Pulido-Herrera and Zunino 2007: 94 (catalog of species, distribution); Carvajal et al. 2011: 322–323 (cited for Ecuador); Krajcik 2012: 174 (complete list of species); Solís and Kohlmann 2012: 8 (cited for Costa Rica); Delgado and Curoe 2014: 66 (characters in key, cited for Panama); Rossini et al. 2018b: 9 (list of species of the curvicornis complex).
Onthophagus (Onthophagus) acuminatus : Chamorro et al. 2018: 96 (cited for Ecuador).

####### Type specimens.

*Onthophagusacuminatus* Harold, 1880. The type is deposited at the MNHN (see Kohlmann and Solís 2001: 167). Locality: Panamá, Colon, Champion, not examined.

####### Distribution.

Colombia, Costa Rica, Ecuador, Mexico, Nicaragua, and Panama.

####### Records examined.

BOLIVAR: Bosque Protector Filo Palanga, 970 m (8 specimens MUTPL). CARCHI: Tobar Donoso, 300 m (3 specimens MECN). COTOPAXI: Guasaganda km 4, 500 m (11 specimens MQCAZ); Las Pampas, 1200 m (8 specimens MQCAZ). EL ORO: Uzhcurrumi, 500 m (1 specimen CEMT; 17 specimens MQCAZ). ESMERALDAS: Calle Mansa (3 specimens CEMT; 45 specimens MQCAZ); Chispero (11 specimens CEMT; 57 specimens MQCAZ; 2 specimens MECN); Colón del Ónzole (36 specimens CEMT; 65 specimens MQCAZ; 3 specimens MECN); Gualpi, El Pajonal (11 specimens CEMT; 45 specimens MQCAZ; 1 specimens MECN); Gualpi (1 specimen CEMT; 18 specimens MQCAZ); Jeyambi PMFC (5 specimens CEMT; 18 specimens MQCAZ); Majua (7 specimens CEMT; 48 specimens MQCAZ); Palma Real (2 specimens MECN; 42 specimens MQCAZ); Playa de Oro (4 specimens CEMT; 81 specimens MQCAZ); Playa de Oro, La Tabla (7 specimens CEMT; 67 specimens MQCAZ); Playa de Oro, Padre Santo (21 specimens CEMT; 85 specimens MQCAZ; 4 speicmens MECN); Playa de Oro, Pote (4 specimens CEMT; 48 specimens MQCAZ; 7 specimens MECN); Tsejpi, Charco Grande (3 specimens CEMT; 28 specimens MQCAZ; 3 specimens MECN). IMBABURA: Lita, 680 m (18 specimens MQCAZ). LOS RÍOS: 47 km S de Santo Domingo, Río Palenque Biológical Station, 200–250 m (205 specimens MQCAZ); Río Palenque Station (44 specimens CEMT; 79 specimens MQCAZ). MANABÍ: Embalse Daule Peripa B.P Carrizal Chone, 110 m (2 specimens MUTPL); Puerto López, Las Tunas, 200 m (1 specimen MUTPL). PICHINCHA: Río Guayllabamba Llurimaguas, 290 m (2 specimens MUTPL); Río Guayllabamba Tortugo, 450 m (3 specimens MUTPL). SANTA ELENA: Olón, 10 m (8 specimens CEMT; 29 specimens MUTPL). SANTO DOMINGO DE LOS TSÁCHILAS: Santo Domingo, Puerto Limón, 340 m (23 specimens MUTPL).

####### Literature records.

GUAYAS: Bucay (Boucomont 1932: 320). PICHINCHA: without specific locality (Boucomont 1932: 320).

####### Temporal data.

Collected every month of the year.

####### Remarks.

Inhabits coastal lowland evergreen forests, coastal lowland semi-deciduous forests, and coastal evergreen foothill forests from 8–1200 m a.s.l. Collected manually and with pitfall traps baited with carrion and human feces.

###### Onthophagus (Onthophagus) basicarinatus

Taxon classificationAnimaliaColeopteraScarabaeidae

Rossini, Vaz-de-Mello & Zunino, 2018

Plate 39B

Onthophagus basicarinatus Rossini, Vaz-de-Mello & Zunino, 2018a: 567 (original description. Type locality: COLOMBIA: AMAZONAS. Leticia, Isla Santa Sofia, 215 m), 547 (figures: 2e-g, 2m-q, 5b), 551–553 (characters in key), 568 (distribution).
Onthophagus basicarinatus : Rossini et al. 2018b: 10 (list of species from osculatii complex).

####### Type specimens.

*Onthophagusbasicarinatus* Rossini, Vaz-de-Mello & Zunino, 2018. The holotype (♂) is deposited at the CMNC (see Rossini et al. 2018a: 568). Locality: Leticia, Isla Santa Sofia, 215 m, not examined.

####### Distribution.

Colombia, Brazil, Ecuador, and Peru.

####### Literature records.

NAPO (= ORELLANA): Estación Cientifica Yasuní, 215 m (Rossini et al. 2018a: 569).

####### Temporal data.

Collected in September.

####### Remarks.

Inhabits the lowland evergreen forests in the Amazon region at 215 m a.s.l. The collection method is unknown.

###### Onthophagus (Onthophagus) bidentatus

Taxon classificationAnimaliaColeopteraScarabaeidae

Drapiez, 1819

Plate 39C

Onthophagus bidentatus Drapiez, 1819: 134 (original description. Type locality: Cayenne).
Onthophagus bidentatus : Gemminger and Harold 1869: 1026 (catalog); Harold 1880a: 33 (redescription, distribution); Gillet 1911a: 204 (catalog); Bruch 1911: 190 (cited for Argentina); Boucomont and Gillet 1927: 204 (catalog of species); Boucomont 1932: 304 (characters in key), 321 (distribution); Balthasar 1941: 352 (cited for Peru); Blackwelder 1944: 211 (list of species from Latin America); Balthasar 1951: 337 (cited for Peru); Roze 1955: 45 (cited for Venezuela); Vulcano and Pereira 1967: 564 (characters in key); Zunino and Halffter 1997: 161 (list of species); Medina et al. 2001: 139 (cited for Colombia); Pulido-Herrera and Zunino 2007: 97 (catalog of species); Ratcliffe et al. 2015: 195 (cited for Peru); Rossini et al. 2016: 496 (comment), 497 (figure 1A); Rossini et al. 2018b: 9 (list of species of the *hircus* complex).
Onthophagus (Onthophagus) bidentatus : Vaz-de-Mello 2000: 194 (cited for Brazil); Chamorro et al. 2018: 96 (cited for Ecuador).
Onthophagus bicornis Castelnau, 1840: 87 (original description); Gemminger and Harold 1869: 1026 (cited as synonym of Onthophagusbidentatus Drapiez, cited as Onthophagusbicornis Beaud. Lafarge); Harold 1880a: 33 (cited as synonym of Onthophagusbidentatus Drap); Gillet 1911a: 204 (cited as synonym of Onthophagusbidentatus Drap, cited as O.bicornis Cast.); Boucomont and Gillet 1927: 204 (cited as synonym of Onthophagusbidentatus Drap., cited as O.bicornis Cast.); Blackwelder 1944: 211 (cited as synonym of Onthophagusbidentatus Drap., cited as O.bicornis Lap.); Pulido-Herrera and Zunino 2007: 97 (cited as synonym of Onthophagusbidentatus Drapiez, see fide Blackwelder 1944, cited as Onthophagusbicornis Laporte de Castelnau); Rossini et al. 2016: 496 (cited as synonym of Onthophagusbidentatus Drapiez, 1819).
Onthophagus femoralis Kirsch, 1871: 346 (original description); Harold 1880a: 33 (cited as synonym of Onthophagusbidentatus Drap.); Gillet 1911a: 204 (cited as synonym of Onthophagusbidentatus Drap.); Boucomont and Gillet 1927: 204 (cited as synonym of Onthophagusbidentatus Drap.); Blackwelder 1944: 211 (cited as synonym of Onthophagusbidentatus Drap. cited as O.femoralis Kirsch); Pulido-Herrera and Zunino 2007: 97 (cited as synonym of Onthophagusbidentatus Drapiez, see *fide*Blackwelder 1944, cited as Onthophagusfemoralis Kirsch).
Onthophagus semichalcites d’Orbigny, 1902: 149 (original description); Gillet, 1911a: 195 (cited for Nigeria); Boucomont and Gillet 1927: 195 (cited for Nigeria); Rossini et al. 2016: 496 (comment incorrect provenance, cited as synonym of Onthophagusbidentatus Drapiez, 1819).

####### Type specimens.

*Onthophagusbidentatus* Drapiez, 1819. The lectotype (♀) is deposited at the MNHN (see Rossini et al. 2016: 498). Locality: Cayenna, not examined.

*Onthophagusbicorni*s Castelnau, 1840. The lectotype (♀) is deposited at the MNHN (see Rossini et al. 2016: 498). Locality: Cayenna (not examined).

*Onthophagusfemoralis* Kirsch, 1871. Type material not examined.

*Onthophagussemichalcites* d’Orbigny, 1902. The lectotype (♂) is deposited at the MNHN Locality: Benin (incorrect provenance, see Rossini et al. 2016: 498), (not examined).

####### Distribution.

Argentina, Colombia, Brazil, Ecuador, Guadalupe, Guyana, Peru, and Venezuela.

####### Records examined.

NAPO: 5 km NE Carlos Julio Arosemena Tola, 800 m (1 specimen CEMT). ZAMORA CHINCHIPE: road El Chorro-La Chonta, 1000 m (3 specimens CEMT).

####### Temporal data.

Collected in April and May.

####### Remarks.

Inhabits the evergreen foothill forests of the Amazon region from 800–1000 m a.s.l. Collected manually.

###### Onthophagus (Onthophagus) confusus

Taxon classificationAnimaliaColeopteraScarabaeidae

Boucomont, 1932

Plate 39D

Onthophagus ophion var. confusus Boucomont, 1932: 306 (original description. Type locality: Bolivie, Équator).
Onthophagus ophion var. confusus : Blackwelder 1944: 211 (list of species for Latin America); Vulcano and Pereira 1967: 564 (characters in key); Carvajal et al. 2011: 322–323 (cited for Ecuador); Rossini et al. 2018b: 10 (list of species from osculatii complex); Chamorro et al. 2018: 97 (cited for Ecuador).
Onthophagus confusus : Rossini et al. 2018a: 549 (figures: 4a-d, 4j-n, 5a), 552–553 (characters in key), 573 (cited as new status), 574 (redescription), 575 (distribution).
Onthophagus nabeleki Balthasar, 1939h: 43 (original description); Martínez 1947: 112 (distribution); Zunino and Halffter 1997: 161 (list of species); Medina et al. 2001: 140 (cited for Colombia); Pulido-Herrera and Zunino 2007: 110 (catalog of species); Carvajal et al. 2011: 322–323 (cited for Ecuador); Krajcik 2012: 182 (complete list of species); Bezdek and Hajek 2013: 414 (catalog of the types of the NMPC); Rossini et al. 2018a: 549 (figures: 4e-f), 576 (cited as junior synonym of O.confusus Boucomont, 1932); Chamorro et al. 2018: 97 (cited for Ecuador).

####### Types specimens.

*Onthophagusconfusus* Boucomont, 1932. The lectotype (♂) is deposited at the MNHN (see Rossini et al. 2018a: 575). Locality: Huigra 1000 m (not examined).

*Onthophagusnabeleki* Balthasar, 1939. The lectotype (♀) is deposited at the SMTD (see Rossini et al. 2018a: 576). Locality: Ecuador (not examined).

####### Distribution.

Ecuador and Peru.

####### Records examined.

AZUAY: Huigra, 1000 (3 specimens MNHN). BOLIVAR: Balzapamba (1 specimen MNHN); Chimbo (2 specimens SMTD). COTOPAXI: Las Pampas, 1800 m (4 specimens CEMT); Otonga, 1800 m (4 specimens CEMT). IMBABURA: Paramba [= Parambas] (3 specimens SMTD). EL ORO: 10 km S de Portovelo (1 specimen CEMT); Piñas, 1200 m (26 specimens CEMT). GUAYAS: Bosque Protector Cerro Blanco, 350 m (27 specimens CEMT); Bucay (1 specimen MSMF). LOJA: 5 km N de Zambi, 1300 m (1 specimen CEMT); Gonzanama, 2000 m (2 specimens CEMT); Jimbura, 2100 m (2 specimens CEMT); Landangui (1 specimen MNHN); Piscobamba (1 specimen MSMF); without specific locality (1 specimen MNHN). PICHINCHA: 5 km SE de Nanegalito (2 specimens CEMT); Mindo, 1200–1500 m (3 specimens CEMT); Nanegalito, 1500 m (1 specimen CEMT); San José de Minas, 2400 m (8 specimens CEMT). SANTA ELENA: Olón, 10 m (291 specimens CEMT).

####### Literature records.

AZUAY: Huigra (Bezdek and Hajek 2013: 414). GUAYAS: Guayaquil (Bezdek and Hajek 2013: 414); Rio Pucay, Bucay, 300 m (Rossini et al. 2018a: 577). GUAYAS [= PICHINCHA]: 30 km NNE Playas, Tinalandia, 680 m (Rossini et al. 2018a: 577). EL ORO: Arenillas, 13 m (Rossini et al. 2018a: 577); Huairapongo [= Huayrapongo] (Bezdek and Hajek 2013: 414); Palestina, 25 km N de Daule, 30 m (Rossini et al. 2018a: 577). LOJA: Ciano (Bezdek and Hajek 2013: 414); Macará-Catacocha, 650 m (Rossini et al. 2018a: 577). ESMERALDAS: Esmeraldas (Rossini et al. 2018a: 577); San Mateo (Rossini et al. 2018a: 577). LOS RÍOS: Quevado [= Quevedo], Pichilingue (Rossini et al. 2018a: 577). MANABÍ: 20 km N Chone, 300 m (Rossini et al. 2018a: 577); Chone (Rossini et al. 2018a: 577). PICHINCHA [= SANTO DOMINGO DE LOS TSÁCHILAS]: 34 km de Santo Domingo de los Colorados, 2000 m (Rossini et al. 2018a: 577); Santo Domingo de Los Colorados (Rossini et al. 2018a: 577); 4 km SE Santo Domingo, 500 m (Rossini et al. 2018a: 577); Tinalandia, 780 m (Rossini et al. 2018a: 577). SANTA ELENA: 27 km S Puerto López, 76 km N Santa Elena, 500 m (Rossini et al. 2018a: 577); Manglar Alto (Rossini et al. 2018a: 577). SANTO DOMINGO DE LOS TSÁCHILAS [= LOS RÍOS]: 47 km S Santo Domingo, Rio Palenque Station, 230–250 m (Rossini et al. 2018a: 577). SANTO DOMINGO DE LOS TSÁCHILAS: Puerto Limón, 397 m (Rossini et al. 2018a: 577).

####### Temporal data.

Collected in January, April, May, June, July, August, September, October, and December.

####### Remarks.

Inhabits coastal lowland semi-deciduous forests and coastal evergreen foothill forests from 10–1200 m a.s.l. In the Andean region, it was recorded in the evergreen lower montane forests and the montane cloud forests from 1300–2400 m a.s.l. Species was collected with pitfall traps baited with pig feces and human feces.

###### Onthophagus (Onthophagus) coscineus

Taxon classificationAnimaliaColeopteraScarabaeidae

Bates, 1887

Plate 40A

Onthophagus coscineus Bates, 1887: 79 (original description. Type locality: PANAMA, volcán de Chiriqui).
Onthophagus coscineus : Gillet 1911a: 205 (complete list of species); Boucomont and Gillet 1927: 205 (catalog of species); Boucomont 1932: 323 (distribution); Blackwelder 1944: 211 (list of species from Latin America); Howden and Young 1981: 95 (characters in key), 111 (redescription); Kohlmann and Solís 2001: 163 (characters in key), 187 (redescription); Medina et al. 2001: 139 (list of species for Colombia); Ratcliffe 2002: 17 (cited for Panama); Pulido-Herrera and Zunino 2007: 102 (cited as synonym of Onthophagusdigitifer Boucomont, 1932); Carvajal et al. 2011: 322–323 (cited for Ecuador); Krajcik 2012: 177 (complete list of species); Solís and Kohlmann 2012: 8 (list of species from Costa Rica); Ratcliffe et al. 2015: 195 (cited for Peru); Delgado and Curoe 2014: 64 (characters in key, cited for Panama).
Onthophagus (Onthophagus) coscineus : Chamorro et al. 2018: 96 (cited for Ecuador).

####### Type specimens.

*Onthophaguscoscineus* Bates, 1887. The lectotype is deposited at the NHML (see Kohlmann and Solís 2001: 187). Locality: V de Chiriquí 25–4000 feet [= 760–1220 m], not examined.

####### Distribution.

Colombia, Costa Rica, Ecuador, and Panama.

####### Records examined.

CARCHI: Tobar Donoso, 300 m (2 specimens MECN); ESMERALDAS: Gualpi (1 specimen CEMT); SANTO DOMINGO DE LOS TSÁCHILAS: Santo Domingo, Puerto Limón, 395 m (1 specimen CEMT).

####### Temporal data.

Collected in March, April, September, and October.

####### Remarks.

Inhabits coastal lowland evergreen forests from 300–395 m a.s.l. Collected with pitfall traps baited with human feces.

###### Onthophagus (Onthophagus) curvicornis

Taxon classificationAnimaliaColeopteraScarabaeidae

Latreille, 1811

Plate 40B

Onthophagus curvicornis Latreille, 1811: 220 (original description. Type locality: Quito).
Onthophagus curvicornis : Gemminger and Harold 1869: 1028 (catalog); Taschenberg 1870: 184 (distribution); Harold 1880a: 29 (redescription, distribution); Gillet 1911a: 205 (catalog); Campos 1921: 57 (cited for Ecuador); Boucomont and Gillet 1927: 205 (catalog of species); Boucomont 1932: 308 (characters in key), 323 (distribution); Blackwelder 1944: 211 (list of species from Latin America); Guêrin 1953: 262 (diagnosis); Roze 1955: 45 (cited for Venezuela); Vulcano and Pereira 1967: 565 (characters in key); Zunino and Halffter 1997: 161 (list of species); Medina et al. 2001: 139 (cited for Colombia); Morón 2003: 71 (cited for Mexico); Pulido-Herrera and Zunino 2007: 101 (catalog of species); Carvajal et al. 2011: 322–323 (cited for Ecuador); Krajcik 2012: 177 (complete list of species); Rossini et al. 2018b: 9 (list of species of the curvicornis complex).
Onthophagus (Onthophagus) curvicornis : Vaz-de-Mello 2000: 194 (cited for Brazil); Chamorro et al. 2018: 97 (cited for Ecuador).
Onthophagus minax Kirsch, 1866: 215 (original description); Gemminger and Harold 1869: 1028 (cited as synonym of Onthophaguscurvicornis Latreille, 1811); Gillet 1911a: 205 (cited as synonym of Onthophaguscurvicornis Latreille, 1811); Boucomont and Gillet 1927: 205 (cited as synonym of Onthophaguscurvicornis Latreille, 1811); Blackwelder 1944: 211 (cited as synonym of Onthophaguscurvicornis Latreille, 1811); Pulido-Herrera and Zunino 2007: 101 (cited as synonym of Onthophaguscurvicornis Latreille, 1811).

####### Type specimens.

*Onthophaguscurvicornis* Latreille, 1811. Type material not examined.

*Onthophagusminax* Kirsch, 1866. Type material not examined.

####### Distribution.

Colombia, Ecuador, and Venezuela.

####### Records examined.

CHIMBORAZO: 2 km S de Puela, 2315 m (3 specimens CEMT). COTOPAXI: Otonga, 2200 m (1 specimen CEMT). EL ORO: Piñas, 1200 m (3 specimens CEMT). LOJA: 5 km N of Zambi, 1300 m (1 specimen CEMT); 10 km N of Zambi, 1850 m (1 specimen CEMT); Gonzanama, 2000 m (1 specimen CEMT); Las Chinchas, 2100 m (3 specimens CEMT); Loja, Villonaco, 2600 m (2 specimens CEMT). PICHINCHA: Quito, Nayón, 2500 m (8 specimens MUTPL). TUNGURAHUA: 10 km SW Baños, 2880 m (1 specimen CEMT).

####### Literature records.

PICHINCHA: Quito (Latreille 1811: 220; Pulido-Herrera and Zunino 2007: 101). LOJA: without specific locality (Taschenberg 1870: 184).

####### Temporal data.

Collected in February, March, April, May, August, and December.

####### Remarks.

Inhabits the evergreen foothill forests in the Amazonian range at 1300 m a.s.l. In the Andean region, it was registered for the montane cloud forests and the high evergreen montane forests from 1850–2880 m a.s.l. Collected manually and in dog feces.

###### Onthophagus (Onthophagus) cyanellus

Taxon classificationAnimaliaColeopteraScarabaeidae

Bates, 1887

Plate 40C

Onthophagus cyanellus Bates, 1887: 81 (original description. Type locality: MEXICO: Parada, Jalapa; GUATEMALA: San Jerónimo, Cerro Zunil; COSTA RICA: Río Sucio, Volcán de Irazu; PANAMA, Bugabá, Volcán de Chiriqui).
Onthophagus cyanellus : Gillet 1911a: 205 (complete list of species); Campos 1921: 57 (cited for Ecuador); Boucomont and Gillet 1927: 205 (catalog of species); Boucomont 1932: 323 (distribution); Blackwelder 1944: 211 (list of species for Latin America); Howden and Young 1981: 95 (characters in key), 114 (redescription); Zunino and Halffter 1988: 131 (redescription); 134 (distribution and biology); Kohlmann and Solís 2001: 164 (characters in key), 196 (redescription); Ratcliffe 2002: 17 (cited for Panama); Morón 2003: 69 (cited for Mexico); Pulido-Herrera and Zunino 2007: 101 (cited for Ecuador); Carvajal et al. 2011: 322–323 (cited for Ecuador); Krajcik 2012: 177 (complete list of species); Solís and Kohlmann 2012: 8 (list of species for Costa Rica); Delgado and Curoe 2014: 66 (characters in key, cited for Panama).
Onthophagus (Onthophagus) cyanellus : Chamorro et al. 2018: 97 (cited for Ecuador).

####### Type specimens.

*Onthophaguscyanellus* Bates, 1887. The lectotype is deposited at the NHML (see Zunino and Halffter 1988: 132). Locality: V de Chirirquí, 1300–2000 m, Panamá, not examined.

####### Distribution.

Colombia, Costa Rica, Ecuador, Guatemala, Mexico, Nicaragua, and Panama.

####### Literature records.

BOLIVAR: Chimbo (Campos 1921: 57). GUAYAS: Bucay (Campos 1921: 57).

####### Temporal data.

It is not known when this species was collected.

####### Remarks.

It is possible that this species may be found in coastal evergreen foothill forests. Campos (1921) reported this species from Chimbo and Bucay. However, we did not find any other record of this species in the collections examined. The collection method is unknown.

###### Onthophagus (Onthophagus) dicranius

Taxon classificationAnimaliaColeopteraScarabaeidae

Bates, 1887

Plate 40D

Onthophagus dicranius Bates, 1887: 72 (original description. Type locality: PANAMA, Bugaba).
Onthophagus dicranius : Gillet 1911a: 205 (complete list of species); Boucomont and Gillet 1927: 205 (catalog of species); Boucomont 1932: 298 (characters in key), 324 (distribution); Blackwelder 1944: 211 (list of species from Latin America); Howden and Young 1981: 96 (characters in key), 112 (redescription); Howden and Gill 1993: 1093 (characters in key); 1094 (redescription); Zunino and Halffter 1997: 165 (list of species); Kohlmann and Solís 2001: 164 (characters in key), 201 redescription); Medina et al. 2001: 139 (cited for Colombia); Ratcliffe 2002: 17 (cited for Panama); Morón 2003: 74 (cited for Mexico); Pulido-Herrera and Zunino 2007: 102 (catalog of species, distribution); Carvajal et al. 2011: 322–323 (cited for Ecuador); Krajcik 2012: 178 (complete list of species); Solís and Kohlmann 2012: 8 (cited for Costa Rica); Delgado and Curoe 2014: 65 (characters in key, cited for Panama).
Onthophagus (Onthophagus) dicranius : Chamorro et al. 2018: 97 (cited for Ecuador).

####### Type specimens.

*Onthophagusdicranius* Bates, 1887. The lectotype is deposited at the NHML (see Kohlmann and Solís 2001: 201). Locality: Bugabá 800–1000 feet [= 240–805 m], not examined.

####### Distribution.

Colombia, Costa Rica, Ecuador, and Panama.

####### Records examined.

LOS RIOS: CCRP [= Centro Científico Río Palenque] (5 specimens CEMT).

####### Literature records.

PICHINCHA [= SANTO DOMINGO DE LOS TSÁCHILAS]: Santo Domingo de los Colorados (Howden and Gill 1993: 1093).

####### Temporal data.

It is unknown when this species was collected.

####### Remarks.

Inhabits coastal lowland evergreen forests. The collection method is unknown; however, according to Howden and Young (1981), this species was collected in Panama using rotten fruit of *Gustavia* sp. and human feces. There are specimens housed at the CEMT labelled as ECUADOR, Los Ríos CCRP (possibly CCRP is the accronym for Centro Científico Río Palenque; also known as Estación Científica Río Palenque).

###### Onthophagus (Onthophagus) dicranoides

Taxon classificationAnimaliaColeopteraScarabaeidae

Balthasar, 1939

Plate 41A

Onthophagus dicranoides Balthasar, 1939h: 43 (original description. Type locality: Bucai [= Bucay], Guayaquil).
Onthophagus dicranoides : Martínez 1947: 112 (distribution); Zunino and Halffter 1997: 165 (list of species); Pulido-Herrera and Zunino 2007: 102 (catalog of species); Carvajal et al. 2011: 322–323 (cited for Ecuador); Krajcik 2012: 178 (complete list of species); Bezdek and Hajek 2013: 404 (catalog of the types of the NMPC).
Onthophagus (Onthophagus) dicranoides : Chamorro et al. 2018: 97 (cited for Ecuador).

####### Type specimens.

*Onthophagusdicranoides* Balthasar, 1939. Five syntypes examined deposited at the NMPC and MSMF. Lectotype to be designated in a future work on this species group.

**Syntype** (♂): “W Ecuador / Pucay / F. Ohaus S. [p]”, “Bucay 300 m / F. Ohs. 12.6.05 [p]”, “P.G. 3345 / Canada balsam / M. Zunino 1980 [hw]”, “TYPUS [p, red label, black margin]”, “Onthophagus / dicranioides / Dr. V. Balthasar det. [p and hw]”, “Mus. Nat. Pragae / 26326 / Inv. [p and hw, orange label]”, “dicranioides m. [hw, green label, black margin]”.

**Syntype** (♀): “Bucay 300 m / F. Ohs. 12.6.05 [p]”, “P.G. 3346 / Canada balsam / M. Zunino 1980 [hw]”, “TYPUS [p, red label, black margin]”, “Onthophagus / dicranioides / n. sp / Dr. V. Balthasar det. [p and hw]”, “Mus. Nat. Pragae / 26327 / Inv. [p and hw, orange label]”.

**Syntype** (♀): “Bucay 300 m / F. Ohs. 20.6.05 [p]”, “Onthophagus / dicranioides / n. sp Typ. / Dr. V. Balthasar det. [p and hw]”, “Senckenberg- / Museum / Frankfurt / Main [p]”, “TYPUS [p, red label, black margin]”, “Senckenberg / Museum [p]”.

**Syntype** (♂): “Bucay 300 m / F. Ohs. 20.6.05 [p]”, “Onthophagus / dicranioides / n. sp Typ. / Dr. V. Balthasar det. [p and hw]”, “Senckenberg- / Museum / Frankfurt / Main [p]”, “TYPUS [p, red label, black margin]”, “Senckenberg / Museum [p]”.

**Syntype** (♂): “W Ecuador / Guayaquil [p]”, “Onthophagus / dicranioides / n. sp. / Dr. V. Balthasar det. [p and hw]”, “Senckenberg- / Museum / Frankfurt / Main [p]”, “Para- / typoid [p, black label]”.

####### Distribution.

Only known from Ecuador.

####### Records examined.

CAÑAR: La Troncal (2 specimens CEMT). EL ORO: Piñas, 1200 m (9 specimens CEMT). GUAYAS: Pucay [= Bucay], 300 m (2 specimens NMPC; 2 specimens MSMF); Guayaquil (1 specimen MSMF). LOS RÍOS: CCRP [= Centro Científico Río Palenque] (13 specimens CEMT); Río Palenque Station (1 specimen CEMT). SANTO DOMINGO DE LOS TSÁCHILAS: Santo Domingo de los Colorados (1 specimen CEMT).

####### Temporal data.

Collected in January, February, March, May, June, and September.

####### Remarks.

Inhabits coastal lowland evergreen forests and evergreen foothill forests from 50–1200 m a.s.l. The collection method is unknown.

###### Onthophagus (Onthophagus) digitifer

Taxon classificationAnimaliaColeopteraScarabaeidae

Boucomont, 1932

Plate 41B

Onthophagus digitifer Boucomont, 1932: 324 (original description. Type locality: Pérou, Puno Chanchamayo, Vilcanota; Colombie orientale, Huaso).
Onthophagus digitifer : Balthasar 1941: 352 (cited for Peru); Blackwelder 1944: 211 (list of species from Latin America); Balthasar 1951: 337 (cited for Peru); Howden and Young 1981: 111 (cited as synonym of Onthophaguscoscineus Bates, 1887); Kohlmann and Solís 2001: 187 (cited as synonym of Onthophaguscoscineus Bates, 1887); Ratcliffe 2002: 17 (cited as synonym of Onthophaguscoscineus Bates, 1887); Pulido-Herrera and Zunino 2007: 102 (catalog of species, distribution); Solís and Kohlmann 2012: 8 (cited as synonym of Onthophaguscoscineus Bates, 1887).
Onthophagus (Onthophagus) digitifer : Chamorro et al. 2018: 97 (cited for Ecuador).

####### Type specimens.

*Onthophagusdigitifer* Boucomont, 1932. Five syntypes examined deposited at the MNHN and SMTD (coll. C Felsche, ex coll. A Boucomont). Lectotype to be designated in a future work on this species group.

####### Distribution.

Colombia, Ecuador, and Peru.

####### Records examined.

ORELLANA: Estación de Biodiversidad Tiputini USFQ, 215 m, Parque Nacional Yasuní (5 specimens CEMT; 1 specimen MUTPL). SUCUMBÍOS: El Dorado de Cascales Pozo Mascarey, 395 m (1 specimen MUTPL).

####### Temporal data.

Collected in May and June.

####### Remarks.

Inhabits the lowland evergreen forests of the Amazon region from 215–395 m a.s.l. Collected with flight interception traps and pitfall traps baited with carrion and human feces.

###### Onthophagus (Onthophagus) embrikianus

Taxon classificationAnimaliaColeopteraScarabaeidae

Paulian, 1936

Plate 41C

Onthophagus embrikianus Paulian, 1936b: 507 (original description. Type locality: Colombie et d’Equateur [= Colombia and Ecuador]).
Onthophagus embrikianus : Vulcano and Pereira 1967: 564 (characters in key); Zunino and Halffter 1997: 161 (list of species); Medina et al. 2001: 139 (cited for Colombia); Pulido-Herrera and Zunino 2007: 103 (catalog of species, distribution); Carvajal et al. 2011: 322–323 (cited for Ecuador); Krajcik 2012: 178 (complete list of species); Rossini et al. 2018b: 10 (comment).
Onthophagus (Onthophagus) embrikianus : Chamorro et al. 2018: 97 (cited for Ecuador).

####### Type specimens.

*Onthophagusembrikianus* Paulian, 1936. Two syntypes examined deposited in MNHN. Lectotype to be designated in a future work on this species group.

####### Distribution.

Colombia and Ecuador.

####### Records examined.

GUAYAS: Bucay (2 specimens MNHN).

####### Literature records.

PICHINCHA: without specific locality (Pulido-Herrera and Zunino 2007: 103).

####### Temporal data.

It is not known when this species was collected.

####### Remarks.

Inhabits coastal lowland evergreen forests. There are no other records of this species in the collections visited by the authors. The collection method is unknown.

###### Onthophagus (Onthophagus) insularis

Taxon classificationAnimaliaColeopteraScarabaeidae

Boheman, 1858

Plate 41D

Onthophagus insularis Boheman, 1858: 47 (original description. Type locality: Insula Taiti).
Onthophagus insularis : Gemminger and Harold 1869: 1031 (catalog); Gillet 1911a: 213 (catalog); Boucomont and Gillet 1927: 213 (catalog of species); Krajcik 2012: 180 (complete list of species); Rossini et al. 2018a: 549 (figures: 4g-i, 4o-r, 5b), 552–553 (characters in key), 578 (redescription), 579 (distribution); Rossini et al. 2018b: 10 (list of species of the osculatii complex).

####### Type specimens.

*Onthophagusinsularis* Boheman, 1858. The lectotype (♂) is deposited at the NHRS (see Rossini et al. 2018a: 580). Locality: Taiti, not examined.

####### Distribution.

Supposedly known only from Ecuador.

####### Literature records.

GUAYAS: Probably Ecuador, around Guayaquil (Rossini et al. 2018a: 579).

####### Temporal data.

It is not known when this species was collected.

####### Remarks.

According to Rossini et al. (2018a), it is possible that this species may be found in the Pacific coastal lowland semi-deciduous forests. The collection method is unknown.

###### Onthophagus (Onthophagus) lojanus

Taxon classificationAnimaliaColeopteraScarabaeidae

Balthasar, 1939

Plate 42A

Onthophagus lojanus Balthasar, 1939h: 44 (original description. Type locality: Loja in den Ostkordillieren).
Onthophagus lojanus : Martínez 1947: 112 (distribution); Pulido-Herrera and Zunino 2007: 108 (catalog of species); Carvajal et al. 2011: 322–323 (cited for Ecuador); Krajcik 2012: 181 (complete list of species); Bezdek and Hajek 2013: 412 (catalog of the types of the NMPC).
Onthophagus (Onthophagus) lojanus : Chamorro et al. 2018: 97 (cited for Ecuador).

####### Type specimens.

*Onthophaguslojanus* Balthasar, 1939. Four syntypes examined deposited at the MSMF and NMPC. Lectotype to be designated in a future work on this species group.

**Syntype** (♂): “Loja Ostcordill. / Sabanilla / F. Ohs. 18.9.05 [p]”, “Onthophagus / lojanus / n. sp. Typ. / Dr. V. Balthasar det. [p and hw]”, “Senckenberg- / Museum / Frankfurt / Main [p]”, “Typus [p, black margin]”.

**Syntype** (♂): “Loja Ostcordill. / Sabanilla / F. Ohs. 1.10.05 [p]”, “Onthophagus / lojanus n. sp. / Type / Dr. V. Balthasar det. [p and hw]”, “Senckenberg- / Museum / Frankfurt / Main [p]”, “Para- / typoid [p, red label, black margin]”.

**Syntype** (♂): “Loja Ostcordill. / Sabanilla / Arsen 5.10.05 [p]”, “P.G. 3344 / Canada balsam / M. Zunino 1980 [hw]”, “TYPUS ! [p, red label, black margin]”, “Onthophagus / lojanus n. sp. / Dr. V. Balthasar det. [p and hw]”, “Mus. Nat. Pragae / 26224/ Inv. [p and hw, orange label]”, “lojanus / m. [hw, green label, black margin]”.

**Syntype** (♀): “Loja Ostcordill. / Sabanilla / F. Ohs. 29.9.05 [p]”, “P.G. 3343 / Canada balsam / M. Zunino 1980 [hw]”, “TYPUS [p, red label, black margin]”, “Onthophagus / lojanus n. sp. / Dr. V. Balthasar det. [p and hw]”, “Mus. Nat. Pragae / 26223 / Inv. [p and hw, orange label]”.

####### Distribution.

Only known from Ecuador.

####### Records examined.

LOJA [= ZAMORA CHINCHIPE]: Ostcordill Sabanilla [= currently El Tambo] (2 specimens NMPC; 2 specimens MSMF).

####### Temporal data.

It is not known when this species was collected.

####### Remarks.

It is possible that this species may be found in montane cloud forests on the Andean slopes. Balthasar (1939h) reported this species in Sabanilla which is a locality in Zamora Chinchipe province. The collection method is unknown.

###### Onthophagus (Onthophagus) marginicollis

Taxon classificationAnimaliaColeopteraScarabaeidae

Harold, 1880

Plate 42B

Onthophagus marginicollis Harold, 1880a: 31 (original description. Type locality: Ambalema).
Onthophagus marginicollis : Gillet 1911a: 207 (catalog of species); Boucomont and Gillet 1927: 207 (catalog of species); Boucomont 1932: 303 (characters in key), 327 (distribution); Blackwelder 1944: 211 (list of species from Latin America); Vulcano and Pereira 1967: 563 (characters in key); Zunino and Halffter 1997: 161 (list of species); Vaz-de-Mello 2000: 194: (cited for Brazil); Medina et al. 2001: 139 (list of species from Colombia); Pulido-Herrera and Zunino 2007: 108 (catalog of species); Krajcik 2012: 182 (complete list of species); Ratcliffe et al. 2015: 195 (cited for Peru); Rossini et al. 2018b: 10 (list of species of the *hircus* complex), 11 (figure 3c); Delgado and Curoe 2014: 65 (characters in key, cited for Panama).
Onthophagus (Onthophagus) marginicollis : Chamorro et al. 2018: 97 (cited for Ecuador).

####### Types specimens.

*Onthophagusmarginicollis* Harold, 1880. Two syntypes examined deposited at the MNHN. Lectotype to be designated in a future work on this species group.

####### Distribution.

Brazil, Bolivia, Colombia, Costa Rica, Cuba, Ecuador, El Salvador, Guatemala, Guyana, Mexico, Nicaragua, Panama, Peru, and Venezuela.

####### Records examined.

ORELLANA: Coca-Napo (1 specimen MQCAZ).

####### Temporal data.

Collected in April.

####### Remarks.

Inhabits the lowland evergreen forests of the Amazon region. The collection method is unknown.

###### Onthophagus (Onthophagus) mirabilis

Taxon classificationAnimaliaColeopteraScarabaeidae

Bates, 1887

Plate 42C

Onthophagus mirabilis Bates, 1887: 74 (original description. Type locality: Ecuador, Río Morona).
Onthophagus mirabilis : Gillet 1911a: 207 (complete list of species); Boucomont and Gillet 1927: 207 (catalog of species); Boucomont 1932: 328 (distribution); Blackwelder 1944: 211 (list of species from Latin America); Howden and Young 1981: 96 (characters in key), 116 (redescription); Howden and Gill 1993: 1098 (characters in key, redescription); Zunino and Halffter 1997: 165 (list of species); Génier and Howden 1999: 131 (characters in key), 134 (comment); Medina et al. 2001: 140 (cited for Colombia); Génier and Medina 2004: 610 (description of the female), 611 (figure 1); Pulido-Herrera and Zunino 2007: 109 (catalog of species, distribution); Carvajal et al. 2011: 322–323 (cited for Ecuador); Krajcik 2012: 182 (complete list of species); Ratcliffe et al. 2015: 195 (cited for Peru); Génier 2017: 6 (characters in key, cited for Ecuador).
Onthophagus (Onthophagus) mirabilis : Chamorro et al. 2018: 97 (cited for Ecuador).

####### Type specimens.

*Onthophagusmirabilis* Bates, 1887. The holotype (♂) is deposited at the MNHN (ex coll. HW Bates) (see Génier and Howden 1999: 134). Locality: Río Morona, Ecuador, not examined.

####### Distribution.

Colombia, Ecuador, and Panama.

####### Records examined.

ZAMORA CHINCHIPE: Cordillera la Curintza, 1790 m (1 specimen CEMT); Tundayme, La Escombrera, 1225 m (2 specimens CEMT).

####### Literature records.

MORONA SANTIAGO: Río Morona (Bates 1887: 74; Génier and Howden 1999: 134).

####### Temporal data.

Collected in September and October.

####### Remarks.

Inhabits the foothill forests of the Amazon region at 1225 m a.s.l. In the Andean region, the species was recorded in the evergreen lower montane forest at 1790 m a.s.l. Collected with pitfall traps baited with human feces.

Génier and Howden (1999) reported that the specimen that was collected in Río Morona was partially covered with spider webs. Howden and Young (1981) and Génier and Medina (2004) reported that this species was collected using pitfall traps baited with carrion and feces, fruit traps, and flight interception traps.

###### Onthophagus (Onthophagus) nasutus

Taxon classificationAnimaliaColeopteraScarabaeidae

Guérin-Méneville, 1855

Onthophagus nasutus Guérin-Méneville, 1855: 589 (original description. Without type locality).
Onthophagus nasutus : Gemminger and Harold 1869: 1033 (catalog); Harold 1880a: 35 (distribution, cited for Nueva Granada [= Colombia]); Gillet 1911a: 207 (catalog); Boucomont and Gillet 1927: 207 (catalog of species); Boucomont 1932: 328 (distribution, cited for Ecuador); Blackwelder 1944: 211 (list of species for Latin America); Vulcano and Pereira 1967: 565 (characters in key); Zunino and Halffter 1997: 165 (list of species); Medina et al. 2001: 140 (cites for Colombia); Pulido-Herrera and Zunino 2007: 110 (catalog of species); Carvajal et al. 2011: 322–323 (cited for Ecuador); Krajcik 2012: 182 (complete list of species).
Onthophagus (Onthophagus) nasutus : Vaz-de-Mello, 2000: 194 (cited for Brazil); Chamorro et al. 2018: 97 (cited for Ecuador).

####### Type specimens.

*Onthophagusnasutus* Guérin-Méneville, 1855. Type material not examined.

####### Distribution.

Brazil, Colombia, and Ecuador.

####### Literature records.

LOJA: without specific locality (Boucomont 1932: 328).

####### Temporal data.

It is not known when this species was collected.

####### Remarks.

The habitat requirements and collection methods are unknown. There are no other records of this species in the collections examined.

###### Onthophagus (Onthophagus) onorei

Taxon classificationAnimaliaColeopteraScarabaeidae

Zunino & Halffter, 1997

Plate 42D

Onthophagus onorei Zunino & Halffter, 1997: 168 (original description. Type locality: Ecuador, Napo, La Joya de los Sachis [= Orellana, La Joya de los Sachas], 290 m).
Onthophagus onorei : Pulido-Herrera and Zunino 2007: 111 (catalog of species, distribution); Carvajal et al. 2011: 322–323 (cited for Ecuador); Krajcik 2012: 183 (complete list of species); Ratcliffe et al. 2015: 195 (cited for Peru); Rossini et al. 2018b: 10 (list of species from rubrescens complex).
Onthophagus (Onthophagus) onorei : Chamorro et al. 2018: 97 (cited for Ecuador, written as Onthophagus (Onthophagus) onoreZunino and Halffter 1997).

####### Type specimens.

*Onthophagusonorei* Zunino & Halffter, 1997. The holotype (♂) is deposited at the MZc (see Zunino and Halffter 1997: 169). Locality: Ecuador, Napo, La Joya de los Sachis, not examined.

####### Distribution.

Ecuador, Peru, and Bolivia.

####### Records examined.

ORELLANA: Dayuma plataforma Ungurahua, 300 m (1 specimen MUTPL). SUCUMBÍOS: El Dorado de Cascales, Pozo Aguas Blancas, 385 m (1 specimen MUTPL); El Dorado de Cascales, Pozo Diamante (1 specimen MUTPL); El Dorado de Cascales, Pozo cristal, 425 m (1 specimen MUTPL); Nueva Loja, plataforma Iguana, 310 m (1 specimen MUTPL); Pacayacu, Campo Libertador, 265 m (1 specimen MUTPL).

####### Literature records.

NAPO [= ORELLANA]: La Joya de los Sachis [= La Joya de los Sachas], 290 m (Zunino and Halffter 1997: 168).

####### Temporal data.

Collected in May, September, October, and December.

####### Remarks.

Inhabits the lowland evergreen forests of the Amazon region from 265–425 m a.s.l. Collected with pitfall traps baited with human feces.

###### Onthophagus (Onthophagus) osculatii

Taxon classificationAnimaliaColeopteraScarabaeidae

Guérin-Méneville, 1855

Plate 43A

Onthophagus osculatii Guérin-Méneville, 1855: 589 (original description. Without type locality).
Onthophagus osculatii : Gemminger and Harold 1869: 1034 (catalog, cited as OnthophagusOsculatii Guér); Harold 1880a: 30 (distribution, cited for Nueva Granada [= Colombia]); Gillet 1911a: 207 (catalog, cited as OnthophagusOsculatii Guér); Boucomont and Gillet 1927: 207 (catalog of species, cited as OnthophagusOsculatii Guér); Boucomont 1932: 305 (characters in key), 329 (distribution, cited for Ecuador); Blackwelder 1944: 212 (list of species from Latin America); Vulcano and Pereira 1967: 564 (characters in key); Zunino and Halffter 1997: 161 (list of species); Medina et al. 2001: 140 (cited for Colombia); Hamel-Leigue et al. 2006: 17 (cited for Bolivia); Pulido-Herrera and Zunino 2007: 112 (catalog of species); Carvajal et al. 2011: 322–323 (cited for Ecuador); Krajcik 2012: 183 (complete list of species); Ratcliffe et al. 2015: 195 (cited for Peru); Rossini et al. 2018a: 546 (figures: 1a-b, 1g-h, 1k-m, 5a), 551–553 (characters in key), 554 (redescription), 555 (distribution); Rossini et al. 2018b: 10 (list of species from osculatii complex).
Onthophagus (Onthophagus) osculatii : Vaz-de-Mello 2000: 194 (cited for Brazil); Chamorro et al. 2018: 97 (cited for Ecuador).

####### Type specimens.

*Onthophagusosculatii* Guérin-Méneville, 1855. The neotype (♂) is deposited at the IRSN (see Rossini et al. 2018a: 557). Locality: Brasil. Amazonas. BR 319 km 350, not examined.

####### Distribution.

Bolivia, Brazil, Colombia, Ecuador, French Guiana, Guyana, Peru, and Surinam.

####### Literature records.

NAPO: 20 km S de Tena (Rossini et al. 2018a: 559); Jatun Sacha Biological Station., 450 m (Rossini et al. 2018a: 559); same locality, 21 km E Puerto Napo, 400 m (Rossini et al. 2018a: 559). NAPO (= ORELLANA): Yasuní., 250 m (Rossini et al. 2018a: 559). ORELLANA: Estación Cientifica Yasuní, 215 m (Rossini et al. 2018a: 559); Tiputini (Rossini et al. 2018a: 560). PASTAZA: Villano (Rossini et al. 2018a: 559). UNDETERMINED PROVINCE: without specific locality (Boucomont 1932: 305).

####### Temporal data.

Collected in February, April, July, August, and September.

####### Remarks.

Inhabits the lowland evergreen forests of the Amazon region from 215–450 m a.s.l. According to Rossini et al. (2018a), this species has been collected with pitfall traps baited with carrion and human feces.

###### Onthophagus (Onthophagus) rubrescens

Taxon classificationAnimaliaColeopteraScarabaeidae

Blanchard, 1843

Plate 43B

Onthophagus rubrescens Blanchard, 1843: 183 (original description. Type locality: Yanacuche, Chupe et Chulumani, dans la province de Yungas).
Onthophagus rubrescens : Gemminger and Harold 1869: 1035 (catalog); Gillet 1911a: 208 (catalog); Boucomont and Gillet 1927: 208 (catalog of species); Blackwelder 1944: 212 (list of species from Latin America); Vulcano and Pereira 1967: 564 (characters in key); Zunino and Halffter 1997: 161 (distribution); Medina et al. 2001: 140 (list of species from Colombia); Hamel-Leigue et al. 2006: 17 (cited for Bolivia); Pulido-Herrera and Zunino 2007: 114 (catalog of species); Krajcik 2012: 185 (complete list of species); Ratcliffe et al. 2015: 195 (cited for Peru); Rossini et al. 2018b: 10 (list of species of the rubrescens complex).
Onthophagus rubrescens var. haematopus : Boucomont 1932: 304 (characters in key), 330 (distribution).
Onthophagus (Onthophagus) rubrescens : Vaz-de-Mello 2000: 194 (cited for Brazil); Chamorro et al. 2018: 97 (cited for Ecuador).

####### Type specimens.

*Onthophagusrubrescens* Blanchard, 1843. Two syntypes examined deposited at the MNHN. Lectotype to be designated in a future work on this species group.

####### Distribution.

Brazil, Bolivia, Colombia, Ecuador, Guyana, and Peru.

####### Records examined.

MORONA SANTIAGO: Bosque Domoso, 1650 m (1 specimen CEMT); Huambi, 900 m (2 specimens CEMT). NAPO: Tena (1 specimen CEMT). ORELLANA: SCYasuní [= Estación Científica Yasuní PUCE], 250 m (5 specimens CEMT); Río Tiputini, Parque Nacional Yasuní (1 specimen CEMT); Rodrigo Borja IAMOE (2 specimens CEMT). PASTAZA: Villano (3 specimens CEMT).

####### Literature records.

CHIMBORAZO: without specific locality (Boucomont 1932: 330). PICHINCHA: without specific locality (Boucomont 1932: 330).

####### Temporal data.

Collected in April, June, July, August, September, and October.

####### Remarks.

Inhabits the lowland evergreen forests, evergreen foothill forests and evergreen lower montane forests in the Amazonian range from 250–1650 m a.s.l. Collected manually, using flight interception traps and with pitfall traps baited with carrion and human feces. According to Boucomont (1932), this species was recorded in the Andean region. However, this record is possibly erroneous.

###### Onthophagus (Onthophagus) sharpi

Taxon classificationAnimaliaColeopteraScarabaeidae

Harold, 1875

Plate 43C

Onthophagus sharpi Harold, 1875d: 138 (original description. Without type locality).
Onthophagus sharpi : Gillet 1911a: 208 (catalog of species, cited as OnthophagusSharpi Har.); Boucomont and Gillet 1927: 208 (catalog of species, cited as OnthophagusSharpi Har.); Boucomont 1932: 300 (characters in key), 330 (distribution); Blackwelder 1944: 212 (list of species from Latin America); Halffter and Matthews 1966: 39 (cited for Panama); Howden and Young 1981: 97 (characters in key), 117 (redescription); Zunino and Halffter 1997: 165 (list of species); Kohlmann and Solís 2001: 166 (characters in key), 234 (redescription); Medina et al. 2001: 140 (cited for Colombia); Ratcliffe 2002: 17 (cited for Panama); Morón 2003: 74 (cited for Mexico); Pulido-Herrera and Zunino 2007: 115 (catalog of species, distribution); Carvajal et al. 2011: 322–323 (cited for Ecuador); Krajcik 2012: 185 (complete list of species); Solís and Kohlmann 2012: 9 (cited for Costa Rica); Delgado and Curoe 2014: 66 (characters in key, cited for Panama).
Onthophagus (Onthophagus) sharpi : Chamorro et al. 2018: 97 (cited for Ecuador).

####### Type specimens.

*Onthophagussharpi* Harold, 1875. The holotype is deposited at the MNHN (see Kohlmann and Solís 2001: 234). Locality: without specific locality, not examined.

####### Distribution.

Colombia, Costa Rica, Ecuador, Mexico, and Nicaragua.

####### Records examined.

PICHINCHA: Llurimaguas, Guayabilla Río Guayllabamba, 520 m (2 specimens CEMT); Tortugo Río Guayllabamba, 450 m (1 specimen CEMT).

####### Temporal data.

Collected in March and December.

####### Remarks.

Inhabits coastal lowland evergreen forests from 450–520 m a.s.l. Collected in aerial fruit traps.

###### Onthophagus (Onthophagus) steinheili

Taxon classificationAnimaliaColeopteraScarabaeidae

Harold, 1880

Plate 43D

Onthophagus steinheili Harold, 1880a: 34 (original description. Type locality: Fusagasugá).
Onthophagus steinheili : Gillet 1911a: 208 (catalog of species, cited as OnthophagusSteinheili Har.); Boucomont and Gillet 1927: 208 (catalog of species, cited as OnthophagusSteinheili Har.); Boucomont 1932: 304 (characters in key), 330 (distribution); Blackwelder 1944: 212 (list of species from Latin America); Vulcano and Pereira 1967: 563 (characters in key); Zunino and Halffter 1997: 161 (list of species); Medina et al. 2001: 140 (list of species from Colombia); Pulido-Herrera and Zunino 2007: 116 (catalog of species); Krajcik 2012: 186 (complete list of species); Rossini et al. 2018a: 548 (figures: 3e-h, 2n-r, 5a), 552–553 (characters in key), 572 (redescription), 573 (distribution); Rossini et al. 2018b: 10 (list of species of the osculatii complex).
Onthophagus (Onthophagus) steinheili : Chamorro et al. 2018: 97 (cited for Ecuador).

####### Types specimens.

*Onthophagussteinheili* Harold, 1880. The lectotype is deposited at the MNHN (see Rossini et al. 2018a: 573). Locality: Fusagasugá (not examined).

####### Distribution.

Colombia and Ecuador.

####### Records examined.

ZAMORA CHINCHIPE: Chito, Río San Francisco, 1800 m (2 specimens CEMT).

####### Temporal data.

Collected in February.

####### Remarks.

Inhabits the montane cloud forests of the Andean region at 1800 m a.s.l. Collected with pitfall traps baited with human feces.

###### Onthophagus (Onthophagus) stockwelli

Taxon classificationAnimaliaColeopteraScarabaeidae

Howden & Young, 1981

Plate 44A

Onthophagus stockwelli Howden & Young, 1981: 101 (original description. Type locality: Panama. Colón Prov., 270 m, 10 mi [= 16 km], SE Colón Santa Rita Ridge).
Onthophagus stockwelli : Kohlmann and Solís 2001: 167 (characters in key), 237 (redescription); Ratcliffe 2002: 17 (cited for Panama); Carvajal et al. 2011: 322–323 (cited for Ecuador); Krajcik 2012: 186 (complete list of species); Delgado and Curoe 2014: 66 (characters in key, cited for Panama); Rossini et al. 2018b: 9 (list of species from curvicornis complex).
Onthophagus (Onthophagus) stockwelli : Chamorro et al. 2018: 97 (cited for Ecuador).

####### Type specimens.

*Onthophagusstockwelli* Howden & Young, 1981. The holotype is deposited at the CMNC (ex coll. H Howden) (see Howden and Young 1981: 102). Locality: Panama. Colón Prov., 270 m, 10 mi, SE Colón Santa Rita Ridge, not examined.

####### Distribution.

Costa Rica, Ecuador, and Panama.

####### Records examined.

LOS RÍOS: Río Palenque Biológical Station, 250 m (5 specimens CEMT).

####### Literature records.

ESMERALDAS: 11 km SE San Lorenzo, La Chiquita, 5 m (Howden and Young 1981: 103). MANABÍ: 73 km NE Chone, 90 km W Santo Domingo, 300 m (Howden and Young 1981: 103). PICHINCHA: [= LOS RÍOS]: 47 km S Santo Domingo, Río Palenque Biológical Station (Howden and Young 1981: 103).

####### Temporal data.

Collected in February, May, June, and July

####### Remarks.

Inhabits coastal lowland evergreen forests and coastal evergreen foothill forests from 5–300 m a.s.l. Collected with pitfall traps baited with human feces.

###### Onthophagus (Onthophagus) transisthmius

Taxon classificationAnimaliaColeopteraScarabaeidae

Howden & Young, 1981

Plate 44B

Onthophagus transisthmius Howden & Young, 1981: 106 (original description. Type locality: Panama. Canal Zone, Gamboa Limbo Hunt Club).
Onthophagus transisthmius : Zunino and Halffter 1997: 161 (list of species); Medina et al. 2001: 140 (list of species for Colombia); Ratcliffe 2002: 17 (cited for Panama); Rossini et al. 2018a: 547 (figures: 2a-d, 2h-i, 5b), 552–553 (characters in key), 563 (redescription), 564 (distribution); Delgado and Curoe 2014: 65 (characters in key, cited for Panama); Rossini et al. 2018b: 10 (list of species of the osculatii complex).
Onthophagus (Onthophagus) transisthmius : Chamorro et al. 2018: 97 (cited for Ecuador).

####### Types specimens.

*Onthophagustransisthmius* Howden & Young, 1981. The holotype is deposited at the USNM (see Howden and Young 1981: 107). Locality: Panama. Canal Zone, Gamboa Limbo Hunt Club (not examined).

####### Distribution.

Bolivia, Colombia, Ecuador, Panama, Peru, and Venezuela.

####### Records examined.

MORONA SANTIAGO: road Mendez-Paute km 8 (1 specimen CEMT). PASTAZA: plataforma Villano (2 specimens CEMT); Villano (4 specimens CEMT).

####### Literature records.

NAPO: Jatun Sacha Biological Station, 450 m (Rossini et al. 2018a: 566), same locality, 21 km E Puerto Napo, 400 m (Rossini et al. 2018a: 566); Tena, 400 m (Rossini et al. 2018a: 566). NAPO [= ORELLANA]: La Joya de los Sachis [= Joya de los Sachas], 290 m (Rossini et al. 2018a: 566). SUCUMBÍOS: Limoncocha, 250 m (Rossini et al. 2018a: 566). TUNGURAHUA: 6 km W de Río Negro, 1200 m (Rossini et al. 2018a: 566).

####### Temporal data.

Collected in January, June, July, and August.

####### Remarks.

Inhabits the lowland evergreen forests and evergreen foothill forests of the Amazon region from 290–1200 m a.s.l. Collected with pitfall traps baited with human feces.

###### Onthophagus (Onthophagus) xanthomerus

Taxon classificationAnimaliaColeopteraScarabaeidae

Bates, 1887

Plate 44C

Onthophagus xanthomerus Bates, 1887: 69 (original description. Type locality: Amazonas, Ega [= Tefé]).
Onthophagus xanthomerus : Gillet 1911a: 208 (complete list of species); Boucomont and Gillet 1927: 208 (catalog of species); Blackwelder 1944: 212 (list of species from Latin America); Zunino 1981: 79 (redescription, distribution), Zunino and Halffter 1997: 165 (list of species); Medina et al. 2001: 140 (cited for Colombia); Pulido-Herrera and Zunino 2007: 119 (catalog of species, distribution); Carvajal et al. 2011: 322–323 (cited for Ecuador); Krajcik 2012: 188 (complete list of species); Ratcliffe et al. 2015: 195 (cited for Peru).
Onthophagus (Onthophagus) xanthomerus : Vaz-de-Mello 2000: 194 (cited for Brazil); Chamorro et al. 2018: 79 (figure 2B), 97 (cited for Ecuador).
Onthophagus canellinus Bates, 1887: 70 (original description); Gillet 1911a: 204 (complete list of species); Boucomont and Gillet 1927: 204 (catalog of species); Boucomont 1932: 299 (characters in key), 321 (distribution); Blackwelder 1944: 211 (list of species from Latin America); Zunino 1981: 79 (synonym of Onthophagusxanthomerus Bates, 1887); Pulido-Herrera and Zunino 2007: 119 (cited as synonym of Onthophagusxanthomerus Bates); Krajcik 2012: 176 (cited as species).

####### Types specimens.

*Onthophagusxanthomerus* Bates, 1887. Two syntypes examined deposited at the MNHN (ex coll. HW Bates and ex coll. R Oberthur). Lectotype to be designated in a future work on this species group.

*Onthophaguscanellinus* Bates, 1887. The holotype (♀) is deposited at the MNHN (see Zunino 1981: 79). Locality: Canelos, Equador (not examined).

####### Distribution.

Brazil, Colombia, Ecuador, and Peru.

####### Records examined.

MORONA SANTIAGO: Comunidad Ángel Rouby, 1300 m, Cordillera del Kutukú (5 specimens MECN); Comunidad Unsuants, 500–1100 m, Cordillera del Kutukú (13 specimens MECN); Nuevo Israel, 1290 m, Cordillera del Kutukú (2 specimens MUTPL). NAPO: Puerto Misahualli (2 specimens MECN); Shiqui cerca al Tena, 480 m, Pungarayacu (1 specimen MUTPL); Tena, 450 m (3 specimens MECN). ORELLANA: Bloque 31, 200 m Parque Nacional Yasuní (7 specimens MECN); Cononaco, Bloque 16 YPF, Parque Nacional Yasuní, 250 m (1 specimen MUTPL); plataforma Daimi 1 (1 specimen CEMT); Dayuma Campo Hormiguero, plataforma Hormiguero, 320 m (1 specimen MUTPL); SCYASUNI [= Estación Científica Yasuní PUCE, Parque Nacional Yasuní], 250 m (2 specimens CEMT); Estación de Biodiversidad Tiputini USFQ, 215 m, Parque Nacional Yasuní (1 specimen MUTPL); Lago San Pedro, plataforma Copal, 310 m (1 specimen MUTPL); Río Tiputini Yasuní Res Stn. (5 specimens CEMT). PASTAZA: Bosque Protector Oglán Alto, 660–809 m (2 specimens MUTPL); Chuyayacu km 25 Oleoducto, 200 m (1 specimen MUTPL); Mera, E. B. Pindo Mirador UTE, 1000 m (1 specimen CEMT). SUCUMBÍOS: Pacayacu Campo Libertador, Tapi, 260 m (1 specimen CEMT); Tarapoa Campo Marian, 260 m, plataforma Fanny 5 (1 specimen MUTPL); Tarapoa, Nuevo Manabí, 270 m (1 specimen MUTPL). ZAMORA CHINCHIPE: Tundayme campamento Ecsa, San Marcos, 900 m (1 specimen MUTPL); Tundayme, campamento Ecsa, vivero, 820 m (1 specimen MUTPL); Zurmi, Comunidad La Wants, 1010 m (1 specimen MEPN; 1 specimen MUTPL).

####### Literature records.

GUAYAS: Bucay (Boucomont 1932: 321). LOJA: without specific locality (Zunino 1981: 80). NAPO [= SUCUMBÍOS], 250 m, Limoncoche [= Limoncocha] (Zunino 1981: 80). PASTAZA: Canelos (Zunino 1981: 80). UNDETERMINED PROVINCE: Thimbo (Boucomont 1932: 321).

####### Temporal data.

Collected in all months except October.

####### Remarks.

Inhabits the lowland evergreen forests, evergreen foothill forests, and evergreen lower montane forests in the Amazonian range from 200–1500 m a.s.l. Collected with flight interception traps and pitfall traps baited with carrion, human feces and dead chilopods. According to Boucomont (1932), this species was recorded in the coastal region too (Bucay, Guayas). However, we consider this record possibly erroneous.

#### Genus *Oruscatus* Bates, 1870

*Oruscatus* Bates, 1870: 174 (original description. Type species: *Oruscatusdavus*Bates 1870).

*Oruscatus*: Gillet 1911a: 88 (catalog); Lucas 1920: 466 (catalog, distribution); d’Olsoufieff 1924: 17 (characters in key), 60 (redescription); Blackwelder 1944: 210 (list of species from Latin America); Martínez 1959: 106 (list of species for Argentina); Halffter and Matthews 1966: 257 (catalog, distribution); Vulcano and Pereira 1967: 565 (characters in key); Edmonds 1972: 816 (comment); Halffter and Edmonds 1982: 137 (catalog, distribution); Zunino 1985: 107 (comment); Medina and Lopera 2000: 305 (characters in key); Medina et al. 2001: 139 (list of species for Colombia); Philips et al. 2004: 50 (comment); Hamel-Leigue et al. 2006: 17 (list of species for Bolivia); Scholtz et al. 2009: 246 (evolutionary history); Vaz-de-Mello et al. 2011: 17 (characters in key); Carvajal et al. 2011: 144 (diagnosis), 322 (list of species from Ecuador); Krajcik 2012: 190 (complete list of species); Figueroa et al. 2014: 136 (distribution of records for Peru); Chamorro et al. 2018: 75 (characters in key), 97 (list of species from Ecuador).

##### Oruscatus opalescens

Taxon classificationAnimaliaColeopteraScarabaeidae

Bates, 1870

Plate 44D

Oruscatus opalescens Bates, 1870: 174 (original description. Type locality: Equador, prope Cuencam [= near Cuenca]).
Oruscatus opalescens : Harold 1880a: 27 (cited for Colombia); Gillet 1911a: 88 (catalog); d’Olsoufieff 1924: 21 (characters in key), 61 (redescription); Blackwelder 1944: 210 (list of species from Latin America); Vulcano and Pereira 1967: 566 (characters in key); Halffter and Edmonds 1982: 137 (catalog, distribution); Medina et al. 2001: 139 (cited for Colombia); Carvajal et al. 2011: 322–323 (cited for Ecuador); Krajcik 2012: 190 (complete list of species); Chamorro et al. 2014b: 288 (figures A, B, C, D), 289 (distribution), 290 (diagnosis); Chamorro et al. 2018: 82 (figure 5E), 83 (figure 6B), 97 (cited for Ecuador).

###### Type specimens.

*Oruscatusopalescens* Bates, 1870. Type material not examined.

###### Distribution.

Colombia and Ecuador.

###### Records examined.

LOJA: Loja (14 specimens MNHN); Parque Nacional Podocarpus (1 specimen CEMT). MORONA SANTIAGO: Macas (1 specimen MHNH); Tinajillas, 2140 m (1 specimen MQCAZ). NAPO: Oyacachi, Río Cedro, 3264–3320 m (2 specimens MUTPL). PICHINCHA: Quito (1 specimen MNHN). UNDETERMINED PROVINCE: without specific locality (8 specimens MNHN); without specific locality (1 specimen NHML).

###### Literature records.

AZUAY: prope Cuencam [= near Cuenca] (Bates 1870: 175).

###### Temporal data.

Collected in November.

###### Remarks.

Inhabits the montane cloud forests and the evergreen high montane forests of the Andean region from 2140–3320 m a.s.l. Collected in Andean tapir feces.

#### Genus *Oxysternon* Laporte, 1840

*Oxysternon* Laporte, 1840: 82 (original description. Type species: *Scarabeusfestivus* Linnaeus, 1767. Type subsequently designated by Edmonds 1972: 838).

*Oxysternon*: Agassiz 1846: 774 (catalog, unjustifiably cited as *Oxysternum*); Nevinson 1892: 8 (catalog, distribution); Gillet 1911a: 87 (catalog, distribution); Lucas 1920: 471 (catalog, distribution); d’Olsoufieff 1924: 18 (characters in key); 111 (redescription), 157 (distribution); Pessôa and Lane 1941: 470 (characters in key); Blackwelder 1944: 210 (list of species from Latin America); Halffter and Matthews 1966: 258 (catalog, distribution); Vulcano and Pereira 1967: 566 (characters in key); Edmonds 1972: 820 (characters in key), 835 (redescription); Howden and Young 1981: 11 (characters in key), 146 (redescription); Halffter and Edmonds 1982: 136 (catalog, distribution); Zunino 1985: 104 (comment); Edmonds 1994: 17 (characters in key); Medina and Lopera 2000: 303 (characters in key); Vitolo 2000: 595 (characters in key); Vaz-de-Mello 2000: 194 (list of species from Brazil); Medina et al. 2001: 140 (list of species from Colombia); Arnaud 2002a: 13 (characters in key), 61 (diagnosis); Ratcliffe 2002: 16 (list of species from Panama); Edmonds and Zídek 2004: 3 (revision); Vitolo 2004: 287 (diagnosis); Philips et al. 2004: 50 (comment); Hamel-Leigue et al. 2006: 17 (list of species from Bolivia); Hamel-Leigue et al. 2009: 62 (distribution of records from Bolivia); Vaz-de-Mello et al. 2011: 24 (characters in key); Carvajal et al. 2011: 140 (diagnosis), 322 (list of species from Ecuador); Solís and Kohlmann 2012: 7 (list of species from Costa Rica); Krajcik 2012: 191 (complete list of species); Boilly and Vaz-de-Mello 2013: 107 (characters in key); Figueroa et al. 2014: 130 (distributional records from Peru); Chamorro et al. 2018: 75 (characters in key), 97 (list of species from Ecuador).

*Sternaspis* Hope, 1837: 52 (original description. Type species: *Scarabaeusfestivus* Linnaeus, 1767); Agassiz 1846: 1018 (catalog); Lacordaire 1856: 100 (synonym of *Oxysternon* Castelnau, 1840); Gillet 1911a: 87 (cited as synonym of *Oxysternon* Castelnau, 1840); Lucas 1920: 612 (cited as synonym of *Oxysternon* Castelnau, 1840); d’Olsoufieff 1924: 156 (cited as *Sternaspsis* synonym of *Oxysternon* Castelnau, 1840); Pessôa and Lane 1941: 486 (cited as synonym of *Oxysternon* Castelnau, 1840); Blackwelder 1944: 210 (cited as synonym of *Oxysternon* Castelnau, 1840); Edmonds and Zídek 2004: 3 (synonym of *Oxysternon* Laporte, 1840; junior homonym of *Sternaspis* Otto, 1821 [Annelida: Polychaeta]); Vitolo 2004: 287 (cited as synonym of *Oxysternon* Castlenau, 1840); Solís and Kohlmann 2012: 7 (cited as synonym of *Oxysternon* Laporte, 1840); Figueroa et al. 2014: 130 (cited as synonym of *Oxysternon* Laporte, 1840).

*Strombodes* Gistel, 1857: 602 (original description. Type species: *Scarabaeusfestivus* Linnaeus, 1767 original designation); Martínez and Pereira 1967: 69 (synonym of *Oxysternon* Castelnau, 1840, comment); Edmonds and Zídek 2004: 3 (synonym of *Oxysternon* Laporte, 1840); Vitolo 2004: 287 (synonym of *Oxysternon* Laporte, 1840); Solís and Kohlmann 2012: 7 (synonym of *Oxysternon* Laporte, 1840); Figueroa et al. 2014: 130 (cited as synonym of *Oxysternon* Laporte, 1840).

##### Subgenus Oxysternon (Mioxysternon) Edmonds, 1972

Oxysternon (Mioxysternon) Edmonds, 1972: 836 (characters in key); 838 (original description. Type species: *Oxysternonspiniferum* Laporte, 1840, original designation); Halffter and Edmonds 1982: 136 (catalog, distribution); Vaz-de-Mello 2000: 194 (list of species from Brazil); Arnaud 2002a: 62 (characters in key), 64 (diagnosis); Edmonds and Zídek 2004: 7 (characters in key), 34 (diagnosis); Vitolo 2004: 289 (diagnosis, cited as *Oxysternon* s. str.); Hamel-Leigue et al. 2009: 64 (distribution of records from Bolivia); Vaz-de-Mello et al. 2011: 24 (characters in key); Krajcik 2012: 191 (cited as subgenus of *Oxysternon* Laporte de Castlenau 1840); Figueroa et al. 2014: 130 (distributional records from Peru); Boilly and Vaz-de-Mello 2013: 107 (characters in key); 75 (characters in key), 97 (list of species from Ecuador).

Oxysternon (Pteroxysternon) Arnaud, 2002: 63 (original description. Type species: *Oxysternonpteroderum* Nevinson, 1892); Edmonds and Zídek 2004: 34 (cited as synonym of Oxysternon (Mioxysternon) Edmonds, 1972).

###### Oxysternon (Mioxysternon) spiniferum

Taxon classificationAnimaliaColeopteraScarabaeidae

Laporte, 1840

Plate 45A

Oxysternon spiniferum Laporte, 1840: 83 (original description. Type locality: Cayenne).
Oxysternon (Mioxysternon) spiniferum : Arnaud 2002a: 62 (characters in key), 65 (diagnosis); Edmonds and Zídek 2004: 10 (characters in key), 35 (diagnosis); Vitolo 2004: 289 (diagnosis); Figueroa et al. 2014: 130 (distribution of records for Peru); Boilly et al. 2016: 89 (characters in key); 92 (figures 19a, 19b and 19c); 95 (cited for Guyana); Chamorro et al. 2018: 97 (cited for Ecuador).
Oxysternon (Mioxysternon) spiniferumspiniferum : Boilly and Vaz-de-Mello 2013: 105 (figure 19).
Oxysternon spiniferum : Hamel-Leigue et al. 2009: 50 (distribution of records for Bolivia); Krajcik 2012: 191 (cited as subgenus of Oxysternon Laporte de Castlenau 1840); Ratcliffe et al. 2015: 197 (cited for Peru).
Oxysternon curvispinum d’Olsoufieff, 1924: 119 (original description. Type locality: Equateur [= Ecuador], Loja).
Oxysternon curvispinum : Pessôa and Lane 1941: 486 (characters in key); Blackwelder 1944: 210 (list of species from Latin America); Vulcano and Pereira 1967: 570 (characters in key); Arnaud 1982a: 117 (catalog of the types of the MNHN); Hamel-Leigue et al. 2009: 64 (cited as synonym of O.spiniferumcurvispinum, Arnaud 2002); Figueroa et al. 2014: 130 (cited as synonym of Oxysternonspiniferumcurvispinum, Arnaud 2002).
Oxysternon spiniferum var. curvispinum : Arnaud 2002a: 65 (diagnosis); Edmonds and Zídek 2004: 35 (synonym of Oxysternonspiniferum Laporte, 1840); Carvajal et al. 2011: 322–323 (cited for Ecuador).

####### Type specimens.

*Oxysternonspiniferum* Laporte, 1840. The neotype (♂) is deposited at the MNHN (ex coll. R Oberthur, ex coll. van Lansberge). Locality: Guyana, examined.

**Neotype** (♂): “Guyana [hw]”, “Museum Paris / ex coll. / R. Oberthur [p, blak margin, green label]”, “Ex- Musæo / VAN LANSBERGE [p, blak margin, white label]”, “Oxysternon / spiniferum / Cast / P.ARNAUD Designation 00 / NEOTYPE ♂ [p and hw, red margin, white label]”.

*Oxysternoncurvispinum* d’Olsoufieff, 1924. The Lectotype is deposited at the MNHN (ex coll. R. Oberthur). Locality: Equateur [= Ecuador], Loja (examined).

**Lectotype** (♂): “Equateur / Loja / Abbé Gaujon [p, black margin]”, “Museum Paris / ex coll. / R. Oberthur [p, green label]”, “Oxysternon / curvispinum sp. nov [p, black margin]”, “LECTOTYPE [p, red label]”, “O. curvispinum / Ols. / Lectotyope ♂ / P. Arnaud DET 1981 [p and hw]”.

####### Distribution.

Brazil, Colombia, Ecuador, French Guiana, and Peru.

####### Records examined.

LOJA: without specific locality (1 specimen MNHN). ORELLANA: Estación Científica Yasuní, Parque Nacional Yasuní (5 specimens MQCAZ); Estación de Biodiversidad Tiputini USFQ, 220 m, Parque Nacional Yasuní (1 specimen MUTPL). PASTAZA: Bosque Protector Oglán Alto, 660 m (1 specimen CEMT; 3 specimens MUTPL). SUCUMBÍOS: Cascales, 400 m, Pozo Ruby 1 (1 specimen MUTPL); La Selva Bio Station 175 km E.S.E del Coca (1 specimen MQCAZ). ZAMORA CHINCHIPE: Tundayme campamento Mirador vivero, 820 m (1 specimen MUTPL); Tundayme campamento Mirador road to Polvorín, 1200 m (1 specimen MUTPL); Zurmi, Comunidad La Wants, 1010 m (2 specimens MUTPL).

####### Literature records.

LOJA: without specific locality (Arnaud 2002a: 65). NAPO: Tena (Edmonds and Zídek 2004: 35); Baeza (Edmonds and Zídek 2004: 35). NAPO [= ORELLANA]: Estación Científica Yasuní (Edmonds and Zídek 2004: 35); Yuturi Lodge, Río Napo, 270 m (Edmonds and Zídek 2004: 35). NAPO [= SUCUMBÍOS]: Limoncocha, 250 m (Edmonds and Zídek 2004: 35). ZAMORA CHINCHIPE: Punguinza, 710 m (Edmonds and Zídek 2004: 35).

####### Temporal data.

Collected in January, March, April, May, June, July, August, September, October, November, and December.

####### Remarks.

Inhabits the lowland evergreen forests and evergreen foothill forests of the Amazon region from 200–1200 m a.s.l. Collected with flight interception traps and pitfall traps baited with human feces.

##### Subgenus Oxysternon (Oxysternon) Laporte, 1840

Oxysternon (Oxysternon) s. str. Laporte, 1840: 82 (original description. Type species: *Scarabeusfestivus* Linnaeus, 1767); Edmonds 1972: 836 (characters in key); 838 (redescription); Halffter and Edmonds 1982: 136 (catalog, distribution); Vaz-de-Mello 2000: 194 (list of species from Brazil); Arnaud 2002a: 62 (characters in key), 66 (diagnosis); Edmonds and Zídek 2004: 7 (characters in key), 10 (diagnosis); Vitolo 2004: 287 (diagnosis); Hamel-Leigue et al. 2009: 62 (distribution of records from Bolivia); Vaz-de-Mello et al. 2011: 24 (characters in key); Boilly and Vaz-de-Mello 2013: 107 (characters in key); Figueroa et al. 2014: 130 (distribution of records from Peru); Chamorro et al. 2018: 75 (characters in key), 97 (list of species from Ecuador).

###### Oxysternon (Oxysternon) conspicillatum

Taxon classificationAnimaliaColeopteraScarabaeidae

(Weber, 1801)

Plate 45B

Copris conspicillata Weber, 1801: 36 (original description. Type locality: Brafilia [= Brazil]).
Copris conspicillatus : Fabricius 1801: 32 (redescription); Schönherr 1806: 35 (cited); Hope 1837: 51 (comment).
Phanaeus conspicillatus : Macleay 1819: 132 (trasnferred to the genus Phanaeus Macleay, 1819); Erichson 1847: 107 (redescription); Gemminger and Harold 1869: 1017 (list, distribution); Harold 1880a: 28 (distribution for Nueva Granada [= Colombia]).
Oxysternon conspicillatum : Nevinson 1892: 8 (transferred to the genus Oxysternon Weber, 1801); Gillet 1911a: 87 (complete list of species); d’Olsoufieff 1924: 47 (characters in key), 113 (redescription, distribution); Balthasar 1941: 351 (cited for Peru); Pessôa and Lane 1941: 486 (characters in key), 487 (comment); Blackwelder 1944: 210 (list of species from Latin America); Contreras 1951: 222 (cited for Colombia); Balthasar 1951: 337 (cited for Peru); Guêrin 1953: 261 (diagnosis); Vulcano and Pereira 1967: 568 (characters in key); Howden and Young 1981: 146 (characters in key, redescription); Vitolo 2000: 599 (characters in key); Medina et al. 2001: 140 (cited for Colombia); Ratcliffe 2002: 16 (cited for Panama); Krajcik 2012: 191 (complete list of species); Ratcliffe et al. 2015: 197 (cited for Peru).
Oxysternon (Oxysternon) conspicillatumvar.conspicillatum : Vaz-de-Mello 2000: 194 (cited for Brazil); Arnaud 2002a: 68 (diagnosis); Hamel-Leigue et al. 2006: 17 (list of species from Bolivia); Carvajal et al. 2011: 322–323 (cited for Ecuador).
Oxysternon (Oxysternon) conspicillatum : Arnaud 2002a: 67 (diagnosis); Edmonds and Zídek 2004: 10 (characters in key), 18 (diagnosis); Vitolo 2004: 287 (diagnosis); Hamel-Leigue et al. 2009: 62 (distributional records from Bolivia); Vaz-de-Mello et al. 2011: 64 (figure 137); Figueroa et al. 2014: 131 (figure 11), 132 (distributional records from Peru); Chamorro et al. 2018: 83 (figure 6H), 84 (figure 7A), 97 (cited for Ecuador).

####### Type specimens.

*Coprisconspicillata* Weber, 1801. Type material not examined.

####### Distribution.

Bolivia, Brazil, Colombia, Ecuador, Panama, Peru, and Venezuela.

####### Records examined.

CARCHI: Tobar Donoso 300 m (5 specimens MECN). COTOPAXI: Chugchilán, 2600 m (2 specimens MQCAZ). EL ORO: San Roque (1 specimen MQCAZ); Salvias, Río San José, 1200 m (1 specimen MQCAZ). ESMERALDAS: 11 km SE San Lorenzo, 5 m, Estación Forestal La Chiquita (8 specimens MQCAZ); Charco Vicente (7 specimens MGO-UC; 21 specimens MQCAZ; 11 specimens MECN); Colón del Onzole (16 specimens MQCAZ; 8 specimens MECN); Majua (1 specimen MGO-UC; 14 specimens MQCAZ; 19 specimens MECN); Pajonal (15 specimens MQCAZ; 12 specimens MECN); Palma Real (10 specimens MQCAZ; 7 specimens MECN); Playa de Oro, La Tabla (5 specimens MGO-UC; 33 specimens MQCAZ; 23 specimens MECN); Playa de Oro, 150 m (2 specimen CEMT; 1 specimen MUTPL; 45 specimens MQCAZ; 8 specimens MECN); Playa de Oro, Playa Rica (4 specimens MGO-UC; 8 specimens MQCAZ; 11 specimens MECN); Playa de Oro, Padre Santo (10 specimens MGO-UC; 36 specimens MQCAZ; 19 specimens MECN); Playa de Oro, Pistolas (2 specimens MQCAZ); Playa de Oro, Pote (2 specimens CEMT; 1 specimen MGO-UC; 18 specimens MQCAZ; 8 specimens MECN); Playa de Oro, Río Santiago, 200 m (1 specimen CEMT; 11 specimens MQCAZ; 5 specimens MECN); Salto del Bravo (9 specimens MQCAZ; 5 specimens MECN); Tsejpi (19 specimens MGO-UC; 22 specimens MQCAZ; 12 specimens MECN); Tsejpi río Zapallo (3 specimens MQCAZ). IMBABURA: Lita, 680 m (7 specimens MQCAZ; 3 specimens MECN). LOJA: Cariamanga (1 specimen MQCAZ); Catamayo (1 specimen MQCAZ). LOS RÍOS: Estación Biológica Río Palenque, 220 m (33 specimens MQCAZ). MANABÍ: sector El Mono, 245 m, Reserva Ecológica Mache Chindul (1 specimen MGO-UC). MORONA SANTIAGO: Comunidad Ángel Rouby, 1300–1700 m, Cordillera del Kutukú (1 specimen MQCAZ; 2 specimens MECN); Comunidad Unsuants, 700–1100 m, Cordillera del Kutukú (5 specimens MQCAZ; 3 specimens MECN); Macas (9 specimens MQCAZ); San Antonio, Limon Indazo, Centro Shuar Wuarints; San Pedro de Apondio, 1600 m (1 specimen MQCAZ); Wisui road to Taisha, 650 m (1 specimen MUTPL). NAPO: Archidona (3 specimens MCQAZ); Comunidad Rumiñahui, sector Kuriurko, 1070 m (1 specimen MUTPL); Pacto Sumaco, 1620 m (1 specimen MUTPL); Parahuacu (2 specimens MECN); Tena (15 specimen MQCAZ). ORELLANA: Bloque 31, Parque Nacional Yasuní, 200 m (3 specimens MECN); Comunidad Kiwcha Chiruisla Station, 180–250 m (24 specimens MQCAZ); Dayuma Campo Hormiguero, plataforma Hormiguero, 320 m (4 specimens MUTPL); Dayuma plataforma Ungurahua, 300 m (1 specimen MUTPL); El Dorado plataforma Guarango, 300 m (1 specimen MUTPL); Eden Yuturí, 225 m, Bloque 15 (1 specimen MUTPL); Rodrigo Borja IAMOE (1 specimen CEMT; 15 specimens MQCAZ); SCYASUNI [= Estación Científica Yasuní PUCE, Parque Nacional Yasuní], 250 m (2 specimens CEMT; 54 specimens MQCAZ); Estación de Biodiversidad Tiputini USFQ, 285 m, Río Tiputini, Parque Nacional Yasuní (1 specimen MUTPL; 2 specimens MGO-UC); Ines Arango Pre-Cooperativa Andina, 300 m (1 specimen MUTPL); Loreto (1 specimen MQCAZ); Parque Nacional Yasuní, 1220 m (1 specimen CEMT; 9 specimens MQCAZ); San Sebastian del Coca, Comuna Guataraco Campo Pata, 345 m (1 specimen MUTPL); Yasuní, 250 m (4 specimens CEMT; 3 specimens MQCAZ); Yasuní (1 specimen MGO-UC). PASTAZA: Bosque Protector Oglán Alto, 660–950 m (1 specimen MUTPL); Llandia 17 km N del Puyo, 1000 m (4 specimens MQCAZ). PICHINCHA: Chespi, 1140 m, Hacienda El Rosario (1 specimen MGO-UC); Estación Biológica La Hesperia (2 specimens MUTPL); Guayabilla Río Guayllabamba, 520 m, Manduriacus (1 specimen MGO-UC); Llurimaguas Río Guayllabamba, 290 m, Pedro Vicente Maldonado (1 specimen MUTPL); Mangaloma, 720 m, San Miguel de los Bancos (3 specimens MUTPL); Nanegal (5 specimens MQCAZ); Nono (1 specimen MQCAZ); San Roque Río Guayllabamba, 580 m, Pedro Vicente Maldonado (1 specimen MUTPL; 1 specimen MGO-UC). SANTO DOMINGO DE LOS TSÁCHILAS: Alluriquín, 800 m (5 specimens MQCAZ); Río Toachi (11 specimens MQCAZ). SUCUMBÍOS: Aucayacu Río El Eno, 275 m, 16 km de Lago Agrio (1 specimen MGO-UC); Cuyabeno (12 specimens MQCAZ); Laguna Grande Cuyabeno, 250 m (1 specimen CEMT; 19 specimens MQCAZ); Nueva Loja plataforma Iguana, 310 m (1 specimen MUTPL); Pacayacu Campo Libertador, 260 m (1 specimen MUTPL); Paña Cocha Sendero Playas del Cuyabeno, 260 m (1 specimen MGO-UC); Sansahuari, 260 m, Pozo Singüe (1 specimen MUTPL); Tarapoa Campo Marian, plataforma Fanny 5, 260 m (1 specimen MUTPL). ZAMORA-CHINCHIPE: Bombuscaro, Parque Nacional Podocarpus, 1100 m (5 specimens MECN); San Andres, 1850 m (2 specimens CEMT); Valladolid, 1645 m (1 specimen MQCAZ); Zamora (5 specimens MQCAZ); Zurmi Comunidad Miazi, 1380 m (1 specimen MEPN, 2 specimens MUTPL); Zurmi Comunidad La Wants, 1010 m (1 specimen MEPN; 3 specimens MUTPL); Zurmi Las Orquideas Río Nangaritza, 870 m (1 specimen MUTPL).

####### Literature records.

COTOPAXI: Las Palmas (Edmonds and Zídek 2004: 20); Chugchilán, 2600 m (Edmonds and Zídek 2004: 20). ESMERALDAS: Estación Biológica Bilsa, 500 m (Edmonds and Zídek 2004: 20); 11 km SE San Lorenzo, Estación Forestal La Chiquita, 5 m (Edmonds and Zídek 2004: 20); Palma Real (Edmonds and Zídek 2004: 20); Pajonal (Edmonds and Zídek 2004: 20); Charco Vicente (Edmonds and Zídek 2004: 20); Salto del Bravo (Edmonds and Zídek 2004: 20); Majua (Edmonds and Zídek 2004: 20). GUYAS: Guayaquil (Edmonds and Zídek 2004: 20). LOJA: Catamayo (Edmonds and Zídek 2004: 20). LOS RÍOS: Quevedo (Edmonds and Zídek 2004: 20); 40 km N Quevedo, Estación Biológica Río Palenque, 150–220 m (Edmonds and Zídek 2004: 20). MANABÍ: Palmar, 200 m (Edmonds and Zídek 2004: 20). MORONA SANTIAGO: Macas (Edmonds and Zídek 2004: 20). NAPO: Río Jatunyacu, 700 m (Edmonds and Zídek 2004: 20); Archidona (Edmonds and Zídek 2004: 20); Zatzayacu (Edmonds and Zídek 2004: 20); Cosanga, 2150 m (Edmonds and Zídek 2004: 20). NAPO [= ORELLANA]: Loreto (Edmonds and Zídek 2004: 20); Estación CIentífica Yasuní (Edmonds and Zídek 2004: 20). NAPO [= SUCUMBÍOS]: Limoncocha, 260 m (Edmonds and Zídek 2004: 20). PASTAZA: Puyo, 1000 m (Edmonds and Zídek 2004: 20); 25 km N Puyo (Edmonds and Zídek 2004: 20); Cusuimi, Río Cusuimi 150 km SE Puyo (Edmonds and Zídek 2004: 20); Ashuara on Río Macuma, 10 km from Río Morona, 300 m (Edmonds and Zídek 2004: 20); Santa Clara (Edmonds and Zídek 2004: 20); Loracachi, 220 m (Edmonds and Zídek 2004: 20); Kapawi, 350 m (Edmonds and Zídek 2004: 20); Bosque Villano (Edmonds and Zídek 2004: 20). PICHINCHA: Nono (Edmonds and Zídek 2004: 20); Nanegal, 1280 m (Edmonds and Zídek 2004: 20). PICHINCHA [= SANTO DOMINGO DE LOS TSÁCHILAS]: km 27 old Santo Domingo Road, 3200 m (Edmonds and Zídek 2004: 20); Alluriguin [= Alluriquín], 800 m (Edmonds and Zídek 2004: 20). Tinalandia, 12 km E Santo Domingo de los Colorados, 750 m (Edmonds and Zídek 2004: 21). SUCUMBÍOS: Cuyabeno (Edmonds and Zídek 2004: 21). ZAMORA CHINCHIPE: Zamora (Edmonds and Zídek 2004: 21); Valladolid, 1645 m (Edmonds and Zídek 2004: 21).

####### Temporal data.

Collected every month of the year.

####### Remarks.

Inhabits coastal lowland evergreen, coastal lowland semi-deciduous, and coastal foothill evergreen forests from 5–1140 m a.s.l. In the Andean region, it was recorded in the montane cloud and high montane evergreen forests from 1800–2600 m a.s.l. In the Amazon, it was recorded from the lowland evergreen, varzea, foothill evergreen, and lower montane evergreen forests from 1380–1700 m a.s.l. Collected manually using flight interception traps and pitfall traps baited with carrion and human feces.

###### Oxysternon (Oxysternon) silenussmaragdinum

Taxon classificationAnimaliaColeopteraScarabaeidae

d’Olsoufieff, 1924

Plate 45C

Oxysternon smaragdinum d’Olsoufieff, 1924: 117 (original description. Type locality: Loja, Amazones [= Amazonas]: Pebas).
Oxysternon smaragdinum : Balthasar 1941: 351 (cited for Peru); Blackwelder 1944: 210 (list of species from Latin America); Balthasar 1951: 337 (cited for Peru); Vulcano and Pereira 1967: 569 (characters in key); Arnaud 1982a: 117 (catalog of the types of the MNHN); Vaz-de-Mello 2000: 194 (cited for Brazil); Vitolo 2000: 599 (characters in key); Medina et al. 2001: 140 (cited for Colombia); Vitolo 2004: 288 (diagnosis); Hamel-Leigue et al. 2009: 63 (cited as synonym of Oxysternonsilenus Laporte de Castelnau, 1840); Solís and Kohlmann 2012: 7 (cited as synonym of Oxysternonsilenus Laporte, 1840); Figueroa et al. 2014: 130 (cited as synonym of Oxysternonsilenus Laporte, 1840).
Oxysternon (Oxysternon) silenussmaragdinum : Arnaud 2002a: 74 (diagnosis); Edmonds and Zídek 2004: 29 (synonym of Oxysternon (Oxysternon) silenus Laporte, 1840); Arnaud 2004: 10 (revalidated status); Carvajal et al. 2011: 322–323 (cited for Ecuador); Ratcliffe et al. 2015: 197 (cited for Peru); Chamorro et al. 2018: 83 (figures 6F and 6G), 97 (cited for Ecuador).

####### Type specimens.

*Oxysternonsmaragdinum* d’Olsoufieff, 1924. The lectotype (♂) is deposited in the MNHN (see Arnaud 1982a: 117). Locality: Équateur [= Ecuador], Loja, not examined.

####### Distribution.

Colombia, Ecuador, Peru, and Venezuela.

####### Records examined.

CARCHI: Tobar Donoso, 300 m (5 specimens MECN). ESMERALDAS: Colón del Onzole (7 specimens MECN; 11 specimens MQCAZ); Charco Vicente (8 specimens MECN; 3 specimens MQCAZ); Estación Biológica Bilsa, 500 m (3 specimens MEPN); Majua (4 specimens MECN; 6 specimens MQCAZ); Palma Real (7 specimens MECN; 11 specimens MQCAZ); Playa de Oro (1 specimen CEMT); Salto del Bravo (5 specimen MECN); Tsejpi Charco Grande (2 specimens CEMT). IMBABURA: El Chontal, El Cauchero, 900 m (1 specimen MUTPL); Lita, 680 m (1 specimen MECN). MORONA SANTIAGO: km 8 road Mendez-Paute, 1250 m (1 specimen CEMT; 4 specimens MQCAZ); San Pedro de Apondio, 1600 m (1 specimen MECN). NAPO: Bosque Protector la Cascada Río Coca, 640 m (1 specimen MUTPL); southeast of Puerto Napo, 610 m, Pungarayacu (1 specimen MQCAZ). ORELLANA: Cononaco, Bloque 16 YPF Parque Nacional Yasuní (1 specimen MUTPL); Dayuma Campo Hormiguero, plataforma Hormiguero, 320 m (1 specimen MUTPL); Dayuma Campo Pindo, 305 m, Pindo Este 1 (1 specimen MUTPL); Dayuma plataforma Ungurahua, 300 m (1 specimen MUTPL); Eden Yuturí, 225 m, Bloque 15 (1 specimen MUTPL); El Dorado plataforma Guarango, 300 m (1 specimen MUTPL); Estación Científica Yasuní PUCE, 215 m, Parque Nacional Yasuní (21 specimens MQCAZ); Estación de Biodiversidad Tiputini USFQ, 280 m, Parque Nacional Yasuní (1 specimen MUTPL); Payamino Research Stn, 300 m (1 specimen CEMT); Rodrigo Borja IAMOE (2 specimens CEMT; 7 specimens MQCAZ); San Sebastian del Coca, Comuna Guataraco, 345 m, Campo Pata (1 specimen MUTPL); San Sebastian del Coca, Comuna Shamanal, 345 m Campo Palo Azul (1 specimen MUTPL); San Pedro del Lago, plataforma Copal, 310 m (1 specimen MUTPL); Yasuní (1 specimen MUTPL). PASTAZA: Bosque Protector Oglán Alto, 555–950 m (1 specimen MUTPL). PICHINCHA: San Roque Río Guayllabamba, 580 m, Pedro Vicente Maldonado (1 specimen MUTPL). SUCUMBÍOS: 6 km de Dureno, 300 m, Precooperativa Los Vergeles (1 specimen MGO-UC); Cuyabeno Campo Hormiga (1 specimen CEMT); Pacayacu Campo Libertador, 260 m (1 specimen MUTPL); Tarapoa, Nuevo Manabí, 270 m (1 specimen MUTPL); Tipishca (2 specimens MECN). ZAMORA CHINCHIPE: km 4 Zumbí-Yantzaza, 900 m (1 specimen CEMT; 4 specimens MQCAZ); Zurmi, Comunidad La Wants, 1010 m (3 specimens MUTPL; 1 specimen MEPN); Zurmi Las Orquideas Río Nangaritza, 870 m (1 specimen MUTPL).

####### Literature records.

CARCHI: without specific locality (Arnaud 2002a: 74). ESMERALDAS: 11 km SE San Lorenzo, Estación Forestal La Chiquita 5 m (Edmonds and Zídek 2004: 31). LOJA: without specific locality (Arnaud 1982a: 117). LOS RÍOS: Quevedo (Edmonds and Zídek 2004: 31). MORONA SANTIAGO: Macas (Edmonds and Zídek 2004: 31); km 8 road Mendez-Paute, 1250 m. NAPO: 20 km E Puerto Napo, 450 m (Edmonds and Zídek 2004: 31); Tena, 400 m (Edmonds and Zídek 2004: 31); 20 km S Tena, 600 m (Edmonds and Zídek 2004: 31); 5 km W Tena, 500 m (Edmonds and Zídek 2004: 31); Jatun-Sacha, Biological Station, 450 m (Edmonds and Zídek 2004: 31); Misahuallí Jungle Lodge, 550 m (Edmonds and Zídek 2004: 31). NAPO [= ORELLANA]: Estación Científica Yasuní, 215 m (Edmonds and Zídek 2004: 31). NAPO [= SUCUMBÍOS]: Limoncocha, 250 m (Edmonds and Zídek 2004: 31). PASTAZA: Ashuara, Río Macuma 10 km hacia el Río Morona, 300 m (Edmonds and Zídek 2004: 31); 25 km NNE Puyo, 1000 m (Edmonds and Zídek 2004: 31); PICHINCHA [= SANTO DOMINGO DE LOS TSÁCHILAS]: Tinalandia, 680 m, 16 km E Santo Domingo de los Colorados (Edmonds and Zídek 2004: 31). SUCUMBÍOS: Cuyabeno, 270 m (Edmonds and Zídek 2004: 31). ZAMORA CHINCHIPE: Sabanilla (Edmonds and Zídek 2004: 31).

####### Temporal data.

Collected every month of the year.

####### Remarks.

Inhabits coastal lowland evergreen forests and coastal evergreen foothill forests from 5–680 m a.s.l. In the Amazon, it was recorded in the lowland evergreen forests, the foothill evergreen forests, and evergreen lower montane forests from 215–1700 m a.s.l. Collected manually and with pitfall traps baited with carrion and human feces.

#### Genus *Phanaeus* Macleay, 1819

*Phanaeus* Macleay, 1819: 124 (original description. Type species: *Scarabaeuscarnifex* Linnaeus, 1767 subsequent designation by d’Olsoufieff, 1924: 23).

*Phanaeus*: Brullé 1837: 302 (redescription); Agassiz 1846: 818 (catalog); Lacordaire 1856: 100 (redescription); Gemminger and Harold 1869: 1016 (catalog); Lacordaire and Chapuis 1876: 276 (catalog); Nevinson 1892: 1 (catalog, distribution); Gillet 1911a: 81 (catalog, distribution); Lucas 1920: 499 (catalog, distribution); Dawson 1922: 61 (characters in key); d’Olsoufieff 1924: 22 (characters in key); 63 (redescription), 140 (distribution); Pessôa 1934: 282 (description), 284 (biology); Pessôa and Lane 1941: 470 (characters in key); Blackwelder 1944: 209 (list of species from Latin America); Roze 1955: 45 (list of species from Venezuela); Martínez 1959: 97 (catalog of species, distribution); Halffter and Matthews 1966: 258 (catalog, distribution); Vulcano and Pereira 1967: 566 (characters in key); Edmonds 1972: 820 (characters in key), 826 (redescription); Howden and Young 1981: 12 (characters in key), 134 (redescription); Halffter and Edmonds 1982: 136 (catalog, distribution); Zunino 1985: 104 (comment); Edmonds 1994: 8 (revision); Medina and Lopera 2000: 303 (characters in key); Vitolo 2000: 595 (characters in key); Vaz-de-Mello 2000: 194 (list of species from Brazil); Medina et al. 2001: 140 (list of species from Colombia); Arnaud 2002a: 13 (characters in key); Ratcliffe 2002: 16 (list of species for Panama); Morón 2003: 60 (redescription); Vitolo 2004: 283 (diagnosis); Philips et al. 2004: 50 (comment); Hamel-Leigue et al. 2006: 17 (list of species from Bolivia); Hamel-Leigue et al. 2009: 64 (distributional records from Bolivia); Vaz-de-Mello et al. 2011: 25 (characters in key); Carvajal et al. 2011: 137 (diagnosis), 318 (cited for Ecuador); Solís and Kohlmann 2012: 7 (list of species from Costa Rica); Edmonds and Zídek 2012: 2 (revision); Krajcik 2012: 204 (complete list of species); Figueroa et al. 2014: 133 (distributional records from Peru); Chamorro et al. 2018: 75 (characters in key), 97–98 (list of species from Ecuador).

*Lonchophorus* Germar, 1824: 106 (original description. Type species: *Scarabaeuscarnifex* Linnaeus, 1767); Brullé 1837: 302 (synonym of *Phanaeus* Macleay, 1819); Agassiz 1846: 620 (catalog); Lacordaire 1856: 100 (synonym of *Phanaeus* Macleay, 1819); Gemminger and Harold 1869: 1016 (synonym of *Phanaeus* Macleay, 1819); Nevinson 1892: 1 (synonym of *Phanaeus* Macleay, 1819); Gillet 1911a: 81 (synonym of *Phanaeus* Macleay, 1819); Lucas 1920: 381 (synonym of *Phanaeus* Macleay, 1819); d’Olsoufieff 1924: 140 (synonym of *Phanaeus* Macleay, 1819); Blackwelder 1944: 209 (synonym of *Phanaeus* Macleay, 1819); Martínez 1959: 97 (synonym of *Phanaeus* Macleay, 1819); Edmonds 1972: 826 (synonym of *Phanaeus* Macleay, 1819); Ratcliffe 2002: 16 (synonym of *Phanaeus* Macleay, 1819); Edmonds 1994: 46 (cited as synonym of *Phanaeus* s. str. Macleay, 1819); Vitolo 2004: 286 (cited as synonym of *Phanaeus* s. str. Macleay, 1819); Solís and Kohlmann 2012: 7 (synonym of *Phanaeus* Macleay, 1819); Figueroa et al. 2014: 133 (synonym of *Phanaeus* Macleay, 1819).

*Onthurgus* Gistel, 1857: 602 (original description. Type species: *Scarabaeuscarnifex* Linnaeus, 1767); Edmonds 1972: 827 (synonym of *Phanaeus* Macleay, 1819); Ratcliffe 2002: 16 (synonym of *Phanaeus* Macleay, 1819); Edmonds 1994: 46 (cited as synonym of *Phanaeus* s. str. Macleay, 1819); Vitolo 2004: 286 (cited as synonym of *Phanaeus* s. str. Macleay, 1819); Solís and Kohlmann 2012: 7 (synonym of *Phanaeus* Macleay, 1819); Figueroa et al. 2014: 133 (synonym of *Phanaeus* Macleay, 1819).

*Palaeocopris* Pierce, 1946: 130 (original description. Type species: *Palaeocoprislabreae* Pierce, 1946); Ratcliffe 2002: 16 (synonym of *Phanaeus* Macleay, 1819); Edmonds 1994: 46 (cited as synonym of *Phanaeus* s. str. Macleay, 1819); Vitolo 2004: 286 (cited as synonym of *Phanaeus* s. str. Macleay, 1819); Solís and Kohlmann 2012: 7 (synonym of *Phanaeus* MacLeay, 1819).

##### Subgenus Phanaeus (Notiophanaeus) Edmonds, 1994

Phanaeus (Notiophanaeus) Edmonds, 1994: 18 (original description. Type species: *Scarabaeussplendidulus* Fabricius, 1781 original combination); Vaz-de-Mello 2000: 194 (list of species from Brazil); Arnaud 2002a: 78 (characters in key), 80 (diagnosis); Morón 2003: 65 (diagnosis); Vitolo 2004: 283 (diagnosis); Hamel-Leigue et al. 2009: 64 (distribution of records from Bolivia); Vaz-de-Mello et al. 2011: 25 (characters in key); Edmonds and Zídek 2012: 7 (characters in key); Krajcik 2012: 204 (cited as subgenus of *Phanaeus* Macleay, 1819); Figueroa et al. 2014: 133 (distribution of records from Peru); Chamorro et al. 2018: 75 (characters in key), 97–98 (list of species from Ecuador).

###### Phanaeus (Notiophanaeus) achilles

Taxon classificationAnimaliaColeopteraScarabaeidae

Boheman, 1858

Plate 45D

Phanaeus achilles Boheman, 1858: 42 (original description. Type locality: Insula Puna [= Isla Puna]).
Phanaeus achilles : Gemminger and Harold 1869: 1016 (catalog, distribution, written as Phanaeus Achiles Bohem); Nevinson 1892: 1 (catalog, distribution, written as Phanaeus Achiles Boheman); Gillet 1911a: 81 (catalog, distribution, written as Phanaeus Achiles Boh); d’Olsoufieff 1924: 40 (characters in key); 97 (diagnosis), 151 (distribution); Blackwelder 1944: 209 (list of species from Latin America); Vulcano and Pereira 1967: 574 (characters in key); Ratcliffe et al. 2015: 197 (cited for Peru).
Phanaeus (Notiophanaeus) achilles : Edmonds 1994: 28 (characters in key), 30 (redescription), 99 (distribution); Edmonds and Zídek 2012: 10 (characters in key); Figueroa et al. 2014: 136 (distributional records from Peru); Chamorro et al. 2018: 97 (cited for Ecuador).
Phanaeus (Notiophanaeus) achillesachiles : Arnaud 2002a: 88 (diagnosis); Streit 2008: 8 (distribution, photography); Carvajal et al. 2011: 320–321 (cited for Ecuador); Krajcik 2012: 204 (complete list of species).
Phanaeus Achilles lydiae Arnaud, 2000: 8 (original description); Arnaud 2002a: 88 (diagnosis, distribution); Streit 2008: 9 (distribution, photography); Carvajal et al. 2011: 320–321 (cited for Ecuador); Krajcik 2012: 204 (complete list of species).
Phanaeus foveolatus Harold, 1880b: 152 (original description); Nevinson 1892: 4 (catalog, distribution); Gillet 1911a: 81 (cited as synonym of Phanaeusachilles Boheman, 1858); Blackwelder 1944: 209 (synonym of Phanaeusachilles Boheman, 1858); Edmonds 1994: 30 (cited as synonym of Phanaeusachilles Boheman, 1858); Arnaud 2002a: 88 (cited as synonym of Phanaeusachilles Boheman, 1858); Figueroa et al. 2014: 136 (cited as synonym of Phanaeusachilles Boheman, 1858).

####### Type specimens.

*Phanaeusachilles* Boheman, 1858. The holotype (♂) is deposited at the NHRS (see Edmonds 1994: 30). Locality: Puna, not examined.

*Phanaeusfoveolatus* Harold, 1880. The holotype is deposited at the NMHU. Locality: Guayaquil, examined.

**Holotype** (♂): “Guayaquil. / Rusj [hw, green label]”, “foveolatus / Harold [hw, green label]”, “38678 [p]”, “1405 [p]”, “♂ HOLOTYPE [hw and p, red label]”.

*Phanaeusachilleslydiae* Arnaud, 2000. The holotype (♂) is deposited at the CPFA (see Arnaud 2000: 8). Locality: Guayas, Los Ceibos Guayaquil (not examined). One paratype is deposited in MQCAZ, examined.

**Paratype** (♂): “ECUADOR (GUA) / Cerecita / 02 / 87 / P. Arnaud leg [p]”, “Phanaeus achilles / lydiae / P. ARNAUD DET 96 / PARATYPE ♂ [p and hw, red margin]”.

####### Distribution.

Ecuador and Peru.

####### Records examined.

EL ORO: 3 km S de Arenillas, 50 m (6 specimens CEMT); Arenillas, 15 m (75 specimens CEMT). GUAYAS: Cerecita (1 specimen MQCAZ); Guayaquil (1 specimen NMHU; 2 specimens CFPL); Los Ceibos Guayaquil (4 specimenes CEMT); Puna [= Isla Puna] (2 specimens NHRS). LOJA: Catamayo, Alamala, 1100 m (16 specimens MUTPL). MANABÍ: El Aromo, La Fabril, 290 m (1 specimen CEMT; 8 specimens MUTPL); El Aromo, Pueblo, 370 m (3 specimens MUTPL); Machalilla, 30 m (1 specimen CEMT); San Juan de Manta, 160 m (3 specimens MUTPL). UNDETERMINED PROVINCE: without specific locality (1 specimen NHRS).

####### Literature records.

GUAYAS: 40 km SW Guayaquil, 50 m (Edmonds 1994: 99); 45 km W Guayaquil (Edmonds 1994: 99); Bucay (Edmonds 1994: 99); Env. Cerecita, Rte. Salinas (Arnaud 2000: 8); Guayaquil (Edmonds 1994: 99); Insula Puna [= Isla Puna] (Boheman 1858: 42; Edmonds 1994: 28); Los Ceibos, Guayaquil (Arnaud 2000: 8; Arnaud 2002a: 88); Posorja, 0 m (Arnaud 2000: 8; Edmonds 1994: 99). LOJA: Catamayo (Edmonds 1994: 99); E35 9.5 km S. Catamayo 1211 m (Streit 2008: 8); without specific locality (Arnaud 2002a: 88); without specific locality (Edmonds 1994: 99). MANABÍ: Montecristi (Streit 2008: 9).

####### Temporal data.

Collected in January, February, March, April, May, June, October, November, and December.

####### Remarks.

Inhabits coastal lowland semi-deciduous forests from 5–370 m a.s.l. In the Andean region, it was recorded in the matorral dry montane forests from 1100–1210 m a.s.l. Collected manually and with pitfall traps baited with human feces.

###### Phanaeus (Notiophanaeus) arletteae

Taxon classificationAnimaliaColeopteraScarabaeidae

Arnaud, 2018

Plate 46A

Phanaeus (Notiophanaeus) arletteae Arnaud, 2018: 4 (original description. Type locality: Point kilométrique 18, Route de Balzar à Quevedo [= km 18, road Balzar to Quevedo], 400 m, Guayas, Ecuador), 5 (figure 2a-c).
Phanaeus (Notiophanaeus) arletteae : Chamorro et al. 2018: 97 (cited for Ecuador).
Phanaeus arletteae : Kohlmann et al. 2018: 83 (characters in key, cited for Ecuador).

####### Type specimens.

Phanaeus (Notiophanaeus) arletteae Arnaud, 2018. The holotype (♂) is deposited at the CPFA (see Arnaud 2018: 4). Locality: ECUADOR, GUAYAS, 400 m, Point km 18 Rte Balzar-Quevedo, not examined.

####### Distribution.

Colombia and Ecuador

####### Records examined.

LOJA: Zapotillo Cabeza de Toro, 510 m (1 specimen CEMT).

####### Literature records.

CAÑAR: Rte La Troncal-Cuenca, Javin, 1400 m (Arnaud, 2018: 4). GUAYAS: Balzar mountain (d’Olsoufieff, 1924: 92; Arnaud, 2002a: 99; Arnaud: 2018: 4), Point km 18 Rte Balzar-Quevedo, 400 m (Arnaud: 2018: 4). LOS RÍOS: without specific locality (Arnaud, 2002a: 99).

####### Temporal data.

Collected in March and May.

####### Remarks.

Inhabits coastal lowland evergreen forests, coastal lowland semi-deciduous forests, and coastal evergreen foothill forests from 400–1400 m a.s.l. Collected with pitfall traps baited with human feces.

###### Phanaeus (Notiophanaeus) bispinus

Taxon classificationAnimaliaColeopteraScarabaeidae

Bates, 1868

Plate 46B

Phanaeus bispinus Bates, 1868: 89 (original description. Type locality: Pastaza, Canelos).
Phanaeus bispinus : Gemminger and Harold 1869: 1017 (catalog); Nevinson 1892: 2 (catalog, distribution); Gillet 1911a: 81 (catalog, distribution); d’Olsoufieff 1924: 34 (characters in key); 84 (redescription), 147 (distribution); Blackwelder 1944: 209 (list of species from Latin America); Vulcano and Pereira 1967: 574 (characters in key); Arnaud 1982a: 114 (list of the types of the MNHN); Vitolo 2000: 597 (characters in key); Medina et al. 2001: 140 (cited for Colombia); Hamel-Leigue et al. 2006: 17 (list of species for Bolivia); Krajcik 2012: 204 (complete list of species); Ratcliffe et al. 2015: 197 (cited for Peru).
Phanaeus (Notiophanaeus) bispinus : Edmonds 1994: 33 (characters in key), 35 (redescription), 100 (distribution); Vaz-de-Mello 2000: 194 (cited for Brazil); Arnaud 2002a: 89 (diagnosis); Vitolo 2004: 283 (diagnosis); Hamel-Leigue et al. 2009: 66 (distributional records from Bolivia); Carvajal et al. 2011: 320–321 (cited for Ecuador); Edmonds and Zídek 2012: 9 (characters in key); Figueroa et al. 2014: 133 (distributional records from Peru); Boilly et al. 2016: 90 (characters in key); 93 (figures 19a, 19b and 19c); 96 (cited for Guyana); Chamorro et al. 2018: 97 (cited for Ecuador).
Phanaeus digitalis d’Olsoufieff, 1924: 34 (original description); Blackwelder 1944: 209 (list of species from Latin America); Pereira and Martínez 1956b: 237 (synonym of Phanaeusbispinus Bates, 1868); Frey 1963: 559 (cited as synonym of Phanaeusbispinus Bates, 1868); Edmonds 1994: 35 (synonym of Phanaeusbispinus Bates, 1868); Arnaud 2002a: 89 (synonym of Phanaeusbispinus Bates, 1868); Vitolo 2004: 283 (synonym of Phanaeusbispinus Bates, 1868); Hamel-Leigue et al. 2009: 66 (synonym of Phanaeusbispinus Bates, 1868); Edmonds and Zídek 2012: 5 (junior synonym of Phanaeusbispinus Bates, 1868); Figueroa et al. 2014: 133 (synonym of Phanaeusbispinus Bates, 1868).

####### Type specimens.

*Phanaeusbispinus* Bates, 1868. The holotype (♂) is deposited at the MNHN (see Edmonds 1994: 35). Locality: Canelos, Ecuador, not examined.

*Phanaeusdigitalis* d’Olsoufieff, 1924. The holotype (♀) is deposited at the MNHN (see Edmonds 1994: 35). Locality: Guyana, not examined.

####### Distribution.

Bolivia, Brazil, Colombia, Ecuador, French Guiana, Guyana, Peru, and Venezuela.

####### Records examined.

NAPO: Puerto Napo, 480 m (2 specimens MECN). ORELLANA: Añangu (1 specimen MQCAZ); Dayuma Campo Palanda, plataforma Primavera 1, 235 m (6 specimens MUTPL); Dayuma Campo Pindo, plataforma Pindo Este 1, 305 m (1 specimen MUTPL); Onkone Gare, 220 m, Parque Nacional Yasuní (1 specimen MUTPL); El Coca, plataforma Oso B (1 specimen MUTPL); Yasuni Puce BS, 200 m, Río Tiputini (3 specimens MQCAZ). SUCUMBÍOS: Garzacocha (1 specimen MQCAZ); Tarapoa, 260 m, Fanny 5 (1 specimen MUTPL); Tarapoa, Nuevo Manabí, 270 m (1 specimen MUTPL).

####### Literature records.

NAPO: Tena, 400 m (Edmonds 1994: 100); without specific locality (Arnaud 2002a: 89). PASTAZA: Canelos (Bates 1868: 89; Edmonds 1994: 100).

####### Temporal data.

Collected in February, May, August, September, October, and November.

####### Remarks.

Inhabits the lowland evergreen forests of the Amazon region from 220–400 m a.s.l. Collected manually and with pitfall traps baited with human feces.

###### Phanaeus (Notiophanaeus) cambeforti

Taxon classificationAnimaliaColeopteraScarabaeidae

Arnaud, 1982

Plate 46C

Phanaeus cambeforti Arnaud, 1982b: 122 (original description. Type locality: Saül, French Guiana).
Phanaeus cambeforti : Vitolo 2000: 597 (characters in key); Medina et al. 2001: 140 (cited for Colombia); Carvajal et al. 2011: 320–321 (cited for Ecuador); Krajcik 2012: 204 (complete list of species); Ratcliffe et al. 2015: 197 (cited for Peru).
Phanaeus (Notiophanaeus) cambeforti : Edmonds 1994: 28 (characters in key), 31 (redescription); Vaz-de-Mello 2000: 194 (cited for Brazil); Arnaud 2002a: 86 (diagnosis); Vitolo 2004: 285 (diagnosis); Hamel-Leigue et al. 2009: 65 (distributional records from Bolivia); Edmonds and Zídek 2012: 10 (characters in key); Figueroa et al. 2014: 134 (distributional records from Peru); Boilly et al. 2016: 90 (characters in key); 93 (figures 20a, 20b and 20c); 96 (cited for Guyana); Chamorro et al. 2018: 98 (cited for Ecuador).

####### Type specimens.

*Phanaeuscambeforti* Arnaud, 1982. The holotype (♂) is deposited at the MNHN (see Edmonds 1994: 31). Locality: French Guiana, Saul, not examined.

####### Distribution.

Bolivia, Brazil, Colombia, Ecuador, French Guiana, Guyana, and Peru.

####### Records examined.

ORELLANA: Daimi 1; Dayuma Campo Hormiguero, plataforma Hormiguero, 320 m (1 specimen MUTPL); Dayuma Campo Palanda, Estación Palanda 5, 320 m (2 specimens MUTPL); Dayuma plataforma Ungurahua, 300 m (1 specimen MUTPL); Estación de Biodiversidad Tiputini USFQ, 280 m, Parque Nacional Yasuní (3 specimens MUTPL); Puce Yasuní Biological Station, 250 m, Río Tiputini (7 specimens MQCAZ); Rodrigo Borja IAMOE (1 specimen CEMT); Ines Arango road Tiwino-río Shiripuno, 250 m (1 specimen MUTPL); Río Tiputini Yasuní Res. Stn. (3 specimens CEMT). PASTAZA: Bosque Protector Oglán Alto, 660 m (1 specimen MUTPL). SUCUMBÍOS: Cascales, 400 m, Pozo Ruby 1 (1 specimen MUTPL); Cuyabeno Lag. Grande (2 specimen MQCAZ); Tarapoa, 260 m, Fanny 5 (2 specimens MUTPL).

####### Literature records.

UNDETERMINED PROVINCE: without specific locality (Arnaud, 1982b: 123).

####### Temporal data.

Collected in January, March, April, May, July, August, and November.

####### Remarks.

Inhabits the lowland evergreen forests and evergreen foothill forests of the Amazon region from 250–660 m a.s.l. Collected manually, with flight interception traps and pitfall traps baited with carrion and human feces.

###### Phanaeus (Notiophanaeus) chalcomelas

Taxon classificationAnimaliaColeopteraScarabaeidae

(Perty, 1830)

Plate 46D

Onitis chalcomelas Perty, 1830: 40 (original description. Type locality: Habitat in mediterraneis Prov. S. Pauli et Minarum [= South America]).
Phanaeus chalcomelas : Harold 1859: 198 (transferred to the PhanaeusMacleay 1819, comment); Gemminger and Harold 1869: 1017 (catalog); Nevinson 1892: 2 (catalog, distribution); Gillet 1911a: 82 (catalog, distribution); d’Olsoufieff 1924: 40 (characters in key); 98 (redescription), 152 (distribution); Blackwelder 1944: 209 (list of species from Latin America); Vulcano and Pereira 1967: 574 (characters in key); Medina et al. 2001: 140 (cited for Colombia); Krajcik 2012: 204 (complete list of species); Ratcliffe et al. 2015: 197 (cited for Peru).
Phanaeus (Phanaeus) chalcomelas : Pessôa 1934: 305 (characters in key), 312 (redescription).
Phanaeus (Notiophanaeus) chalcomelas : Edmonds 1994: 27 (characters in key), 28 (redescription), 100 (distribution); Vitolo 2000: 597 (characters in key); Vaz-de-Mello 2000: 194 (cited for Brazil); Arnaud 2002a: 84 (diagnosis); Hamel-Leigue et al. 2009: 65 (distributional records from Bolivia); Edmonds and Zídek 2012: 10 (characters in key); Figueroa et al. 2014: 134 (distribution of records for Peru); Boilly et al. 2016: 90 (characters in key); 93 (figures 21a, 21b and 21c); 96 (cited for Guyana); Chamorro et al. 2018: 98 (cited for Ecuador).
Phanaeus (Notiophanaeus) chalcomelaschalcomelas : Arnaud 2002a: 85 (diagnosis); Vitolo 2004: 285 (diagnosis); Carvajal et al. 2011: 320–321 (cited for Ecuador).

####### Type specimens.

*Onitischalcomelas* Perty, 1830. The holotype (♀) is deposited at the ZSM (see Edmonds 1994: 29). Locality: Brasilia [=Brazil], not examined.

####### Distribution.

Bolivia, Brazil, Colombia, Ecuador, French Guiana, Guyana, and Peru.

####### Records examined.

MORONA SANTIAGO: Comunidad Ángel Rouby, 1300 m, Cordillera del Kutukú (1 specimen MECN); Comunidad Unsuants, 700 m, Cordillera del Kutukú (3 specimens MECN). NAPO: Jatun Sacha, 500 m (3 specimens MECN); sur oeste de Puerto Napo, 535 m, Pungarayacu (1 specimen MQCAZ). ORELLANA: Comunidad Kiwcha Chiruisla Station, 180–250 m (7 specimens MQCAZ); Dayuma Campo Palanda plataforma Primavera 1, 235 m (1 specimen MUTPL); Dayuma plataforma Ungurahua, 300 m (1 specimen MUTPL); Daimi (3 specimens MQCAZ); Estación de Biodiversidad Tiputini USFQ, 290 m, Parque Nacional Yasuní (2 specimens MUTPL); San Sebastian del Coca Comuna Guataraco Campo Pata, 345 m (1 specimen MUTPL); Yasuní Puce BS, 250 m, Río Tiputini (18 specimens MQCAZ); Lago San Pedro, plataforma Copal, 310 m (1 specimen MUTPL); Yampuna; Zamona-Yuturi (2 specimens MQCAZ). PASTAZA: Bosque Protector Oglán Alto, 645–810 m (2 specimens MUTPL); Estación Biológica Pindo Mirador UTE, 1000 m (1 specimen MUTPL). SUCUMBÍOS: Bermejo plataforma ER-A road to Lumbaqui (1 specimen MUTPL); Lago Agrio, 250 m (1 specimen MQCAZ); Limoncocha, 220 m (2 specimens MQCAZ); Pacayacu Campo Libertador, Tapi, 265 m (1 specimen MUTPL); Río Pañayacu Las Palmeras del Ecuador, 270 m (1 specimen MQCAZ); RPF Cuyabeno (4 specimens MQCAZ); Sacha Lodge, 270 m (2 specimens MQCAZ); Tarapoa, Nuevo Manabí, 270 m (1 specimen MUTPL).

####### Literature records.

MORONA SANTIAGO: Macuma (Edmonds 1994: 100). NAPO [= SUCUMBÍOS]: Lago Agrio, 250 m (Edmonds 1994: 100); Limoncocha, 250 m (Edmonds 1994: 100).

####### Temporal data.

Collected in January, February, March, April, May, June, July, August, September, November, and December.

####### Remarks.

Inhabits the lowland evergreen forests and evergreen foothill forests of the Amazon region from 180–1300 m a.s.l. Collected manually and using canopy fogging methods, flight interception traps and pitfall traps baited with carrion and human feces.

###### Phanaeus (Notiophanaeus) haroldi

Taxon classificationAnimaliaColeopteraScarabaeidae

Kirsch, 1871

Plate 47A

Phanaeus haroldi Kirsch, 1871: 342 (original description. Type locality: Bogotá).
Phanaeus haroldi : Nevinson 1892: 4 (catalog, distribution, written as PhanaeusHaroldi, Kirsch); Gillet 1911a: 83 (catalog, distribution, written as PhanaeusHaroldi, Kirsch); d’Olsoufieff 1924: 34 (characters in key); 85 (redescription), 147 (distribution); Blackwelder 1944: 209 (list of species from Latin America); Vulcano and Pereira 1967: 575 (characters in key); Vitolo 2000: 597 (characters in key); Medina et al. 2001: 140 (cited for Colombia); Noriega et al. 2009: 406 (distribution); Krajcik 2012: 204 (complete list of species); Ratcliffe et al. 2015: 197 (cited for Peru).
Phanaeus (Notiophanaeus) haroldi : Edmonds 1994: 21 (characters in key), 24 (redescription), 101 (distribution); Vaz-de-Mello 2000: 194 (cited for Brazil); Arnaud 2002a: 82 (diagnosis); Vitolo 2004: 283 (diagnosis); Carvajal et al. 2011: 320–321 (cited for Ecuador); Edmonds and Zídek 2012: 12 (characters in key); Figueroa et al. 2014: 133 (distributional records from Peru); Chamorro et al. 2018: 98 (cited for Ecuador).
Phanaeus schneblei Frey, 1963: 558 (original description); Arnaud 2002a: 82 (cited as synonym of Phanaeusharoldi Kirsch, 1871).

####### Type specimens.

*Phanaeusharoldi* Kirsch, 1871. The holotype (♀) is deposited at the SMTD (see Edmonds 1994: 24). Locality: Bogota, not examined.

*Phanaeusschneblei* Frey, 1963. Type material not examined.

####### Distribution.

Colombia, Ecuador, Peru, and Venezuela.

####### Records examined.

MORONA SANTIAGO: Huambi, 900 m (2 specimens CEMT); Logroño (1 specimen MQCAZ); Taisha (1 specimen MQCAZ); km 8 road to Mendez-Paute (1 specimen MQCAZ). NAPO: 4 km de San Pedro de Arajuno, 370 m (1 specimen MQCAZ); Avila Viejo Ek olm, 750 m (1 specimen MQCAZ); Cotundo Comunidad Rumiñahui, 1070 m, Kuriurcu (1 specimen MUTPL); Pano (1 specimen MQCAZ); Puerto Napo, 480 m (2 specimens MQCAZ; 1 specimen MECN); Talag Marungachi, 750 m (1 specimen MQCAZ). ORELLANA: Coca (1 specimen MQCAZ); Dayuma Campo Palanda, Pindo 14, 255 m (3 specimens MUTPL); El Coca plataforma Oso B, 250 m (1 specimen MECN); Estación Científica Yasuní PUCE, 250 m (8 specimen MQCAZ); Rodrigo Borja IAMOE (6 specimens, CEMT; 2 specimens MQCAZ; 1 specimen MECN); Ines Arango road Tiwino-río Shiripuno, 250 m (1 specimen MQCAZ); Taracoa (1 specimen MQCAZ). PASTAZA: Bosque Protector Oglán Alto, 555 m (1 specimen MUTPL); Cononaco (1 specimen MQCAZ). SUCUMBÍOS: 6 km de Dureno, Precooperativa Los Vergeles, 306 m (1 specimen MGO-UC); Campo Bermejo, 600 m (1 specimen MECN); Shusufindi Recinto la Pantera, 250 m (1 specimen CEMT). ZAMORA CHICNHIPE: km 1 road Cumbaritza-Gualaquiza (1 specimen MQCAZ); El Pangui, Eneretza, 925 m (1 specimen MQCAZ); Tunantza, Timbara (1 specimen MQCAZ).

####### Literature records.

LOJA: without specific locality (Noriega et al. 2009: 406). NAPO: Aliñahui Amazon Basin (Noriega et al. 2009: 406); Archidona (Noriega et al. 2009: 406); Pto Misahualli (Noriega et al. 2009: 406); 29 km E of San Pedro de Arajuno, 362 m (Noriega et al. 2009: 406); Tena, 400 m (Edmonds 1994: 101; Noriega et al. 2009: 406); Tena, 650 m (Noriega et al. 2009: 406). ORELLANA: Payamino Research Station, 400 m (Noriega et al. 2009: 407); Loreto (Edmonds 1994: 101; Noriega et al. 2009: 407). PASTAZA: Puyo (Edmonds 1994: 101; Noriega et al. 2009: 407); Canelos (Edmonds 1994: 101; Edmonds 1994: 101; Noriega et al. 2009: 407). PICHINCHA: Quito (Noriega et al. 2009: 407). SUCUMBÍOS: Lago Agrio, 250 m (Edmonds 1994: 101; Noriega et al. 2009: 407).

####### Temporal data.

Collected in February, April, May, June, July, August, September, October, November, and December.

####### Remarks.

Inhabits the lowland evergreen forests and evergreen foothill forests of the Amazon region from 255–1070 m a.s.l. Collected with pitfall traps baited with human feces. The record for Quito is probably erroneous.

###### Phanaeus (Notiophanaeus) meleagris

Taxon classificationAnimaliaColeopteraScarabaeidae

Blanchard, 1843

Plate 47B

Phanaeus meleagris Blanchard, 1843: 176 (original description. Type locality: Province de Santa-Cruz de la Sierra, Yungas).
Phanaeus meleagris : Gemminger and Harold 1869: 1018 (catalog, written as *Phanaes Meleagris* Blanch); Nevinson 1892: 5 (catalog, distribution); Gillet 1911a: 84 (catalog, distribution); d’Olsoufieff 1924: 41 (characters in key); 99 (redescription), 152 (distribution); Blackwelder 1944: 210 (list of species from Latin America); Balthasar 1941: 351 (cited for Peru); Balthasar 1951: 336 (cited for Peru); Vulcano and Pereira 1967: 574 (characters in key); Medina et al. 2001: 140 (cited for Colombia); Hamel-Leigue et al. 2006: 17 (cited for Bolivia); Krajcik 2012: 204 (complete list of species); Ratcliffe et al. 2015: 197 (cited for Peru).
Phanaeus (Phanaeus) meleagris : Pessôa 1934: 305 (characters in key), 312 (redescription).
Phanaeus (Notiophanaeus) meleagris : Edmonds 1994: 28 (characters in key), 31 (redescription), 102 (distribution); Vitolo 2000: 597 (characters in key); Arnaud 2002a: 85 (diagnosis); Vitolo 2004: 284 (diagnosis); Hamel-Leigue et al. 2009: 65 (distributional records from Bolivia); Carvajal et al. 2011: 322–323 (cited for Ecuador); Edmonds and Zídek 2012: 11 (characters in key); Figueroa et al. 2014: 136 (distributional records from Peru); Chamorro et al. 2018: 82 (figures 5G and 5H), 98 (cited for Ecuador).
Phanaeus (Notiophanaeus) meleagrisminos : Arnaud 2002a: 86 (diagnosis); Carvajal et al. 2011: 322–323 (cited for Ecuador); Krajcik 2012: 204 (cited as subespecie for Phanaeusmeleagris Blanchard).
Phanaeus minos Erichson, 1847: 106 (original description); Gemminger and Harold 1869: 1018 (catalog, cited as *Phanaes Minos* Erichs); Harold 1870: 105 (synonym of Phanaeusmeleagris Blanch); Nevinson 1892: 5 (synonym of Phanaeusmeleagris Blanchard); Gillet 1911a: 84 (synonym of Phanaeusmeleagris Blanchard); d’Olsoufieff 1924: 152 (synonym of Phanaeusmeleagris Blanchard); Pessôa 1934: 312 (synonym of Phanaeusmeleagris Blanchard); Blackwelder 1944: 210 (synonym of Phanaeusmeleagris Blanchard); Hamel-Leigue et al. 2009: 65 (synonym of Phanaeusmeleagris Blanchard).

####### Type specimens.

*Phanaeusmeleagris* Blanchard, 1843. The lectotype (♂) is deposited at the MNHN (see Edmonds 1994: 31). Locality: Yungas, not examined.

*Phanaeusminos* Erichson, 1847. The lectotype (♂) is deposited at the NMHU (see Edmonds 1994: 31). Locality: mountains of Peru, not examined.

####### Distribution.

Bolivia, Colombia, Ecuador, Peru, and Venezuela.

####### Records examined.

LOJA: Rta Loja-Zamora, 1400 m (7 specimens MQCAZ). MORONA SANTIAGO: Comunidad Ángel Rouby, 1300–1700 m, Cordillera del Kutukú (11 specimens MQCAZ); Comunidad Unsuants, Cordillera del Kutukú, 500–1100 m (8 specimens MQCAZ). Mera (3 specimens MQCAZ). NAPO: Bosque Protector La Cascada Quebrada Granadillas, 1300 m, Parque Nacional Sumaco (1 specimen MUTPL); Bosque Protector la Cascada Río Coca, 640 m (1 specimen MUTPL); Punte Río Quijos, 1402 m, Parque Nacional Sumaco (1 specimen MUTPL); Puente Río el Salado-Río Quijos, 1280 m, Parque Nacional Sumaco (1 specimen MUTPL); Río Hollín, 1100 m (4 specimens MQCAZ). PASTAZA: Estación Biologica Pindo Mirador UTE, 1000 m (1 specimen MUTPL). SUCUMBÍOS: Gonzalo Pizarro Simón Bolivar, 1200 m (1 specimen MECN). TUNGURAHUA: Baños El Topo, 1590 m (2 specimens MUTPL); San Francisco del Río Pastaza, 1200 m (3 specimens MQCAZ). ZAMORA CHINCHIPE: Palanda (1 specimen MUTPL); Chito Río San Francisco, 1800 m (2 specimens CEMT; 2 specimens MUTPL); Tundayme campamento Mirador, La Escombrera, 1223 m (1 specimen MUTPL); Tundayme campamento Mirador, Cara de Indio; Yantzatza, 1477 m (1 specimen MUTPL); Zurmi, Comunidad La Wants, 1000 m (2 specimens MUTPL).

####### Literature records.

PASTAZA: Mera (Edmonds, 1994: 102); Canelos (Edmonds 1994: 102). PASTAZA [= TUNGURAHUA]: San Francisco del Río Pastaza, 1200 m (Edmonds 1994: 102). ZAMORA CHINCHIPE: Sabanilla (Edmonds 1994: 102).

####### Temporal data.

Collected in February, March, April, August, September, October, and December.

####### Remarks.

Inhabits the evergreen foothill forests and evergreen lower montane forests across the Amazon region from 500–1800 m a.s.l. Collected with flight interception traps and pitfall traps baited with carrion and human feces.

###### Phanaeus (Notiophanaeus) pyrois

Taxon classificationAnimaliaColeopteraScarabaeidae

Bates, 1887

Plate 47C

Phanaeus pyrois Bates, 1887: 58 (original description. Type locality: Nicaragua, Chontales; Costa Rica: Panama, Bugaba, Chiriqui Volcano, 2000–3000 feet [= 610–915 m]; South America, Colombia).
Phanaeus pyrois : Nevinson 1892: 6 (catalog, distribution); Gillet 1911a: 85 (complete list of species); d’Olsoufieff 1924: 37 (characters in key); 93 (comment, distribution), 152 (distribution); Blackwelder 1944: 210 (list of species from Latin America); Medina et al. 2001: 140 (cited for Colombia); Krajcik 2012: 204 (complete list of species); Moctezuma and Halffter 2017: 55 (characters in key, cited for Ecuador); Kohlmann et al. 2018: 79 (figures 8b and d), 83 (characters in key, cited for Ecuador).
Phanaeus (Notiophanaeus) pyrois : Edmonds 1994: 41 (characters in key), 45 (redescription), 103 (distribution); Vitolo 2000: 597 (characters in key); Vitolo 2004: 284 (diagnosis); Carvajal et al. 2011: 322–323 (cited for Ecuador); Edmonds and Zídek 2012: 13 (characters in key), 53 (distribution figure 143); Bezdek and Hajek 2013: 432 (cited as junior subjective synonym of Phanaeus (Notiophanaeus) pyrois Bates, 1887. Catalog of the types of the NMPC); Chamorro et al. 2018: 84 (figure 7D), 98 (cited for Ecuador); Arnaud 2018: 4 (comment), 5 (figures 2e-d).
Phanaeus blanchardi d’Olsoufieff, 1924: 37 (characters in key), 92 (original description, cited for Ecuador); Vulcano and Pereira 1967: 575 (characters in key); Martínez and Pereira 1967: 68 (synonym of Phanaeus (Phanaeus) funereus Balthasar, 1939. Cited for Ecuador); Edmonds, 1994: 45 (synonym of Phanaeuspyrois Bates, 1887); Vitolo 2004: 284 (cited as synonym for Phanaeuspyrois Bates, 1887); Carvajal et al. 2011: 320–321 (cited for Ecuador); Edmonds and Zídek, 2012: 5 (cited as jr. prim. hom. of blanchardi Harold. Permanently unavailable, valid name pyrois Bates), 13 (comment).
Phanaeus (s. str.) funereus Balthasar, 1939: 241 (original description, cited for Ecuador).
Phanaeus funereus : Blackwelder 1944: 209 (list of species from Latin America); Martínez and Pereira 1967: 68 (distribution, comment); Edmonds 1994: 45 (synonym of Phanaeuspyrois Bates, 1887); 103 (distribution); Edmonds and Zídek 2012: 5 (cited as junior synonym for Phanaeuspyrois Bates, 1887).
Phanaeus (Notiophanaeus) pyroisfunereus : Arnaud 2002a: 97 (diagnosis); Carvajal et al. 2011: 322–323 (cited for Ecuador).

####### Type specimens.

*Phanaeuspyrois* Bates, 1887. The lectotype (♂) is deposited at the NHML (ex coll. H. Bates). Locality: Chontales, Nicaragua, examined.

**Lectotype** (♂): “Chontales / Nicaragua / T. Belt. [p]”, “P. pyrois ♂ [hw]”, “B.C.A. / p. 58, sp 8. [p]”, “Sp. figured. [p]”, “Phanaeus / pyrois. Bates / Lectotype ♂ / P. ARNAUD DET 1980 [hw and p, red margin]”.

*Phanaeusblanchardi* d’Olsoufieff, 1924. The lectotype (♂) is deposited at the MNHN (see Arnaud 1982a: 116). Locality: Colombie Vallée de Cauca [= Colombia, Valle del Cauca], not examined.

Phanaeus(s. str.)funereus Balthasar, 1939. The holotype (♂) is deposited at the NMPC (ex coll. V. Balthasar). Locality: Pucay [= Bucay], examined.

**Holotype** (♂): “ECUADOR / Pucay / F.O., 10.6.05 [p]”, “♂ [hw]”, “H. Blut Determ. / Phanaeus / blanchardi Olsuf. [hw and p]”, “Felsche det. / Phanaeus / pyrois Bts. [hw and p]”, “Ph. funereus / m. n.sp. / Dr. v. Balthasar det. [hw and p]”, “Typus [p, red label, black margin]”, “Mus. Nat. Pragae / Inv. [p], 26347 [hw, orange label]”, “ex coll. V. Balthasar / National Museum Prague / Czech Republic [p]”.

####### Distribution.

Colombia and Ecuador.

####### Records examined.

BOLIVAR: Altamira de Echeandia, 520–720 m (1 specimen MUTPL). CARCHI: Tobar Donoso, 300 m (5 specimens MECN). ESMERALDAS: Colón del Onzole (1 specimen MQCAZ; 8 specimens MECN); Chisprero (5 specimens MECN); E C Río Canandé, 390 m (2 specimens MQCAZ); Jeyambi PMFC (1 specimen MUTPL; 4 specimens MECN); Kumanii Lodge, 40 m (1 specimen MQCAZ); Majua (1 specimen CEMT; 7 specimens MECN); Gualpi El Pajonal (7 specimens MECN); Palma Real (1 specimen CEMT; 5 specimens MECN); Playa de Oro Estero Pote, 200 m (6 specimens CEMT; 11 specimens MECN); Playa de Oro La Tabla (7 specimens MECN; 2 specimens MQCAZ); Playa de Oro Playa Rica (4 specimens MECN); Playa de Oro Padre Santo (1 specimen MQCAZ; 9 specimens MECN); Salto del Bravo (5 specimens MECN); Tjespi (7 specimens MECN); Tjespi río Zapallo (1 specimen MQCAZ). GUAYAS: Guayaquil, 50 m (1 specimen MQCAZ); Pucay [= Bucay] (1 specimen NMPC). IMBABURA: El Chontal, El Cauchero, 900 m (1 specimen MUTPL); Lita, 680 (3 specimens MECN). LOS RÍOS: Quevedo Pichilingue (1 specimen CEMT). MANABÍ: Puerto López, Guale, 310 m (1 specimen MUTPL); Puerto López, Las Tunas, 200 m (1 specimen MUTPL); Puerto López, Puerto Rico, 110 m (1 specimen MUTPL). PICHINCHA: Guayabilla, 515 m, Río Guayllabamba, Manduriacus (1 specimen CEMT); El Encuentro, 620 m, San Miguel de los Bancos (3 specimens MUTPL); El Tigre Río Guayllabamba, Pedro Vicente Maldonado (2 specimens MUTPL); Llurimaguas, 290 m, Río Guayllabamba, Pedro Vicente Maldonado (1 specimen MUTPL); Mangaloma, 820 m, San Miguel de los Bancos (1 specimen MUTPL); Mindo, 1500 m (2 specimens MECN); San Roque, 580 m, Río Guayllabamba, Pedro Vicente Maldonado (2 specimens MUTPL); Tortugo Río Guayllabamba, 450 m, Pedro Vicente Maldonado (1 specimen MUTPL). SANTA ELENA: La Rinconada, 10 m (1 specimen MQCAZ); Olón 50 m (2 specimens CEMT). SANTO DOMINGO DE LOS TSÁCHILAS: Otongachi, 960 m (3 specimens MQCAZ); Santo Domingo, 500 m (2 specimens MECN).

####### Literature records.

ESMERALDAS: 11 km SE San Lorenzo (Edmonds 1994: 103); San Mateo (Martínez and Pereira 1967: 68). LOS RÍOS: Quevedo, 45 m (Edmonds 1994: 103). MANABÍ: 78 km NE de Chone, 450 m (Edmonds 1994: 103). PICHINCHA [= SANTO DOMINGO DE LOS TSÁCHILAS]: 4 km SE de Santo Domingo, 500 m (Edmonds 1994: 103). UNDETERMINED PROVINCE: without specific locality (Balthasar 1939e: 242).

####### Temporal data.

Collected every month of the year.

####### Remarks.

Inhabits coastal lowland evergreen forests and coastal evergreen foothill forests from 45–820 m a.s.l. Species was collected with pitfall traps baited with fungus, carrion, and human feces.

##### Subgenus Phanaeus (Phanaeus) Macleay, 1819

Phanaeus (Phanaeus) s. str. Macleay, 1819: 124 (original description. Type species: *Scarabaeuscarnifex* Linnaeus, 1767 original combination); d’Olsoufieff 1924: 79 (redescription); Pessôa 1934: 304 (cited as subgenus of *Phanaeus* Macleay, 1819); Pessôa and Lane 1941: 475 (characters in key); Martínez 1959: 103 (cited as subgenus of *Phanaeus* Macleay, 1819); Halffter and Matthews 1966: 258 (cited as subgenus of *Phanaeus* Macleay, 1819); Vulcano and Pereira 1967: 570 (characters in key); Edmonds 1994: 18 (characters in key), 46 (redescription); Arnaud 2002a: 78 (characters in key), 99 (diagnosis); Morón 2003: 60 (diagnosis); Vitolo 2004: 286 (diagnosis); Hamel-Leigue et al. 2009: 67 (distributional records from Bolivia); Vaz-de-Mello et al. 2011: 25 (characters in key); Edmonds and Zídek 2012: 7 (characters in key); Figueroa et al. 2014: 136 (distributional records from Peru); Chamorro et al. 2018: 75 (characters in key), 98 (list of species from Ecuador).

###### Phanaeus (Phanaeus) lunaris

Taxon classificationAnimaliaColeopteraScarabaeidae

Taschenberg, 1870

Plate 47D

Phanaeus lunaris Taschenberg, 1870: 183 (original description. Type locality: Loja, Ecuador).
Phanaeus lunaris : Nevinson 1892: 5 (catalog, distribution); Gillet 1911a: 84 (catalog, distribution); d’Olsoufieff 1924: 46 (characters in key); 110 (redescription), 156 (distribution); Blackwelder 1944: 210 (list of species from Latin America); Vulcano and Pereira 1967: 573 (characters in key); Streit 2008: 8 (distribution, photography); Krajcik 2012: 204 (complete list of species); Ratcliffe et al. 2015: 197 (cited for Peru).
Phanaeus (Phanaeus) lunaris : Edmonds 1994: 62 (characters in key), 67 (redescription), 102 (distribution); Arnaud 2002a: 114 (diagnosis); Carvajal et al. 2011: 322–323 (cited for Ecuador); Edmonds and Zídek 2012: 18 (characters in key); Figueroa et al. 2014: 136 (distributional records from Peru); Chamorro et al. 2018: 84 (figure 7C), 98 (cited for Ecuador).
Phanaeus charon Harold, 1880b: 151 (original description); Nevinson 1892: 2 (catalog, distribution, written as PhanaeusCharon Harold); Gillet 1911a: 84 (synonym of Phanaeuslunaris Taschb); Gillet 1911b: 319 (synonym of Phanaeuslunaris TASCH); d’Olsoufieff 1924: 156 (cited as synonym of Phanaeuslunaris Taschb); Blackwelder 1944: 210 (synonym of Phanaeuslunaris Taschb); Edmonds 1994: 67 (synonym of Phanaeus (Phanaeus) lunaris Taschenberg, 1870); Arnaud 2002a: 89 (cited as synonym of Phanaeus (Phanaeus) lunaris Taschenberg); Edmonds and Zídek 2012: 5 (junior synonym of Phanaeuslunaris Taschenberg); Figueroa et al. 2014: 136 (synonym of Phanaeuslunaris Taschenberg, 1870).

####### Type specimens.

*Phanaeuslunaris* Taschenberg, 1870. Type material not examined.

*Phanaeuscharon* Harold, 1880. Type material not examined.

####### Distribution.

Ecuador and Peru.

####### Records examined.

AZUAY: Santa Isabel río Jubones, 1035 m (4 specimens MQCAZ). CAÑAR: Javin (8 specimens CFPL); without specific locality (2 specimens CEMT). LOJA: 20 km N de Amaluza, 1250 m (2 specimens MQCAZ); Malacatos (2 specimens MQCAZ); Sozoranga (3 specimens MQCAZ); Vilcabamba, 1520 m (1 specimen MQCAZ). LOS RÍOS: Quevedo, 75 m (8 specimens CEMT); Quevedo, Pichilingue (2 specimens CEMT); La Clementina, 200 m (3 specimens MQCAZ). SANTA ELENA: Olón, 50 m (13 specimens CEMT). SANTO DOMINGO DE LOS TSÁCHILAS: Santo Domingo de los Colorados (1 specimen CEMT).

####### Literature records.

AZUAY: Huigra 1300 m (Edmonds 1994: 102). BOLIVAR: Balzapamba (Edmonds 1994: 102). EL ORO: Zaruma (Edmonds 1994: 102). ESMERALDAS: San Mateo (Edmonds 1994: 102); Telimbelo (Edmonds 1994: 102). GUAYAS: Guayaquil (Edmonds 1994: 102). LOS RÍOS: Quevedo, 75 m (Edmonds 1994: 102); Babahoyo (Edmonds 1994: 102); 45 km N Babahoyo 700 feet [= 210 m] (Edmonds 1994: 102). LOJA: Loja (Edmonds 1994: 102); Cariamanga (Edmonds 1994: 102). E35, 24 km S of Catamayo, 1811 m (Streit 2008: 8).

####### Temporal data.

Collected in January, February, March, May, June, July, September, November, and December.

####### Remarks.

Inhabits coastal lowland evergreen forests and coastal evergreen foothill forests from 50–1500 m a.s.l. In the Andean region, it was recorded in the evergreen lower montane forests from 1520–1810 m a.s.l. Collected with pitfall traps baited with human and cow feces.

#### Genus *Scatimus* Erichson, 1847

*Scatimus* Erichson, 1847: 110 (original description. Type species: *Scatimuscucullatus* Erichson, 1847 by monotypy).

*Scatimus*: Lacordaire 1856: 92 (redescription); Harold 1868b: 54 (characters in key); Gemminger and Harold 1869: 1001 (catalog); Bates 1887: 43 (distribution); Gillet 1911a: 49 (catalog, distribution); Lucas 1920: 582 (catalog, distribution); Paulian 1938: 232 (characters in key); Balthasar 1939i: 90 (list of species); Pessôa and Lane 1941: 440 (comment); Blackwelder 1944: 203 (catalog); Halffter and Matthews 1966: 259 (catalog, distribution); Vulcano and Pereira 1967: 575 (characters in key); Howden and Young 1981: 13 (characters in key); 48 (redescription); Halffter and Edmonds 1982: 137 (catalog, distribution); Kohlmann and Solís 1996: 99 (redescription); Medina and Lopera 2000: 306 (characters in key); Vaz-de-Mello 2000: 194 (list of species from Brazil); Medina et al. 2001: 139 (list of species from Colombia); Ratcliffe 2002: 15 (list of species from Panama); Génier and Kohlmann 2003: 72 (revision); Morón 2003: 57 (list of species from Mexico); Vaz-de-Mello 2008: 8 (comment), 14 (characters in key); Vaz-de-Mello et al. 2011: 22 (characters in key); Carvajal et al. 2011: 131 (diagnosis), 316 (list of species from Ecuador); Krajcik, 2012: 238 (complete list of species); Solís and Kohlmann 2012: 5 (list of species from Costa Rica); Chamorro et al. 2018: 74 (characters in key), 98 (list of species from Ecuador).

##### Scatimus cribrosus

Taxon classificationAnimaliaColeopteraScarabaeidae

Génier & Kohlmann, 2003

Plate 48A

Scatimus cribrosus Génier & Kohlmann, 2003: 81 (original description. Type locality: ECU: Pich.; 250 m, 47 km S Sto. Domingo, Río Palenque Sta. [= provincia de Los Ríos]).
Scatimus cribrosus : Carvajal et al. 2011: 316–317 (cited for Ecuador); Krajcik 2012: 238 (complete list of species); Chamorro et al. 2018: 98 (cited for Ecuador).

###### Type specimens.

*Scatimuscribrosus* Génier & Kohlmann, 2003. The holotype (♂) is deposited at the CMNC (see Génier and Kohlmann 2003: 84). Locality: ECUADOR Pichincha, 250 m, 47 km S Sto. Domingo, Río Palenque Sta, not examined.

###### Distribution.

Only known from Ecuador.

###### Records examined.

CAÑAR: Javín, 850–1300 m (5 specimens, CEMT). LOS RÍOS: 47 km S Santo Domingo, 215 m (1 specimen CEMT); Estación Científica Río Palenque, 230–250 m (3 specimens CEMT). SANTO DOMINGO DE LOS TSÁCHILAS: Tinalandia, 850 m (3 specimens CEMT).

###### Literature records.

PICHINCHA [= LOS RÍOS]: 47km S Sto. Domingo, Río Palenque Sta, 250m (Génier and Kohlmann 2003: 84); 47 km S Sto. Domingo, 700 feet [= 215 m] (Génier and Kohlmann 2003: 84); Río Palenque sta, 250 m (Génier and Kohlmann 2003: 84); Río Palenque sta, 230 m (Génier and Kohlmann 2003: 84). CAZAR [= CAÑAR]: R[ou]te La Troncal-CaZar [= Ruta la Troncal-Cañar] near Suscal, 1200 m (Génier and Kohlmann 2003: 84). GUAYAS: Bucay (Génier and Kohlmann 2003: 84). LOS RÍOS: Quevedo, Pichilinque (Génier and Kohlmann 2003: 84); Río Palenque Biol. Sta (Génier and Kohlmann 2003: 84). PICHINCHA [= SANTO DOMINGO DE LOS TSÁCHILAS]: 4 km SE Sto. Domingo, 500 m (Génier and Kohlmann 2003: 84); 15 km E Sto. Domingo, Tinalandia, 700 m (Génier and Kohlmann 2003: 84); 16 km SE Sto. Domingo, Tinalandia, 680m (Génier and Kohlmann 2003: 84).

###### Temporal data.

Collected in January, February, March, May, June, July, October, and December.

###### Remarks.

Inhabits coastal lowland evergreen forests and coastal evergreen foothill forests from 215–1300 m a.s.l. Collected with pitfall traps baited with human feces.

##### Scatimus fernandezi

Taxon classificationAnimaliaColeopteraScarabaeidae

Martínez, 1988

Plate 48B

Scatimus fernandezi Martínez, 1988b: 85 (original description. Type locality: Maracay, Estado Barinas, Santa Bárbara, Solano).
Scatimus fernandezi : Medina et al. 2001: 139 (cited for Colombia); Génier and Kohlmann 2003: 64 (characters in key); 76 (redescription); Carvajal et al. 2011: 316–317 (cited for Ecuador); Chamorro et al. 2018: 98 (cited for Ecuador).

###### Type specimens.

*Scatimusfernandezi* Martínez, 1988. The holotype (♂) is deposited at the MACN (see Martínez 1988b: 85). Locality: Maracay, Estado Barinas, Santa Bárbara, Solano, not examined.

###### Distribution.

Colombia, Ecuador, Trinidad, and Venezuela.

###### Records examined.

ORELLANA: Dayuma Campo Palanda-Yuca Sur, 235 m, plataforma Primavera 1 (1 specimen MUTPL); Eden, Campo Eden plataforma G, 220 m (1 specimen CEMT); Indillama, Río Tiputini Parque Nacional Yasuní, 220 (1 specimen MUTPL). SUCUMBÍOS: Aucayacu Río El Eno, 16 km de Lago Agrio, 275 m (1 specimen MUTPL); Limoncocha, Yamanunca, 225 m (1 specimen CEMT); Pacayacu, 280 m (1 specimen CEMT).

###### Literature records.

NAPO [= SUCUMBÍOS]: Limoncocha, 250 m (Génier and Kohlmann 2003: 77).

###### Temporal data.

Collected in May, August, November, and December.

###### Remarks.

Inhabits the lowland evergreen forests of the Amazon region from 220–280 m a.s.l. Collected with canopy fogging methods and pitfall traps baited with human feces.

##### Scatimus furcatus

Taxon classificationAnimaliaColeopteraScarabaeidae

Balthasar, 1939

Plate 48C

Scatimus furcatus Balthasar, 1939i: 88 (original description. Type locality: Ecuador).
Scatimus furcatus : Blackwelder 1944: 203 (catalog); Vulcano and Pereira 1967: 576 (characters in key); Génier and Kohlmann 2003: 64 (characters in key); 89 (redescription); Carvajal et al. 2011: 316–317 (cited for Ecuador); Bezdek and Hajek 2011: 356 (catalog of types NMPC); Krajcik 2011: 356 (complete list of species); Chamorro et al. 2018: 80 (figure 3H), 98 (cited for Ecuador).

###### Type specimens.

*Scatimusfurcatus* Balthasar, 1939. The holotype (♀) is deposited at the NMPC (see Génier and Kohlmann 2003: 91). Locality: Ecuador, not examined.

###### Distribution.

Only known from Ecuador.

###### Records examined.

PICHINCHA: Cerro San Cristobal Curipoglio, 1800 m (1 specimen CEMT); Choconde San Miguel de los Bancos, 1200 m (1 specimen CEMT); E.B. La Hesperia, 1200 m (1 specimen CEMT); Mindo, 1500 m (1 specimen MQCAZ); road to Chiriboga, 1400 m (1 specimen MQCAZ).

###### Literature records.

PICHINCHA [= SANTO DOMINGO DE LOS TSÁCHILAS]: 29 km E Alluriquín (Génier and Kohlmann 2003: 91). PICHINCHA: Chiriboga Rd., 1400m (Génier and Kohlmann 2003: 91); La Armenia, 1400m (Génier and Kohlmann 2003: 91); Mindo, 1500m (Génier and Kohlmann 2003: 91).

###### Temporal data.

Collected in January, March, June, and December.

###### Remarks.

Inhabits the evergreen lower montane forests of the Andean region from 1400–1800 m a.s.l. Collected with pitfall traps baited with human feces.

##### Scatimus monstrosus

Taxon classificationAnimaliaColeopteraScarabaeidae

Balthasar, 1939

Plate 48D

Scatimus monstrosus Balthasar, 1939i: 88 (original description. Type locality: Ecuador: Loja, Sigiro, Arenal, Catamayo, Ciano).
Scatimus monstrosus : Blackwelder 1944: 203 (catalog); Vulcano and Pereira 1967: 576 (characters in key); Génier and Kohlmann 2003: 63 (characters in key); 97 (redescription); Bezdek and Hajek 2011: 356 (catalog of types NMPC); Carvajal et al. 2011: 316–317 (cited for Ecuador); Krajcik 2011: 356 (complete list of species); Chamorro et al. 2018: 98 (cited for Ecuador).

###### Type specimens.

*Scatimusfurcatus* Balthasar, 1939. The lectotype (♂) and three paralectotype are deposited at the NMPC (see Génier and Kohlmann 2003: 98). Locality: Ecuador, Loja, not examined.

###### Distribution.

Ecuador and Peru.

###### Records examined.

LOJA: 5 km N de Gonzanama, 2400 m (1 specimen CEMT); Cariamanga (3 specimens MQCAZ); Cariamanga, Utuana, 2500 m (1 specimen MQCAZ); Jimbura, 2100 m (1 specimen CEMT); Olmedo (2 specimens MQCAZ); Sozoranga (1 specimen MUTPL).

###### Literature records.

LOJA: Catamayo (Balthasar 1939i: 89; Génier and Kohlmann 2003: 98); Ciano (Balthasar 1939i: 89; Génier and Kohlmann 2003: 98); Hacienda El Arenal (Balthasar 1939i: 89; Génier and Kohlmann 2003: 98; Bezdek and Hajek 2011: 357); Hacienda Sigiro (Balthasar 1939i: 89; Génier and Kohlmann 2003: 98; Bezdek and Hajek 2011: 357). UNDETERMINED PROVINCE: without specific locality (Génier and Kohlmann 2003: 98; Bezdek and Hajek 2011: 356).

###### Temporal data.

Collected in January, February, March, and May.

###### Remarks.

Inhabits the montane cloud forests and the evergreen high montane forests of the Andean region from 2100–2500 m a.s.l. The collection method is unknown.

##### Scatimus onorei

Taxon classificationAnimaliaColeopteraScarabaeidae

Génier & Kohlmann, 2003

Plate 49A

Scatimus onorei Génier & Kohlmann, 2003: 92 (original description. Type locality: ECUADOR: Loja, Celica).
Scatimus onorei : Donoso et al. 2009: Appendix II. 18 (catalog of types MQCAZ); Carvajal et al. 2011: 316–317 (cited for Ecuador); Krajcik 2012: 238 (complete list of species); Chamorro et al. 2018: 98 (cited for Ecuador).

###### Type specimens.

*Scatimusonorei* Génier & Kohlmann, 2003. The holotype (♂) is deposited at the MQCAZ (see Génier and Kohlmann 2003: 94). Locality: Ecuador, Loja, Célica, not examined.

###### Distribution.

Only known form Ecuador.

###### Records examined.

EL ORO: Arenillas, 15 m (2 specimens CEMT). LOJA: El Tundo, Papayo (1 specimen MUTPL); Macará Cangonama Chico, Reserva Laipuna, 830 m (1 specimen CEMT).

###### Literature records.

AZUAY: Palmar (Génier and Kohlmann 2003: 94). LOJA: Celica (Génier and Kohlmann 2003: 94; Donoso et al. 2009: Appendix II. 18).

###### Temporal data.

Collected in February, March, April, and June.

###### Remarks.

Inhabits coastal lowland semi-deciduous forests and coastal matorral dry montane forests from 15–830 m a.s.l. Collected with pitfall traps baited with human feces.

##### Scatimus pacificus

Taxon classificationAnimaliaColeopteraScarabaeidae

Génier & Kohlmann, 2003

Plate 49B

Scatimus pacificus Génier & Kohlmann, 2003: 87 (original description. Type locality: Ecuador: Guayas Prov., 25 km SW Guayaquil, 50m).
Scatimus pacificus : Carvajal et al. 2011: 316–317 (cited for Ecuador); Krajcik 2012: 238 (complete list of species); Chamorro et al. 2018: 98 (cited for Ecuador).

###### Type specimens.

*Scatimuspacificus* Génier & Kohlmann, 2003. The holotype (♂) is deposited at the CMNC (see Génier and Kohlmann 2003: 89). Locality: Guayas Prov., 25 km SW Guayaquil, 50 m, not examined.

###### Distribution.

Only known from Ecuador.

###### Literature records.

GUAYAS: 25 km SW Guayaquil, 50 m (Génier and Kohlmann 2003: 89); 45 km W Guayaquil (Génier and Kohlmann 2003: 89); Guayaquil, 50 m (Génier and Kohlmann 2003: 89). MANABÍ: 20 km N Chone, 300 m (Génier and Kohlmann 2003: 89); Bahía de Caráquez (Génier and Kohlmann 2003: 89); Chone (Génier and Kohlmann 2003: 89). UNDETERMINED PROVINCE: without specific locality (Génier and Kohlmann 2003: 89).

###### Temporal data.

Collected in February, March, May, and June.

###### Remarks.

Inhabits coastal lowland semi-deciduous forests from 50–300 m a.s.l. According to Génier and Kohlmann (2003), this species was collected with pitfall traps baited with human feces.

##### Scatimus strandi

Taxon classificationAnimaliaColeopteraScarabaeidae

Balthasar, 1939

Plate 49C

Scatimus strandi Balthasar, 1939i: 87 (original description. Type locality: Ecuador).
Scatimus strandi : Blackwelder 1944: 203 (catalog); Vulcano and Pereira 1967: 575 (characters in key); Medina et al. 2001: 139 (cited for Colombia); Génier and Kohlmann 2003: 64 (characters in key); 94 (redescription); Carvajal et al. 2011: 316–317 (cited for Ecuador); Bezdek and Hajek 2011: 357 (catalog of the types of the NMPC); Krajcik 2011: 238 (complete list of species); Ratcliffe et al. 2015: 197 (cited for Peru); Chamorro et al. 2018: 81 (figure 4D), 98 (cited for Ecuador).

###### Type specimens.

*Scatimusstrandi* Balthasar, 1939. The holotype (♂) is deposited at the NMPC (see Krajcik 2011: 357). Locality: Ecuador, not examined.

###### Distribution.

Colombia, Ecuador, and Peru.

###### Records examined.

MORONA SANTIAGO: Comunidad Ángel Rouby, 1300 m, Cordillera del Kutukú (1 specimen MUTPL; 3 specimens MQCAZ); Comunidad Unsuants, 900–1100 m, Cordillera del Kutukú (1 specimen MQCAZ). NAPO: Quebrada Granadillas Bosque Protector La Cascada, 1300 m, Parque Nacional Sumaco (2 specimens MUTPL); Río El Salado-Río Quijos, 1280 m (1 specimen CEMT); Río Chonta Yacu, 1100 m, road Tena-Coca (4 specimens CEMT); PASTAZA: Mera, Estación Biologica Pindo Mirador ETE, 1000 m (1 specimen MUTPL). TUNGURAHUA: Baños El Topo, 1590 m (7 specimens CEMT). ZAMORA CHINCHIPE: Tundayme campamento Ecsa, Jardín Botánico, 925 m (1 specimen CEMT); Tundayme campamento Mirador, La Escombrera, 1225 m (1 specimen MUTPL); Zurmi, Pachikuntza, 1685 m (1 specimen MUTPL).

###### Literature records.

NAPO: Archidona; km 7.3 Sarayacú-Loreto Rd., 1200m (Génier and Kohlmann 2003: 95); km 11.1 Sarayacú-Loreto Rd., 1200 m (Génier and Kohlmann 2003: 95). SUCUMBÍOS: Reventador, 1400 m (Génier and Kohlmann 2003: 95). TUNGARAHUA [= PASTAZA]: 6 km E Río Negro, 1500 m (Génier and Kohlmann 2003: 95); 8 km E Río Negro, 1400m (Génier and Kohlmann 2003: 95). UNDETERMINED PROVINCE: without specific locality (Génier and Kohlmann 2003: 95; Bezdek and Hajek 2011: 357).

###### Temporal data.

Collected in January, February, March, November, and December.

###### Remarks.

Inhabits the evergreen foothill forests and evergreen lower montane forests across the Amazonian range from 900–1685 m a.s.l. Collected with pitfall traps baited with carrion and human feces.

#### Genus *Scybalocanthon* Martínez, 1948

*Scybalocanthon* Martínez, 1948b: 4 (original description. Type species: *Canthonmoniliatus*Bates 1887 = *Scybalocanthonmonilliatus* (Bates, 1887) cited as comb. n.).

*Scybalocanthon*: Martínez 1949b: 188 (comment); Pereira and Martínez 1956a: 96 (characters in key), 114 (list of species); Halffter 1961: 231 (characters in key); Vulcano and Pereira 1964: 637 (catalog of species); Halffter and Matthews 1966: 261 (catalog, distribution); Vulcano and Pereira 1967: 549 (characters in key); Halffter and Martínez 1977: 38 (characters in key), 67 (diagnosis), 68 (list of species); Halffter and Edmonds 1982: 139 (catalog, distribution); Medina and Lopera 2000: 311 (characters in key); Vaz-de-Mello 2000: 195 (list of species from Brazil); Medina et al. 2001: 137 (list of species from Colombia); Medina et al. 2003: 65 (distribution); Hamel-Leigue et al. 2006: 15 (list of species from Bolivia); Vaz-de-Mello et al. 2011: 26 (characters in key); Carvajal et al. 2011: 116 (diagnosis), 314 (list of species from Ecuador); Krajcik 2012: 63 (cited as subgenus of *Canthon* Hoffmannsegg, 1817); Solís and Kohlmann 2012: 3 (cited as subgenus of *Canthon* Hoffmannsegg, 1817); Boilly and Vaz-de-Mello 2013: 108 (characters in key); Chamorro et al. 2018: 76 (characters in key), 98 (list of species from Ecuador).

##### Scybalocanthon kaestneri

Taxon classificationAnimaliaColeopteraScarabaeidae

(Balthasar, 1939)

Plate 49D

Canthon kästneri Balthasar, 1939d: 227 (original description. Type locality: Ecuador).
Canthon kästneri : Blackwelder 1944: 199 (list of species of Latin America); Pereira and Martínez 1956a: 114 (comment); Vulcano and Pereira 1964: 616 (cited for Ecuador); Carvajal et al. 2011: 314–315 (cited for Ecuador); Krajcik 2012: 63 (complete list of species).
Scybalocanthon kastneri : Halffter and Martínez 1977: 68 (cited as S.kastneri (Balthasar), list of species); Carvajal et al. 2011: 314–315 (cited for Ecuador); Bezdek and Hajek 2011: 363 (catalog of types NMPC); Chamorro et al. 2018: 87 (figure 10B), 98 (cited for Ecuador).

###### Type specimens.

*Canthonkästneri* Balthasar, 1939. Two syntypes examined deposited at the NMPC. Lectotype to be designated in a future work on this species group.

**Syntype** (♀): “Ecuador [hw]”, “Typus [p, red label, black margin]”, “kästneri m. [hw, green label, black margin]”, “Scybalocanthon / kaestneri (Balth.) / Det. B.D. Gill‘96 [p, blak margin]”.

**Syntype** (♀): “Mera / Ecuador [p]”, “Typus [p, red label, black margin]”.

###### Distribution.

Only known from Ecuador.

###### Records examined.

MORONA SANTIAGO: 1 specimen, Bosque Domoso, 1650 m (CEMT); Comunidad Ángel Rouby, 1300 m, Cordillera del Kutukú (3 specimens MECN; 26 specimens MQCAZ); Comunidad Unsuants, 900–1100 m, Cordillera del Kutukú (4 specimens MECN; 12 specimens MQCAZ). NAPO: Puerto Napo, 480 m (2 specimens MQCAZ). PASTAZA: Bosque Protector Oglán Alto, 810 m (1 specimen CEMT; 1 specimen MUTPL); Mera (1 specimen NMPC); Mera, Estación Pindo Mirador UTE, 1000 m (9 specimens CEMT). UNDETERMINED PROVINCE: without specific locality (1 specimen NMPC).

###### Temporal data.

Collected in January, February, May, June, August, October, and December.

###### Remarks.

Inhabits the evergreen foothill forests of the Amazon region from 810–1300 m a.s.l. Collected with pitfall traps baited with carrion and human feces.

##### Scybalocanthon maculatus

Taxon classificationAnimaliaColeopteraScarabaeidae

(Schmidt, 1920)

Plate 50A

Canthon maculatus Schmidt, 1920: 127 (original description. Type locality: Sante Jnéz [= Santa Inés], Ecuador).
Canthon maculatus : Schmidt 1922: 77 (distribution); Balthasar 1939d: 193 (characters in key); Carvajal et al. 2011: 314–315 (cited for Ecuador); Krajcik 2012: 64 (complete list of species).
Canthon maculatum : Blackwelder 1944: 200 (list of species from Latin America).
Scybalocanthon maculatus : Pereira and Martínez 1956a: 115 (characters in key), 119 (cited as n. comb.); Vulcano and Pereira 1964: 638 (catalog of species); Vulcano and Pereira 1967: 555 (catalog of species); Halffter and Martínez 1977: 68 (cited as S.macultatus (Schmidt), list of species); Carvajal et al. 2011: 316–317 (cited for Ecuador); Chamorro et al. 2018: 98 (cited for Ecuador); Vaz-de-Mello and Cupello 2018b: 45 (cited for Ecuador), 46 (figure 29).

###### Type specimens.

*Canthonmaculatus* Schmidt, 1920. Thirteen syntypes examined deposited at the NMHU, ZMHB, and SMTD (see Vaz-de-Mello and Cupello 2018b: 45–47). Lectotype to be designated in a future work on this species group.

**Syntype** (♂): “Santa Jnéz / (Ecuad.) / R.Haensch S. [p, black margin]”, “Typus [p, red label, black margin]”, “maculatus / Type m. [hw]”, “Typ. [p]”, “9846 / E92 + [p, red label]”.

**Syntype** (♂): “Santa Jnéz / (Ecuad.) / R.Haensch S. [p, black margin]”, “9843 / E92 + [p, red label]”, “Scybalocanhton / maculatus (Schm) / P. Pereira det. 60 [p and hw, black margin]”.

**Syntype** (♂): “Santa Jnéz / (Ecuad.) / R.Haensch S. [p, black margin]”, “9842 / E92 + [p, red label]”, “Scybalocanhton / maculatus (Schm) / P. Pereira det. 60 [p and hw, black margin]”.

**Syntype** (♀): “Santa Jnéz / (Ecuad.) / R.Haensch S. [p, black margin]”, “9842 / E92 + [p, red label]”.

**Syntype** (♀): “Santa Jnéz / (Ecuad.) / R.Haensch S. [p, black margin]”, “9844 / E92 + [p, red label]”, “26 / 56 [p and hw, pink label]”.

**Syntype** (♂): “Santa Jnéz / (Ecuad.) / R.Haensch S. [p, black margin]”, “[one face, p] 106947 [oposite face, hw] 106947”.

**Syntype** (♂): “Ecuador / Baron [p]”, “Coll. C. Felsche / Kauf 20, 1918 [p, green label, black margin]”.

**Syntype** (♂): “Santa Jnéz / (Ecuad.) / R.Haensch S. [p, black margin]”, “Coll. C. Felsche / Kauf 20, 1918 [p, green label, black margin]”.

**Syntype** (♀): “Santa Jnéz / (Ecuad.) / R.Haensch S. [p, black margin]”, “106947 [hw]”, “Canthon / maculatus / n. sp. a. Schmidt [hw]”.

**Syntype** (♂): “Ecuador / 5255 [hw, green label]”, “Canthon / maculatus / n. sp. a. Schmidt [hw]”.

**Syntype** (♀): “Santa Jnéz / (Ecuad.) / R.Haensch S. [p, black margin]”, “Coll. C. Felsche / Kauf 20, 1918 [p, green label, black margin]”.

**Syntype** (♀): “Ecuador / Baron [p]”, “Coll. C. Felsche / Kauf 20, 1918 [p, green label, black margin]”.

**Syntype** (♀): “Santa Jnéz / (Ecuad.) / R.Haensch S. [p, black margin]”, “Coll. C. Felsche / Kauf 20, 1918 [p, green label, black margin]”, “Canthon / maculatus / n. sp. a. Schmidt [hw]”, “Typus [p, red label]”.

###### Distribution.

Only known from Ecuador.

###### Records examined.

NAPO: Cosanga Río Cosanga, 2000 m (1 specimen MQCAZ); El Salado, Rio Malo, 1315 m (1 specimen MQCAZ); Los Guacamayos Piviyacu, 1800 m (1 specimen CEMT); Pacto Sumaco, 1620 m (4 specimens MUTPL); Río Hollín, 1100 m (3 specimens CEMT); Río Jondachi, La Merced de Jondachi, 1175 m (3 specimens CEMT); San Rafael (1 specimen MQCAZ); road Hollín-Loreto km 7 (1 specimen MQCAZ). PASTAZA: Mera, Estación Pindo Mirador UTE, 1000 m (2 specimens CEMT). TUNGURAHUA: 4.3 km E de Río Negro, 1200 m (2 specimens CEMT); Baños El Topo, 1590 m (20 specimens CEMT). SUCUMBÍOS: El Reventador (1 specimen MQCAZ); Gonzalo Pizarro, Simon Bolivar, 1200 m (4 specimens MECN); Lumbaqui (1 specimen MQCAZ). UNDETERMINED PROVINCE: Santa Inés (5 specimens NMRS; 3 specimens SMTD; 2 specimens ZMHB); without specific locality (3 specimens SMTD).

###### Literature records.

UNDETERMINED PROVINCE: Santa Inez (Vaz-de-Mello and Cupello 2018b: 45–46); without specific locality (Vaz-de-Mello and Cupello 2018b: 47).

###### Temporal data.

Collected in January, May, April, June, August, and December.

###### Remarks.

Inhabits the evergreen foothill forests and evergreen lower montane forests across the Amazonian range from 1000–1800 m a.s.l. Collected with pitfall traps baited with human feces.

##### Scybalocanthon moniliatus

Taxon classificationAnimaliaColeopteraScarabaeidae

(Bates, 1887)

Plate 50B

Canthon moniliatus Bates, 1887: 27 (original description. Type locality: PANAMA, Bugaba).
Canthon moniliatus : Gillet 1911a: 31 (complete list of species); Schmidt 1922: 77 (distribution); Balthasar 1939d: 191 (characters in key); Howden and Young 1981: 21 (characters in key), 22 (redescription); Solís and Kohlmann 2002: 4 (characters in key), 36 (redescription); Krajcik 2012: 64 (complete list of species).
Canthon moniliatum : Blackwelder 1944: 200 (list of species for Latin America).
Scybalocanthon moniliatus : Martínez 1948b: 6 (new combination, distribution); Martínez 1949b: 189 (characters in key); Pereira and Martínez 1956a: 114 (characters in key), 115 (distribution); Vulcano and Pereira 1964: 638 (catalog of species); Vulcano and Pereira 1967: 554 (characters in key); Halffter and Martínez 1977: 68 (cited as S.moniliatus (Bates), list of species); Medina et al. 2003: 65 (distribution); Ratcliffe et al. 2015: 195 (cited for Peru); Chamorro et al. 2018: 98 (cited for Ecuador).

###### Type specimens.

*Canthonmoniliatus* Bates, 1887. Three syntypes examined deposited in NHML. Lectotype to be designated in a future work on this species group.

###### Distribution.

Costa Rica, Ecuador, Mexico, Nicaragua, Panama, and Peru.

###### Records examined.

COTOPAXI: Guasaganda km 4, 500 m (1 specimen MQCAZ).

###### Temporal data.

Collected in December.

###### Remarks.

Inhabits coastal lowland evergreen forests at 500 m a.s.l. Collected with pitfall traps baited with human feces.

##### Scybalocanthon trimaculatus

Taxon classificationAnimaliaColeopteraScarabaeidae

(Schmidt, 1922)

Plate 50C

Canthon trimaculatus Schmidt, 1922: 94 (original description. Type locality: Cachábé, Columbien [= Colombia], Paramba, Surinam).
Canthon trimaculatus : Balthasar 1939d: 191 (characters in key); Vulcano and Pereira 1964: 633 (catalog of species); Krajcik 2012: 64 (complete list of species).
Canthon trimaculatum : Blackwelder 1944: 202 (list of species from Latin America); Contreras 1951: 221 (cited for Colombia).
Scybalocanthon trimaculatus : Vulcano and Pereira 1967: 554 (new combination, catalog of species); Halffter and Martínez 1977: 68 (cited as S.trimaculatus (Schmitd), list of species); Medina et al. 2001: 137 (cited for Colombia); Medina et al. 2003: 65 (distribution); Carvajal et al. 2011: 316–317 (cited for Ecuador); Ratcliffe et al. 2015: 195 (cited for Peru); Chamorro et al. 2018: 98 (cited for Ecuador); Vaz-de-Mello and Cupello 2018b: 69 (figure 105), 70 (cited for Ecuador).

###### Type specimens.

*Canthontrimaculatus* Schmidt, 1922. Eight syntypes examined deposited at the NMHU, NMRS and SMTD. Lectotype to be designated in a future work on this species group.

###### Distribution.

Colombia, Ecuador, and Peru.

###### Records examined.

CAÑAR: Javín, 850–1300 m (1 specimen CEMT; 5 specimens MQCAZ). CARCHI: Cabeceras Río Baboso (8 specimens MQCAZ); Tobar Donoso, 300 m (5 specimens MECN). COTOPAXI: Guasaganda km 4, 500 m (1 specimen CEMT; 7 specimens MQCAZ). ESMERALDAS: Carondelet (11 specimens MQCAZ; 5 specimens MECN); Colón del Ónzole (27 specimens MQCAZ; 16 specimens MECN); Charco Vicente (7 specimens MGO-UC; 22 specimens MQCAZ; 17 specimens MECN); Chispero (10 specimens MGO-UC; 14 specimens MQCAZ; 8 specimens MECN); Gualpi (19 specimens MQCAZ; 11 specimens MECN); Jeyambi (9 specimens MQCAZ; 4 specimens MECN); Kumanii Lodge, 40 m (14 specimens MQCAZ); El Progreso (10 specimens MQCAZ; 4 specimens MECN); Estación Forestal La Chiquita, 60 m (1 specimen CEMT; 7 specimens MQCAZ); E.C. Río Canandé, 400 m (18 specimens MQCAZ); Majua (14 specimens MGO-UC; 22 specimens MECN; 28 specimens MQCAZ); Nuevo Ecuador (6 specimens MQCAZ); Palma Real (12 specimens MGO-UC; 11 specimens MECN; 18 specimens MQCAZ); Playa de Oro, La Tabla (2 specimens MGO-UC; 33 specimens MQCAZ; 16 specimens MECN); Playa de Oro, Pote (4 specimens CEMT; 6 specimens MGO-UC; 11 specimens MQCAZ; 7 specimens MECN); Playa de Oro, Padre Santo (16 specimens MGO-UC; 37 specimens MQCAZ); Playa de Oro, Playa Rica (13 specimens MGO-UC; 16 specimens MQCAZ; 9 specimens MECN); Ricauter (7 specimens MQCAZ); Tsejpi (8 specimens MGO-UC; 16 specimens MQCAZ; 5 specimens MECN); Tsejpi, Cuartel II (5 specimens MQCAZ); Tsejpi, Charco Grande (4 specimens MQCAZ); Zabalito (1 specimen MQCAZ). IMBABURA: Lita, 680 m (5 specimens MQCAZ). LOS RÍOS: Río Palenque Estación Científica, 150–220 m (3 specimens CEMT; 19 specimens MQCAZ). PICHINCHA: Chiriboga Road, 1200 m (1 specimen CEMT; 4 specimens MQCAZ); Los Bancos (8 specimens MQCAZ); Puerto Quito (3 specimens MQCAZ); Río Guayllabamba Guayabilla, Manduriacus, 520 m (3 specimens MUTPL). SANTO DOMINGO DE LOS TSACHILAS: La Perla (5 specimens MQCAZ).

###### Temporal data.

Collected in January, February, March, April, May, June, July, August, September, October, November, and December.

###### Remarks.

Inhabits coastal lowland evergreen forests and coastal evergreen foothill forests from 60–1300 m a.s.l. Collected with pitfall traps baited with carrion and human feces. Since Schmidt (1922), no future specimens of *S.trimaculatus* were recorded from Surinam.

#### Genus *Sinapisoma* Boucomont, 1928

*Sinapisoma* Boucomont, 1928c: 3 (original description. Type species: *Sinapisomaminutum* Boucomont, 1928 by monotypy).

*Sinapisoma*: Boucomont 1928a: 186 (diagnosis); Paulian 1938: 234 (characters in key); Paulian 1939: 30 (redescription); Balthasar 1939d: 236 (comment); Balthasar 1941: 344 (cited for Peru); Blackwelder 1944: 203 (list of species from Latin America); Balthasar 1951: 329 (cited for Peru); Pereira and Martínez 1956a: 94 (characters in key); Halffter 1961: 230 (characters in key); Vulcano and Pereira 1964: 584 (catalog of species); Halffter and Matthews 1966: 261 (catalog, distribution); Halffter and Martínez 1968: 241 (diagnosis); Vulcano and Pereira 1967: 548 (characters in key); Halffter and Martínez 1977: 35 (characters in key); Halffter and Edmonds 1982: 139 (catalog, distribution); Vaz-de-Mello 2000: 195 (list of species from Brazil); Medina et al. 2001: 137 (list of species from Colombia); Vaz-de-Mello et al. 2011: 27 (characters in key); Krajcik 2012: 245 (complete list of species); Boilly and Vaz-de-Mello 2013: 108 (characters in key); Chamorro et al. 2018: 76 (characters in key), 88 (figure 11A), 98 (cited for Ecuador).

**Remarks.** Thus far, no species have been recorded from Ecuador. However, on compiling this catalog, we found a possible new species from Orellana province. Its description will be included in a future work on this genus.

#### Genus *Streblopus* Lansberge, 1874

*Streblopus* Lansberge, 1874a: 9 (original description. Type species: *Streblopusopatroides* Lansberge, 1874).

*Streblopus*: Gillet 1911a: 42 (complete list of species); Lucas 1920: 617 (catalog, distribution); Paulian 1938: 234 (characters in key); Paulian 1939: 26 (redescription); Blackwelder 1944: 203 (list of species from Latin America); Pereira and Martínez 1956a: 94 (characters in key); Halffter 1961: 230 (characters in key); Vulcano and Pereira 1964: 580 (catalog of species); Halffter and Matthews 1966: 260 (catalog, distribution); Vulcano and Pereira 1967: 548 (characters in key); Halffter and Martínez 1966: 154 (diagnosis); Halffter and Martínez 1977: 34 (characters in key); Halffter and Edmonds 1982: 139 (catalog, distribution); Vaz-de-Mello 2000: 195 (list of species from Brazil); Vaz-de-Mello et al. 2011: 25 (characters in key); Carvajal et al. 2011: 122 (diagnosis), 316 (list of species from Ecuador); Krajcik 2012: 249 (complete list of species); Chamorro et al. 2018: 76 (characters in key), 98 (list of species from Ecuador).

*Colonychus* Harold, 1868d: 10 (nom. nud. Type species: unnamed); Gillet 1911a: 42 (cited as synonym de *Streblopus* Lansberge, 1874); Lucas 1920: 197 (cited as synonym of *Streblopus* Lansberge, 1874); Paulian 1939: 26 (cited as synonym of *Streblopus* Lansberge, 1874); Blackwelder 1944: 203 (cited as synonym of *Streblopus* Lansberge, 1874); Pereira and Martínez 1956a: 99 (cited as synonym of *Streblopus* Lansberge, 1874); Vulcano and Pereira 1964: 580 (cited as synonym of *Streblopus* Lansberge, 1874); Halffter and Martínez 1966: 152 (comment, cited as n. nudum).

*Streblopoides* Balthasar, 1938: 215 (original description. Type species: *Streblopoidespunctatus* Balthasar, 1938); Blackwelder 1944: 203 (list of species from Latin America); Pereira and Martínez 1956a: 99 (comment); Vulcano and Pereira 1964: 580 (catalog of species); Halffter and Matthews 1966: 260 (catalog, distribution); Halffter and Martínez 1966: 153 (synonym of *Streblopus* Lansberge, 1874).

##### Streblopus punctatus

Taxon classificationAnimaliaColeopteraScarabaeidae

(Balthasar, 1938)

Plate 50D

Streblopoides punctatus Balthasar, 1938: 216 (original description. Type locality: Nord-Peru, Huancabamba, 3000 m).
Streblopoides punctatus : Balthasar 1941: 346 (cited for Peru); Vulcano and Pereira 1964: 580 (catalog of species); Balthasar 1951: 331 (cited for Peru).
Streblopoides punctata : Blackwelder 1944: 203 (list of species from Latin America).
Streblopus punctatus : Halffter and Martínez 1966: 162 (cited as new combination, redescription); Carvajal et al. 2011: 316–317 (cited for Ecuador); Bezdek and Hajek 2011: 374 (catalog of the types of the NMPC); Carvajal 2012: 196 (redescription), 197 (distribution); Krajcik 2012: 249 (complete list of species); Ratcliffe et al. 2015: 196 (cited for Peru); Chamorro et al. 2018: 85 (figure 8C), 98 (cited for Ecuador).

###### Type specimens.

*Streblopoidespunctatus* Balthasar, 1938. The holotype is deposited at the NMPC (ex coll. V. Balthasar). Locality: Huancabamba, N Peru, 3000 m, examined.

**Holotype** (♀): “Huancabamba / N. Peru, 3000m / H. Rolle [p]”, “Typus [p, red label, black margin]”, “Genotyp / Str. punctatus / n.sp. / Dr. V. Balthasar det. [p and hw]”, “punctatus / m. [hw, green label]”.

###### Distribution.

Ecuador and Peru.

###### Records examined.

ZAMORA CHINCHIPE: RVS El Zarza conseción Zarza, Cordillera del Cóndor, 1555 m (3 specimens CEMT; 2 specimens MUTPL); RVS El Zarza conseción Colibrí, Cordillera del Cóndor, 1445 m (1 specimen MEPN); Yantzatza T3, 1435 m (2 specimens CEMT); Zurmi Comunidad Miazi, 1380 m (1 specimen MEPN; 1 specimen MUTPL).

###### Temporal data.

Collected in January, September, November, and December.

###### Remarks.

Inhabits the evergreen lower montane forests across the Amazonian range from 1380–1555 m a.s.l. Collected with pitfall traps baited with human feces.

#### Genus *Sulcophanaeus* d’Olsoufieff, 1924

*Sulcophanaeus* d’Olsoufieff, 1924: 23 (original description. Type species: *Scarabaeussulcatus* Drury, 1770 by original designation).

*Sulcophanaeus*: Blackwelder 1944: 209 (list of species from Latin America); Halffter and Matthews 1966: 258 (cited as subgenus of *Phanaeus* Macleay, 1819); Vulcano and Pereira 1967: 570 (characters in key. Cited as subgenus of *Phanaeus* Macleay, 1819); Edmonds 1972: 820 (characters in key), 821 (redescription); Howden and Young 1981: 12 (characters in key), 137 (redescription); Halffter and Edmonds 1982: 136 (catalog, distribution); Zunino 1985: 104 (comment); Edmonds 1994: 17 (characters in key); Edmonds 2000: 3 (revision); Medina and Lopera 2000: 303 (characters in key); Vitolo 2000: 595 (characters in key); Vaz-de-Mello 2000: 195 (list of species from Brazil); Medina et al. 2001: 140 (list of species from Colombia); Arnaud 2002a: 14 (characters in key); Ratcliffe 2002: 17 (list of species from Panama); Morón 2003: 60 (diagnosis); Vitolo 2004: 280 (diagnosis); Philips et al. 2004: 50 (comment); Hamel-Leigue et al. 2006: 18 (list of species from Bolivia); Hamel-Leigue et al. 2009: 67 (distribution of records from Bolivia); Vaz-de-Mello et al. 2011: 25 (characters in key); Carvajal et al. 2011: 139 (diagnosis), 320 (list of species from Ecuador); Solís and Kohlmann 2012: 8 (list of species from Costa Rica); Krajcik 2012: 204 (cited as subgenus of *Phanaeus* Macleay, 1819); Boilly and Vaz-de-Mello 2013: 107 (characters in key); Figueroa et al. 2014: 132 (distribution of records for Peru); Chamorro et al. 2018: 75 (characters in key), 98 (list of species from Ecuador).

*Eucopricus* Gistel, 1857: 602 (nomen oblitum, synonym of *Sulcophanaeus* d’Olsoufieff, 1924. Type species: *Phanaeuscolumbi* Macleay, 1819 original designation); Edmonds 2000: 3 (cited as nome oblitum, synonym of *Sulcophanaeus* d’Olsoufieff, 1924); Solís and Kohlmann 2012: 8 (cited as synonym of *Sulcophanaeus* d’Olsoufieff, 1924); Figueroa et al. 2014: 132 (cited as synonym of *Sulcophanaeus* d’Olsoufieff, 1924).

##### Sulcophanaeus faunus

Taxon classificationAnimaliaColeopteraScarabaeidae

(Fabricius, 1775)

Scarabaeus faunus Fabricius, 1775: 23 (original description. Type locality: Cayennae).
Scarabaeus faunus : Olivier 1789: 103 (redescription).
Copris faunus : Olivier 1790: 154 (new combination under the genus Copris “Müller, 1764”, redescription); Sturm 1802: 62 (redescription).
Phanaeus faunus : Gemminger and Harold 1869: 1017 (list, distribution, cited as new combination and mentioned as PhanaeusFaunus Fabr); Nevinson 1892: 4 (list of species of the genus Phanaeus, mentioned as PhanaeusFaunus Fabricius); Gillet 1911a: 83 (complete list of species); d’Olsoufieff, 1924: 32 (characters in key), 80 (diagnosis), 146 (distribution); Blackwelder 1944: 209 (list of species from Latin America).
Phanaeus (Phanaeus) faunus : Pessôa 1934: 304 (characters in key) 305 (redescription); Vulcano and Pereira 1967: 572 (characters in key).
Sulcophanaeus faunus : Edmonds 2000: 8 (cited as new combination, characters in key), 9 (diagnosis); Vaz-de-Mello 2000: 195 (cited for Brazil); Vitolo 2000: 595 (characters in key); Medina et al. 2001: 140 (cited for Colombia); Arnaud 2002a: 131 (diagnosis); Vitolo 2004: 280 (diagnosis); Hamel-Leigue et al. 2006: 18 (cited for Bolivia); Hamel-Leigue et al. 2009: 67 (comment); Carvajal et al. 2011: 320–321 (cited for Ecuador); Krajcik 2012: 204 (cited as species of the genus Phanaeus Macleay, 1819); Boilly and Vaz-de-Mello 2013: 105 (figure 21); Figueroa et al. 2014: 132 (distributional records from Peru); Ratcliffe et al. 2015: 195 (cited for Peru); Boilly et al. 2016: 89 (figures 7a, 7b and 7c); 95 (cited for Guyana); Chamorro et al. 2018: 98 (cited for Ecuador).

###### Type specimens.

*Scarabaeusfaunus* Fabricius, 1775. Type material not examined.

###### Distribution.

Bolivia, Brazil, Colombia, Ecuador, French Guiana, Guyana, Paraguay, and Peru.

###### Records examined.

UNDETERMINED PROVINCE: without specific locality (1 specimen MECN.

###### Temporal data.

It is not known when this species was collected.

###### Remarks.

Ecological needs unknown, it may be found in Amazonian lowland forests. The collection method is unknown.

##### Sulcophanaeus miyashitai

Taxon classificationAnimaliaColeopteraScarabaeidae

Arnaud, 2002

Plate 51A

Sulcophanaeus miyashitai Arnaud, 2002c: 3 (original description. Type locality: Ecuador, Esmeraldas, Alto Tambo, 650 m).
Sulcophanaeus miyashitai : Arnaud 2002a: 140 (diagnosis); Carvajal et al. 2011: 320–321 (cited for Ecuador); Krajcik 2012: 204 (included in the genus Phanaeus Macleay, 1819); Chamorro et al. 2018: 98 (cited for Ecuador).

###### Types specimens.

*Sulcophanaeusmiyashitai* Arnaud, 2002. The holotype (♂) is deposited at the CPFA (see Arnaud 2002c: 3). Locality: Ecuador, Esmeraldas, Alto Tambo, 650 m (not examined).

###### Distribution.

Colombia and Ecuador.

###### Records examined.

CARCHI: Tobar Donoso, 300 m (3 specimens MECN). ESMERALDAS: Alto Tambo, 650 m (3 specimens MQCAZ); Carondelet (7 specimens MQCAZ; 3 specimens MECN); Chispero (1 specimen CEMT; 5 specimens MGO-UC; 10 specimens MQCAZ; 4 specimens MECN); Calle Mansa (3 specimens MGO-UC; 12 specimens MECN; 14 specimens MQCAZ); Charco Vicente (1 specimen MGO-UC; 3 specimens MECN; 9 specimens MQCAZ); Colón del Ónzole (1 specimen CEMT; 16 specimens MQCAZ; 5 specimens MECN); Gallinazo (3 specimens MQCAZ); Guadal (2 specimens MQCAZ; 3 specimens MECN); Gualpi El Pajonal (8 specimens MQCAZ; 5 specimens MECN); Jeyambi (4 specimens MGO-UC; 7 specimens MQCAZ); Majua (17 specimens MGO-UC; 12 specimens MQCAZ; 10 specimens MECN); Los Ajos (3 specimens MQCAZ); Palma Real (2 specimens CEMT; 11 specimens MQCAZ; 5 specimens MECN); Playa de Oro (2 specimens CEMT; 13 specimens MGO-UC; 8 specimens MQCAZ; 5 specimens MECN); Playa de Oro, Padre Santo (4 specimens CEMT; 11 specimens MQCAZ; 6 specimens MECN); Playa de Oro, Playa Rica (1 specimen CEMT; 1 specimen MGO-UC; 3 specimens MQCAZ); Playa de Oro, Pote (6 specimens CEMT; 17 specimens MGO-UC; 7 specimens MQCAZ; 5 specimens MECN); Playa de Oro, La Tabla (18 specimens MQCAZ; 5 specimens MECN); Tsejpi, Charco Grande (10 specimen MGO-UC; 4 specimens MQCAZ; 4 specimens MECN); Tsejpi, río Zapallo (3 specimens MQCAZ); Ricaurte (1 specimen MQCAZ); San Miguel (3 specimens MQCAZ); Santa Rita (2 specimens MQCAZ); Zabalito (1 specimen MQCAZ). IMBABURA: Lita, 680 m (2 specimens MQCAZ; 1 specimen MECN). LOS RÍOS: Quevedo, Estación Experimental Tropical Pichilingue (2 specimens MQCAZ); Río Palenque, Estación Biológica, 250 m (4 specimens MQCAZ).

###### Literature records.

ESMERALDAS: Alto Tambo, 650 m (Arnaud 2002c: 3); 11 km Se San Lorenzo (Arnaud, 2002c: 3). LOS RÍOS: Quevedo, Pichilingue (Arnaud, 2002c: 3). PICHINCHA [= LOS RÍOS]: Station. Biol. Río Palenque (Arnaud, 2002c: 3). IMBABURA: Paramba [= Parambas]. MANABÍ: 73 km NE de Chona [= Chone], 300 m (Arnaud, 2002c: 3). PICHINCHA [= SANTO DOMINGO DE LOS TSÁCHILAS]: Tinalandia (Arnaud, 2002c: 3).

###### Temporal data.

Collected in January, February, March, April, May, August, September, October, November, and December.

###### Remarks.

Inhabits coastal lowland evergreen forests and coastal evergreen foothill forests from 250–680 m a.s.l. Collected with pitfall traps baited with carrion and human feces.

##### Sulcophanaeus velutinus

Taxon classificationAnimaliaColeopteraScarabaeidae

(Murray, 1856)

Plate 51B

Phanaeus velutinus Murray, 1856: 213 (original description. Type locality. Ecuador, neighborhood of Quito).
Phanaeus velutinus : Gemminger and Harold 1869: 1020 (list, distribution); Nevinson 1892: 8 (list of species of the genus Phanaeus); Gillet 1911a: 87 (complete list of species).
Phanaeus (Phanaeus) velutinus : d’Olsoufieff 1924: 33 (characters in key), 83 (diagnosis), 147 (distribution); Blackwelder 1944: 210 (list of species from Latin America); Vulcano and Pereira 1967: 575 (characters in key).
Sulcophanaeus velutinus : Howden and Young 1981: 138 (characters in key, redescription); Edmonds 2000: 20 (characters in key), 23 (diagnosis); Vitolo 2000: 595 (characters in key); Medina et al. 2001: 140 (cited for Colombia); Arnaud 2002a: 135 (diagnosis); Ratcliffe 2002: 17 (cited for Panama); Vitolo 2004: 282 (diagnosis); Carvajal et al. 2011: 320–321 (cited for Ecuador); Krajcik 2012: 204 (cited as species of the genus Phanaeus Macleay, 1819); Solís and Kohlmann 2012: 8 (cited for Costa Rica); Chamorro et al. 2018: 81 (figure 4H), 84 (figure 7B), 98 (cited for Ecuador).

###### Type specimens.

*Phanaeusvelutinus* Murray, 1856. The holotype (♂) is deposited at the NHML. Locality: Quito, examined.

**Holotype** (♂): “Quito / 78.19 [hw]”, “Velutinus / (Type) Murray [hw]”, “Type [p, red margin]”, “9642 [hw]”, “Velutinus Murr / Edin. New. Phil. J. 1857/ Quito [hw]”, “Phanaeus / velutinus Murr. / LECTOTYPE ♂ / P. ARNAUD DET 1983 [p and hw, red margin]”, “Phanaeus / velutinus / Murray, 1856 / HOLOTYPE”.

###### Distribution.

Colombia, Costa Rica, Ecuador, and Panama.

###### Records examined.

IMBABURA: Santa Cecilia (2 specimens MECN). PICHINCHA: 10.6 km Mindo Road, 1460 m (1 specimen MQCAZ); Estación Biológica la Hesperia (2 specimens CEMT; 20 specimens MUTPL); Quito (1 specimen NHML). Pampas Argentinas (1 specimen MQCAZ).

###### Literature records.

AZUAY: Pucay [= Bucay, GUAYAS province] (Edmonds, 2000: 24). BOLIVAR: Balzapamba (d’Olsoufieff, 1924: 83); Chimbo (d’Olsoufieff, 1924: 83). CAÑAR: Javín, 1300 m (Edmonds, 2000: 24). LOS RÍOS: Quevedo, Pichilingue, 45 m (Edmonds, 2000: 24). LOS RÍOS [= SANTO DOMINGO DE LOS TSÁCHILAS]: Santo Domingo (Edmonds, 2000: 24).

###### Temporal data.

Collected in January, February, March, April, May, July, November, and December.

###### Remarks.

Inhabits coastal lowland evergreen forests and coastal evergreen foothill forests from 45–1460 m a.s.l. Collected with flight interception traps and pitfall traps baited with carrion, human and pig feces.

#### Genus *Sylvicanthon* Halffter & Martínez, 1977

*Sylvicanthon* Halffter & Martínez, 1977: 61 (original description. Type species: *Canthoncandezei* Harold, 1869 by original designation).

*Sylvicanthon*: Halffter and Edmonds 1982: 139 (catalog, distribution); Medina and Lopera 2000: 311 (characters in key); Vaz-de-Mello 2000: 195 (list of species from Brazil); Medina et al. 2001: 137 (list of species from Colombia); Medina et al. 2003: 65 (distribution); Hamel-Leigue et al. 2006: 15 (list of species from Bolivia); Vaz-de-Mello et al. 2011: 26 (characters in key); Carvajal et al. 2011: 117 (diagnosis), 316 (list of species from Ecuador); Krajcik 2012: 63 (complete list of species, cited as subgenus of *Canthon* Hoffmannsegg, 1817); Solís and Kohlmann 2012: 3 (list of species from Costa Rica, cited as subgenus of *Canthon* Hoffmannsegg, 1817); Boilly and Vaz-de-Mello 2013: 107 (characters in key); Chamorro et al. 2018: 76 (characters in key), 98 (list of species from Ecuador); Cupello and Vaz-de-Mello 2018: 20 (redescription), 56 (characters in key).

##### Sylvicanthon bridarollii

Taxon classificationAnimaliaColeopteraScarabaeidae

(Martínez, 1949)

Plate 51C

Glaphyrocanthon bridarollii Martínez, 1949c: 283 (original description, Type locality: Bolivia, Dep. de Cochabmaba, Peia de Chapare, Río Coni 400 m).
Glaphyrocanthon bridarollii : Martínez 1949a: 171 (distribution); Pereira and Martínez 1956a: 126 (characters in key); 129 (distribution); Vulcano and Pereira 1964: 661 (catalog of species); Vulcano and Pereira 1967: 561 (characters in key).
Canthon bridarollii : Krajcik 2012: 63 (complete list of species, cited as subgenus of Canthon Hoffmannsegg, 1817).
Sylvicanthon bridarollii : Halffter and Martínez 1977: 63 (cited as nov. comb.); Vaz-de-Mello 2000: 195 (cited for Brazil); Medina et al. 2001: 137 (cited for Colombia); Medina et al. 2003: 65 (distribution); Hamel-Leigue et al. 2006: 15 (cited for Bolivia); Carvajal et al. 2011: 316–317 (cited for Ecuador); Ratcliffe et al. 2015: 196 (cited for Peru); Chamorro et al. 2018: 86 (figure 9D), 98 (written as Silvicanthonbridarollii (Martínez, 1948). Cited for Ecuador); Cupello and Vaz-de-Mello 2018: 58 (characters in key), 109 (figures 32A, B, C and D), 114: (redescription); 117 (figure 34 distribution).

###### Type specimens.

*Glaphyrocanthonbridarollii* Martínez, 1949. The holotype (♂) is deposited at the MACN. Locality: Bolivia, Dep. de Cochabmaba, Chapare, 400 m, examined.

**Holotype** (♂): “BOLIVIA / Dep. Cochabmaba / Chapare - 400 mts / R. Zischra - leg. / Coll. Martínez. [hw]”, “BOLIVIA / Chapare / 400 M. / Zischra [p, blak margin]”, “MACN-En / 937 [p, black margin]”, “HOLOTIPO ♂ [hw, red label]”, “Glaphyrocanthon / bridarolli ♂ / sp. n. / A. MARTÍNEZ. DET. 1949 [p and hw, red label, black margin]”.

###### Distribution.

Bolivia, Brazil, Colombia, Ecuador, and Peru.

###### Records examined.

MORONA SANTIAGO: Untsuant sítio 1, 700 m (3 specimens CMNC); Untsuant sítio 3, 700 m (1 specimen CMNC); Untsuant sítio 5, 600 m (4 specimens CMNC); Untsuant sítio 6, 600 m (7 specimens CMNC). NAPO: 5 km W de Tena (19 specimens CMNC); 20 km S de Tena, 600m (3 specimens CMNC); Puerto Misahualli, Jungle Hotel (3 specimens TAMU); Tena, 400 m (9 specimens CMNC). ORELLANA: Tiputini Biodiversity Station (2 specimens NHML). PASTAZA: Bosque Protector Oglán 590 m (1 specimen MGO-UC); Chuyayacu Oleoducto km 25, 200 m (1 specimen MGO-UC). SUCUMBÍOS: Reserva Biológica Limoncocha, 300 m (14 specimens CMNC).

###### Literature records.

MORONA SANTIAGO: Untsuants, Sítio 1, 700 m (Cupello and Vaz-de-Mello 2018: 111); Untsuante [= Untsuants], Sítio 3, 700 m (Cupello and Vaz-de-Mello 2018: 111); Untsuante [= Untsuants], Sítio 5, 600 m (Cupello and Vaz-de-Mello 2018: 111); Untsuante [Untsuants], Sítio 6, 600 m (Cupello and Vaz-de-Mello 2018: 111). NAPO: Puerto Misahualli, Jungle Hotel (Cupello and Vaz-de-Mello 2018: 111); Tena, 400 m (Cupello and Vaz-de-Mello 2018: 111); 5 km W Tena, 500 m (Cupello and Vaz-de-Mello 2018: 111) 20 km S Tena, 600 m (Cupello and Vaz-de-Mello 2018: 111). ORELLANA: Tiputini Biodiversity Station (Cupello and Vaz-de-Mello 2018: 112). SUCUMBÍOS: Shushufindi, Reserva Biológica Limoncocha, 300 m (Cupello and Vaz-de-Mello 2018: 112); Shushufindi, Limoncocha, 250 m (Cupello and Vaz-de-Mello 2018: 112).

###### Temporal data.

Collected in January, February, March, May, June, July, September, November, and December.

###### Remarks.

Inhabits the lowland evergreen forests and evergreen foothill forests of the Amazon region from 300–700 m a.s.l. Collected with pitfall traps baited with human feces.

##### Sylvicanthon edmonsi

Taxon classificationAnimaliaColeopteraScarabaeidae

Cupello & Vaz-de-Mello, 2018

Plate 51D

Sylvicanthon edmonsi Cupello & Vaz-de-Mello, 2018: 58 (characters in key), 117 (figure 34 distribution), 132 (original description. Type locality: Orellana, Parque Nacional Yasuní, Estación Científica Yasuní, 215 m), 134 (figures 38A, B).

###### Type specimens.

*Sylvicanthonedmonsi* Cupello & Vaz-de-Mello, 2018. The holotype (♂) is deposited st the TAMU (see Cupello and Vaz-de-Mello 2018: 132). Locality: Orellana, Parque Nacional Yasuní, Estación Científica Yasuní, 215 m, not examined.

###### Distribution.

Colombia, Ecuador, and Peru.

###### Literature records.

MORONA SANTIAGO: Untsuants, sítio 3, 700 m (Cupello and Vaz-de-Mello 2018: 132). ORELLANA: Parque Nacional Yasuní, Estación Científica Yasuní, 215 m (Cupello and Vaz-de-Mello 2018: 132); Parque Nacional Yasuní, via Maxus km Onkone Gare, 220 m (Cupello and Vaz-de-Mello 2018: 132); Rodrigo Borja, IAMOE (Cupello and Vaz-de-Mello 2018: 132); Tiputini Biodiversity Station, 220 m (Cupello and Vaz-de-Mello 2018: 132); Tiputini Biodiversity Station, Río Tiputini (Cupello and Vaz-de-Mello 2018: 132).

###### Temporal data.

Collected in January, June, July, August, September, and November.

###### Remarks.

Inhabits the lowland evergreen forests and the foothill evergreen forests of the Amazon region from 215–700 m a.s.l. According to Cupello and Vaz-de-Mello (2018), this species has been collected with flight interception trap, canopy fogging methods and pitfall traps baited with carrion and human feces.

##### Sylvicanthon genieri

Taxon classificationAnimaliaColeopteraScarabaeidae

Cupello & Vaz-de-Mello, 2018

Plate 52A

Sylvicanthon genieri Cupello & Vaz-de-Mello, 2018: 57 (characters in key), 72 (figure 24 distribution), 80 (original description. Type locality: Tungurahua, 6 km east from Río Negro, 1500 m), 84 (figure 27A, B).

###### Type specimens.

*Sylvicanthongenieri* Cupello & Vaz-de-Mello, 2018. The holotype (♂) is deposited at the CMNC (see Cupello and Vaz-de-Mello 2018: 81). Locality: Tungurahua, 6 km east from Río Negro, 1500 m, not examined.

###### Distribution.

Ecuador and Peru.

###### Literature records.

MORONA SANTIAGO: Untsuants, site 4, 1100 m (Cupello and Vaz-de-Mello 2018: 81); Untsuante [= Untsuants], site 7, 900 m (Cupello and Vaz-de-Mello 2018: 81). NAPO: km 7.3 Sarayacu-Loreto Road, 1200 m (Cupello and Vaz-de-Mello 2018: 81); km 11.1 Sarayacu-Loreto Road, 1200 m (Cupello and Vaz-de-Mello 2018: 81); km 25.4 Sarayacu-Loreto Road, 950 m (Cupello and Vaz-de-Mello 2018: 81). ORELLANA: Onkone Gare Camp, 220 m (Cupello and Vaz-de-Mello 2018: 81). PASTAZA: 09 km ESE Veracruz (Cupello and Vaz-de-Mello 2018: 81); 22 km SE Puyo, 900 m (Cupello and Vaz-de-Mello 2018: 81); Puyo, Llandia, 17 km N Puyo (Cupello and Vaz-de-Mello 2018: 81); Mera, 1 km E Mera, 1100 m (Cupello and Vaz-de-Mello 2018: 81). PASTAZA [= TUNGURAHUA]: 4.3 km Rio Negro, 1200 m (Cupello and Vaz-de-Mello 2018: 81). TUNGURAHUA: 3 km W Río Negro, 1200 m (Cupello and Vaz-de-Mello 2018: 81); 4.3 km E Río Negro, 1200 m (Cupello and Vaz-de-Mello 2018: 81); 6 km E Río Negro, 1500 m (Cupello and Vaz-de-Mello 2018: 81); Baños El Topo, 1590 m (Cupello and Vaz-de-Mello 2018: 81). TUNGURAHUA [= PASTAZA]: 8 km E Rio Negro, 10 km W Pastaza (= Shell), 1400 m (Cupello and Vaz-de-Mello 2018: 81). ZAMORA CHINCHIPE: Upper Río Comainas, Cordillera del Cóndor, 1150 m (Cupello and Vaz-de-Mello 2018: 81).

###### Temporal data.

Collected in January, July, August, and October.

###### Remarks.

Inhabits the lowland evergreen forests, evergreen foothill forests, and evergreen lower montane forests across the Amazonian range from 220–1590 m a.s.l. According to Cupello and Vaz-de-Mello (2018), this species has been collected beneath the leaf litter and with pitfall traps baited with human feces. The locality Upper Río Comainas, Cordillera del Cóndor, 1150 m a.s.l, and their coordinates 03°54'S, 78°25'W (see Cupello and Vaz-de-Mello 2018, page 81) are located in Peru, Amazonas.

##### Sylvicanthon proseni

Taxon classificationAnimaliaColeopteraScarabaeidae

(Martínez, 1949)

Plate 52B

Glaphyrocanthon proseni Martínez, 1949c: 287 (original description. Type locality: Bolivia, Dep. de la Paz, Pcia. de Nor. Yungas, río Choro, 700 m).
Glaphyrocanthon proseni : Martínez 1949a: 171 (catalog of species); Pereira and Martínez 1956a: 126 (characters in key), 128 (list of species); Martínez et al. 1964: 13 (characters in key); Vulcano and Pereira 1964: 663 (catalog of species); Vulcano and Pereira 1967: 561 (characters in key); Halffter and Martínez 1977: 91 (synonym of Canthon (Canthon) aequinoctialis (Harold, 1868), comment).
Canthon (Canthon) proseni : Chamorro et al. 2018: 87 (figures D and F), 92 (cited for Ecuador).
Sylvicanthon proseni : Cupello and Vaz-de-Mello 2018: 58 (characters in key), 87 (figures 28 C and D), 93 (figure 30 distribution), 96 (revalidated name and new combination), 101 (redescription).

###### Type specimens.

*Glaphyrocanthonproseni* Martínez, 1949. The holotype (♂) is deposited at the MACN. Locality: Bolivia, Dep. La Paz, Prov. Nor Yungas, examined.

**Holotype** (♂): “MACN-En / 1412 [p, black margin]”, “Ene-949 / BOLIVIA / Dep. La Paz / Prov. Nor Yungas / Ríos Corioco y Choro / 700 m alt / Coll. Martínez [hw]”, “HOLOTYPUS [p, red label]”, “glaphyrocanthon / proseni ♂ / sp. n / A. MARTÍNEZ-DET 1949 [p and hw, red label, black margin]”.

###### Distribution.

Bolivia, Brazil, Colombia, Ecuador, and Peru.

###### Records examined.

MORONA SANTIAGO: Cumpi, Cordillera del Kutukú (2 specimens MUTPL). NAPO: Estación Biológica Jatun Sacha, 450 m (61 specimens MQCAZ); Pungarayacu cerca al Tena, 505 m (1 specimen MQCAZ). ORELLANA: Bloque 31, Parque Nacional Yasuní, 200 m (17 specimens MQCAZ); Dayuma Campo Hormiguero Plataforma Hormiguero, 320 m (1 specimen MUTPL); Dayuma Campo Pindo, plataforma Pindo 14, 255 m (1 specimen MUTPL); Dayuma Campo Palanda-Yuca Sur, plataforma Primavera 1, 235 m (1 specimen MUTPL); Dayuma plataforma Ungurahua, 300 m (1 specimen MUTPL); Daimi (53 specimens MQCAZ); El Dorado plataforma Guarango, 300 m (1 specimen MUTPL); Estación Científica Yasuní PUCE, 250 m (3 specimens CEMT; 155 specimens MQCAZ); Estación de Biodiversidad Tiputini, Parque Nacional Yasuní (1 specimen MGO-UC; 14 specimens MQCAZ); Lago San Pedro, plataforma Copal, 310 m (1 specimen MUTPL); Rodrigo Borja IAMOE (4 specimens CEMT; 52 specimens MQCAZ); San Sebastian de Coca Comuna Guataraco Campo Pata, 345 m (1 specimen MGO-UC); San Sebastian de Coca Comuna Shamanal Campo Palo Azul, 345 m (1 specimen MUTPL); Yampuna (1 specimen MGO-UC). PASTAZA: Bosque Protector Oglán Alto, 550–945 m (1 specimen MUTPL); Nuevo San Jose del Curaray, 245 m (1 specimen MUTPL); Tipirishca (7 specimens MQCAZ). SUCUMBÍOS: 6 km de Dureno, Precooperativa Los Vergeles, 287 m (2 specimens MGO-UC); Aucayacu Río El Eno, 16 km de Lago Agrio, 290 m (13 specimens MGO-UC); Bermejo plataforma, ER-A road to Lumbaqui (1 specimen MUTPL); La Selva Bio Station 175 km E.S.E del Coca (7 specimens MQCAZ); Pacayacu Campo Libertador, 260 m (1 specimen MUTPL); Tarapoa, Nuevo Manabí, 270 m (1 specimen MUTPL). ZAMORA CHINCHIPE: Tundayme, campamento Mirador, Las Maravillas, 1060 m (1 specimen MUTPL); Tundayme, campamento Mirador, Enerentsa, 1030 m (1 specimen MUTPL); Zurmi Comunidad Miazi, 1380 m (1 specimen MEPN); Zurmi, Comunidad La Wants, 1010 m (1 specimen MEPN); Zurmi Las Orquideas Río Nangaritza, 870 m (1 specimen MUTPL).

###### Literature records.

ORELLANA: Estación Científica Yasuní, 215 m (Cupello and Vaz-de-Mello 2018: 99); Parque Nacional Yasuní, Scyasuni, 200 m (Cupello and Vaz-de-Mello 2018: 99); Payamino Research Station, 300 m (Cupello and Vaz-de-Mello 2018: 99); Puerto Francisco de Orellana [= El Coca] (Cupello and Vaz-de-Mello 2018: 99); Rodrigo Borja, IAMOE (Cupello and Vaz-de-Mello 2018: 99); Tiputini Biodiversity Station, 220 m (Cupello and Vaz-de-Mello 2018: 100).

###### Temporal data.

Collected in January, February, March, April, May, June, July, August, September, October, November, and December.

###### Remarks.

Inhabits the lowland evergreen forests and the foothill evergreen forests of the Amazon region from 200–1380 m a.s.l. Collected with pitfall traps baited with carrion and human feces.

#### Genus *Trichillidium* Vaz-de-Mello, 2008

*Trichillidium* Vaz-de-Mello, 2008: 44 (original description. Type species: *Pedaridiumquadridens* Arrow, 1913 by original combination).

*Trichillidium*: Vaz-de-Mello et al. 2011: 22 (characters in key); Krajcik 2012: 255 (complete list of species); Solís and Kohlmann 2012: 5 (list of species for Costa Rica); Boilly and Vaz-de-Mello 2013: 106 (characters in key); Chamorro et al. 2018: 74 (characters in key), 98 (list of species from Ecuador).

##### Trichillidium pilosum

Taxon classificationAnimaliaColeopteraScarabaeidae

(Robinson, 1948)

Plate 52C

Trichillum pilosum Robinson, 1948b: 149 (original description. Type locality: Panama, Barro Colorado Island, CANAL ZONE).
Pedaridium pilosum : Howden and Young 1981: 43 (cited as new combination, transferred to the genus Pedaridium Harold, 1868); Ferreira and Galileo 1993: 8 (characters in key), 34 (redescription); Barbero 2001: 7 (distribution, cited for Nicaragua); Medina et al. 2001: 139 (cited for Colombia); Ratcliffe 2002: 14 (cited for Panama).
Trichillidium pilosum : Vaz-de-Mello 2008: 46 (cited as new combination, distribution), 67 (figures of head and parameres); Solís and Kohlmann 2012: 5 (cited for Costa Rica); Krajcik 2012: 255 (complete list of species); Chamorro et al. 2018: 79 (figure 2G), 80 (figure 3F, G), 98 (cited for Ecuador).

###### Type specimens.

*Trichillumpilosum* Robinson, 1948. The holotype (sex unknown) is deposited at the USNM (see Vaz-de-Mello 2008: 46). Locality: Panama, Barro Colorado Island, not examined.

###### Distribution.

Colombia, Costa Rica, Ecuador, Nicaragua, and Panama.

###### Records examined.

ESMERALDAS: Estación Biológica Bilsa, 500 m (2 specimens MEPN). LOS RÍOS: Quevedo, Pichilingue (1 specimen CEMT). PICHINCHA: Bosque Potector Milpe-río Pachijal, 1200 m (1 specimen MUTPL); Estación Biológica la Hesperia (1 specimen MUTPL). SANTO DOMINGO DE LOS TSÁCHILAS: 16 km E Santo Domingo, Tinalandia, 680 m (1 specimen CEMT).

###### Literature records.

ESMERALDAS: La Chiquita, 5 m, 11 km SE San Lorenzo (Vaz-de-Mello 2008: 46). GUAYAS [= SANTA ELENA]: 27 km S Pto López, 76 km N Santa Elena (Vaz-de-Mello 2008: 46). LOS RÍOS: Quevedo, Pichilingue (Vaz-de-Mello 2008: 46); Quevedo (Vaz-de-Mello 2008: 46). MANABÍ: 73 km NE Chone, 90 km W Sto Domingo, 300 m (Vaz-de-Mello 2008: 46). PICHINCHA: 113 km NW Quito, en Puerto Quito Rd, 2600 m (Vaz-de-Mello 2008: 46); Pachijal Rd 104 km NW Quito (Vaz-de-Mello 2008: 46). PICHINCHA [= LOS RÍOS]: Rio Palenque Station, 230 m (Vaz-de-Mello 2008: 46); Río Palenque (Vaz-de-Mello 2008: 46). PICHINCHA [= DOMINGO DE LOS TSÁCHILAS]: 16 km E Sto Domingo, Tinalandia, 680 m (Vaz-de-Mello 2008: 46); 47 km S Sto Domingo (Vaz-de-Mello 2008: 46); 16 km SE Santo Domingo, Tinalandia, 680 m (Vaz-de-Mello 2008: 46).

###### Temporal data.

Collected in February, April, May, June, July, August, and September.

###### Remarks.

Inhabits coastal lowland evergreen forests and coastal evergreen foothill forests from 5–1200 m a.s.l. Collected with canopy fogging methods and pitfall traps baited with human feces.

#### Genus *Uroxys* Westwood, 1842

*Uroxys* Westwood, 1842: 59 (original description. Type species: *Uroxyscuprescens* Westwood, 1842 by monotypy).

*Uroxys*: Westwood 1843: 61 (redescription); Agassiz 1846: 1111 (catalog) Westwood 1847: 229 (redescription); Lacordaire 1856: 91 (redescription); Harold 1868b: 37 (redescription); Gemminger and Harold 1869: 1001 (catalog); Bates 1887: 43 (distribution); Gillet 1911a: 49 (catalog); Lucas 1920: 666 (catalog, distribution); Arrow 1933: 387 (list of species); Paulian 1938: 233 (characters in key); Pessôa and Lane 1941: 441 (diagnosis, written as *Uroxis* Westwood, 1842); Blackwelder 1944: 203 (catalog); Pereira 1954a: 56 (characters in key); Roze 1955: 43 (list of species from Venezuela); Halffter and Matthews 1966: 256 (catalog, distribution); Vulcano and Pereira 1967: 576 (characters in key); Howden and Young 1981: 13 (characters in key); 50 (redescription); Halffter and Edmonds 1982: 137 (catalog, distribution); Medina and Lopera 2000: 306 (characters in key); Vaz-de-Mello 2000: 195 (list of species from Brazil); Medina et al. 2001: 139 (list of species from Colombia); Ratcliffe 2002: 15 (list of species from Panama); Morón 2003: 56 (diagnosis); Hamel-Leigue et al. 2006: 12 (list of species from Bolívia); Vaz-de-Mello et al. 2011: 22 (characters in key); Carvajal et al. 2011: 132 (diagnosis), 318 (list of species from Ecuador); Krajcik 2012: 262 (complete list of species); Solís and Kohlmann 2012: 5 (list of species from Costa Rica); Boilly and Vaz-de-Mello 2013: 106 (characters in key); Solís and Kohlmann 2013: 290 (redescription); Chamorro et al. 2018: 74 (characters in key), 81, figs 4B, C, 98–99 (list of species from Ecuador).

*Pseuduroxys* Balthasar, 1938: 210 (original description. Type species: *Pseuduroxysohausi*Balthasar 1938); Blackwelder 1944: 204 (catalog, distribution); Halffter and Matthews 1966: 256 (catalog, distribution); Halffter and Edmonds 1982: 137 (catalog, distribution); Vaz-de-Mello et al. 2011: 3 (junior synonym of *Uroxys* Westwood, 1842); Carvajal et al. 2011: 135 (diagnosis), 318 (cited as genus *Pseudouroxys* Balthasar, 1938); Krajcik 2012: 230 (complete list of species); Solís and Kohlmann 2012: 5 (synonym of *Uroxys* Westwood, 1842).

##### Uroxys elongatus

Taxon classificationAnimaliaColeopteraScarabaeidae

Harold, 1868

Plate 52D

Uroxys elongatus Harold, 1868b: 44 (original description. Type locality: Quito).
Uroxys elongatus : Gemminger and Harold 1869: 1002 (catalog); Bates 1891: 24 (cited for Ecuador); Gillet 1911a: 49 (catalog); Campos 1921: 55 (cited for Ecuador); Arrow 1933: 388 (characters in key); Vulcano and Pereira 1967: 580 (characters in key); Medina et al. 2001: 139 (cited for Colombia); Carvajal et al. 2011: 318–319 (cited for Ecuador); Krajcik 2012: 262 (complete list of species); Ratcliffe et al. 2015: 196 (cited for Peru); Chamorro et al. 2018: 79 (figure 2C), 98 (cited for Ecuador).
Uroxys elongata : Blackwelder 1944: 203 (list of species from Latin America).

###### Type specimens.

*Uroxyselongatus* Harold, 1868. Five syntypes examined deposited in MNHN (ex coll. HW Bates and ex coll. R Oberthur). Lectotype to be designated in a future work on this species group.

**Syntype** (♂): “Quito [hw]”, “Ex-Musæo / H. W. BATES / 1892 [p, black margin]”, “Uroxys / elongatus / ♀ C. Hft III. Harold / typ. [hw]”, “MUSÉUM PARIS / 1952 / COLL. R. OBERTHUR [p, green label, black margin]”.

**Syntype** (♂): “Quito [hw]”, “Ex-Musæo / H. W. BATES / 1892 [p, black margin]”, “Uroxys / elongatus / ♀ Harold [hw]”, “MUSÉUM PARIS / 1952 / COLL. R. OBERTHUR [p, green label, black margin]”.

**Syntype** (♂): “Quito [hw]”, “Ex-Musæo / H. W. BATES / 1892 [p, black margin]”, “MUSÉUM PARIS / 1952 / COLL. R. OBERTHUR [p, green label, black margin]”.

**Syntype** (♀): “Cotacachi, / Ecuador. / 11–13500 feet. / Ed. Whymper [p and hw]”, “Uroxys / elongatus / Harold [hw]”, “MUSÉUM PARIS / 1952 / COLL. R. OBERTHUR [p, green label, black margin]”.

**Syntype** (♀): “Cotacachi, / Ecuador. / 11–13500 feet. / Ed. Whymper [p and hw]”, “MUSÉUM PARIS / 1952 / COLL. R. OBERTHUR [p, green label, black margin]”.

###### Distribution.

Colombia and Ecuador.

###### Records examined.

CARCHI: 10 km W de Tufiño, 3600 m (3 specimens CEMT); 15 km SW de Tulcán (2 specimens CEMT); km 3 road Tufiño-Maldonado, 3400 m (1 specimen CEMT); Montufar Los Encinos, 3450 m (2 specimens CEMT); Tulcán ciudadela del maestro, 2950 m (3 specimens MUTPL). IMBABURA: Cotacachi (2 specimens MNHN). PICHINCHA: Quito (3 specimens MNHN).

###### Temporal data.

Collected in April, July, and October.

###### Remarks.

Inhabits the evergreen high montane forests of the Andean region from 2950–3350 m a.s.l. Collected manually in cow dung and with pitfall trap baited with pig feces.

##### Uroxys frankenbergeri

Taxon classificationAnimaliaColeopteraScarabaeidae

Balthasar, 1940

Plate 53A

Uroxys frankenbergeri Balthasar, 1940: 35 (original description. Type locality: Loja).
Uroxys frankenbergeri : Blackwelder 1944: 203 (list of species for Latin America); Vulcano and Pereira 1967: 579 (characters in key); Carvajal et al. 2011: 318–319 (cited for Ecuador); Bezdek and Hajek 2011: 358 (catalog of types NMPC); Krajcik 2012: 262 (complete list of species); Chamorro et al. 2018: 98 (cited for Ecuador).

###### Type specimens.

*Uroxysfrankenbergeri* Balthasar, 1940. The holotype (♀) is deposited at the NMPC (ex coll. V. Balthasar). Locality: Cajanuma, examined.

**Holotype** (♀): “S ECUADOR / Cajanuma / Ohaus S. [p]”, “Loja Cajanuma / F. Ohs. 25.8.05 [p]”, “Arrow determ. / Uroxys / sp. [p and hw]”, “Typus [p, red label, black margin]”, “Uroxys / frankenbergeri / n. sp. / Dr. V. Balthasar det. [p and hw]”, “frankenbergeri / m. [hw, green label, black margin]”, “Mus. Nat. Pragae / 65709 / Inv. [hw and p, red label]”.

###### Distribution.

Only known from Ecuador.

###### Records examined.

LOJA: Loja, Cajanuma (1 specimen NMPC).

###### Temporal data.

Collected in September

###### Remarks.

This species is thought to occur in the evergreen high montane forests of the Andean region. The collection method is unknown.

##### Uroxys gorgon

Taxon classificationAnimaliaColeopteraScarabaeidae

Arrow, 1933

Plate 53B

Uroxys gorgon Arrow, 1933: 397 (original description. Type locality: COLOMBIA: Gorgona I).
Uroxys gorgon : Blackwelder 1944: 203 (list of species of Latin America); Contreras 1951: 222 (cited for Colombia); Vulcano and Pereira 1967: 581 (characters in key); Bacchus 1978: 103 (catalogue of the types of the species described by Arrow); Howden and Young 1981: 51 (characters in key); 53 (redescription); Medina et al. 2001: 139 (cited for Colombia); Ratcliffe 2002: 15 (cited for Panama); Carvajal et al. 2011: 318–319 (cited for Ecuador); Krajcik 2012: 262 (complete list of species); Solís and Kohlmann 2012: 5 (cited for Costa Rica); Solís and Kohlmann 2013: 291 (characters in key), 307 (redescription); Chamorro et al. 2018: 98 (cited for Ecuador).

###### Type specimens.

*Uroxysgorgon* Arrow, 1933. The lectotype (♂) and nine paralectotypes are deposited in NHML (see Bacchus 1978: 103). Locality: Colombia: Gorgona Island, not examined.

###### Distribution.

Colombia, Costa Rica, Ecuador, and Panama

###### Records examined.

MANABÍ: Pedernales, 100 m (1 specimen MQCAZ).

###### Temporal data.

Collected in November.

###### Remarks.

Inhabits lowland evergreen forests at 100 m a.s.l. This species was found in the fur of a three-toed sloth.

##### Uroxys latesulcatus

Taxon classificationAnimaliaColeopteraScarabaeidae

Bates, 1891

Plates 53C , 58A–B

Uroxys latesulcatus Bates, 1891: 24 (original description. Type locality: Ecuador: Pichincha, 12,000 feet [= 3655 m]; Machachi, 9–10,000 feet [= 2745–3050 m]).
Uroxys latesulcatus : Gillet 1911a: 50 (catalog); Campos 1921: 55 (cited for Ecuador); Arrow 1933: 388 (characters in key); Vulcano and Pereira 1967: 580 (characters in key); Carvajal et al. 2011: 318–319 (cited for Ecuador); Krajcik 2012: 262 (complete list of species); Chamorro et al. 2018: 98 (cited for Ecuador).
Uroxys latesulcata : Blackwelder 1944: 203 (list of species for Latin America).
Uroxys magnus Balthasar, 1940: 37 (original description); Vulcano and Pereira 1967: 580 (characters in key); Carvajal et al. 2011: 318–319 (cited for Ecuador); Bezdek and Hajek 2011: 359 (catalog of types NMPC); Krajcik 2012: 262 (complete list of species, cited as Uroxysmagnus Balthasar, 1947), Chamorro et al. 2018: 98 (cited for Ecuador), **syn. n.**

###### Type specimens.

*Uroxyslatesulcatus* Bates, 1891. The lectotype (♂) (here designated) and two paralectotypes are deposited at the MNHN (ex coll. HW Bates and ex coll. R Oberthur). Locality: Ecuador (examined). One paralectotype is deposited at the NHML, examined.

**Lectotype** (**here designated**) (♂): “Ecuador. / feet. / Ed. Whymper. [p]”, “Uroxys / latesulcatus / Bates [p, black margin]”, “Ex-Musæo / H. W. BATES / 1892 [p, black margin]”, “MUSÉUM PARIS / 1952 / COLL. R. OBERTHUR [p, green label, black margin]”, “LECTOTYPE ♂ / Uroxys / latesulcatus / Bates / des. F.Z. Vaz-de-Mello. 2014 [p and hw, red label, black margin]”.

**Paralectotype** (♂): “Ecuador. / feet. / Ed. Whymper. [p]”, “Uroxys / latesulcatus / Bates [p, black margin]”, “Ex-Musæo / H. W. BATES / 1892 [p, black margin]”, “MUSÉUM PARIS / 1952 / COLL. R. OBERTHUR [p, green label, black margin]”, “PARALECTOTYPE / Uroxys ♂ / latesulcatus / Bates / des. F.Z. Vaz-de-Mello, 2014 [hw and p, yellow label, black margin]”.

**Paralectotype** (♂): “Ecuador. / feet. / Ed. Whymper. [p]”, “latesulcatus / Bates [p]”, “Ex-Musæo / H. W. BATES / 1892 [p, black margin]”, “MUSÉUM PARIS / 1952 / COLL. R. OBERTHUR [p, green label, black margin]”, “PARALECTOTYPE / Uroxys ♂ / latesulcatus / Bates / des. F.Z. Vaz-de-Mello, 2014 [hw and p, yellow label, black margin]”.

**Paralectotype** (♂): “Uroxys / latesulcatus, / (Type) Bates [hw]”, “92-24 [p]”, “SYN- / TYPE [p, blue label]”, “PARALECTOTYPE / Uroxys ♂ / latesulcatus / Bates / des. F.Z. Vaz-de-Mello, 2014 [hw and p, yellow label, black margin]”.

*Uroxysmagnus* Balthasar, 1940. The lectotype (♂) (here designated) is deposited at the NMPC (ex coll. V Balthasar). Locality: Ecuador (examined).

**Lectotype** (**here designated**) (♂): “Ecuador / D. Stübel / 5351 [hw, green label]”, “4684 [hw, red letters]”, “Typus [p, red label]”, “Uroxys / magnus / n. sp. / DR. BALTHASAR. DET. [p]”, “magnus / m. [hw, green label]”, “Mus. Nat. Pragae / 65711 / Inv. [p and hw, red label]”, “LECTOTYPE ♂ / Uroxys / magnus / Balth / des. F.Z. Vaz-de-Mello. 2013 [p and hw, red label, black margin]”.

###### Distribution.

Only known from Ecuador.

###### Records examined.

BOLIVAR: Cashca Totoras (5 specimens MQCAZ). COTOPAXI: Sigchos (2 specimens MQCAZ). IMBABURA: Selva Alegre (3 specimens MQCAZ). PICHINCHA: Atahualpa, Bosque Protector Piganta, 2880 m (2 specimens MUTPL); Cochasquí, 3100 m (56 specimens CEMT); Chiriboga (5 specimens MQCAZ); EL Chalpar, 3300 m (1 specimen CEMT, 3 specimens MQCAZ); Machachi, 2700 m (21 specimens MQCAZ); La Cocha (1 specimen CEMT; 8 specimens MQCAZ); Nono (8 specimens MQCAZ); Palmeras, 2200 m (5 specimens MQCAZ); Pasochoa, 3500 m (1 specimen CEMT; 8 specimens MQCAZ); Pingtag, 2880 m; Quito, Cumbayá, 2340 m (13 specimens MQCAZ); Reserva Ecológica Los Illinizas (2 specimens MUTPL); San José de Minas (3 specimens CEMT; 4 specimens MQCAZ); Yanacocha (2 specimens MQCAZ). UNDETERMINED PROVINCE: without specific locality (3 specimens MNHN; 1 specimen NHML, 1 specimen NNPC).

###### Literature records.

PICHINCHA: without specific locality 12.000 feet [= 3655 m] (Bates 1891: 24); Machachi 9–10000 feet [= 3045 m] (Bates 1891: 24).

###### Temporal data.

Collected in January, February, March, April, May, June, October, November, and December.

###### Remarks.

Inhabits the high montane evergreen forests of the Andean region from 2340–3300 m a.s.l. Collected with pitfall trap baited with pig or cow feces.

Balthasar in 1940, described *Uroxysmagnus* for Ecuador (without type locality) as a different species from *Uroxyslatesulcatus* Bates, 1891 (recorded for Ecuador, Pichincha at 12,000 feet [= Pichincha volcano] and Machachi 9 at 10,000 feet, as type localities). However, upon examining the external and genital morphology of the type specimens, *U.latesulcatus* (lectotype ♂ here designated, deposited at the MNHN, Plate 58A) and *U.magnus* (lectotype ♂ here designated, deposited at the NMPC, Plate 58B), we believe that they belong to the same species due to similar characteristics (specifically, the shape of the head, dorsal colouration, body length and aedeagus). Therefore, we propose that *U.magnus* is a synonym of *U.latesulcatus* (see ICZN 1999, Article 23). Two lectotypes (without specific localities for Ecuador) are here designated and illustrated (♂ Plate 58A, B).

##### Uroxys lojanus

Taxon classificationAnimaliaColeopteraScarabaeidae

Arrow, 1933

Plate 53D

Uroxys lojanus Arrow, 1933: 395 (original description. Type locality: ECUADOR: Loja Pucara, Loja Calvario, 6600–6900 feet [= 2010–2100 m]).
Uroxys lojanus : Vulcano and Pereira 1967: 580 (characters in key); Bacchus 1978: 105 (catalogue of the types of the species described by Arrow); Carvajal et al. 2011: 318–319 (cited for Ecuador); Krajcik 2012: 262 (complete list of species); Chamorro et al. 2018: 98 (cited for Ecuador).
Uroxys lojana : Blackwelder 1944: 203 (list of species of Latin America).

###### Type specimens.

*Uroxyslojanus* Arrow, 1933. The lectotype is deposited at the NHML. Locality: Ecuador, Loja, Pucara, examined.

**Lectotype** (♂): “Loja Pucara / F. Ohs. 8.8.05 [p]”, “Uroxys / lojanus / type arrw [hw]”, “F. Ohaus / 1907. 117 [hw]”, “LECTO- / TYPE [p, violet label]”, “Uroxys ♂ / lojanus arrow / M. E. Bacchus det 1975. / LECTOTYPE [p and hw]”.

###### Distribution.

Only known from Ecuador.

###### Records examined.

LOJA: Pucara (1 specimen BMNB)

###### Literature records.

LOJA: Clavario [= El Calvario] (Bacchus 1978: 105); Loja Calvario, 6600–6900 feet [= 2010–2100 m] (Arrow 1933: 395).

###### Temporal data.

It is not known when this species was collected.

###### Remarks.

Inhabits the montane cloud forests of the Andean region at 2100 m a.s.l. The collection method is unknown.

##### Uroxys monstruosus

Taxon classificationAnimaliaColeopteraScarabaeidae

Balthasar, 1940

Plate 54A

Uroxys monstruosus Balthasar, 1940: 34 (original description. Type locality: Ecuador).
Uroxys monstruosus : Vulcano and Pereira 1967: 580 (characters in key); Carvajal et al. 2011: 318–319 (cited for Ecuador); Bezdek and Hajek 2011: 359 (catalog of types NMPC); Krajcik 2012: 262 (complete list of species); Chamorro et al. 2018: 98 (cited for Ecuador).

###### Type specimens.

*Uroxysmonstruosus* Balthasar, 1940. The holotype is deposited at the NMPC. Locality: Ecuador, without specific locality, examined.

**Holotype** (sex unknown): “Ecuador / Baron [p]”, “Typus [p, red label, black margin]”, “Uroxys / monstrosus / n. sp. m. / Dr. V. Balthasar det. [p and hw]”, “monstrosus m. [hw, green label, black margin]”, “HOLOTYPE [hw, red label]”.

###### Distribution.

Only known from Ecuador.

###### Records examined.

BOLIVAR: Cashca Totoras (4 specimens MQCAZ). CAÑAR: La Carbonería, 2850 m (7 specimens CEMT). UNDETERMINED PROVINCE: without specific locality (1 specimen NMPC).

###### Temporal data.

Collected in January and December.

###### Remarks.

Inhabits the high montane forests of the Andean region at 2850 m a.s.l. The collection method is unknown.

##### Uroxys ohausi

Taxon classificationAnimaliaColeopteraScarabaeidae

(Balthasar, 1938)

Plate 54B

Pseuduroxys ohausi Balthasar, 1938: 211 (original description. Type locality: Ecuador: Loja).
Pseuduroxys ohausi : Blackwelder 1944: 204 (list of species from Latin America); Carvajal et al. 2011: 318–319 (cited for Ecuador); Bezdek and Hajek 2011: 356 (catalog of the types of the NMPC); Krajcik 2012: 230 (complete list of species).
Uroxys ohausi : Chamorro et al. 2018: 98 (cited for Ecuador).

###### Type specimens.

*Pseuduroxysohausi* Balthasar, 1938. Four syntypes examined deposited at the MSMF and NMPC (ex coll. V Balthasar). Lectotype to be designated in a future work on this species group.

**Syntype** (♂): “ECUADOR / Loja / Ohaus S. [p]”, “Loja Villonaco / F. Ohs. 31.8.05 [p]”, “genotyp ! / Pseuduroxys / ohausi / n. sp / Dr. V. Balthasar. det. [p and hw]”, “Senckenberg- / Museum / Frankfurt / Main [p]”, “Typus [p, red label, black margin]”.

**Syntype** (♂): “ECUADOR / Loja / Ohaus S. [p]”, “Loja Villonaco / F. Ohs. 5.9.05 [p]”, “Typus [p, red label, black margin]”, “Pseuduroxys / ohausi n.sp. / Dr. V. Balthasar det. [p and hw]”, “Ohausi / m [hw, green label]”, “Mus. Nat. Pragae / 65705 / Inv. [p and hw, red label]”, “PRAGUE MUSEUM / LOANED: X. 2008 / D.J.MANN [p, blue label]”.

**Syntype** (♂): “ECUADOR / Loja / Ohaus S. [p]”, “Loja Villonaco / F. Ohs. 5.9.05 [p]”, “Typus [p, red label, black margin]”, “tp:taxon-name-part taxon-name-part-type="genus" full-name="Pseuduroxys">Pseuduroxys / ohausi n.sp. / Dr. V. Balthasar det. [p and hw]”, “Box 82 / det. D.J. Mann. 2008 [p, blue label]”.

**Syntype** (♀): “‘ECUADOR / Loja / Ohaus S. [p]”, “Loja Villonaco / F. Ohs. 5.9.05 [p]”, “Typus [p, red label, black margin]”, “Pseuduroxys / ohausi n.sp. / Dr. V. Balthasar det. [p and hw]”, “Mus. Nat. Pragae / 65705 / Inv. [p and hw, red label]”, “PRAGUE MUSEUM / LOANED: X. 2008 / D.J.MANN [p, blue label]”.

###### Distribution.

Only known from Ecuador.

###### Records examined.

LOJA: Loja, Villonaco (2 specimens CEMT; 3 specimens MNPC; 1 specimen MSMF).

###### Temporal data.

Collected in August and September.

###### Remarks.

This species may be distributed in the montane cloud forests and/or high montane evergreen forests of the Andean region. The collection method is unknown.

##### Uroxys pauliani

Taxon classificationAnimaliaColeopteraScarabaeidae

Balthasar, 1940

Plate 54C

Uroxys pauliani Balthasar, 1940: 34 (original description. Type locality: Columbia, Pichinde).
Uroxys pauliani : Medina et al. 2001: 139 (cited for Colombia); Carvajal et al. 2011: 318–319 (cited for Ecuador); Bezdek and Hajek 2011: 359 (catalog of the types of the NMPC); Krajcik 2012: 262 (complete list of species); Solís and Kohlmann 2012: 5 (cited for Costa Rica); Solís and Kohlmann 2013: 291 (characters in key), 326 (redescription); Chamorro et al. 2018: 99 (cited for Ecuador).
Uroxys depressifrons Howden & Young, 1981: 55 (original description); Medina et al. 2001: 139 (cited for Colombia); Ratcliffe 2002: 15 (cited for Panama); Krajcik 2012: 262 (complete list of species); Solís and Kohlmann 2012: 5 (synonym of Uroxyspauliani Balthasar, 1940); Solís and Kohlmann 2013: 326 (cited as synonym of Uroxyspauliani Balthasar, 1940).

###### Type specimens.

*Uroxyspauliani* Balthasar, 1940. Three syntypes are deposited at the SMTD and NMPC. (ex coll. V Balthasar). Lectotype to be designated in a future work on this species group.

*Uroxysdepressifrons* Howden & Young, 1981. The holotype (♂) is deposited at the CMNC (ex coll. H Howden) (see Howden and Young 1981: 56). Locality: Panama, Chiriqui Prov., 15 km NW Hato dl Volcan (not examined).

###### Distribution.

Colombia, Costa Rica, Ecuador, and Panama

###### Records examined.

COTOPAXI: Bosque Integral Otonga, 2000 m (16 specimens CEMT). IMBABURA: Lita, 500 m (1 specimen CEMT).

###### Temporal data.

Collected in March and September.

###### Remarks.

Inhabits coastal evergreen foothill forests at 500 m a.s.l. In the Andean region, it was registered in the montane cloud forests at 2000 m a.s.l. Collected with pitfall traps baited with human feces.

##### Uroxys rugatus

Taxon classificationAnimaliaColeopteraScarabaeidae

Boucumont, 1928

Plate 54D

Uroxys rugatus Boucumont, 1928a: 188 (original description. Type locality: Uruguay).
Uroxys rugatus : Arrow 1933: 388 (characters in key, redescription), 389 (comment); Vulcano and Pereira 1967: 581 (characters in key); Carvajal et al. 2011: 318–319 (cited for Ecuador); Krajcik 2012: 262 (complete list of species); Chamorro et al. 2018: 99 (cited for Ecuador).
Uroxys rugata : Blackwelder 1944: 204 (list of species from Latin America).

###### Type specimens.

*Uroxysrugatus* Boucumont, 1928. Two syntypes examined deposited at the MNHN. Lectotype to be designated in a future work on this species group.

###### Distribution.

Ecuador and Peru.

###### Records examined.

LOJA: Amaluza, Angashcola, 2740 m (2 specimens CEMT); Loja (1 specimen CEMT); Cerro Villonaco, 2740 (2 specimens MUTPL); Cariamanga (2 specimens MQCAZ); Loja, 2600 m (1 specimen MQCAZ). ZAMORA CHINCHIPE: 15 km S de Jimbura, 3000 m (3 specimens CEMT).

###### Temporal data.

Collected in March and December.

###### Remarks.

Inhabits the evergreen high montane forests of the Andean region from 2600–3000 m a.s.l. Collected with pitfall traps baited with human feces. Arrow (1933) suggested that the type locality of this species, Uruguay, is erroneous. We did not find any other specimen of *U.rugatus* collected in Uruguay in the scientific collections from that country.

##### Uroxys spaethi

Taxon classificationAnimaliaColeopteraScarabaeidae

Balthasar, 1940

Plate 55A

Uroxys späthi Balthasar, 1940: 37 (original description. Type locality: Ecuador, Santa Inez [= Santa Inés]).
Uroxys späthi : Vulcano and Pereira 1967: 580 (characters in key); Carvajal et al. 2011: 318–319 (cited for Ecuador).
Uroxys spaethi : Bezdek and Hajek 2011: 360 (catalog of the types of the NMPC); Krajcik 2012: 262 (complete list of species); Chamorro et al. 2018: 99 (cited for Ecuador).

###### Type specimens.

*Uroxysspäthi* Balthasar, 1940. The holotype (♀) is deposited at the NMPC (ex coll. V Baltashar). Locality: Ecuador, Santa Jnéz [= Santa Inés], examined.

**Holotype** (♀): “Santa Jnéz / (Ecuad.) / R. Haensch S. [p]”, “Typus [p, red label]”, “U. Spaethi / n.sp. / Dr. V. Balthasar det. [p and hw]”, “spaethi / m. [hw, green label]”, “HOLOTYPE [hw, red label]”.

###### Distribution.

Only known from Ecuador.

###### Records examined.

TUNGURAHUA: Baños, El Topo, 1590 m (17 specimens CEMT). UNDETERMINED PROVINCE: Santa Jnez [= Santa Inés] (1 specimen NMPC).

###### Temporal data.

Collected in January.

###### Remarks.

Inhabits the evergreen lower montane forests of the Andean region at 1590 m a.s.l. Collected with pitfall traps baited with human feces.

##### Uroxys sulcicollis

Taxon classificationAnimaliaColeopteraScarabaeidae

Harold, 1880

Plate 55B

Uroxys sulcicollis Harold, 1880a: 18 (original description. Type locality: Fusagasugá).
Uroxys sulcicollis : Gillet 1911a: 50 (catalog); Arrow 1933: 387 (characters in key); Blackwelder 1944: 204 (list of species from Latin America); Contreras 1951: 222 (cited for Colombia); Vulcano and Pereira 1967: 579 (characters in key); Krajcik 2012: 262 (complete list of species); Chamorro et al. 2018: 99 (cited for Ecuador).

###### Type specimens.

*Uroxyssulcicollis* Harold, 1880. Four syntypes examined deposited in NMPC. Lectotype to be designated in a future work on this species group.

###### Distribution.

Colombia and Ecuador.

###### Records examined.

CAÑAR: La Carboneira, 2850 m (5 specimens CEMT; 4 specimens MQCAZ).

###### Temporal data.

Collected in January and December

###### Remarks.

Inhabits the high montane forests of the Andean region at 2850 m a.s.l. The collection method is unknown.

##### Uroxys sulai

Taxon classificationAnimaliaColeopteraScarabaeidae

Balthasar, 1940

Plate 55C

Uroxys sulai Balthasar, 1940: 33 (original description. Type locality: Ecuador, Prov. Guayaz, Guayaquil).
Uroxys sulai : Vulcano and Pereira 1967: 579 (characters in key); Carvajal et al. 2011: 318–319 (cited for Ecuador); Bezdek and Hajek 2011: 360 (catalog of the types of the NMPC); Krajcik 2012: 262 (complete list of species); Chamorro et al. 2018: 99 (cited for Ecuador).

###### Type specimens.

*Uroxyssulai* Balthasar, 1940. The holotype (♂) is deposited at the NMPC (ex coll. V Baltashar). Locality: Guayaquil, examined.

**Holotype** (♂): “Guayaquil / F. Ohs. S. 18. 6. 05 [p]”, “Typus [p, red label, black margin]”, “Uroxys / Šulai m. / Typus! N. sp. / Dr. V. Balthasar det. [p and hw]”, “Šulai m. [hw, green label]”, “Mus. Nat. Pragae / 65714 / Inv [p and hw, red label]”, “HOLOTYPE [hw, red label]”.

###### Distribution.

Only known from Ecuador.

###### Records examined.

GUAYAS: Guayaquil (1 specimen NMPC).

###### Temporal data.

Collected in June

###### Remarks.

Inhabits coastal lowland evergreen forests at 50 m a.s.l. The collection method is unknown.

### Species erroneously recorded from Ecuador

#### Canthon (Canthon) cyanellussallei

Taxon classificationAnimaliaColeopteraScarabaeidae

Harold, 1863

Canthon Sallei Harold, 1863: 174 (original description. Type locality: Nicaragua).

##### Remarks.

This species was recorded from Ecuador by the following authors: Howden and Young (1981: 27) cited as *CanthoncyanellusSallei* Harold; Barbero (2001: 2) cited as ssp. *Sallei* (Harold, 1863); Solís and Kohlmann (2002: 11) cited as *Canthoncyanellus* LeConte); Carvajal et al. (2011: 314–315) list of species.

Nolasco-Soto et al. (2017: 181) mentioned this subspecies was recorded in Guatemala, Honduras, Costa Rica, Panama, Colombia, Peru, Nicaragua, and Mexico. According to our data, there are no other records of this species in the collections listed.

#### Canthon (Canthon) lituratus

Taxon classificationAnimaliaColeopteraScarabaeidae

(Germar, 1813)

Ateuchus lituratus (Germar, 1813): 117 (original description. Type locality: Brasilien [=Brazil]).

##### Remarks.

This species was recorded for Ecuador by the following authors: Guérin-Méneville (1855: 587) cited as *C.quadripustulatum* original description, distribution: Napo-Amazon region; Gillet (1911a: 31) cited as *Canthonlituratusquadripustulatus* Guér); Blackwelder (1944: 200) cited as *Canthonlituratum* v. *quadripustulatum*; Solís and Kohlmann (2002: 30) cited as *Canthonlituratus* (Germar); Carvajal et al. (2011: 314–315) list of species.

It is possible that Guérin-Méneville (1855) described a different species that was mistaken for *C.lituratus* (Germar, 1813). According to our data, there are no other records of this species in the collections listed.

#### Canthon (Canthon) morsei

Taxon classificationAnimaliaColeopteraScarabaeidae

Howden, 1966

Canthon (Glaphyrocanthon?) morsei Howden, 1966: 728 (original description. Type locality: Fortin, Veracruz, Mexico).

##### Remarks.

This species was recorded for Ecuador by the following authors: Howden and Young (1981: 29) cited as *Canthonmorsei* group; Solís and Kohlmann (2002: 39) cited as *Canthonmorsei* Howden; Carvajal et al. 2011: (314–315) list of species. According to our data, there are no other records of this species in the collections listed.

#### Canthon (Canthon) mutabilis

Taxon classificationAnimaliaColeopteraScarabaeidae

Lucas, 1857

Canthon mutabile Lucas, 1857: 100 (original description. Type locality: Pebas, Haute-Amazone [= Upper Amazon].

##### Remarks.

This species was recorded for Ecuador by the following authors: Campos (1921: 55) in the localities of: Naranjito, San Rafael, Bucay, Chimbo, Balzapamba, Posorja, El Morro, Chanduy and Naranjal; Carvajal et al. (2011: 314–315) list of species. According to our data, there are no other records of this species in the collections listed.

#### Canthon (Glaphyrocanthon) rubrescens

Taxon classificationAnimaliaColeopteraScarabaeidae

Blanchard, 1843

Canthon rubrescens Blanchard, 1843: 167 (original description. Type locality: province de Chiquitos-Guarayos).

##### Remarks.

This species was recorded for Ecuador by the following authors: Pereira and Martínez (1956a: 170) cited as *Geocanthonrubrescens* (Blanchard) n. com, cited for Chimborazo; Vulcano and Pereira (1964: 673) cited as *Geocanthonrubrescens* (Blanchard); Vulcano and Pereira (1967: 550) cited as *Geocanthonrubrescens* (Blanchard, 1843); Carvajal et al. (2011: 314–315) list of species.

It is possible that Pereira and Martínez (1956) refer to a different species that was mistaken for *C.rubrescens* Blanchard, 1843. According to our data, there are no other records of this species in the collections listed.

#### Canthon (Goniocanthon) smaragdulussmaragdulus

Taxon classificationAnimaliaColeopteraScarabaeidae

Fabricius, 1781

Scarabaeus smaragdulus Fabricius, 1781: 34 (original description. Type locality: America meridionali [= South America]).

##### Remarks.

This species was recorded for Ecuador by the following authors: Blackwelder (1944: 200) cited as *Canthonspeculifer* Lap, current synonym of Canthon (Goniocanthon) smaragdulussmaragdulus Fabricius, 1781; Carvajal et al. (2011: 314–315) quoting Blackwelder (1944) as reference.

Nunes et al. (2018: 9) mentioned this subspecies was recorded in Argentina, Brazil, and Paraguay. According to our data, there are no other records of this species in the collections listed.

#### Copris (Copris) incertus

Taxon classificationAnimaliaColeopteraScarabaeidae

Say, 1835

Copris incérta Say, 1835: 175 (original description. Type locality: Mexico).

##### Remarks.

This species was recorded for Ecuador by the following authors: Blackwelder (1944: 208) list of species for Latin America; Pereira and D’Andretta (1955b: 261) in the localities of: Pucay [= Bucay], Balzapamba, Ana María [= Hacienda Ana María, Quevedo], Sigiro, Arenal, Capilla Zaruma, and Galapagos; Matthews (1961: 44) in the localities of: Guayas-Naranjal, Los Ríos-location undetermined, Paramba, Lita, and San Rafael; Carvajal et al. (2011: 320–321) list of species; Chamorro et al. (2018: 93) list of species.

Darling and Génier (2018: 19) mentioned this species was distributed in Mexico, has been subsequently introduced to Hawaii, New Zealand, New Caledonia, Solomon Island, Vanuatu, and Fiji. According to our data, there are no other records of this species in the collections listed.

#### Copris (Copris) lugubris

Taxon classificationAnimaliaColeopteraScarabaeidae

Boheman, 1858

Copris lugubris Boheman, 1858: 42 (original description. Type locality: Insulæ Gallapagos [= Galápagos Islands, Ecuador]).

##### Remarks.

This species was recorded for Ecuador by the following authors: Gillet (1911a: 75) cited for Galapagos-Insel; Blackwelder (1944: 208) cited as Is. Galápagos; Carvajal et al. (2011: 320–321) list of species; Krajcik (2012: 79) cited for Galapagos Islands.

Peck (2005: 81) mentioned this species as an erroneous record for the Galapagos Islands. The reports by Boheman (1858) are probably erroneous with regard to their type localities (see Bousquet 2016, Cupello 2018).

#### Deltochilum (Deltochilum) tumidum

Taxon classificationAnimaliaColeopteraScarabaeidae

Howden, 1966

Deltochilum (Deltochilum) tumidum Howden, 1966: 738 (original description. Type locality: Mexico, N. Mazatlan, Sinaloa).

##### Remarks.

This species was recorded from Ecuador by the following authors: Campos (1921: 55) cited as *Deltochilumtumidus* Gillet [= doubtful description], in the localities of Bucay and Chimbo; Carvajal et al. (2011: 316–317) quoting Campos (1921) as reference).

Gillet never described *Deltochilumtumidus*. Subsequently Génier (2012: 34) mentioned this species is recorded only in Mexico. According to our data, there are no other records of this species in the collections listed.

#### Deltochilum (Deltohyboma) femorale

Taxon classificationAnimaliaColeopteraScarabaeidae

Bates, 1870

Deltochilum femorale Bates, 1870: 178 (original description. Type locality: Amazons).

##### Remarks.

Carvajal et al. (2011: 316–317) erroneously quotes Howden and Young (1981) as a reference for Ecuador. According to our data, there are no other records of this species in the collections listed.

#### Deltochilum (Deltohyboma) parile

Taxon classificationAnimaliaColeopteraScarabaeidae

Bates, 1887

Deltochilum parile Bates, 1887: 35 (original description. Type locality: Mexico, Santecomapan; Panama, Volcan de Chiriqui).

##### Remarks.

This species was recorded from Ecuador by the following authors: Howden and Young (1981: 38) provide a distribution; Carvajal et al. (2011: 316–317) give a list of species. According to our data, there are no other records of this species in the collections listed.

#### Deltochilum (Deltohyboma) spinipes

Taxon classificationAnimaliaColeopteraScarabaeidae

Paulian, 1938

Deltochilum (Deltochilum) spinipes Paulian, 1938: 280 (original description. Type locality: Colombie [= Colombia] Santa Fé de Bogota, Muzo, Antioquia, Fiasagusuga = Fusagasugá]. Équator [= Ecuador]: environs d’Ambato [= around Ambato], Macas).

##### Remarks.

This species was recorded for Ecuador by the following authors: Vulcano and Pereira (1964: 659, list of species); Vulcano and Pereira (1967: 557, list of species); Carvajal et al. (2011: 316–317; list of species); Krajcik (2012: 88, list of species).

Silva and Vaz-de-Mello (2014: 281–283) explained that the type (holotype) and cotypes (paratypes) designated by Paulian (1938) are significantly different from the specimens known from Ecuador. Specifically, Ecuadorian specimens are dull blue in color and have longer bodies compared to those described by Paulian, which were bright green in coloration and smaller in size. It is possible that they belong to different species within this group. According to Silva and Vaz-de-Mello (2014), the type series of *D.spinipes* exhibit variations (especially in body length and coloration), suggesting that *D.spinipes* might represent a complex of species.

#### Dichotomius (Dichotomius) alyattes

Taxon classificationAnimaliaColeopteraScarabaeidae

Harold, 1880

Dichotomius alyattes Harold, 1880: 24 (original description. Type locality: von Ibagué, S. Rosa und Abejorrál [= of Ibagué, Santa Rosa, and Abejorral]; die männchen von Aguada [= ♂ of Aguadas] dann zwischen Manizales und Salamina [= between Manizales and Salamina]).

##### Remarks.

This species was recorded for Ecuador by the following authors: Gillet (1911a: 59) cited as *PinotusAlyattes* Har; Campos (1921: 56) in the localities of Bucay and Chimbo; Luederwaldt (1929: 35) cited as *PinotusAlyattes* Har; Blackwelder (1944: 206) cited as *Pinotusacuminiger* Kirsch, a synonym of *Pinotusalyattes* Har; Carvajal et al. (2011: 320–321), list of species; Krajcik (2012: 91), list of species. According to our data, there are no other records of this species in the collections listed.

#### Dichotomius (Dichotomius) horridus

Taxon classificationAnimaliaColeopteraScarabaeidae

Felsche, 1911

Pinotus horridus Felsche, 1911: 136 (original description. Type locality: Cayenne).

##### Remarks.

This species was recorded for Ecuador by the following authors: Luederwaldt (1929: 24), Bucay locality; Blackwelder (1944: 207) cited as *Pinotushorridus* Fels; Pereira (1954b: 464), characters in key; Vulcano and Pereira (1967: 584), characters in key; Carvajal et al. (2011: 320–321), list of species. According to our data, there are no other records of this species in the collections listed.

#### Dichotomius (Dichotomius) longiceps

Taxon classificationAnimaliaColeopteraScarabaeidae

(Taschenberg, 1870)

Copris longiceps Taschenberg, 1870: 180 (original description. Type locality: Loja).

##### Remarks.

This species was recorded for Ecuador by the following authors: Gillet (1911a: 61) cited as *Pinotuslongiceps* Taschb; Luederwaldt (1929: 18), distribution; Blackwelder (1944: 207) cited as *Pinotuslongiceps* Tasch; Carvajal et al. (2011: 320–321), list of species; Krajcik (2012: 91), list of species; Boilly (2015b: 83, figs 1, 2), cited for Argentina, Bolivia, Brazil, Colombia, Ecuador, and Guyane.

The record of Taschenberg (1870) is possibly incorrect with regards to the type locality. According to our data, there are no other records of this species in the collections listed.

#### Dichotomius (Luederwaltinia) carbonarius

Taxon classificationAnimaliaColeopteraScarabaeidae

Mannerheim, 1829

Copris carbonaria Mannerheim, 1829: 43 (original description. Type locality: Brésil [= Brazil]).

##### Remarks.

This species was recorded for Ecuador by the following authors: Campos (1921: 56) in the localities of Chimbo and Naranjapata; Carvajal et al. (2011: 320–321) provides a list of species, quoting Campos (1921) as reference. According to our data, there are no other records of this species in the collections listed.

#### Eucranium cyclosoma

Taxon classificationAnimaliaColeopteraScarabaeidae

Burmeister, 1861

Eucranium cyclosoma Burmeister, 1861: 60 (original description. Type locality: Ecuador).

##### Remarks.

This species was recorded for Ecuador by the following authors: Gemminger and Harold (1869: 983), list, distribution; Gillet (1911a: 5), complete list of species; Blackwelder (1944: 197), list of species from Latin America; Martínez (1959: 16), but the Ecuadorian record is here considered doubtful; Carvajal et al. (2011: 314–315), list of species, quoting Burmeister (1861) and Blackwelder (1944) as references; Krajcik (2012: 105), list of species.

Ocampo (2010: 14) suggested the Burmeister’s (1861) Ecuadorian record to be erroneous because the genus is endemic to Argentina. Moreover, we did not find any specimens collected in Ecuador in the collections we visited.

#### Ontherus (Ontherus) appendiculatus

Taxon classificationAnimaliaColeopteraScarabaeidae

(Mannerheim, 1829)

Copris appendiculata Mannerheim, 1829: 43 (original description. Type locality: Tijuco [= Brazil, Paraná]).

##### Remarks.

This species was recorded for Ecuador by the following authors: Génier (1996: 81) reported this species in Ecuador Napo [= Napo River], but the author marked this locality with an asterisk (see Génier 1996: 7) to indicate that this records may be incomplete since it does not indicate the exact location along the Napo River; Carvajal et al. (2011: 318–319) quoting Génier (1996); Chamorro et al. (2018: 96) quoting Génier (1996). According to our data, there are no other records of this species in the collections listed.

#### Ontherus (Caelontherus) obliquus

Taxon classificationAnimaliaColeopteraScarabaeidae

Génier, 1996

Ontherus (Caelontherus) obliquus Génier, 1996: 43 (original description. Type locality: Bolivia-Yungas, Incachaca, 2100 m).

##### Remarks.

This species was recorded for Ecuador by the following authors: Génier (1996: 45), no locality [= only reported one male specimen in the IRSN]; Carvajal et al. (2011: 318–319) quoting Génier (1996); Chamorro et al. (2018: 96) quoting Génier (1996). According to our data, there are no other records of this species in the collections listed.

#### Ontherus (Ontherus) sulcator

Taxon classificationAnimaliaColeopteraScarabaeidae

(Fabricius, 1775)

Scarabaeus sulcator Fabricius, 1775: 27 (original description. Type locality: Cajennae [= Cayenne]).

##### Remarks.

This species was recorded for Ecuador by the following authors: Campos (1921: 56), no locality; Génier (1996: 78), no locality; Carvajal et al. 2011: (318–319) quoting Génier (1996); Chamorro et al. (2018: 96) quoting Génier (1996). According to our data, there are no other records of this species in the collections listed.

#### Onthophagus (Onthophagus) clypeatus

Taxon classificationAnimaliaColeopteraScarabaeidae

Blanchard, 1843

Onthophagus clypeatus Blanchard, 1843: 182 (original description. Type locality: province of Santa-Cruz de la Sierra).

##### Remarks.

This species was recorded for Ecuador by the following authors: Boucomont (1932: 322) in the localities of Loja and Cordillère orientale Sabanilla; Pulido-Herrera and Zunino (2007: 99), catalog of species, distribution; Carvajal et al. (2011: 322–323) quoting Boucomont (1932).

It is possible that Boucomont (1932) refers to a different species also belonging to the *clypeatus* group. According to our data, there are no other records of this species in the collections listed.

#### Onthophagus (Onthophagus) incensus

Taxon classificationAnimaliaColeopteraScarabaeidae

Say, 1835

Onthophagus incénsus Say, 1835: 173 (original description. Type locality: Mexico).

##### Remarks.

This species was recorded for Ecuador by the following authors: Boucomont (1932: 308, 324), distribution, described as O.curvicornisvar.incensus Say; Vulcano and Pereira (1967: 565), characters in key, cited as *O.c.incensus* Say, 1837; Howden and Young (1981: 98) characters in key, cited as *Onthophagusincensus* Say; Kohlmann and Solís (2001: 210) cited as *Onthophagusincensus* Say; Pulido-Herrera and Zunino (2007: 106) cited as OnthophaguscurvicornisLatreillevar.incensus; Carvajal et al. (2011: 322–323), list of species; Chamorro et al. (2018: 97) quoting Pulido-Herrera and Zunino (2007).

It is possible that Boucomont (1932) described a completely different species given that *O.incensus* Say, 1835 (currently within the *hircus* group and *curvicornis* complex) is only found in Central America (Howden and Cartwright 1963, Rossini et al 2018b). According to our data, there are no other records of this species in the collections listed.

#### Onthophagus (Onthophagus) ophion

Taxon classificationAnimaliaColeopteraScarabaeidae

Erichson, 1847

Onthophagus ophion Erichson, 1847: 105 (original description. Type locality: Peru).

##### Remarks.

This species was recorded for Ecuador by the following authors: Boucomont (1932: 328), no locality; Pulido-Herrera and Zunino (2007: 111), catalog of species; Carvajal et al. (2011: 322–323) provides list of species. According to our data, there are no other records of this species in the collections listed.

#### Sulcophanaeus actaeon

Taxon classificationAnimaliaColeopteraScarabaeidae

(Erichson, 1847)

Phanaeus actaeon Erichson, 1847: 107 (original description. Type locality: Peru).

##### Remarks.

This species was recorded for Ecuador by the following authors: Gillet (1911a: 81) cited as *PhanaeusActaeon* Er; Blackwelder (1944: 209) cited as *Phanaeusactaeon* Er; Vulcano and Pereira (1967: 574), characters in key, cited as *Phanaeusactaeon* Erichson, 1847; Carvajal et al. (2011: 320–321) provide a list of species.

Edmonds (2000: 22) and Arnaud (2002: 137) mentioned that *S.actaeon* (Erichson, 1847) has only been recorded for Peru (Ica, Junín, and Huanuco).

#### Sulcophanaeus noctis

Taxon classificationAnimaliaColeopteraScarabaeidae

(Bates, 1887)

Phanaeus noctis Bates, 1887: 56 (original description. Type locality: Panama, Bugaba Volcan de Chiriqui. South America, Colombia).

##### Remarks.

This species was recorded for Ecuador by the following authors: Gillet (1911a: 85) cited as *Phanaeusnoctis* Bates; Blackwelder (1944: 210) cited as *Phanaeusnoctis* Bates; Vulcano and Pereira (1967: 575) listed characters in key, cited as *Phanaeusnoctis* Bates, 1887; Edmonds (2000: 27), distribution; Carvajal et al. (2011: 320–321), list of species.

Arnaud (2002: 139) mentioned that *S.noctis* (Bates, 1887) was recorded in Costa Rica, Nicaragua, and Panama. According to our data, there are no other records of this species in the collections listed.

#### Sylvicanthon candezei

Taxon classificationAnimaliaColeopteraScarabaeidae

(Harold, 1869)

Canthon Candezei Harold, 1869a: 96 (original description. Type locality: Tapajos).

##### Remarks.

This species was recorded for Ecuador by the following authors: Blackwelder (1944: 198) cited as *Canthoncandenzei* Har; Martínez et al. (1964: 8–9) distribution; Vulcano and Pereira (1964: 661) cited as *Glaphyrocanthon candènzei* (Harold, 1869); Vulcano and Pereira (1967: 561), characters in key, cited as *Glaphyrocanthon candènzei* (Harold, 1869); Carvajal et al. (2011: 317–318), list of species.

Cupello and Vaz-de-Mello (2018: 72) mentioned that *S.candezei* (Harold, 1869) has only been recorded for Brazil. According to our data, there are no other records of this species in the collections listed.

#### Sylvicanthon aequinoctialis

Taxon classificationAnimaliaColeopteraScarabaeidae

(Harold, 1868)

Canthon aequinoctialis Harold, 1868d: 14 (characters in key), 79 (original description. Type locality: Columbien, Neu-Granada [= Colombia, Nueva Granada]).

##### Remarks.

This species was recorded for Ecuador by the following authors: Schmidt (1922: 72), Coca locality [= El Coca city, formerly known as Francisco de Orellana]; Blackwelder (1944: 198) misspelled the name *Canthonaequinoctiale* Har; Pereira and Martínez (1956a: 128) distribution, cited as *Glaphyrocanthonaequinoctialis* (Harold, 1868) comb. n.; Martínez et al. (1964: 8–9), distribution; Vulcano and Pereira (1964: 661) cited as *Glaphyrocanthonaequinoctialis* (Harold, 1868); Solís and Kohlmann (2002: 6), redescription.

Cupello and Vaz-de-Mello (2018: 93) mentioned that *S.aequinoctialis* (Harold, 1868) has been recorded in Colombia, Costa Rica, Honduras, Nicaragua, and Panama. According to our data, there are no other records of this species in the collections listed.

## Discussion

Including comparative information from historical catalogs and checklists with Ecuadorian records (see Table 2), the number of species registered in the country has increased to more than 220 valid records .

**Table 2.** History of authors and the number of species of Scarabaeinae they recorded in Ecuador.

Author (year of publication)	Type of study	Number of species
Gemminger and Harold (1869)	Catalog	11
Gillet (1911a)	Catalog	30
Campos (1921)	Checklist	26
Blackwelder (1944)	Checklist	82
Carvajal et al. (2011)	Book-checklist	265
Krajcik (2012)	Checklist	94
Chamorro et al. (2018)	Illustrated key to the genera and subgenera, and checklist	220
Current research	Annotated catalog and bibliography, original material examined where possible	223

However, some catalogs and checklists have errors in the geographical distribution records of several species or are incorrect regarding the nomenclature and validity of scientific names (genus and/or species). For example, in his checklist, Carvajal et al. (2011) listed *Canthon kästneri* Balth. 1839 [= Balthasar, 1839], and *Scybalocanthonkastneri* Balthasar, 1939 (without italics for all species names in this list) as two different species, whereas Halffter and Martínez (1977) and Bezdek and Hajek (2011) cited *Scybalocanthonkastneri* (Balthasar, 1939) as the only valid species name.

Regarding Scarabaeinae richness in Ecuador and other Neotropical countries, there are only a few taxonomic catalogs that include distributional data. It is important to emphasize that Ecuador, being one of the smallest countries in South America, has considerable species richness compared to the larger countries such as Argentina, Bolivia, and Venezuela (see Table 3).

**Table 3.** Comparison of studies on dung beetles Scarabaeinae (Coleoptera: Scarabaeidae) in other Neotropical countries.

Country (author and year of publication)	Type of study	Registered species	Endemic species	Area
Argentina (Martínez 1959)	Catalog	202	42	2,780,400 km²
Bolivia (Hamel-Leigue et al. 2006)	Checklist	216		1,098,581 km²
Brazil (Vaz-de-Mello 2000)	Checklist	618	223	8,514,877 km²
Colombia (Medina et al. 2001)	Checklist	283		1,141,748 km²
Costa Rica (Solís and Kohlmann 2012)	Checklist	182		51,100 km^2^
Ecuador (Carvajal et al. 2011)	Book-checklist	265		283,561 km^2^
Ecuador (Chamorro et al. 2018)	Checklist and illustrated key to the genera-subgenera of Scarabaeinae	220		283,561 km^2^
Ecuador current research	Annotated catalog and bibliography, original material examined where possible	223	45	283,561 km^2^
Mexico (Morón 2003)	Book	228		1,964,375 km²
Panama (Howden and Young 1981)	Catalog	113	20	78,200 km^2^
Panama (Ratcliffe 2002)	Checklist	132		78,200 km^2^
Peru (Ratcliffe et al. 2015)	Checklist	278	26	1,285,216 km^2^
Venezuela (Roze 1955)	Checklist	72		916,445 km^2^

Currently, there is little interest among researchers to develop a catalog of this group of scarab beetles. See Table 3 for a summary of the Neotropical countries where this kind of studies was conducted on dung beetles.

Finally, the number of known species from Ecuador is predicted to increase over time due to revisions of genera and new records of the Scarabaeinae (especially in the genera *Anomiopus*, *Ateuchus*, *Canthidium*, *Canthon*, *Canthonella*, *Cryptocanthon*, *Deltochilum*, *Dichotomius*, *Eutrichillum*, *Malagoniella*, *Onthophagus*, *Scybalocanthon, Sinapisoma*, and *Uroxys*).

## Plates

**Plate 2.**
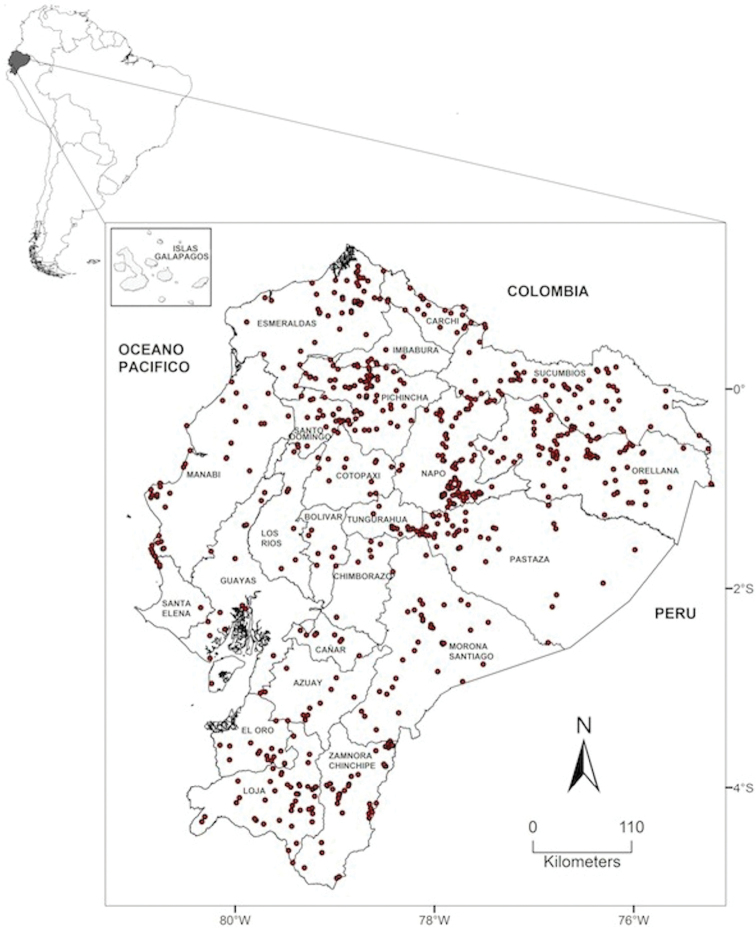
Geographic distribution of localities in Scarabaeinae (Coleoptera: Scarabaeidae) from Ecuador (political limits of Ecuadorian provinces as of 2012).

**Plate 3.**
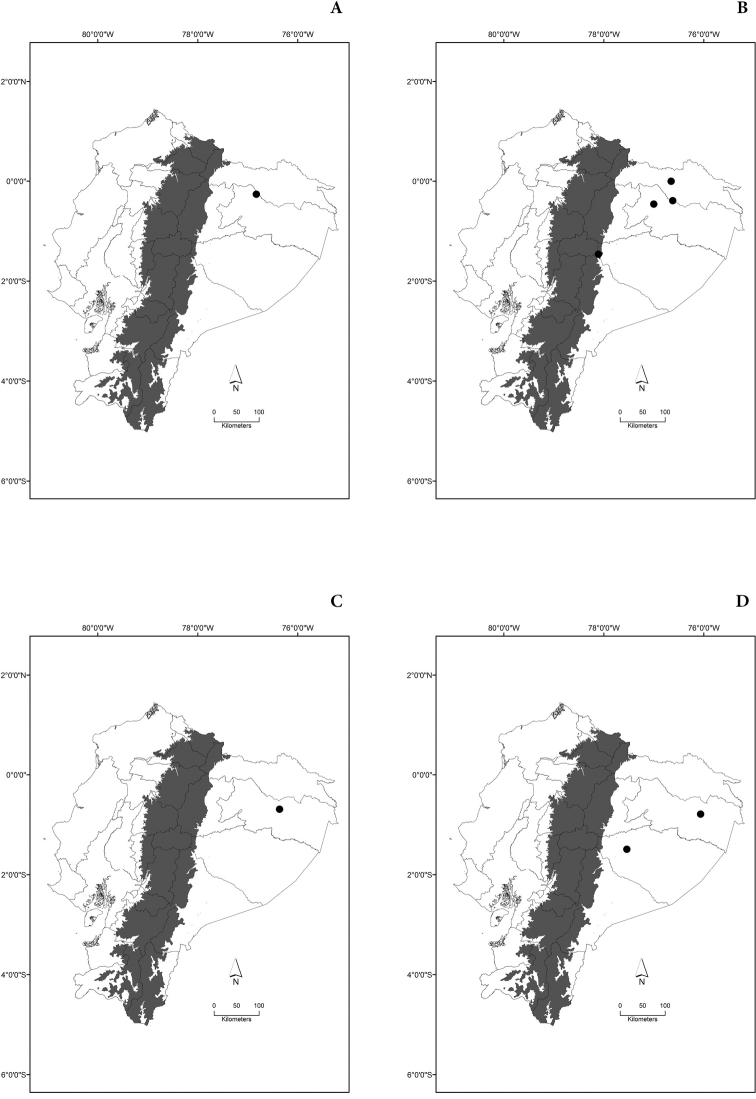
Distribution of: **A***Anomiopusbrevipes* (Waterhouse, 1891) **B***Anomiopusintermedius* (Waterhouse, 1891) **C***Anomiopuspictus* (Harold, 1862) **D***Ateuchusaeneomicans* (Harold, 1868).

**Plate 4.**
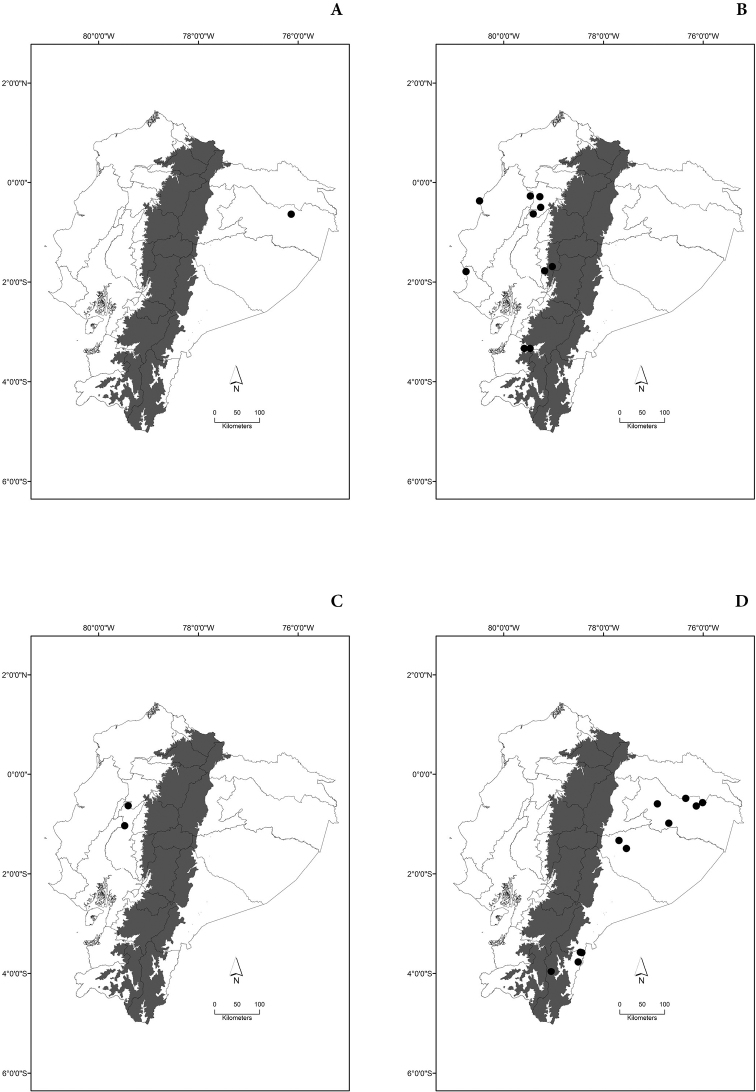
Distribution of: **A***Ateuchusconnexus* (Harold, 1868) **B***Ateuchusecuadorensis* (Boucomont, 1928) **C***Ateuchusparvus* (Balthasar, 1939) **D***Ateuchusscatimoides* (Balthasar, 1939).

**Plate 5.**
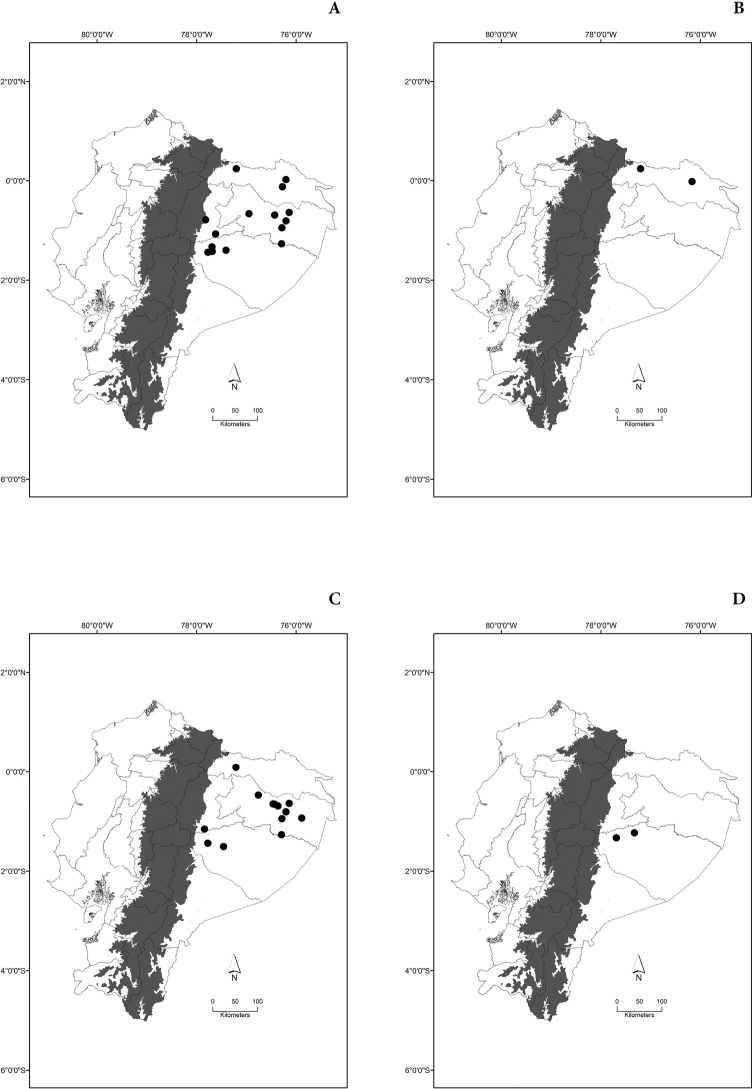
Distribution of: **A***Bdelyrusgenieri* Cook, 1998 **B***Bdelyrusgrandis* Cook, 1998 **C***Bdelyrushowdeni* Cook, 1998 **D***Bdelyruslobatus* Cook, 1998.

**Plate 6.**
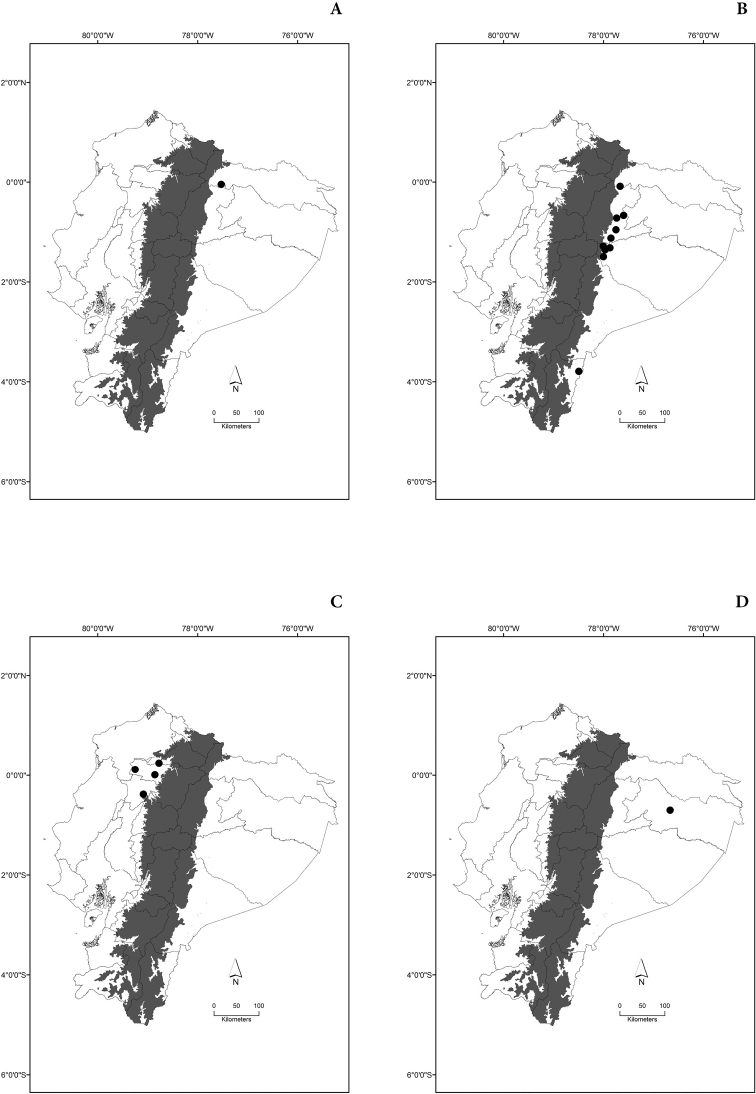
Distribution of: **A***Bdelyrusparvoculus* Cook, 1998 **B***Bdelyruspecki* Cook, 1998 **C***Bdelyrusseminudus* Bates, 1887 **D***Bdelyrustriangulus* Cook, 1998.

**Plate 7.**
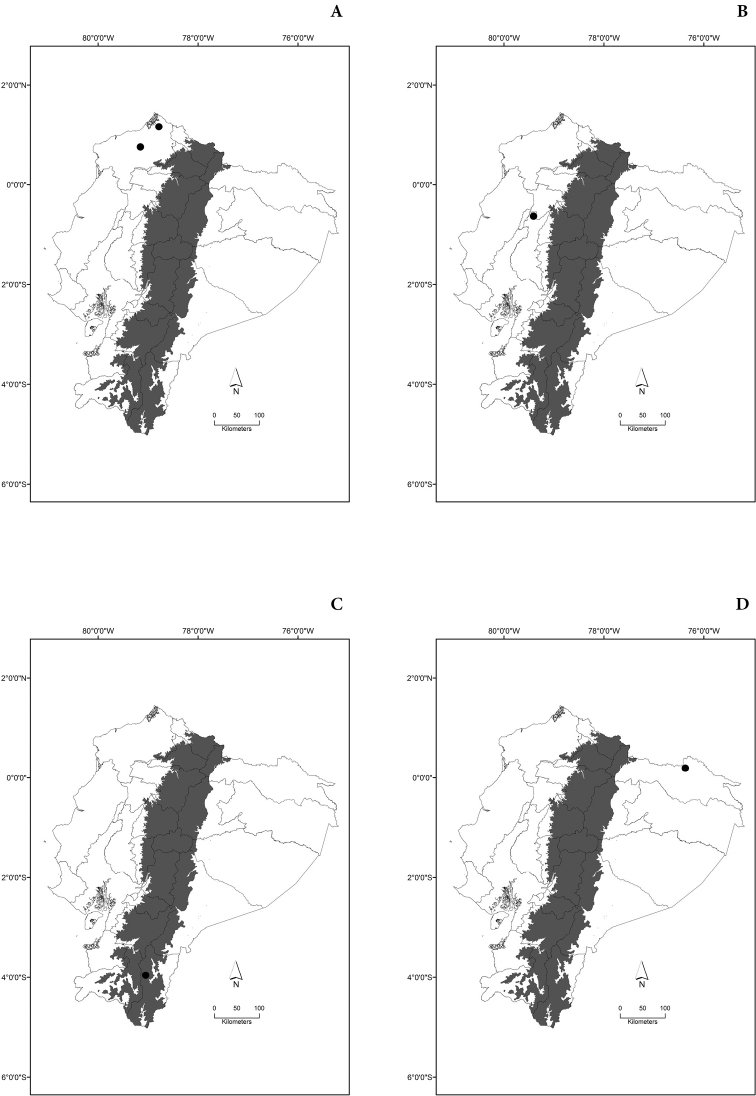
Distribution of: **A***Bradypodidiumbradyporum* (Boucomont, 1928) **B**Canthidium (Canthidium) aurifex Bates, 1887 **C**Canthidium (Canthidium) flavum Balthasar, 1939 **D**Canthidium (Canthidium) funebre Balthasar, 1939.

**Plate 8.**
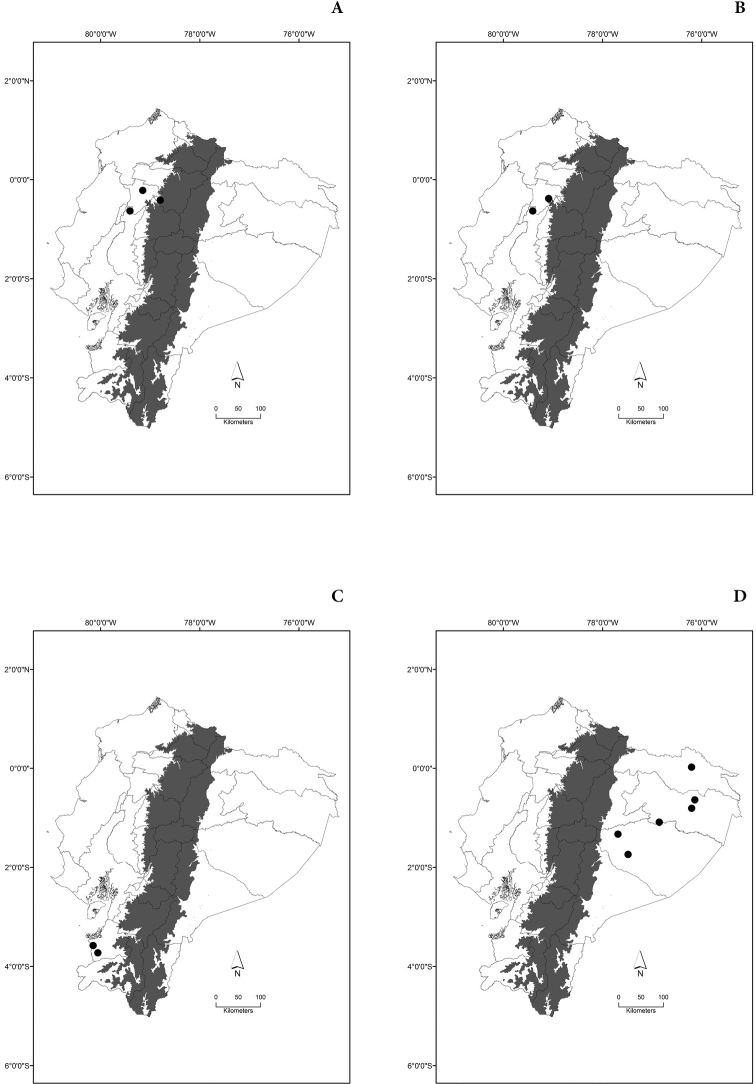
Distribution of: **A**Canthidium (Canthidium) hespenheidei Howden & Young, 1981 **B**Canthidium (Canthidium) macroculare Howden & Gill, 1987 **C**Canthidium (Canthidium) muticum (Boheman, 1858) **D**Canthidium (Canthidium) onitoides (Perty, 1830).

**Plate 9.**
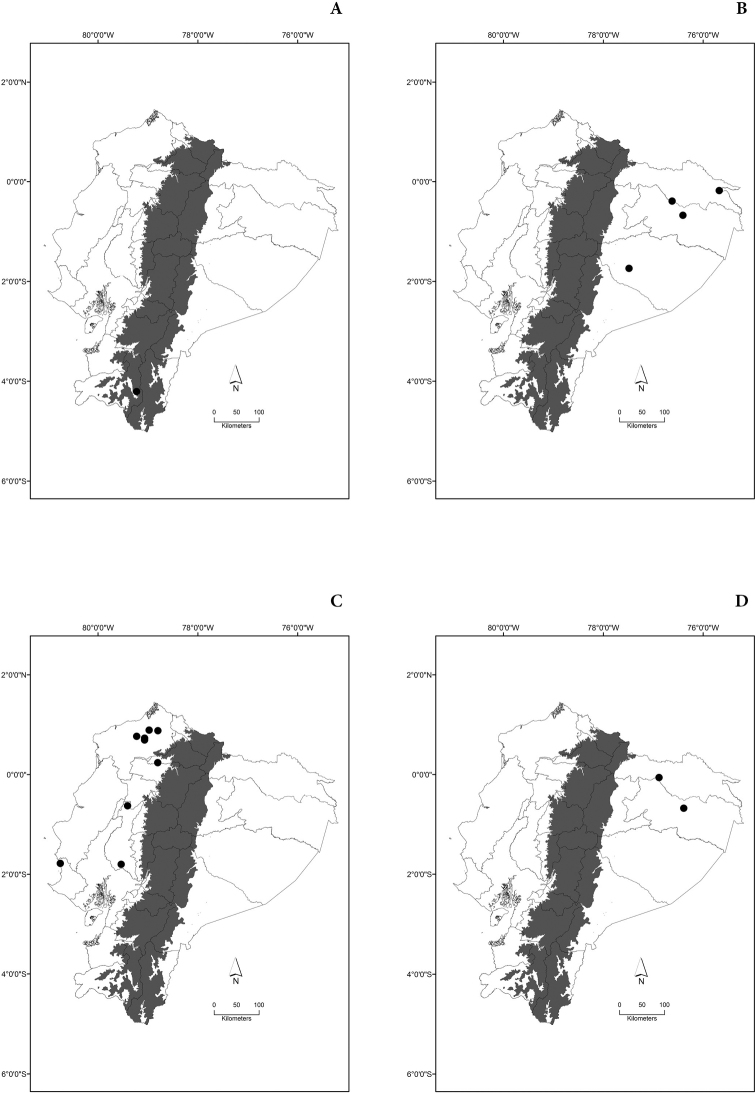
Distribution of: **A**Canthidium (Canthidium) opacum Balthasar, 1939 **B**Canthidium (Canthidium) orbiculatum (Lucas, 1857) revalidated name **C**Canthidium (Canthidium) pseudaurifex Balthasar, 1939 **D**Canthidium (Canthidium) rufinum Harold, 1867.

**Plate 10.**
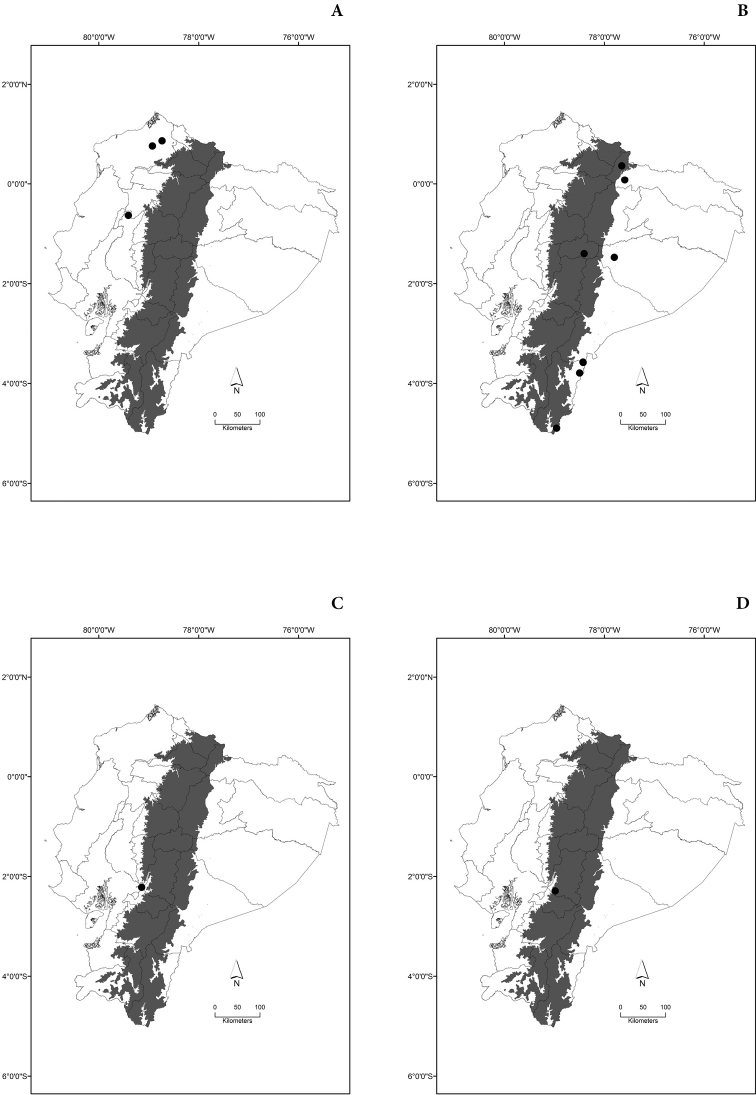
Distribution of: **A**Canthidium (Neocanthidium) centrale Boucomont, 1928 **B**Canthidium (Neocanthidium) coerulescens Balthasar, 1939 **C**Canthidium (Neocanthidium) escalerai Balthasar, 1939 **D**Canthidium (Neocanthidium) inoptatum Balthasar, 1939.

**Plate 11.**
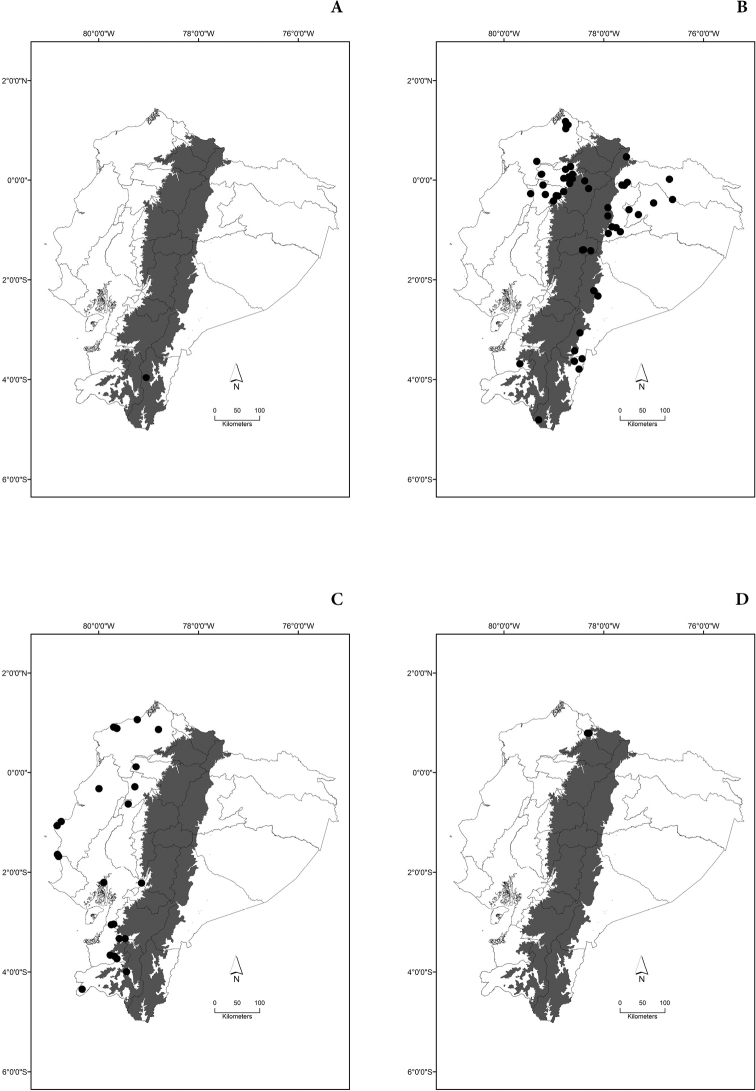
Distribution of: **A**Canthidium (Neocanthidium) luteum Balthasar, 1939 **B**Canthon (Canthon) aberrans (Harold, 1868) **C**Canthon (Canthon) delicatulus Balthasar, 1939 **D**Canthon (Canthon) obscuriellus Schmidt, 1922.

**Plate 12.**
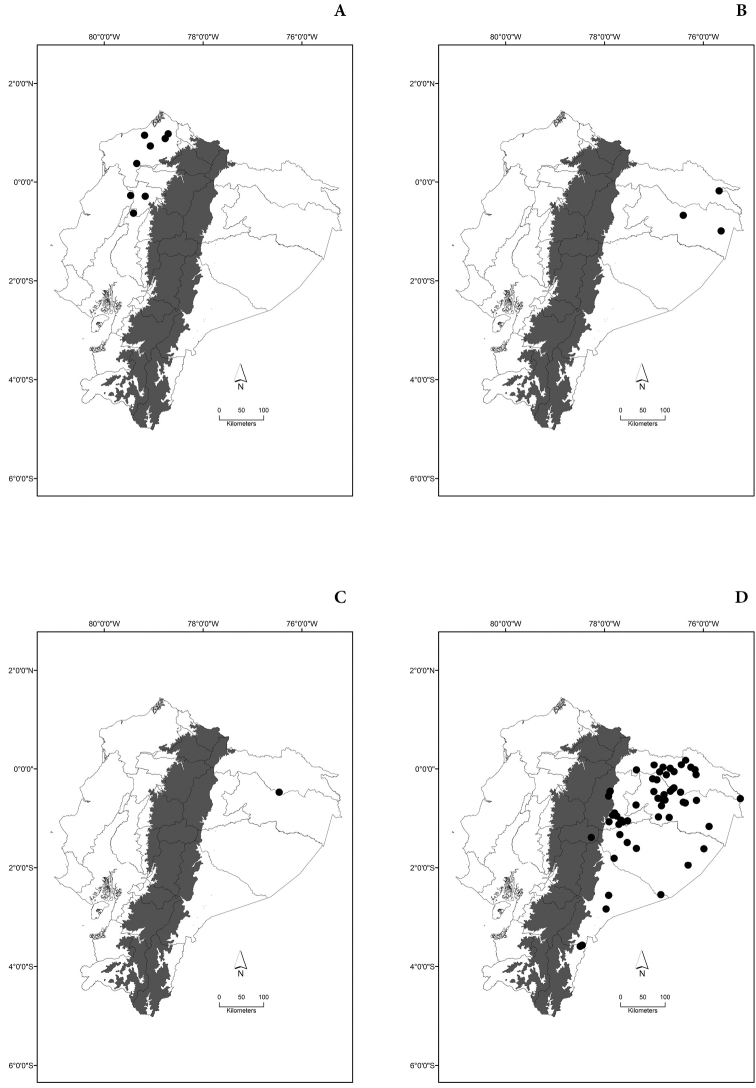
Distribution of: **A**Canthon (Glaphyrocanthon) angustatus Harold, 1867 **B**Canthon (Glaphyrocanthon) bimaculatus Schmidt, 1922 **C**Canthon (Glaphyrocanthon) brunnipennis Schmidt, 1922 **D**Canthon (Glaphyrocanthon) luteicollis Erichson, 1847.

**Plate 13.**
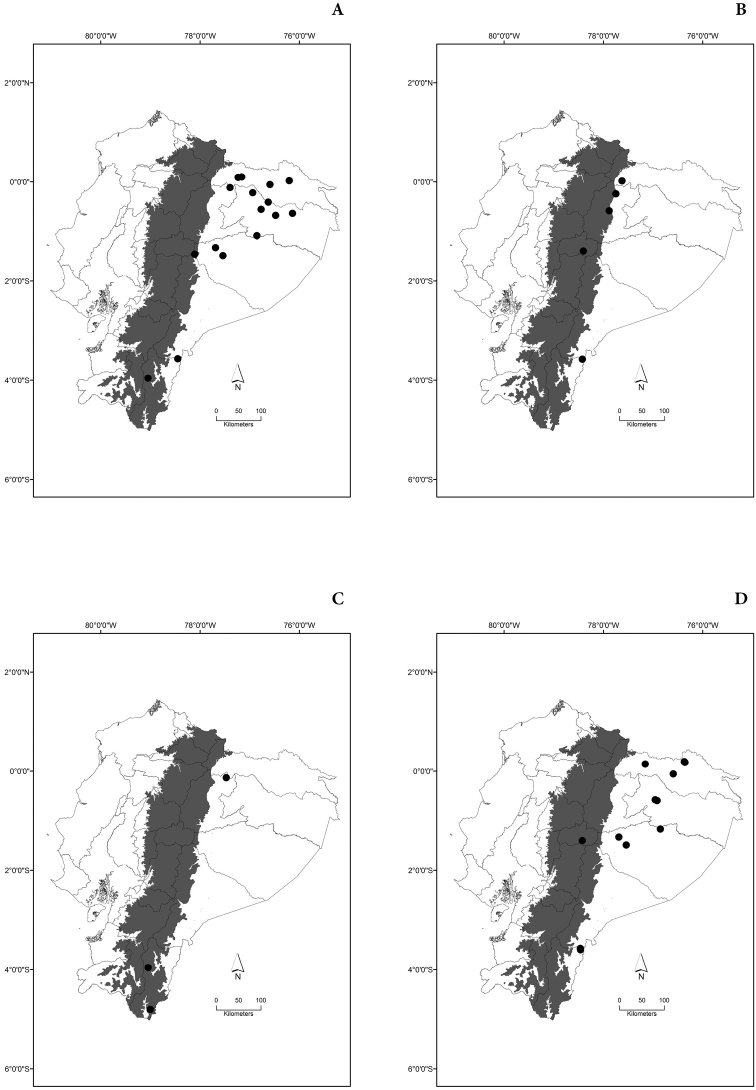
Distribution of: **A**Canthon (Glaphyrocanthon) ohausi Balthasar, 1939, stat. n. **B**Canthon (Glaphyrocanthon) pallidus Schmidt, 1922 **C**Canthon (Glaphyrocanthon) politus Harold, 1868 **D**Canthon (Glaphyrocanthon) quadriguttatus (Olivier, 1789).

**Plate 14.**
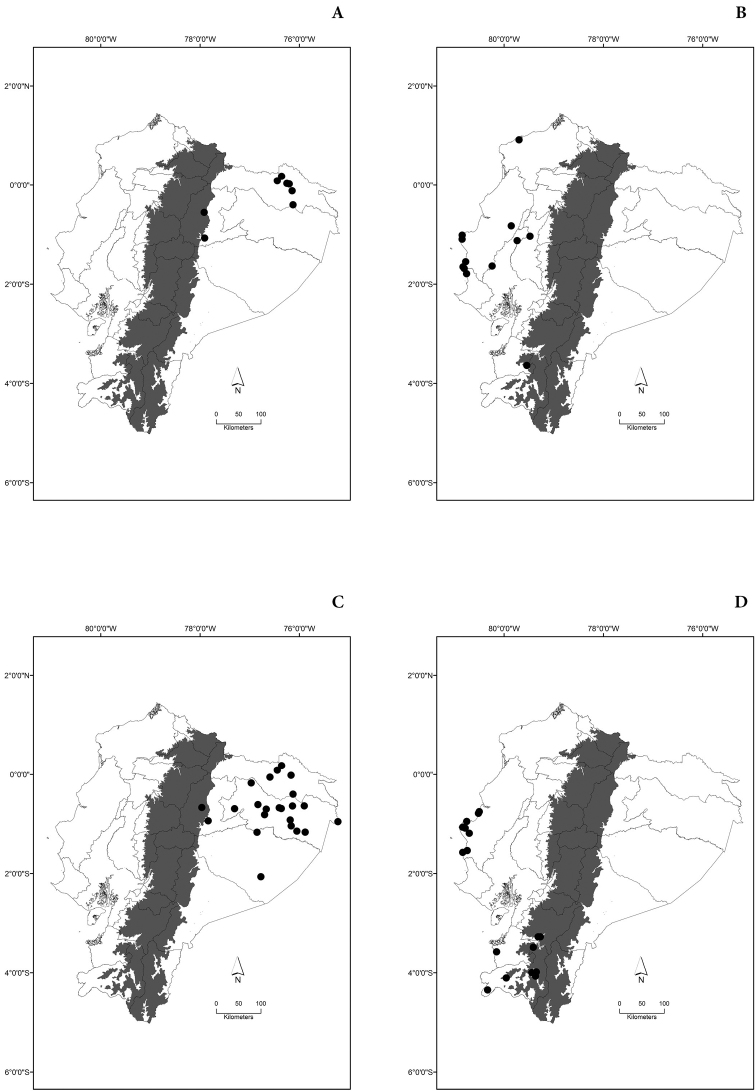
Distribution of: **A**Canthon (Glaphyrocanthon) semiopacus Harold, 1868 **B**Canthon (Glaphyrocanthon) subhyalinoides Balthasar, 1939 **C**Canthon (Goniacanthon) fulgidusmartinezi Nunes, Nunes & Vaz-de-Mello, 2018 **D***Canthonbalteatus* Boheman, 1858.

**Plate 15.**
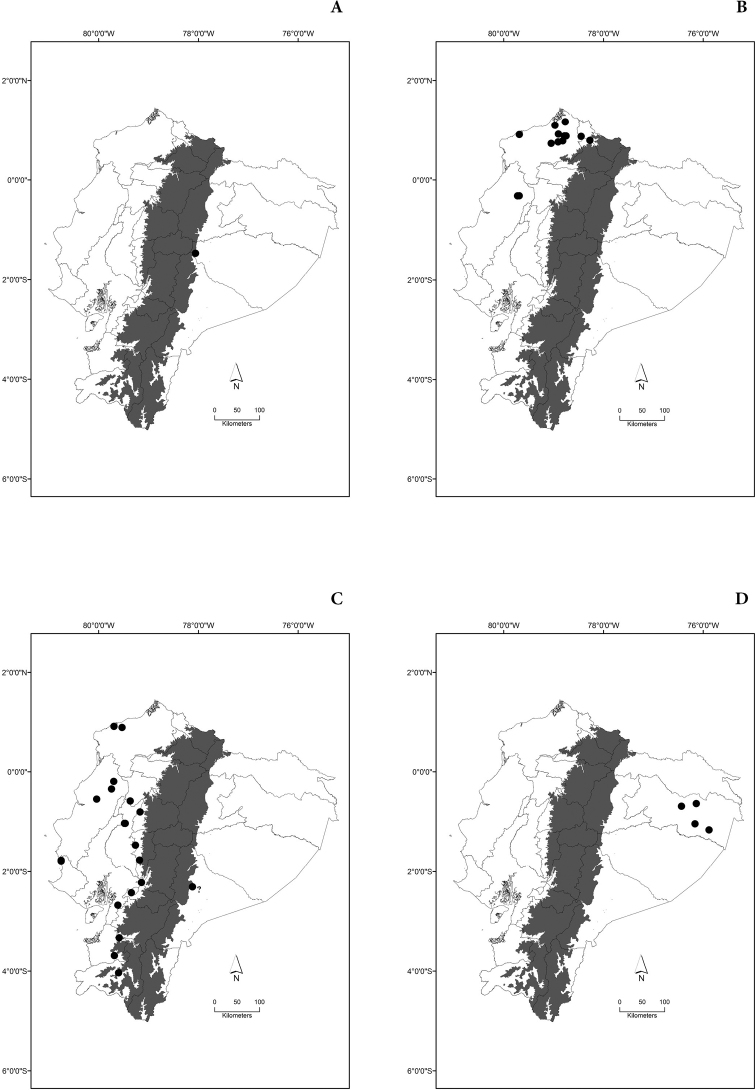
Distribution of: **A***Canthonsericatus* Schmidt, 1922 **B**Copris (Copris) davidi Darling & Génier, 2018 **C**Copris (Copris) susanae Darling & Génier, 2018 **D**Coprophanaeus (Coprophanaeus) callegarii Arnaud, 2002.

**Plate 16.**
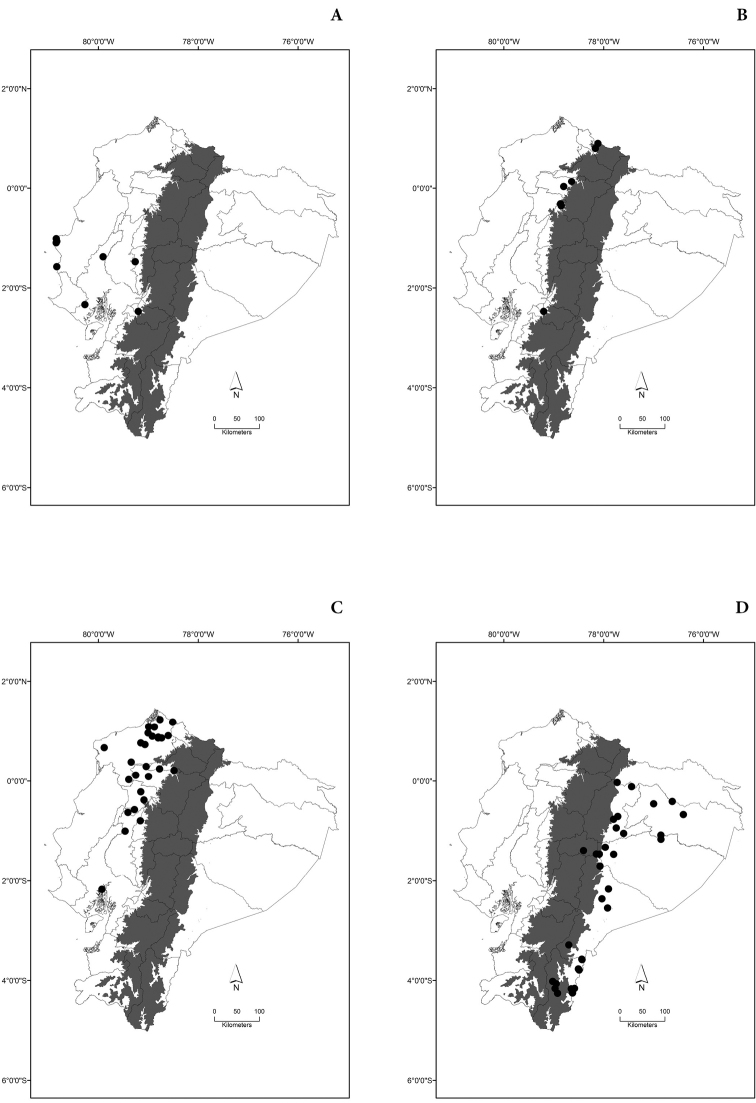
Distribution of: **A**Coprophanaeus (Coprophanaeus) conocephalus (d’Olsoufieff, 1924) **B**Coprophanaeus (Coprophanaeus) edmondsi Arnaud, 1997 **C**Coprophanaeus (Coprophanaeus) morenoi Arnaud, 1982 **D**Coprophanaeus (Coprophanaeus) ohausi (Felsche, 1911).

**Plate 17.**
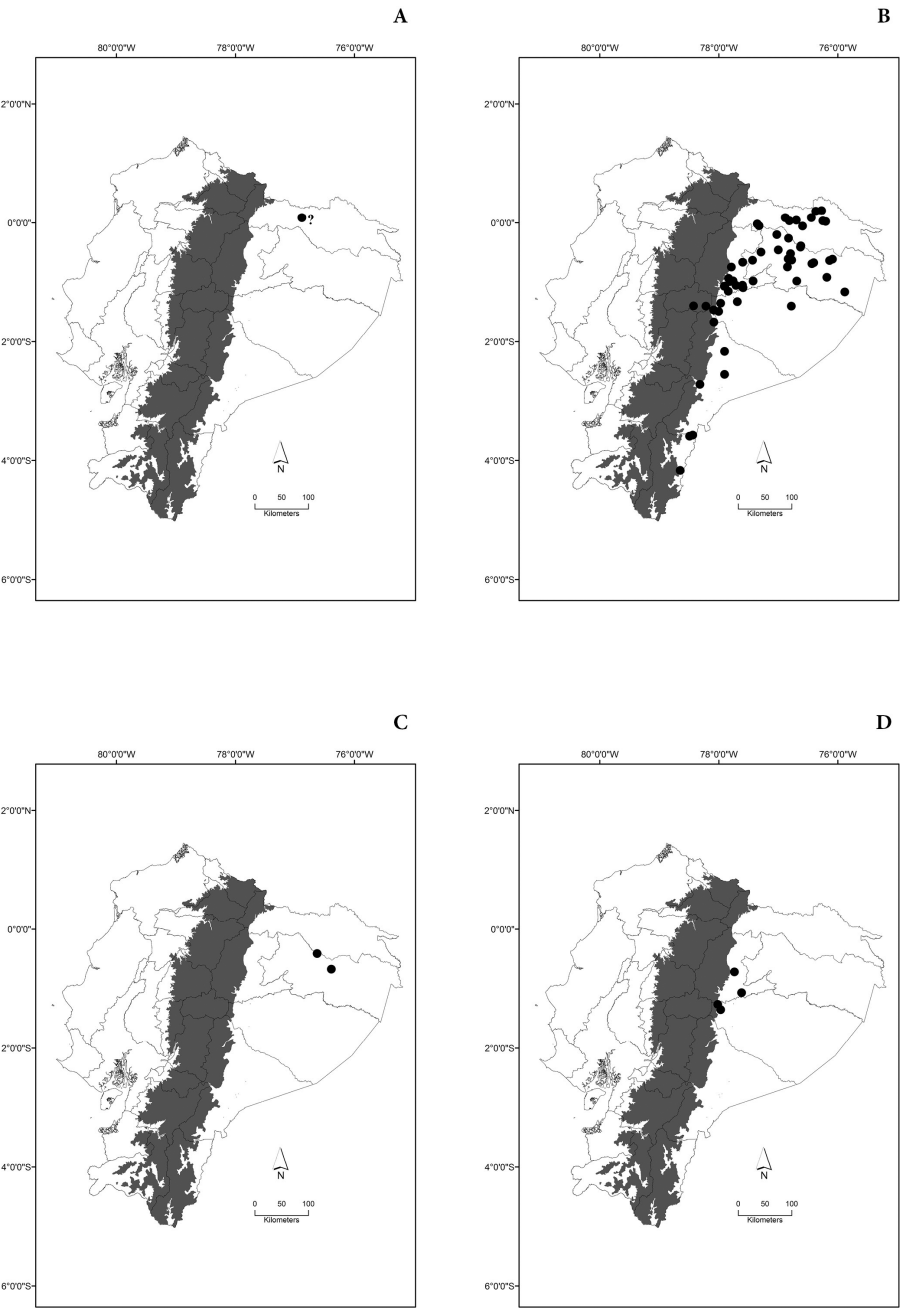
Distribution of: **A**Coprophanaeus (Coprophanaeus) suredai Arnaud, 1996 **B**Coprophanaeus (Coprophanaeus) telamon (Erichson, 1847) **C***Cryptocanthoncurticrinis* Cook, 2002 **D***Cryptocanthongenieri* Cook, 2002.

**Plate 18.**
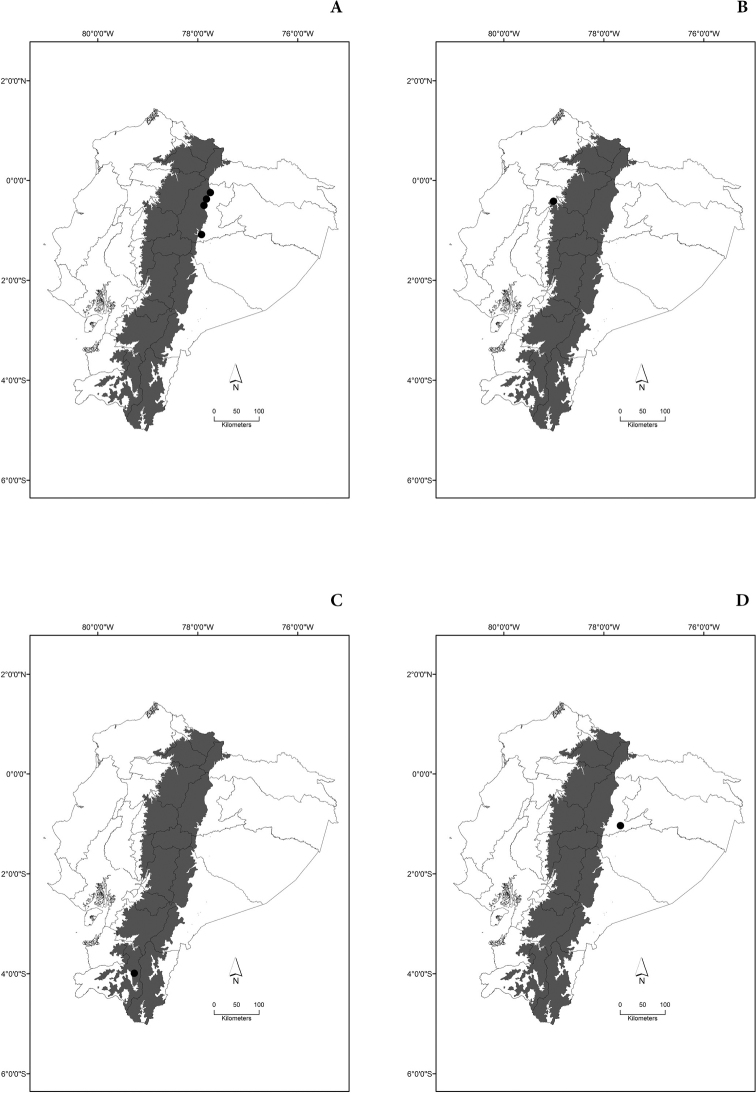
Distribution of: **A***Cryptocanthonnapoensis* Cook, 2002 **B***Cryptocanthonotonga* Cook, 2002 **C***Cryptocanthonparadoxus* Balthasar, 1942 **D***Cryptocanthonurguensis* Cook, 2002.

**Plate 19.**
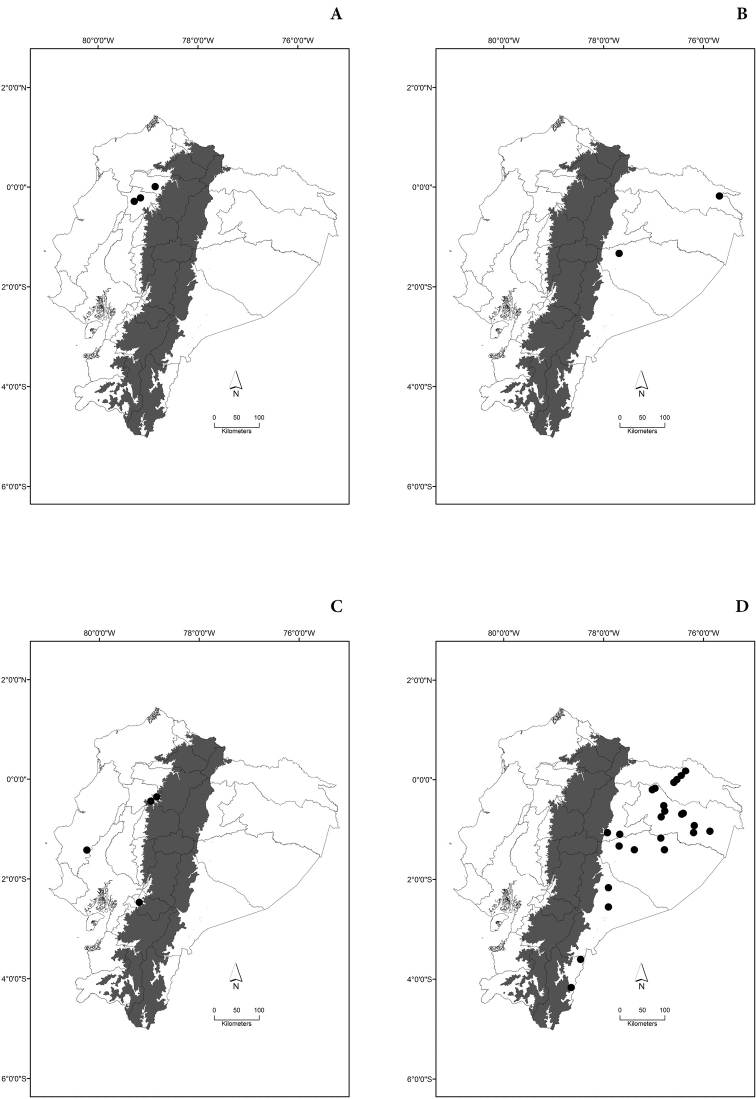
Distribution of: **A**Deltochilum (Aganhyboma) arturoi Silva, Louzada & Vaz-de-Mello, 2015 **B**Deltochilum (Aganhyboma) larseni Silva, Louzada & Vaz-de-Mello, 2015 **C**Deltochilum (Calhyboma) arrowi Paulian, 1939, stat. n. **D**Deltochilum (Calhyboma) carinatum (Westwood, 1837).

**Plate 20.**
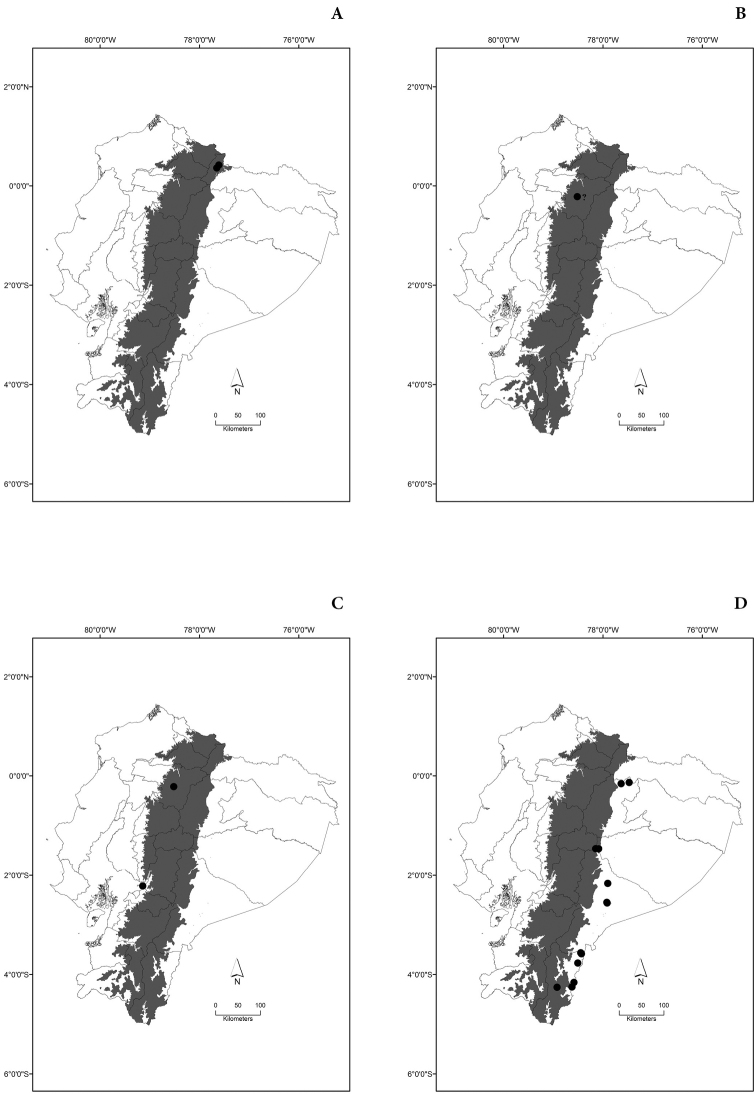
Distribution of: **A**Deltochilum (Calhyboma) hypponum (Buquet, 1844) **B**Deltochilum (Calhyboma) luederwaldti Pereira & D’Andretta, 1955 **C**Deltochilum (Calhyboma) mexicanum Burmeister, 1848 **D**Deltochilum (Calhyboma) robustus Molano & González, 2009.

**Plate 21.**
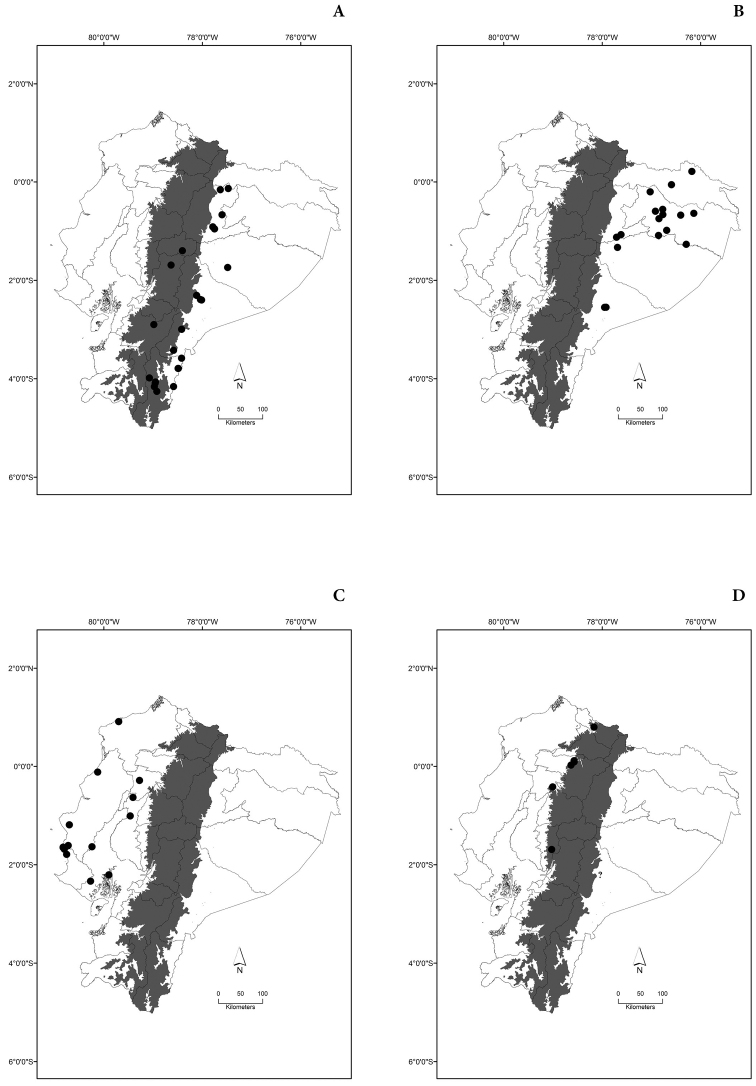
Distribution of: **A**Deltochilum (Calhyboma) tessellatum Bates, 1870 **B**Deltochilum (Deltochilum) orbiculare Lansberge, 1874 **C**Deltochilum (Deltochilum) rosamariae Martínez, 1991 **D**Deltochilum (Deltohyboma) aequinoctiale (Buquet, 1844).

**Plate 22.**
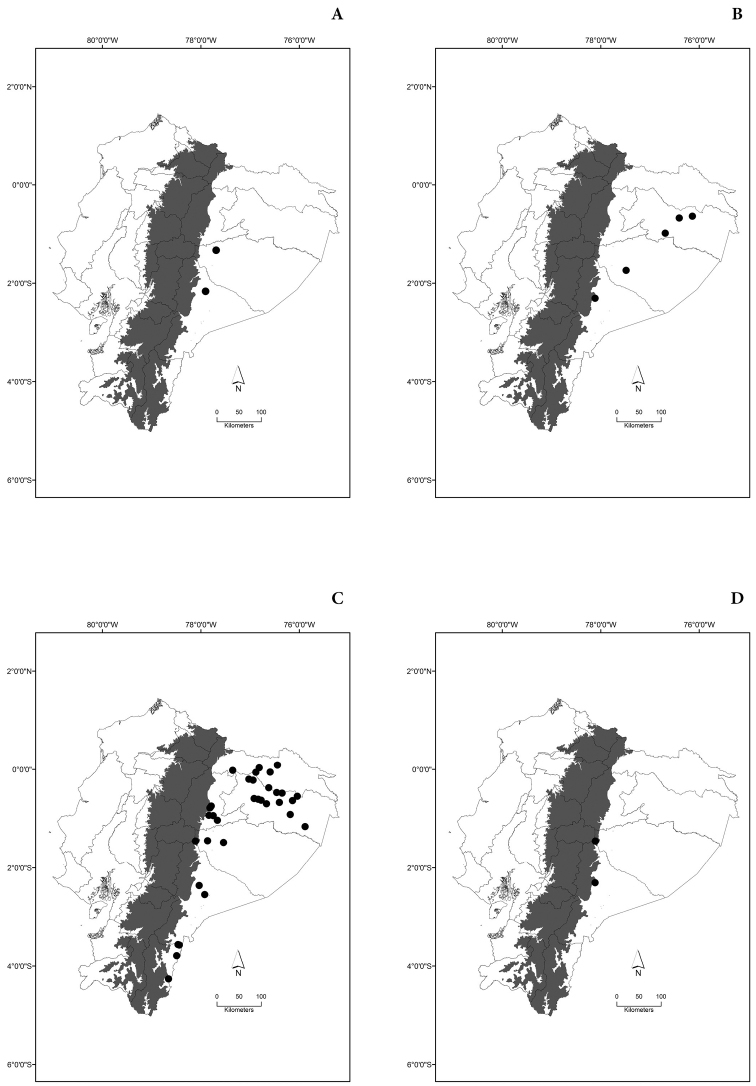
Distribution of: **A**Deltochilum (Deltohyboma) barbipes Bates, 1870 **B**Deltochilum (Deltohyboma) batesi Paulian, 1938 **C**Deltochilum (Deltohyboma) crenulipes Paulian, 1938 **D**Deltochilum (Deltohyboma) peruanum Paulian, 1938.

**Plate 23.**
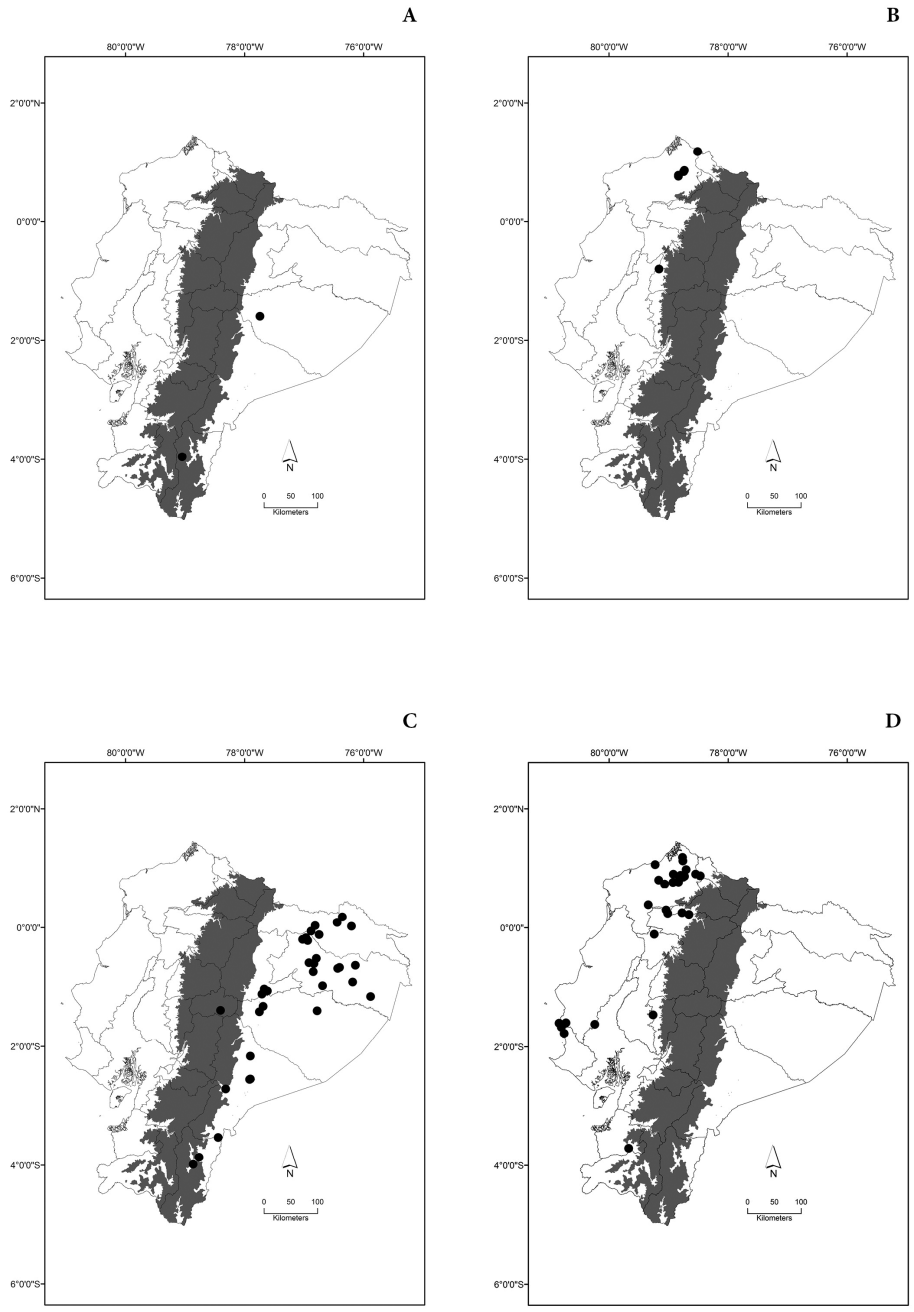
Distribution of: **A**Deltochilum (Deltohyboma) speciosissimum Balthasar, 1939 **B**Deltochilum (Hybomidium) loperae González & Molano, 2009 **C**Deltochilum (Hybomidium) orbignyiamazonicum Bates, 1887 **D**Deltochilum (Hybomidium) panamensis Howden, 1966.

**Plate 24.**
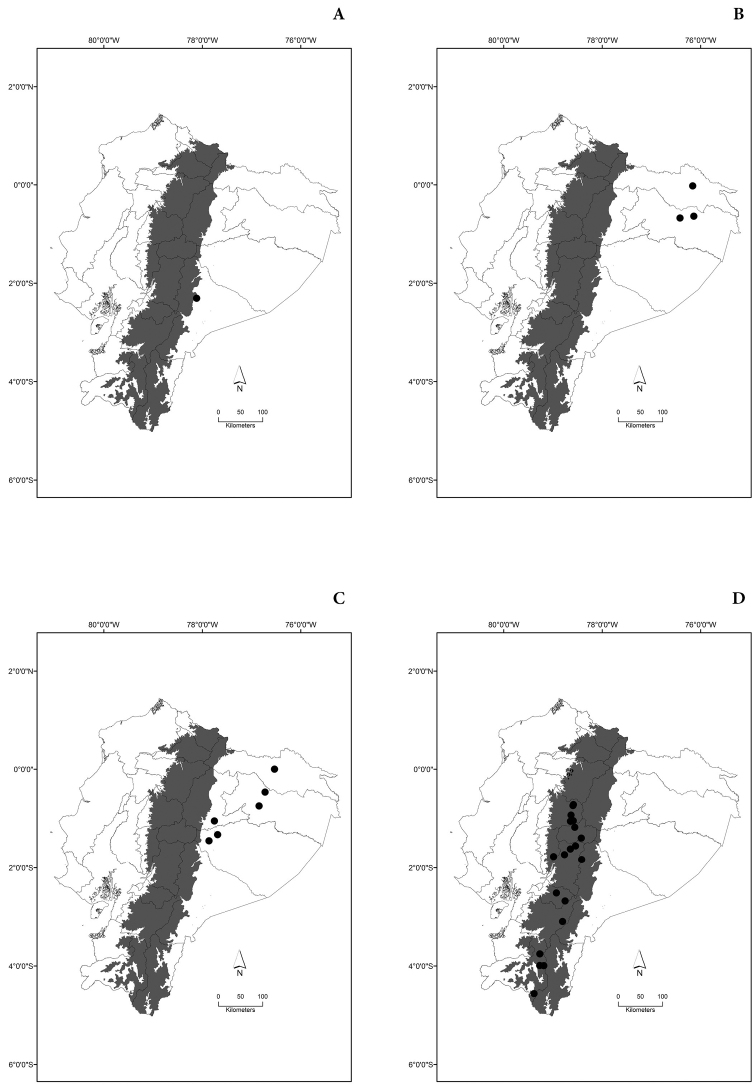
Distribution of: **A**Dendropaemon (Crassipaemon) morettoi Génier & Arnaud, 2016 **B**Dendropaemon (Glaphyropaemon) angustipennis Harold, 1869 **C**Dichotomius (Dichotomius) compressicollis (Luederwaldt, 1929) **D**Dichotomius (Dichotomius) cotopaxi (Guerin-Meneville, 1855).

**Plate 25.**
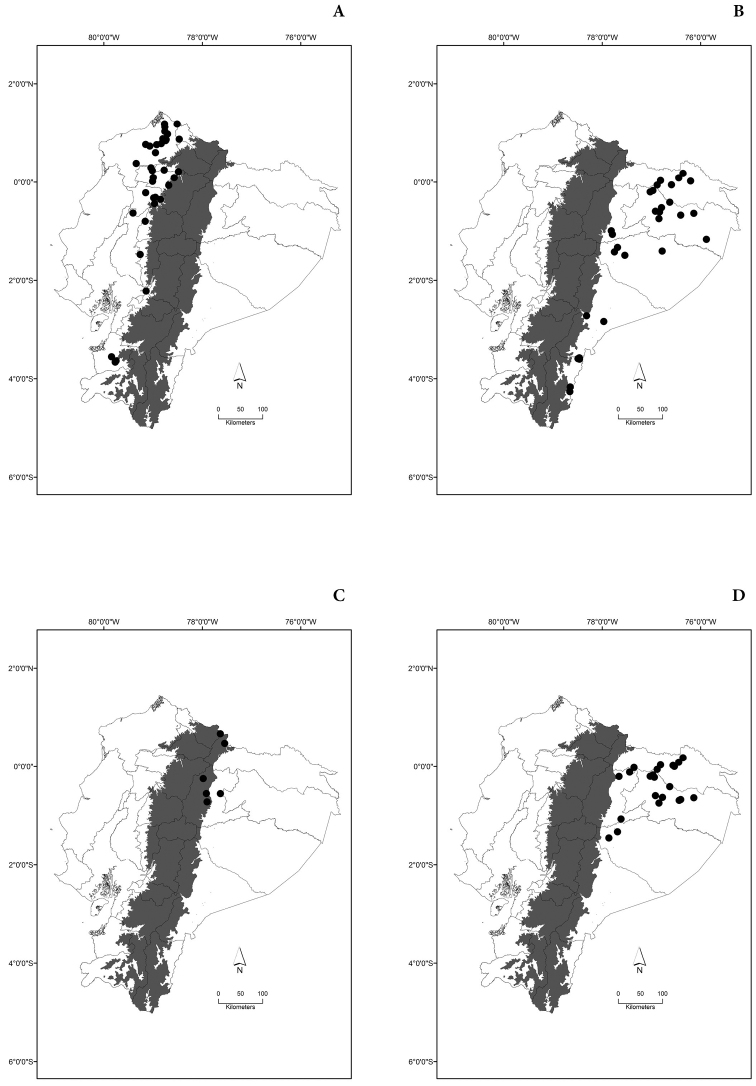
Distribution of: **A**Dichotomius (Dichotomius) divergens (Luederwaldt, 1923) **B**Dichotomius (Dichotomius) mamillatus (Felsche, 1901) **C**Dichotomius (Dichotomius) monstrosus (Harold, 1875) **D**Dichotomius (Dichotomius) ohausi (Luederwaldt, 1923).

**Plate 26.**
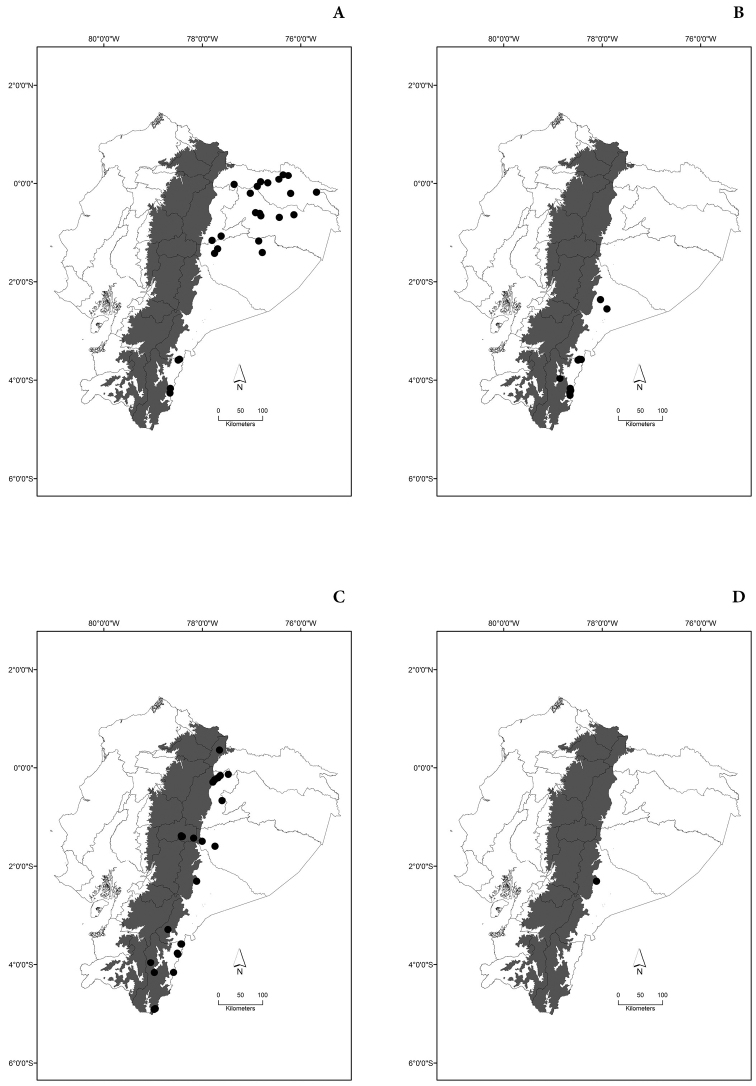
Distribution of: **A**Dichotomius (Dichotomius) podalirius (Felsche, 1901) **B**Dichotomius (Dichotomius) prietoi Martínez & Martínez, 1982 **C**Dichotomius (Dichotomius) protectus (Harold, 1867) **D**Dichotomius (Dichotomius) provisorius (Luederwaldt, 1925).

**Plate 27.**
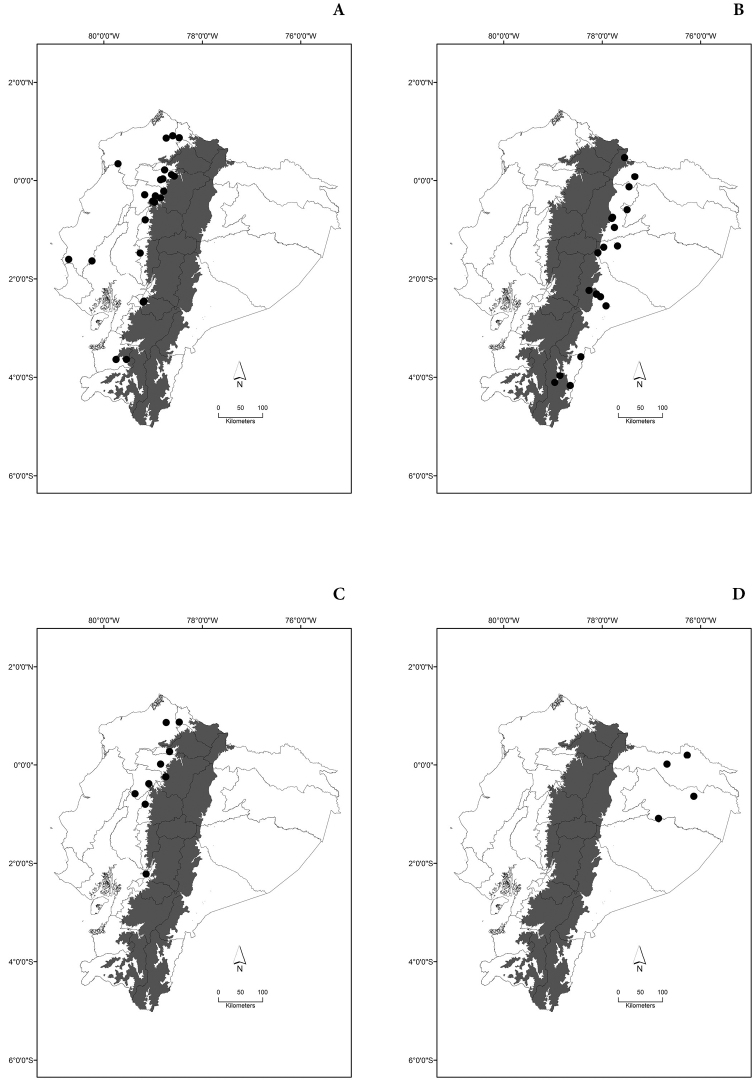
Distribution of: **A**Dichotomius (Dichotomius) quinquedens (Felsche, 1910) **B**Dichotomius (Dichotomius) quiquelobatus (Felsche, 1901) **C**Dichotomius (Dichotomius) reclinatus (Felsche, 1901) **D**Dichotomius (Dichotomius) robustus (Luederwaldt, 1935).

**Plate 28.**
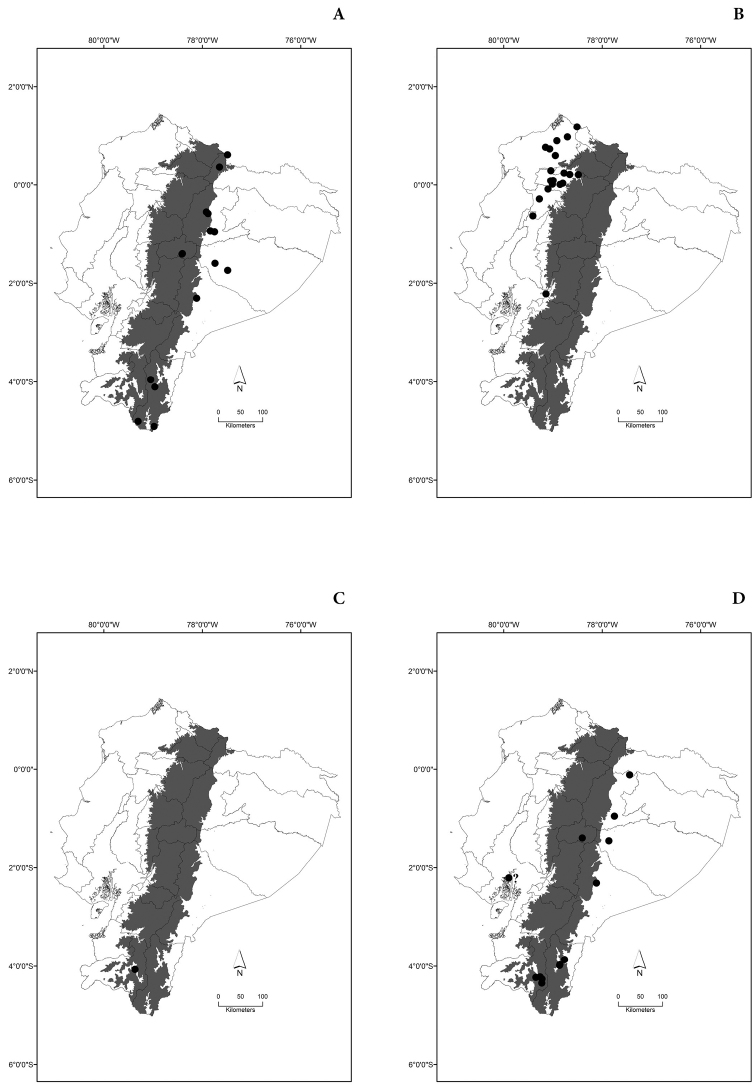
Distribution of: **A**Dichotomius (Dichotomius) satanasangustus (Luederwaldt, 1923) **B**Dichotomius (Luederwaldtinia) fortepunctatus (Luederwaldt, 1923), revalidated name **C**Dichotomius (Luederwaldtinia) hempeli (Pereira, 1942) **D**Dichotomius (Luederwaldtinia) problematicus (Luederwaldt, 1923).

**Plate 29.**
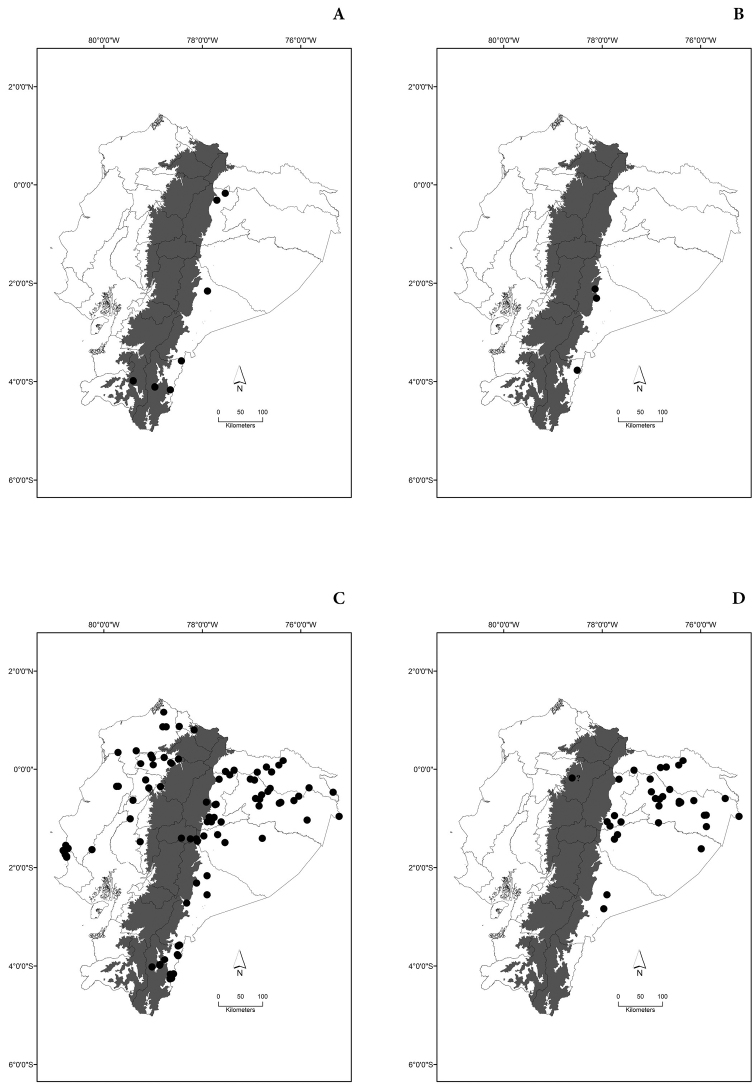
Distribution of: **A**Dichotomius (Luederwaldtinia) simplicicornis (Luederwaldt, 1935) **B**Dichotomius (Selenocopris) fonsecae (Luederwaldt, 1926) **C***Eurysternuscaribaeus* (Herbst, 1789) **D***Eurysternuscayennensis* Castelnau, 1840.

**Plate 30.**
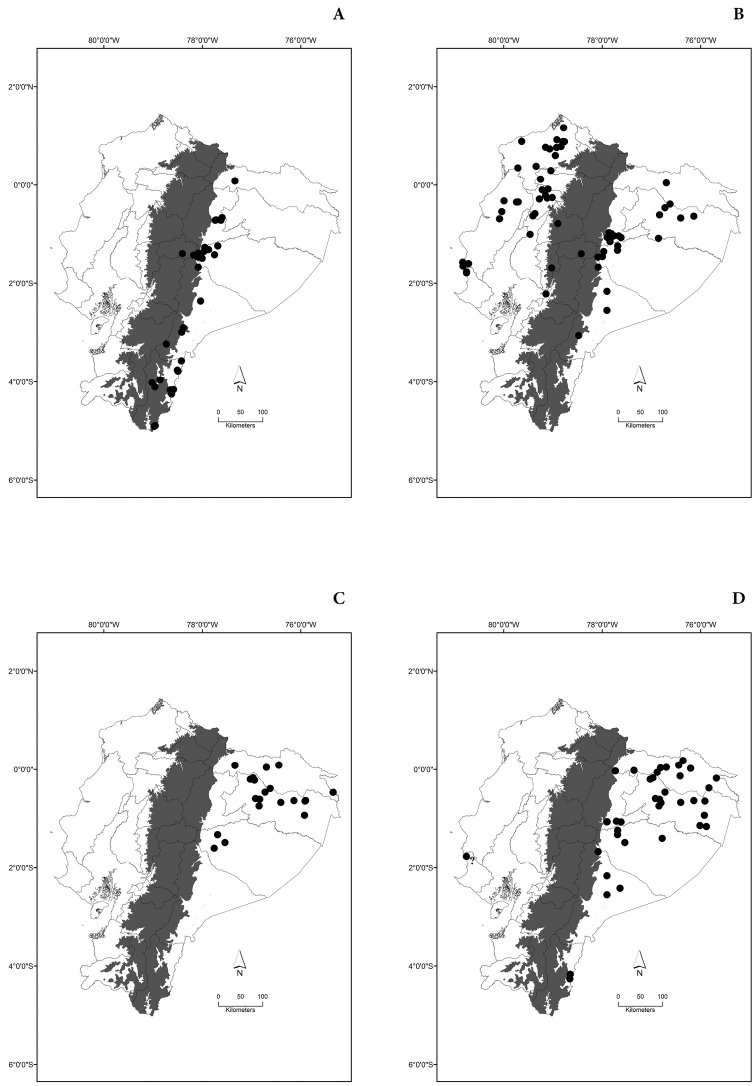
Distribution of: **A***Eurysternuscontractus* Génier, 2009 **B***Eurysternusfoedus* Guérin-Méneville, 1830 **C***Eurysternushamaticollis* Balthasar, 1939 **D***Eurysternushypocrita* Balthasar, 1939.

**Plate 31.**
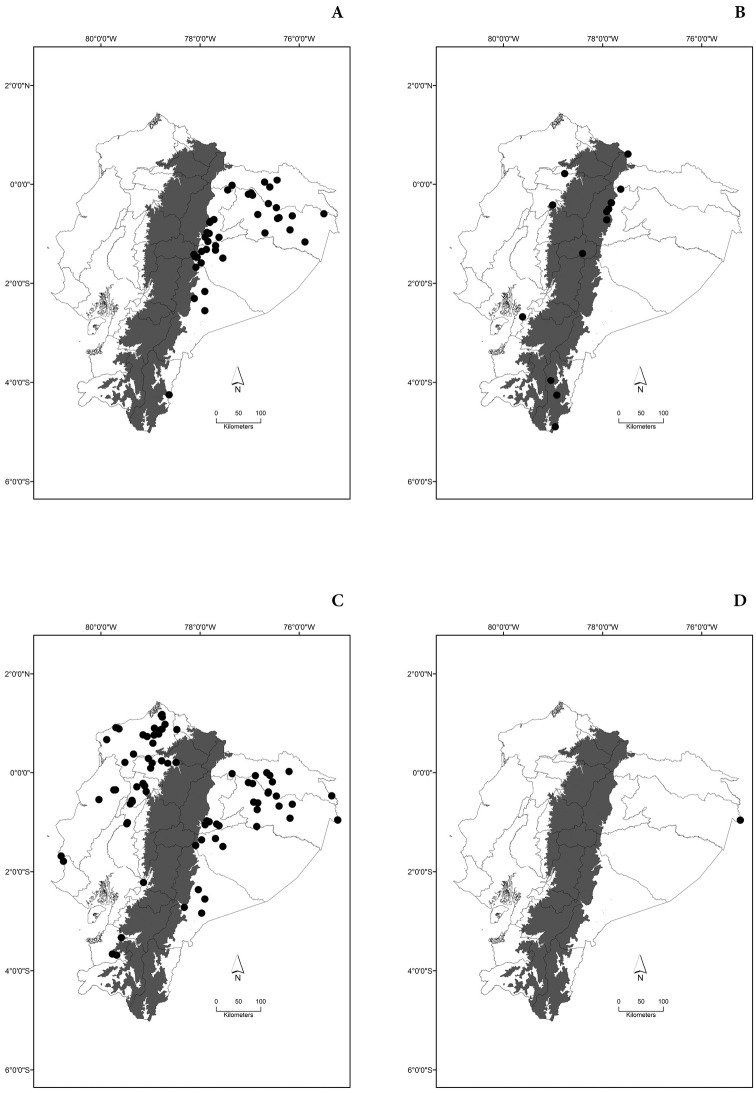
Distribution of: **A***Eurysternuslanuginosus* Génier, 2009 **B***Eurysternusmarmoreus* Castelnau, 1840 **C***Eurysternusplebejus* Harold, 1880 **D***Eurysternussquamosus* Génier, 2009.

**Plate 32.**
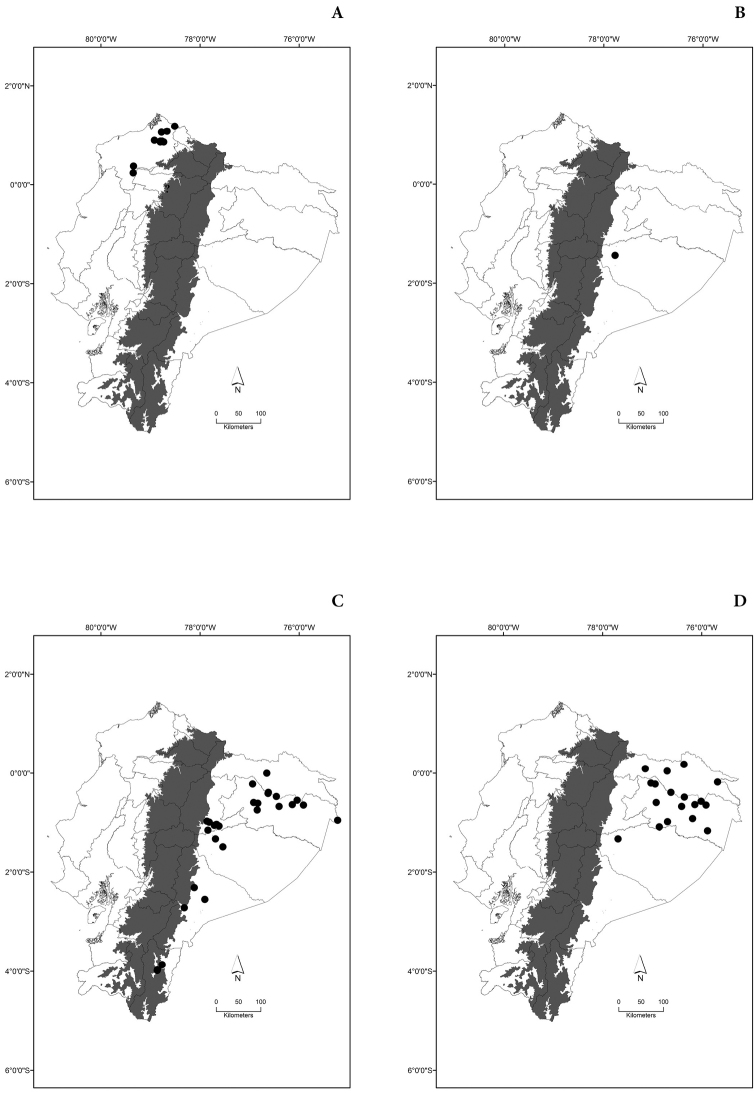
Distribution of: **A***Eurysternusstreblus* Génier, 2009 **B***Eurysternusstrigilatus* Génier, 2009 **C***Eurysternusvastiorum* Martínez, 1988 **D***Eurysternuswittmerorum* Martínez, 1988.

**Plate 33.**
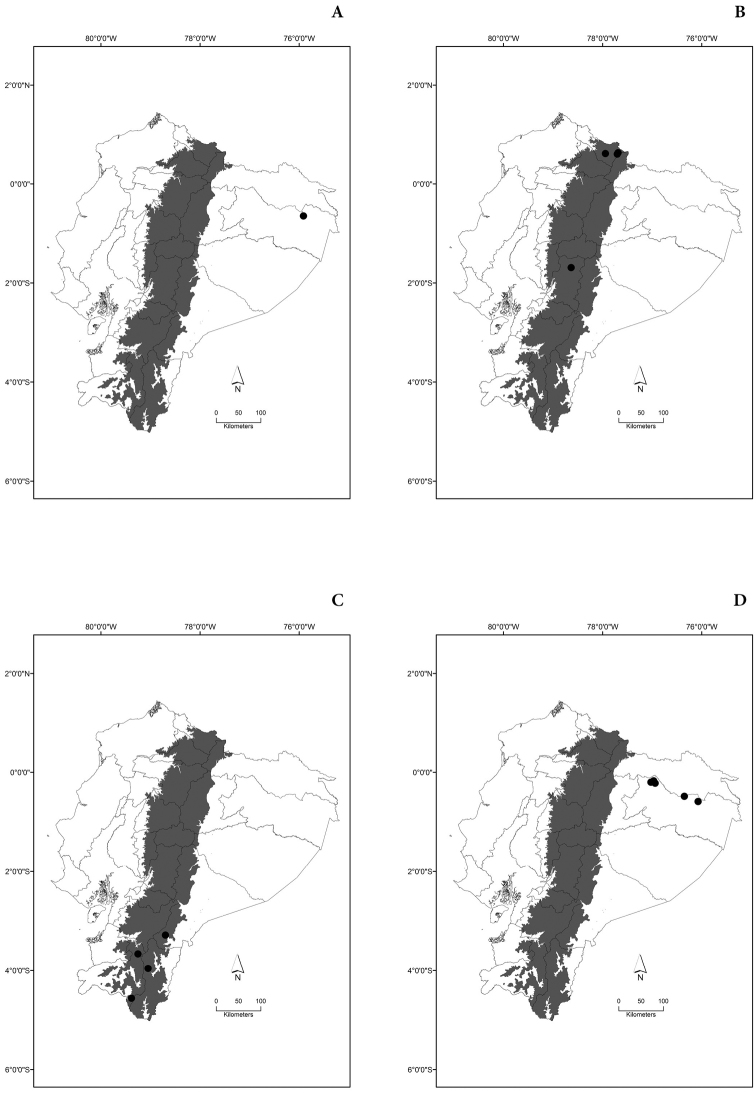
Distribution of: **A***Gromphasaeruginosa* (Perty, 1830) **B***Homocoprisachamas* (Harold, 1867) **C***Homocoprisbuckleyi* (Waterhouse, 1891) **D**Malagoniella (Malagoniella) astyanaxpolita Halffter, Pereira & Martínez, 1960.

**Plate 34.**
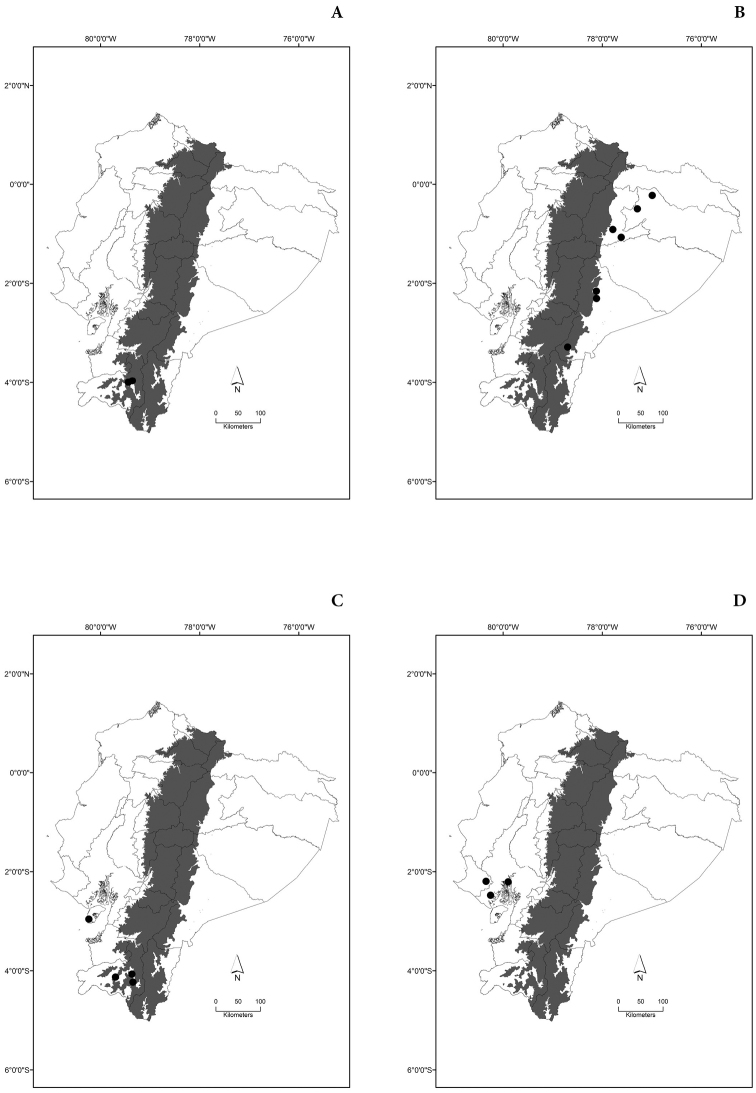
Distribution of: **A**Malagoniella (Megatophomima) cupreicollis (Waterhouse, 1890) **B***Megatharsisbuckleyi* Waterhouse, 1891 **C***Onoreidiumcristatum* (Arrow, 1931) **D***Onoreidiumhowdeni* (Ferreira & Galileo, 1993).

**Plate 35.**
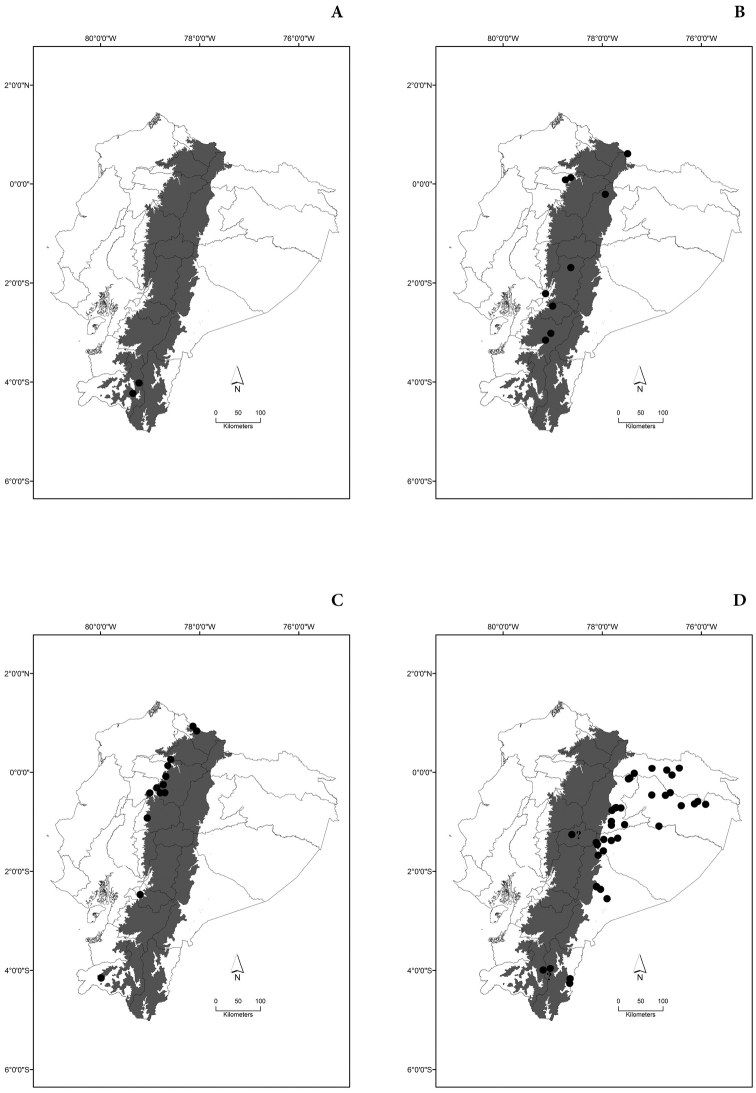
Distribution of: **A***Onoreidiumohausi* (Arrow, 1931) **B**Ontherus (Caelontherus) aequatorius Bates, 1891 **C**Ontherus (Caelontherus) compressicornis Luederwaldt, 1931 **D**Ontherus (Caelontherus) diabolicus Génier, 1996.

**Plate 36.**
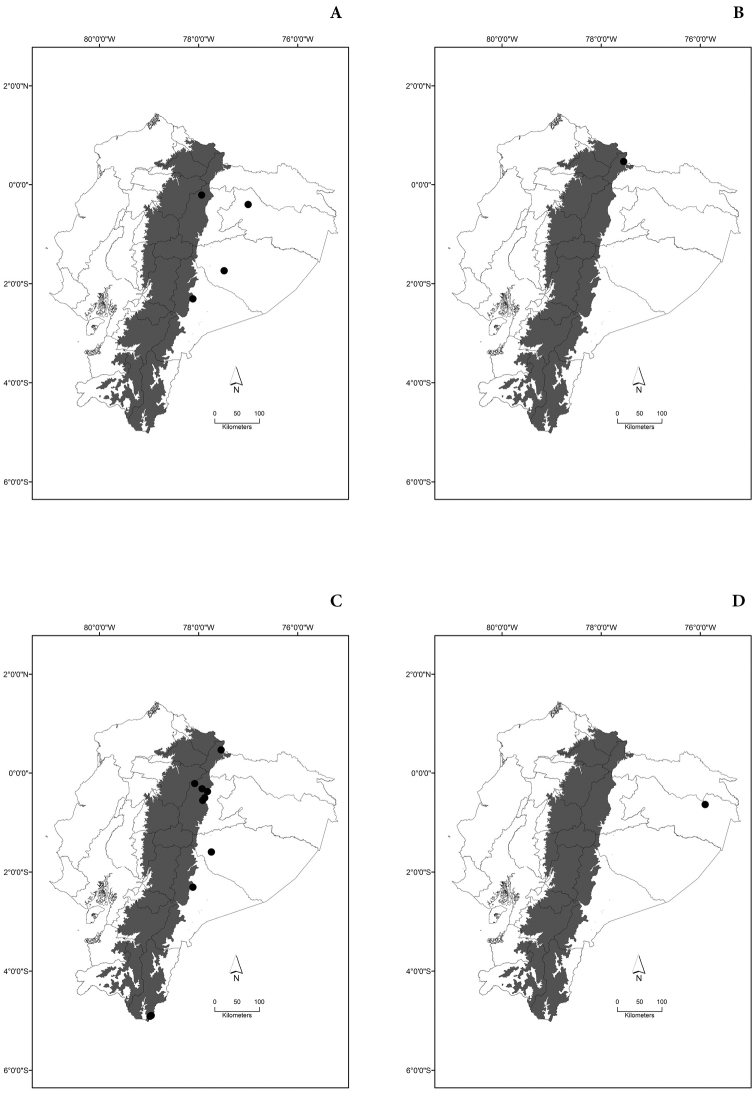
Distribution of: **A**Ontherus (Caelontherus) hadros Génier, 1996 **B**Ontherus (Caelontherus) howdeni Génier, 1996 **C**Ontherus (Caelontherus) incisus (Kirsch, 1871) **D**Ontherus (Caelontherus) laminifer Balthasar, 1938.

**Plate 37.**
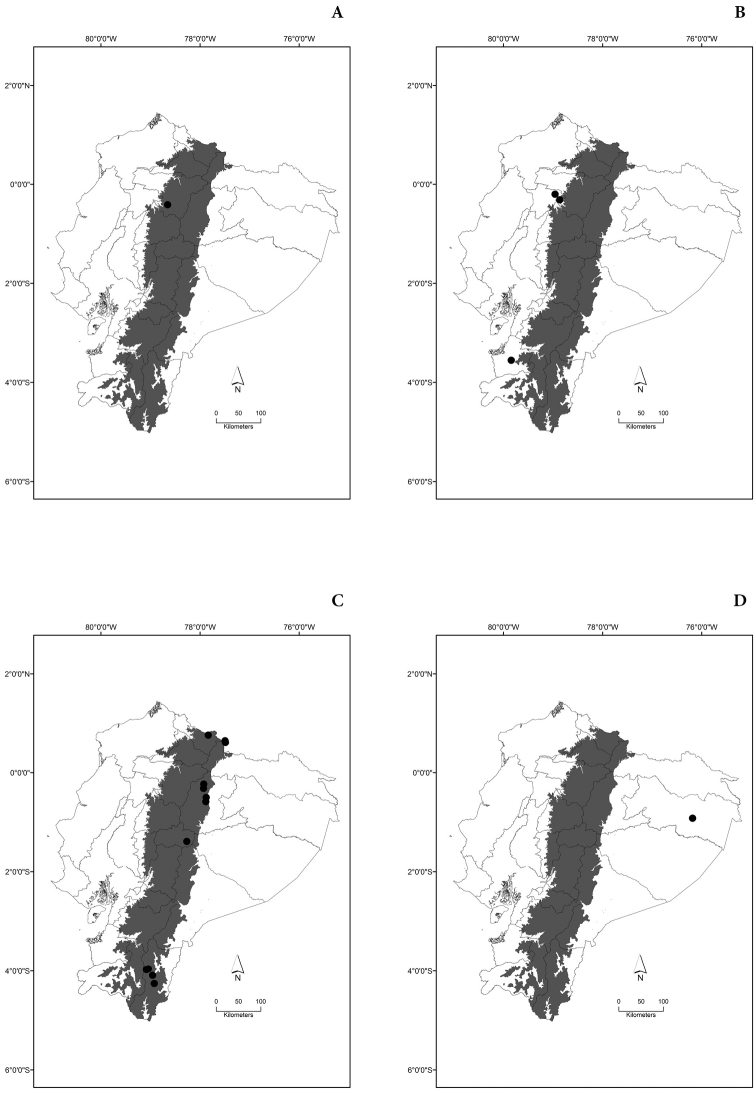
Distribution of: **A**Ontherus (Caelontherus) magnus Génier, 1996 **B**Ontherus (Caelontherus) pilatus Génier, 1996 **C**Ontherus (Caelontherus) politus Génier, 1996 **D**Ontherus (Caelontherus) tenustriatus Génier, 1996.

**Plate 38.**
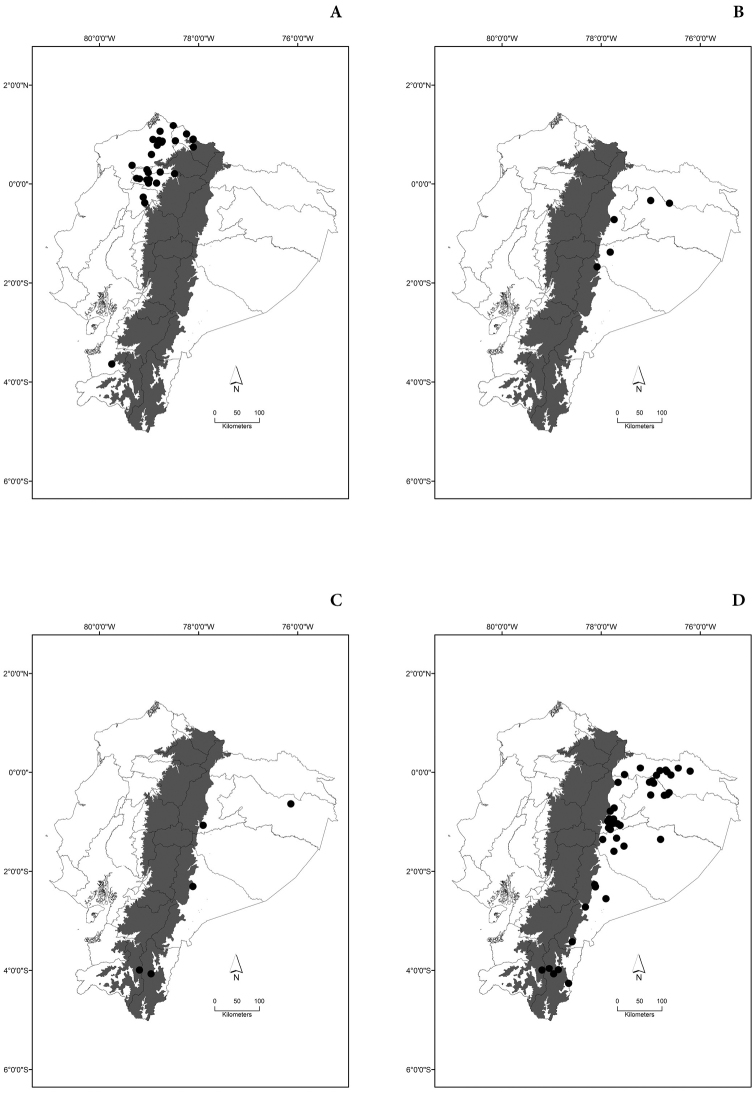
Distribution of: **A**Ontherus (Caelontherus) trituberculatus Balthasar, 1938 **B**Ontherus (Ontherus) azteca Harold, 1869 **C**Ontherus (Ontherus) edentulus Génier, 1996 **D**Ontherus (Ontherus) pubens Génier, 1996.

**Plate 39.**
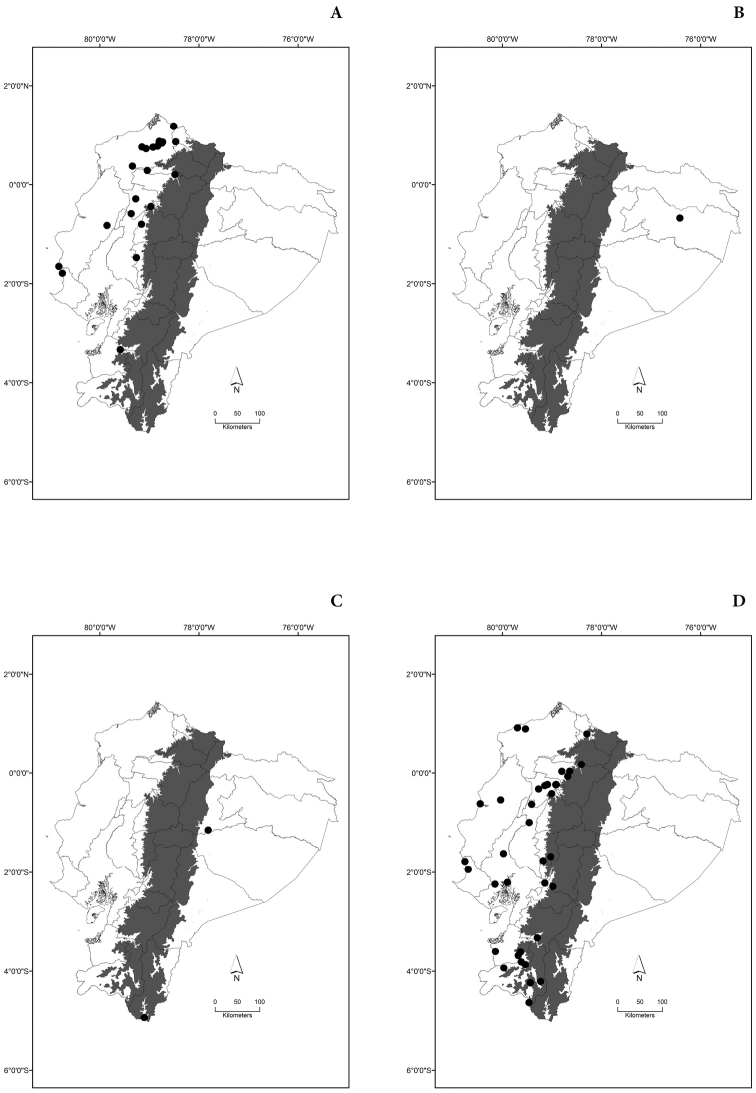
Distribution of: **A**Onthophagus (Onthophagus) acuminatus Harold, 1880 **B**Onthophagus (Onthophagus) basicarinatus Rossini, Vaz-de-Mello & Zunino, 2018 **C**Onthophagus (Onthophagus) bidentatus Drapiez, 1819 **D**Onthophagus (Onthophagus) confusus Boucomont, 1932.

**Plate 40.**
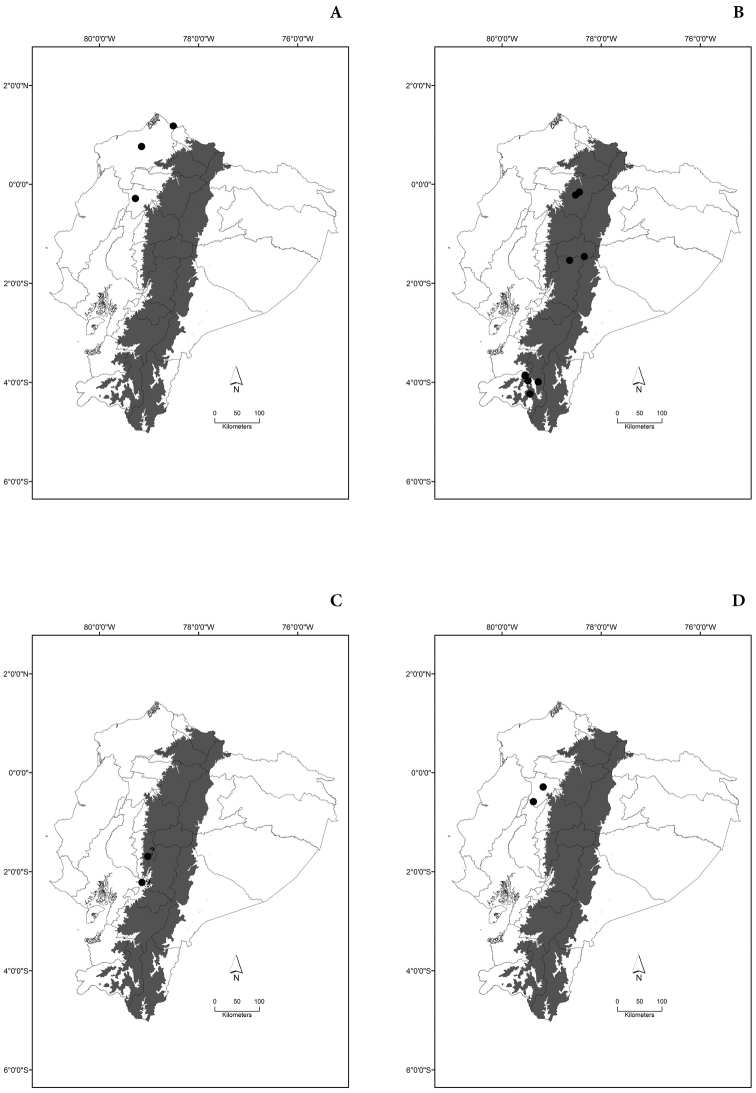
Distribution of: **A**Onthophagus (Onthophagus) coscineus Bates, 1887 **B**Onthophagus (Onthophagus) curvicornis Latreille, 1811 **C**Onthophagus (Onthophagus) cyanellus Bates, 1887 **D**Onthophagus (Onthophagus) dicranius Bates, 1887.

**Plate 41.**
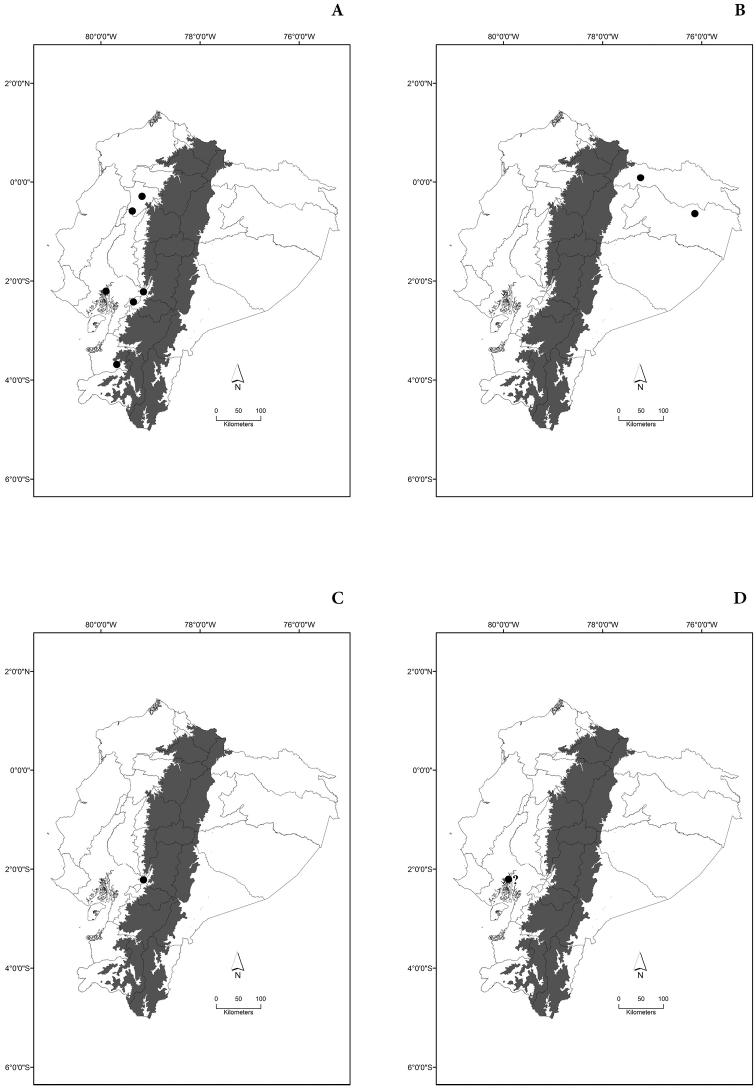
Distribution of: **A**Onthophagus (Onthophagus) dicranoides Balthasar, 1939 **B**Onthophagus (Onthophagus) digitifer Boucomont, 1932 **C**Onthophagus (Onthophagus) embrikianus Paulian, 1936 **D**Onthophagus (Onthophagus) insularis Boheman, 1858.

**Plate 42.**
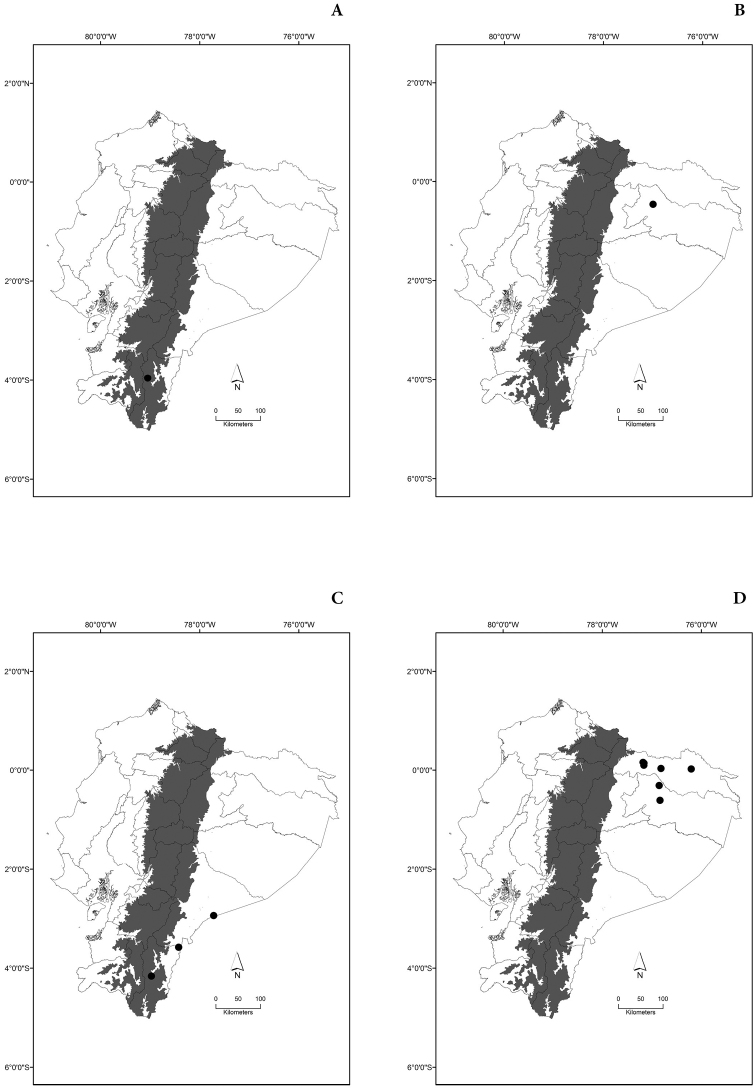
Distribution of: **A**Onthophagus (Onthophagus) lojanus Balthasar, 1939 **B**Onthophagus (Onthophagus) marginicollis Harold, 1880 **C**Onthophagus (Onthophagus) mirabilis Bates, 1887 **D**Onthophagus (Onthophagus) onorei Zunino & Halffter, 1997.

**Plate 43.**
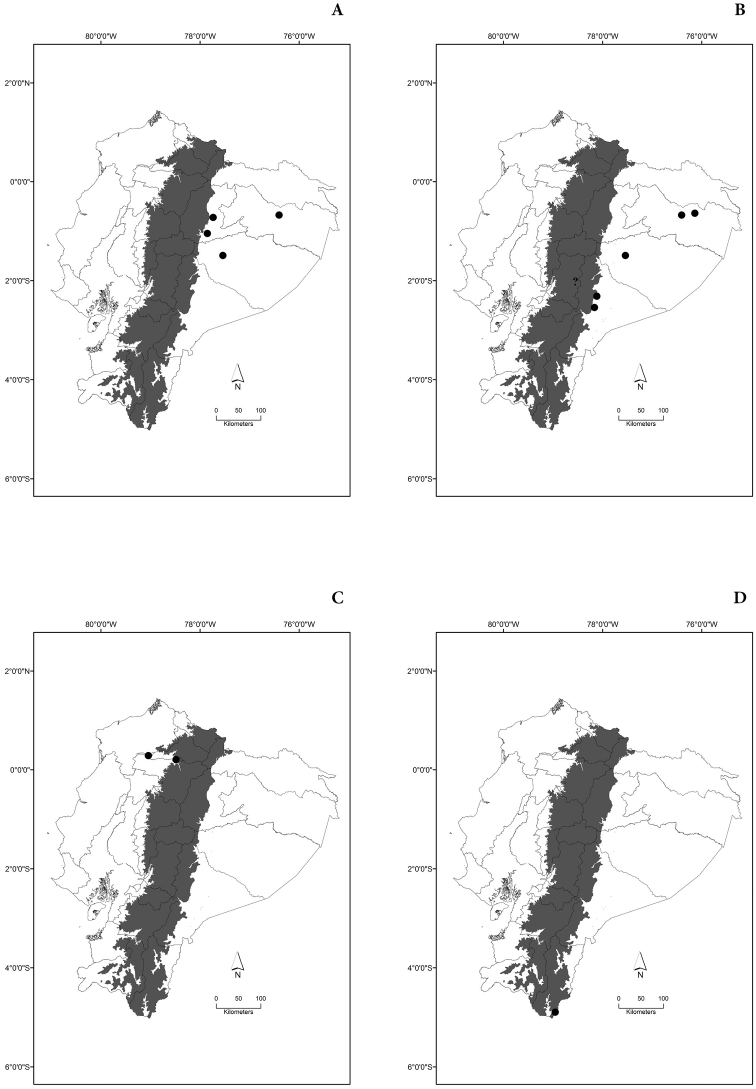
Distribution of: **A**Onthophagus (Onthophagus) osculatii Guérin-Méneville, 1855 **B**Onthophagus (Onthophagus) rubrescens Blanchard, 1843 **C**Onthophagus (Onthophagus) sharpi Harold, 1875 **D**Onthophagus (Onthophagus) steinheili Harold, 1880.

**Plate 44.**
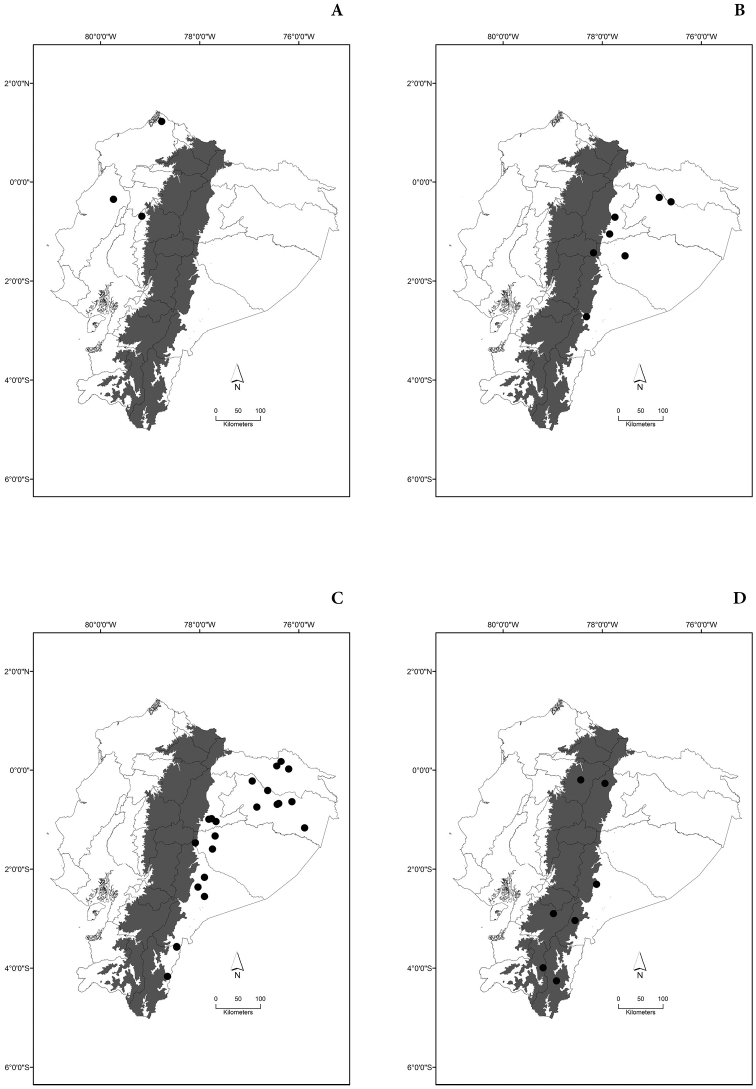
Distribution of: **A**Onthophagus (Onthophagus) stockwelli Howden & Young, 1981 **B**Onthophagus (Onthophagus) transisthmius Howden & Young, 1981 **C**Onthophagus (Onthophagus) xanthomerus Bates, 1887 **D***Oruscatusopalescens* Bates, 1870.

**Plate 45.**
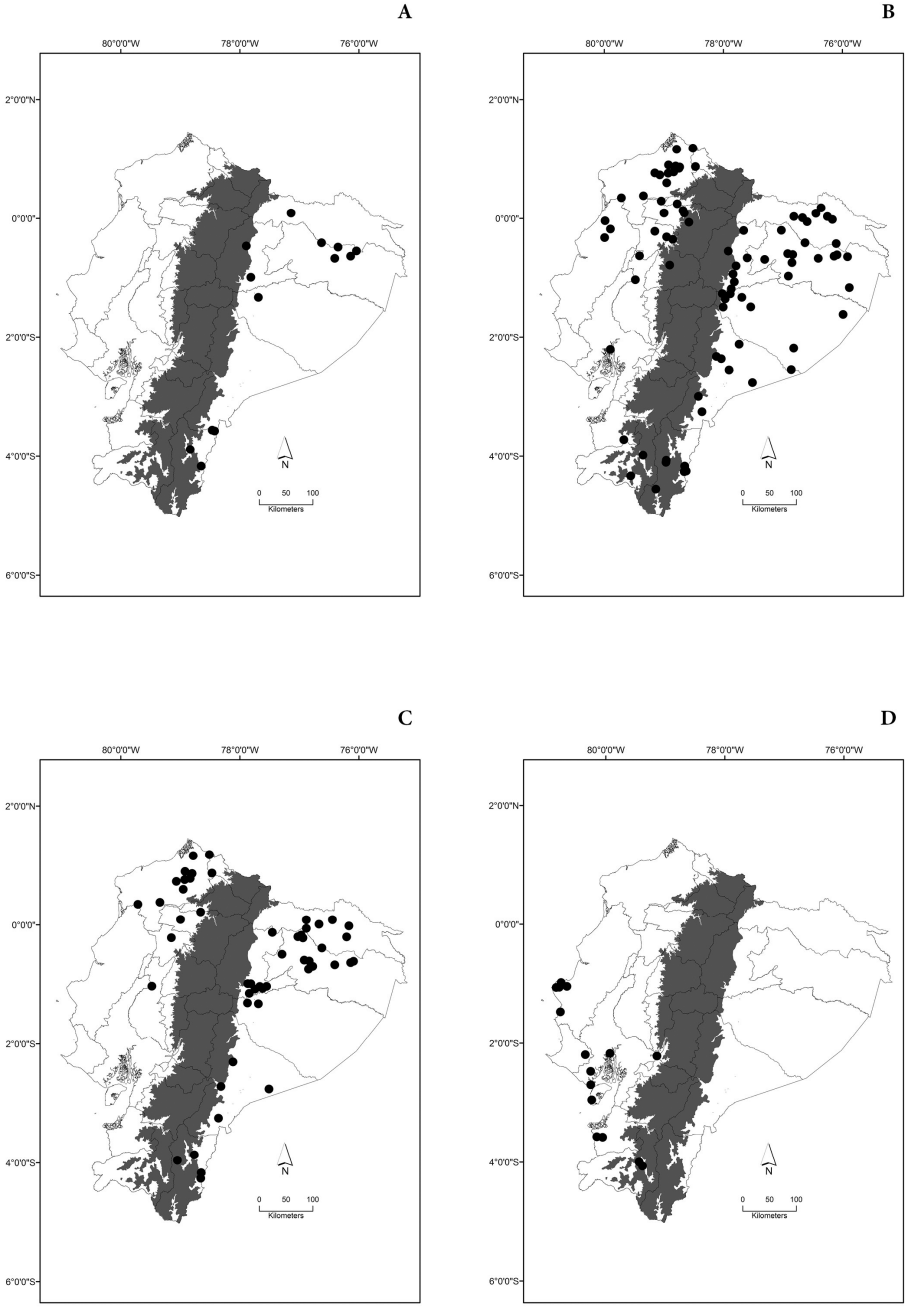
Distribution of: **A**Oxysternon (Mioxysternon) spiniferum Laporte, 1840 **B**Oxysternon (Oxysternon) conspicillatum (Weber, 1801) **C**Oxysternon (Oxysternon) silenussmaragdinum d’Olsoufieff, 1924 **D**Phanaeus (Notiophanaeus) achilles Boheman, 1858.

**Plate 46.**
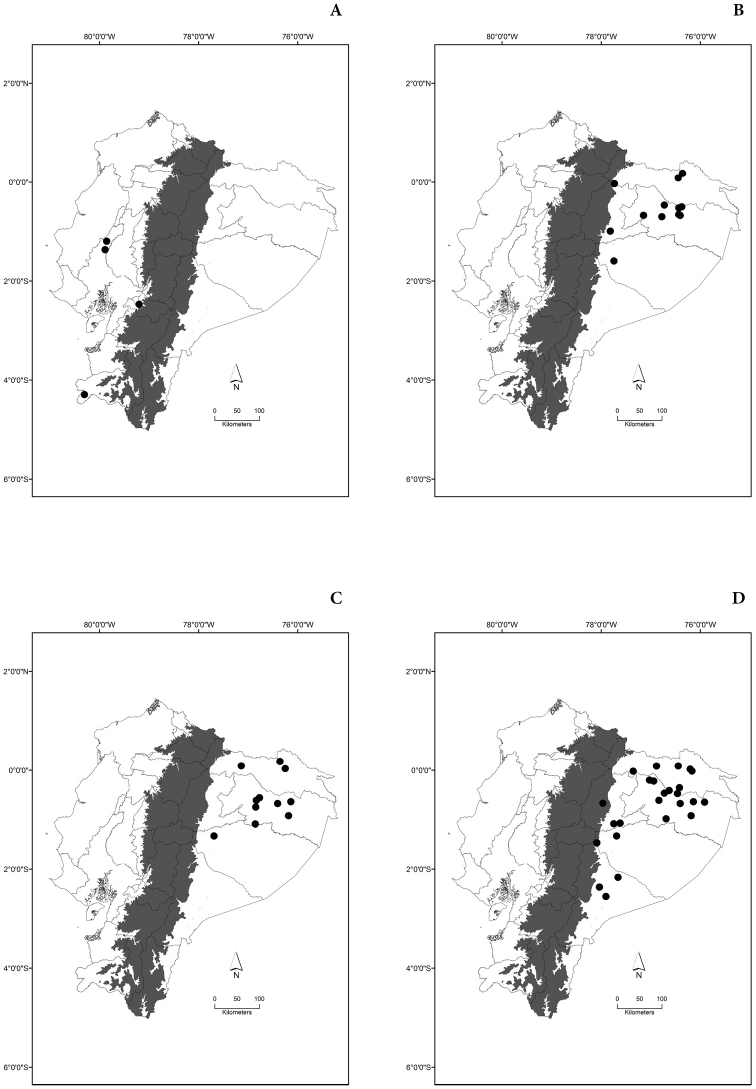
Distribution of: **A**Phanaeus (Notiophanaeus) arletteae Arnaud, 2018 **B**Phanaeus (Notiophanaeus) bispinus Bates, 1868 **C**Phanaeus (Notiophanaeus) cambeforti Arnaud, 1982 **D**Phanaeus (Notiophanaeus) chalcomelas (Perty, 1830).

**Plate 47.**
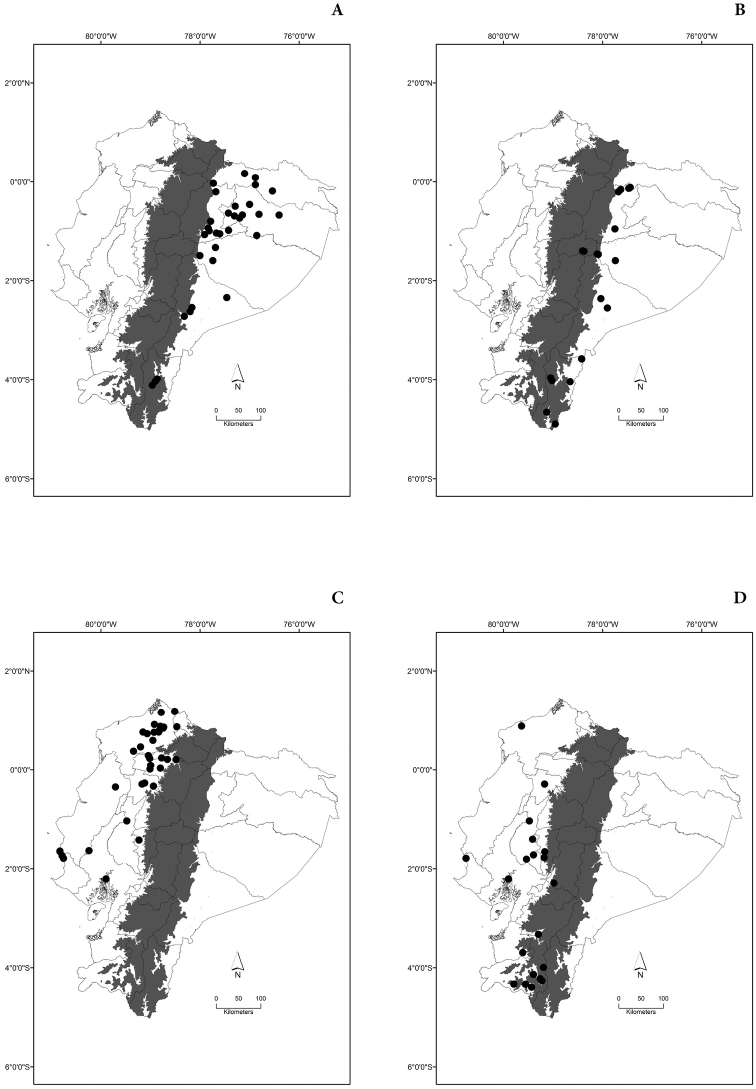
Distribution of: **A**Phanaeus (Notiophanaeus) haroldi Kirsch, 1871 **B**Phanaeus (Notiophanaeus) meleagris Blanchard, 1843 **C**Phanaeus (Notiophanaeus) pyrois Bates, 1887 **D**Phanaeus (Phanaeus) lunaris Taschenberg, 1870.

**Plate 48.**
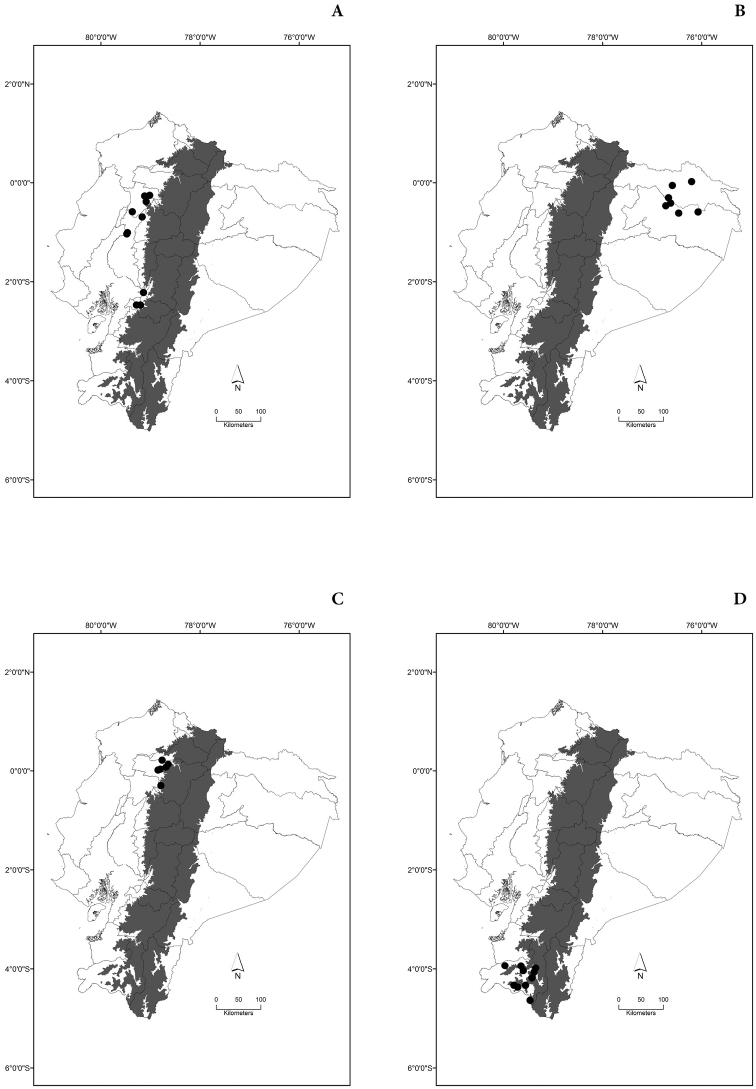
Distribution of: **A***Scatimuscribrosus* Génier & Kohlmann, 2003 **B***Scatimusfernandezi* Martínez, 1988 **C***Scatimusfurcatus* Balthasar, 1939 **D***Scatimusmonstrosus* Balthasar, 1939.

**Plate 49.**
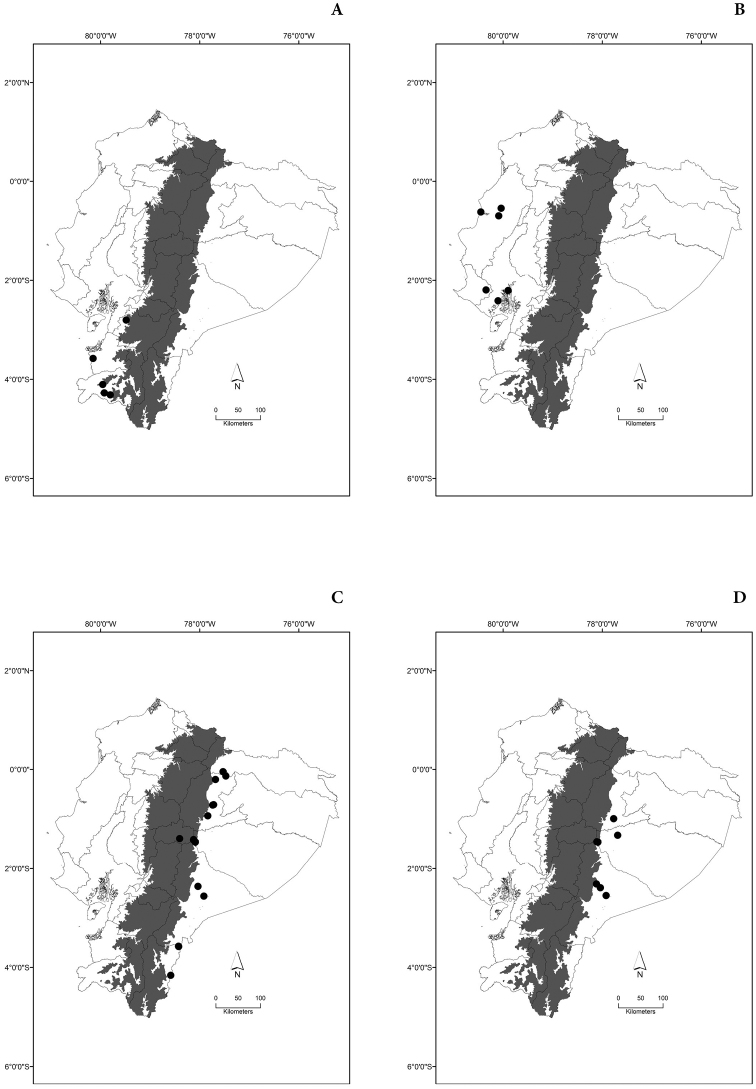
Distribution of: **A***Scatimusonorei* Génier & Kohlmann, 2003 **B***Scatimuspacificus* Génier & Kohlmann, 2003 **C***Scatimusstrandi* Balthasar, 1939 **D***Scybalocanthonkaestneri* (Balthasar, 1939).

**Plate 50.**
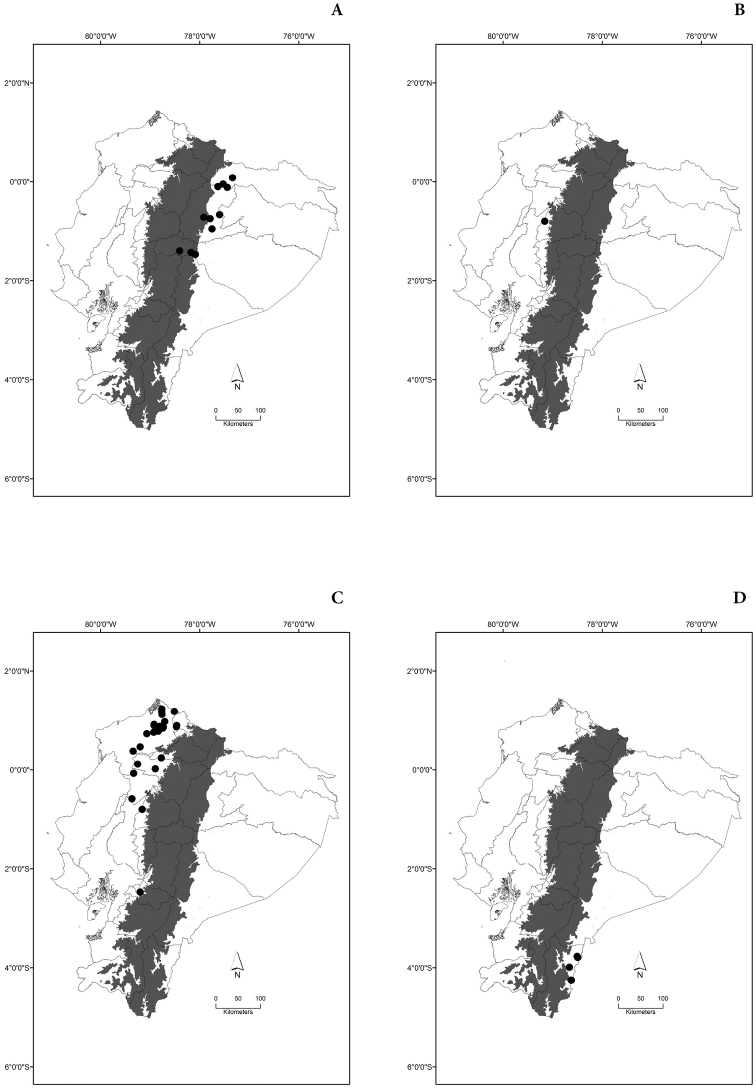
Distribution of: **A***Scybalocanthonmaculatus* (Schmidt, 1920) **B***Scybalocanthonmoniliatus* (Bates, 1887) **C***Scybalocanthontrimaculatus* (Schmidt, 1922) **D***Streblopuspunctatus* Balthasar, 1938.

**Plate 51.**
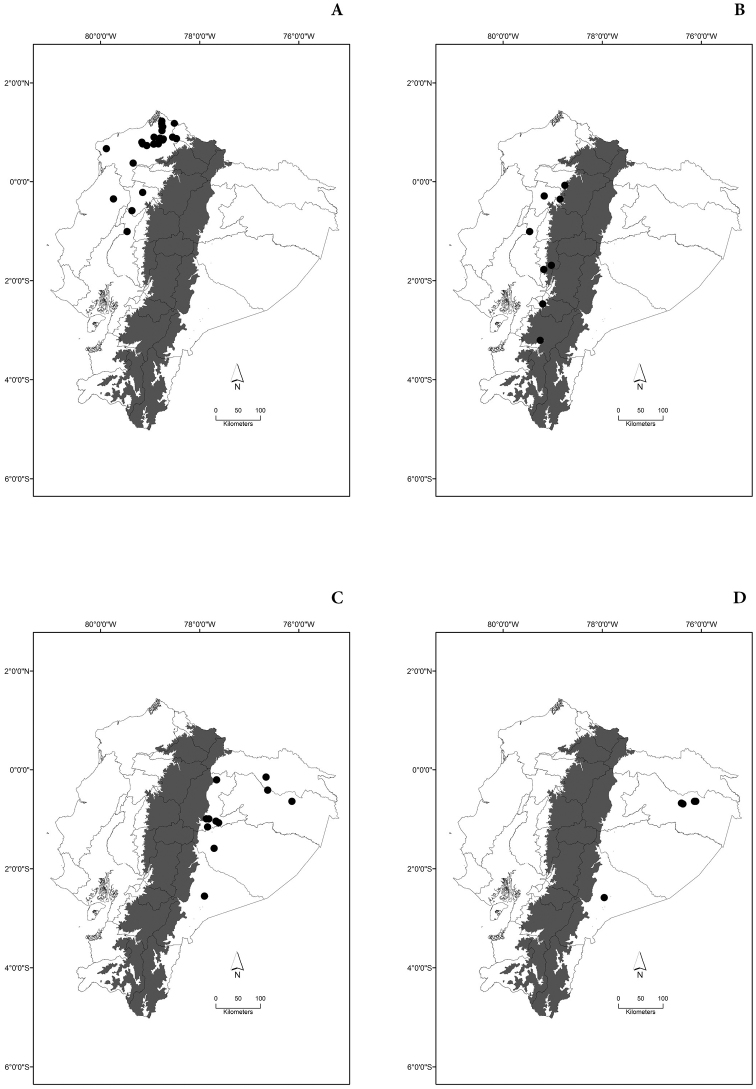
Distribution of: **A***Sulcophanaeusmiyashitai* Arnaud, 2002 **B***Sulcophanaeusvelutinus* (Murray, 1856) **C***Sylvicanthonbridarollii* Martínez, 1948 **D***Sylvicanthonedmonsi* Cupello & Vaz-de-Mello, 2018.

**Plate 52.**
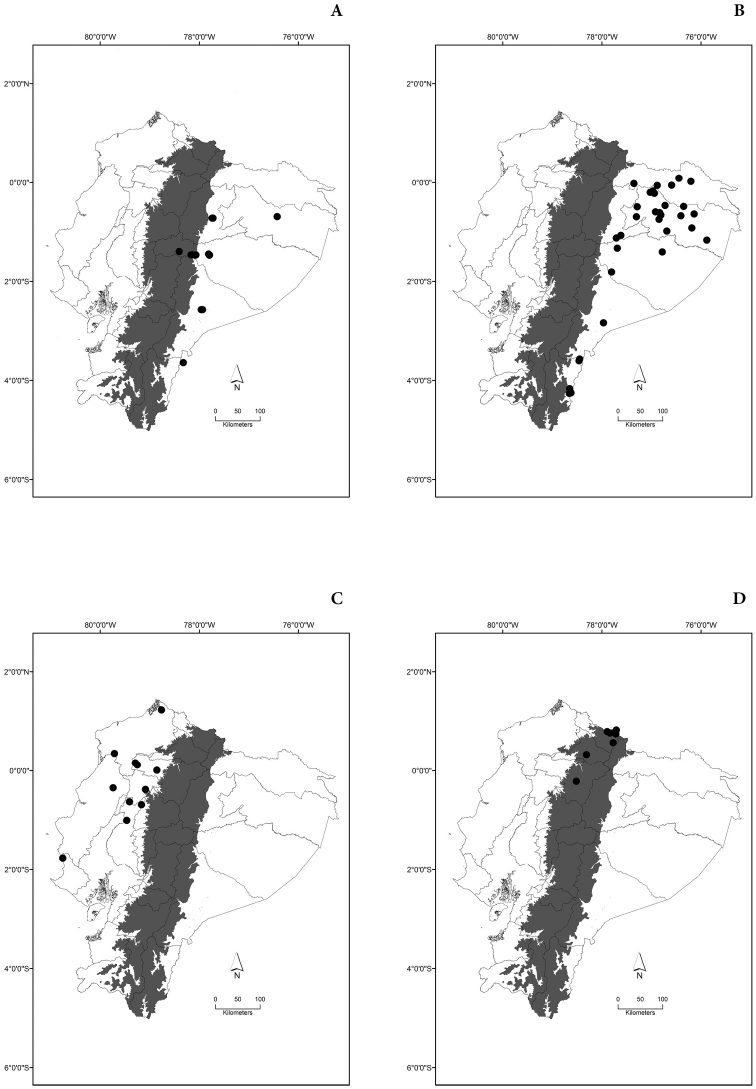
Distribution of: **A***Sylvicanthongenieri* Cupello & Vaz-de-Mello, 2018 **B***Sylvicanthonproseni* (Martínez, 1948) **C***Trichillidiumpilosum* (Robinson, 1948) **D***Uroxyselongatus* Harold, 1868.

**Plate 53.**
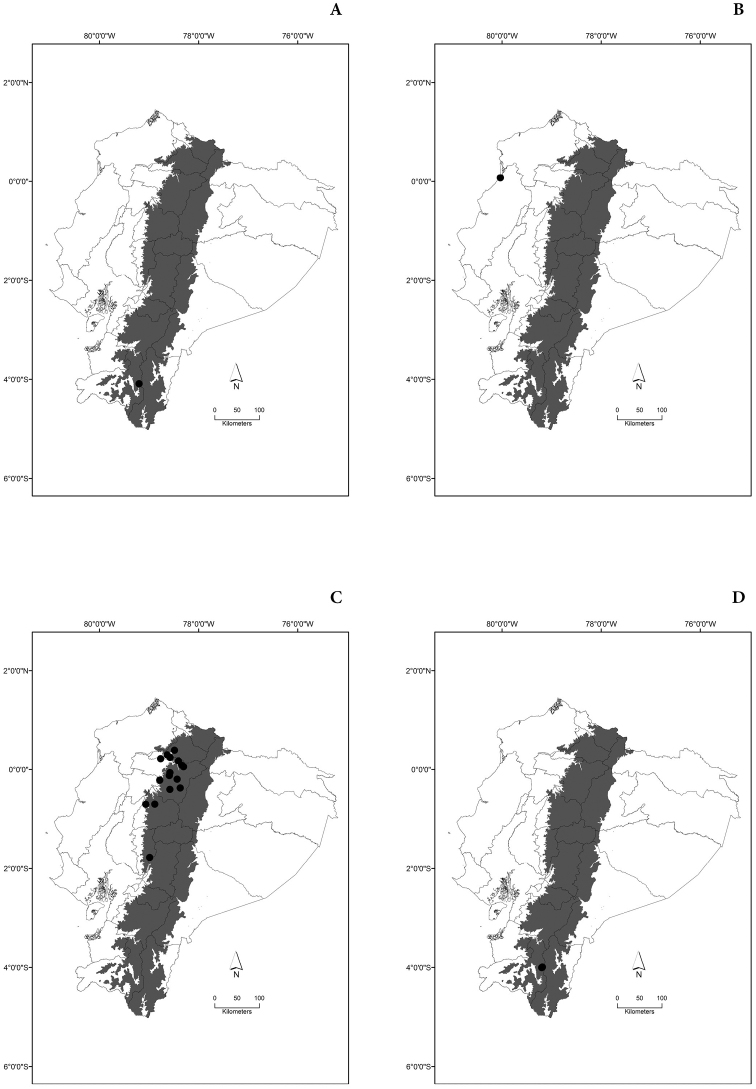
Distribution of: **A***Uroxysfrankenbergeri* Balthasar, 1940 **B***Uroxysgorgon* Arrow, 1933 **C***Uroxyslatesulcatus* Bates, 1891 **D***Uroxyslojanus* Arrow, 1933.

**Plate 54.**
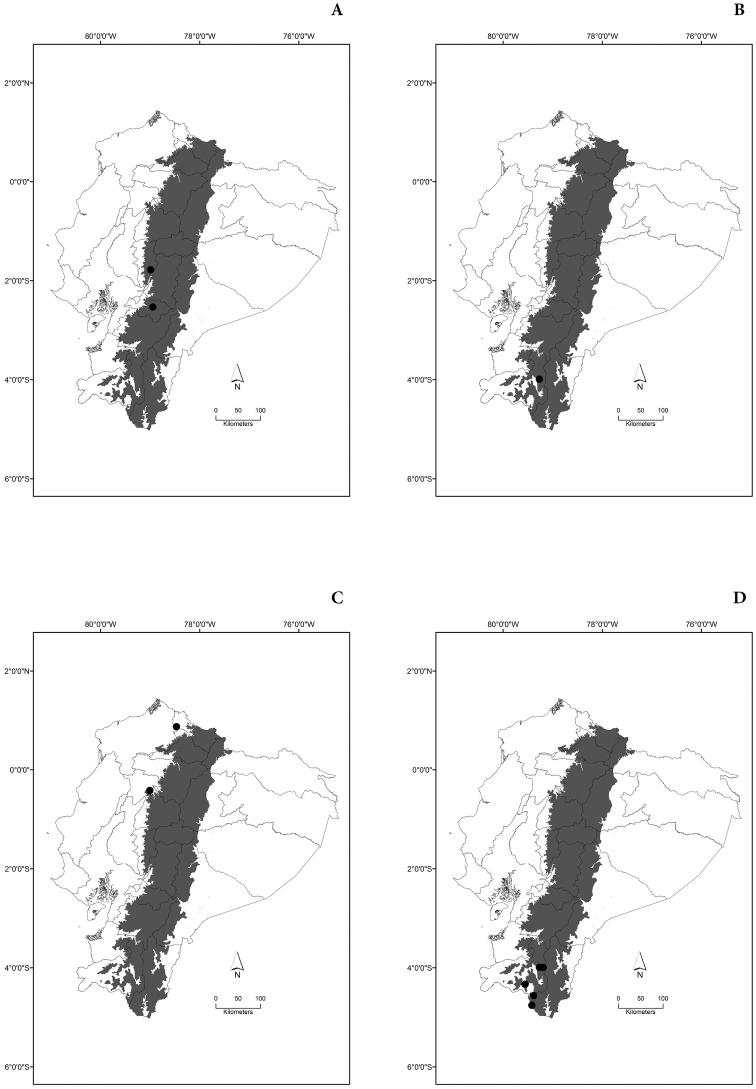
Distribution of: **A***Uroxysmonstruosus* Balthasar, 1940 **B***Uroxysohausi* (Balthasar, 1938) **C***Uroxyspauliani* Balthasar, 1940 **D***Uroxysrugatus* Boucumont, 1928.

**Plate 55.**
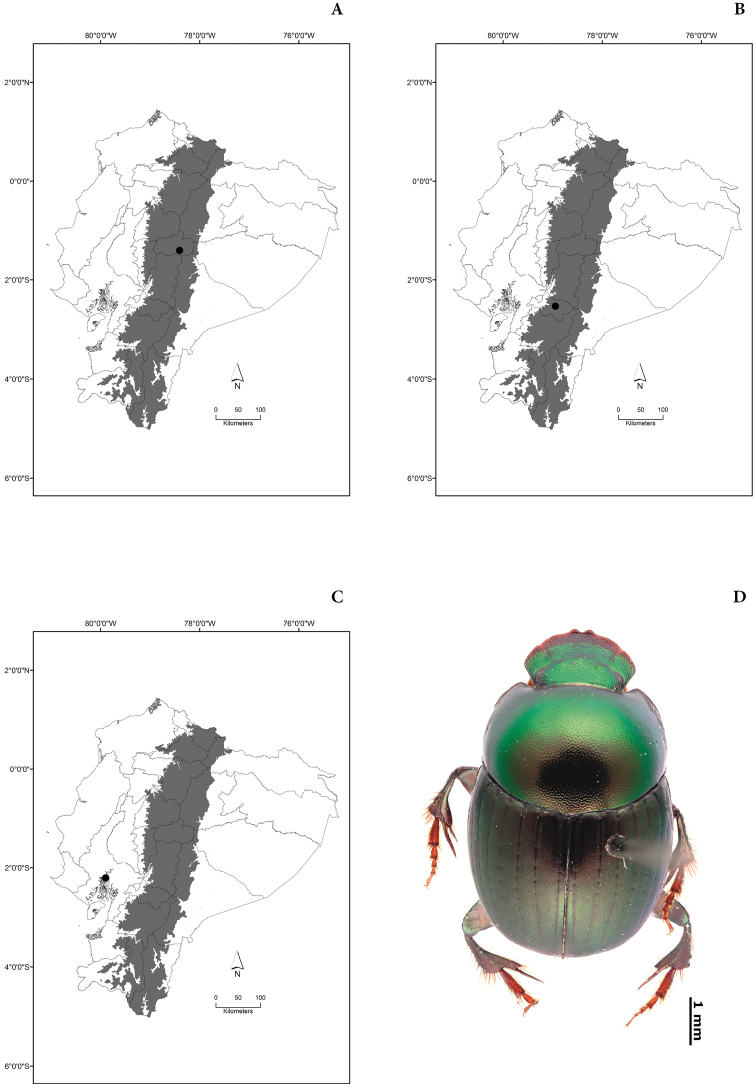
Distribution of: **A***Uroxysspaethi* Balthasar, 1940 **B***Uroxyssulcicollis* Harold, 1880 **C***Uroxyssulai* Balthasar, 1940 **D** Lectotype (♂, here designated) of *Choeridiumorbiculatum* Lucas, 1857.

**Plate 56.**
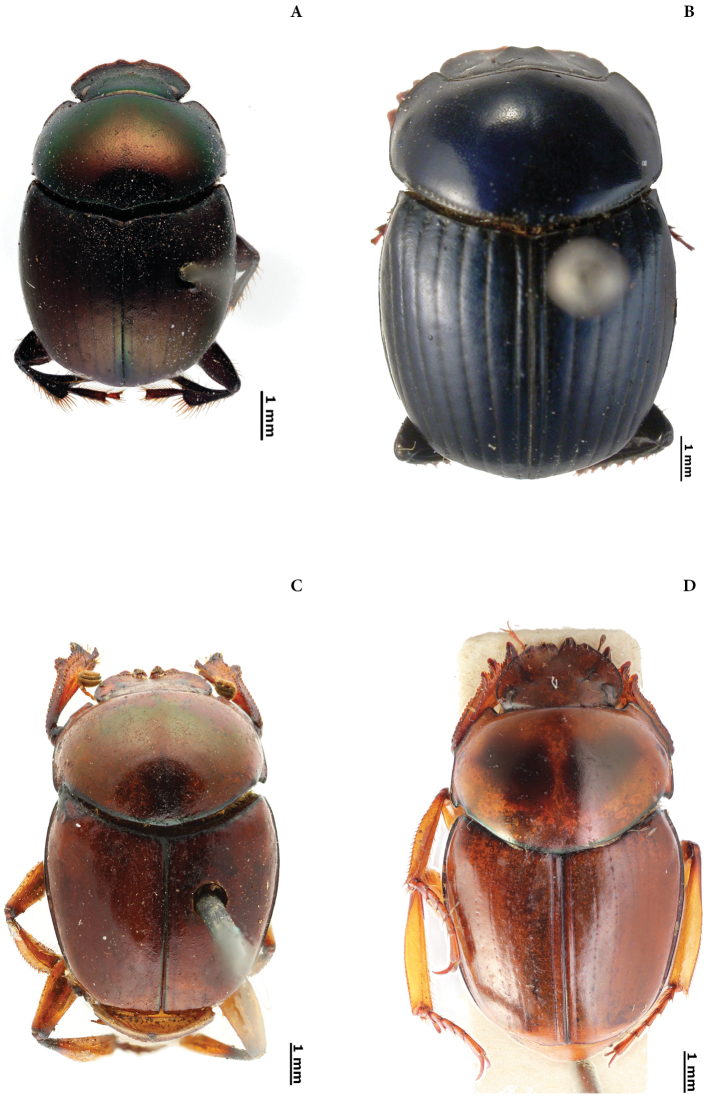
**A** Lectotype (♂, here designated) of *Choeridiumcupreum* Blanchard, 1846 **B** Lectotype (♂, here designated) of *Canthidiumcoerulescens* Balthasar, 1939 **C** Lectotype (♂, here designated) of *Canthonangustatus* Harold, 1867 **D** Lectotype (♂, here designated) of *Canthonangustatusohausi* Balthasar, 1939.

**Plate 57.**
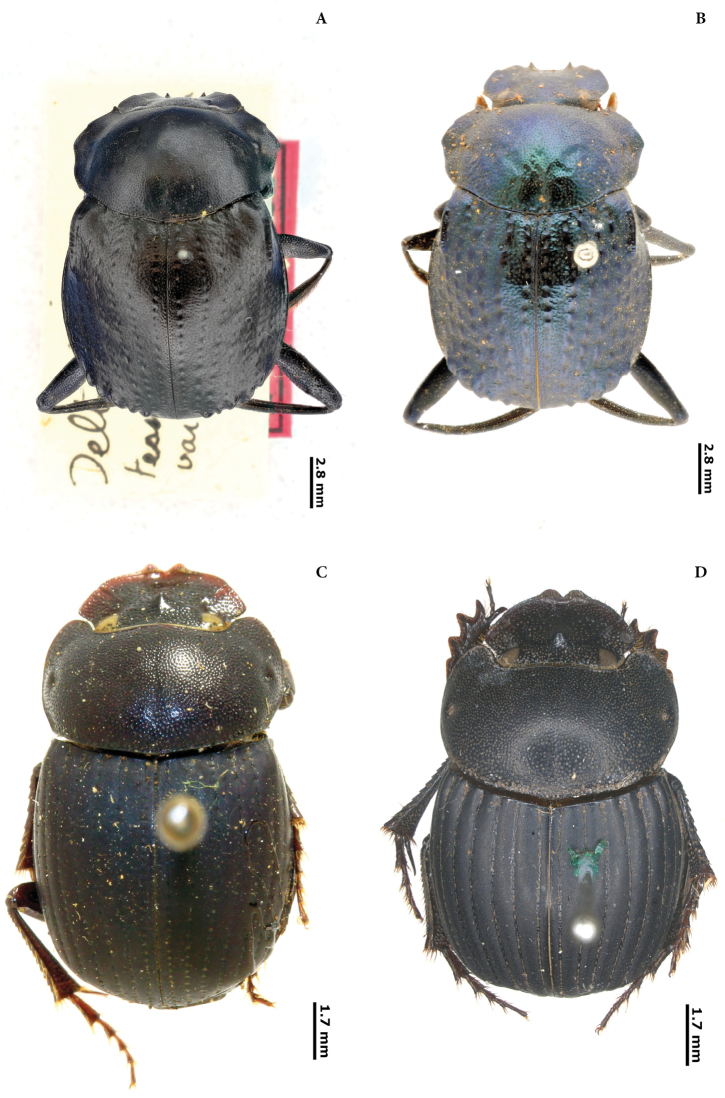
**A** Holotype (♂) of Deltochilumtessellatumvar.arrowi Paulian, 1939 **B** Lectotype (♂, here designated) of *Deltochilumtessellatum* Bates, 1870 **C** Lectotype (♂, here designated) of *Pinotusfortepunctatus* Luederwaldt, 1923 **D** Lectotype (♂, here designated) of *Pinotusglobulus* Felsche, 1901.

**Plate 58.**
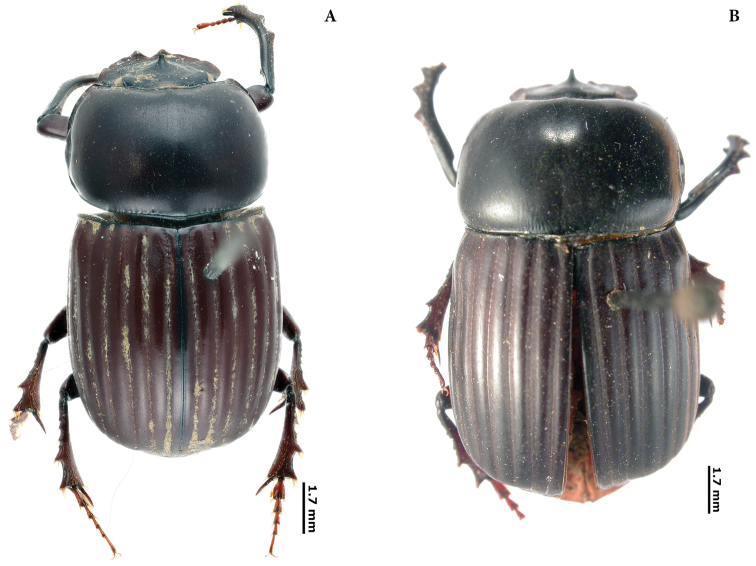
**A** Lectotype (♂, here designated) of *Uroxyslatesulcatus* Bates, 1891 **B** Lectotype (♂, here designated) of *Uroxysmagnus* Balthasar, 1940.
